# Modular allylation of C(sp^3^)–H bonds by combining decatungstate photocatalysis and HWE olefination in flow†

**DOI:** 10.1039/d2sc01581a

**Published:** 2022-05-31

**Authors:** Luca Capaldo, Stefano Bonciolini, Antonio Pulcinella, Manuel Nuño, Timothy Noël

**Affiliations:** Flow Chemistry Group, Van’t Hoff Institute for Molecular Sciences (HIMS), University of Amsterdam Science Park 904 1098 XH Amsterdam The Netherlands t.noel@uva.nl www.NoelResearchGroup.com; Vapourtec Ltd, Park Farm Business Centre Fornham St Genevieve Bury St Edmunds Suffolk IP28 6TS UK

## Abstract

The late-stage introduction of allyl groups provides an opportunity to synthetic organic chemists for subsequent diversification, furnishing a rapid access to new chemical space. Here, we report the development of a modular synthetic sequence for the allylation of strong aliphatic C(sp^3^)–H bonds. Our sequence features the merger of two distinct steps to accomplish this goal, including a photocatalytic Hydrogen Atom Transfer and an ensuing Horner–Wadsworth–Emmons (HWE) reaction. This practical protocol enables the modular and scalable allylation of valuable building blocks and has been applied to structurally complex molecules.

## Abstract 2

We report a flow platform for the modular allylation of strong aliphatic C(sp^3^)–H bonds based on the merger of photocatalytic HAT and a HWE reaction. This approach enables both early- and late-stage diversification of various hydroalkanes.

## Graphical abstract

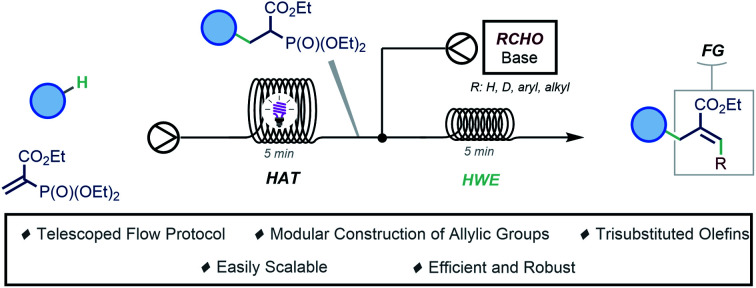

Modern drug discovery programs capitalize increasingly on the application of late-stage functionalization methodologies to accelerate the lead optimization phase.^1,2^ Such strategies allow for the rapid and cost-efficient^3,4^ diversification of the parent molecule by exploiting native functionalities (*e.g.*, C–H bonds), thus effectively avoiding the need to redesign its entire synthetic route to access new leads.^5–7^ More specifically, the late-stage decoration of organic molecules with multipurpose functional groups would provide new points of entry for subsequent diversification.^8^ Such a strategy could be particularly convenient when it is realized *via* a chemo- and regioselective functionalization of C–H bonds in the absence of any proximal directing or activating groups.^7^ However, while C(sp^2^)–H activation has been extensively investigated, the direct functionalization of C(sp^3^)–H bonds remains challenging and is often narrow in scope.^9^ Recently, photocatalytic Hydrogen Atom Transfer (HAT) has been exploited to enable the late-stage functionalization of C(sp^3^)–H bonds, showing remarkable levels of regioselectivity even in complex molecules (Scheme 1A).^10^ In HAT photocatalysis, a catalyst converts light energy into chemical energy for the homolytic cleavage of strong aliphatic C–H bonds.

**Scheme 1:**
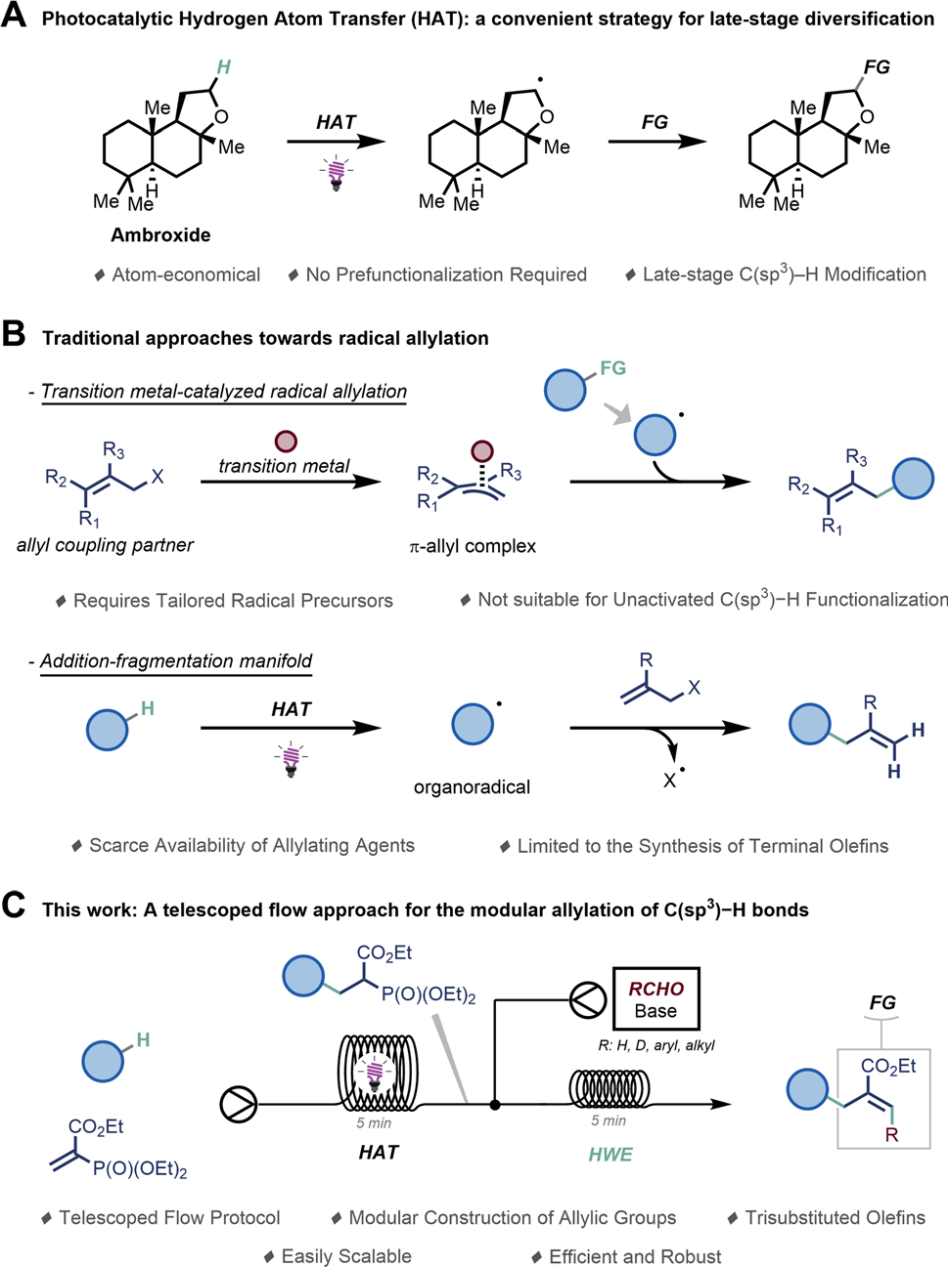
Allylation of C(sp^3^)–H bonds. (A) Photocatalytic HAT enables late-stage functionalization of structurally complex molecules. (B) Reported approaches for the photocatalyzed radical allylation of organic molecules. (C) A telescoped approach for the modular allylation of C(sp^3^)–H bonds (this work).

Especially, the decatungstate anion ([W_10_O_32_]^4−^) has shown remarkable selectivity for specific C(sp^3^)–H bonds, governed by an intricate balance between steric and electronic interactions.^9,11,12^

We envisioned that the regioselective introduction of an allyl moiety onto hydrocarbon frameworks would be particularly useful as it provides a convenient branching point for further late-stage synthetic exploitation.^13^ To install such moieties, radical allylation has manifested itself as a valuable strategy. One approach relies on the use of transition metal complexes to activate a substrate containing an allylic leaving group to afford a π-allyl complex, which is then suited to trap a *C*-centered radical (Scheme 1B).^14^ This strategy can engage a diverse set of allyl coupling partners but typically requires purposely designed radical precursors, which prevents the direct allylation of unactivated C(sp^3^)–H bonds.

Another tactic exploits radicofugal groups X (*e.g.*, X = halide, SO_2_R, SnR_3_, *etc*) in the allylic position to afford the desired product *via* a radical addition–fragmentation process (Scheme 1B).^15–28^ However, while synthetically useful, this transformation is not suitable for the synthesis of densely functionalized allylic functionalities.

Seeking to address these challenges, we sought to develop a robust and versatile synthetic platform for the allylation of strong aliphatic C(sp^3^)–H bonds. Hereto, a modular synthetic sequence is preferred in which the allylic moiety is assembled in a stepwise fashion, enabling the rapid generation of structurally diverse analogues. Specifically, our sequence features the merger of two distinct synthetic steps to accomplish this goal (Scheme 1C). First, we planned to activate C(sp^3^)–H bonds *via* decatungstate-catalyzed Hydrogen Atom Transfer^29,30^ and subsequently trap the resulting *C*-centered radical with a vinyl phosphonate.^31,32^ The ensuing radical addition product serves as a suitable linchpin for the second step, in which a classical Horner–Wadsworth–Emmons (HWE) olefination^33,34^ is able to deliver the targeted allylated compounds. In order to streamline these two steps, we reasoned that a telescoped flow protocol where the reactions are performed in tandem without the need for tedious purification of intermediates would be indispensable not only to accelerate access to these valuable building blocks but also to ensure facile scalability.^35–37^ Herein, we report the successful realization of a flow platform enabling the allylation of a wide range of unactivated hydrocarbons.

Our investigations commenced with the decatungstate-enabled hydroalkylation of ethyl 2-(diethoxyphosphoryl)acrylate (2) using cyclohexane as the H-donor (see ESI, Table S1†). Following a careful optimization of different reaction parameters, we found that the photocatalytic radical addition performed optimal in continuous-flow using a commercially available Vapourtec UV-150 photochemical reactor (PFA (perfluoroalkoxy) capillary, ID: 0.75 mm; *V* = 3.06 mL, flow rate = 0.612 mL min^−1^, *t*_r_ = 5 min) equipped with a 60 W UV-A LED light source (*λ* = 365 nm), which matches the measured absorption spectrum of decatungstate. A 65% NMR yield (64% after isolation) was obtained for the targeted hydroalkylated compound when a CH_3_CN solution of the acrylate (0.1 M), cyclohexane (20 equivalents) and tetrabutylammonium decatungstate (TBADT, (Bu_4_N)_4_[W_10_O_32_]) as the photocatalyst (1 mol%)^38–46^ was irradiated for 5 minutes (see ESI, Table S1, Entry 9†). Other HAT photocatalysts, such as Eosin Y,^47^ anthraquinone,^48^ 5,7,12,14-pentacenetetrone^28^ and 9-fluorenone^49^ were also evaluated, but failed to deliver the targeted product. Interestingly, benzophenone^50,51^ showed a comparable activity to the decatungstate anion, although only when used at high catalyst loading (20 mol%, 68% NMR yield). Due to the lower extinction coefficient of benzophenone compared to TBADT (<200 *vs.* 13 500 M^−1^ cm^−1^),^52,53^ and its known tendency to dimerize to form benzopinacol upon UV-A irradiation, we selected TBADT as the best photocatalyst for the targeted hydroalkylation reaction. Notably, this transformation is quite general and a diverse set of alkylphosphonates (3) could be readily isolated and characterized (see ESI, Section 7). A mechanistic study confirmed the radical nature of the process (see ESI, Section 5), where HAT is likely to occur during the rate-determining step (KIE = 1.9).

Next, the obtained alkylphosphonates were subjected to the successive HWE olefination (Scheme 2). A telescoped flow approach was developed in which the two individual steps were connected in a single streamlined flow process without intermediate purification. We selected 1,3-benzodioxole (1a), a common moiety in many medicinally-relevant molecules, as the H-donor and exposed it to the photocatalytic reaction conditions. Upon exiting the photochemical reactor, the reaction mixture containing the alkylphosphonate is merged with a stream containing paraformaldehyde (3 equiv.) and lithium *tert*-butoxide (1.1 equiv.) in tetrahydrofuran. The combined reaction mixture is subsequently introduced into a second capillary microreactor (PFA, ID: 0.75 mm; *V* = 7.1 mL; *t*_r_ = 5 min; *T* = 40 °C) and, after only 5 minutes of residence time, the targeted C(sp^3^)–H allylated product 4 could be obtained in 80% overall NMR yield (70% after isolation). Interestingly, the reaction performed decently also with 1 equivalent of 1a (65% NMR yield). Notably, the tactical combination of these two steps in flow results in a very efficient and operationally simple protocol, delivering these coveted scaffolds in only 10 minutes overall reaction time. As another benefit, the flow process could be readily scaled to produce 10 mmol of the desired compound 4 (1.52 g, 65% isolated yield, Scheme 2) without the need for tedious reoptimization of the reaction conditions, which is typically associated with batch-type scale up procedures.

**Scheme 2:**
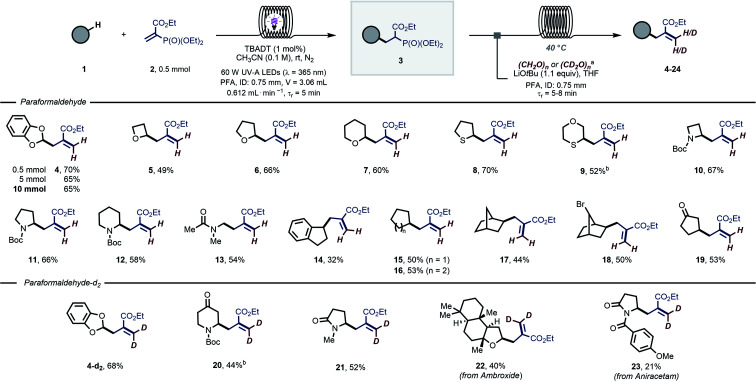
Scope of the modular allylation of strong aliphatic C–H bonds with (deuterated) paraformaldehyde. Yields are given over two steps. For further experimental details see the SI. ^a^ For (CH_2_O)_*n*_: 0.23 M aldehyde and 0.084 M LiO*t*Bu solution in tetrahydrofuran; flow rate = 0.802 mL min^−1^; *t*_R_ = 5 min. For (CD_2_O)_*n*_: 0.11 M aldehyde and 0.084 M LiO*t*Bu solution in tetrahydrofuran; flow rate = 0.802 mL min^−1^; *t*_R_ = 8 min. ^b^ TBADT was used 5 mol%.

This telescoped strategy could be subsequently applied to a wide variety of hydrogen atom donors 1 (Scheme 2). Activated substrates, such as hydrocarbon scaffolds with α-to-O C(sp^3^)–H bonds (5–7), were regioselectively allylated in yields ranging from 49–66% over two steps. Similarly, substrates containing α-to-S (8 and 9) and α-to-N (10–13) C(sp^3^)–H bonds were functionalized without difficulty (52–70% overall yield). Allylic functional groups could also be appended to activated benzylic positions (14, 32% overall yield).

Finally, even strong, non-activated aliphatic C–H bonds could be readily allylated using our approach (15–19, 44–53% overall yield).

To further demonstrate the potential of this operationally facile approach to introduce allylic functional groups, we wondered whether paraformaldehyde-d_2_ could be used in the HWE step. Such a straightforward, regioselective introduction of deuterium atoms in organic molecules would be of tremendous importance for mechanistic,^54,55^ spectroscopic and tracer studies.^56^ Using our two-step flow protocol, the analogous deutero-allylated compound 4-d_2_ was isolated in 68% yield, perfectly matching the result obtained for the non-deuterated version 4. Similarly, *N*-Boc piperidinone and *N*-methyl-2-pyrrolidone were competent substrates for this protocol affording the deuterated products 20 and 21 in 44% and 52% yield, respectively. Finally, in an effort to demonstrate the applicability of this method to the late-stage functionalization of medicinally relevant molecules, we subjected biologically active molecules to our two-step flow protocol: the terpenoid ambroxide (22, 40% yield) and the nootropic drug aniracetam (23, 21% yield) could be decorated with a deuterated allylic moiety.

In a similar vein, we turned our attention to introduce aromatic and aliphatic aldehydes in the second step, yielding trisubstituted allylic moieties, which are particularly challenging to synthesize *via* traditional photocatalyzed radical allylation approaches (Scheme 1B). By exploiting our modular protocol, a virtually limitless array of substituents can be systematically introduced (Scheme 3). In most cases, prolonged reaction times were required to obtain full conversion. In particular, electron-deficient aldehydes were convenient substrates for a fully telescoped manifold, where the flow exiting the photoreactor was directly merged with a stream containing the aldehyde and the base (see *e.g.*, 26–30, 35–40). The HWE step required 30 minutes residence time and the temperature was kept at 40 °C. We found that a range of pyridine-derived nicotinaldehydes and heteroaromatic aldehydes (35–41) were ideal substrates for this approach as well. As for electron-neutral and -rich carbonyl compounds, the HWE step required considerably longer reaction times and thus a fed-batch approach was found to be more practical (*e.g.*, 25, 31). Here, the reaction stream exiting the photoreactor was directly dosed into a stirring solution of aldehyde and base. It is important to stress that a fully telescoped approach was still possible in these cases, however higher reaction temperature (60 °C) and a back-pressure regulator (BPR, 2.8 bar) were needed to obtain full conversion within 1 hour (*e.g.*, 24, 33, 45). Another general observation that could be made is that the presence of *ortho*-substituents resulted in higher *E* : *Z* ratios (*e.g.*, 28–31, 33 and 40).

**Scheme 3:**
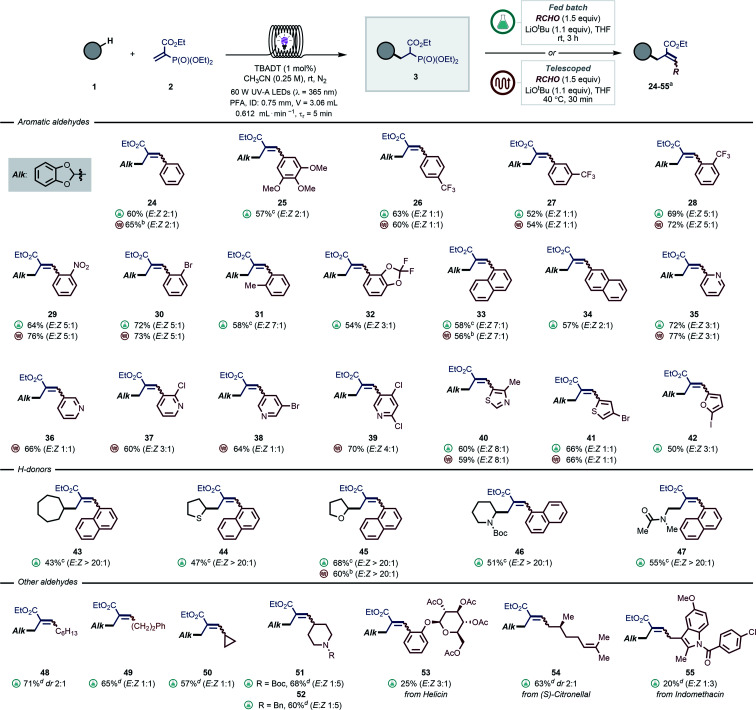
Scope of the modular allylation of strong aliphatic C–H bonds with aromatic and aliphatic aldehydes. Yields are given over two steps. For the experimental details of the fed-batch procedure see GP4 in the ESI,† while for fully telescoped approach see GP5. ^*a*^ Reactions were carried out on a 0.5 mmol scale and yields refer to isolated products, *E* : *Z* ratios were measured by ^1^H-NMR. ^*b*^ Reaction performed according to GP5, but the HWE step required 60 °C, a BPR (2.8 bar) and 1 hour residence time. ^*c*^ Reaction time: 16 h. ^*d*^ Reaction performed *via* general procedure GP6 in the ESI.†

Next, we turned to investigate different classes of hydrogen donors, such as hydrocarbons (43, 43%), (thio)ethers (44–45, 47–68%), protected amines (46, 51%) and amides (47, 55%): all proved to be competent reaction partners. In all cases, the reaction performed well, delivering densely functionalized alkenes in good yields and stereoselectivity.

It is important to note that it would be extremely challenging to access either of these motifs with the current radical allylation methodologies (Scheme 1B). Unfortunately, all attempts to engage ketones in the HWE step did not afford the desired fully-substituted olefins.

Interestingly, our protocol was also amenable to aliphatic aldehydes containing enolizable positions (48–52, 57–71% yield). The use of protected piperidine-4-carboxaldehydes allowed to obtain the corresponding allylated products 51 and 52 in excellent yields (60–68%) and with good diastereomeric ratios. In addition, medicinal agents and natural products containing carbonyls, such as acetyl-protected helicin, citronellal and indomethacin aldehyde derivatives, were also reactive delivering the targeted olefins in synthetically useful yields (53–55, 20–63%). This proves the potential of this strategy to rapidly diversify double bonds.

Next, the importance of the ester moiety as electron-withdrawing group (EWG) in the substrates to enable the targeted transformations was evaluated (Scheme 4A). Thus, we synthesized different vinyl phosphonates (2′–2′′′) and found that all of them performed well (40–68% ^1^H-NMR yield) in the photocatalytic radical hydroalkylation. We then tested our streamlined process with benzaldehyde (GP4) to study the effect of the EWG on the diastereomeric ratio in the final allylated compound. The cyano group-bearing substrate furnished the targeted compound 56 with an excellent diasteroselectivity; however, a poor mass balance was observed (22% yield despite full conversion of **3′**). In contrast, products 57 and 58 (EWG : COR) were not formed, with a complete recovery of 3′′ and 3′′′. Interestingly, we found that compound 2′′′′ could serve as a suitable radical trap as well (Scheme 4B). Using 1a as coupling partner, the targeted hydroalkylation product was obtained in excellent yield (3′′′′, 90% by ^1^H-NMR). A solvent switch and a stronger base (*n*BuLi, *n*-butyl lithium) were however required to induce the subsequent HWE step yielding styrenes 59–61 in good yields after isolation (see GP7 in the ESI†).^57,58^

**Scheme 4:**
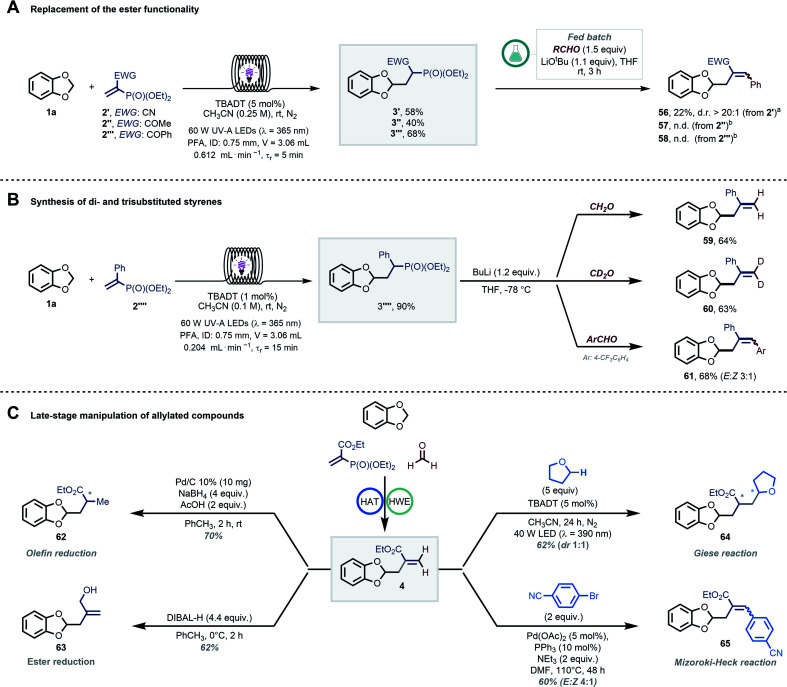
(A) Effect of the EWG on the diasteroselectivity in the final allylated product; (B) synthesis of densely functionalized styrenes by exploiting phenyl-substituted vinyl phosphonate 2′′′′; (C) examples of further diversification of compound 4, including olefin reduction, ester reduction, Giese-type radical addition and Mizoroki–Heck coupling. ^*a*^ Full conversion of 3′ was observed. ^b^ Full recovery of the alkyl phosphonates.

The regioselective and late-stage installation of allylic groups opens up innumerable possibilities for further diversification.^13^ As an illustration of this synthetic potential, we explored diverse conditions for the conversion of 4 into functionalized derivatives (Scheme 4C). The olefin and the ester functionalities could be orthogonally reduced by exploiting different reduction conditions, yielding compounds 62 (70%) and 63 (62%), respectively.^59,60^ Moreover, compound 4 was an ideal substrate for another Giese-type radical addition using decatungstate-photocatalyzed HAT (64, 62%). Finally, product 65 could be obtained *via* a classical Mizoroki–Heck-type coupling (60%).^61^

## Conclusions

In conclusion, we have developed a practical methodology which enables the modular and regioselective allylation of C(sp^3^)–H bonds. Our strategy involves a synergistic merger of a photocatalytic Hydrogen Atom Transfer and an ensuing Horner–Wadsworth–Emmons olefination in a scalable and telescoped flow protocol. In its present form, the synthetic platform offers rapid access to various di- and tri-substituted olefins from commodity chemicals containing native functionalities such as C(sp^3^)–H bonds and aldehydes. The operational simplicity of our flow protocol, requiring no intermediate purification, should facilitate a rapid transition from academic to industrial settings.

## Data availability

Electronic Supplementary Information (ESI) available: experimental details, used materials, sample preparation and analytical data (NMR). The primary NMR FID files for compounds 3e, 3f, 3n–q, 3u, 3v, 4-*d*_2_, 4–56, 59–65 are available in the FigShare repository at https://doi.org/10.21942/uva.16917640.

## Author contributions

L. C., S. B. and A. P. conceived the idea for this work and carried out the experiments. T. N. provided direction for the scientific strategy. M. N. and T. N. supervised the flow experiments. L. C. and T. N. wrote the manuscript with input from all authors.

## Conflicts of interest

There are no conflicts to declare.

## Supplementary Material

SC-013-D2SC01581A-s001
