# Supplementary material for: Modular allylation of C(sp3)–H bonds by combining decatungstate photocatalysis and HWE olefination in flow
Source: Chem Sci. 2022 May 31;13(24):7325–31. doi: 10.1039/d2sc01581a (PMC9214841; doi:10.1039/d2sc01581a)

Electronic Supplementary Material (ESI) for Chemical Science.
This journal is © The Royal Society of Chemistry 2022

Supporting Information

Modular allylation of C(sp^3)−H bonds by combining decatungstate photocatalysis and HWE olefination in flow

Luca Capaldo,[1,†] Stefano Bonciolini,[1,†] Antonio Pulcinella,[1] Manuel Nuño,[2] Timothy Noël[1,]*

[1] Flow Chemistry Group, van 't Hoff Institute for Molecular Sciences (HIMS), University of Amsterdam, Science Park 904, 1098 XH Amsterdam, The Netherlands.

[2] Vapourtec Ltd., Park Farm Business Centre, Fornham St Genevieve, Bury St Edmunds, Suffolk IP28 6TS, United Kingdom.

* *Email:* t.noel@uva.nl

Table of Contents

1. General information S4

2. Chart of Starting Materials S5

3. Synthesis of Starting Materials S6

   Synthesis of protected amines **1h-j**. S6

   Synthesis of protected helicin **S21**. S6

   Synthesis of indomethacin aldehyde derivative **S23**. S7

   Synthesis of diethyl (1-cyanovinyl)phosphonate **2'**. S7

   Synthesis of diethyl (3-oxobut-1-en-2-yl)phosphonate **2''**. S7

   Synthesis of diethyl (3-oxo-3-phenylprop-1-en-2-yl)phosphonate **2'''**. S7

   Synthesis of diethyl (1-phenylvinyl)phosphonate **2''''.** S7

4. Optimization of reaction conditions S9

   Optimization of reaction conditions: first step (radical addition) S9

   Screening of SOMOphiles in the Giese reaction S11

   Optimization of reaction conditions with (deuterated) paraformaldehyde S12

   Optimization of reaction conditions with aromatic aldehydes S13

5. Mechanistic investigation S15

   Kinetic Isotope Effect (KIE) in flow S15

   Chemical quenching in flow S16

   Mechanism proposal S17

6. General procedures S18

   General procedure for the Giese-type radical addition step (GP1) S18

   General procedure for the allylation with paraformaldehyde (GP2) S18

   General procedure for the allylation with deuterated paraformaldehyde (GP3) S18

   General procedure for the allylation with aromatic aldehydes – fed-batch (GP4) S19

   General procedure for the allylation with aromatic aldehydes – telescoped (GP5) S19

   General procedure for the allylation with aliphatic aldehydes – fed-batch (GP6) S20

   General procedure for the synthesis of compounds **59−61** (GP7) S20

7. Characterization data of synthesized compounds S22

   Characterization data of alkylphosphonates **3a**, **3e**, **3f**, **3n-q**, **3u**, **3v** S22

   Characterization data of compounds **4-19** S26

Characterization data of compounds **4-d$_2$**, **20-23** S31

Characterization data of compounds **24-47** S33

Characterization data of compounds **48-52** S44

Characterization data of compounds **53-55** S47

8. Characterization data of compounds **56**, **59-65** S49
9. Scale-up procedure (10 mmol) S53
10. Limitation of the scope S54
11. References S55
12. NMR Spectra of compounds **2'**, **2''**. S56
13. NMR Spectra of compounds **3a**, **3e**, **3f**, **3n-q**, **3u**, **3v** S60
14. NMR Spectra of compounds **4-23** S78
15. NMR Spectra of compounds **24-55** S100
16. NMR Spectra of compounds **56**, **59-65** S176

1. General information

All reagents and solvents were used as received without further purification, unless stated otherwise. Reagents and solvents were bought from Sigma Aldrich and TCI and, if applicable, kept under argon atmosphere. Diethyl vinylphosphonate was commercially available and use as received. Ethyl 2-(diethoxyphosphoryl)acrylate was synthesized as reported in the literature.[1] Technical solvents were bought from VWR International and Biosolve, and were used as received. The catalyst TBADT (tetrabutylammonium decatungstate, $(n\text{-}Bu_4N)_4W_{10}O_{32}$) was prepared according to a published procedure.[2] All capillary tubing and microfluidic fittings were purchased from IDEX Health & Science. Disposable syringes were purchased from Laboratory Glass Specialist. Syringe pumps were purchased from Chemix Inc. model Fusion 200 Touch. Product isolation was performed manually, using silica (60, F254, Merck™) or by means of a Biotage system. TLC analysis was performed using Silica on aluminum foils TLC plates (F254, Supelco Sigma-Aldrich™) with visualization under ultraviolet light (254 nm and 365 nm) or appropriate TLC staining (cerium ammonium molybdate or potassium permanganate). ^1H (400 MHz), ^{13}C (101 MHz), ^{19}F NMR (376 MHz) and ^{31}P (162 MHz) spectra were recorded unless stated otherwise at ambient temperature using a Bruker AV400 or a Bruker AV300. ^1H NMR spectra are reported in parts per million (ppm) downfield relative to $CDCl_3$ (7.26 ppm) and all ^{13}C NMR spectra are reported in ppm relative to $CDCl_3$ (77.16 ppm) unless stated otherwise. The following abbreviations have been adopted to describe the multiplicity: bs (broad singlet), s (singlet), d (doublet), t (triplet), q (quartet), p (pentet), h (hextet), hept (heptet), m (multiplet), dd (double doublet), td (triple doublet), tt (triplet of triplets). $^{31}P\{^1H\}$ NMR was used for the quantitative analysis of the outcome of the photocatalyzed step (triphenyl phosphate as external standard): a 1D sequence with inverse-gated decoupling using 30° flip angle and a d1 = 30 s was used.
Coupling constants (*J*) are reported in hertz (Hz). NMR data were processed using the MestReNova 14.1.0 software package. Known products were characterized through comparison with the corresponding ^1H NMR and ^{13}C NMR from literature. The melting points were measured using a Büchi Melting Point M-565 apparatus. High resolution mass spectra (HRMS) were collected on an AccuTOF LC, JMS-T100LP Mass spectrometer (JEOL, Japen). The names of all products were generated using the PerkinElmer ChemBioDraw Ultra v.12.0.2 software package.
For batch experiments, a 3D-printed (PLA) reactor (inner diameter: 12.5 cm) internally coated with LED strips (365 nm, 2.5 m, 300 SMD5050 LEDs, 36 W) equipped with a 3D-printed (PLA) lid with 8 holes serving as vials holder was used; in this way, up to 8 reactions could be run simultaneously. Cooling was applied via a strong compressed air flow to keep the temperature below 30 °C. For flow experiments, a Vapourtec system UV-150 equipped with a 3.06 mL PFA coil (ID = 0.75 mm) and 60 W 365 nm LED was used for photochemical reactions in flow.

2. Chart of Starting Materials

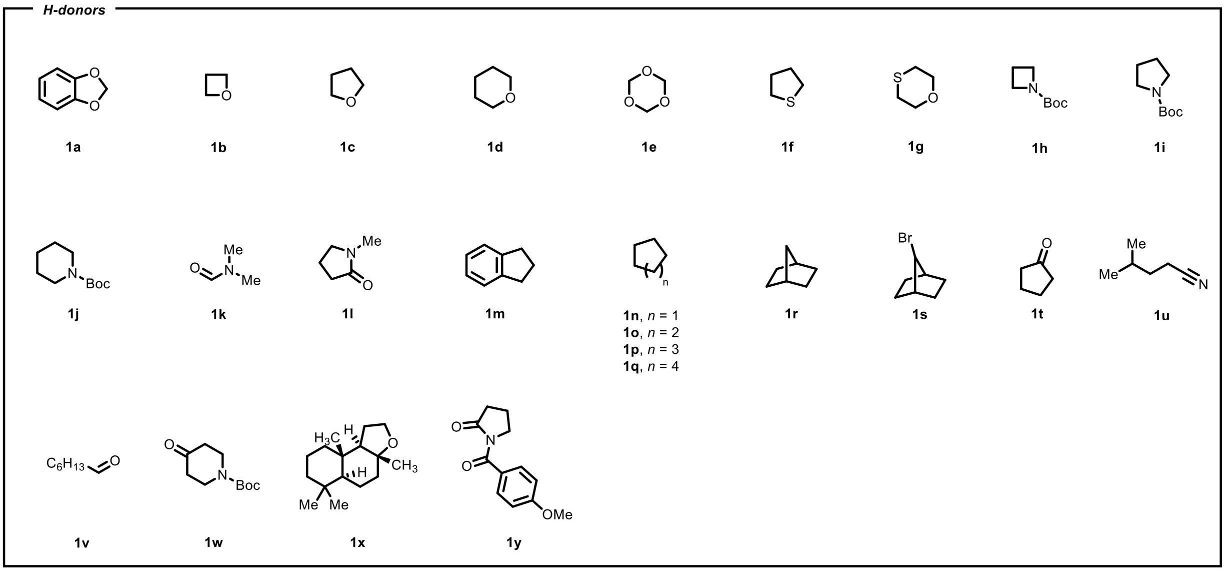

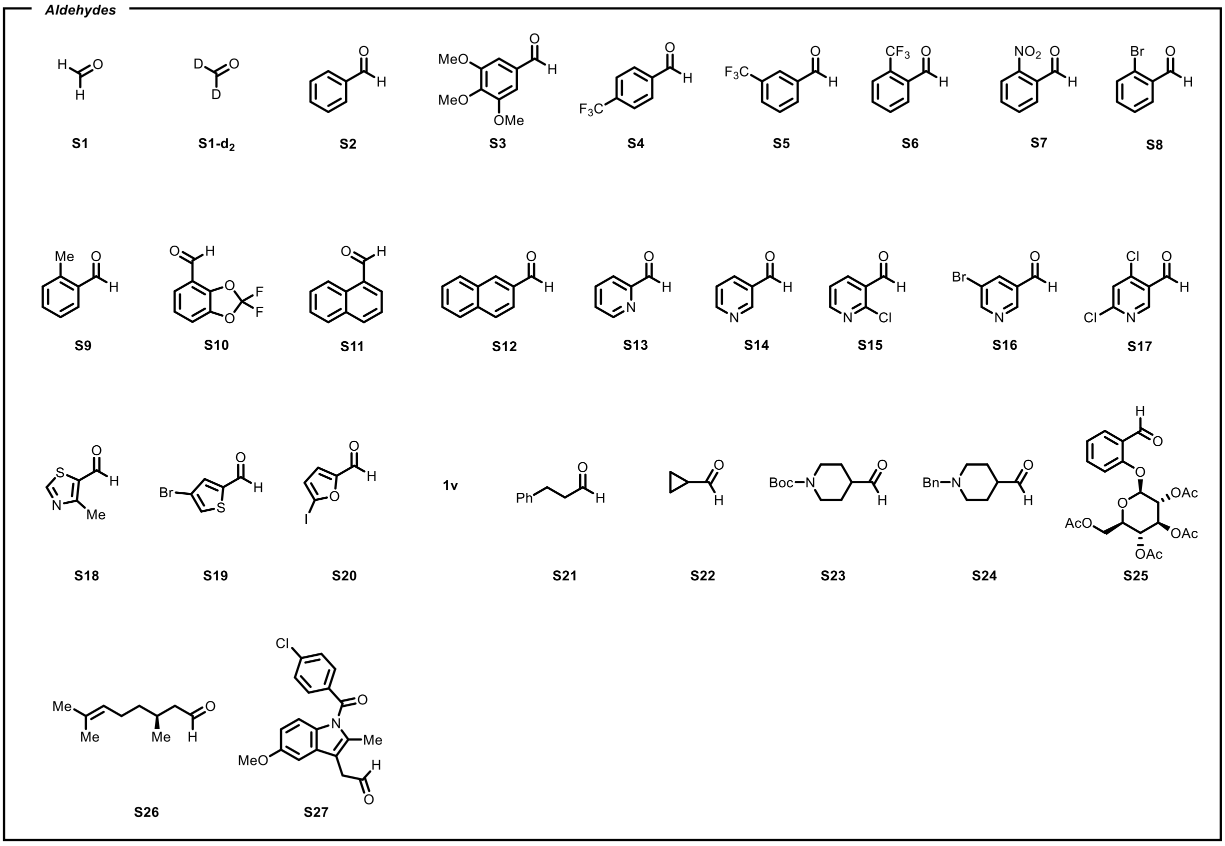

3. Synthesis of Starting Materials

*Synthesis of protected amines **1h-j**.*

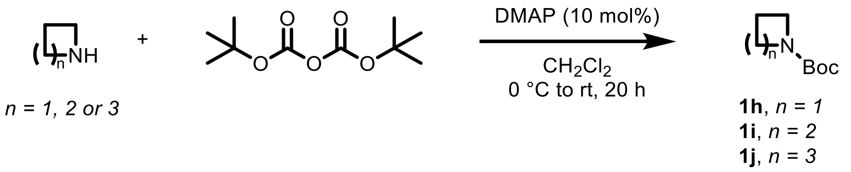

Compounds **1h-j** were synthesized adapting a procedure reported in the literature.[3]

In particular, Boc_2O (2.4 g, 11 mmol, 1.1 equiv) was dissolved in CH_2Cl_2 in an oven-dried vial under inert atmosphere. In the meantime, in a 100 mL round-bottom flask, 4-DMAP (122 mg, 1 mmol, 10 mol%) together with azetidine (674 µL, 10 mmol, ρ = 0.847 g mL^{-1}), pyrrolidine (821 µL, 10 mmol, ρ = 0.866 g mL^{-1}) or piperidine (988 µL, 10 mmol, ρ = 0.862 g mL^{-1}) were dissolved in 30 mL of dry CH_2Cl_2. The solution was placed in an ice bath and the Boc_2O solution was added dropwise via a syringe. The resulting solution was stirred at 0 °C for 10 mins and then at room temperature for 20 hours. Reaction was monitored via ^1H NMR, quenched with water once completed and the organic phase was washed with water (3x25 mL) and once with brine (25 mL). The resulting organic phase was then dried over Na_2SO_4 and the solvent was removed under reduced pressure. The crude was purified via column chromatography (SiO_2, Hexane: Ethyl Acetate 90:10). Yields: **1h**, colorless liquid, 64%; **1i**, colorless liquid, 72%; **1j**, colorless liquid, 69%;

Spectroscopic data for compounds **1h**, **1i**, **1j** are in accordance with those reported in the literature.

1h: ^1H NMR (400 MHz, $CDCl_3$): δ 3.95 (t, 4H), 2.18 (p, 2H), 1.45 (s, 9H).[4]

1i: ^1H NMR (400 MHz, $CDCl_3$) δ 3.30-3.28 (m, 4H), 1.86-1.74 (m, 4H), 1.45 (s, 9H).[3]

1j: ^1H NMR (400 MHz, $CDCl_3$): δ 3.37 (t, 4H), 1.63-1.49 (m, 6H), 1.47 (s, 9H).[3]

*Synthesis of protected helicin **S21**.*

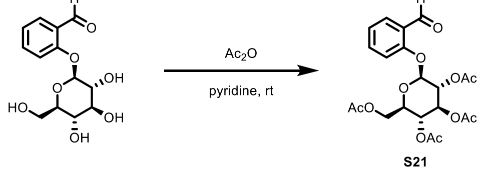

Helicin was protected using a procedure reported in the literature.[5] Spectroscopic data are in accordance with those reported in the literature. ^1H NMR (300 MHz, $CDCl_3$) δ 10.34 (s, 1H), 7.86 (dd, J_1 = 8, J_2 = 2 Hz, 1H), 7.56 (m, 1H), 7.18 (t, J = 8 Hz, 1H), 7.11 (d, J = 8 Hz, 1H), 5.43 – 5.27 (m, 2H), 5.27 – 5.13 (m, 2H), 4.30 (dd, J_1 = 12, J_2 = 5 Hz, 1H), 4.17 (dd, J_1 = 12, J_2 = 2 Hz, 1H), 3.90 (ddd, J_1 = 10, J_2 = 5 Hz, J_3 = 2 Hz, 1H), 2.09 (s, 3H), 2.08 (s, 3H), 2.06 (s, 3H), 2.02 (s, 3H).[5]

Synthesis of indomethacin aldehyde derivative ***S23***.

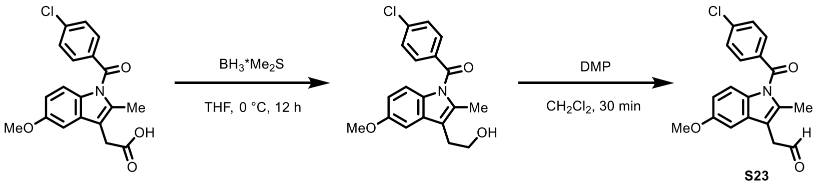

S23 was synthesized using a procedure reported in the literature.[6] Spectroscopic data are in accordance with those reported in the literature. ^{1}H NMR (400 MHz, $CDCl_3$) δ 9.71 (t, J = 2 Hz, 1H), 7.73 – 7.61 (m, 2H), 7.53 – 7.41 (m, 2H), 6.91 – 6.81 (m, 2H), 6.69 (dd, J_1 = 9, J_2 = 3Hz, 1H), 3.83 (s, 3H), 3.73 (d, J = 2 Hz, 2H), 2.39 (s, 3H).[6]

Synthesis of diethyl (1-cyanovinyl)phosphonate ***2'***.

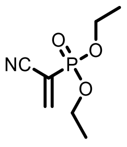

2'

Synthesized according to a procedure present in literature.[7]
^{1}H NMR (400 MHz, $CDCl_3$) δ 6.96 – 6.69 (m, 2H), 4.24 – 4.13 (m, 4H), 1.37 (t, J = 7 Hz, 6H).
^{13}C NMR (101 MHz, $CDCl_3$) δ 148.2, 148.1, 116.6, 114.8, 114.7, 114.7, 64.0, 63.9, 16.3, 16.3.
^{31}P NMR (121 MHz, $CDCl_3$) δ 7.2.
HRMS (FI+) (m/z): [M+H]+ calcd. for $C_7H_{12}NO_3P$, 190.0633; found, 190.0628.

Synthesis of diethyl (3-oxobut-1-en-2-yl)phosphonate ***2''***.

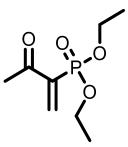

2''

Synthesized according to a procedure present in literature.[7]
^{1}H NMR (400 MHz, $CDCl_3$) δ 6.83 (dd, J_1 = 17 Hz, J_2 = 1 Hz, 1H), 6.72 (dd, J_1 = 6 Hz, J_2 = 1 Hz, 1H), 4.26 – 4.11 (m, 4H), 2.44 (s, 3H), 1.36 (t, J = 7 Hz, 6H).
^{13}C NMR (101 MHz, $CDCl_3$) δ 196.4, 196.3, 141.9, 141.0, 141.0, 140.1, 62.8, 62.7, 27.9, 27.9, 16.5, 16.4.
^{31}P NMR (121 MHz, $CDCl_3$) δ 13.4.

Synthesis of diethyl (3-oxo-3-phenylprop-1-en-2-yl)phosphonate ***2'''***.

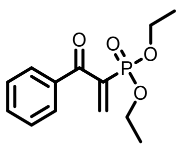

2'''

Synthesized according to a procedure present in literature. Spectroscopic data in accordance with literature.[7]

Synthesis of diethyl (1-phenylvinyl)phosphonate ***2''''***.

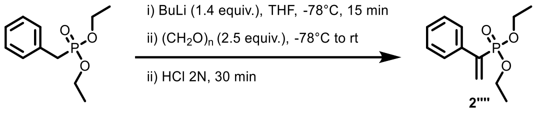

A solution of n-BuLi (2.3 M, 3.4 mL, 1.4 equiv.) in hexane was added dropwise into an oven-dried two-neck round-bottom flask containing a solution of diethyl benzylphosphonate (1.27 g, 5.6 mmol) in THF (12.0 mL) at -

78˚C under N_2. The solution was stirred at this temperature for 15 min until a bright yellow color appeared. Paraformaldehyde (0.42 g, 14 mmol) was then added and the cooling bath was removed. The reaction was continued at room temperature with stirring overnight. Then the solution was acidified by HCl 2 M, stirred for 30 minutes and extracted with CH_2Cl_2 (3x50 mL). The resulting organic phase was then dried over Na_2SO_4 and the solvent was removed under reduced pressure. The crude was purified via flash column chromatography on silica gel (Hexane:Ethyl Acetate 70:30) to get 0.503 g of **2''''** as a colorless oil (38% yield). Spectroscopic data are in accordance with those reported in the literature.[8] ^{1}H NMR (300 MHz, $CDCl_3$) δ 7.58 – 7.47 (m, 2H), 7.41 – 7.29 (m, 3H), 6.34 (dd, J_1 = 22, J_2 = 2 Hz, 1H), 6.16 (dd, J_1 = 46 Hz, J_2 = 2 Hz, 1H) 4.23 – 3.98 (m, 4H), 1.28 (t, *J* = 7 Hz, 6H). ^{13}C NMR (76 MHz, $CDCl_3$) (101 MHz, $CDCl_3$) δ 139.8 (d, *J* = 175 Hz), 136.8 (d, *J* = 12 Hz), 131.8 (d, *J* = 8 Hz), 128.5, 128.4, 127.6 (d, *J* = 6 Hz), 62.3 (d, *J* = 6 Hz), 16.4 (d, *J* = 6 Hz). ^{31}P NMR (121 MHz, $CDCl_3$) δ 17.1.

4. Optimization of reaction conditions

Optimization of reaction conditions: first step (radical addition)

The preliminary optimization of the first step of the telescoped approach, namely the Giese-type radical addition, was performed in batch conditions (Table S1, entries 1-8) by studying the radical addition of cyclohexane (**1o**) onto ethyl 2-(diethoxyphosphoryl)acrylate in CH_3CN (1 mL). Afterwards, we adapted our chemistry to flow conditions (Table S1, entries 9-17). As a quantification method a proton-decoupled, inverse-gated quantitative ^{31}P-NMR was used since the starting material and the product show very defined peaks at 12.2 ppm and 23.5 ppm, respectively. Triphenyl phosphate was selected as the external standard (−16.2 ppm).

<u>*Reaction conditions for the optimization in batch:*</u> In a 7 mL borosilicate glass vial equipped with a screw cap, ethyl 2-(diethoxyphosphoryl)acrylate (**2**, 0.025-0.2 M), **1o** (3-20 equiv) and TBADT (1 mol%) were dissolved in CH_3CN (1 mL). The resulting solution was Ar-bubbled for 1 min and irradiated with UV-A LEDs (λ = 365 nm, 36 W) for 20 hours. After irradiation, the vial was opened and triphenyl phosphate was added as an external standard. The mixture was sonicated for 2 minutes to ensure complete solubilization of the standard, finally an aliquot was withdrawn to perform quantitative ^{31}P-NMR to evaluate consumption and yield.

<u>*Reaction conditions for the optimization in flow:*</u> In a 7 mL borosilicate glass vial, ethyl 2-(diethoxyphosphoryl)acrylate (**2**, 0.1 M), **1o** (20 equiv) and TBADT (1 mol%) were dissolved in CH_3CN (1 mL) and the vial was sealed with a septum. The resulting solution was Ar-bubbled for 1 min and taken up with a syringe. Finally, the syringe was mounted on a syringe pump and pushed into a Vapourtec UV-150 equipped with UV-A or blue LEDs (λ = 365 or 456 nm, 60 W) for the required residence time. The outflow was collected in a 10 mL round-bottom flask and triphenyl phosphate was added as an external standard. The mixture was sonicated for 2 minutes to ensure complete solubilization of the standard, finally an aliquot was withdrawn to perform quantitative ^{31}P-NMR to evaluate consumption and yield.

Table S1. Optimization of reaction conditions in batch and in flow for the Giese-type radical addition.

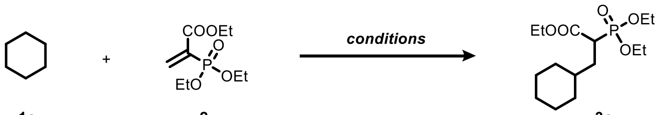

1o + **2** → **3o**

	Entry	**1o** (eq.)	[**2**]	Reaction conditions	**2a** consumption	Yield[a]
batch	1	5	0.025 M	TBADT (1 mol%)<br>MeCN (1 mL), rt, N_2<br>36 W LED (λ = 365 nm)<br>20 h	quant.	19%
	2	"	0.05 M	"	quant.	28%
	3	"	0.075 M	"	quant.	34%
	4	"	0.1 M	"	quant.	40%
	5	"	0.2 M	"	quant.	40%
	6	3	0.1 M	"	quant.	31%
	7	10	"	"	quant.	52%
	8	20[b]	"	"	quant.	62%
	9	10	"	TBADT (1 mol%)<br>MeCN (1 mL), rt, N_2<br>no light, 20 h	11%	n.d.
	10	10	"	no TBADT<br>MeCN (1 mL), rt, N_2<br>36 W LED (λ = 365 nm)<br>20 h	<5%	n.d.
flow	11	"	"	TBADT (1 mol%)<br>MeCN (1 mL), rt, N_2<br>60 W LED (λ = 365 nm)<br>τ_r: 5 min	quant.	65%<br>(64%)
	12	"	"	TBADT (1 mol%)<br>MeCN (1 mL), rt, N_2<br>60 W LED (λ = 365 nm)<br>τ_r: 3 min	87%	46%
	13	"	"	TBADT (1 mol%)<br>MeCN (1 mL), rt, N_2<br>60 W LED (λ = 365 nm)<br>τ_r: 1 min	43%	25%
	14	"	"	BP (1 mol%)<br>MeCN (1 mL), rt, N_2<br>60 W LED (λ = 365 nm)<br>τ_r: 5 min	38%	7%
	15	"	"	BP (10 mol%)<br>MeCN (1 mL), rt, N_2<br>60 W LED (λ = 365 nm)<br>τ_r: 5 min	88%	45%
	16	"	"	BP (20 mol%)<br>MeCN (1 mL), rt, N_2<br>60 W LED (λ = 365 nm)<br>τ_r: 5 min	quant.	68%
	17	"	"	EY (10 mol%)<br>MeCN (1 mL), rt, N_2<br>60 W LED (λ = 450 nm)<br>τ_r: 5 min	12%	n.d.
	18	"	"	FL (10 mol%)<br>MeCN (1 mL), rt, N_2<br>60 W LED (λ = 365 nm)<br>τ_r: 5 min	21%	n.d.
	19[c]	"	"	AQ or PT (10 mol%)<br>MeCN (1 mL), rt, N_2	n.a.	n.a.

[a] Yields determined by ^{31}P NMR spectroscopy, triphenyl phosphate as the external standard. [b] the solution was *gently* heated up prior to irradiation to promote complete solubilization of **1o**. [c] the reaction mixture was not homogeneous even upon prolonged sonication and heating, and could not be used under flow conditions. n.d.: not detected; n.a.: not available. BP: benzophenone; EY: Eosin Y; FL: fluorenone; AQ: anthraquinone; PT: 5,7,12,14-pentacenetetrone

Screening of SOMOphiles in the Giese reaction

In a typical experiment, (substituted) vinylphosphonate (0.2 mmol), **1a** (115 µL, 1.0 mmol, 5 equiv) and TBADT (34 mg, 5 mol%) were dissolved in CH_3CN (0.68 mL) in an oven-dried 7 mL vial. The vial was sealed with a rubber septum and the solution was sparged with N_2 (1 min). The mixture was taken up with a 2 mL syringe and mounted on a syringe pump (Feed A) connected to a Vapourtec system UV-150 equipped with a 3.06 mL PFA coil (ID = 0.75 mm) and 60 W 365 nm LEDs.

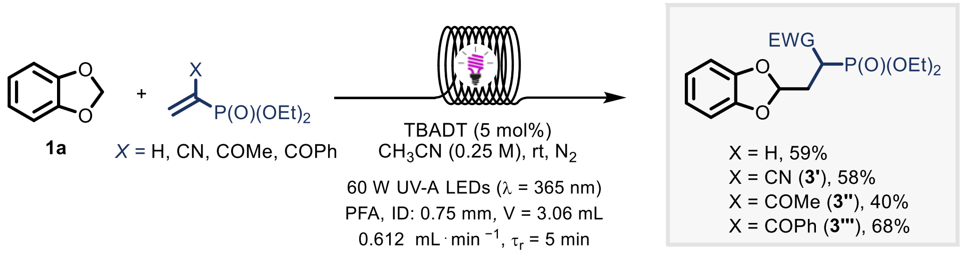

Scheme 1. Screening of different SOMOphiles.

Optimization of reaction conditions with (deuterated) paraformaldehyde

In a typical experiment, ethyl 2-(diethoxyphosphoryl)acrylate (**2**, 118 mg, 0.5 mmol), **1a** (288 µL, 2.5 mmol, 5 equiv) and TBADT (17 mg, 1 mol%) were dissolved in CH_3CN (4.7 mL) in an oven-dried 7 mL vial. The vial was sealed with a rubber septum and the solution was sparged with N_2 (5 min). The mixture was taken up with a 5 mL syringe and mounted on a syringe pump (Feed A) connected to a Vapourtec system UV-150 equipped with a 3.06 mL PFA coil (ID = 0.75 mm) and 60 W 365 nm LEDs.

Parallelly, in an oven-dried vial a stock solution of **S1** or **S1-d₂** in dry THF was prepared and LiOtBu 1 M in dry THF was added to obtain final concentrations as shown in Table S2. Upon addition of LiOtBu and following sonication (10 min) the suspension turned to a flowable solution. This stock solution was taken up with a 10 mL syringe and mounted on a syringe pump (Feed B).

Feed A was pumped at 0.612 mL min^{-1} through the Vapourtec system (V = 3.06 mL, τ_R=5 min). The blue outflow of the latter (due to the reduced form of the photocatalyst, TBADT) was then mixed with Feed B (pumped at 0.802 mL min^{-1}) through a PEEK T-mixer. When the outflow of the photoreactor turned back to colorless (marking the end of the photoreaction), neat acetonitrile was loaded on both syringe pumps to push the combined feeds into a PFA coil (ID = 0.75 mm) kept in an ultrasonic bath at 40 °C. Finally, the resulting reaction crude was directly collected into a sat'd NH_4Cl solution for quenching. The mixture was extracted three times with Ethyl Acetate and the organic phases were collected and dried over Na_2SO_4. After filtration and rotary evaporation, the crude was analyzed via ^1H NMR to determine the reaction yield (CH_2Br_2 or trichloroethylene as the external standard).

Table S2. Optimization of the reaction with (deuterated) paraformaldehyde.

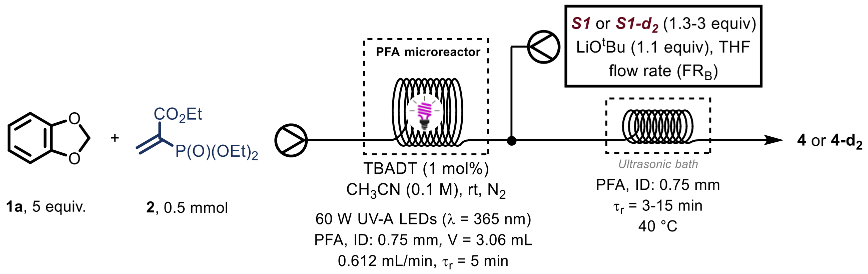

Entry	[base]	[**S1**]	FR_B	τ_r	^1H-NMR yield[a]	RSM
1	0.19 M (1.1 equiv.)	0.23 M (1.3 equiv.)	0.348 mL/min	10 min	52%	30%
2	0.19 M (1.1 equiv.)	0.23 M (1.3 equiv.)	0.348 mL/min	12 min	60%	20%
3	0.19 M (1.1 equiv.)	0.23 M (1.3 equiv.)	0.348 mL/min	15 min	78%	< 5%
4	0.084 M (1.1 equiv.)	0.23 M (3.0 equiv.)	0.802 mL/min	10 min	80%	n.d.
**5**	**0.084 M (1.1 equiv.)**	**0.23 M (3.0 equiv.)**	**0.802 mL/min**	**5 min**	**80%**	**n.d.**
6	0.084 M (1.1 equiv.)	0.23 M (3.0 equiv.)	0.802 mL/min	3 min	75%	traces

Entry	[base]	[**S1-d₂**]	FR_B	τ_r	^1H-NMR yield[a]	RSM
1	0.084 M (1.1 equiv.)	0.23 M (3.0 equiv.)	0.802 mL/min	5 min	70%	n.d.
2	0.084 M (1.1 equiv.)	0.11 M (1.5 equiv.)	0.802 mL/min	5 min	65%	5%
3	0.084 M (1.1 equiv.)	0.11 M (1.5 equiv.)	0.802 mL/min	8 min	72%	n.d.

[a] Yields determined by ^1H NMR spectroscopy, CH_2Br_2 or trichloroethylene as the external standard.

Optimization of reaction conditions with aromatic aldehydes

In a typical experiment, ethyl 2-(diethoxyphosphoryl)acrylate (**2**, 118 mg, 0.5 mmol), **1a** (288 μL, 2.5 mmol, 5 equiv) and TBADT (17 mg, 1 mol%) were dissolved in CH_3CN (1.7 mL) in an oven-dried 7 mL vial. The vial was sealed with a rubber septum and the solution was sparged with N_2 (2 min). The mixture was taken up with a 5 mL syringe and mounted on a syringe pump (Feed A) connected to a Vapourtec system UV-150 equipped with a 3.06 mL PFA coil (ID = 0.75 mm) and 60 W 365 nm LEDs. Then Method A or Method B was applied.

Method A (Telescoped)

Parallelly, in an oven-dried vial a stock solution of benzaldehyde **S2** in dry THF was prepared under N_2 atmosphere, to which LiOtBu 1 M in dry THF was added. This stock solution was taken up with a 10 mL syringe and mounted on a syringe pump.

Feed A was pumped at 0.612 mL min^{-1} through the Vapourtec system (V = 3.06 mL, τ_R=5 min). The blue outflow of the latter (due to the reduced form of the photocatalyst, TBADT) was then mixed with Feed B (pumped at 0.420 mL min^{-1}) through a PEEK T-mixer. When the outflow of the photoreactor turned back to colorless (marking the end of the photoreaction), neat acetonitrile was loaded on both syringe pumps to push the combined feeds into a PFA coil (ID = 0.75 mm) at 1.032 mL min^{-1} for the required residence time. Finally, the resulting reaction crude was directly collected into a sat'd NH_4Cl solution for quenching. The mixture was extracted three times with Ethyl Acetate and the organic phases were collected and dried over Na_2SO_4. After filtration and rotary evaporation, the crude was analyzed via ^1H NMR to determine the reaction yield (CH_2Br_2 as the external standard) and diastereomeric ratio.

Method B (Fed-batch)

Parallelly, in an oven-dried vial benzaldehyde **S2** was dissolved in dry THF under N_2 atmosphere. The solution was kept under inert atmosphere with a balloon filled with N_2.

Feed A was pumped at 0.612 mL min^{-1} through the Vapourtec system (V = 3.06 mL, τ_R=5 min). Before the first drop of the outflow of the photoreactor entered in contact with the aldehyde solution, LiOtBu 1 M in dry THF (0.550 mL, 0.55 mmol, 1.1 equiv) was added in one portion to the latter to obtain a final concentration of benzaldehyde of 0.54 M. The blue outflow of the latter was directly added to aldehyde solution via a needle. After all Feed A was added, the solution was kept stirring at room temperature for the indicated time. Eventually, the solution was quenched with sat'd NH_4Cl solution. The mixture was extracted three times with Ethyl Acetate and the organic phases were collected and dried over Na_2SO_4. After filtration and rotary evaporation, the crude was analyzed via ^1H NMR to determine the reaction yield (CH_2Br_2 as the external standard) and diastereomeric ratio.

Table S3. Optimization of the reaction with aromatic aldehydes.

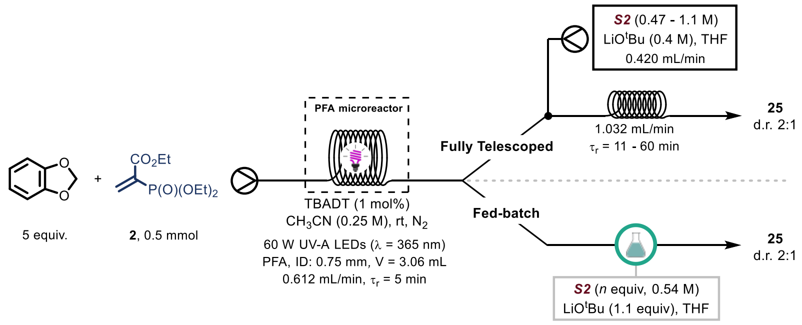

Fully Telescoped

Entry	[**S2**]	τ_r	^{1}H-NMR yield[a]	RSM
1	0.47 M (1.3 equiv)	11 min	24%	49%
2[b]	0.47 M (1.3 equiv)	11 min	27%	48%
3[c]	0.47 M (1.3 equiv)	11 min	40%	29%
4[c]	0.47 M (1.3 equiv)	51 min	67%	10%
5[d]	0.47 M (1.3 equiv)	11 min	61%	10%
**6[d]**	**0.55 M (1.5 equiv)**	**60 min**	**68%**	**traces**
7	1.08 M (3.0 equiv)	11 min	41%	45%
8[d]	1.08 M (3.0 equiv)	11 min	60%	11%

Fed-batch

Entry	**S2** (equiv)	time (h)	^{1}H-NMR yield[a]	RSM
1	1.3 equiv. (0.54 M)	1	49%	22%
2	1.3 equiv. (0.54 M)	2	63%	8%
3	1.3 equiv. (0.54 M)	3	62%	7%
**4**	**1.5 equiv. (0.54 M)**	**3**	**65% (60%)**	**traces**

[a] Yields determined by ^{1}H NMR spectroscopy, CH_2Br_2 as the external standard. [b] the coil for the second step (HWE olefination) was heated at 30 °C by means of a water bath. [c] the coil for the second step (HWE olefination) was heated at 50 °C by means of a water bath. [d] the coil for the second step (HWE olefination) was heated at 60 °C by means of a water bath; a back-pressure regulator (BPR, 2.8 bar) was used.

5. Mechanistic investigation

Kinetic Isotope Effect (KIE) in flow

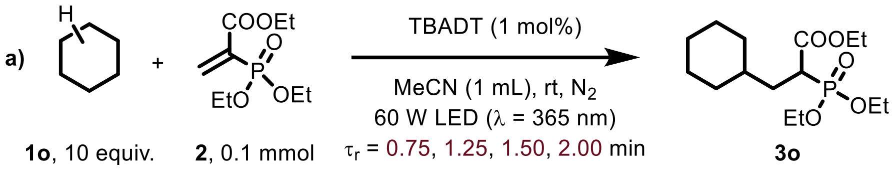

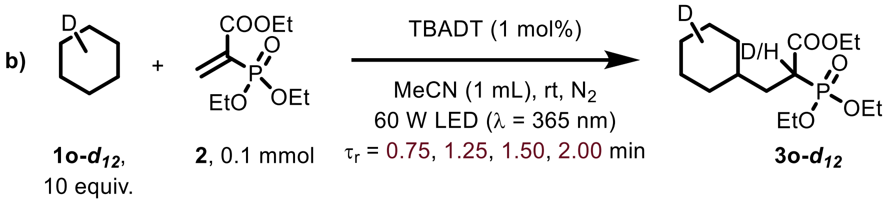

In a 7 mL borosilicate glass vial, ethyl 2-(diethoxyphosphoryl)acrylate (**2**, 0.1 M), **1o** or **1o-*d12*** (10 equiv) and TBADT (1 mol%) were dissolved in CH_3CN (1 mL) and the vial was sealed with a septum. The resulting solution was N_2-bubbled for 1 min and taken up with a syringe. Finally, the syringe was mounted on a syringe pump and pushed into a Vapourtec UV-150 equipped with UV-A or blue LEDs (λ = 365 nm, 60 W) for the required residence time (τ_r = 0.75, 1.25, 1.50, 2.00 min). The outflow was collected in a 10 mL round-bottom flask and triphenyl phosphate was added as an external standard. The mixture was sonicated for 2 minutes to ensure complete solubilization of the standard, finally an aliquot was withdrawn to perform quantitative ^{31}P-NMR to evaluate the yield.

Residence time (min)	Yield (%) for reaction a)	Yield (%) for reaction b)
0	0	0
0.75	24	12
1.25	36	20
1.50	45	22
2.00	52	27

Yield for reactions a) and b) was plotted vs residence time (Figure S1) to give a linear correlation and the KIE was calculated as the ratio between the slopes of the two curves to be **1.9**.

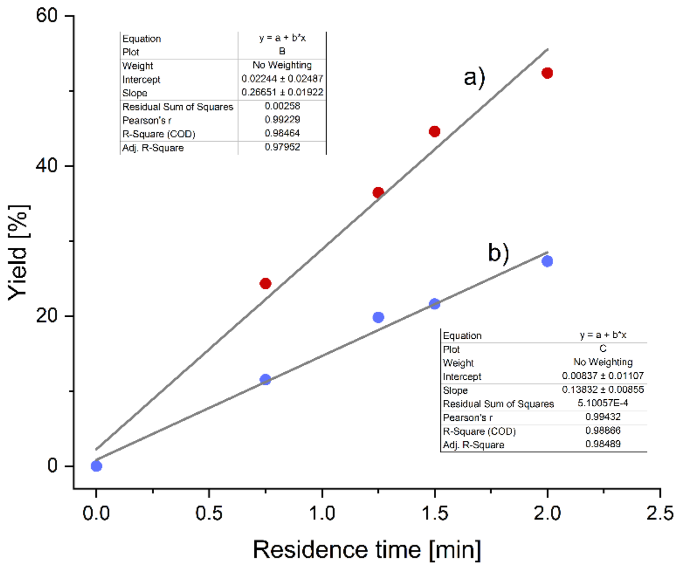

Figure S1. Yield vs residence time for the evaluation of the KIE.

Chemical quenching in flow

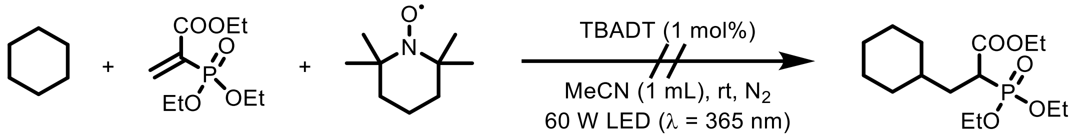

1o, 10 equiv. **2**, 0.1 mmol 5 equiv. **3o**

In a 7 mL borosilicate glass vial, ethyl 2-(diethoxyphosphoryl)acrylate (**2**, 0.1 M), TEMPO (5 equiv) and TBADT (1 mol%) were dissolved in CH_3CN (1 mL) and the vial was sealed with a septum. The resulting solution was N_2-bubbled for 1 min, **1o** was added and the reaction mixture was taken up with a syringe. Finally, the syringe was mounted on a syringe pump and pushed into a Vapourtec UV-150 equipped with UV-A or blue LEDs (λ = 365 nm, 60 W) for the required residence time (τ_r = 5 min). The outflow was collected in a 10 mL round-bottom flask and triphenyl phosphate was added as an external standard. The mixture was sonicated for 2 minutes to ensure complete solubilization of the standard, finally an aliquot was withdrawn to perform quantitative ^{31}P-NMR to evaluate the yield. Product **3o** was not detected.

Mechanism proposal

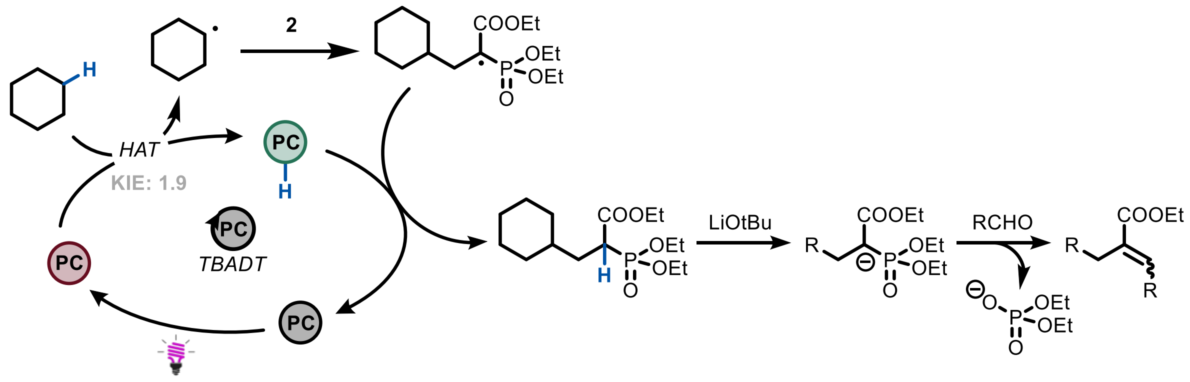

Scheme 2. Mechanism proposal for the modular allylation of C(sp^3)−H bonds via the combination of decatungstate photocatalysis and HWE olefination.

6. General procedures

General procedure for the Giese-type radical addition step (GP1)

In a typical experiment, ethyl 2-(diethoxyphosphoryl)acrylate (**2**, 118 mg, 0.5 mmol), **1a** (288 µL, 2.5 mmol, 5 equiv) and TBADT (17 mg, 1 mol%) were dissolved in CH_3CN (4.7 mL) in an oven-dried 7 mL vial. The vial was sealed with a rubber septum and the solution was sparged with N_2 (5 min). In the case of volatile compounds, these were added after degassing via syringe through the septum. The mixture was taken up with a 5 mL syringe and mounted on a syringe pump (Feed A) connected to a Vapourtec system UV-150 equipped with a PFA coil (ID = 0.75 mm) and 60 W 365 nm LEDs.

Feed A was pumped at 0.612 mL min^{-1} through the Vapourtec system (V = 3.06 mL, τ_R=5 min). The blue outflow of the latter (due to the reduced form of the photocatalyst, TBADT) was collected in a round-bottom flask. Finally, the solvent was removed and the reaction crude was purified via column chromatography.

This procedure was used for compounds **3a**, **3e**, **3f**, **3n-q**, **3u**, **3v**.

General procedure for the allylation with paraformaldehyde (GP2)

In a typical experiment, ethyl 2-(diethoxyphosphoryl)acrylate (**2**, 118 mg, 0.5 mmol), **1a** (288 µL, 2.5 mmol, 5 equiv) and TBADT (17 mg, 1 mol%) were dissolved in CH_3CN (5 mL) in an oven-dried 7 mL vial. The vial was sealed with a rubber septum and the solution was sparged with N_2 (5 min). In the case of volatile compounds, these were added after degassing via syringe through the septum. The mixture was taken up with a 5 mL syringe and mounted on a syringe pump (Feed A) connected to a Vapourtec system UV-150 equipped with a 3.06 mL PFA coil (ID = 0.75 mm) and 60 W 365 nm LEDs.

Parallelly, in an oven-dried vial a stock solution 0.23 M in paraformaldehyde (**S1**) and 0.084 M in LiOtBu (a 1 M in dry THF solution was used) was prepared. Upon addition of LiOtBu and following sonication (10 min) the suspension turned to a flowable solution. This stock solution was taken up with a 10 mL syringe and mounted on a syringe pump (Feed B).

Feed A was pumped at 0.612 mL min^{-1} through the Vapourtec system (V = 3.06 mL, τ_R=5 min). The blue outflow of the latter (due to the reduced form of the photocatalyst, TBADT) was then mixed with Feed B (pumped at 0.802 mL min^{-1}) through a PEEK T-mixer. When the outflow of the photoreactor turned back to colorless (marking the end of the photoreaction), neat acetonitrile was loaded on both syringe pumps to push the combined feeds into a 7.10 mL PFA coil (ID = 0.75 mm) at 1.414 mL min^{-1} (τ_R = 5 min). This second coil was kept in an ultrasonic bath at 40 °C. Finally, the resulting reaction crude was directly collected into a sat'd NH_4Cl solution for quenching. The mixture was extracted three times with Ethyl Acetate and the organic phases were collected and dried over Na_2SO_4. After filtration and rotary evaporation, the crude was analyzed via ^1H NMR to determine the reaction yield (CH_2Br_2 as the external standard) and then purified via column chromatography.

This procedure was used for compounds **4-19**.

General procedure for the allylation with deuterated paraformaldehyde (GP3)

In a typical experiment, ethyl 2-(diethoxyphosphoryl)acrylate (**2**, 118 mg, 0.5 mmol), **1a** (288 µL, 2.5 mmol, 5 equiv) and TBADT (17 mg, 1 mol%) were dissolved in CH_3CN (5 mL) in an oven-dried 7 mL vial. The vial was sealed with a rubber septum and the solution was sparged with N_2 (5 min). In the case of volatile

compounds, these were added after degassing via syringe through the septum. The mixture was taken up with a 5 mL syringe and mounted on a syringe pump (Feed A) connected to a Vapourtec system UV-150 equipped with a 3.06 mL PFA coil (ID = 0.75 mm) and 60 W 365 nm LEDs.
Parallelly, in an oven-dried vial a stock solution 0.11 M in deuterated paraformaldehyde (**S1-d$_2$**) and 0.084 M in LiOtBu (a 1 M in dry THF solution was used) was prepared. Upon addition of LiOtBu and following sonication (20 min) the suspension turned to a flowable solution. This stock solution was taken up with a 10 mL syringe and mounted on a syringe pump (Feed B).
Feed A was pumped at 0.612 mL min^{-1} through the Vapourtec system (V = 3.06 mL, τ_R=5 min). The blue outflow of the latter (due to the reduced form of the photocatalyst, TBADT) was then mixed with Feed B (pumped at 0.802 mL min^{-1}) through a PEEK T-mixer. When the outflow of the photoreactor turned back to colorless (marking the end of the photoreaction), neat acetonitrile was loaded on both syringe pumps to push the combined feeds into a 11.3 mL PFA coil (ID = 0.75 mm) at 1.414 mL min^{-1} (τ_R = 8 min). This second coil was kept in an ultrasonic bath at 40 °C. Finally, the resulting reaction crude was directly collected into sat'd NH_4Cl solution for quenching. The mixture was extracted three times with Ethyl Acetate and the organic phases were collected and dried over Na_2SO_4. After filtration and rotary evaporation, the crude was analyzed via ^{1}H NMR to determine the reaction yield (CH_2Br_2 as the external standard) and then purified via column chromatography.
This procedure was used for compounds **4-d$_2$**, **20-23**.

General procedure for the allylation with aromatic aldehydes – fed-batch (GP4)

In a typical experiment, ethyl 2-(diethoxyphosphoryl)acrylate (**2**, 118 mg, 0.5 mmol), **1a** (288 µL, 2.5 mmol, 5 equiv) and TBADT (17 mg, 1 mol%) were dissolved in CH_3CN (1.7 mL) in an oven-dried 7 mL vial. The vial was sealed with a rubber septum and the solution was sparged with N_2 (2 min). In the case of volatile compounds, these were added after degassing via syringe through the septum. The mixture was taken up with a 5 mL syringe and mounted on a syringe pump (Feed A) connected to a Vapourtec system UV-150 equipped with a 3.06 mL PFA coil (ID = 0.75 mm) and 60 W 365 nm LEDs.
Parallelly, in an oven-dried vial benzaldehyde (**S2**, 76 µL, 0.75 mmol, 1.5 equiv, ρ = 1.04 g mL^{-1}) was dissolved in dry THF (830 µL) under N_2 atmosphere. The solution was kept under inert atmosphere with a balloon filled with N_2 (Batch).
Feed A was pumped at 0.612 mL min^{-1} through the Vapourtec system (V = 3.06 mL, τ_R=5 min). Before the first drop of the outflow of the photoreactor entered in contact with the Batch solution, LiOtBu 1 M in dry THF (0.550 mL, 0.55 mmol, 1.1 equiv) was added in one portion to the latter to obtain a final concentration of benzaldehyde of 0.54 M. After all Feed A was added, the solution was kept stirring at room temperature for the indicated time. Eventually, the solution was quenched with sat'd NH_4Cl solution. The mixture was extracted three times with Ethyl Acetate and the organic phases were collected and dried over Na_2SO_4. After filtration and rotary evaporation, the crude was analyzed via ^{1}H NMR to determine the reaction yield (CH_2Br_2 as the external standard) and diastereomeric ratio and then purified via column chromatography.
This procedure was used for compounds **24-35**, **40-47**, **53**.

General procedure for the allylation with aromatic aldehydes – telescoped (GP5)

In a typical experiment, ethyl 2-(diethoxyphosphoryl)acrylate (**2**, 118 mg, 0.5 mmol), **1a** (288 µL, 2.5 mmol, 5 equiv) and TBADT (17 mg, 1 mol%) were dissolved in CH_3CN (1.7 mL) in an oven-dried 7 mL vial. The vial was sealed with a rubber septum and the solution was sparged with N_2 (2 min). The mixture was taken

up with a 5 mL syringe and mounted on a syringe pump (Feed A) connected to a Vapourtec system UV-150 equipped with a 1.35 mL PFA coil (ID = 0.75 mm) and 60 W 365 nm LEDs.

Parallelly, in an oven-dried vial a stock solution 0.54 M in 2-(trifluoromethyl)benzaldehyde **S6** and 0.40 M in LiOtBu (a 1 M in dry THF solution was used) was prepared. This stock solution was taken up with a 10 mL syringe and mounted on a syringe pump (Feed B).

Feed A was pumped at 0.270 mL min^{-1} through the Vapourtec system (V = 1.35 mL, τ_R=5 min). The blue outflow of the latter (due to the reduced form of the photocatalyst, TBADT) was then mixed with Feed B (pumped at 0.190 mL min^{-1}) through a PEEK T-mixer. When the outflow of the photoreactor turned back to colorless (marking the end of the photoreaction), neat acetonitrile was used to push the combined feeds into a 14.4 mL PFA coil (ID = 0.75 mm) at 0.46 mL min^{-1} at 40 °C (τ_R = 30 min) or 0.24 mL min^{-1} at 60 °C (τ_R = 60 min, BPR: 2.8 bar). Finally, the resulting reaction crude was directly collected into a sat'd NH_4Cl solution for quenching. The mixture was extracted three times with Ethyl Acetate and the organic phases were collected and dried over Na_2SO_4. After filtration and rotary evaporation, the crude was analyzed via ^{1}H NMR to determine the reaction yield (CH_2Br_2 as the external standard) and diastereomeric ratio and then purified via column chromatography.

This procedure was used for compounds **24**, **26-30**, **33**, **35-41**, **45**.

General procedure for the allylation with aliphatic aldehydes – fed-batch (GP6)

In a typical experiment, ethyl 2-(diethoxyphosphoryl)acrylate (**2**, 118 mg, 0.5 mmol), **1a** (288 µL, 2.5 mmol, 5 equiv) and TBADT (17 mg, 1 mol%) were dissolved in CH_3CN (1.7 mL) in an oven-dried 7 mL vial. The vial was sealed with a rubber septum and the solution was sparged with N_2 (2 min). The mixture was taken up with a 5 mL syringe and mounted on a syringe pump (Feed A) connected to a Vapourtec system UV-150 equipped with a 3.06 mL PFA coil (ID = 0.75 mm) and 60 W 365 nm LEDs.

Parallelly, in an oven-dried vial a solution 0.084 M in LiOtBu (a 1 M in dry THF solution was used) was prepared. The solution was kept under inert atmosphere with a balloon filled with N_2 (Batch).

Feed A was pumped at 0.612 mL min^{-1} through the Vapourtec system (V = 3.06 mL, τ_R=5 min) and completely collected in the Batch solution. After 5 minutes of stirring, heptaldehyde (**1v**, 106 µL, 0.75 mmol, 1.5 equiv, ρ = 0.809 g mL^{-1}) was added in one portion. The resulting solution was kept stirring at room temperature for the indicated time. Eventually, the solution was quenched with sat'd NH_4Cl solution. The mixture was extracted three times with Ethyl Acetate and the organic phases were collected and dried over Na_2SO_4. After filtration and rotary evaporation, the crude was analyzed via ^{1}H NMR to determine the reaction yield (CH_2Br_2 as the external standard) and diastereomeric ratio and then purified via column chromatography.

This procedure was used for compounds **48-52**, **54-55**.

General procedure for the synthesis of compounds 59−61 (GP7)

In a typical experiment, diethyl (1-phenylvinyl)phosphonate (**2''''**, 48 mg, 0.2 mmol), **1a** (115 µL, 1.0 mmol, 5 equiv) and TBADT (7 mg, 1 mol%) were dissolved in CH_3CN (0.89 mL) in an oven-dried 7 mL vial. The vial was sealed with a rubber septum and the solution was sparged with N_2 (2 min). The mixture was taken up with a 5 mL syringe and mounted on a syringe pump (Feed A) pumped at 0.204 mL min^{-1} (τ_R=15 min) into a Vapourtec system UV-150 equipped with a 3.06 mL PFA coil (ID = 0.75 mm) and 60 W 365 nm LEDs. The blue outflow was collected in a 10 mL round-bottom flask and the solvent was removed under vacuum. The crude was analyzed via ^{1}H NMR to determine the reaction yield (CH_2Br_2 as the external standard), the

deuterated solvent was removed and the crude was stripped twice with neat tetrahydrofuran (2x5 mL) to remove traces of MeCN. Next, an oven-dried magnetic stirrer was added and the flask was sealed with a septum; an inert atmosphere was applied. Dry THF (2 mL) was added to redissolve the crude and the solution was cooled at −78 °C. BuLi (1.2 equiv.) was added dropwise at low temperature and the resulting green solution was kept stirring for 10 minutes, then the aldehyde (dissolved in 1 mL of dry THF) was added and the flask was removed from the cold bath and stirred at room temperature overnight. Finally, the reaction was quenched with a saturated aqueous NH_4Cl solution. The mixture was extracted three times with Ethyl Acetate and the organic phases were collected and dried over Na_2SO_4. After filtration and rotary evaporation, the crude was analyzed via ^1H NMR to determine the reaction yield (CH_2Br_2 as the external standard) and diastereomeric ratio and then purified via column chromatography.

7. Characterization data of synthesized compounds

Characterization data of alkylphosphonates 3a, 3e, 3f, 3n-q, 3u, 3v

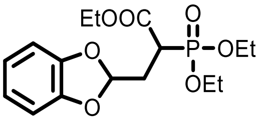

ethyl 3-(benzo[d][1,3]dioxol-2-yl)-2-(diethoxyphosphoryl)propanoate (3a). Prepared according to GP1 from **1a** (288 µL, 2.5 mmol, 5.0 equiv, ρ = 1.06 g mL^{-1}) and **2** (118 mg, 0.5 mmol). Purified via flash column chromatography on silica gel (Cyclohexane:Ethyl Acetate 1:1) to afford the product as light yellow oil (148 mg, 83%). ^{1}H NMR (400 MHz, $CDCl_3$) δ 6.84 – 6.69 (m, 4H), 6.19 (dd, *J* = 5 Hz, J_2 = 4 Hz, 1H), 4.28 – 4.09 (m, 6H), 3.34 – 3.20 (m, 1H), 2.72 (dddd, J_1 = 15 Hz, J_2 = 11 Hz, J_3 = 7 Hz, J_4 = 4 Hz, 1H), 2.44 (dddd, J_1 = 15 Hz, J_2 = 12 Hz, J_3 = 5 Hz, J_4 = 3 Hz, 1H), 1.34 (t, J_1 = 7 Hz, 3H), 1.33 (t, J_1 = 7 Hz, 3H), 1.24 (t, *J* = 7 Hz, 3H). ^{13}C NMR (101 MHz, $CDCl_3$) δ 168.5, 168.4, 147.2, 147.1, 121.6, 121.5, 109.1, 108.9, 108.6, 108.5, 63.1, 63.0, 62.9, 62.8, 61.6, 40.3, 39.0, 31.7, 31.6, 16.3, 16.3, 16.2, 16.2, 13.9. ^{31}P{^{1}H} NMR (162 MHz, $CDCl_3$) δ 21.7. HRMS (ESI+) (m/z): $[M+H]^+$ calcd. for $C_{16}H_{23}N_7P$, 359.1260; found: 359.1247.

When the same reaction was performed with only 1 equivalent of **1a**, product **3a** was formed in 65% yield (^{1}H-NMR, CH_2Br_2 as the external standard).

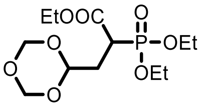

ethyl 2-(diethoxyphosphoryl)-3-(1,3,5-trioxan-2-yl)propanoate (3e). Prepared according to GP1 from **1e** (225 mg, 2.5 mmol, 5.0 equiv) and **2** (118 mg, 0.5 mmol). Purified via flash column chromatography on silica gel (Cyclohexane:Ethyl Acetate 1:1) to afford the product as light colorless oil (90 mg, 55%). ^{1}H NMR (400 MHz, $CDCl_3$) δ 5.17 – 5.09 (m, 2H), 5.04 – 4.92 (m, 3H), 4.23 – 4.05 (m, 6H), 3.27 – 3.11 (m, 1H), 2.39 (dddd, J_1 = 15 Hz, J_2 = 11 Hz, J_3 = 8 Hz, J_4 = 4 Hz, 1H), 2.14 (dddd, J_1 = 14 Hz, J_2 = 12 Hz, J_3 = 5 Hz, J_4 = 3 Hz, 1H), 1.35 – 1.17 (m, 9H). ^{13}C NMR (101 MHz, $CDCl_3$) δ 168.8, 168.7, 99.8, 99.6, 93.2, 93.2, 63.0, 63.0, 62.9, 62.8, 61.5, 40.6, 39.2, 31.6, 31.5, 16.4, 16.4, 16.3, 16.3, 14.1. ^{31}P{^{1}H} NMR (162 MHz, $CDCl_3$) δ 22.1. HRMS (ESI+) (m/z): $[M+H]^+$ calcd. for $C_{12}H_{23}O_8P$, 327.1209; found: 327.1197.

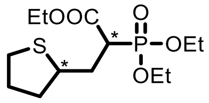

ethyl 2-(diethoxyphosphoryl)-3-(tetrahydrothiophen-2-yl)propanoate (3f). Prepared according to GP1 from **1f** (220 µL, 2.5 mmol, 5.0 equiv, ρ = 0.999 g mL^{-1}) and **2** (118 mg, 0.5 mmol). Purified via flash column chromatography on silica gel (Hexane:Ethyl Acetate 1:2) to afford the product as a colorless oil (104 mg, 64%) as an inseparable mixture of two diastereomers in a 2:1 ratio. ^{1}H NMR (400 MHz, $CDCl_3$) δ 4.29 – 4.07 (m, 12H, *major+minor*), 3.45 – 3.28 (m, 2H, *major+minor*), 3.21 – 3.09 (m, 1H, *major*), 3.08 – 2.98 (m, 1H, *minor*), 2.90 – 2.78 (m, 4H), 2.37 (dddd, J_1 = 13 Hz, J_2 = 11 Hz, J_3 = 7 Hz, J_4 = 4 Hz, 2H,

major+minor), 2.29 – 1.81 (m, 8H, *major+minor*), 1.70 – 1.50 (m, 2H *major+minor*), 1.38 – 1.22 (m, 18H, *major+minor*). ^{13}C NMR (101 MHz, $CDCl_3$) δ 169.1 (*minor*), 169.1 (*minor*), 169.1 (*major*), 169.0 (*major*), 63.0 (*major+minor*), 62.9 (1x *major*+2x *minor*), 62.8 (*major+minor*), 62.80 (*major*), 61.7 (*minor*), 61.6 (*major*), 47.6 (*major*), 47.4 (*major*), 47.15 (*minor*), 47.02 (*minor*), 45.7 (*major+minor*), 44.4 (*major+minor*), 37.4 (*major*), 36.5 (*minor*), 34.8 (*major*), 34.8 (*major*), 34.7 (*minor*), 34.6 (*minor*), 32.4 (*minor*), 32.4 (*major*), 30.2 (*minor*), 30.1 (*major*), 16.5 (4x *major*+4x *minor*), 14.3 (*major*), 14.2 (*minor*). ^{31}P{^{1}H} NMR (162 MHz, $CDCl_3$) δ 22.4 (*major*), 22.3 (*minor*).

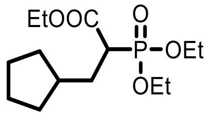

ethyl 3-cyclopentyl-2-(diethoxyphosphoryl)propanoate (3n). Prepared according to GP1 from **1n** (933 μL, 10.0 mmol, 20.0 equiv, ρ = 0.751 g mL^{-1}) and **2** (118 mg, 0.5 mmol). Purified via flash column chromatography on silica gel (Cyclohexane:Ethyl Acetate 7:3 → 4:6) to afford the product as a yellowish oil (108 mg, 71%). ^{1}H NMR (400 MHz, $CDCl_3$) δ 4.22 – 4.06 (m, 6H), 3.03 – 2.89 (m, 1H), 2.14 – 2.01 (m, 1H), 1.81 – 1.64 (m, 4H), 1.63 – 1.52 (m, 2H), 1.52 – 1.42 (m, 2H), 1.30 (t, *J* = 7 Hz, 3H), 1.29 (t, *J* = 7 Hz, 3H), 1.25 (t, *J* = 7 Hz, 3H), 1.14 – 0.97 (m, 2H). ^{13}C NMR (101 MHz, $CDCl_3$) δ 169.6, 169.5, 62.8, 62.7, 62.7, 61.4, 46.0, 44.7, 39.1, 39.0, 33.0, 33.0, 32.8, 31.8, 25.2, 25.0, 16.5, 16.5, 16.4, 16.4, 14.2. ^{31}P{^{1}H} NMR (162 MHz, $CDCl_3$) δ 23.1. HRMS (ESI+) (m/z): $[M+H]^+$ calcd. for $C_{14}H_{27}O_5P$, 307.1674; found: 307.1684.

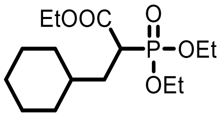

ethyl 3-cyclohexyl-2-(diethoxyphosphoryl)propanoate (3o). Prepared according to GP1 from **1o** (1.1 mL, 10.0 mmol, 20.0 equiv, ρ = 0.774 g mL^{-1}) and **2** (118 mg, 0.5 mmol). Purified via flash column chromatography on silica gel (Cyclohexane:Ethyl Acetate 1:1) to afford the product as colorless oil (103 mg, 64%). ^{1}H NMR (400 MHz, $CDCl_3$) δ 4.15 (q, *J* = 7 Hz, 2H), 4.1 – 4.0 (m, 4H), 3.11 – 2.87 (m, 1H), 1.97 – 1.83 (m, 1H), 1.77 – 1.50 (m, 6H), 1.27 (t, *J* = 7 Hz, 3H), 1.26 (t, *J* = 7 Hz, 3H), 1.22 (t, *J* = 7 Hz, 3H), 1.20 – 0.99 (m, 4H), 0.94 – 0.72 (m, 2H). ^{13}C NMR (101 MHz, $CDCl_3$) δ 169.3, 169.3, 62.5, 62.5, 62.4, 62.4, 61.1, 43.8, 42.5, 36.2, 36.1, 33.9, 33.9, 33.3, 31.8, 26.2, 25.9, 25.8, 16.2, 16.2, 16.2, 16.1, 14.0. ^{31}P{^{1}H} NMR (162 MHz, $CDCl_3$) δ 23.5. HRMS (ESI+) (m/z): $[M+H]^+$ calcd. for $C_{15}H_{29}O_5P$, 321.1831; found: 321.1827.

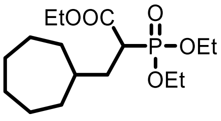

ethyl 3-cycloheptyl-2-(diethoxyphosphoryl)propanoate (3p). Prepared according to GP1 from **1p** (303 μL, 2.5 mmol, 5.0 equiv, ρ = 0.811 g mL^{-1}) and **2** (118 mg, 0.5 mmol). Purified via flash column chromatography on silica gel (Cyclohexane:Ethyl Acetate 1:1) to afford the product as colorless oil (100 mg, 60%). ^{1}H NMR (400 MHz, $CDCl_3$) δ 4.20 (q, *J* = 7 Hz, 2H), 4.17 – 4.08 (m, 4H), 3.12 – 2.84 (m, 1H), 1.95

(dddd, J_1 = 13 Hz, J_2 = 11 Hz, J_3 = 8 Hz, J_4 = 5 Hz, 1H), 1.72 – 1.34 (m, 12H), 1.31 (t, J = 7 Hz, 3H), 1.31 (t, J = 7 Hz, 3H), 1.27 (t, J = 7 Hz, 3H), 1.24 – 1.04 (m, 2H). ^{13}C NMR (101 MHz, $CDCl_3$) δ 169.6, 169.6, 62.8, 62.8, 62.7, 62.7, 61.4, 44.6, 43.3, 37.9, 37.8, 35.2, 34.7, 34.7, 32.9, 28.6, 28.4, 26.3, 26.1, 16.5, 16.5, 16.5, 16.4, 14.3. ^{31}P{^{1}H} NMR (162 MHz, $CDCl_3$) δ 23.5. HRMS (ESI+) (m/z): $[M+H]^+$ calcd. for $C_{16}H_{31}O_5P$, 335.1987; found: 335.1980.

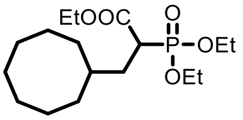

ethyl 3-cyclooctyl-2-(diethoxyphosphoryl)propanoate (3q). Prepared according to GP1 from **1q** (336 µL, 2.5 mmol, 5.0 equiv, ρ = 0.834 g mL^{-1}) and **2** (118 mg, 0.5 mmol). Purified via flash column chromatography on silica gel (Cyclohexane:Ethyl Acetate 1:1) to afford the product as colorless oil (106 mg, 61%). ^{1}H NMR (400 MHz, $CDCl_3$) δ 4.27 – 4.04 (m, 6H), 3.12 – 2.96 (m, 1H), 1.93 (dddd, J_1 = 14 Hz, J_2 = 11 Hz, J_3 = 8 Hz, J_4 = 4 Hz, 1H), 1.75 – 1.36 (m, 14H), 1.32 (t, J = 7 Hz, 3H), 1.31 (t, J = 7 Hz, 3H), 1.27 (t, J = 7 Hz, 3H), 1.30 – 1.17 (m, 2H). ^{13}C NMR (101 MHz, $CDCl_3$) δ 169.6, 169.5, 62.8, 62.7, 62.6, 61.3, 44.8, 43.1, 35.9, 35.8, 34.7, 34.6, 33.3, 30.3, 27.4, 27.0, 26.3, 25.5, 25.1, 16.5, 16.5, 16.4, 16.4, 14.3. ^{31}P{^{1}H} NMR (162 MHz, $CDCl_3$) δ 23.5. HRMS (ESI+) (m/z): $[M+H]^+$ calcd. for $C_{17}H_{33}O_5P$, 349.2144; found: 349.2145.

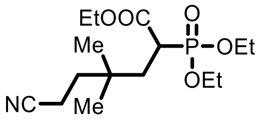

ethyl 6-cyano-2-(diethoxyphosphoryl)-4,4-dimethylhexanoate (3u). Prepared according to GP1 from **1u** (1.2 mL, 10.0 mmol, 20.0 equiv, ρ = 0.8 g mL^{-1}) and **2** (118 mg, 0.5 mmol). Purified via flash column chromatography on silica gel (Cyclohexane:Ethyl Acetate 4:1 → 1:1) to afford the product as colorless oil (153 mg, 92%). ^{1}H NMR (400 MHz, $CDCl_3$) δ 4.17 (q, J = 7 Hz, 2H), 4.14 – 4.03 (m, 4H), 2.97 – 2.82 (m, 1H), 2.28 – 2.23 (m, 2H), 2.04 (ddd, J_1 = 15 Hz, J_2 = 11 Hz, J_3 = 3 Hz, 1H), 1.69 (ddd, J_1 = 16 Hz, J_2 = 15 Hz, J_3 = 1 Hz, 1H), 1.61 – 1.48 (m, 2H), 1.29 (t, J = 7 Hz, 3H), 1.28 (t, J = 7 Hz, 3H), 1.25 (t, J = 7 Hz, 3H), 0.84 (s, 6H).^{13}C NMR (101 MHz, $CDCl_3$) δ 170.0, 169.9, 120.1, 63.08, 63.0, 62.9, 62.9, 61.8, 42.2, 40.9, 37.0, 36.9, 36.8, 33.7, 33.5, 26.0, 25.9, 16.4, 16.4, 16.4, 16.3, 14.1, 12.3. ^{31}P{^{1}H} NMR (162 MHz, $CDCl_3$) δ 23.1. HRMS (ESI+) (m/z): $[M+H]^+$ calcd. for $C_{15}H_{28}NO_5P$, 334.1777; found: 334.1783.

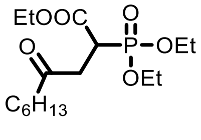

ethyl 2-(diethoxyphosphoryl)-4-oxodecanoate (3v). Prepared according to GP1 from **1v** (70 µL, 0.5 mmol, 1.0 equiv, ρ = 0.82 g mL^{-1}) and **2** (118 mg, 0.5 mmol). Purified via flash column chromatography on silica gel (Cyclohexane:Ethyl Acetate 1:1) to afford the product as colorless oil (130 mg, 74%). ^{1}H NMR (400 MHz, $CDCl_3$) δ 4.21 – 4.00 (m, 6H), 3.49 – 3.35 (m, 1H), 3.16 (ddd, J_1 = 18 Hz, J_2 = 11 Hz, J_3 = 7 Hz, 1H), 2.85 – 2.73 (m, 1H), 2.42 – 2.34 (m, 2H), 1.56 – 1.45 (m, 2H), 1.28 (t, J = 7 Hz, 3H), 1.26 (t, J = 7 Hz, 3H), 1.22 – 1.16 (m, 6H), 1.21 (t, J = 7 Hz, 3H), 0.80 (t, J = 7 Hz, 3H). ^{13}C NMR (101 MHz, $CDCl_3$) δ 207.3, 207.1, 168.2, 168.2, 62.7, 62.7, 62.6, 62.6, 61.4, 42.3, 40.4, 39.1, 39.1, 31.3, 28.6, 23.5, 22.2, 16.2, 16.1,

16.1, 13.8, 13.8. $^{31}P\{^{1}H\}$ NMR (162 MHz, $CDCl_3$) δ 22.6. HRMS (ESI+) (m/z): $[M+H]^+$ calcd. for $C_{16}H_{31}O_6P$, 351.1936; found: 351.1926.

Characterization data of compounds 4-19

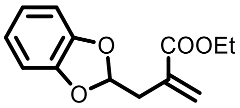

ethyl 2-(benzo[d][1,3]dioxol-2-ylmethyl)acrylate (4). Prepared according to GP2 from **1a** (288 μL, 2.5 mmol, 5.0 equiv, ρ = 1.06 g mL^{-1}) and **2** (118 mg, 0.5 mmol). Purified via flash column chromatography on silica gel (Hexane:Ethyl Acetate 98:2) to afford the product as a yellowish oil (82 mg, 70% yield). ^{1}H NMR (400 MHz, $CDCl_3$) δ 6.85 – 6.74 (m, 4H), 6.38 (d, *J* = 1 Hz, 1H), 6.32 (t, *J* = 5, 1H), 5.81 (d, *J* = 1 Hz, 1H), 4.25 (q, *J* = 7 Hz, 2H), 2.96 (dd, J_1 = 5 Hz, J_2 = 1 Hz, 2H), 1.32 (t, *J* = 7 Hz, 3H). ^{13}C NMR (101 MHz, $CDCl_3$) δ 166.5, 147.4, 133.7, 129.5, 121.6, 109.6, 108.7, 61.2, 37.5, 14.3. HRMS (FI+) (m/z): [M]$^+$ calcd. for $C_{13}H_{14}O_4$, 234.0892; found: 234.0879.

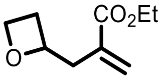

ethyl 2-(oxetan-2-ylmethyl)acrylate (5). Prepared according to GP2 from **1b** (162 μL, 2.5 mmol, 5.0 equiv, ρ = 0.893 g mL^{-1}) and **2** (118 mg, 0.5 mmol). Purified via flash column chromatography on silica gel (Hexane:Ethyl Acetate 98:2 → 90:10) to afford the product as a yellowish oil (42 mg, 70% yield). ^{1}H NMR (400 MHz, $CDCl_3$) δ 6.22 (d, *J* = 1 Hz, 1H), 5.61 (q, *J* = 1 Hz, 1H), 4.96 (qd, J_1 = 7 Hz, J_2 = 6 Hz, 1H), 4.63 (ddd, J_1 = 8 Hz, J_2 = 8 Hz, J_3 = 6 Hz, 1H), 4.49 (dt, J_1 = 9 Hz, J_2 = 6 Hz, 1H), 4.18 (q, *J* = 7 Hz, 2H), 2.82 – 2.68 (m, 2H), 2.66 (m, 1H), 2.36 (m, 1H), 1.27 (t, *J* = 7 Hz, 3H). ^{13}C NMR (101 MHz, $CDCl_3$) δ 167.0, 136.0, 127.1, 80.7, 68.1, 60.9, 40.0, 27.3, 14.3. HRMS (FI+) (m/z): [M]$^+$ calcd. for $C_9H_{14}O_3$, 170.0943; found: 170.0952.

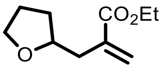

ethyl 2-((tetrahydrofuran-2-yl)methyl)acrylate (6). Prepared according to GP2 from **1c** (203 μL, 2.5 mmol, 5.0 equiv, ρ = 0.889 g mL^{-1}) and **2** (118 mg, 0.5 mmol). Purified via flash column chromatography on silica gel (Hexane:Ethyl Acetate 98:2 → 90:10) to afford the product as a yellowish oil (61 mg, 66% yield). ^{1}H NMR (400 MHz, $CDCl_3$) δ 6.19 (d, *J* = 2 Hz, 1H), 5.63 (q, *J* = 1 Hz, 1H), 4.17 (q, *J* = 7 Hz, 2H), 4.06 – 3.95 (m, 1H), 3.84 (m, 1H), 3.70 (m, 1H), 2.49 (dd, J_1 = 6 Hz, J_2 = 1 Hz, 2H), 2.03 – 1.75 (m, 3H), 1.48 (m, 1H), 1.27 (t, *J* = 7 Hz, 3H). ^{13}C NMR (101 MHz, $CDCl_3$) δ 167.2, 138.0, 126.6, 77.6, 67.8, 60.7, 38.0, 31.2, 25.6, 14.3. HRMS (FI+) (m/z): [M]$^+$ calcd. for $C_{10}H_{16}O_3$, 184.1099; found: 184.1083.

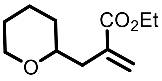

ethyl 2-((tetrahydro-2H-pyran-2-yl)methyl)acrylate (7). Prepared according to GP2 from **1d** (245 μL, 2.5 mmol, 5.0 equiv, ρ = 0.86 g mL^{-1}) and **2** (118 mg, 0.5 mmol). Purified via flash column chromatography on silica gel (Hexane:Ethyl Acetate 90:10 → 80:20) to afford the product as a colorless oil (60 mg, 60% yield). ^{1}H NMR (400 MHz, $CDCl_3$) δ 6.19 (d, *J* = 2 Hz, 1H), 5.61 (q, *J* = 1 Hz, 1H), 4.19 (q, *J* = 7 Hz, 2H), 3.93 (m, 1H), 3.50 – 3.32 (m, 2H), 2.51 – 2.36 (m, 2H), 1.80 (m, 1H), 1.64 – 1.36 (m, 4H) 1.30 – 1.22 (m, 4H).

^{13}C NMR (101 MHz, $CDCl_3$) δ 167.3, 137.5, 126.9, 76.2, 68.6, 60.7, 39.2, 31.9, 26.1, 23.6, 14.3. HRMS (FI+) (m/z): $[M]^+$ calcd. for $C_{11}H_{18}O_3$, 198.1256; found: 198.1248.

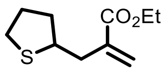

ethyl 2-((tetrahydrothiophen-2-yl)methyl)acrylate (8). Prepared according to GP2 from **1f** (220 µL, 2.5 mmol, 5.0 equiv, ρ = 0.999 g mL^{-1}) and **2** (118 mg, 0.5 mmol). Purified via flash column chromatography on silica gel (Hexane:Ethyl Acetate 20:1 → 15:1) to afford the product as a yellowish oil (70 mg, 70% yield). ^1H NMR (400 MHz, $CDCl_3$) δ 6.17 (d, J = 1 Hz, 1H), 5.59 (q, J = 1 Hz, 1H), 4.17 (q, J = 7 Hz, 2H), 3.54 (m, 1H), 2.91 – 2.77 (m, 2H), 2.66 (ddd, J_1 = 14 Hz, J_2 = 6 Hz, J_3 =1 Hz, 1H), 2.45 (ddd, J_1 = 14 Hz, J_2 = 8 Hz, J_3 =1 Hz, 1H), 2.12 – 1.98 (m, 2H), 1.95 – 1.81 (m, 1H), 1.65 – 1.53 (m, 1H), 1.27 (t, J = 7 Hz, 3H). ^{13}C NMR (101 MHz, $CDCl_3$) δ 167.0, 139.1, 126.4, 60.8, 47.3, 40.0, 37.0, 32.4, 30.2, 14.3. HRMS (FI+) (m/z): $[M]^+$ calcd. for $C_{10}H_{16}O_2S$, 200.0871; found: 200.0879.

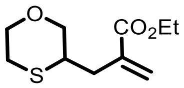

ethyl 2-((1,4-oxathian-3-yl)methyl)acrylate (9). Prepared according to GP2 from **1g** (234 µL, 2.5 mmol, 5.0 equiv, ρ = 1.114 g mL^{-1}) and **2** (118 mg, 0.5 mmol). Purified via flash column chromatography on silica gel (Hexane:Ethyl Acetate 90:10) to afford the product as a colorless oil (56 mg, 52% yield). ^1H NMR (400 MHz, $CDCl_3$) δ 6.25 (d, J = 1 Hz, 1H), 5.62 (q, J = 1 Hz, 1H), 4.20 (qd, J_1 = 7 Hz, J_2 = 1 Hz, 2H), 4.08 – 3.96 (m, 2H), 3.70 (ddd, J_1 = 12 Hz, J_2 = 9 Hz, J_3 =2 Hz, 1H), 3.49 (dd, J_1 = 12 Hz, J_2 = 8 Hz, 1H), 3.03 (m, 1H), 2.72 (m, 1H), 2.62 – 2.50 (m, 2H), 2.41 (ddd, J_1 = 14 Hz, J_2 = 8 Hz, J_3 =1 Hz, 1H), 1.29 (t, J = 7 Hz, 3H). ^{13}C NMR (101 MHz, $CDCl_3$) δ 166.7, 136.9, 127.5, 73.5, 68.6, 61.0, 37.8, 34.4, 26.6, 14.3. HRMS (FI+) (m/z): $[M]^+$ calcd. for $C_{10}H_{16}O_3S$, 216.0820; found: 216.0814.

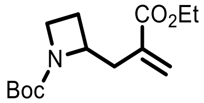

tert-butyl 2-(2-(ethoxycarbonyl)allyl)azetidine-1-carboxylate (10). Prepared according to GP2 from **1h** (393 mg, 2.5 mmol, 5.0 equiv) and **2** (118 mg, 0.5 mmol). Purified via flash column chromatography on silica gel (Hexane:Ethyl Acetate 20:1 → 15:1 → 10:1) to afford the product as a colorless oil (90 mg, 67% yield). ^1H NMR (400 MHz, $CDCl_3$) δ 6.19 (d, J = 1 Hz, 1H), 5.52 (d, J = 1 Hz, 1H), 4.38 – 4.26 (m, 1H), 4.17 (q, J = 7 Hz, 2H), 3.83 – 3.68 (m, 2H), 2.91 (ddd, J_1 = 14 Hz, J_2 = 5 Hz, J_3 =1 Hz, 1H), 2.56 (dd, J_1 = 14 Hz, J_2 = 8 Hz , 1H), 2.19 (m, 1H), 1.83 (m, 1H), 1.40 (s, 9H), 1.27 (t, J = 7 Hz, 3H). ^{13}C NMR (101 MHz, $CDCl_3$) δ 167.0, 156.6, 136.8, 127.0, 79.3, 61.0, 60.8, 46.3, 37.5, 28.5, 21.6, 14.3. HRMS (FI+) (m/z): $[M]^+$ calcd. for $C_{14}H_{23}NO_4$, 269.1627; found: 269.1629.

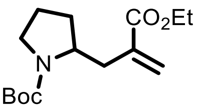

tert-butyl 2-(2-(ethoxycarbonyl)allyl)pyrrolidine-1-carboxylate (11). Prepared according to GP2 from **1i** (438 µL, 2.5 mmol, 5.0 equiv, ρ = 0.977 g mL^{-1}) and **2** (118 mg, 0.5 mmol). Purified via flash column

chromatography on silica gel (Hexane:Ethyl Acetate 98:2 → 90:10) to afford the product as a yellowish oil (94 mg, 66% yield) as a mixture of two conformers (**11a** and **11b**). ^{1}H NMR (400 MHz, $CDCl_3$) δ 6.16 (d, *J* = 1 Hz, 1H), 5.55 – 5.47 (m, 1H), 4.16 (q, *J* = 7 Hz, 2H), 3.97 (m, 1H), 3.44 – 3.18 (m, 2H), 2.75 – 2.51 (m, 1H), 2.45 – 2.19 (m, 1H), 1.88 – 1.72 (m, 3H), 1.69 – 1.60 (m, 1H), 1.41 (s, 9H), 1.26 (t, *J* = 7 Hz, 3H). ^{13}C NMR (101 MHz, $CDCl_3$) δ 167.3 (**11a**), 167.0 (**11b**), 154.6 (**11a**+**11b**), 138.2 (**11a**+**11b**), 127.1 (**11b**), 126.6 (**11a**), 79.3 (**11b**), 78.9 (**11a**), 60.7 (**11a**+**11b**), 57.0 (**11a**), 56.7 (**11b**), 46.5 (**11a**), 45.9 (**11b**), 36.5 (**11b**), 35.2 (**11a**), 30.2 (**11b**), 29.3 (**11a**), 28.5 (**11a**+**11b**), 23.6 (**11a**), 22.7 (**11b**), 14.3 (**11a**+**11b**). HRMS (FI+) (m/z): $[M]^+$ calcd. for $C_{15}H_{25}NO_4$, 283.1784; found: 283.1774.

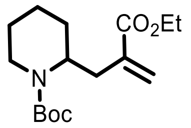

tert-butyl 2-(2-(ethoxycarbonyl)allyl)piperidine-1-carboxylate (12). Prepared according to GP2 from **1j** (463 mg, 2.5 mmol, 5.0 equiv) and **2** (118 mg, 0.5 mmol). Purified via flash column chromatography on silica gel (Hexane:Ethyl Acetate 95:5) to afford the product as a yellowish oil (87 mg, 58% yield). ^{1}H NMR (400 MHz, $CDCl_3$) δ 6.15 (d, *J* = 1 Hz, 1H), 5.52 (s, 1H), 4.40 (bs, 1H), 4.20 (q, *J* = 7 Hz, 2H), 4.00 (bs, 1H), 2.80 (td, J_1 = 13 Hz, J_2 = 3 Hz, 1H), 2.63 (m, 1H), 2.44 (m, 1H), 1.70 – 1.48 (m, 5H), 1.38 (m, 10H), 1.29 (t, *J* = 7 Hz, 3H). ^{13}C NMR (101 MHz, $CDCl_3$) δ 166.9, 155.2, 138.1, 126.9, 79.3, 60.7, 50.0, 38.5, 32.3, 28.9, 28.4, 25.7, 19.1, 14.3. HRMS (FI+) (m/z): $[M]^+$ calcd. for $C_{16}H_{27}NO_4$, 297.1940; found: 297.1927.

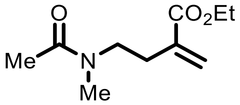

ethyl 4-(N-methylacetamido)-2-methylenebutanoate (13). Prepared according to GP2 from **1k** (185 μL, 2.0 mmol, 4.0 equiv, ρ = 0.94 g mL^{-1}) and **2** (118 mg, 0.5 mmol). Purified via flash column chromatography on silica gel (Hexane:Ethyl Acetate 1:1) to afford the product as light yellow oil (54 mg, 54% yield) as a 1:1 mixture of two rotamers. ^{1}H NMR (400 MHz, $CDCl_3$) δ 6.23 (d, *J* = 1 Hz, 1H), 6.18 (d, *J* = 1 Hz, 1H), 5.60 (m, 2H), 4.21 (q, *J* = 7 Hz, 2H), 4.20 (q, *J* = 7 Hz, 2H), 3.48 (dd, J_1 = 8 Hz, J_2 = 6 Hz, 2H), 3.44 – 3.38 (m, 2H), 2.97 & 2.91 (rotameric singlets, 2x3H), 2.58 – 2.49 (m, 4H), 2.06 & 2.04 (rotameric singlets, 2x3H), 1.30 (t, *J* = 7 Hz, 3H), 1.29 (t, *J* = 7 Hz, 3H). ^{13}C NMR (101 MHz, $CDCl_3$) δ 170.7, 170.6, 167.0, 166.6, 138.0, 137.1, 127.8, 126.8, 61.1, 60.9, 50.3, 47.1, 36.7, 33.4, 31.7, 30.1, 22.0, 21.2, 14.3. HRMS (FI+) (m/z): $[M]^+$ calcd. for $C_{10}H_{17}NO_3$, 199.1208; found: 199.1204.

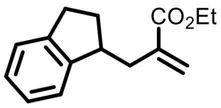

ethyl 2-((2,3-dihydro-1H-inden-1-yl)methyl)acrylate (14). Prepared according to GP2 from **1m** (306 μL, 2.5 mmol, 5.0 equiv, ρ = 0.965 g mL^{-1}) and **2** (118 mg, 0.5 mmol). Purified via flash column chromatography on silica gel (Hexane → Hexane:Ethyl Acetate 98:2) to afford the product colorless oil (37 mg, 32% yield). ^{1}H NMR (400 MHz, $CDCl_3$) δ 7.28 – 7.20 (m, 2H), 7.17 (dd, J_1 = 6 Hz, J_2 = 3 Hz, 2H), 6.24 (d, *J* = 2 Hz, 1H), 5.57 (q, *J* = 1 Hz, 1H), 4.25 (q, *J* = 7 Hz, 2H), 3.38 (dtd, J_1 = 9 Hz, J_2 = 7 Hz, J_3 = 5 Hz, 1H), 3.02 – 2.76 (m, 3H), 2.36 (ddd, J_1 = 14 Hz, J_2 = 9 Hz, J_3 = 1 Hz, 1H), 2.29 – 2.16 (m, 1H), 1.73 (ddt, J_1 = 13 Hz, J_2 = 8 Hz, J_3 = 7 Hz, 1H), 1.33 (t, *J* = 7 Hz, 3H). ^{13}C NMR (101 MHz, $CDCl_3$) δ

167.4, 146.9, 144.0, 139.6, 126.6, 126.2, 126.0, 124.6, 124.0, 60.8, 43.5, 37.9, 31.8, 31.2, 14.4. HRMS (FI+) (m/z): $[M]^+$ calcd. for $C_{15}H_{18}O_2$, 230.1307; found: 230.1301.

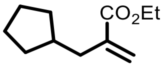

ethyl 2-(cyclopentylmethyl)acrylate (15). Prepared according to GP2 from **1n** (934 µL, 10.0 mmol, 20.0 equiv, ρ = 0.751 g mL^{-1}) and **2** (118 mg, 0.5 mmol). Purified via flash column chromatography on silica gel (Hexane → Hexane:Ethyl Acetate 90:10) to afford the product as a volatile colorless oil (46 mg, 50% yield). ^{1}H NMR (400 MHz, $CDCl_3$) δ 6.11 (d, J = 2 Hz, 1H), 5.50 (d, J = 1 Hz, 1H), 4.19 (q, J = 7 Hz, 2H), 2.29 (d, J = 7 Hz, 2H), 2.02 (hept, J = 8 Hz, 1H), 1.78 – 1.67 (m, 2H), 1.66 – 1.44 (m, 4H), 1.29 (t, J = 7 Hz, 3H), 1.19 – 1.05 (m, 2H). ^{13}C NMR (101 MHz, $CDCl_3$) δ 167.7, 140.7, 124.9, 60.7, 38.7, 38.2, 32.5, 25.1, 14.3. HRMS (FI+) (m/z): $[M]^+$ calcd. for $C_{11}H_{18}O_2$, 182.1307; found: 182.1309.

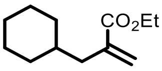

ethyl 2-(cyclohexylmethyl)acrylate (16). Prepared according to GP2 from **1o** (544 µL, 5.0 mmol, 10.0 equiv, ρ = 0.774 g mL^{-1}) and **2** (118 mg, 0.5 mmol. Purified via flash column chromatography on silica gel (Hexane:Ethyl Acetate 98:2) to afford the product as colorless oil (52 mg, 53% yield). ^{1}H NMR (400 MHz, $CDCl_3$) δ 6.13 (d, J = 2 Hz, 1H), 5.46 (q, J = 1 Hz, 1H), 4.19 (q, J = 7 Hz, 2H), 2.18 (dd, J_1 = 7 Hz, J_2 = 1 Hz, 2H), 1.75 – 1.59 (m, 5H), 1.52 – 1.36 (m, 1H), 1.29 (t, J = 7 Hz, 3H), 1.25 – 1.06 (m, 3H), 0.98 – 0.77 (m, 2H). ^{13}C NMR (101 MHz, $CDCl_3$) δ 167.7, 139.7, 125.5, 60.7, 40.0, 36.8, 33.2, 26.7, 26.4, 14.3. Spectroscopic data are in accordance with those reported in the literature.[9]

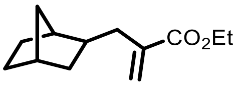

ethyl 2-(((1S,4R)-bicyclo[2.2.1]heptan-2-yl)methyl)acrylate (17). Prepared according to GP2 from **1r** (240 mg, 2.5 mmol, 5.0 equiv) and **2** (118 mg, 0.5 mmol). Purified via flash column chromatography on silica gel (Hexane → Hexane:Ethyl Acetate 98:2) to afford the product as colorless oil (46 mg, 44% yield). ^{1}H NMR (400 MHz, $CDCl_3$) δ 6.13 (d, J = 2 Hz, 1H), 5.47 (q, J = 1 Hz, 1H), 4.19 (q, J = 7 Hz, 2H), 2.26 (ddd, J_1 = 15 Hz, J_2 = 8 Hz, J_3 = 1 Hz, 1H), 2.20 – 2.17 (m, 1H), 2.08 (ddd, J_1 = 15 Hz, J_2 = 8 Hz, J_3 = 1 Hz, 1H), 1.99 – 1.93 (m, 1H), 1.68 – 1.56 (m, 1H), 1.53 – 1.35 (m, 3H), 1.35 – 1.25 (m, 4H), 1.21 – 0.97 (m, 4H). ^{13}C NMR (101 MHz, $CDCl_3$) δ 167.7, 140.0, 125.0, 60.7, 40.8, 40.5, 38.7, 37.9, 36.8, 35.3, 30.0, 28.9, 14.3. Spectroscopic data are in accordance with those reported in the literature.[10]

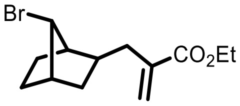

***exo*-Ethyl 2-(((1R,4S)-7-bromobicyclo[2.2.1]heptan-2-yl)methyl)acrylate (18).** Prepared according to GP2 from **1s** (317 µL, 2.5 mmol, 5.0 equiv, ρ = 1.38 g mL^{-1}) and **2** (118 mg, 0.5 mmol). Purified via flash column chromatography on silica gel (Hexane:Ethyl Acetate 98:2) to afford the product as a volatile light yellow oil (72 mg, 50% yield). ^{1}H NMR (400 MHz, $CDCl_3$) δ 6.16 (s, 1H), 5.48 (s, 1H), 4.19 (q, J = 7 Hz, 2H), 4.06 (t, J = 2 Hz, 1H), 2.38 – 2.26 (m, 2H), 2.15 (ddd, J_1 = 15 Hz, J_2 = 8 Hz, J_3 = 1 Hz, 1H), 2.08 (d, J = 4 Hz, 1H), 2.03 – 1.90 (m, 2H), 1.87 – 1.76 (m, 1H), 1.57 (dd, J_1 = 13 Hz, J_2 = 9 Hz, 1H), 1.34 – 1.14 (m,

6H). ^{13}C NMR (101 MHz, $CDCl_3$) δ 167.2, 139.1, 125.7, 60.9, 57.1, 47.1, 43.6, 39.3, 38.3, 35.7, 28.1, 26.7, 14.3. HRMS (FI+) (m/z): $[M]^+$ calcd. for $C_{13}H_{19}BrO_2$, 286.0568; found: 286.0568. NOESY experiment is reported below.

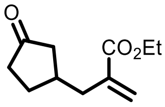

ethyl 2-((3-oxocyclopentyl)methyl)acrylate (19). Prepared according to GP2 from **1t** (221 µL, 2.5 mmol, 5.0 equiv, ρ = 0.950 g mL^{-1}) and **2** (118 mg, 0.5 mmol). Purified via flash column chromatography on silica gel (Hexane:Ethyl Acetate 98:2 → 80:20) to afford the product as a volatile colorless oil (52 mg, 53% yield). ^{1}H NMR (400 MHz, $CDCl_3$) δ 6.17 (d, J = 1 Hz, 1H), 5.54 (q, J = 1 Hz, 1H), 4.18 (q, J = 7 Hz, 2H), 2.49 – 2.20 (m, 5H), 2.20 – 2.04 (m, 2H), 1.89 – 1.74 (m, 1H), 1.61 – 1.46 (m, 1H), 1.28 (t, J = 7 Hz, 3H). ^{13}C NMR (101 MHz, $CDCl_3$) δ 219.1, 167.0, 139.0, 126.1, 60.9, 44.9, 38.4, 37.8, 36.0, 29.2, 14.3. HRMS (FI+) (m/z): $[M]^+$ calcd. for $C_{11}H_{16}O_3$, 196.1099; found: 196.1106.

Characterization data of compounds 4-d2, 20-23

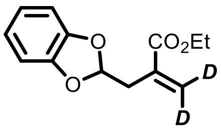

ethyl 2-(benzo[d][1,3]dioxol-2-ylmethyl)acrylate-d2 (4-d2). Prepared according to GP3 from **1a** (288 μL, 2.5 mmol, 5 equiv, ρ = 1.06 g mL^{-1}) and **2** (118 mg, 0.5 mmol). Purified by flash column chromatography on silica gel (Hexane:Ethyl Acetate 30:1 → 10:1) to afford the product as colorless oil (80 mg, 68%). ^1H NMR (400 MHz, $CDCl_3$) δ 6.83 – 6.72 (m, 4H), 6.31 (t, *J* = 5 Hz, 1H), 4.25 (q, *J* = 7 Hz, 2H), 2.96 (d, *J* = 5 Hz, 2H), 1.32 (t, *J* = 7 Hz, 3H). ^{13}C NMR (101 MHz, $CDCl_3$) δ 166.6, 147.5, 133.6, 128.8 (pent, *J* = 24 Hz), 121.6, 109.6, 108.7, 61.2, 37.4, 14.3. HRMS (FI+) (m/z): $[M]^+$ calcd. for $C_{13}H_{12}D_2O_4$, 236.1018; found: 236.1011.

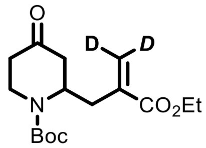

tert-butyl 2-(2-(ethoxycarbonyl)allyl-3,3-d2)-4-oxopiperidine-1-carboxylate (20). Prepared according to GP3 from **1w** (498 mg, 2.5 mmol, 5 equiv) and **2** (118 mg, 0.5 mmol) and TBADT (81 mg, 0.05 mol). Purified by flash column chromatography on silica gel (Hexane:Ethyl Acetate 5:1) to afford the product as a yellowish oil (68 mg, 43%). ^1H NMR (400 MHz, $CDCl_3$) δ 4.83 (s, 1H), 4.38 (s, 1H), 4.20 (q, *J* = 7 Hz, 2H), 3.27 – 3.13 (m, 1H), 2.66 (dd, J_1 = 15 Hz, J_3 = 7 Hz, 1H), 2.55 – 2.39 (m, 3H), 2.32 (d, *J* = 15 Hz, 2H), 1.42 (s, 9H), 1.29 (t, *J* = 7 Hz, 3H). ^{13}C NMR (101 MHz, $CDCl_3$) δ 208.1, 166.4, 154.6, 136.5, 80.6, 61.0, 51.5, 45.3, 40.7, 38.1, 34.9, 28.3, 14.3. CD_2 could not be detected even after 4096 scans. HRMS (FD) (m/z): $[M]^+$ calcd for $C_{16}H_{23}D_2NO_5$: 313.1858; found: 313.1852.

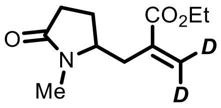

ethyl 2-((1-methyl-5-oxopyrrolidin-2-yl)methyl)acrylate-d2 (21). Prepared according to GP3 from **1l** (241 μL, 2.5 mmol, 5 equiv, ρ = 1.028 g mL^{-1}) and **2** (118 mg, 0.5 mmol). Purified by flash column chromatography on silica gel (Hexane:Ethyl Acetate 4:1) to afford the product as a yellowish oil (55 mg, 52%). ^1H NMR (400 MHz, $CDCl_3$) δ 4.20 (qd, J_1 = 7 Hz, J_2 = 3 Hz, 2H), 3.67 (dt, J_1 = 8 Hz, J_2 = 4 Hz, 1H), 2.97 – 2.72 (m, 4H), 2.37 (dt, J_1 = 17 Hz, J_2 = 8 Hz, 1H), 2.26 (td, J_1 = 11 Hz, J_2 = 5 Hz, 1H), 2.22 – 2.09 (m, 1H), 2.03 (ddt, J_1 = 13 Hz, J_2 = 10 Hz, J_1 = 8 Hz, 1H), 1.67 (dp, J_1 = 14 Hz, J_2 = 5 Hz, 1H), 1.29 (t, *J* = 7 Hz, 3H). ^{13}C NMR (101 MHz, $CDCl_3$) δ 175.0, 166.7, 136.5, 127.4 (pent, *J* = 24 Hz), 61.1, 59.0, 36.0, 29.7, 28.1, 23.5, 14.3. HRMS (FI+) (m/z): $[M]^+$ calcd. for $C_{11}H_{15}D_2NO_3$, 213.1334; found: 213.1342.

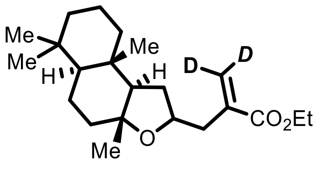

ethyl 2-(((3aR,5aS,9aS,9bR)-3a,6,6,9a-tetramethyldodecahydronaphtho[2,1-b]furan-2-yl)methyl)acrylate-d$_2$ (22). Prepared according to GP3 from **1x** (591 mg, 2.5 mmol, 5 equiv) and **2** (118 mg, 0.5 mmol). Purified by flash column chromatography on silica gel (Hexane:Ethyl Acetate 3:1) to afford the product as an inseparable mixture of diastereoisomers (2:1 ratio by LC-MS) as a colorless oil (70 mg, 40%) along with 451 mg of **1x**. ^{1}H NMR (400 MHz, CDCl$_3$) δ 4.35 – 3.99 (m, 3H), 2.63 – 2.36 (m, 2H), 1.92 (dt, J_1 = 11 Hz, J_2 = 3 Hz, 1H), 1.87 – 1.78 (m, 1H), 1.75 – 1.68 (m, 1H), 1.66 – 1.58 (m, 1H), 1.53 – 1.35 (m, 6H), 1.28 (td, J_1 = 7 Hz, J_2 = 2 Hz, 4H), 1.15 (d, J = 8 Hz, 2H), 1.10 (s, 2H), 1.06 – 0.88 (m, 2H), 0.86 (m, 3H), 0.84 – 0.79 (m, 6H). ^{13}C NMR (101 MHz, CDCl$_3$) δ 167.4 (*major*), 167.3 (*minor*), 137.8 (*minor*), 137.7 (*major*), 126.2 (*major*, pent, J = 23 Hz), 81.2 (*major*), 80.7 (*minor*), 77.1 (*minor*), 74.2 (*major*), 61.0 (*minor*), 60.8 (*major*), 59.0 (*major*), 57.4 (*major*), 57.2 (*minor*), 42.6 (*major*), 40.6 (*major*), 40.6 (*minor*), 40.1 (*major*), 40.1 (*minor*), 39.3 (*major*), 36.4 (*minor*), 36.2 (*major*), 33.7 (*major*), 33.6 (*minor*), 33.2 (*major*), 33.2 (*minor*), 29.4 (*minor*), 28.0 (*major*), 25.0 (*minor*), 21.7 (*major*), 21.2 (*major*), 21.2 (*minor*), 20.9 (*minor*), 20.7 (*major*), 18.5 (*major*), 18.5 (*minor*), 15.7 (*minor*), 15.1 (*major*), 14.3 (*major*), 14.3 (*minor*). HRMS (FI+) (m/z): $[M]^+$ calcd. for $C_{22}H_{34}D_2O_3$, 350.2790; found: 350.2785.

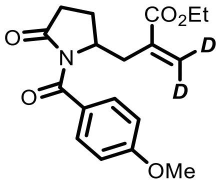

ethyl 2-((1-(4-methoxybenzoyl)-5-oxopyrrolidin-2-yl)methyl)acrylate-d$_2$ (23). Prepared according to GP3 from **1y** (548 mg, 2.5 mmol, 5 equiv) and **2** (118 mg, 0.5 mmol). Purified by flash column chromatography on silica gel (Hexane:Ethyl Acetate 3:1) to afford the product as a colorless oil (35 mg, 21%). ^{1}H NMR (400 MHz, CDCl$_3$) δ 7.63 (d, J = 9 Hz, 2H), 6.89 (d, J = 9 Hz, 2H), 4.71 (tt, J_1 = 8 Hz, J_2 = 5 Hz, 1H), 4.21 (tq, J_1 = 7 Hz, J_2 = 3 Hz, 2H), 3.85 (s, 3H), 2.94 (dd, J_1 = 14 Hz, J_2 = 5 Hz, 1H), 2.71 – 2.56 (m, 2H), 2.47 (ddd, J_1 = 18 Hz, J_2 = 9 Hz, J_3 = 6 Hz, 1H), 2.26 – 2.07 (m, 1H), 1.91 (dq, J_1 = 13 Hz, J_2 = 5 Hz, 1H), 1.31 (t, J = 7 Hz, 3H). ^{13}C NMR (101 MHz, CDCl$_3$) δ 175.1, 170.1, 166.9, 163.1, 136.7, 131.8, 126.8, 113.3, 61.2, 57.2, 55.5, 35.7, 32.1, 23.1, 14.3. CD_2 could not be detected even after 4096 scans. HRMS (FI+) (m/z): $[M]^+$ calcd. for $C_{18}H_{19}D_2NO_5$, 333.1522; found: 333.1540.

Characterization data of compounds 24-47

N.B.: A complete characterization of the two isomers is reported when d.r. was < 4:1. When the geometry of the double bond could not be determined directly via NOESY spectroscopy, the geometry of the isomers was assigned in analogy with other compounds for which a 3D NMR complete characterization of both isomers was possible (e.g. ***25, 30, 34, 42****).*

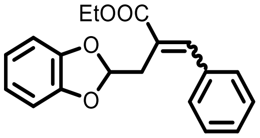

ethyl 2-(benzo[d][1,3]dioxol-2-ylmethyl)-3-phenylacrylate (24). Prepared according to GP4 from **1a** (288 µL, 2.5 mmol, 5.0 equiv, ρ = 1.06 g mL^{-1}), **2** (118 mg, 0.5 mmol) and **S2** (76 µL, 0.75 mmol, 1.5 equiv, ρ = 1.04 g mL^{-1}). Reaction time: 3 h. *E*:*Z* ratio was determined via ^{1}H NMR of the crude to be 2:1. Purified via flash column chromatography on silica gel (Hexane:Ethyl Acetate 98:2 → 95:5) to afford the two diastereomers (93 mg, 60% combined yield).

<u>*Major (E)*</u>: colorless oil. ^{1}H NMR (400 MHz, $CDCl_3$) δ 7.94 (s, 1H), 7.50 – 7.43 (m, 2H), 7.38 – 7.27 (m, 3H), 6.86 – 6.72 (m, 4H), 6.46 (t, *J* = 5 Hz, 1H), 4.32 (q, *J* = 7 Hz, 2H), 3.19 (dd, J_1 = 5 Hz, J_2 = 1 Hz, 2H), 1.37 (t, *J* = 7 Hz, 3H). ^{13}C NMR (101 MHz, $CDCl_3$) δ 167.8, 147.4, 143.4, 135.2, 129.4, 128.9 128.6, 126.0, 121.6, 110.2, 108.7, 61.3, 33.5, 14.4. HRMS (FI+) (m/z): [M]$^+$ calcd. for $C_{19}H_{18}O_4$, 310.1205; found: 310.1222.

<u>*Minor (Z)*</u>: colorless oil. ^{1}H NMR (400 MHz, $CDCl_3$) δ 7.34 – 7.23 (m, 5H), 6.92 (s, 1H), 6.79 (p, *J* = 2 Hz, 4H), 6.36 (t, *J* = 5 Hz, 1H), 4.12 (q, *J* = 7 Hz, 2H), 3.05 (dd, J_1 = 5 Hz, J_2 = 1 Hz, 2H), 1.08 (t, *J* = 7 Hz, 3H). ^{13}C NMR (101 MHz, $CDCl_3$) δ 168.7, 147.5, 139.4, 136.0, 128.5, 128.2, 128.1, 126.9, 121.6, 109.8, 108.7, 61.0, 40.7, 13.8. HRMS (FI+) (m/z): [M]$^+$ calcd. for $C_{19}H_{18}O_4$, 310.1205; found: 310.1201.

The reaction was also performed using GP5 (HWE: 60 °C, 60 min, 2.8 bar) to afford **24** in 65% yield (*E:Z* 2:1).

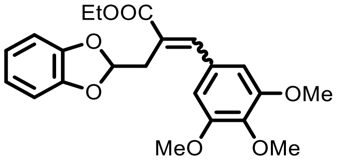

ethyl 2-(benzo[d][1,3]dioxol-2-ylmethyl)-3-(3,4,5-trimethoxyphenyl)acrylate (25). Prepared according to GP4 from **1a** (288 µL, 2.5 mmol, 5.0 equiv, ρ = 1.06 g mL^{-1}), **2** (118 mg, 0.5 mmol) and **S3** (147 mg, 0.75 mmol, 1.5 equiv). Reaction time: 16 h. *E*:*Z* ratio was determined via ^{1}H NMR of the crude to be 2:1. Purification via flash column chromatography on silica gel (Hexane:Ethyl Acetate 98:2 → 95:5) afforded the two diastereomers (114 mg, 57% combined yield).

<u>*Major (E)*</u>: white solid, m.p.: 89-92 °C. ^{1}H NMR (400 MHz, $CDCl_3$) δ 7.91 – 7.85 (m, 1H), 6.85 – 6.70 (m, 6H), 6.47 (t, *J* = 5 Hz, 1H), 4.31 (q, *J* = 7 Hz, 2H), 3.84 (s, 3H), 3.68 (s, 6H), 3.28 (dd, J_1 = 5 Hz, J_2 = 1 Hz, 2H), 1.37 (t, *J* = 7 Hz, 3H). ^{13}C NMR (101 MHz, $CDCl_3$) δ 167.7, 153.2, 147.5, 143.4, 138.8, 130.4, 125.1, 121.8, 110.3, 108.7, 106.8, 61.3, 61.0, 56.1, 33.7, 14.4. HRMS (FI+) (m/z): [M]$^+$ calcd. for $C_{22}H_{24}O_7$, 400.1522; found: 400.1516. NOESY experiments is reported below.

<u>*Minor (Z)*</u>: colorless oil. ^{1}H NMR (400 MHz, $CDCl_3$) δ 6.85 – 6.75 (m, 4H), 6.81 (s,1H), 6.55 – 6.51 (m,

2H), 6.34 (t, J = 5 Hz, 1H), 4.14 (q, J = 7 Hz, 2H), 3.84 (s, 3H), 3.83 (s, 6H), 3.03 (dd, J_1 = 5 Hz, J_2 = 1 Hz, 2H), 1.13 (t, J = 7 Hz, 3H). ^{13}C NMR (101 MHz, $CDCl_3$) δ 168.8, 153.0, 147.5, 138.7, 138.3, 131.4, 126.6, 121.6, 109.8, 108.7, 105.9, 61.1, 61.0, 56.2, 40.8, 14.0. HRMS (FI+) (m/z): $[M]^+$ calcd. for $C_{22}H_{24}O_7$, 400.1522; found: 400.1521. NOESY experiments is reported below.

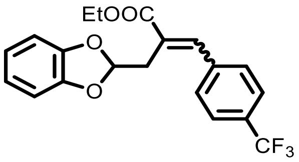

ethyl 2-(benzo[d][1,3]dioxol-2-ylmethyl)-3-(4-(trifluoromethyl)phenyl)acrylate (26). Prepared according to GP4 from **1a** (288 µL, 2.5 mmol, 5.0 equiv, ρ = 1.06 g mL^{-1}), **2** (118 mg, 0.5 mmol) and **S4** (102 µL, 0.75 mmol, 1.5 equiv, ρ = 1.275 g mL^{-1}). Reaction time: 3 h. *E*:*Z* ratio was determined via ^{1}H NMR of the crude to be ca. 1:1. Purified via flash column chromatography on silica gel (Hexane:Ethyl Acetate 98:2 → 90:10) to afford the two diastereomers (119 mg, 63% combined yield).

Isomer 1 (E): colorless oil. ^{1}H NMR (400 MHz, $CDCl_3$) δ 7.93 (s, 1H), 7.62 – 7.52 (m, 4H), 6.86 – 6.79 (m, 2H), 6.79 – 6.72 (m, 2H), 6.44 (t, J = 5 Hz, 1H), 4.33 (q, J = 7 Hz, 2H), 3.13 (dd, J_1 = 5 Hz, J_2 = 1 Hz, 2H), 1.38 (t, J = 7 Hz, 3H). ^{13}C NMR (101 MHz, $CDCl_3$) δ 167.3, 147.2, 141.7, 138.8, 130.6 (q, J = 32 Hz), 129.5, 128.0, 125.6 (q, J = 4 Hz), 121.8, 109.8, 108.7, 61.6, 33.5, 14.4. ^{19}F NMR (376 MHz, $CDCl_3$) δ −62.7. HRMS (FI+) (m/z): $[M]^+$ calcd. for $C_{20}H_{17}F_3O_4$, 378.1079; found: 378.1072.

Isomer 2 (Z): colorless oil. ^{1}H NMR (400 MHz, $CDCl_3$) 7.56 (d, J = 8 Hz, 2H), 7.38 – 7.31 (m, 2H), 6.95 (s, 1H), 6.86 – 6.73 (m, 4H), 6.37 (t, J = 5 Hz, 1H), 4.10 (q, J = 7 Hz, 2H), 3.07 (dd, J_1 = 5 Hz, J_2 = 1 Hz, 2H), 1.06 (t, J = 7 Hz, 3H). ^{13}C NMR (101 MHz, $CDCl_3$) δ 167.9, 147.5, 139.8, 138.2, 130.0 (q, J = 32 Hz), 129.0, 128.7, 125.1 (q, J = 4 Hz), 121.7, 109.5, 108.7, 61.2, 40.6, 13.8. ^{19}F NMR (376 MHz, $CDCl_3$) δ −62.7. HRMS (FI+) (m/z): $[M]^+$ calcd. for $C_{20}H_{17}F_3O_4$, 378.1079; found: 378.1091.

The reaction was also performed using GP5 (HWE: 40 °C, 30 min) to afford **26** in 60% yield (*E:Z* 1:1).

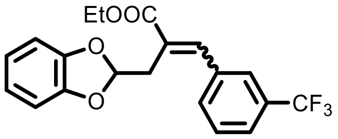

ethyl 2-(benzo[d][1,3]dioxol-2-ylmethyl)-3-(3-(trifluoromethyl)phenyl)acrylate (27). Prepared according to GP4 from **1a** (288 µL, 2.5 mmol, 5.0 equiv, ρ = 1.06 g mL^{-1}), **2** (118 mg, 0.5 mmol) and **S5** (100 µL, 0.75 mmol, 1.5 equiv, ρ = 1.301 g mL^{-1}). Reaction time: 3 h. *E*:*Z* ratio was determined via ^{1}H NMR of the crude to be 1:1. Purified via flash column chromatography on silica gel (Hexane:Ethyl Acetate 98:2 → 95:5) to afford the two diastereomers (98 mg, 52% combined yield).

Isomer 1 (E): colorless oil. ^{1}H NMR (400 MHz, $CDCl_3$) δ 7.93 (s, 1H), 7.74 (dq, J_1 = 2 Hz, J_2 = 1 Hz, 1H), 7.63 (d, J = 8 Hz, 1H), 7.59 – 7.52 (m, 1H), 7.45 (t, J = 8 Hz, 1H), 6.85 – 6.69 (m, 4H), 6.44 (t, J = 5 Hz, 1H), 4.33 (q, J = 7 Hz, 2H), 3.13 (dd, J_1 = 5 Hz, J_2 = 1 Hz, 2H), 1.37 (t, J = 7 Hz, 3H). ^{13}C NMR (101 MHz, $CDCl_3$) δ 167.2, 147.1, 141.6, 135.8, 132.1, 131.0 (q, J = 32 Hz), 129.0, 127.7, 125.9 (q, J = 4 Hz), 125.2 (q, J = 4 Hz), 123.8 (q, J = 272 Hz), 121.6, 109.7, 108.6, 61.4, 33.4, 14.3. ^{19}F NMR (376 MHz, $CDCl_3$) δ −62.8. HRMS (FI+) (m/z): $[M]^+$ calcd. for $C_{20}H_{17}F_3O_4$, 378.1079; found: 378.1072.

Isomer 2 (Z): colorless oil. ^{1}H NMR (400 MHz, $CDCl_3$) δ 7.57 – 7.48 (m, 2H), 7.43 (dd, J_1 = 5 Hz, J_2 = 1

Hz, 2H), 6.95 (s, 1H), 6.85 – 6.77 (m, 4H), 6.37 (t, *J* = 5 Hz, 1H), 4.10 (q, *J* = 7 Hz, 2H), 3.07 (dd, J_1 = 5 Hz, J_2 = 1 Hz, 2H), 1.06 (t, *J* = 7 Hz, 3H). ^{13}C NMR (101 MHz, $CDCl_3$) δ 167.8, 147.3, 137.8, 136.7, 131.6 (q, *J* = 1 Hz), 130.5 (q, *J* = 32 Hz), 128.6, 128.5, 125.2 (q, *J* = 4 Hz), 124.6 (q, *J* = 4 Hz), 124.0 (q, *J* = 272 Hz), 121.6, 109.4, 108.6, 61.1, 40.4, 13.6. ^{19}F NMR (376 MHz, $CDCl_3$) δ −62.7. HRMS (FI+) (m/z): $[M]^+$ calcd. for $C_{20}H_{17}F_3O_4$, 378.1079; found: 378.1068.
The reaction was also performed using GP5 (HWE: 40 °C, 30 min) to afford **27** in 54% yield (*E:Z* 1:1).

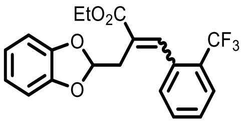

ethyl 2-(benzo[d][1,3]dioxol-2-ylmethyl)-3-(2-(trifluoromethyl)phenyl)acrylate (28). Prepared according to GP4 from **1a** (288 µL, 2.5 mmol, 5.0 equiv, ρ = 1.06 g mL^{-1}), **2** (118 mg, 0.5 mmol) and **S6** (99 µL, 0.75 mmol, 1.5 equiv, ρ = 1.32 g mL^{-1}). Reaction time: 3 h. *E*:*Z* ratio was determined via ^{1}H NMR of the crude to be 5:1. Purified via flash column chromatography on silica gel (Hexane:Ethyl Acetate 98:2 → 95:5) to afford the afford the two diastereomers (130 mg, 69% combined yield).
Major (E): colorless oil. ^{1}H NMR (400 MHz, $CDCl_3$) δ 8.10 (q, *J* = 2 Hz, 1H), 7.68 (dd, *J* = 7.5, 1.7 Hz, 1H), 7.54 – 7.33 (m, 3H), 6.84 – 6.68 (m, 4H), 6.44 (t, *J* = 5 Hz, 1H), 4.34 (q, *J* = 7 Hz, 2H), 2.94 (dd, J_1 = 5, J_2 = 1 Hz, 2H), 1.38 (t, *J* = 7.1 Hz, 3H). ^{13}C NMR (101 MHz, $CDCl_3$) δ 166.9, 147.2, 140.3, 133.9 (q, *J* = 2 Hz), 131.8, 130.5, 128.8, 128.7 (q, *J* = 30 Hz), 128.4, 126.1 (q, *J* = 5 Hz), 124.0 (q, *J* = 274 Hz), 121.6, 109.6, 108.6, 61.5, 33.8, 14.3. ^{19}F NMR (376 MHz, $CDCl_3$) δ -60.6. HRMS (FI+) (m/z): $[M]^+$ calcd. for $C_{20}H_{17}F_3O_4$, 378.1079; found: 378.1086. NOESY experiment is reported below.
The reaction was also performed using GP5 to afford **28** in 72% yield (*E:Z* 1:1).

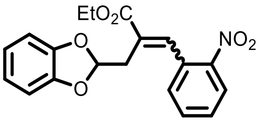

ethyl 2-(benzo[d][1,3]dioxol-2-ylmethyl)-3-(2-nitrophenyl)acrylate (29). Prepared according to GP4 from **1a** (288 µL, 2.5 mmol, 5.0 equiv, ρ = 1.06 g mL^{-1}), **2** (118 mg, 0.5 mmol) and **S7** (113 mg, 0.75 mmol, 1.5 equiv). Reaction time: 3 h. *E*:*Z* ratio was determined via ^{1}H NMR of the crude to be 5:1. Purified via flash column chromatography on silica gel (Hexane:MTBE 85:15) to afford the two diastereomers (113 mg, 64% combined yield).
Major (E): colorless oil. ^{1}H NMR (400 MHz, $CDCl_3$) δ 8.23 (d, *J* = 1 Hz, 1H), 8.16 (dd, J_1 = 8 Hz, J_2 = 1 Hz, 1H), 7.55 (td, J_1 = 8 Hz, J_2 = 1 Hz, 1H), 7.50 – 7.39 (m, 2H), 6.79 – 6.64 (m, 4H), 6.32 (t, *J* = 5 Hz, 1H), 4.34 (q, *J* = 7 Hz, 2H), 2.86 (dd, J_1 = 5 Hz, J_2 = 1 Hz, 2H), 1.38 (t, *J* = 7 Hz, 3H). ^{13}C NMR (101 MHz, $CDCl_3$) δ 166.8, 147.7, 147.1, 141.2, 133.7, 131.7, 130.9, 129.3, 126.9, 124.9, 121.6, 109.4, 108.6, 61.6, 33.5, 14.4. HRMS (FI+) (m/z): $[M]^+$ calcd. for $C_{19}H_{17}NO_6$, 355.1056; found: 355.1064. NOESY experiment is reported below.
The reaction was also performed using GP5 (HWE: 40 °C, 30 min) to afford **29** in 76% yield (*E:Z* 5:1).

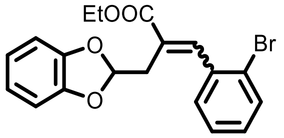

ethyl 2-(benzo[d][1,3]dioxol-2-ylmethyl)-3-(2-cyanophenyl)acrylate (30). Prepared according to GP4 from **1a** (288 µL, 2.5 mmol, 5.0 equiv, ρ = 1.06 g mL^{-1}), **2** (118 mg, 0.5 mmol) and **S8** (87 µL, 0.75 mmol, 1.5 equiv, ρ = 1.585 g mL^{-1}). Reaction time: 3 h. *E*:*Z* ratio was determined via ^{1}H NMR of the crude to be 5:1. Purified via flash column chromatography on silica gel (Hexane:Ethyl Acetate 95:5) to afford the desired product (140 mg, 72% combined yield).

Major (E): waxy solid. ^{1}H NMR (400 MHz, $CDCl_3$) δ 7.91 (s, 1H), 7.59 (dd, J_1 = 8 Hz, J_2 = 1 Hz, 1H), 7.45 (dd, J_1 = 8 Hz, J_2 = 2 Hz, 1H), 7.21 (td, J_1 = 7 Hz, J_2 = 1 Hz, 1H), 7.15 (td, J_1 = 8 Hz, J_2 = 2 Hz, 1H), 6.83 – 6.70 (m, 4H), 6.43 (t, J = 5 Hz, 1H), 4.34 (q, J = 7 Hz, 2H), 3.01 (d, J = 5 Hz, 2H), 1.38 (t, J = 7 Hz, 3H). ^{13}C NMR (101 MHz, $CDCl_3$) δ 167.2, 147.2, 142.8, 135.7, 132.8, 130.5, 130.0, 127.5, 127.4, 124.0, 121.6, 109.8, 108.7, 61.5, 33.5, 14.4. HRMS (FI+) (m/z): [M]$^{+}$ calcd. for $C_{19}H_{17}BrO_4$, 388.0310; found: 388.0310. NOESY experiment is reported below.

Minor (Z): colorless oil. ^{1}H NMR (400 MHz, $CDCl_3$) δ 7.55 (dd, J_1 = 8 Hz, J_2 = 1 Hz, 1H), 7.25 – 7.11 (m, 3H), 7.00 (s, 1H), 6.84 – 6.71 (m, 4H), 6.42 (t, J = 5 Hz, 1H), 4.03 (q, J = 7 Hz, 2H), 3.08 (dd, J_1 = 5 Hz, J_2 = 1 Hz, 2H), 0.98 (t, J = 7 Hz, 3H). ^{13}C NMR (101 MHz, $CDCl_3$) δ 167.5, 147.5, 140.2, 137.3, 132.3, 130.1, 129.4, 128.2, 126.8, 122.9, 121.6, 109.8, 108.7, 60.9, 39.9, 13.7. HRMS (FI+) (m/z): [M]$^{+}$ calcd. for $C_{19}H_{17}BrO_4$, 388.0310; found: 388.0310. NOESY experiment is reported below.

The reaction was also performed using GP5 (HWE: 40 °C, 30 min) to afford **30** in 73% yield (*E:Z* 5:1).

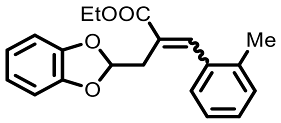

ethyl 2-(benzo[d][1,3]dioxol-2-ylmethyl)-3-(o-tolyl)acrylate (31). Prepared according to GP4 from **1a** (288 µL, 2.5 mmol, 5.0 equiv, ρ = 1.06 g mL^{-1}), **2** (118 mg, 0.5 mmol) and **S9** (88 µL, 0.75 mmol, 1.5 equiv, ρ = 1.019 g mL^{-1}). Reaction time: 16 h. *E*:*Z* ratio was determined via ^{1}H NMR of the crude to be 7:1. Purified via flash column chromatography on silica gel (Hexane:Ethyl Acetate 95:5) to afford the desired product (94 mg, 58% yield).

Major (E): colorless oil. ^{1}H NMR (400 MHz, $CDCl_3$) δ 7.99 (s, 1H), 7.31 (dd, J_1 = 7 Hz, J_2 = 1 Hz, 1H), 7.21 – 7.18 (m, 2H), 7.10 (dt, J_1 = 8 Hz, J_2 = 4 Hz, 1H), 6.81 – 6.75 (m, 2H), 6.72 (dt, J_1 = 5 Hz, J_2 = 4 Hz, 2H), 6.40 (t, J = 5 Hz, 1H), 4.33 (q, J = 7 Hz, 2H), 3.03 (dd, J_1 = 5 Hz, J_2 = 1 Hz, 2H), 2.29 (s, 3H), 1.38 (t, J = 7 Hz, 3H). ^{13}C NMR (101 MHz, $CDCl_3$) δ 167.6, 147.3, 143.3, 136.7, 134.6, 130.1, 128.6, 128.5, 126.7, 125.9, 121.5, 109.9, 108.6, 61.3, 33.3, 20.0, 14.4. HRMS (FI+) (m/z): [M]$^{+}$ calcd. for $C_{20}H_{20}O_4$, 324.1362; found: 324.1351. NOESY experiment is reported below.

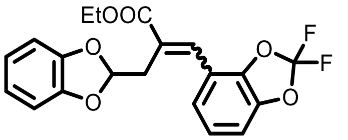

ethyl 2-(benzo[d][1,3]dioxol-2-ylmethyl)-3-(2,2-difluorobenzo[d][1,3]dioxol-4-yl)acrylate (32). Prepared according to GP4 from **1a** (288 µL, 2.5 mmol, 5.0 equiv, ρ = 1.06 g mL^{-1}), **2** (118 mg, 0.5 mmol)

and **S10** (118 mg, 0.75 mmol, 1.5 equiv, ρ = 1.423 g mL^{-1}). Reaction time: 3 h. *E*:*Z* ratio was determined via ^{1}H NMR of the crude to be 3:1. Purified via flash column chromatography on silica gel (Hexane:Ethyl Acetate 95:5) to afford the desired product (105 mg, 54% yield).

Major (E): white solid. m.p.: 62-64 °C. ^{1}H NMR (400 MHz, $CDCl_3$) δ 7.81 (d, *J* = 1 Hz, 1H), 7.24 – 7.19 (m, 1H), 7.02 (s, 1H), 7.00 (d, *J* = 1 Hz, 1H), 6.83 – 6.75 (m, 2H), 6.75 – 6.67 (m, 2H), 6.42 (t, *J* = 5 Hz, 1H), 4.33 (q, *J* = 7 Hz, 2H), 3.17 (dd, J_1 = 5 Hz, J_2 = 1 Hz, 2H), 1.38 (t, *J* = 7 Hz, 3H). ^{13}C NMR (101 MHz, $CDCl_3$) δ 166.9, 147.2, 143.8, 141.7, 134.2, 131.5 (t, *J* = 256 Hz), 129.5, 124.1, 123.7, 121.7, 118.6, 109.8, 109.7, 108.7, 61.7, 33.9, 14.4. ^{19}F NMR (376 MHz, $CDCl_3$) δ -49.7. HRMS (FI+) (m/z): $[M]^+$ calcd. for $C_{20}H_{16}F_2O_6$, 390.0915; found: 390.0922. NOESY experiment is reported below.

Minor (Z): colorless oil. ^{1}H NMR (400 MHz, $CDCl_3$) δ 7.06 – 6.93 (m, 3H), 6.85 – 6.75 (m, 5H), 6.38 (t, *J* = 5 Hz, 1H), 4.15 (q, *J* = 7 Hz, 2H), 3.09 (dd, J_1 = 5 Hz, J_2 = 1 Hz, 2H), 1.11 (t, *J* = 7 Hz, 3H). ^{13}C NMR (101 MHz, $CDCl_3$) δ 167.3, 147.4, 143.6, 141.4, 131.5 (t, *J* = 255 Hz), 131.3, 130.5, 124.0, 123.2, 121.7, 119.5, 109.5, 109.2, 108.7, 61.3, 40.5, 13.8. ^{19}F NMR (376 MHz, $CDCl_3$) δ -49.7. HRMS (FI+) (m/z): $[M]^+$ calcd. for $C_{20}H_{16}F_2O_6$, 390.0915; found: 390.0920. NOESY experiment is reported below.

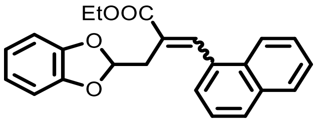

ethyl 2-(benzo[d][1,3]dioxol-2-ylmethyl)-3-(naphthalen-1-yl)acrylate (33). Prepared according to GP4 from **1a** (288 µL, 2.5 mmol, 5.0 equiv, ρ = 1.06 g mL^{-1}), **2** (118 mg, 0.5 mmol) and **S11** (102 µL, 0.75 mmol, 1.5 equiv, ρ = 1.15 g mL^{-1}). Reaction time: 3 h. *E*:*Z* ratio was determined via ^{1}H NMR of the crude to be 7:1. Purified via flash column chromatography on silica gel (Hexane:Ethyl Acetate 95:5) to afford the desired product (104 mg, 58% yield).

Major (E): ^{1}H NMR (400 MHz, $CDCl_3$) δ 8.47 – 8.40 (m, 1H), 7.98 – 7.83 (m, 2H), 7.80 (dd, J_1 = 8 Hz, J_2 = 1 Hz, 1H), 7.59 – 7.47 (m, 3H), 7.39 (dd, J_1 = 8 Hz, J_2 = 7 Hz, 1H), 6.79 – 6.67 (m, 2H), 6.65 – 6.53 (m, 2H), 6.42 (t, *J* = 5 Hz, 1H), 4.39 (q, *J* = 7 Hz, 2H), 3.07 (dd, J_1 = 5 Hz, J_2 = 1 Hz, 2H), 1.42 (t, *J* = 7 Hz, 3H). ^{13}C NMR (101 MHz, $CDCl_3$) δ 167.4, 147.2, 142.4, 133.5, 132.6, 131.5, 128.9, 128.6, 128.3, 126.5, 126.4, 126.3, 125.4, 124.9, 121.5, 109.8, 108.5, 61.4, 33.7, 14.5. HRMS (FI+) (m/z): $[M]^+$ calcd. for $C_{23}H_{20}O_4$, 360.1362; found: 360.1347. NOESY experiment is reported below.

The reaction was also performed using GP5 (HWE: 60 °C, 60 min, 2.8 bar) to afford **33** in 56% yield (*E:Z* 7:1).

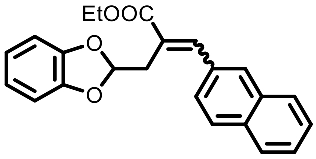

ethyl 2-(benzo[d][1,3]dioxol-2-ylmethyl)-3-(naphthalen-2-yl)acrylate (34). Prepared according to GP4 from **1a** (288 µL, 2.5 mmol, 5.0 equiv, ρ = 1.06 g mL^{-1}), **2** (118 mg, 0.5 mmol) and **S12** (117 mg, 0.75 mmol, 1.5 equiv). Reaction time: 3 h. *E*:*Z* ratio was determined via ^{1}H NMR of the crude to be 2:1. Purified via flash column chromatography on silica gel (Hexane:Ethyl Acetate 95:5) to afford the two diastereomers (103 mg, 57% combined yield).

<u>Major (E)</u>: colorless liquid. ^{1}H NMR (400 MHz, $CDCl_3$) δ 8.10 (s, 1H), 7.98 (s, 1H), 7.85 – 7.76 (m, 2H), 7.70 – 7.61 (m, 1H), 7.54 (dd, J_1 = 8 Hz, J_2 = 2 Hz, 1H), 7.51 – 7.43 (m, 2H), 6.89 – 6.76 (m, 4H), 6.55 (t, J = 5 Hz, 1H), 4.35 (q, J = 7 Hz, 2H), 3.27 (dd, J_1 = 5 Hz, J_2 = 1 Hz, 2H), 1.40 (t, J = 7 Hz, 3H). ^{13}C NMR (101 MHz, $CDCl_3$) δ 167.8, 147.4, 143.5, 133.3, 133.2, 132.6, 129.2, 128.6, 128.3, 127.7, 127.0, 126.8, 126.6, 126.1, 121.7, 110.3, 108.8, 61.4, 33.8, 14.5. HRMS (FI+) (m/z): $[M]^+$ calcd. for $C_{23}H_{20}O_4$, 360.1362; found: 360.1377. NOESY, COSY experiments are reported below.
<u>Minor (Z)</u>: colorless liquid. ^{1}H NMR (400 MHz, $CDCl_3$) δ 7.85 – 7.73 (m, 4H), 7.53 – 7.43 (m, 2H), 7.39 (dd, J_1 = 8 Hz, J_2 = 2 Hz, 1H), 7.07 (d, J = 1 Hz, 1H), 6.81 (d, J = 1 Hz, 4H), 6.41 (t, J = 5 Hz, 1H), 4.14 (q, J = 7 Hz, 2H), 3.11 (dd, J_1 = 5 Hz, J_2 = 1 Hz, 2H), 1.05 (t, J = 7 Hz, 3H). ^{13}C NMR (101 MHz, $CDCl_3$) δ 168.8, 147.5, 139.4, 133.4, 133.1, 133.1, 128.3, 128.2, 127.7, 127.6, 127.1, 126.5, 126.4, 126.3, 121.7, 109.8, 108.7, 61.0, 40.9, 13.9. HRMS (FI+) (m/z): $[M]^+$ calcd. for $C_{23}H_{20}O_4$, 360.1362; found: 360.1372. NOESY, HSQC experiments are reported below.

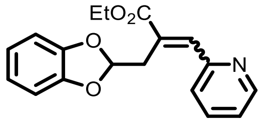

ethyl 2-(benzo[d][1,3]dioxol-2-ylmethyl)-3-(pyridin-2-yl)acrylate (35). Prepared according to GP4 from **1a** (288 μL, 2.5 mmol, 5.0 equiv, ρ = 1.06 g mL^{-1}), **2** (118 mg, 0.5 mmol) and **S13** (71 μL, 0.75 mmol, 1.5 equiv, ρ = 1.126 g mL^{-1}). Reaction time: 3 h. *N.B.: the reaction was quenched with sat'd NH_4Cl solution and then neutralized with sat'd $NaHCO_3$ solution before the extraction*. *E*:*Z* ratio was determined via ^{1}H NMR of the crude to be 3:1. Purified via flash column chromatography on silica gel (Hexane:Ethyl Acetate 90:10 → 80:20) to afford the two diastereomers (113 mg, 72% yield).
<u>Major (E)</u>: colorless oil. ^{1}H NMR (400 MHz, $CDCl_3$) δ 8.59 (ddd, J_1 = 5 Hz, J_2 = 2 Hz, J_3 = 1 Hz, 1H), 7.78 (s, 1H), 7.68 (td, J_1 = 8 Hz, J_2 = 2 Hz, 1H), 7.40 (dt, J_1 = 8 Hz, J_2 = 1 Hz, 1H), 7.18 (ddd, J_1 = 8 Hz, J_2 = 5 Hz, J_3 = 1 Hz, 1H), 6.82 – 6.66 (m, 4H), 6.53 (t, J = 5 Hz, 1H), 4.30 (q, J = 7 Hz, 2H), 3.71 (dd, J_1 = 5 Hz, J_2 = 1 Hz, 2H), 1.33 (t, J = 7 Hz, 3H). ^{13}C NMR (101 MHz, $CDCl_3$) δ 168.0, 154.4, 149.7, 147.5, 139.3, 136.5, 129.6, 126.6, 123.1, 121.3, 110.9, 108.6, 61.4, 32.8, 14.4. HRMS (FI+) (m/z): $[M]^+$ calcd. for $C_{18}H_{17}NO_4$, 311.1158; found: 311.1153.
<u>Minor (Z)</u>: colorless oil. ^{1}H NMR (400 MHz, $CDCl_3$) δ 8.53 (ddd, J_1 = 5 Hz, J_2 = 2 Hz, J_3 = 1 Hz, 1H), 7.63 (td, J_1 = 8 Hz, J_2 = 2 Hz, 1H), 7.23 (dt, J_1 = 8 Hz, J_2 = 1 Hz, 1H), 7.15 (ddd, J_1 = 8 Hz, J_2 = 5 Hz, J_3 = 1 Hz, 1H), 6.79 (d, J = 1 Hz, 4H), 6.77 (d, J = 1 Hz, 1H), 6.37 (t, J = 5 Hz, 1H), 4.21 (q, J = 7 Hz, 2H), 3.06 (dd, J_1 = 5 Hz, J_2 = 1 Hz, 2H), 1.17 (t, J = 7 Hz, 3H). ^{13}C NMR (101 MHz, $CDCl_3$) δ 169.4, 153.7, 149.2, 147.4, 136.3, 134.7, 130.9, 123.7, 122.7, 121.7, 109.4, 108.8, 61.1, 40.5, 14.0. HRMS (FI+) (m/z): $[M]^+$ calcd. for $C_{18}H_{17}NO_4$, 311.1158; found: 311.1147.
The reaction was also performed using GP5 (HWE: 40 °C, 30 min) to afford **35** in 77% yield (*E:Z* 3:1).

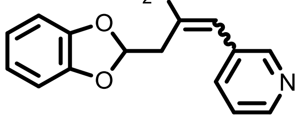

ethyl 2-(benzo[d][1,3]dioxol-2-ylmethyl)-3-(pyridin-3-yl)acrylate (36). Prepared according to GP5 from **1a** (288 μL, 2.5 mmol, 5.0 equiv, ρ = 1.06 g mL^{-1}), **2** (118 mg, 0.5 mmol). and **S14**. Residence time: 30 min (40 °C). *E*:*Z* ratio was determined via ^{1}H NMR of the crude to be 1:1. Purified via flash column chromatography on silica gel (Hexane:Ethyl Acetate 4:1) to afford the two diastereomers (103 mg, 66%

yield).

Isomer 1 (E): colorless oil. ^{1}H NMR (400 MHz, $CDCl_3$) δ 8.62 (s, 1H), 8.50 – 8.44 (m, 1H), 7.80 (s, 1H), 7.78 – 7.72 (m, 1H), 7.24 – 7.16 (m, 1H), 6.78 – 6.63 (m, 4H), 6.36 (t, *J* = 5 Hz, 1H), 4.25 (q, *J* = 7 Hz, 2H), 3.08 (d, *J* = 5 Hz, 2H), 1.30 (t, *J* = 7 Hz, 3H). ^{13}C NMR (101 MHz, $CDCl_3$) δ 167.1, 150.1, 149.5, 147.2, 139.4, 136.3, 131.2, 128.3, 123.5, 121.8, 109.7, 108.7, 61.6, 33.4, 14.4. HRMS (FD+) (m/z): $[M]^+$ calcd. for $C_{18}H_{17}NO_4$, 311.1158; found: 311.1149.

Isomer 2 (Z): colorless oil. ^{1}H NMR (400 MHz, $CDCl_3$) δ 8.53 – 8.45 (m, 2H), 7.61 (dt, J_1 = 8 Hz, J_2 = 2 Hz, 1H), 7.29 – 7.21 (m, 1H), 6.90 (s, 1H), 6.85 – 6.72 (m, 4H), 6.36 (t, *J* = 5 Hz, 1H), 4.12 (q, *J* = 7 Hz, 2H), 3.08 (dd, J_1 = 5 Hz, J_2 = 1 Hz, 2H), 1.08 (t, *J* = 7 Hz, 3H). ^{13}C NMR (101 MHz, $CDCl_3$) δ 167.6, 149.3, 148.7, 147.4, 136.3, 135.8, 132.0, 129.2, 123.0, 121.7, 109.5, 108.7, 61.2, 40.6, 13.9 HRMS (FD+) (m/z): $[M]^+$ calcd. for $C_{18}H_{17}NO_4$, 311.1158; found: 311.1144

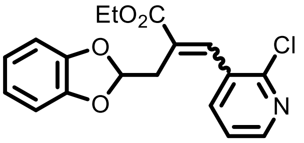

ethyl 2-(benzo[d][1,3]dioxol-2-ylmethyl)-3-(2-chloropyridin-3-yl)acrylate (37). Prepared according to GP5 from **1a** (288 μL, 2.5 mmol, 5.0 equiv, ρ = 1.06 g mL^{-1}), **2** (118 mg, 0.5 mmol) and **S15.** Residence time: 30 min (40 °C). *E*:*Z* ratio was determined via ^{1}H NMR of the crude to be 3:1. Purified via flash column chromatography on silica gel (Hexane:Ethyl Acetate 90:10 → 80:20) to afford the two diastereomers (104 mg, 60% yield).

Major (E): white solid. ^{1}H NMR (400 MHz, $CDCl_3$) δ 8.32 (dd, J_1 = 5 Hz, J_2 =2 Hz, 1H), 7.88 (s, 1H), 7.84 (dd, J_1 = 8, J_2 = 2 Hz, 1H), 7.16 (dd, J_1 = 8, J_2 = 5 Hz, 1H), 6.83 – 6.69 (m, 4H), 6.42 (t, *J* = 5 Hz, 1H), 4.34 (q, *J* = 7 Hz, 2H), 2.98 (d, *J* = 5 Hz, 2H), 1.38 (t, *J* = 7 Hz, 3H). ^{13}C NMR (101 MHz, $CDCl_3$) δ 166.7, 150.7, 149.5, 147.0, 138.9, 138.4, 130.5, 129.2, 122.4, 121.8, 109.4, 108.7, 61.7, 33.5, 14.4. HRMS (FD+) (m/z): $[M]^+$ calcd. for $C_{18}H_{16}ClNO_4$, 345.0768; found: 345.0774

Minor (Z): colorless oil. ^{1}H NMR (400 MHz, $CDCl_3$) δ 8.32 (dd, J_1 = 5 Hz, J_2 = 2 Hz, 1H), 7.54 (dd, J_1 = 8 Hz, J_2 = 2 Hz, 1H), 7.18 (dd, J_1 = 8 Hz, J_2 = 5 Hz, 1H), 6.99 (s, 1H), 6.86 – 6.74 (m, 4H), 6.41 (t, *J* = 5 Hz, 1H), 4.06 (q, *J* = 7 Hz, 2H), 3.11 (dd, J_1 =5 Hz, J_2 = 1.0 Hz, 2H), 1.03 (t, *J* = 7 Hz, 3H). ^{13}C NMR (101 MHz, $CDCl_3$) δ 166.7, 149.7, 149.0, 147.4, 138.9, 136.4, 131.8, 129.8, 121.8, 121.7, 109.5, 108.7, 61.2, 40.0, 13.9. HRMS (FD+) (m/z): $[M]^+$ calcd. for $C_{18}H_{16}ClNO_4$, 345.0768; found: 345.0753.

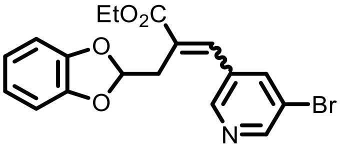

ethyl 2-(benzo[d][1,3]dioxol-2-ylmethyl)-3-(5-bromopyridin-3-yl)acrylate (38). Prepared according to GP5 from **1a** (288 μL, 2.5 mmol, 5.0 equiv, ρ = 1.06 g mL^{-1}), **2** (118 mg, 0.5 mmol) and **S16.** Residence time: 30 min (40 °C). *E*:*Z* ratio was determined via ^{1}H NMR of the crude to be 1:1. Purified via flash column chromatography on silica gel (Hexane:Ethyl Acetate 90:10 → 80:20) to afford the two diastereomers (125 mg, 64% yield).

Isomer 1 (E): colorless oil. ^{1}H NMR (400 MHz, $CDCl_3$) δ 8.58 (d, J = 10 Hz, 2H), 7.97 (t, J = 2 Hz, 1H), 7.80 (s, 1H), 6.87 – 6.73 (m, 4H), 6.44 (t, J = 5Hz, 1H), 4.33 (q, J = 7 Hz, 2H), 3.10 (d, J = 5 Hz, 2H), 1.37 (t, J = 7 Hz, 3H). ^{13}C NMR (101 MHz, $CDCl_3$) δ 166.8, 150.6, 148.1, 147.0, 138.6, 138.0, 132.6, 129.5, 121.9, 120.7, 109.6, 108.8, 61.8, 33.7, 14.4. HRMS (FD+) (m/z): $[M]^+$ calcd. for $C_{18}H_{16}BrNO_4$, 389.0262; found: 389.0278.

Isomer 2 (Z):colorless oil. ^{1}H NMR (400 MHz, $CDCl_3$) δ 8.57 (s, 1H), 8.45 – 8.30 (m, 1H), 7.73 (t, J = 2 Hz, 1H), 6.84 (s, 1H), 6.83 – 6.76 (m, 4H), 6.36 (t, J = 5 Hz, 1H), 4.13 (q, J = 7 Hz, 2H), 3.08 (dd, J_1 = 5 Hz, J_2 =1 Hz, 2H), 1.11 (t, J = 7 Hz, 3H). ^{13}C NMR (101 MHz, $CDCl_3$) δ 167.1, 149.9, 147.6, 147.3, 138.1, 134.8, 133.5, 130.4, 121.8, 120.1, 109.3, 108.7, 61.4, 40.5, 13.9. HRMS (FD+) (m/z): $[M]^+$ calcd. for $C_{18}H_{16}BrNO_4$, 389.0262; found: 389.0279.

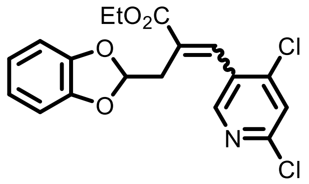

ethyl 2-(benzo[d][1,3]dioxol-2-ylmethyl)-3-(4,6-dichloropyridin-3-yl)acrylate (39). Prepared according to GP6 from **1a** (288 µL, 2.5 mmol, 5.0 equiv, ρ = 1.06 g mL^{-1}), **2** (118 mg, 0.5 mmol) and **S17**. Residence time: 30 min (40 °C). *E*:*Z* ratio was determined via ^{1}H NMR of the crude to be 4:1. Purified via flash column chromatography on silica gel (Hexane:Ethyl Acetate 95:5 → 90:10) to afford the two diastereomers (133 mg, 70% yield).

Major (E): colorless oil. ^{1}H NMR (400 MHz, $CDCl_3$) δ 8.50 (s, 1H), 7.77 (s, 1H), 7.43 (s, 1H), 6.83 – 6.69 (m, 4H), 6.37 (t, J = 5 Hz, 1H), 4.34 (q, J = 7.1 Hz, 2H), 3.04 (d, J = 5 Hz, 2H), 1.37 (t, J = 7 Hz, 3H). ^{13}C NMR (101 MHz, $CDCl_3$) δ 166.4, 151.9, 150.0, 147.0, 145.5, 135.5, 130.4, 129.4, 124.6, 121.9, 109.2, 108.8, 61.9, 33.7, 14.4. HRMS (FD+) (m/z): $[M]^+$ calcd. for $C_{18}H_{15}Cl_2NO_4$, 379.0378; found: 379.0387.

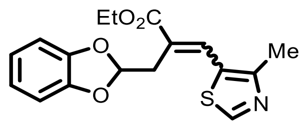

ethyl 2-(benzo[d][1,3]dioxol-2-ylmethyl)-3-(4-methylthiazol-5-yl)acrylate (40). Prepared according to GP4 from **1a** (288 µL, 2.5 mmol, 5.0 equiv, ρ = 1.06 g mL^{-1}), **2** (118 mg, 0.5 mmol) and **S18** (95 mg, 0.75 mmol, 1.5 equiv). Reaction time: 3 h. *N.B.: the reaction was quenched with sat'd NH_4Cl solution and then neutralized with sat'd $NaHCO_3$ solution before the extraction.* *E*:*Z* ratio was determined via ^{1}H NMR of the crude to be 8:1. Purified via flash column chromatography on silica gel (Hexane:Ethyl Acetate 80:20) to afford the major diastereomer as a deliquescent colorless solid (100 mg, 60% yield).

Major (E): colorless oil. ^{1}H NMR (400 MHz, $CDCl_3$) δ 8.70 (s, 1H), 8.06 (d, J = 1 Hz, 1H), 6.84 – 6.76 (m, 2H), 6.76 – 6.68 (m, 2H), 6.39 (t, J = 5 Hz, 1H), 4.31 (q, J = 7 Hz, 2H), 3.28 (d, J = 5 Hz, 2H), 2.60 (s, 3H), 1.35 (t, J = 7 Hz, 3H). ^{13}C NMR (101 MHz, $CDCl_3$) δ 167.3, 157.4, 153.3, 147.2, 132.1, 125.6, 123.6, 121.7, 109.8, 108.7, 61.5, 34.3, 16.2, 14.4. HRMS (FI+) (m/z): $[M]^+$ calcd. for $C_{17}H_{17}NO_4S$, 331.0878; found: 331.0881.

The reaction was also performed using GP5 (HWE: 40 °C, 30 min) to afford **40** in 59% yield (*E:Z* 8:1).

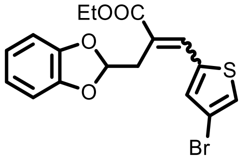

ethyl 2-(benzo[d][1,3]dioxol-2-ylmethyl)-3-(4-bromothiophen-2-yl)acrylate (41). Prepared according to GP4 from **1a** (288 µL, 2.5 mmol, 5.0 equiv, ρ = 1.06 g mL^{-1}), **2** (118 mg, 0.5 mmol) and **S19** (143 mg, 0.75 mmol, 1.5 equiv). Reaction time: 3h. *E*:*Z* ratio was determined via ^{1}H NMR of the reaction crude to be ca. 1:1. Purification via flash column chromatography on silica gel (Hexane:Ethyl Acetate 90:10) afforded the two diastereomers an inseparable mixture as a yellowish oil (130 mg, 66% yield).

^{1}H NMR (400 MHz, $CDCl_3$) δ 7.90 (s, 1H), 7.31 (ddd, J_1 = 11 Hz, J_2 = 1 Hz, J_3 = 1 Hz, 2H), 7.25 – 7.19 (m, 2H), 6.99 – 6.94 (m, 1H), 6.87 – 6.72 (m, 8H), 6.42 (t, *J* = 5 Hz, 1H), 6.32 (t, *J* = 5 Hz, 1H), 4.31 (qd, J_1 = 7 Hz, J_2 = 5 Hz, 4H), 3.31 (d, *J* = 5 Hz, 2H), 3.04 (dd, J_1 = 5 Hz, J_2 = 1 Hz, 2H), 1.35 (q, *J* = 7 Hz, 6H). ^{13}C NMR (101 MHz, $CDCl_3$) δ 167.3, 166.7, 147.4, 147.2, 139.0, 138.6, 135.9, 134.6, 134.5, 133.8, 127.8, 126.3, 123.8, 121.8, 121.7, 121.7, 110.8, 109.8, 109.8, 109.6, 108.8, 108.7, 61.5, 61.3, 40.9, 34.4, 14.4, 14.3.

The reaction was also performed using GP5 (HWE: 40 °C, 30 min) to afford **41** in 76% yield (*E:Z* 5:1).

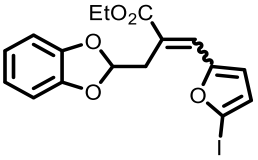

ethyl 2-(benzo[d][1,3]dioxol-2-ylmethyl)-3-(5-iodofuran-2-yl)acrylate (42). Prepared according to GP4 from **1a** (288 µL, 2.5 mmol, 5.0 equiv, ρ = 1.06 g mL^{-1}), **2** (118 mg, 0.5 mmol) and **S20** (166 mg, 0.75 mmol, 1.5 equiv). Reaction time: 3 h. *E*:*Z* ratio was determined via ^{1}H NMR of the crude to be 3:1. Purified via flash column chromatography on silica gel (Hexane:Ethyl Acetate 90:10) to afford the two diastereomers (106 mg, 50% combined yield).

<u>Major (E)</u>: yellowish oil. ^{1}H NMR (400 MHz, $CDCl_3$) δ 7.52 (s, 1H), 6.84 – 6.69 (m, 4H), 6.60 (d, *J* = 4 Hz, 1H), 6.54 (d, *J* = 4 Hz, 1H), 6.39 (t, *J* = 5 Hz, 1H), 4.28 (q, *J* = 7 Hz, 2H), 3.38 (d, *J* = 5 Hz, 2H), 1.33 (t, *J* = 7 Hz, 3H). ^{13}C NMR (101 MHz, $CDCl_3$) δ 167.6, 156.4, 147.4, 127.5, 122.8, 122.2, 121.6, 118.7, 110.2, 108.7, 92.3, 61.4, 33.8, 14.4. HRMS (FI+) (m/z): $[M]^+$ calcd. for $C_{17}H_{15}IO_5$, 425.9964; found: 425.9968. NOESY experiment is reported below.

<u>Minor (Z)</u>: colorless oil. ^{1}H NMR (400 MHz, $CDCl_3$) δ 6.97 (d, *J* = 4 Hz, 1H), 6.84 – 6.76 (m, 4H), 6.65 (s, 1H), 6.60 (d, *J* = 4 Hz, 1H), 6.30 (t, *J* = 5 Hz, 1H), 4.30 (q, *J* = 7 Hz, 2H), 3.01 (dd, J_1 = 5 Hz, J_2 = 1 Hz, 2H), 1.33 (t, *J* = 7 Hz, 3H). ^{13}C NMR (101 MHz, $CDCl_3$) δ 167.2, 155.9, 147.4, 126.8, 122.9, 122.2, 121.7, 117.2, 109.8, 108.8, 90.3, 61.2, 40.8, 14.4. HRMS (FD+) (m/z): $[M]^+$ calcd. for $C_{17}H_{15}IO_5$, 425.9964; found: 425.9956. NOESY experiment is reported below.

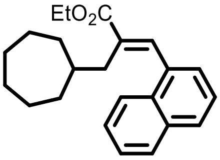

(E)-ethyl 2-(cycloheptylmethyl)-3-(naphthalen-1-yl)acrylate (43). Prepared according to GP4 from **1p** (302 μL, 2.5 mmol, 5.0 equiv, ρ = 0.811 g mL^{-1}), **2** (118 mg, 0.5 mmol) and **S11** (102 μL, 0.75 mmol, 1.5 equiv, ρ = 1.15 g mL^{-1}). Reaction time: 16 h. Only one diastereomer was detected via ^{1}H NMR of the reaction crude. Purification via flash column chromatography on silica gel (Hexane:Ethyl Acetate 98:2) afforded the desired product as a colorless oil (73 mg, 43% yield). ^{1}H NMR (400 MHz, $CDCl_3$) δ 8.12 (s, 1H), 7.95 – 7.79 (m, 3H), 7.54 – 7.44 (m, 3H), 7.36 (dt, J_1 = 7 Hz, J_2 = 1 Hz, 1H), 4.34 (q, J = 7 Hz, 2H), 2.38 – 2.31 (m, 2H), 1.75 – 1.64 (s, 1H), 1.63 – 1.51 (m, 2H), 1.50 – 1.36 (m, 6H), 1.35 – 1.13 (m, 5H), 0.92 (dtd, J_1 = 14 Hz, J_2 = 10 Hz, J_3 = 3 Hz, 2H). ^{13}C NMR (101 MHz, $CDCl_3$) δ 168.6, 138.2, 135.6, 133.8, 133.5, 131.7, 128.55, 128.3, 126.3, 126.2, 126.2, 125.3, 125.1, 60.9, 38.8, 35.4, 34.3, 28.5, 26.1, 14.5. HRMS (FI+) (m/z): $[M]^+$ calcd. for $C_{23}H_{28}O_2$, 336.2089; found: 336.2090. NOESY, COSY, HSQC, HMBC experiments are reported below.

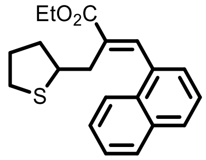

(E)-ethyl 3-(naphthalen-1-yl)-2-((tetrahydrothiophen-2-yl)methyl)acrylate (44). Prepared according to GP4 from **1f** (220 μL, 2.5 mmol, 5.0 equiv, ρ = 1.0 g mL^{-1}), **2** (118 mg, 0.5 mmol) and **S11** (102 μL, 0.75 mmol, 1.5 equiv, ρ = 1.15 g mL^{-1}). Reaction time: 16 h. Only one diastereomer was detected via ^{1}H NMR of the reaction crude. Purification via flash column chromatography on silica gel (Hexane:Ethyl Acetate 98:2 → 95:5) afforded the desired product as a colorless oil (77 mg, 47% yield). ^{1}H NMR (400 MHz, $CDCl_3$) δ 8.20 (t, J = 1 Hz, 1H), 7.96 – 7.78 (m, 3H), 7.59 – 7.42 (m, 4H), 4.35 (q, J = 7 Hz, 2H), 3.67 – 3.52 (m, 1H), 2.82 – 2.63 (m, 4H), 1.93 – 1.78 (m, 1H), 1.78 – 1.56 (m, 2H), 1.40 (t, J = 7 Hz, 4H). ^{13}C NMR (101 MHz, $CDCl_3$) δ 167.9, 139.4, 134.1, 133.5, 133.2, 131.6, 128.6, 128.6, 126.4, 126.4, 126.2, 125.3, 124.9, 61.1, 47.5, 36.5, 34.9, 32.3, 29.6, 14.5. HRMS (FI+) (m/z): $[M]^+$ calcd. for $C_{20}H_{22}O_2S$, 326.1340; found: 326.1339. NOESY experiment is reported below.

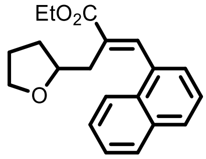

(E)-ethyl 3-(naphthalen-1-yl)-2-((tetrahydrofuran-2-yl)methyl)acrylate (45). Prepared according to GP4 from **1c** (203 μL, 2.5 mmol, 5.0 equiv, ρ = 0.889 g mL^{-1}), **2** (118 mg, 0.5 mmol) and **S11** (102 μL, 0.75 mmol, 1.5 equiv, ρ = 1.15 g mL^{-1}). Reaction time: 16 h. Only one isomer was detected via ^{1}H NMR of the reaction crude. Purification via flash column chromatography on silica gel (Hexane → Hexane:Ethyl Acetate 95:5) afforded the desired product as a colorless oil (107 mg, 68% yield). ^{1}H NMR (400 MHz, $CDCl_3$) δ 8.24 (s, 1H), 7.97 – 7.78 (m, 3H), 7.59 (dt, J_1 = 7 Hz, J_2 = 1 Hz, 1H), 7.55 – 7.43 (m, 3H), 4.35 (q, J = 7 Hz, 2H), 4.17 – 4.06 (m, 1H), 3.71 – 3.56 (m, 2H), 2.70 (dd, J_1 = 13 Hz, J_2 = 8 Hz, 1H), 2.59 (ddd, J_1 = 13 Hz, J_2 = 6 Hz, J_3 = 1 Hz, 1H), 1.88 (ddt, J_1 = 12 Hz, J_2 = 8 Hz, J_3 = 6 Hz, 1H), 1.81 – 1.63 (m, 2H), 1.40 (t, J = 7 Hz, 3H), 1.37 – 1.22 (m, 1H). ^{13}C NMR (101 MHz, $CDCl_3$) δ 168.1, 139.3, 133.5, 133.2, 132.8, 131.6,

128.5 (2x), 126.6, 126.4, 126.1, 125.4, 124.9, 77.9, 67.6, 61.0, 33.9, 31.4, 25.6, 14.4. HRMS (FI+) (m/z): $[M]^+$ calcd. for $C_{20}H_{22}O_3$, 310.1569; found: 310.1582. NOESY experiment is reported below.
The reaction was also performed using GP5 (HWE: 60 °C, 60 min, 2.8 bar) to afford **45** in 60% yield (*E:Z* > 20:1).

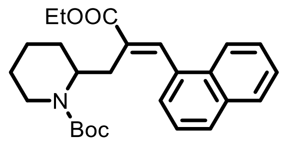

(E)-tert-butyl 2-(2-(ethoxycarbonyl)-3-(naphthalen-1-yl)allyl)piperidine-1-carboxylate (46). Prepared according to GP4 from **1j** (480 μL, 2.5 mmol, 5.0 equiv, ρ = 0.964 g mL^{-1}), **2** (118 mg, 0.5 mmol) and **S11** (102 μL, 0.75 mmol, 1.5 equiv, ρ = 1.15 g mL^{-1}). Reaction time: 16 h. Only one isomer was detected via ^1H NMR of the reaction crude. Purification via flash column chromatography on silica gel (Hexane:Ethyl Acetate 90:10) afforded the desired product as a colorless oil (108 mg, 51% yield). ^1H NMR (400 MHz, $CDCl_3$) δ 8.16 (s, 1H), 7.91 – 7.78 (m, 3H), 7.54 – 7.46 (m, 3H), 7.34 (d, J = 7 Hz, 1H), 4.52 (s, 1H), 4.34 (q, J = 7 Hz, 2H), 3.56 (m, 1H), 2.85 (dd, J_1 = 14 Hz, J_2 = 10 Hz, 1H), 2.38 (dd, J_1 = 14 Hz, J_2 = 4 Hz, 1H), 1.90 (bs, 1H), 1.54 – 1.36 (m, 14H), 1.33 – 0.89 (m, 4H). ^{13}C NMR (101 MHz, $CDCl_3$) δ 167.7, 154.8, 139.5, 133.7, 133.5, 131.3, 128.5, 126.4, 126.2, 126.2, 125.4, 125.1, 79.2, 61.0, 50.3, 38.3, 29.5, 28.5, 28.5, 25.5, 19.1, 14.4. HRMS (FI+) (m/z): $[M]^+$ calcd. for $C_{26}H_{33}NO_4$, 423.2410; found: 423.2400. HSQC experiment is reported below. *The geometry of **42** was assigned in analogy with compounds **39-41**.*

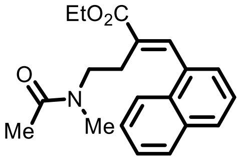

(E)-ethyl 4-(N-methylacetamido)-2-(naphthalen-1-ylmethylene)butanoate (47). Prepared according to GP4 from **1k** (186 μL, 2.0 mmol, 4.0 equiv, ρ = 0.937 g mL^{-1}), **2** (118 mg, 0.5 mmol) and **S11** (102 μL, 0.75 mmol, 1.5 equiv, ρ = 1.15 g mL^{-1}). Reaction time: 16 h. Only one diastereomer was detected via ^1H NMR of the reaction crude. Purification via flash column chromatography on silica gel (Hexane:Ethyl Acetate 60:40) afforded the desired product as a colorless oil (90 mg, 55% yield). ^1H NMR (400 MHz, $CDCl_3$) δ 8.28 (d, J = 1 Hz, 2H), 7.96 – 7.77 (m, 6H), 7.59 – 7.43 (m, 6H), 7.34 – 7.28 (m, 2H), 4.36 (q, J = 7 Hz, 4H), 3.51 (t, J = 7 Hz, 2H), 3.36 – 3.24 (m, 2H), 2.73 – 2.53 (m, 10H) 1.79 & 1.78 (rotameric singlets, 2x3H), 1.41 (t, J = 7 Hz, 6H). ^{13}C NMR (101 MHz, $CDCl_3$) δ 170.5, 170.5, 167.8, 167.4, 141.1, 139.7, 133.6, 133.5, 132.9, 132.8, 132.4, 132.0, 131.6, 131.2, 129.1, 128.9, 128.8, 128.7, 126.8, 126.6, 126.5, 126.5, 126.2, 125.6, 125.5, 125.3, 124.7, 124.7, 61.4, 61.2, 50.2, 47.1, 36.1, 33.2, 31.0, 27.2, 25.9, 21.7, 20.8, 14.5. HRMS (FI+) (m/z): $[M]^+$ calcd. for $C_{20}H_{23}NO_3$, 325.1678; found: 325.1670. *The geometry of **43** was assigned in analogy with compounds **39-41**.*

Characterization data of compounds 48-52

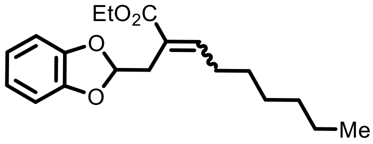

ethyl 2-(benzo[d][1,3]dioxol-2-ylmethyl)non-2-enoate (48). Prepared according to GP6 from **1a** (288 µL, 2.5 mmol, 5.0 equiv, ρ = 1.06 g mL^{-1}), **2** (118 mg, 0.5 mmol) and **1v** (104 µL, 0.75 mmol, 1.5 equiv, ρ = 0.82 g mL^{-1}). Reaction time: 3 h. *d.r.* was determined via ^{1}H NMR of the crude to be 2:1. Purified via flash column chromatography on silica gel (Hexane:Ethyl Acetate 98:2) to afford the two diastereomers as an inseparable mixture as a colorless oil (113 mg, 71% yield). ^{1}H NMR (400 MHz, $CDCl_3$) δ 7.02 (t, *J* = 8 Hz, 1H, *major*), 6.85 – 6.71 (m, 8H, *major+minor*), 6.28 (t, *J* = 5 Hz, 1H, *major*), 6.26 (t, *J* = 5 Hz, 1H, *minor*), 6.13 (tt, J_1 = 7 Hz, J_2 = 1 Hz, 1H, *minor*), 4.23 (q, *J* = 7 Hz, 2H, *minor*), 4.22 (q, *J* = 7 Hz, 2H, *major*), 2.96 (d, *J* = 5 Hz, 2H, *major*), 2.87 (dd, J_1 = 5 Hz, J_2 = 1 Hz, 2H, *minor*), 2.51 (q, *J* = 7 Hz, 2H, *minor*), 2.23 (q, *J* = 7 Hz, 2H, *major*), 1.50 – 1.36 (m, 4H), 1.35 – 1.24 (m, 12H), 1.31 (t, *J* = 7 Hz, 6H, *major+minor*) 0.93 – 0.83 (m, 6H, *major+minor*). ^{13}C NMR (101 MHz, $CDCl_3$) δ 167.4 (*major*), 167.3 (*minor*), 148.7 (*minor*), 147.9 (*major*), 147.6 (*minor*), 147.5 (*major*), 124.8 (*major*), 124.4 (*minor*), 121.5 (*major*), 121.5 (*minor*), 110.3 (*major*), 110.2 (*minor*), 108.6 (*major*), 108.6 (*minor*), 60.9 (*major*), 60.5 (*minor*), 40.2 (*minor*), 32.6 (*major*), 31.8 (*major*), 31.8 (*minor*), 30.0 (*minor*), 29.3 (*major+minor*), 29.2 (*major*), 29.1 (*minor*), 28.7 (*major*), 22.7 (*minor*), 22.7 (*major*), 14.4 (*major*), 14.4 (*minor*), 14.2 (*major+minor*). HRMS *major* (FI+) (m/z): $[M]^+$ calcd. for $C_{19}H_{26}O_4$, 318.1831; found: 318.1830. HRMS *minor* (FI+) (m/z): $[M]^+$ calcd. for $C_{19}H_{26}O_4$, 318.1831; found: 318.1831.

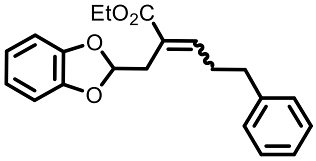

ethyl 2-(benzo[d][1,3]dioxol-2-ylmethyl)-5-phenylpent-2-enoate (49). Prepared according to GP6 from **1a** (288 µL, 2.5 mmol, 5.0 equiv, ρ = 1.06 g mL^{-1}), **2** (118 mg, 0.5 mmol) and **S21** (100 µL, 0.75 mmol, 1.5 equiv, ρ = 1.01 g mL^{-1}). Reaction time: 3 h. *E*:*Z* ratio was determined via ^{1}H NMR of the crude to be 1:1. Purified via flash column chromatography on silica gel (Hexane:Ethyl Acetate 98:2 → 95:5) to afford the two diastereomers as an inseparable mixture as a colorless oil (111 mg, 65% yield). ^{1}H NMR (400 MHz, $CDCl_3$) δ 7.35 – 7.27 (m, 4H), 7.25 – 7.16 (m, 6H), 7.09 (t, *J* = 7 Hz, 1H), 6.86 – 6.74 (m, 8H), 6.27 (t, *J* = 5 Hz, 2H), 6.19 (t, *J* = 7 Hz, 1H), 4.24 (qd, J_1 = 7 Hz, J_2 = 3 Hz, 4H), 2.96 (d, *J* = 5 Hz, 2H), 2.93 – 2.68 (m, 8H), 2.58 (q, *J* = 8 Hz, 2H), 1.32 (td, J_1 = 7 Hz, J_2 = 3 Hz, 6H). ^{13}C NMR (101 MHz, $CDCl_3$) δ 167.2, 167.0, 147.5, 147.5, 147.0, 146.3, 141.4, 141.1, 128.6 (2x), 128.5, 128.5, 126.3, 126.1, 125.5, 125.2, 121.6, 121.5, 110.2, 110.1, 108.6, 108.6, 60.9, 60.6, 40.1, 35.4, 34.8, 32.6, 31.5, 31.1, 14.4 (2x). HRMS *isomer 1* (FI+) (m/z): $[M]^+$ calcd. for $C_{21}H_{22}O_4$, 338.1518; found: 338.1528. HRMS *isomer 2* (FI+) (m/z): $[M]^+$ calcd. for $C_{21}H_{22}O_4$, 338.1518; found: 338.1520.

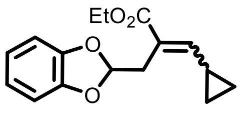

ethyl 2-(benzo[d][1,3]dioxol-2-ylmethyl)-3-cyclopropylacrylate (50). Prepared according to GP6 from **1a** (288 µL, 2.5 mmol, 5.0 equiv, ρ = 1.06 g mL^{-1}), **2** (118 mg, 0.5 mmol) and **S22** (56 µL, 0.75 mmol, 1.5 equiv, ρ = 0.93 g mL^{-1}). Reaction time: 3 h. *E*:*Z* ratio was determined via ^{1}H NMR of the crude to be ca. 1:1. Purified via flash column chromatography on silica gel (Hexane:Ethyl Acetate 98:2 → 95:5) to afford the two diastereomers as an inseparable mixture as a colorless oil (79 mg, 57% yield).

^{1}H NMR (400 MHz, $CDCl_3$) δ 6.83 – 6.71 (m, 8H), 6.37 (d, *J* = 11 Hz, 1H), 6.32 (t, *J* = 5 Hz, 1H), 6.24 (t, *J* = 5 Hz, 1H), 5.40 (d, *J* = 11 Hz, 1H), 4.26 (q, *J* = 7 Hz, 2H), 4.21 (q, *J* = 7 Hz, 2H), 3.06 (d, *J* = 5 Hz, 2H), 2.84 (d, *J* = 5 Hz, 2H), 2.67 (dddd, J_1 = 11 Hz, J_2 = 8 Hz, J_3 = 5 Hz, J_4 = 3 Hz, 1H), 1.75 – 1.61 (m, 1H), 1.32 (t, *J* = 7 Hz, 3H), 1.29 (t, *J* = 7 Hz, 3H), 1.02 – 0.92 (m, 4H), 0.72 – 0.62 (m, 2H), 0.58 – 0.50 (m, 2H). ^{13}C NMR (101 MHz, $CDCl_3$) δ 167.4, 167.2, 154.2, 152.4, 147.5, 147.5, 122.3, 121.6, 121.5, 121.4, 110.4, 110.2, 108.5, 108.5, 60.7, 60.5, 39.9, 32.7, 14.4 (2x), 12.6, 12.2, 9.0, 8.8. HRMS *isomer 1* (FI+) (m/z): $[M]^+$ calcd. for $C_{16}H_{18}O_4$, 274.1205; found: 274.1207. HRMS *isomer 2* (FI+) (m/z): $[M]^+$ calcd. for $C_{16}H_{18}O_4$, 274.1205; found: 274.1210.

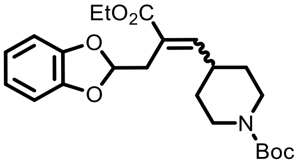

***tert*-butyl 4-(2-(benzo[d][1,3]dioxol-2-ylmethyl)-3-ethoxy-3-oxoprop-1-en-1-yl)piperidine-1-carboxylate (51).** Prepared according to GP6 from **1a** (288 µL, 2.5 mmol, 5.0 equiv, ρ = 1.06 g mL^{-1}), **2** (118 mg, 0.5 mmol) and **S23** (160 mg, 0.75 mmol, 1.5 equiv). Reaction time: 16 h. *Z:E* ratio was determined via ^{1}H NMR of the crude to be 5:1. Purified via flash column chromatography on silica gel (Hexane:Ethyl Acetate 95:5 → 90:10) to afford the two diastereomers as an inseparable mixture as a colorless oil (142 mg, 68%).

Major (Z): ^{1}H NMR (400 MHz, $CDCl_3$) δ 6.84 – 6.68 (m, 4H), 6.24 (t, *J* = 5 Hz, 1H), 5.87 (d, *J* = 10 Hz, 1H), 4.21 (q, *J* = 7 Hz, 2H), 4.14 – 3.96 (m, 2H), 3.11 (m, 1H), 2.85 (d, *J* = 5 Hz, 2H), 2.81 – 2.64 (m, 2H), 1.62 (m, 2H), 1.45 (s, 9H), 1.34 – 1.17 (m, 5H). ^{13}C NMR (101 MHz, $CDCl_3$) δ 166.9, 154.9, 151.0, 147.5, 123.9, 121.5, 109.9, 108.4, 79.5, 60.7, 43.5, 39.9, 36.6, 31.4, 28.5, 14.3. HRMS (ESI+) (m/z): $[M+Na]^+$ calcd. for $C_{23}H_{31}NO_6$, 440.2049; found: 440.2048. NOESY, HSQC experiments are reported below.

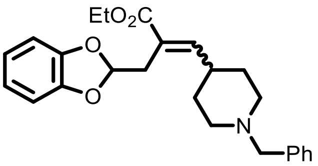

ethyl 2-(benzo[d][1,3]dioxol-2-ylmethyl)-3-(1-benzylpiperidin-4-yl)acrylate (52). Prepared according to GP6 from **1a** (288 µL, 2.5 mmol, 5.0 equiv, ρ = 1.06 g mL^{-1}), **2** (118 mg, 0.5 mmol) and **S24** (145 µL, 0.75 mmol, 1.5 equiv, ρ = 1.05 g mL^{-1}). Reaction time: 16 h. Purified via flash column chromatography on silica gel (Hexane:Ethyl Acetate 95:5 → 90:10) to afford the two diastereomers (*Z*:*E* ratio 5:1 by NMR) as an inseparable mixture as a colorless oil (122 mg, 60%).

Major (Z): ^{1}H NMR (400 MHz, $CDCl_3$) δ 7.34 (m, 4H), 7.31 – 7.22 (m, 1H), 6.79 (m, 4H), 6.33 – 6.25 (m, 1H), 5.97 (d, J = 10 Hz, 1H), 4.30 – 4.20 (m, 2H), 3.52 (s, 2H), 3.06 – 2.96 (m, 1H), 2.96 – 2.84 (m, 4H) 2.02 (tdd, J_1 = 14 Hz, J_2 = 10 Hz, J_3 = 4 Hz, 2H), 1.79 – 1.62 (m, 2H), 1.59 – 1.38 (m, 2H), 1.33 (m, 3H). ^{13}C NMR (101 MHz, $CDCl_3$) δ 167.1, 152.1, 147.5, 138.5, 129.3, 128.3, 127.0, 123.5, 121.4, 110.1, 108.4, 63.6, 60.6, 53.3, 40.0, 36.6, 31.8, 14.3. HRMS (FI+) (m/z): $[M]^+$ calcd. for $C_{25}H_{29}NO_4$, 407.2097; found: 407.2104. NOESY experiment is reported below.

Characterization data of compounds 53-55

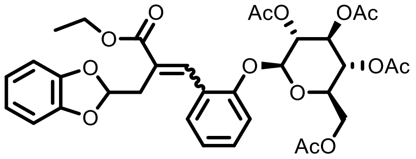

(2R,3R,4S,5R,6S)-2-(acetoxymethyl)-6-(2-(2-(benzo[d][1,3]dioxol-2-ylmethyl)-3-ethoxy-3-oxoprop-1-en-1-yl)phenoxy)tetrahydro-2H-pyran-3,4,5-triyl triacetate (53). Prepared according to GP4 from **1a** (288 µL, 2.5 mmol, 5.0 equiv, ρ = 1.06 g mL^{-1}), **2** (118 mg, 0.5 mmol) and **S25** (0.75 mmol, 1.5 equiv, 339 mg). Reaction time: 24 h. Purified via flash column chromatography on silica gel (Hexane:Ethyl Acetate 70:30) to afford the two diastereomers as an inseparable mixture (82 mg, 25% yield, 40% RSM). *E*:*Z* ratio was determined via ^{1}H NMR of the collected purified product to be 3:1.

^{1}H NMR (400 MHz, $CDCl_3$) δ 7.87 (s, 1H, *major*), 7.50 – 7.43 (m, 1H, *major*), 7.28 – 7.26 (m, 1H, *minor*), 7.26 – 7.17 (m, 1H, *major*), 7.05 (dd, J_1 = 8 Hz, J_2 = 1 Hz, 1H, *major*), 7.00 – 6.94 (m, 1H *major* + 2H *minor*), 6.90 (s, 1H, *minor*), 6.83 – 6.66 (m, 4H *major* + 5H *minor*), 6.41 (t, *J* = 5 Hz, 1H, *major*), 6.41 (t, *J* = 5 Hz, 1H, *minor*), 5.37 – 5.23 (m, 2H *major* + 2H *minor*), 5.22 – 5.11 (m, 1H *major* + 1H *minor*), 5.11 – 4.99 (m, 1H *major* + 1H *minor*), 4.34 – 4.25 (m, 3H *major* + 1H *minor*), 4.20 – 4.12 (m, 1H *major* + 1H *minor*), 4.10 – 4.03 (m, 2H, *minor*), 3.90 – 3.83 (m, 1H *major* + 1H minor), 3.06 (d, *J* = 5 Hz, 2H, *major*), 3.01 (ddd, J_1 = 13, J_2 = 5 Hz, J_3 = 1 Hz, 2H, *minor*), 2.07 (s, 3H *major*), 2.06 (s, 3H *minor*), 2.04 – 2.03 (m, 3H *major* + 3H *minor*), 2.02 (s, 3H *major*), 2.01 (s, 3H *major*), 2.00 (s, 3H *minor*), 1.98 (s, 3H *minor*) 1.37 (t, *J* = 7 Hz, 3H, *major*), 1.03 (t, *J* = 7 Hz, 3H, *minor*). ^{13}C NMR (101 MHz, $CDCl_3$) δ 170.6 (*major*), 170.6 (*minor*), 170.3 (*major* + *minor*), 169.5 (*major*), 169.5 (*minor*), 169.2 (*minor*), 169.1 (*major*), 168.2 (*minor*), 167.4 (*major*), 154.9 (*major*), 154.1 (*minor*), 147.5 (*minor*), 147.4 (*minor*), 147.3 (*major*), 147.3 (*major*), 138.6 (*major*), 135.1 (*minor*), 130.2 (*major*), 130.2 (*major*), 130.1 (*minor*), 129.5 (*minor*), 127.8 (*minor*), 126.9 (*major*), 126.7 (*minor*), 125.6 (*major*), 123.3 (*major*), 122.7 (*minor*), 121.6 (*major*), 121.6 (*major*), 121.5 (*minor*), 121.5 (*minor*), 115.5 (*major*), 114.8 (*minor*), 110.0 (*minor*), 110.0 (*major*), 108.7 (*major*), 108.7 (*minor*), 108.6 (*minor*), 108.6 (*major*), 99.5 (*major*), 99.3 (*minor*), 72.7 (*major*), 72.7 (*minor*), 72.2 (*major*), 72.1 (*minor*), 71.1 (*minor*), 70.9 (*major*), 68.4 (1C *major* + 1C *minor*), 62.0 (*major*), 62.0 (*minor*), 61.3 (*major*), 60.8 (*minor*), 40.5 (*minor*), 33.6 (*major*), 20.8 – 20.5 (4C *major* + 4C *minor*), 14.3 (*major*), 13.8 (*minor*). HRMS (FD+) (m/z): $[M]^+$ calcd. for $C_{33}H_{36}O_{14}$, 656.2105; found: 656.2101. NOESY, COSY, HSQC experiments are reported below

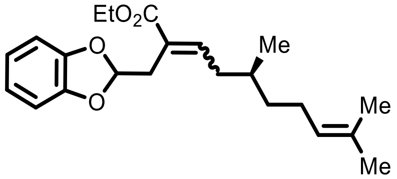

ethyl (S)-2-(benzo[d][1,3]dioxol-2-ylmethyl)-5,9-dimethyldeca-2,8-dienoate (54). Prepared according to GP6 from **1a** (288 µL, 2.5 mmol, 5.0 equiv, ρ = 1.06 g mL^{-1}), **2** (118 mg, 0.5 mmol) and **S26** (136 µL, 0.75 mmol, 1.5 equiv, ρ = 0.85 g mL^{-1}). Reaction time: 3 h. *d.r.* ratio was determined via ^{1}H NMR of the crude to be 2:1. Purified via flash column chromatography on silica gel (Hexane → Hexane:Ethyl Acetate 98:2) to afford the two diastereomers as an inseparable mixture (113 mg, 63% combined yield). ^{1}H NMR (400 MHz, $CDCl_3$) δ 7.05 (t, *J* = 7 Hz, 1H *minor*), 6.77 (m, 4H *major* + 4H *minor*), 6.27 (t, *J* = 7 Hz, 1H *major*), 6.27 (t, *J* = 7 Hz, 1H *minor*), 6.14 (t, *J* = 7 Hz, 1H *major*), 5.14 – 5.04 (m, 1H *major* + 1H *minor*), 4.23 (q, *J* = 7

Hz, 2H *major*), 4.23 (q, J = 7 Hz, 2H *minor*), 2.96 (d, J = 5 Hz, 2H *minor*), 2.89 (d, J = 5 Hz, 2H *major*), 2.59 – 2.36 (m, 2H *major*), 2.29 – 2.07 (m, 2H, *minor*), 2.05 – 1.87 (m, 2H *major* + 2H *minor*), 1.70 (s, 3H *major* + 3H *minor*), 1.70 – 1.63 (m, 1H *minor*), 1.61 – 1.53 (m, 1H *major*), 1.60 (s, 3H *major*), 1.60 (s, 3H *minor*), 1.42 – 1.25 (m, 4H *major* + 4H *minor*), 1.25 – 1.13 (m, 1H *major* + 1H *minor*), 0.91 (t, J = 7 Hz, 3H *major*), 0.91 (t, J = 7 Hz, 3H *minor*). ^{13}C NMR (101 MHz, $CDCl_3$) δ 167.3 (*minor*), 167.3 (*major*), 147.6 (*major*), 147.5 (*minor*), 146.7 (*major+minor*), 131.6 (*minor*), 131.4 (*major*), 125.5 (*minor*), 125.0 (*minor*), 124.8 (*major*), 124.6 (*major*), 121.5 (*minor*), 121.5 (*major*), 110.3 (*minor*), 110.2 (*major*), 108.6 (*major+minor*), 60.9 (*minor*), 60.5 (*major*), 40.2 (*major*), 37.0 (*minor*), 37.0 (*major*), 36.9 (*major*), 36.4 (*minor*), 33.3 (*major+minor*), 32.8 (*minor*), 25.8 (*major*), 25.8 (*minor*), 25.7 (*minor*), 25.7 (*major*), 19.8 (*minor*), 19.6 (*major*), 17.8 (*minor*), 17.8 (*major*), 14.4 (*minor*), 14.4 (*major*). HRMS *isomer 1* (FI+) (m/z): $[M]^+$ calcd. for $C_{22}H_{30}O_4$, 358.2144; found: 358.2139. HRMS *isomer 2* (FI+) (m/z): $[M]^+$ calcd. for $C_{22}H_{30}O_4$, 358.2144; found: 358.2134. COSY, HSQC experiments are reported below.

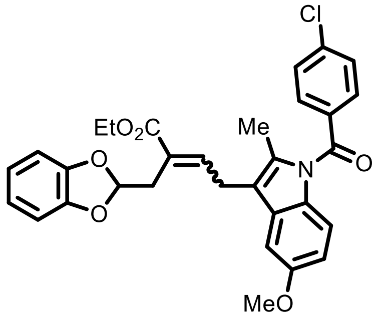

ethyl 2-(benzo[d][1,3]dioxol-2-ylmethyl)-4-(1-(4-chlorobenzoyl)-5-methoxy-2-methyl-1H-indol-3-yl)but-2-enoate (55). Prepared according to GP6 from **1a** (288 µL, 2.5 mmol, 5.0 equiv, ρ = 1.06 g mL^{-1}), **2** (118 mg, 0.5 mmol) and **S27** (256 mg, 0.75 mmol, 1.5 equiv). Reaction time: 16 h. Purified via flash column chromatography on silica gel (Hexane:Ethyl Acetate 90:10) to afford the two diastereomers (54 mg, 20% combined yield, *Z*:*E* ratio 3:1 by NMR, 43% RSM). Further purification afforded the clean major isomer. ^{1}H NMR (400 MHz, $CDCl_3$) δ 7.71 – 7.60 (m, 2H), 7.50 – 7.44 (m, 2H), 6.97 (d, J = 2 Hz, 1H), 6.89 (d, J = 9 Hz, 1H), 6.80 – 6.64 (m, 5H), 6.28 (t, J = 5 Hz, 1H), 6.23 – 6.10 (m, 1H), 4.34 (q, J = 7 Hz, 2H), 3.95 (d, J = 7 Hz, 2H), 3.83 (s, 3H), 2.91 (dd, J_1 = 5 Hz, J_2 = 1 Hz, 2H), 2.32 (s, 3H), 1.41 – 1.28 (m, 3H). ^{13}C NMR (101 MHz, $CDCl_3$) δ 168.5, 167.1, 156.1, 147.4, 145.5, 139.3, 134.8, 134.3, 131.3, 131.1, 131.0, 129.2, 125.0, 121.6, 117.5, 115.1, 111.6, 110.0, 108.6, 101.7, 60.9, 55.8, 40.0, 24.9, 14.5, 13.4. HRMS (FI+) (m/z): $[M]^+$ calcd. for $C_{31}H_{28}ClNO_6$, 407.2097; found: 407.2104. NOESY experiment is reported below.

8. Characterization data of compounds 56, 59-65

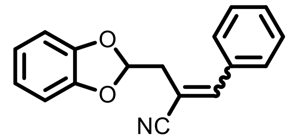

2-(benzo[d][1,3]dioxol-2-ylmethyl)-3-phenylacrylonitrile (56). Prepared according to GP4 from **1a** (115 μL, 1.0 mmol, 5.0 equiv, ρ = 1.06 g mL^{-1}), **2'** (38 mg, 0.2 mmol) and **S2** (0.3 mmol, 1.5 equiv.). Purified via flash column chromatography on silica gel (Hexane:Ethyl Acetate 40:1) to afford product **56** (12 mg, 22%, d.r 20:1).

^{1}H NMR (400 MHz, $CDCl_3$) δ 6.85 – 6.71 (m, 4H), 6.24 (t, *J* = 5 Hz, 1H), 6.21 (s, 1H), 6.05 (t, *J* = 1 Hz, 1H), 2.92 (dd, J_1 = 5 Hz, J_2 = 1 Hz, 2H), 2.37 (s, 3H). ^{13}C NMR (101 MHz, $CDCl_3$) δ 199.1, 147.5, 142.0, 129.7, 121.6, 109.6, 108.6, 36.4, 25.7. HRMS (FI+) (m/z): $[M]^+$ calcd. for $C_{17}H_{13}NO_2$, 263.0946; found, 263.0933.

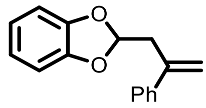

2-(2-phenylallyl)benzo[*d*][1,3]dioxole (59). Prepared according to GP7 from **1a** (115 μL, 1.0 mmol, 5.0 equiv, ρ = 1.06 g mL^{-1}), **2''''** (48 mg, 0.2 mmol) and **S1** (18 mg, 0.6 mmol, 3 equiv.). Purified via flash column chromatography on silica gel (Hexane:Ethyl Acetate 99:1) to afford product **59** (27 mg; ^{1}H-NMR yield of the first step: 90%; isolated yield of the second step: 64%; yield over two steps: 58%).

^{1}H NMR (400 MHz, $CDCl_3$) δ 7.48 – 7.43 (m, 2H), 7.40 – 7.33 (m, 2H), 7.33 – 7.27 (m, 1H), 6.79 (s, 4H), 6.22 (t, *J* = 5 Hz, 1H), 5.52 (d, *J* = 1 Hz, 1H), 5.31 (q, *J* = 1 Hz, 1H), 3.16 (dd, J_1 = 5 Hz, J_2 = 1 Hz, 2H).

^{13}C NMR (101 MHz, $CDCl_3$) δ 147.5, 141.5, 140.5, 128.6, 127.9, 126.3, 121.6, 116.6, 110.2, 108.7, 40.8.

HRMS (FI+) (m/z): $[M]^+$ calcd. for $C_{16}H_{14}O_2$, 238.0994; found: 238.0999.

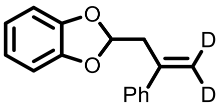

2-(2-phenylallyl-3,3-d2)benzo[*d*][1,3]dioxole (60). Prepared according to GP7 from **1a** (115 μL, 1.0 mmol, 5.0 equiv, ρ = 1.06 g mL^{-1}), **2''''** (48 mg, 0.2 mmol) and **S1-d$_2$** (19 mg, 0.6 mmol, 3 equiv.). Purified via flash column chromatography on silica gel (Hexane:Ethyl Acetate 98:2) to afford product **60** (30 mg; ^{1}H-NMR yield of the first step: 90%; isolated yield of the second step: 63%; yield over two steps: 57%).

^{1}H NMR (400 MHz, $CDCl_3$) δ 7.49 – 7.42 (m, 2H), 7.40 – 7.34 (m, 2H), 7.33 – 7.27 (m, 1H), 6.85 – 6.73 (m, 4H), 6.23 (t, *J* = 5 Hz, 1H), 3.16 (d, *J* = 5 Hz, 2H).

^{13}C NMR (101 MHz, $CDCl_3$) δ 147.5, 141.4, 140.5, 128.6, 127.9, 126.2, 121.5, 110.2, 108.7, 40.7.

HRMS (FI+) (m/z): $[M]^+$ calcd. for $C_{16}H_{12}D_2O_2$, 240.1119; found: 240.1114.

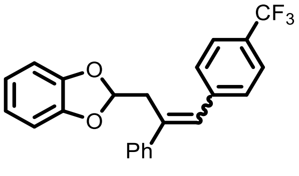

2-(2-phenyl-3-(4-(trifluoromethyl)phenyl)allyl)benzo[d][1,3]dioxole (61). Prepared according to GP7 from **1a** (115 µL, 1.0 mmol, 5.0 equiv, ρ = 1.06 g mL^{-1}), **2''''** (48 mg, 0.2 mmol) and **S4** (41 µL, 0.30 mmol, 1.5 equiv, ρ = 1.28 g mL^{-1}). Purified via flash column chromatography on silica gel (Hexane:Ethyl Acetate 99:1) to afford product **61** as an inseparable mixture of the two diastereomers (*E:Z* 3:1 ratio) as a colorless oil (52 mg; ^{1}H-NMR yield of the first step: 90%; isolated yield of the second step: 68%; yield over two steps: 61%).

^{1}H NMR (300 MHz, $CDCl_3$) δ 7.63 – 7.46 (m, 6H *major*), 7.45 – 7.29 (m, 3H *major* + 5H *minor*), 7.20 (dd, *J* = 7.4, 2.1 Hz, 2H *minor*), 7.05 (d, *J* = 8 Hz, 2H *minor*), 6.92 (s, 1H *major*), 6.86 – 6.68 (m, 4H *major* + 4H *minor*), 6.64 (s, 1H *minor*), 6.14 (t, *J* = 5 Hz, 1H *major*), 6.13 (t, *J* = 5 Hz, 1H *minor*), 3.35 (dd, J_1 = 5 Hz, J_2= 1 Hz, 2H *major*), 3.15 (dd, J_1 = 5 Hz, J_2= 1 Hz, 2H *minor*). ^{13}C NMR (75 MHz, $CDCl_3$) δ 147.5 (*minor*), 147.3 (*major*), 141.6 (*major*), 141.0 (*major*), 140.5 (*minor*), 139.7 (*minor*), 138.3 (*minor*), 137.5 (*major*), 131.2 (*major*), 129.4, 129.3, 129.1, 128.9, 128.8, 128.6, 128.2, 127.9, 126.9, 125.5 (*major*, q, *J* = 4 Hz), 124.9 (*minor*, q, *J* = 4 Hz), 124.3 (*major*, q, *J* = 272 Hz) 124.3 (*minor*, q, *J* = 272 Hz), 121.7 (*major*), 121.6 (*minor*), 109.8 (*major*), 109.6 (*minor*), 108.7 (*minor*), 108.7 (*major*), 45.7 (*minor*), 36.1 (*major*). ^{19}F NMR (282 MHz, $CDCl_3$) δ -62.48, -62.54.

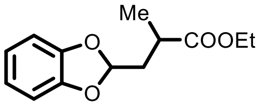

ethyl 3-(benzo[d][1,3]dioxol-2-yl)-2-methylpropanoate (62). Prepared according to a procedure adapted from the literature.[11] In particular, an oven-dried vial was charged with 10 mg of 10% Pd/C, then compound **4** (0.2 mmol) was added. Toluene (1 mL) was added, followed by acetic acid (23 µL, 0.4 mmol, 2 equiv, ρ = 1.049 g mL^{-1}) and $NaBH_4$ (30 mg, 0.8 mmol, 4 equiv). The resulting mixture was stirred for 2 hours, after which it was carefully quenched with HCl 0.1 N. Finally, sat'd $NaHCO_3$ was added to neutralized the excess of acid. The mixture was extracted with diethyl ether (3x10 mL) and the collected organic phases were dried over $MgSO_4$ and the solvent removed under reduced pressure. The crude was purified via flash column chromatography on silica gel (Hexane:Ethyl Acetate 97:3 → 95:5) to get 33 mg the product as a colorless oil (70% yield). ^{1}H NMR (400 MHz, $CDCl_3$) δ 6.86 – 6.71 (m, 4H), 6.17 (dd, J_1 = 5 Hz, J_2 = 4 Hz, 1H), 4.14 (qd, J_1 = 7 Hz, J_2 = 1 Hz, 2H), 2.79 (dqd, J_1 = 8 Hz, J_2 = 7 Hz, J_3 = 5 Hz, 1H), 2.42 (ddd, J_1 = 14 Hz, J_2 = 8 Hz, J_3 = 4 Hz, 1H), 2.01 (dt, J_1 = 14 Hz, J_2 = 5 Hz, 1H), 1.27 (d, *J* = 7 Hz, 3 H), 1.24 (t, *J* = 7 Hz, 3H). ^{13}C NMR (101 MHz, $CDCl_3$) δ 175.8, 147.6, 121.6, 121.6, 110.0, 108.6, 60.8, 38.1, 34.6, 17.9, 14.3. HRMS (FI+) (m/z): $[M]^+$ calcd. for $C_{13}H_{16}O_4$, 236.1049; found: 236.1054.

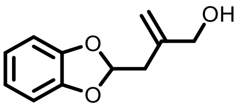

2-(benzo[d][1,3]dioxol-2-ylmethyl)prop-2-en-1-ol (63). Prepared according to a procedure adapted from the literature.[12] In particular, in an oven-dried 10 mL round-bottom flask compound **4** was dissolved in 0.8 mL of dry toluene and DIBAL-H (880 µL, 0.88 mmol, 4.4 equiv; 1 M solution in hexanes was used) was added dropwise under inert atmosphere (N_2) at 0 °C (ice bath). After 30 minutes, the ice bath was removed ant the solution was stirred for 2 hours at room temperature. Finally, the reaction was quenched with HCl 0.1 N, neutralized with sat'd $NaHCO_3$, filtered through a pad of celite to remove aluminum salts and finally extracted with Ethyl Acetate (3x10 mL). The organic phases were collected, dried over Na_2SO_4 and the solvent was removed under reduced pressure. The crude was purified via flash column chromatography on

silica gel (Hexane:Ethyl Acetate 80:20) to get 24 mg the product as a colorless oil (62% yield). ^{1}H NMR (400 MHz, $CDCl_3$) δ 6.79 (dd, J_1 = 4 Hz, J_2 = 2 Hz, 4H), 6.24 (t, J = 5 Hz, 1H), 5.26 (q, J = 2 Hz, 1H), 5.13 (q, J = 1 Hz, 1H), 4.18 (s, 2H), 2.75 (dd, J_1 = 5 Hz, J_2 = 1 Hz, 2H), 1.76 (bs, 1H).^{13}C NMR (101 MHz, $CDCl_3$) δ 147.5, 142.0, 121.7, 114.9, 110.6, 108.7, 66.4, 38.4. HRMS (FI+) (m/z): $[M]^+$ calcd. for $C_{11}H_{12}O_3$, 192.0786; found: 192.0781.

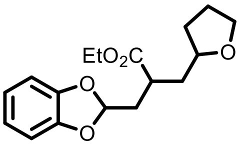

ethyl 3-(benzo[d][1,3]dioxol-2-yl)-2-((tetrahydrofuran-2-yl)methyl)propanoate (64). Ethyl 2-(benzo[d][1,3]dioxol-2-ylmethyl)acrylate (**4**, 0.35 mmol, 81 mg) and TBADT (58 mg, 5 mol%) were dissolved in CH_3CN (3.5 mL) in an oven-dried 7 mL vial. The vial was sealed with a rubber septum and the solution was sparged with N_2 (3 min). After degassing, **1c** (142 µL, 1.75 mmol, 5 equiv, ρ = 0.89 g mL^{-1}) was added via syringe through the septum. The resulting solution was irradiated with a PR160L Kessil lamp (390 nm, 40 W) for 16 hours. After irradiation, the vial was opened, and the solvent was removed under reduced pressure. The crude was purified via flash column chromatography on silica gel (Hexane:Ethyl Acetate 95:5) to afford an inseparable mixture of the two diastereomers (ca 1:1 ratio) as a colorless oil (66 mg, 62% yield). ^{1}H NMR (400 MHz, $CDCl_3$) δ 6.83 – 6.69 (m, 8H), 6.17 – 6.12 (m, 2H), 4.23 – 4.03 (m, 4H), 3.89 – 3.77 (m, 4H), 3.73 – 3.64 (m, 2H), 2.92 (tt, J_1 = 9 Hz, J_2 = 5 Hz, 1H), 2.81 (dddd, J_1 = 9 Hz, J_2 = 8 Hz, J_3 = 6 Hz, J_4 = 4 Hz, 1H), 2.44 – 2.34 (m, 2H), 2.17 – 2.09 (m, 2H), 2.06 – 1.78 (m, 8H), 1.75 – 1.60 (m, 2H), 1.52 – 1.38 (m, 2H), 1.22 (t, J_1 = 7 Hz, 3H), 1.22 (t, J_1 = 7 Hz, 3H). ^{13}C NMR (101 MHz, $CDCl_3$) δ 175.3, 175.2, 147.6, 147.6, 147.5, 121.5, 121.5, 121.5, 109.9, 108.6, 108.6, 108.6, 77.0, 76.9, 67.8, 67.7, 60.7, 38.7, 38.4, 38.0, 37.9, 37.3, 36.7, 31.7, 31.6, 25.7, 25.7, 14.3, 14.2. HRMS (FI+) (m/z): $[M]^+$ calcd. for $C_{17}H_{22}O_5$, 306.1467; found: 306.1474.

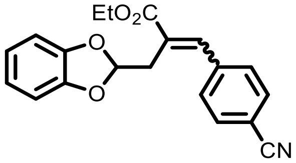

ethyl 2-(benzo[d][1,3]dioxol-2-ylmethyl)-3-(4-cyanophenyl)acrylate (65). Prepared according to a procedure adapted from the literature.[13] In particular, **4** (70 mg, 0.30 mmol) and 4-bromobenzonitrile (109 mg, 0.60 mmol, 2 equiv) were added in an oven-dried 5 ml Schlenk flask under under N_2 atmosphere. Dry DMF (3.0 mL) was added, followed by triethylamine (83 µL, 0.60 mmol, 2 equiv, ρ = 0.728 g mL^{-1}), triphenylphosphine (8.0 mg, 0.030 mmol, 0.1 equiv) and $Pd(OAc)_2$ (3.4 mg, 0.015 mmol, 0.05 equiv). The resulting mixture was stirred at 110°C (oil bath) for 48 h, after which it was cooled to room temperature and quenched with HCl 0.1 M. The mixture was extracted with ethyl acetate (3x10 mL) and the collected organic phases were dried over Na_2SO_4 and the solvent removed under reduced pressure. *d.r.* was determined via ^{1}H NMR of the crude to be 4:1. The crude was purified via flash column chromatography on silica gel (Hexane:Ethyl Acetate 95:5 → 90:10) to afford the two diastereomers (60 mg, 60% combined yield).
Major (E): colorless oil. ^{1}H NMR (400 MHz, $CDCl_3$) δ 7.89 (s, 1H), 7.61 (d, J = 8 Hz, 2H), 7.54 (d, J = 8 Hz, 2H), 6.85 – 6.71 (m, 4H), 6.44 (t, J = 5 Hz, 1H), 4.33 (q, J = 7 Hz, 2H), 3.10 (d, J = 5 Hz, 2H), 1.37 (t,

J = 7 Hz, 3H). ^{13}C NMR (101 MHz, $CDCl_3$) δ 167.0, 147.1, 141.1, 139.8, 132.3, 129.8, 128.7, 121.9, 118.5, 112.3, 109.6, 108.7, 61.7, 33.5, 14.4. HRMS (FI+) (m/z): $[M]^+$ calcd. for $C_{20}H_{17}NO_4$, 335.1158; found: 335.1157.

9. Scale-up procedure (10 mmol)

Ethyl 2-(diethoxyphosphoryl)acrylate (2.4 g, 10 mmol), **1a** (5.8 mL, 50 mmol, 5 equiv) and TBADT (166 mg, 1 mol%) were dissolved in dry CH_3CN (50 mL) in an oven-dried 100 mL round-bottom flask. The flask was sealed with a rubber septum and the solution was sparged with N_2 (10 min). The mixture was taken up with a 50 mL syringe and mounted on a syringe pump (Feed A) connected to a Vapourtec system UV-150 equipped with a 3.06 mL PFA coil (ID = 0.75 mm) and 60 W 365 nm LEDs.
Parallelly, in an oven-dried vial a stock solution 0.11 M in paraformaldehyde and 0.084 M in LiOtBu (a 1 M in dry THF solution was used) was prepared. Upon addition of LiOtBu and following sonication (10 min) the suspension turned to a flowable solution. This stock solution was used for Feed B.
Feed A was pumped at 0.612 mL min^{-1} through the Vapourtec system (V = 3.06 mL, τ_R=5 min). The blue outflow of the latter (due to the reduced form of the photocatalyst, TBADT) was then mixed with Feed B (pumped at 0.802 mL min^{-1}) through a PEEK T-mixer. When the outflow of the photoreactor turned back to colorless (marking the end of the photoreaction), neat acetonitrile was loaded on both syringe pumps to push the combined feeds into a 11.3 mL PFA coil (ID = 0.75 mm) at 1.414 mL min^{-1} (τ_R = 8 min). This second coil was kept in an ultrasonic bath at 40 °C. Finally, the resulting reaction crude was directly collected into a sat'd NH_4Cl solution for quenching (Figure S2). The mixture was extracted three times with Ethyl Acetate and the organic phases were collected and dried over Na_2SO_4. After filtration and rotary evaporation, the crude was analyzed via ^{1}H NMR to determine the reaction yield (CH_2Br_2 as the external standard) and product **4** was purified via column chromatography (SiO_2, Cyclohexane: Ethyl Acetate 98:2) to afford 1.52 g of the pure compound (65% yield).

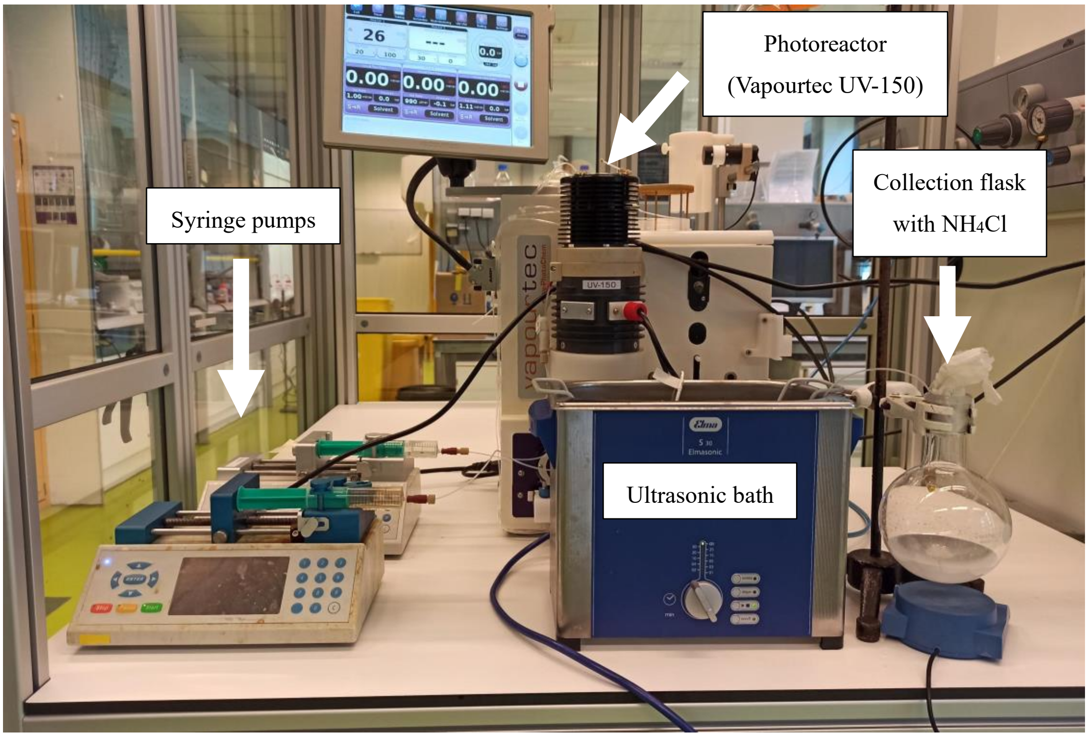

Figure S2. Picture of the experimental setup used for the 10 mmol scale-up.

10. Limitation of the scope

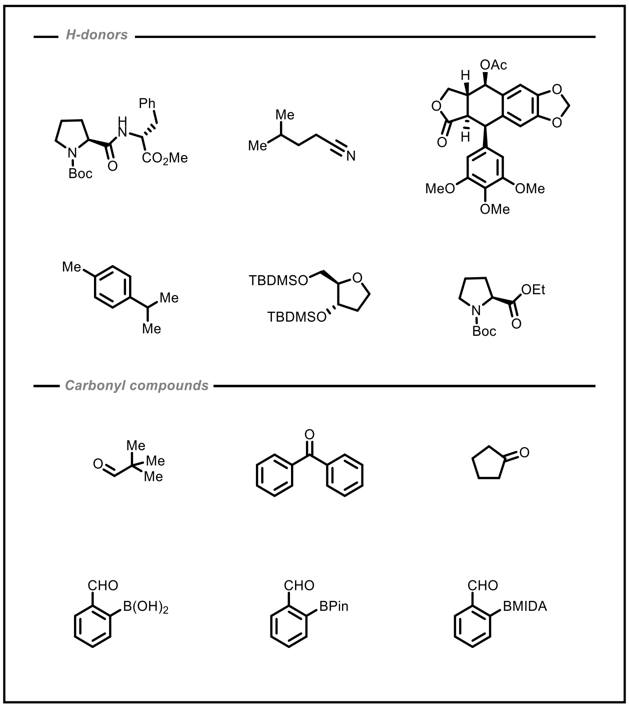

11. References

1. Magalhães, J. *et al.* Refining the structure–activity relationships of 2-phenylcyclopropane carboxylic acids as inhibitors of O-acetylserine sulfhydrylase isoforms. *J. Enzyme Inhib. Med. Chem.* **34**, 31–43 (2019).
2. Protti, S., Ravelli, D., Fagnoni, M. & Albini, A. Solar light-driven photocatalyzed alkylations. Chemistry on the window ledge. *Chem. Commun.* 7351–7353 (2009) doi:10.1039/b917732a.
3. Abadie, B., Jardel, D., Pozzi, G., Toullec, P. & Vincent, J. M. Dual Benzophenone/Copper-Photocatalyzed Giese-Type Alkylation of C(sp3)–H Bonds. *Chem. - A Eur. J.* **25**, 16120–16127 (2019).
4. Ackerman, L. K. G., Martinez Alvarado, J. I. & Doyle, A. G. Direct C-C Bond Formation from Alkanes Using Ni-Photoredox Catalysis. *J. Am. Chem. Soc.* **140**, 14059–14063 (2018).
5. Van Quaquebeke, E. *et al.* 2,2,2-Trichloro-N-({2-[2-(dimethylamino)ethyl]-1,3-dioxo-2, 3-dihydro-1H-benzo[de]isoquinolin-5-yl}carbamoyl)acetamide (UNBS3157), a novel nonhematotoxic naphthalimide derivative with potent antitumor activity. *J. Med. Chem.* **50**, 4122–4134 (2007).
6. Cao, H. Q. *et al.* Silver-Promoted Direct Phosphorylation of Bulky C(sp2)-H Bond to Build Fully Substituted β-Phosphonodehydroamino Acids. *Org. Lett.* **22**, 6414–6419 (2020).
7. De Fusco, C., Fuoco, T., Croce, G. & Lattanzi, A. Noncovalent Organocatalytic Synthesis of Enantioenriched Terminal Aziridines with a Quaternary Stereogenic Center. *Org. Lett.* **14**, 4078–4081 (2012).
8. Chen, T., Zhao, C. Q. & Han, L. B. Hydrophosphorylation of Alkynes Catalyzed by Palladium: Generality and Mechanism. *J. Am. Chem. Soc.* **140**, 3139–3155 (2018).
9. Corcé, V. *et al.* Silicates as Latent Alkyl Radical Precursors: Visible-Light Photocatalytic Oxidation of Hypervalent Bis-Catecholato Silicon Compounds. *Angew. Chem. Int. Ed.* **54**, 11414–11418 (2015).
10. Liang, L. *et al.* Copper-Catalyzed Intermolecular Alkynylation and Allylation of Unactivated C(sp3)-H Bonds via Hydrogen Atom Transfer. *Org. Lett.* **23**, 8575–8579 (2021).
11. Russo, A. T., Amezcua, K. L., Huynh, V. A., Rousslang, Z. M. & Cordes, D. B. A simple borohydride-based method for selective 1,4-conjugate reduction of α,β-unsaturated carbonyl compounds. *Tetrahedron Lett.* **52**, 6823–6826 (2011).
12. Ghera, E., Yechezkel, T. & Hassner, A. Utilization of Ethyl 2-((Phenylsulfonyl)methyl)acrylate for the Synthesis of α-Methylenevalerolactones. *J. Org. Chem.* **55**, 5977–5982 (1990).
13. Bianco, A., Cavarischia, C. & Guiso, M. Total synthesis of anthocyanidins via Heck reaction. *Nat. Prod. Res.* **20**, 93–97 (2006).

12. NMR Spectra of compounds 2', 2''.

^{1}H NMR (400 MHz, $CDCl_3$) of compound **2'**

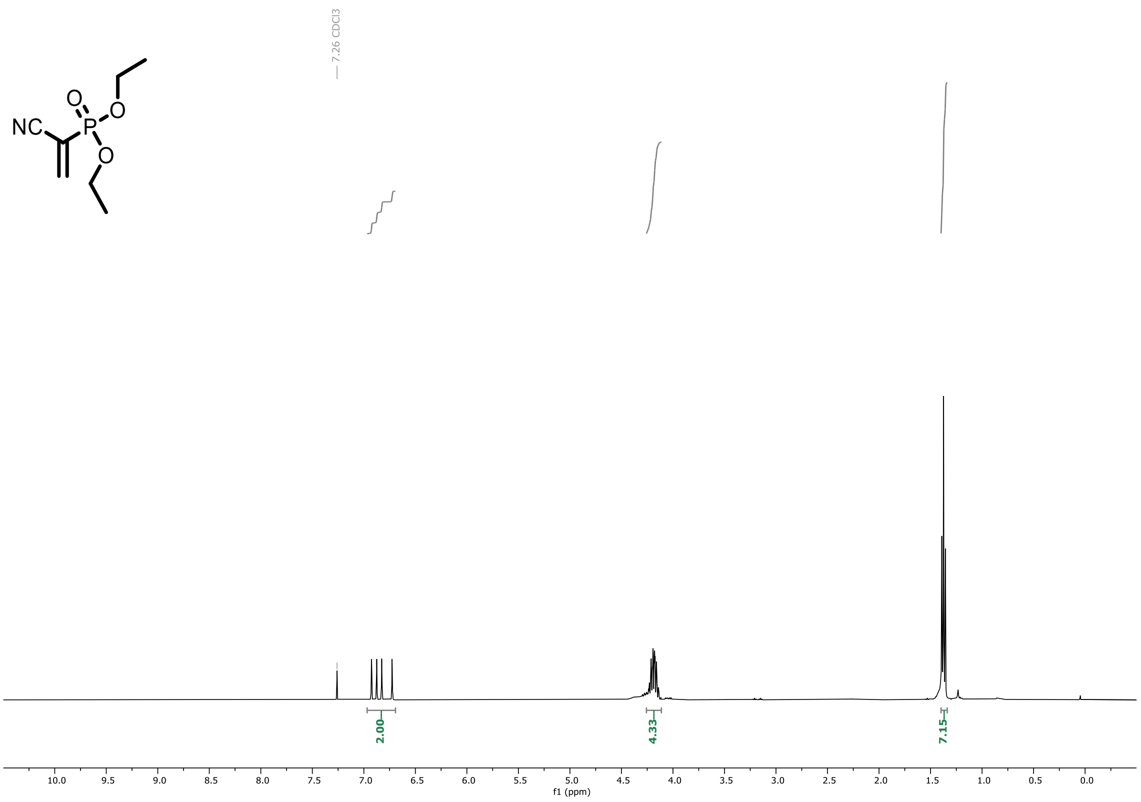

^{13}C NMR (101 MHz, $CDCl_3$) of compound **2'**

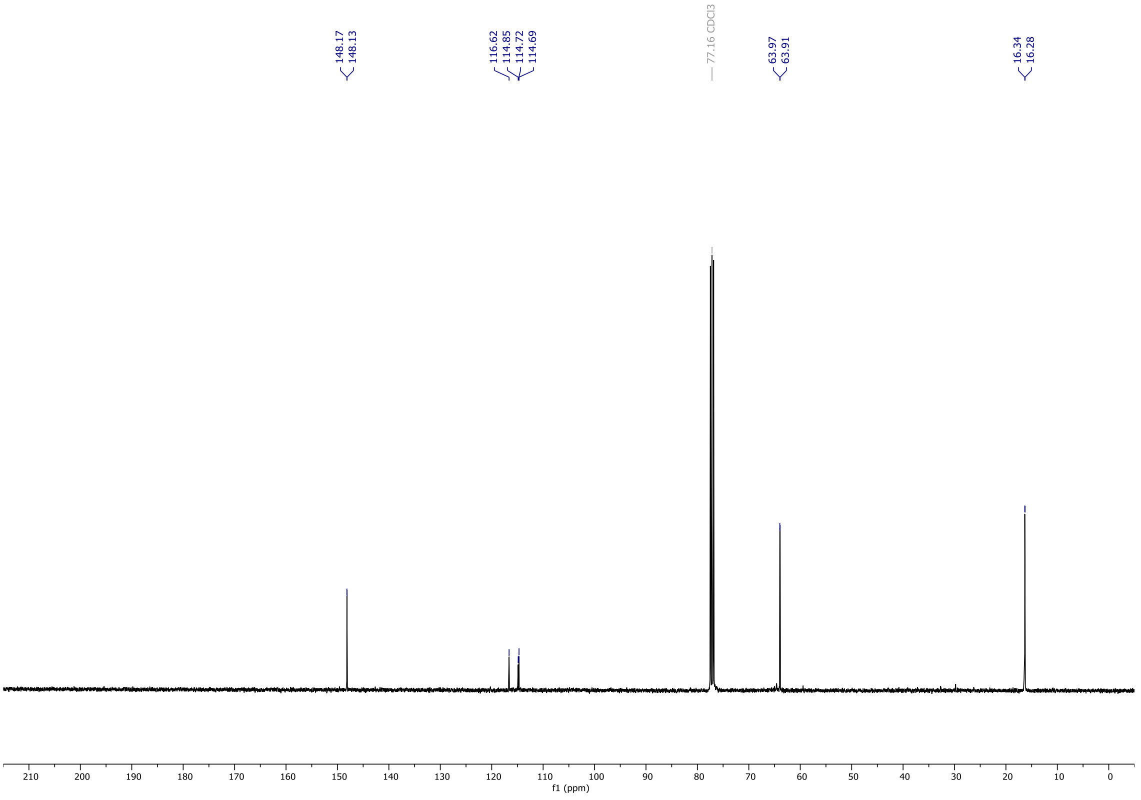

^{31}P NMR (121 MHz, $CDCl_3$) of compound **2'**

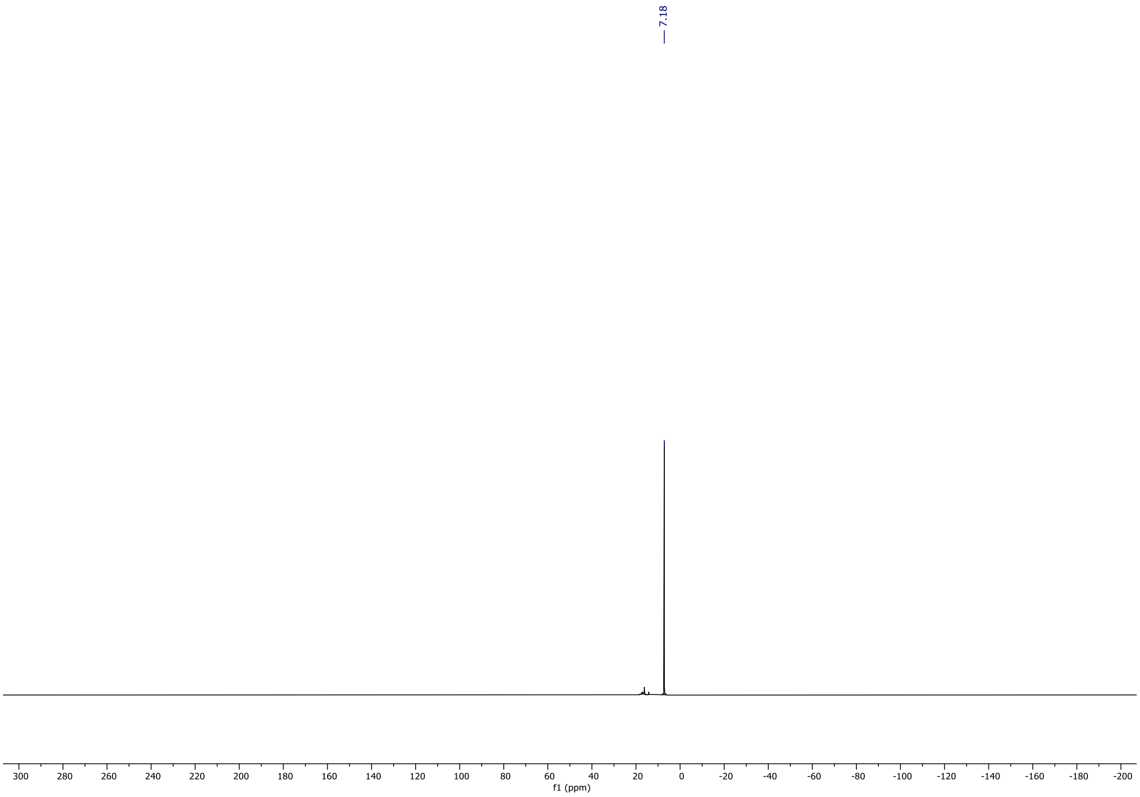

^{1}H NMR (400 MHz, $CDCl_3$) of compound **2''**

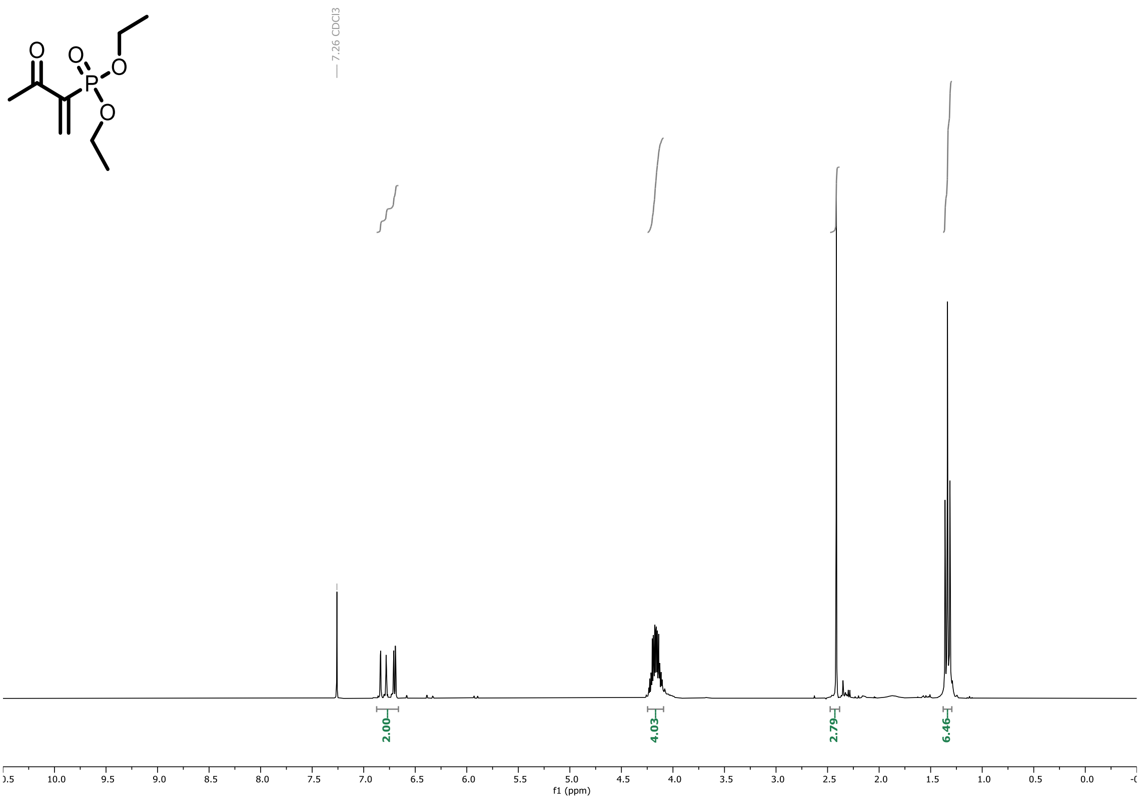

^{13}C NMR (101 MHz, $CDCl_3$) of compound **2'**

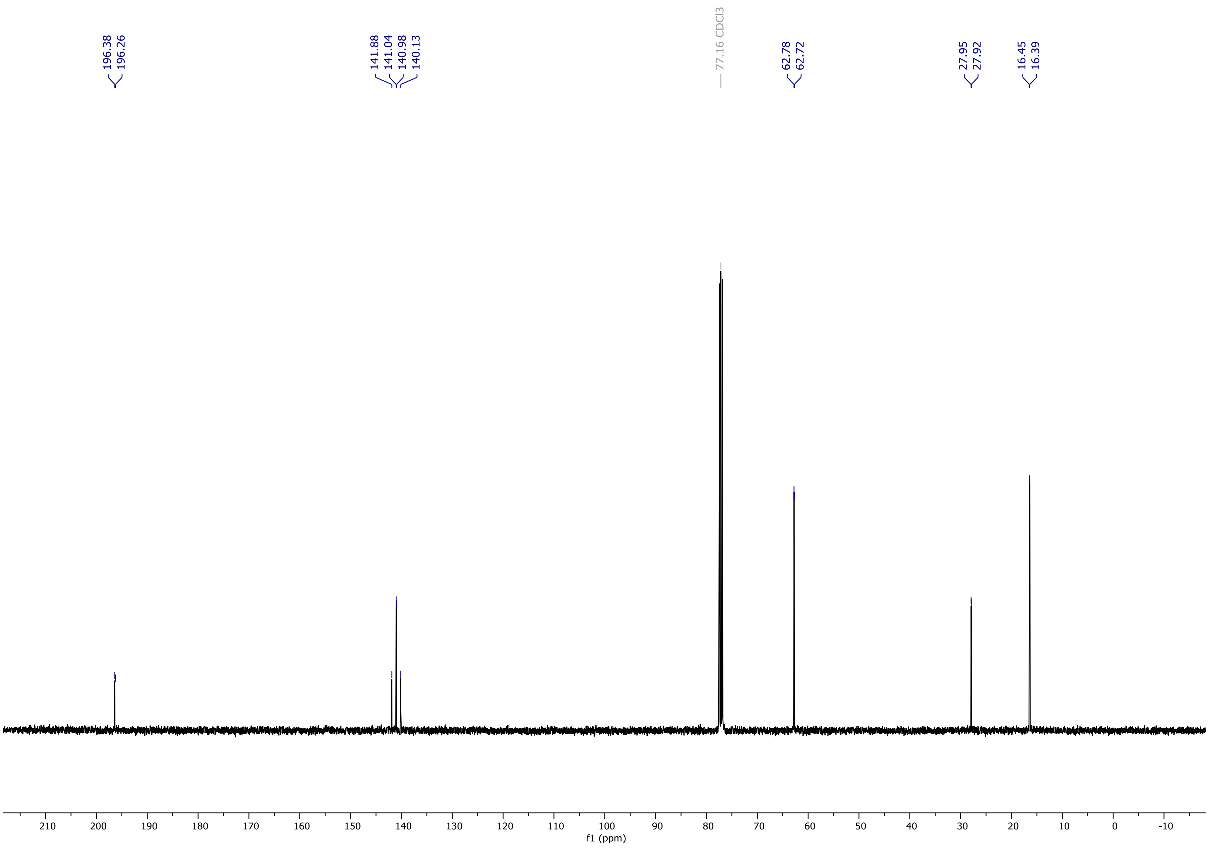

^{31}P NMR (121 MHz, $CDCl_3$) of compound **2''**

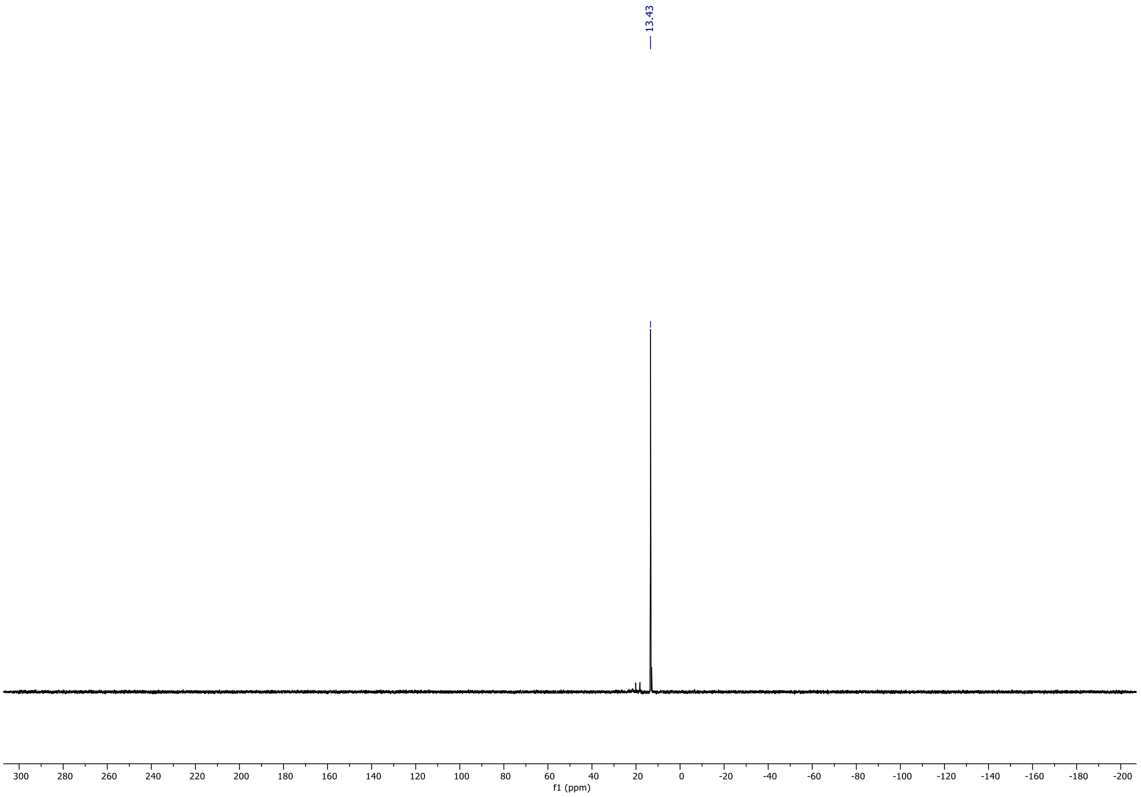

13. NMR Spectra of compounds 3a, 3e, 3f, 3n-q, 3u, 3v

^{1}H NMR (400 MHz, $CDCl_3$) of compound **3a**

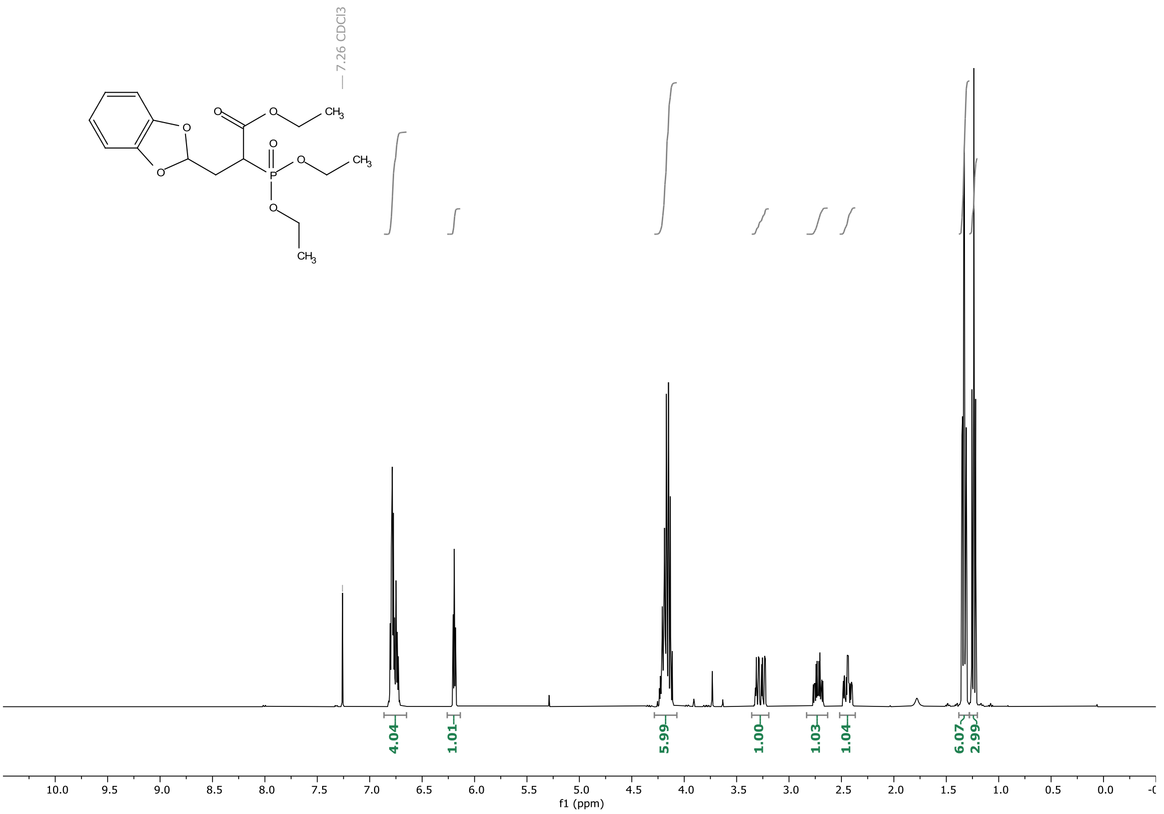

^{13}C NMR (101 MHz, $CDCl_3$) of compound **3a**

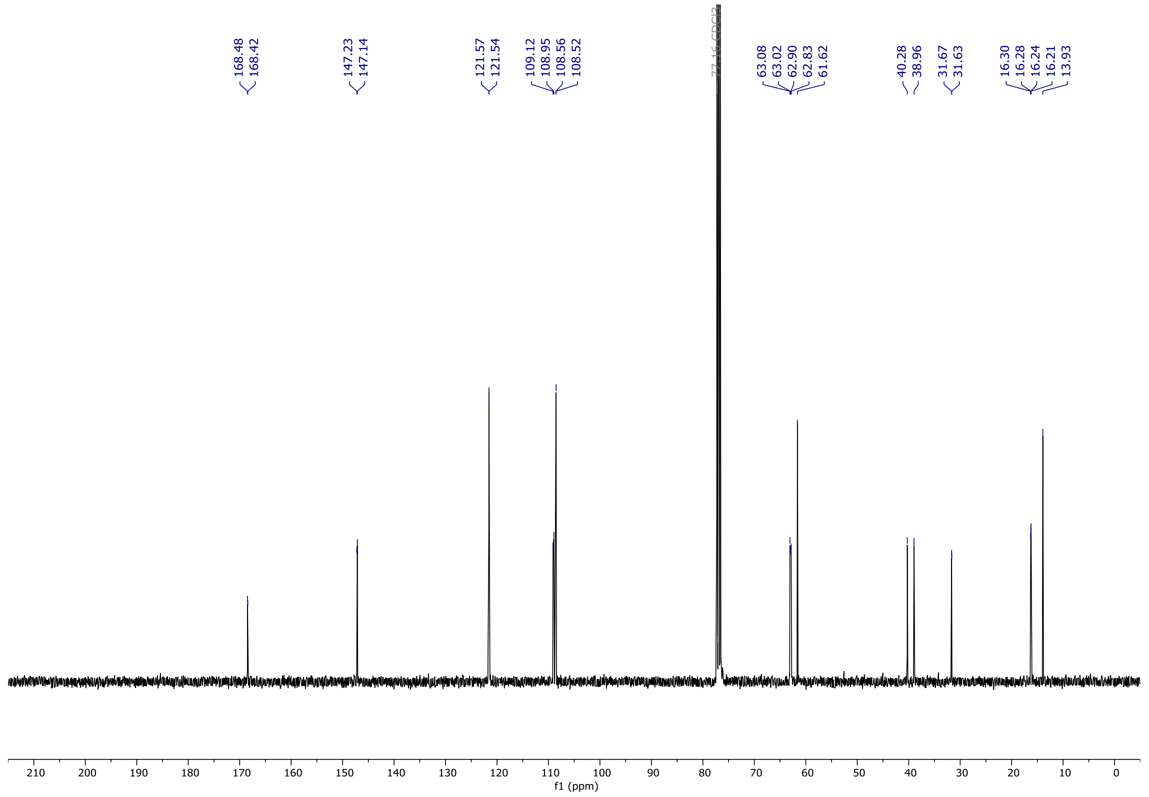

^{31}P NMR (162 MHz, $CDCl_3$) of compound **3a**

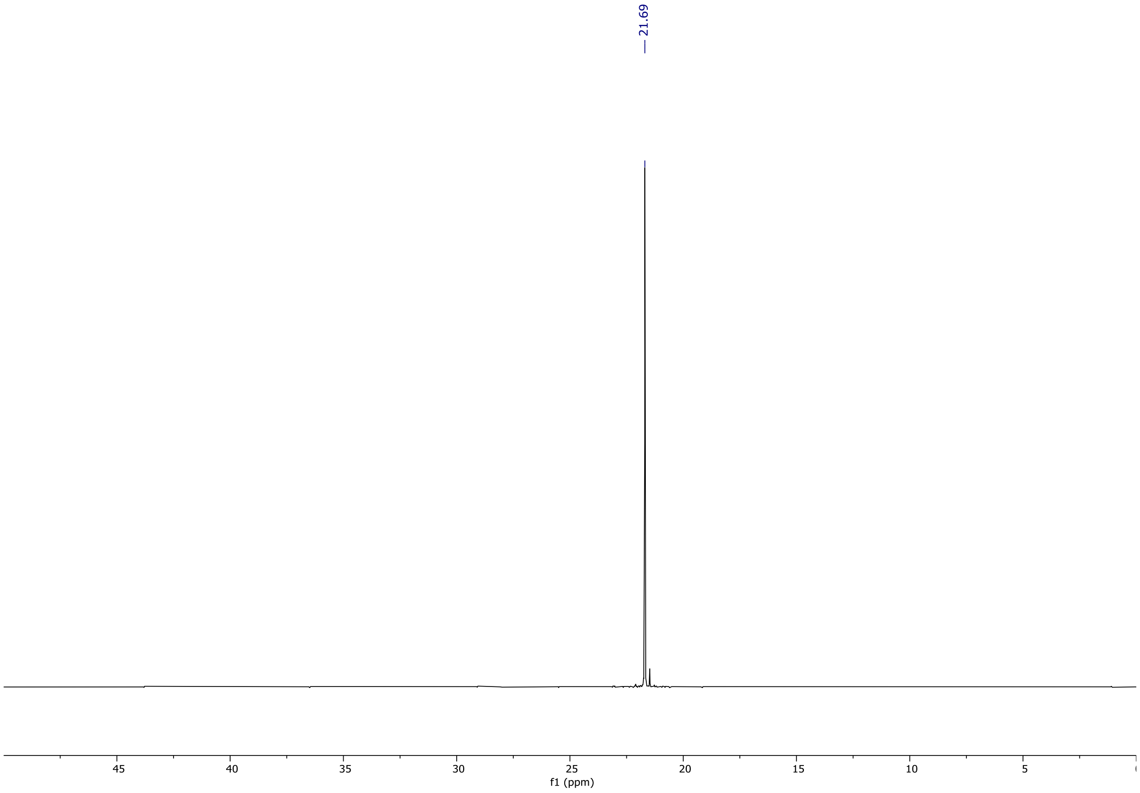

^{1}H NMR (400 MHz, $CDCl_3$) of compound **3e**

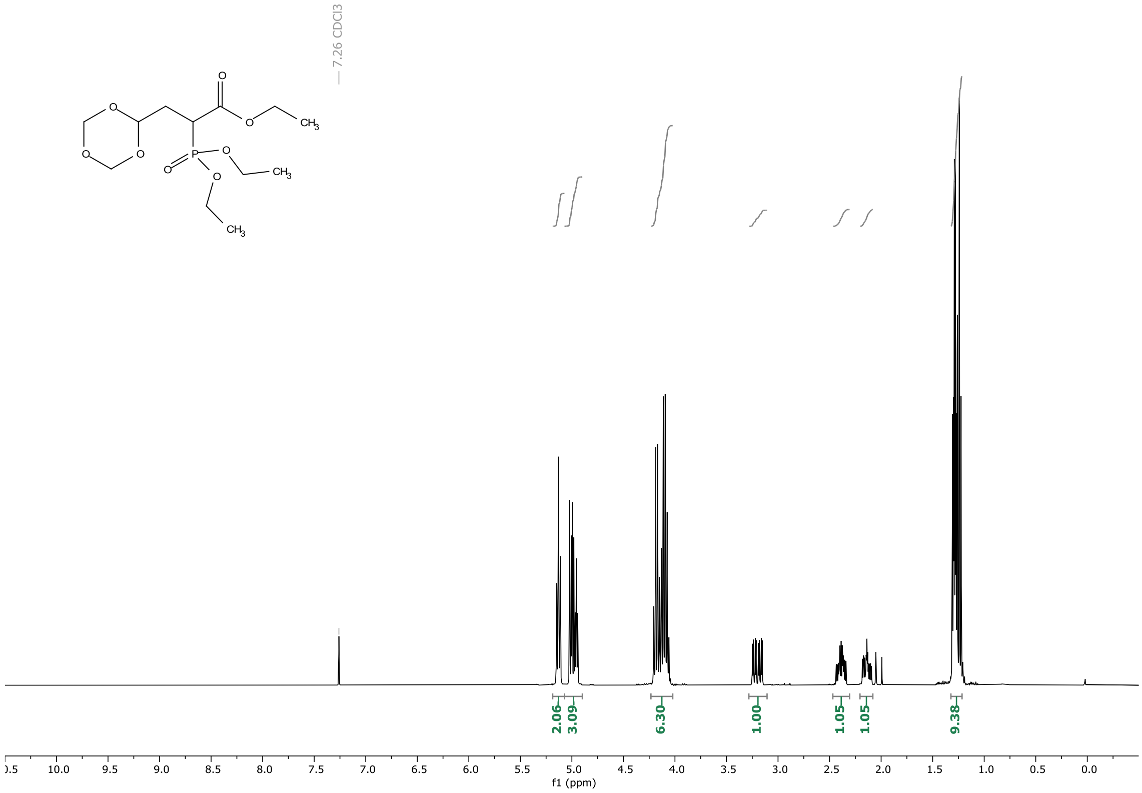

^{13}C NMR (101 MHz, $CDCl_3$) of compound **3e**

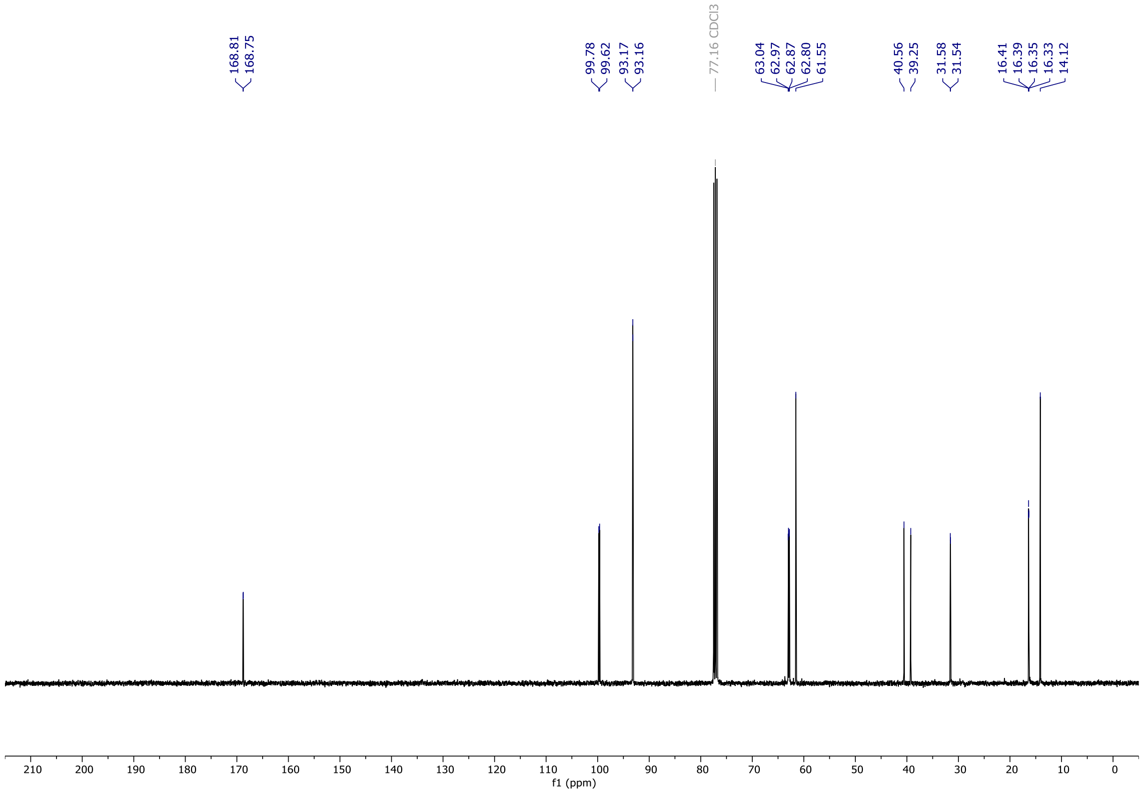

^{31}P NMR (162 MHz, $CDCl_3$) of compound **3e**

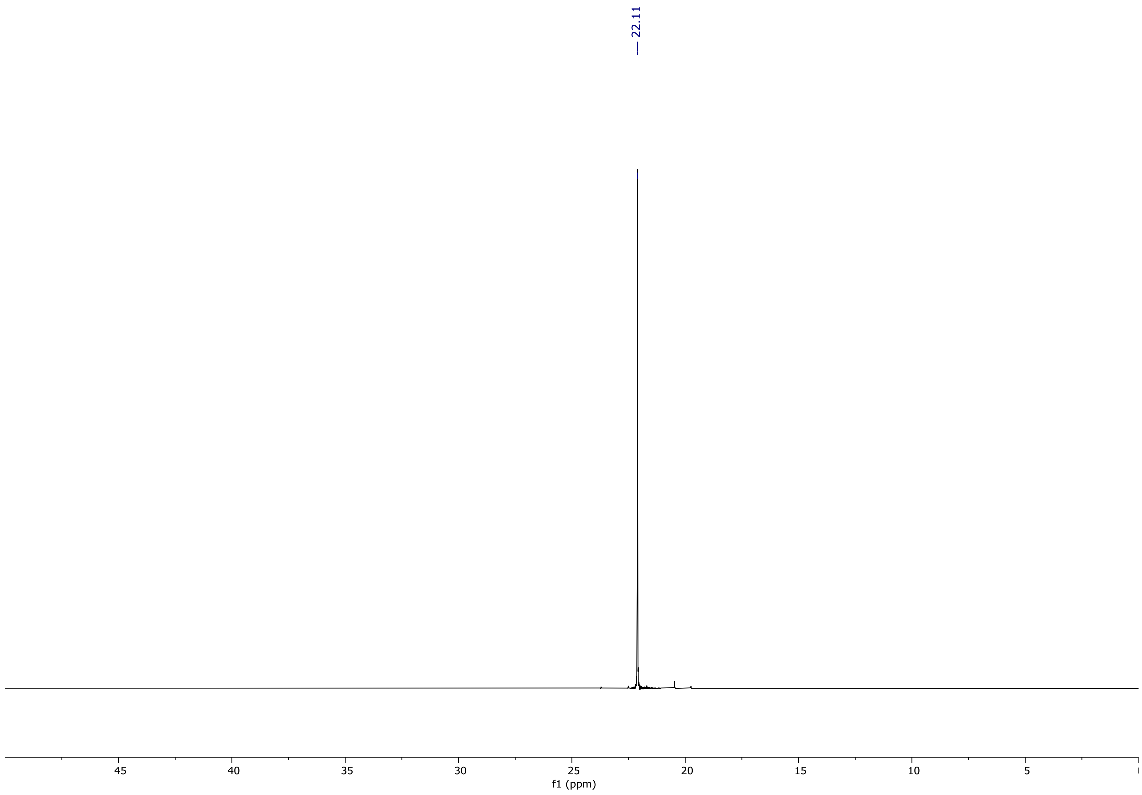

^{1}H NMR (400 MHz, $CDCl_3$) of compound **3f**

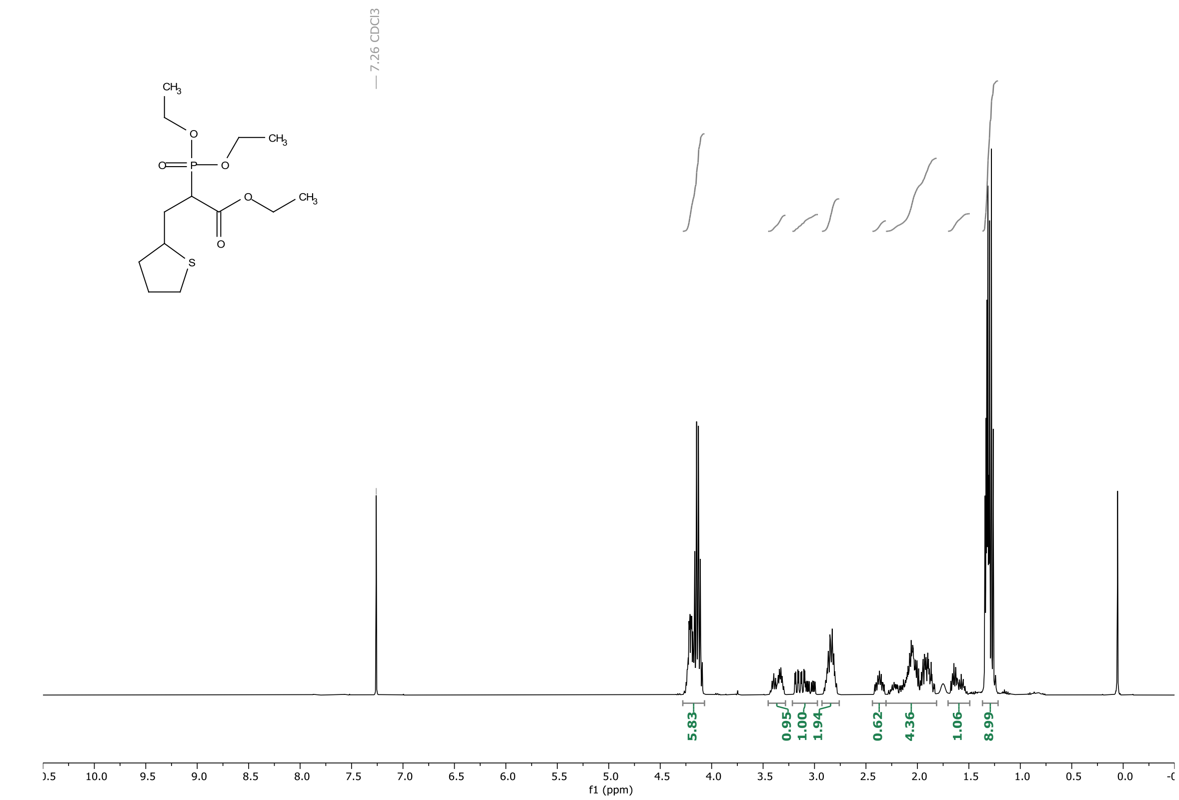

^{13}C NMR (101 MHz, $CDCl_3$) of compound **3f**

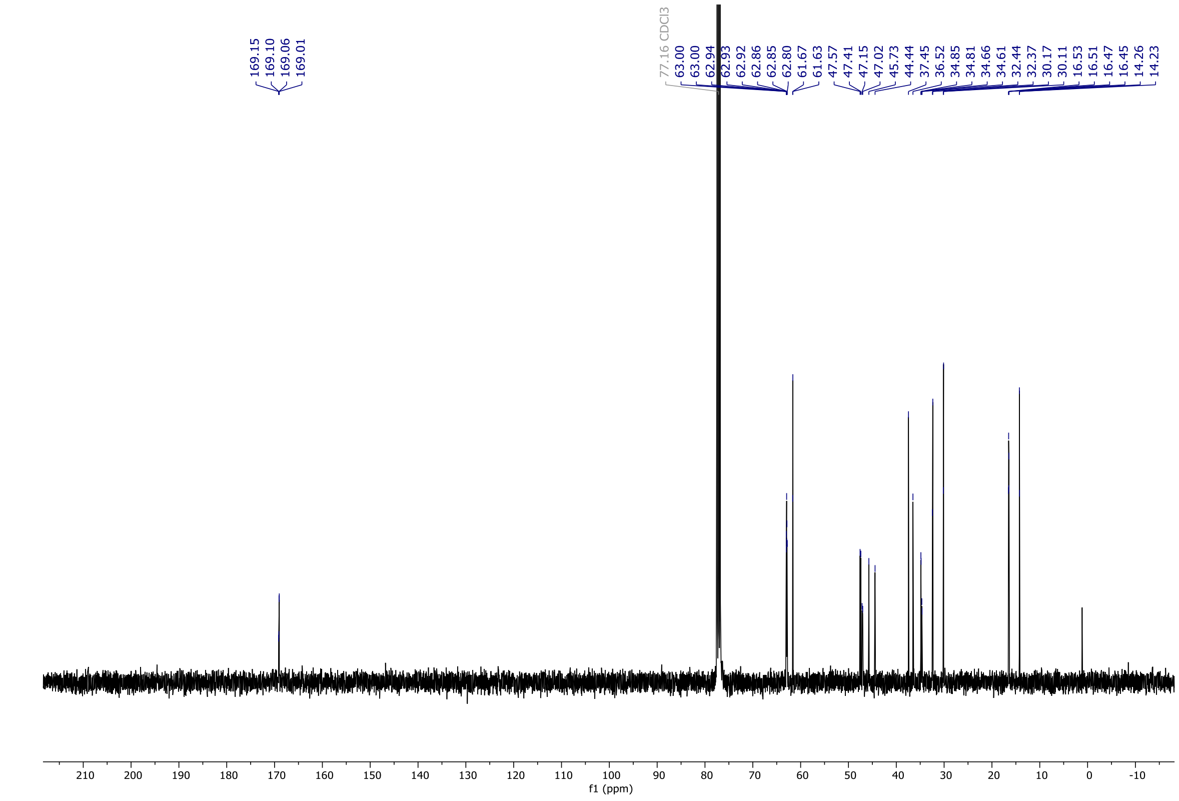

^{31}P NMR (162 MHz, $CDCl_3$) of compound **3f**

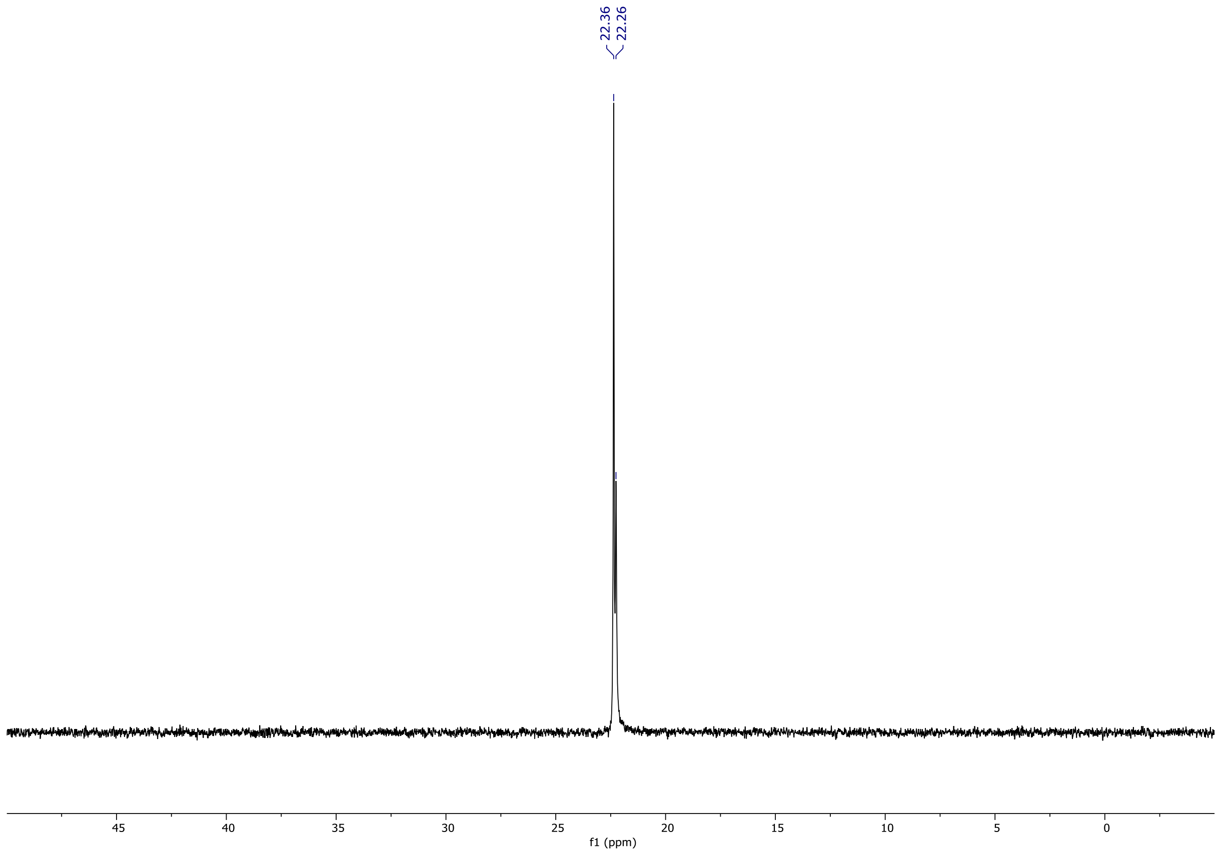

^{1}H NMR (400 MHz, $CDCl_3$) of compound **3n**

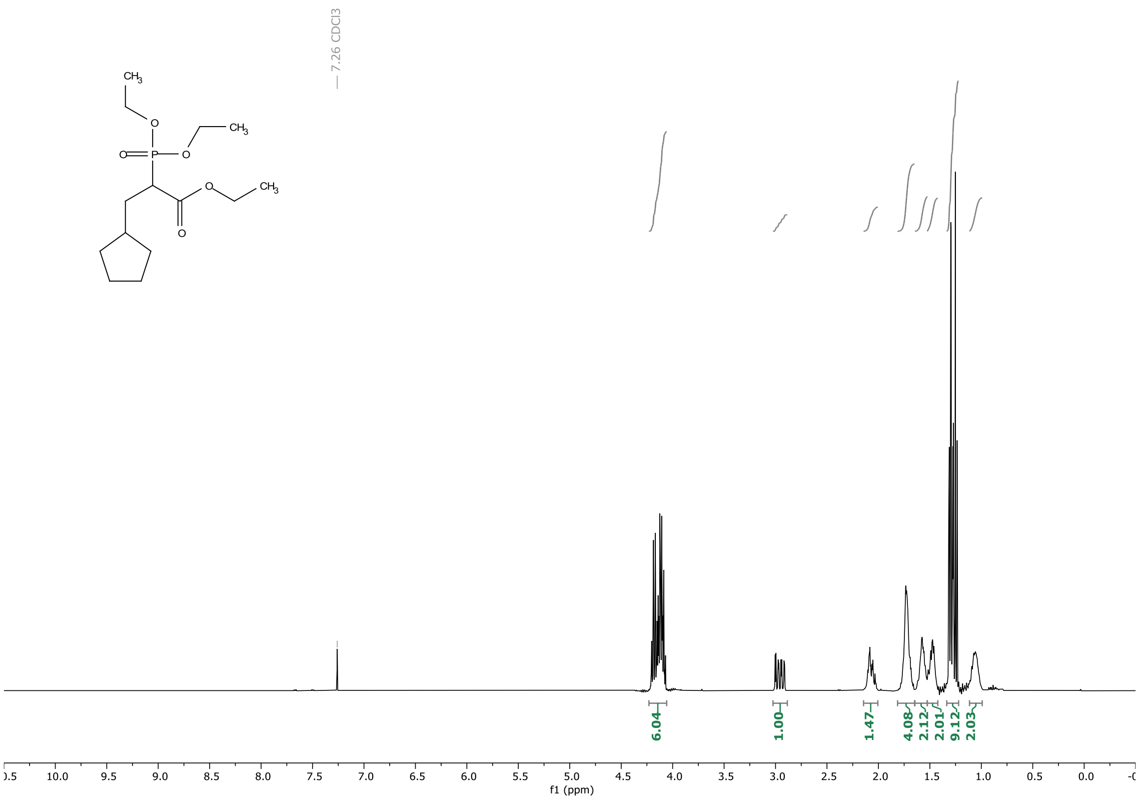

^{13}C NMR (101 MHz, $CDCl_3$) of compound **3n**

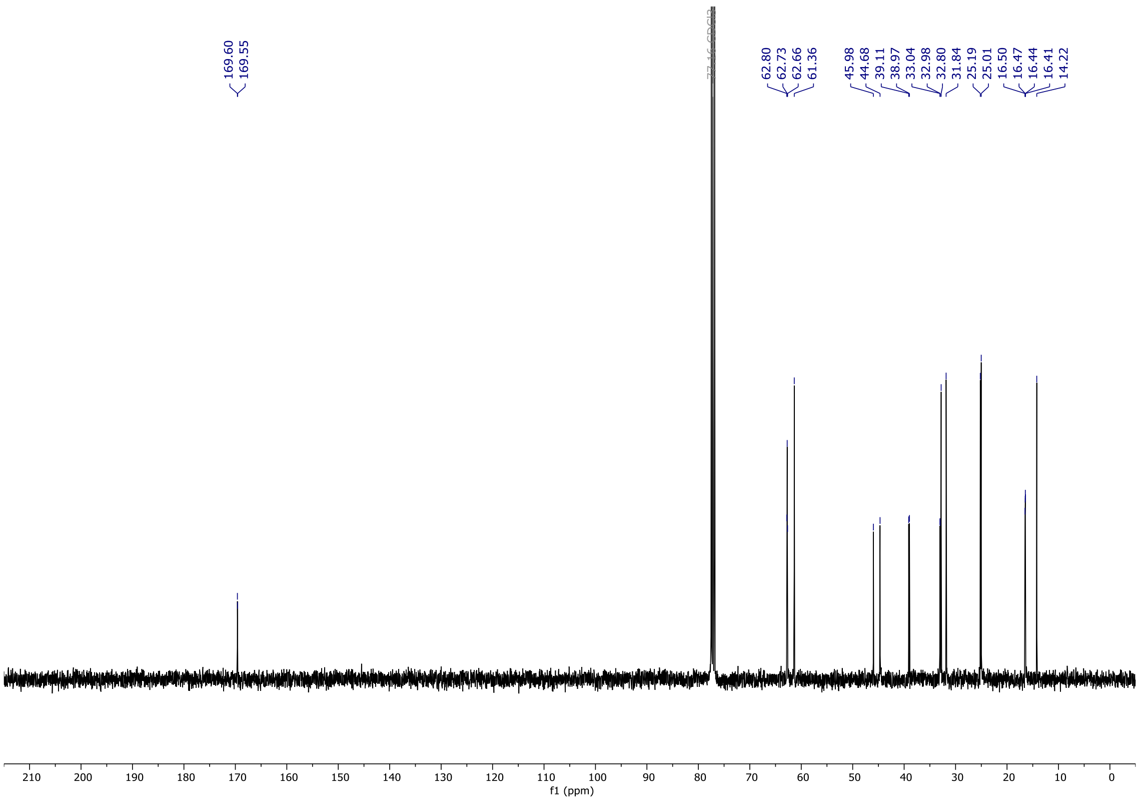

^{31}P NMR (162 MHz, $CDCl_3$) of compound **3n**

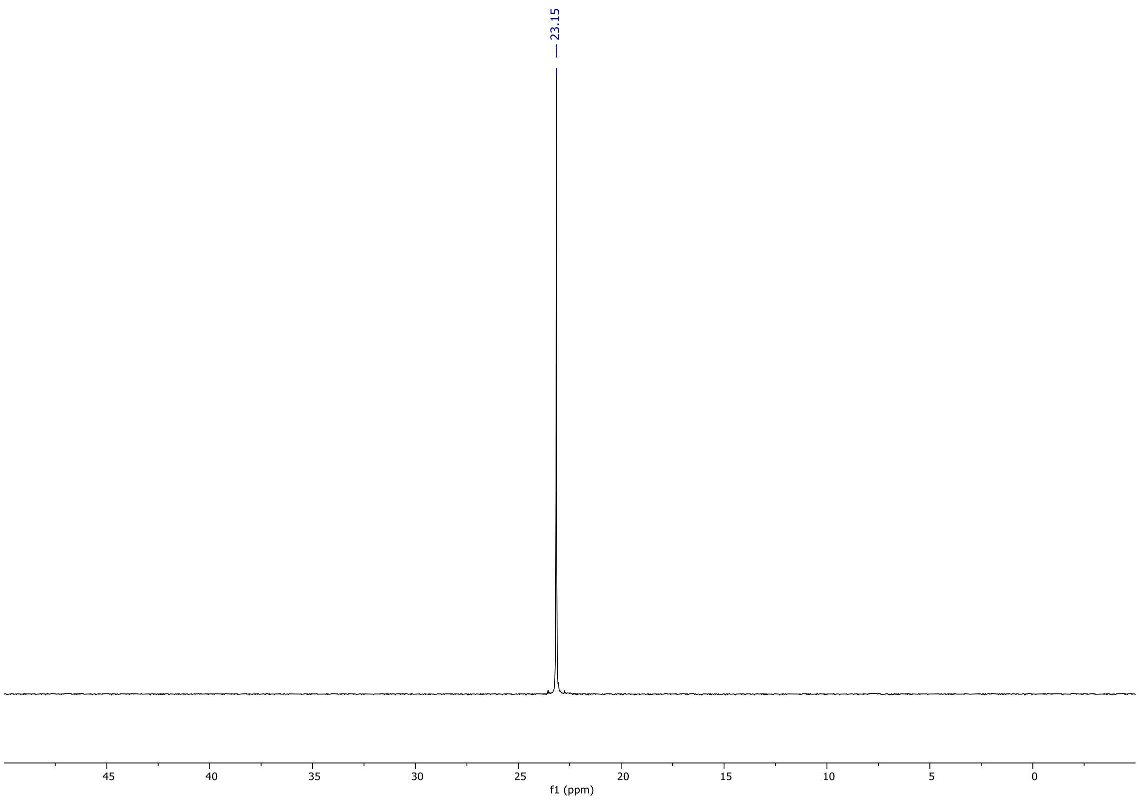

^{1}H NMR (400 MHz, $CDCl_3$) of compound **3o**

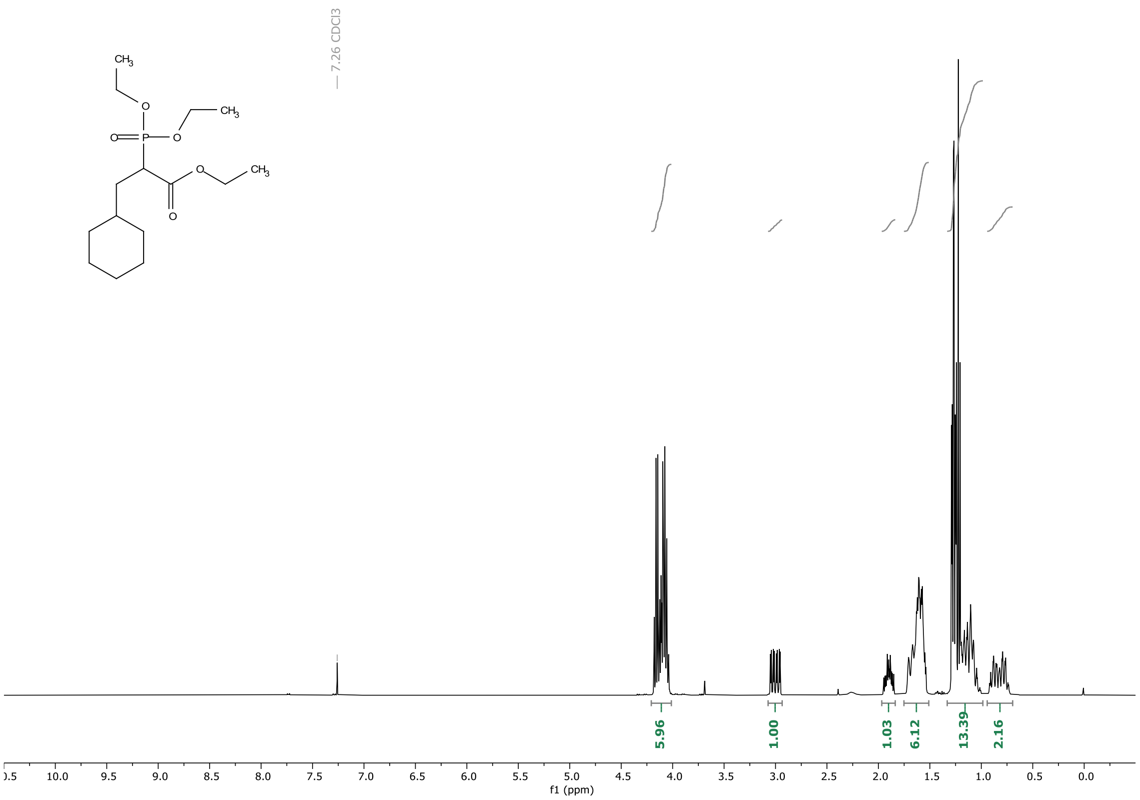

^{13}C NMR (101 MHz, $CDCl_3$) of compound **3o**

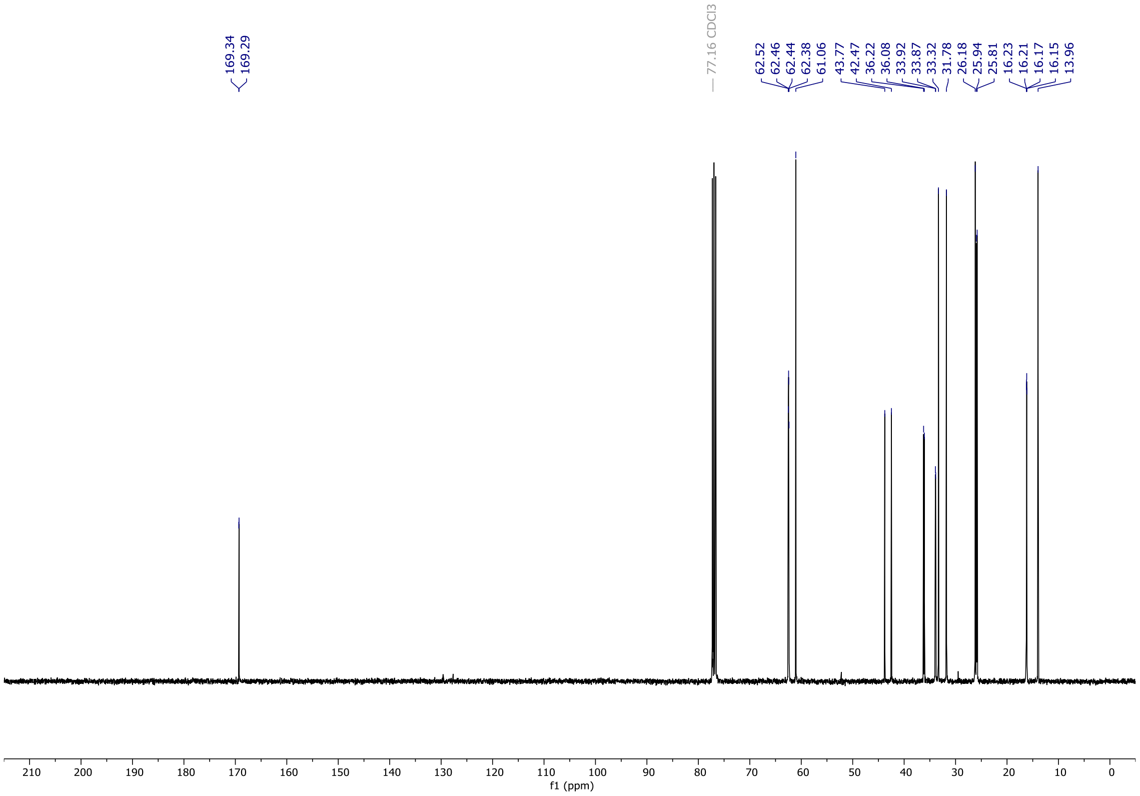

^{31}P NMR (162 MHz, $CDCl_3$) of compound **3o**

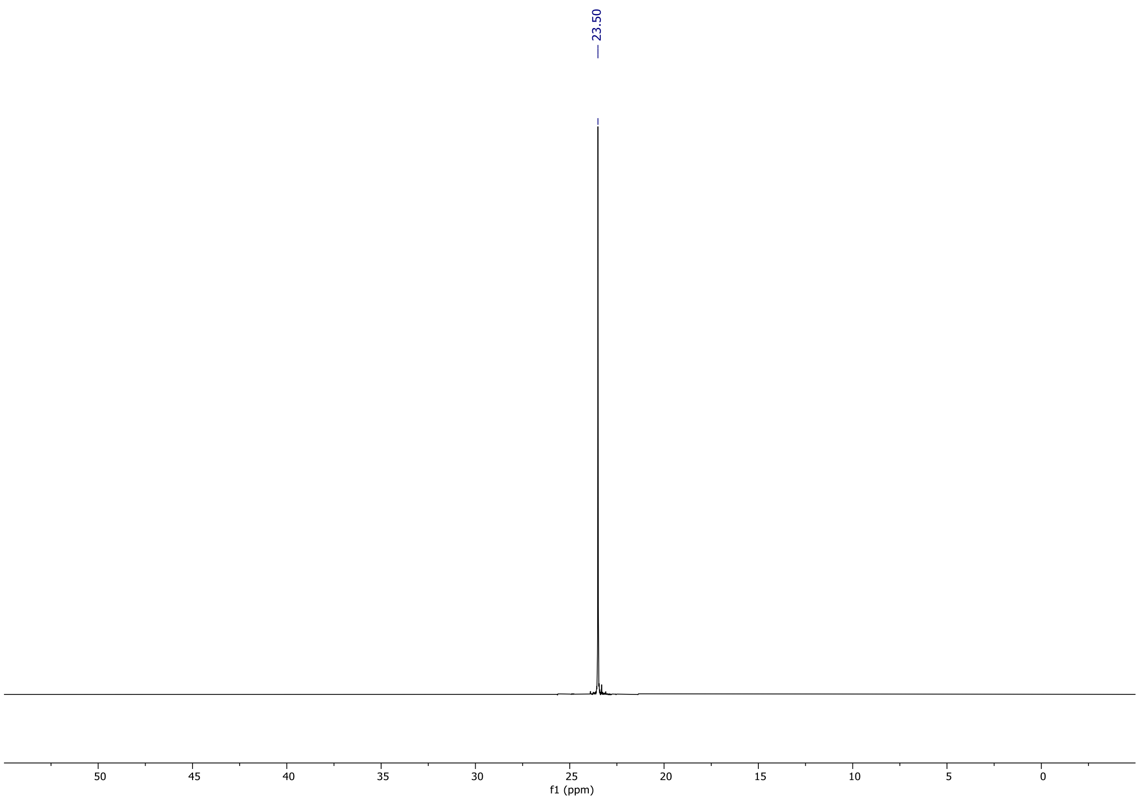

^{1}H NMR (400 MHz, $CDCl_3$) of compound **3p**

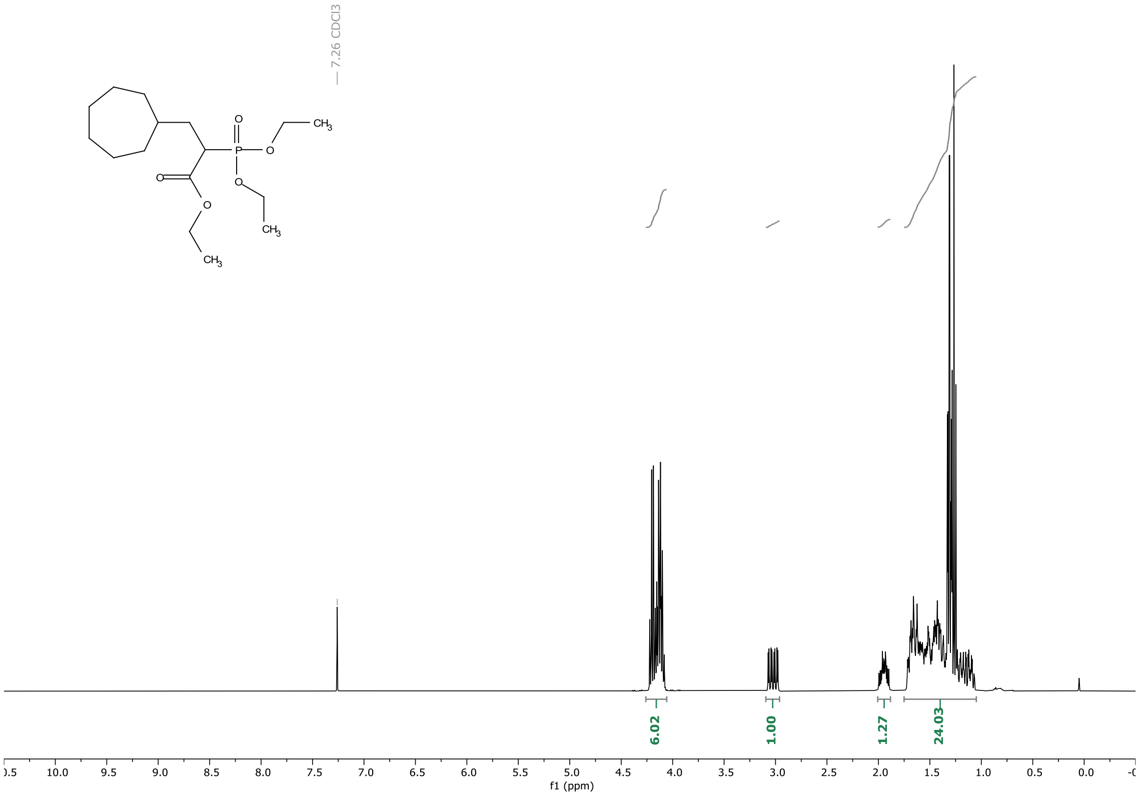

^{13}C NMR (101 MHz, $CDCl_3$) of compound **3p**

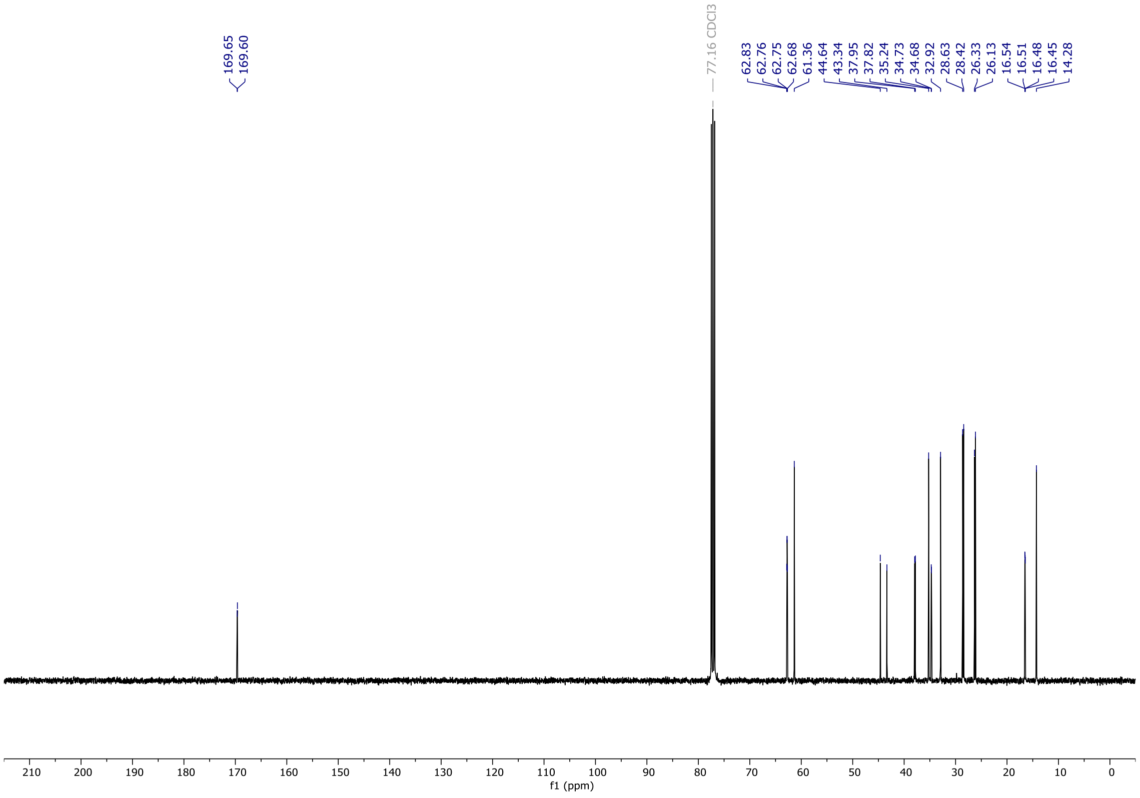

^{31}P NMR (162 MHz, $CDCl_3$) of compound **3p**

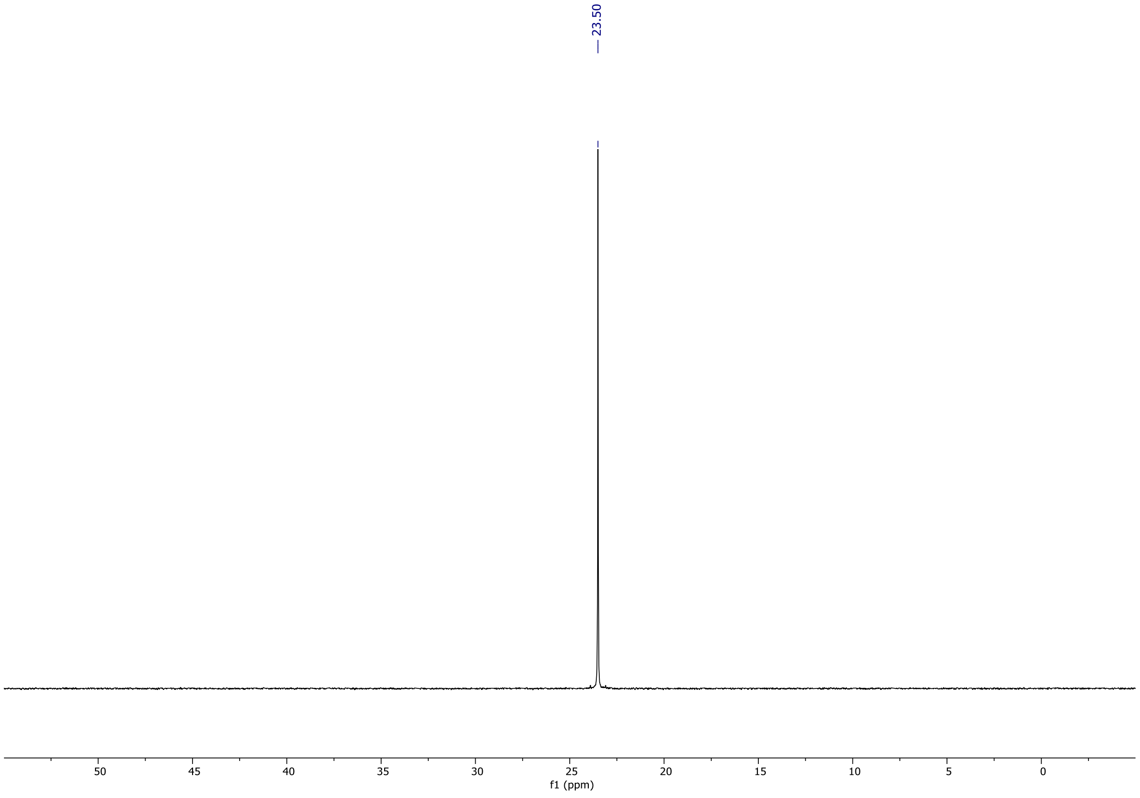

^{1}H NMR (400 MHz, $CDCl_3$) of compound **3q**

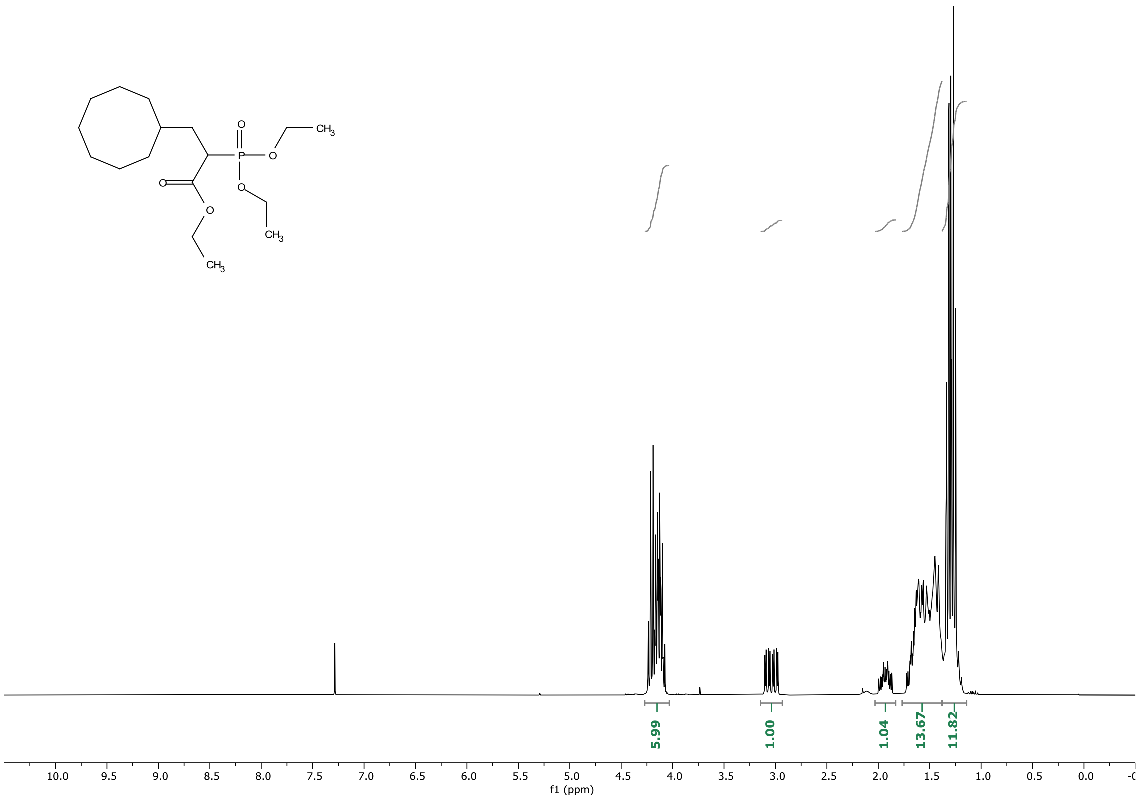

^{13}C NMR (101 MHz, $CDCl_3$) of compound **3q**

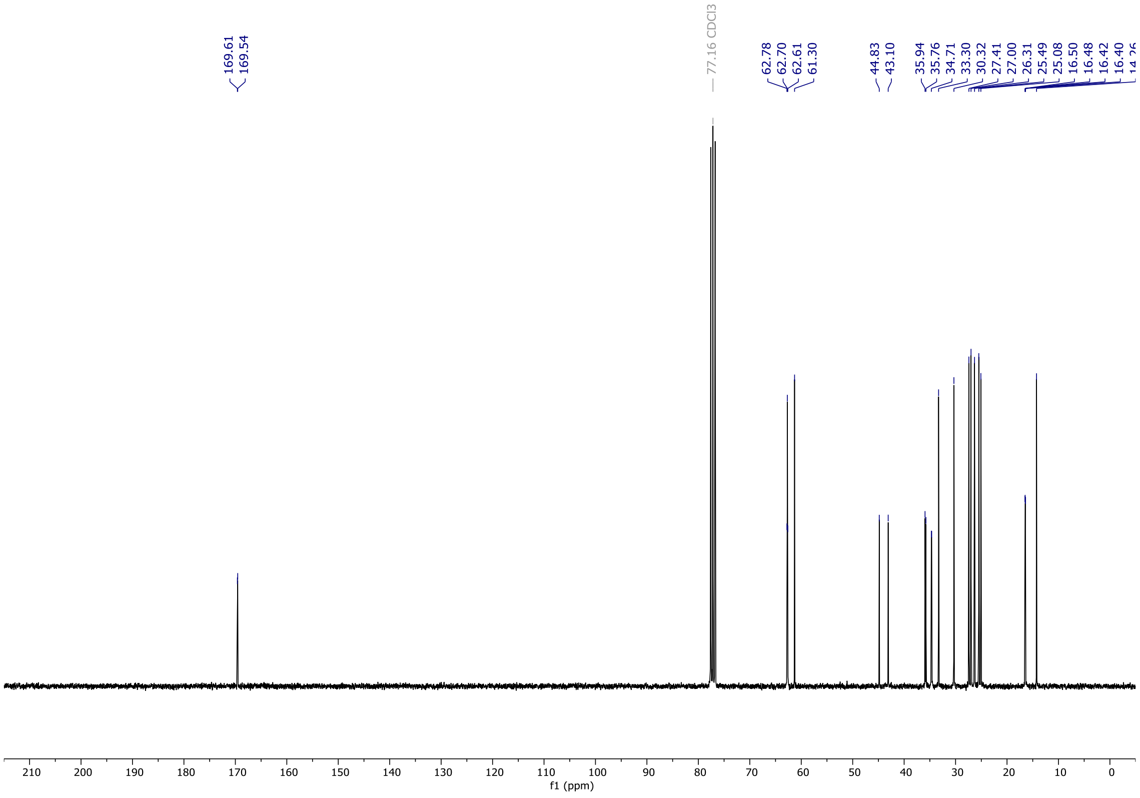

^{31}P NMR (162 MHz, $CDCl_3$) of compound **3q**

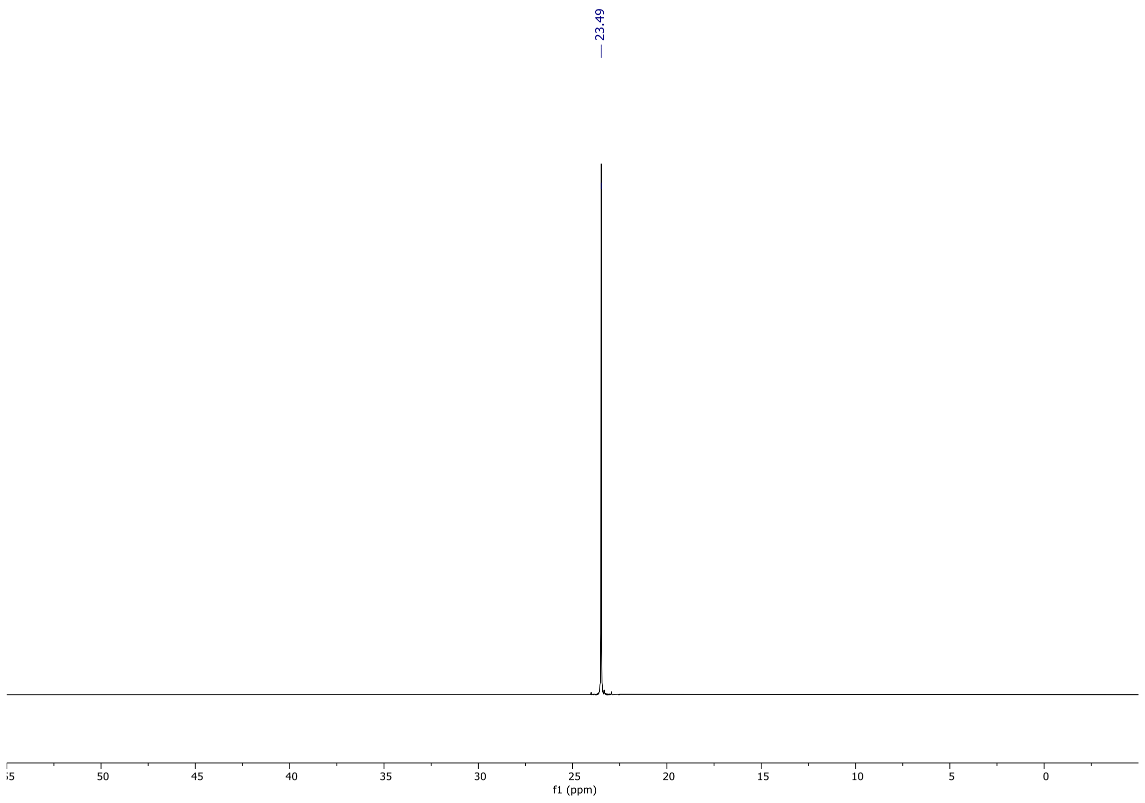

^{1}H NMR (400 MHz, CDCl3) of compound **3u**

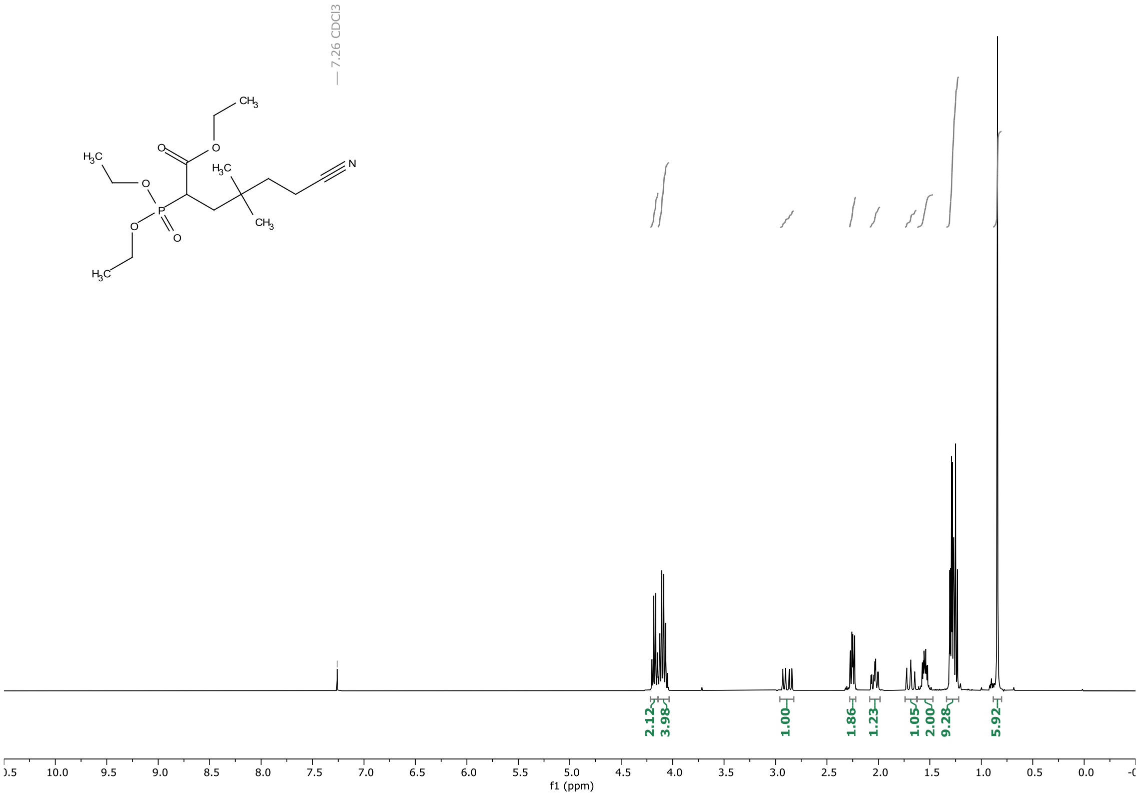

^{13}C NMR (101 MHz, CDCl3) of compound **3u**

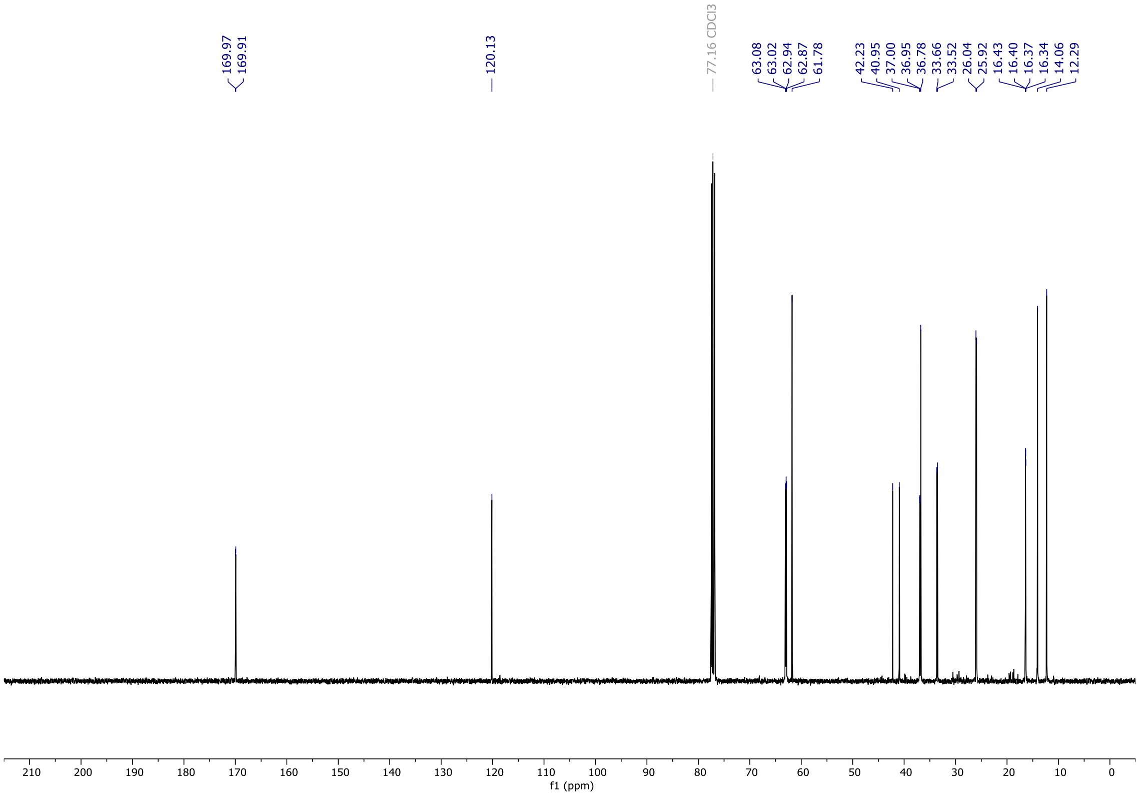

^{31}P NMR (162 MHz, $CDCl_3$) of compound **3u**

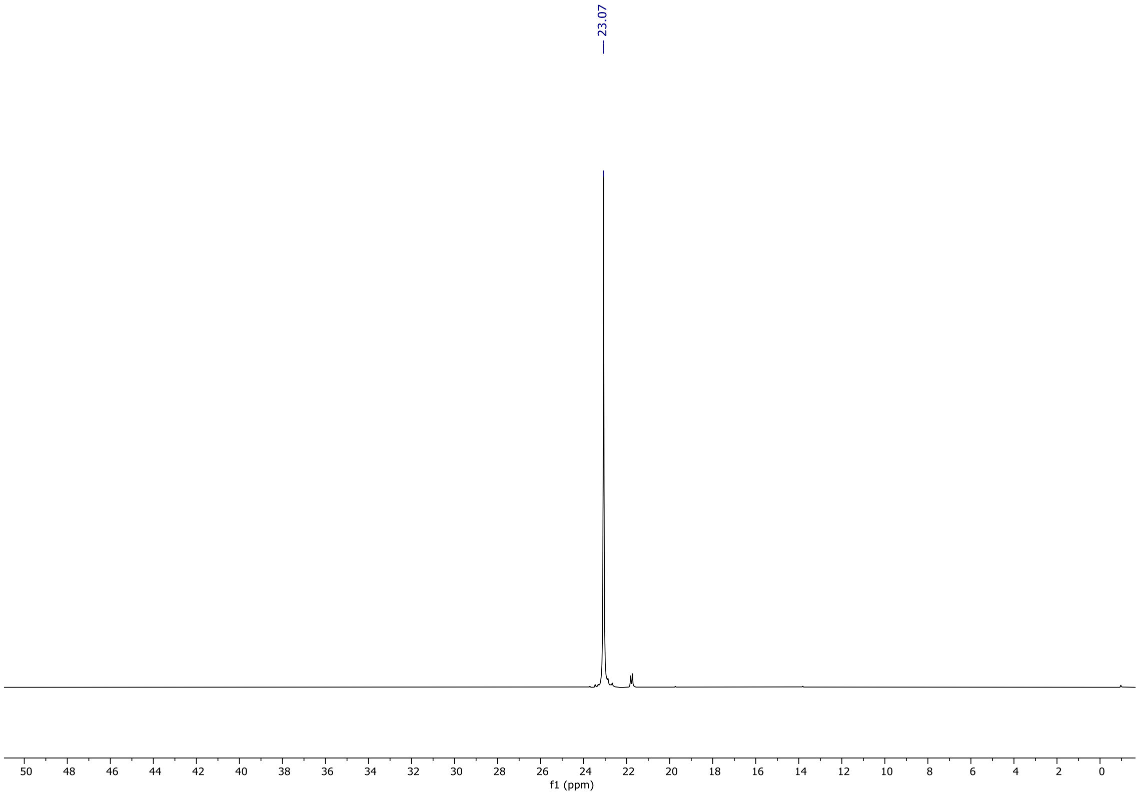

^{1}H NMR (400 MHz, $CDCl_3$) of compound **3v**

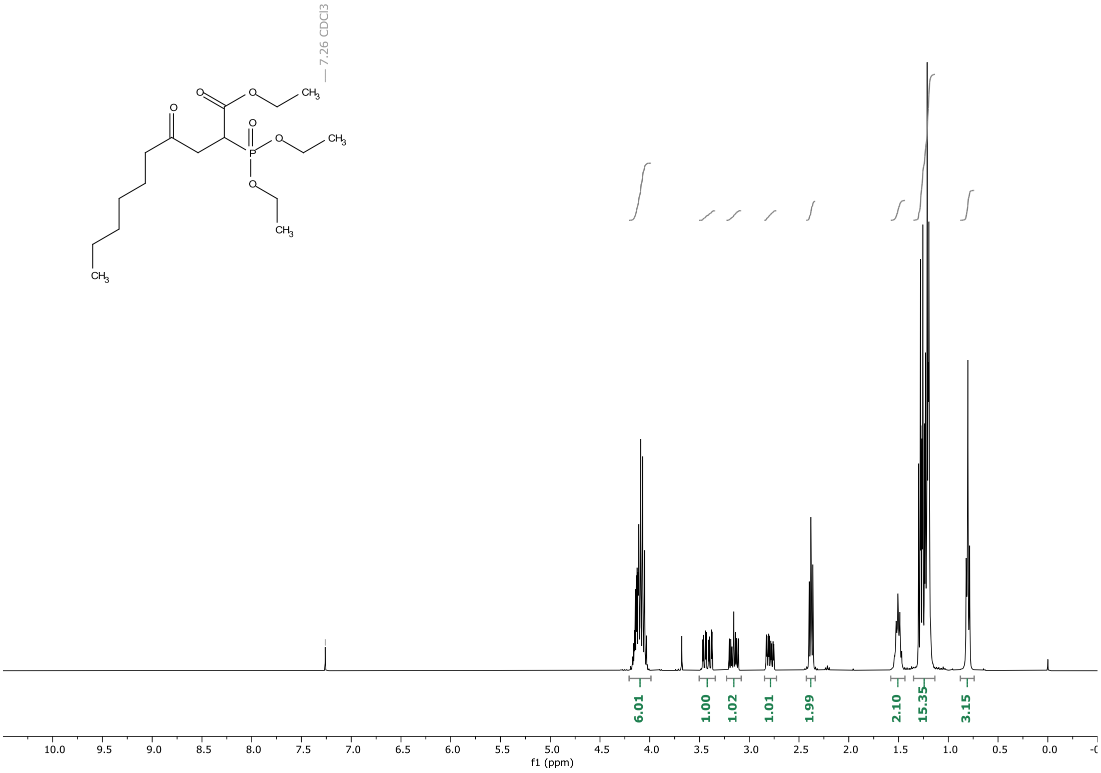

^{13}C NMR (101 MHz, $CDCl_3$) of compound **3v**

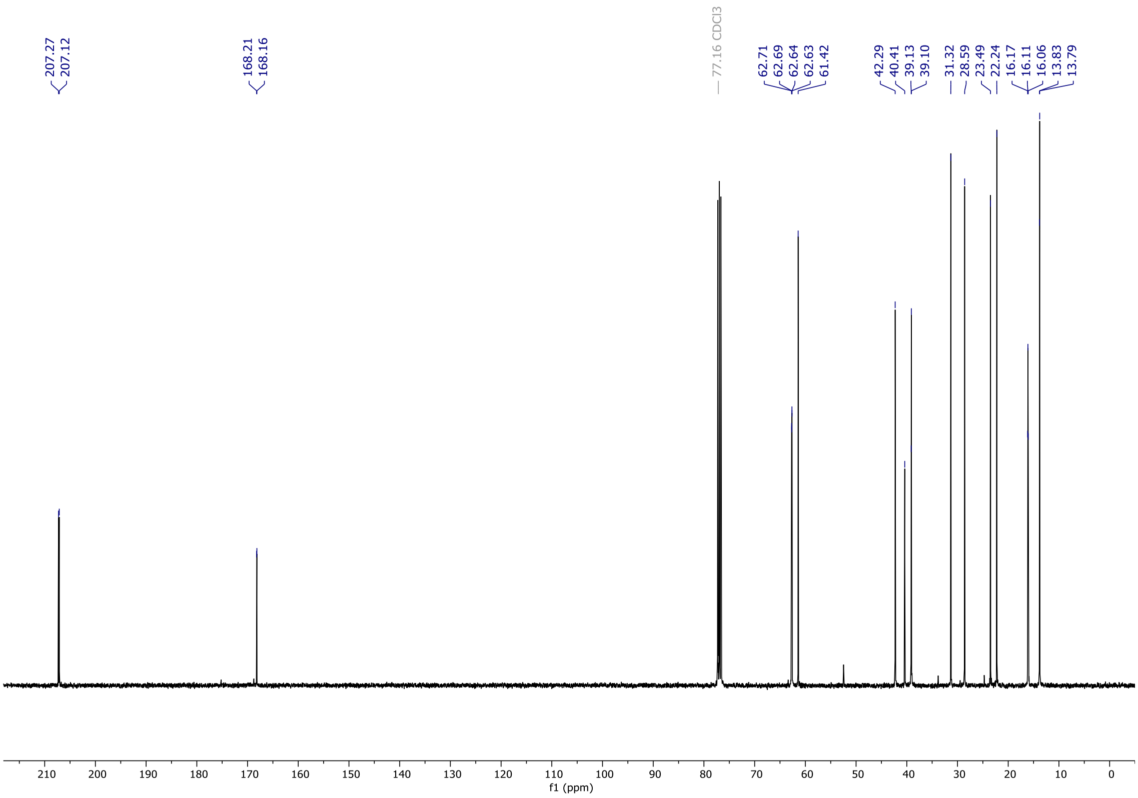

^{31}P NMR (162 MHz, $CDCl_3$) of compound **3v**

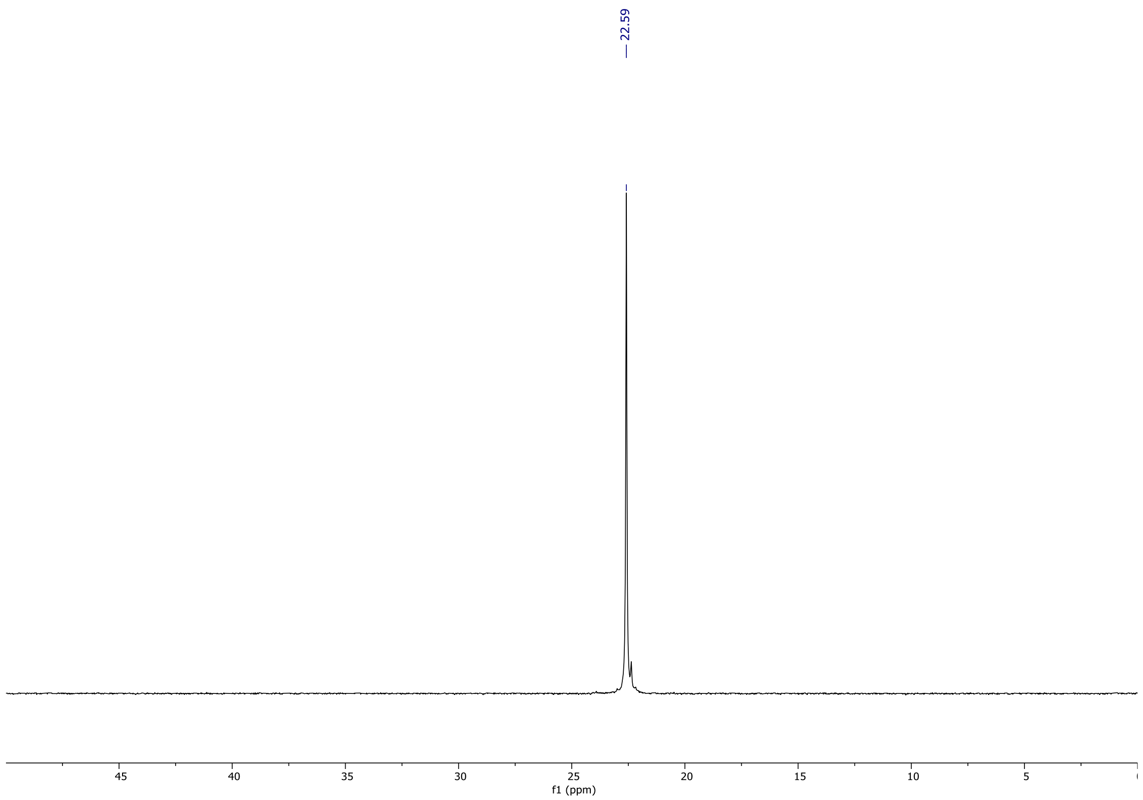

14. NMR Spectra of compounds 4-23

^{1}H NMR (400 MHz, $CDCl_3$) of compound **4**

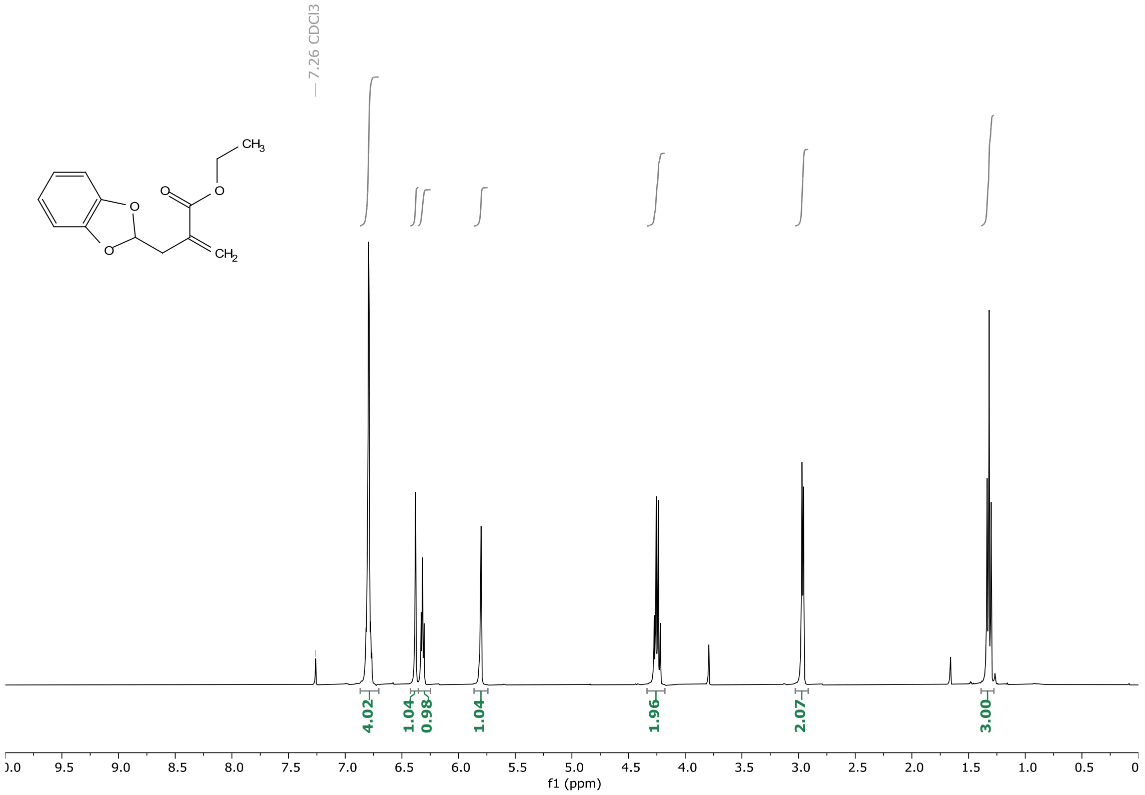

^{13}C NMR (101 MHz, $CDCl_3$) of compound **4**

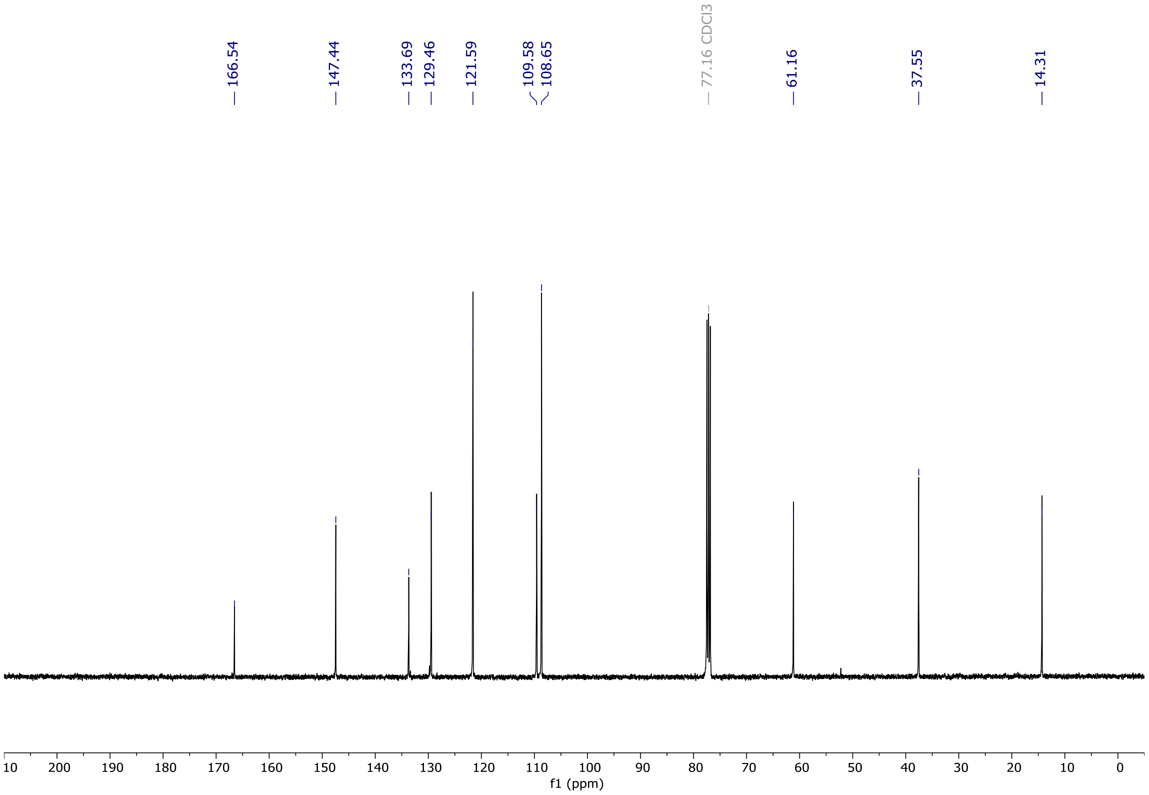

^{1}H NMR (400 MHz, $CDCl_3$) of compound **5**

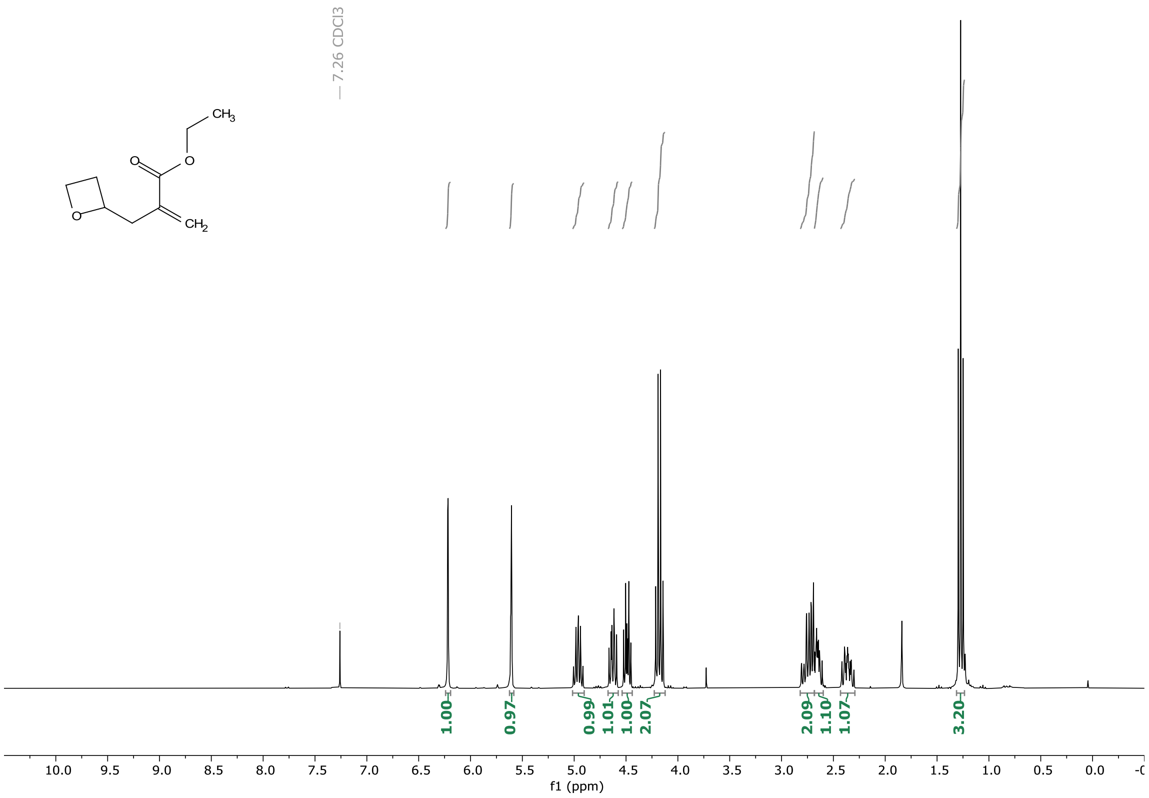

^{13}C NMR (101 MHz, $CDCl_3$) of compound **5**

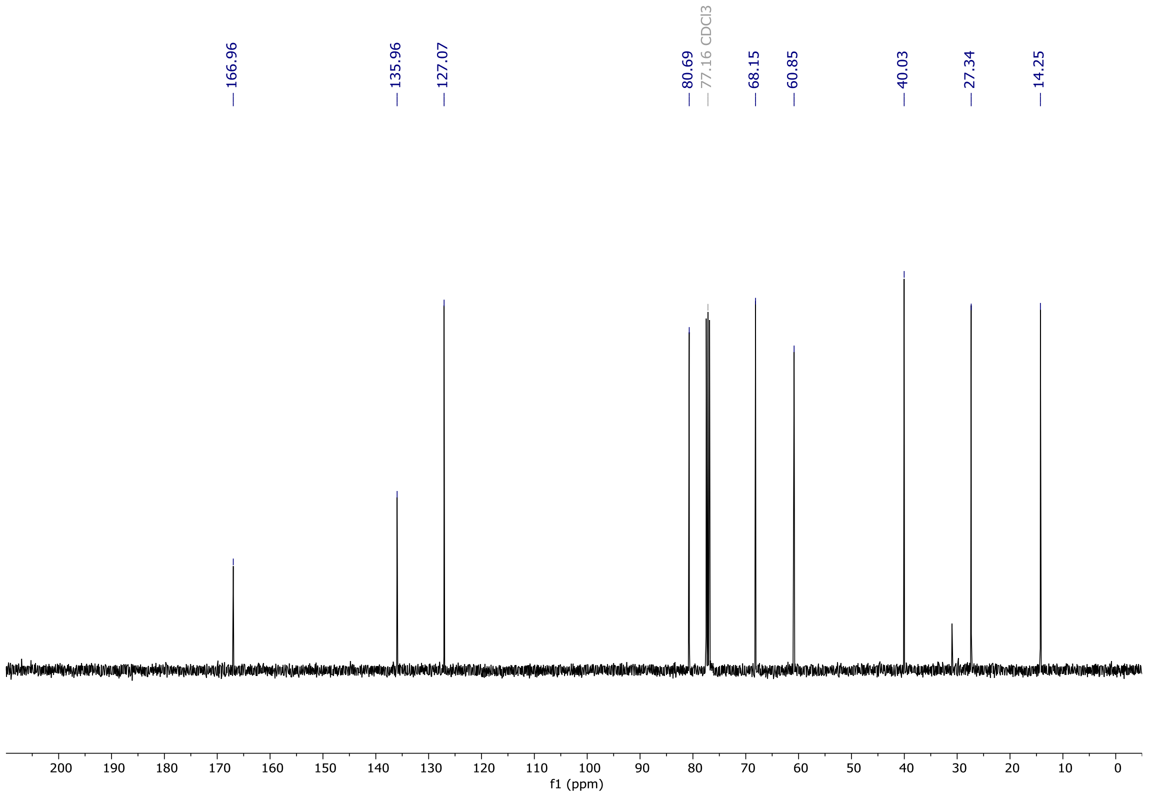

^{1}H NMR (400 MHz, $CDCl_3$) compound **6**

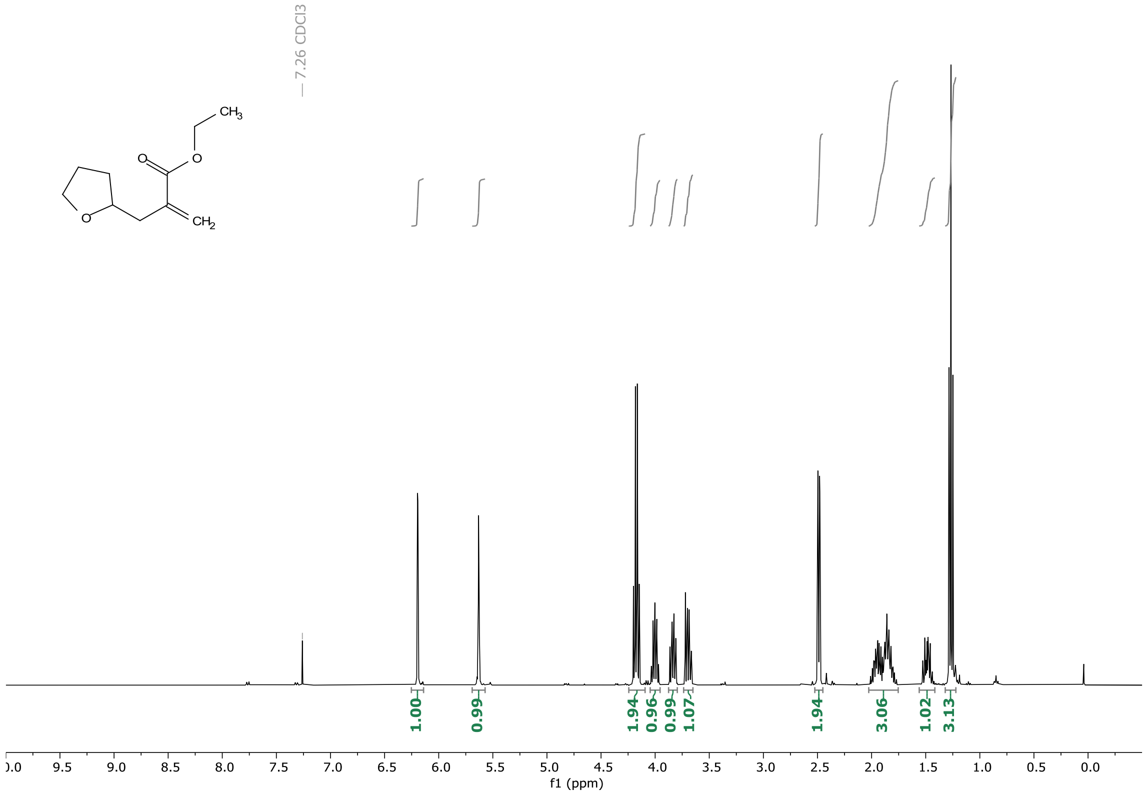

^{13}C NMR (101 MHz, $CDCl_3$) compound **6**

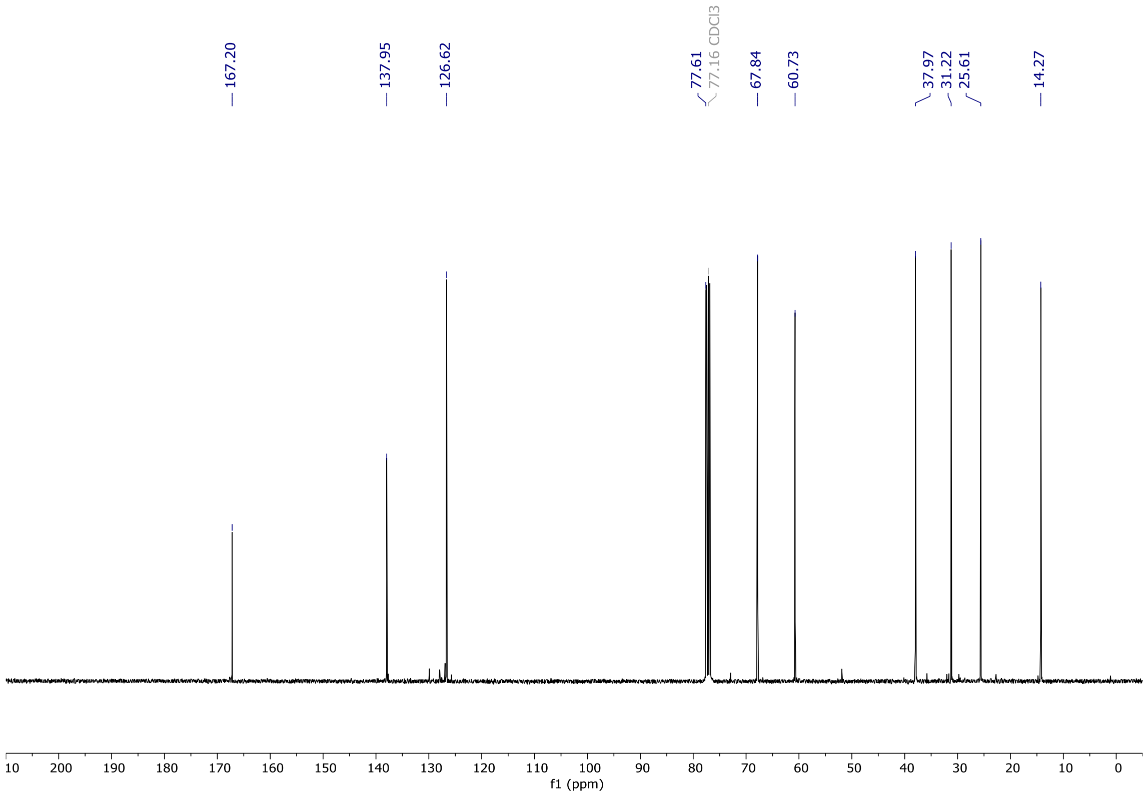

^{1}H NMR (400 MHz, $CDCl_3$) of compound **7**

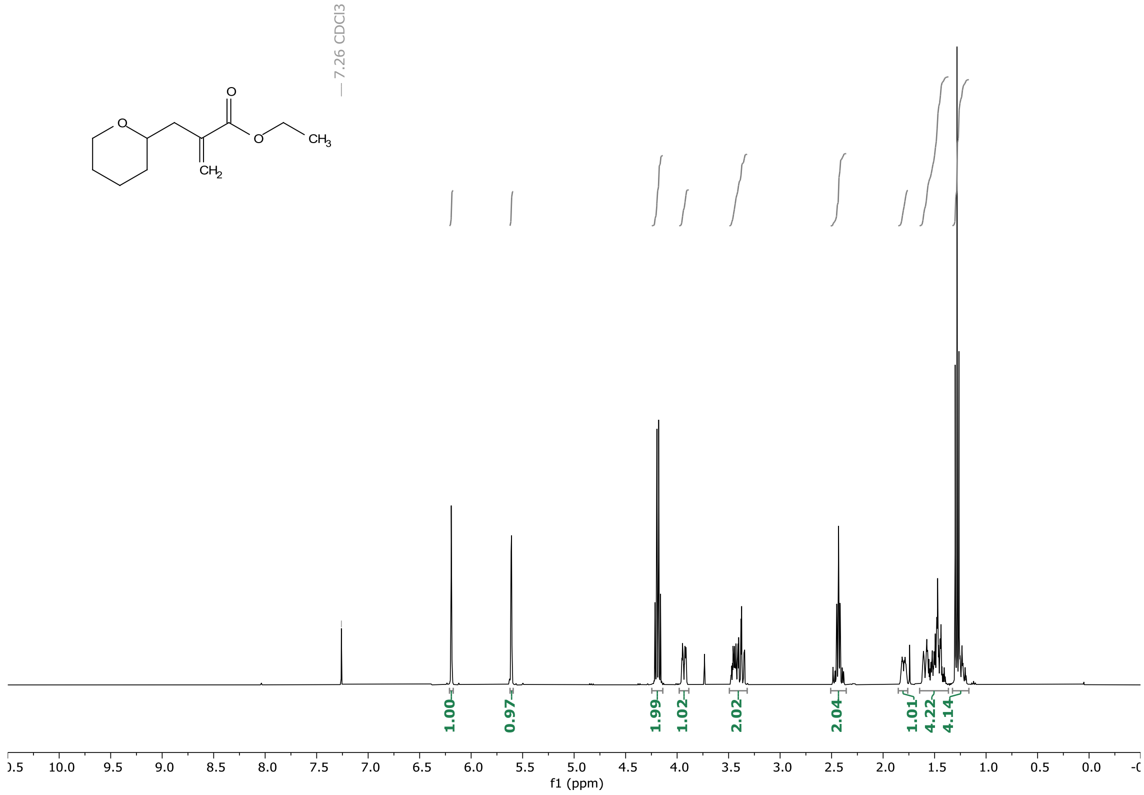

^{13}C NMR (101 MHz, $CDCl_3$) of compound **7**

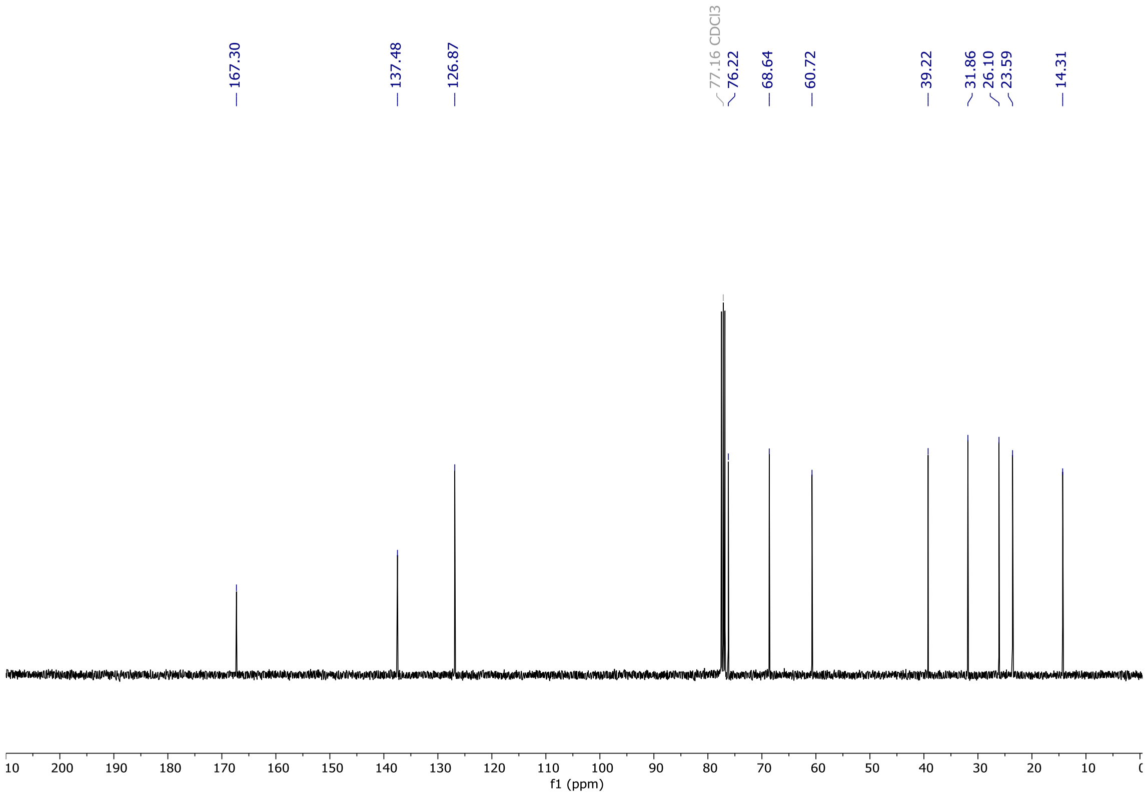

^{1}H NMR (400 MHz, $CDCl_3$) of compound **8**

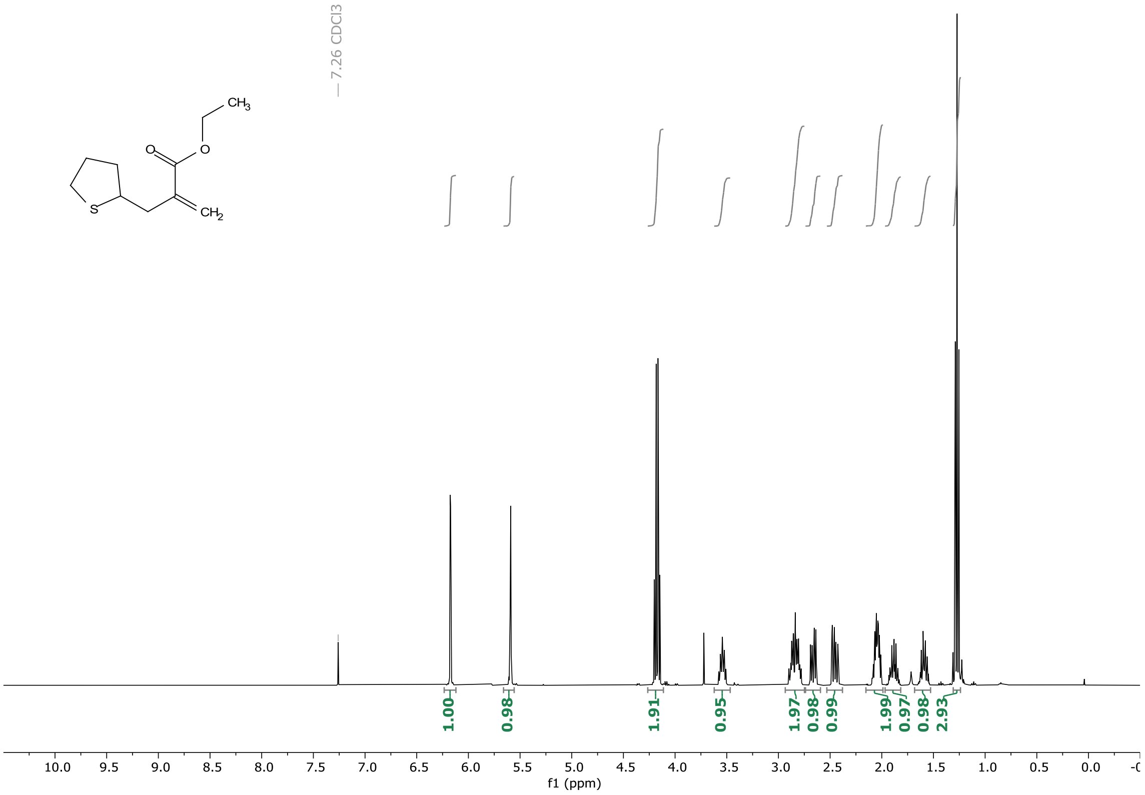

^{13}C NMR (101 MHz, $CDCl_3$) of compound **8**

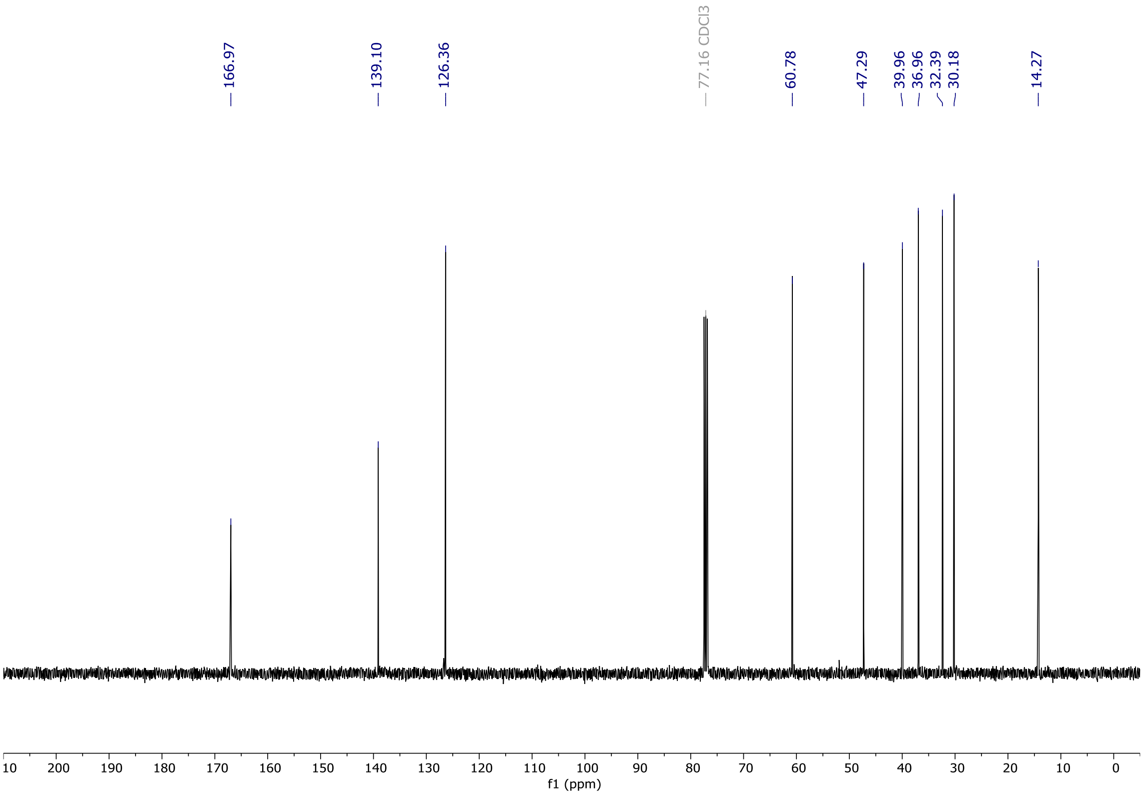

^{1}H NMR (400 MHz, $CDCl_3$) of compound **9**

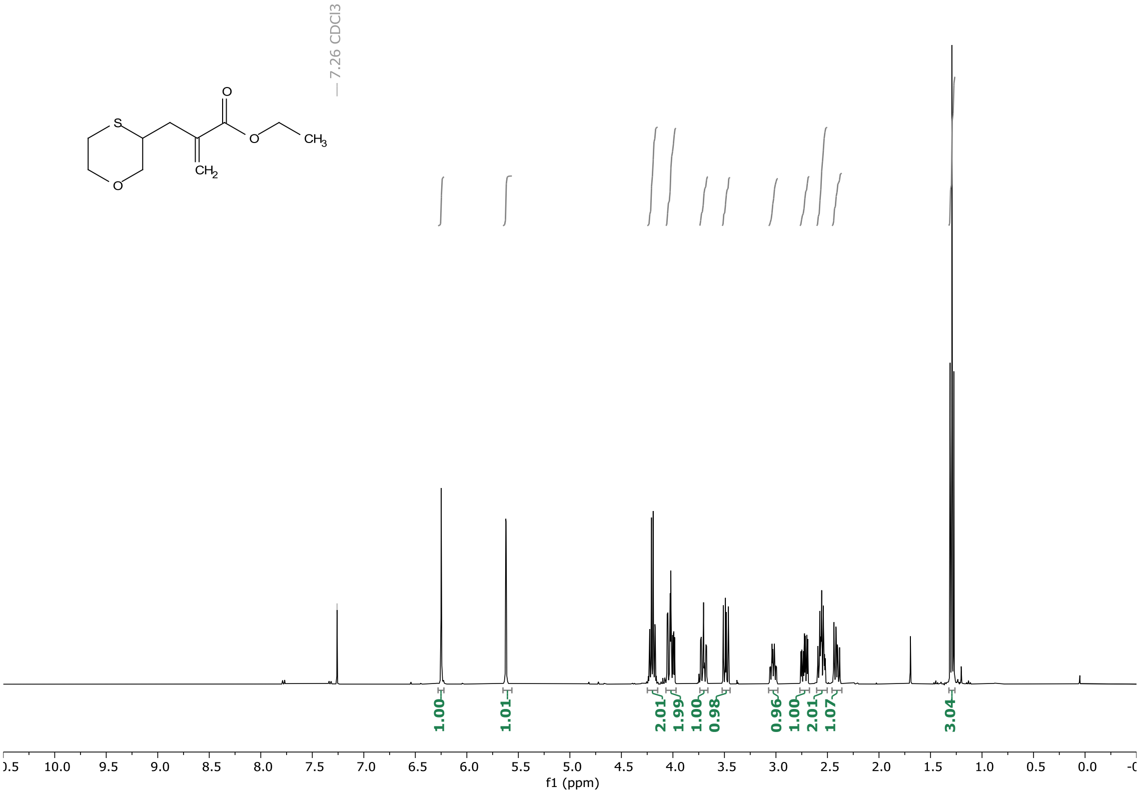

^{13}C NMR (101 MHz, $CDCl_3$) of compound **9**

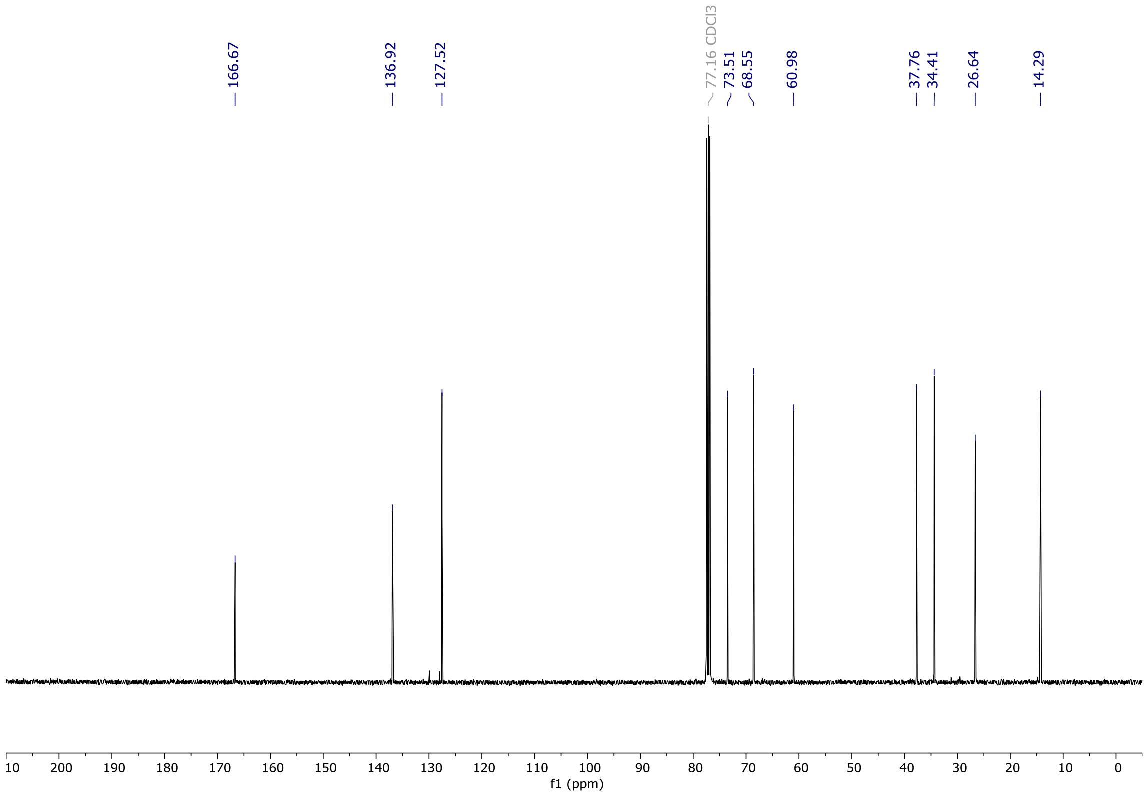

^{1}H NMR (400 MHz, $CDCl_3$) of compound **10**

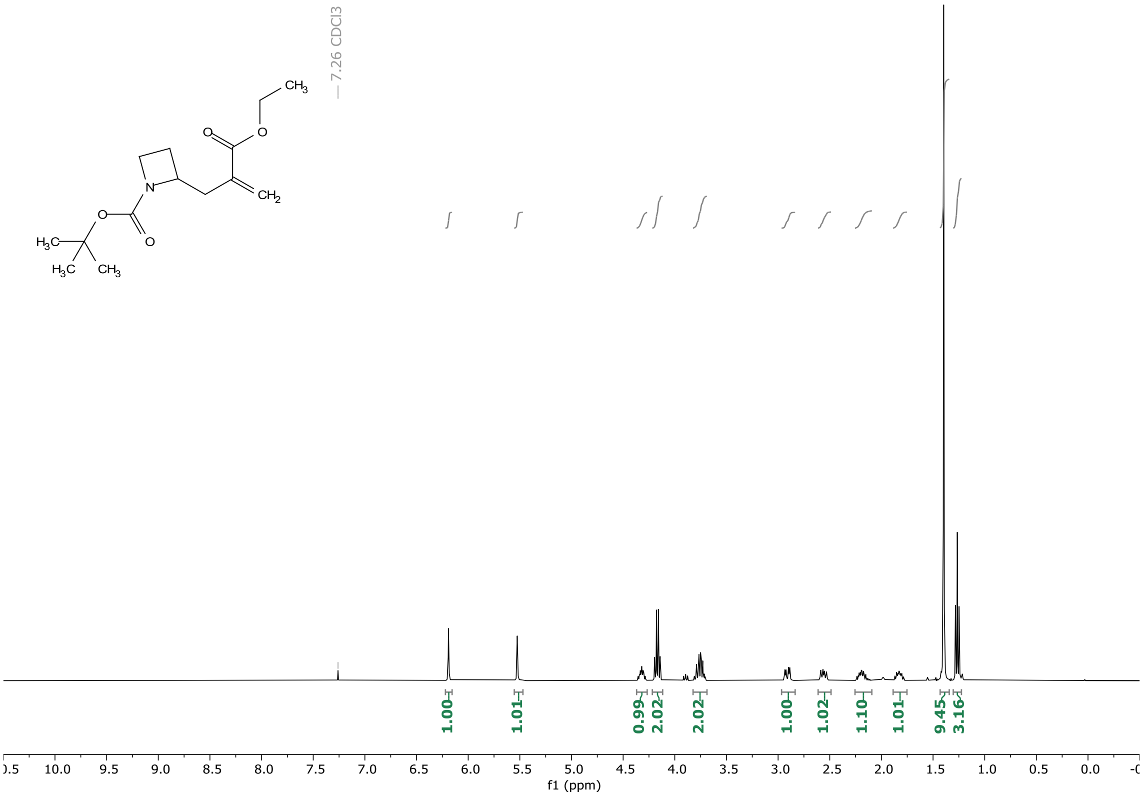

^{13}C NMR (101 MHz, $CDCl_3$) of compound **10**

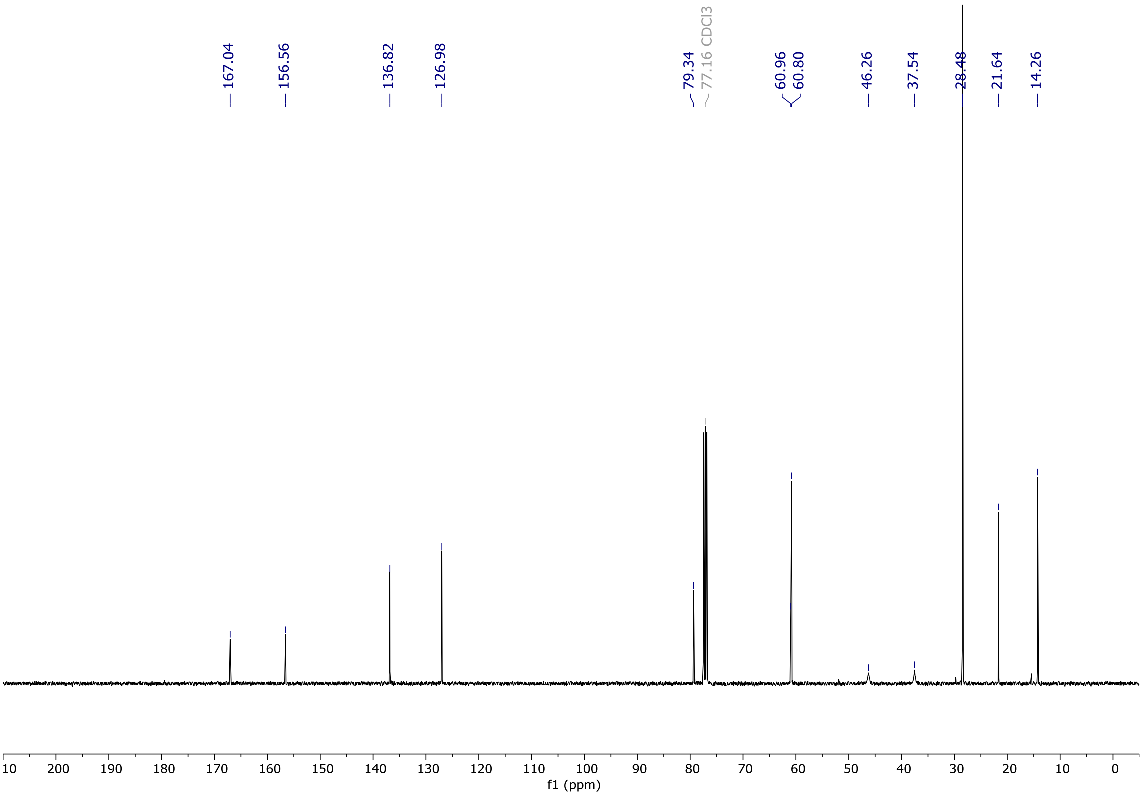

^{1}H NMR (400 MHz, $CDCl_3$) of compound **11**

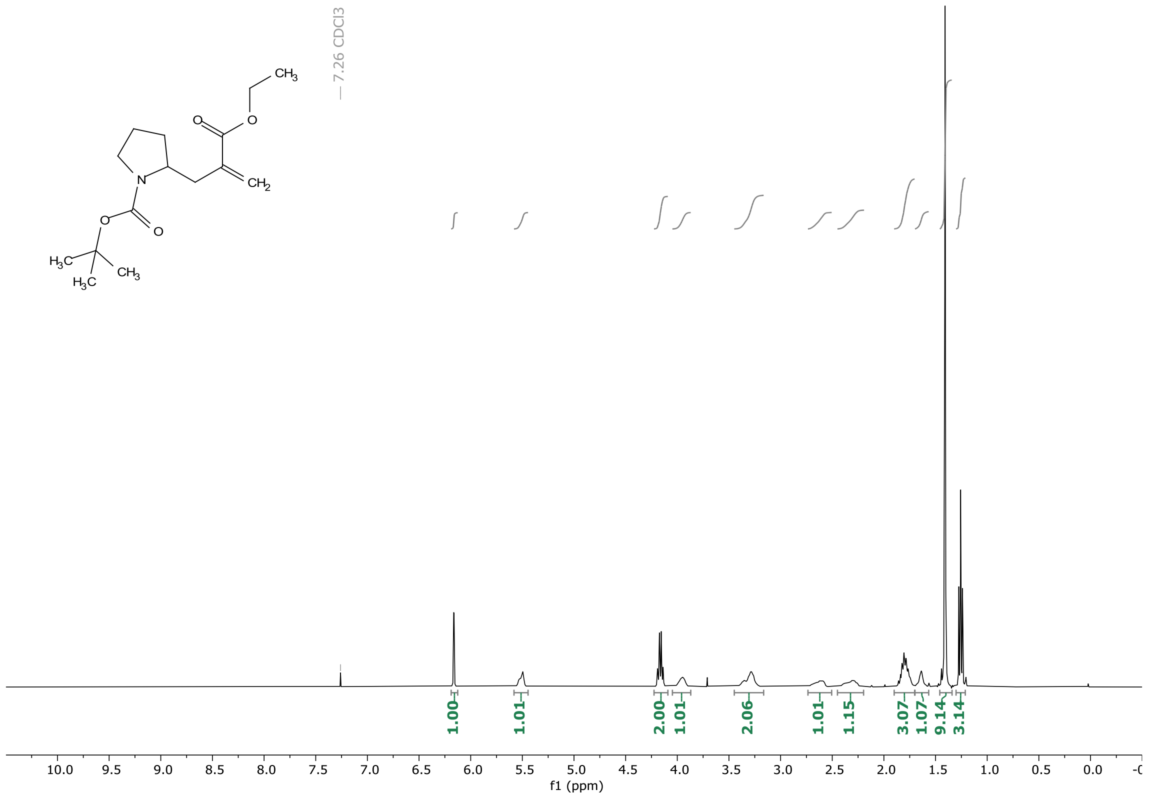

^{13}C NMR (100 MHz, $CDCl_3$) of compound **11**

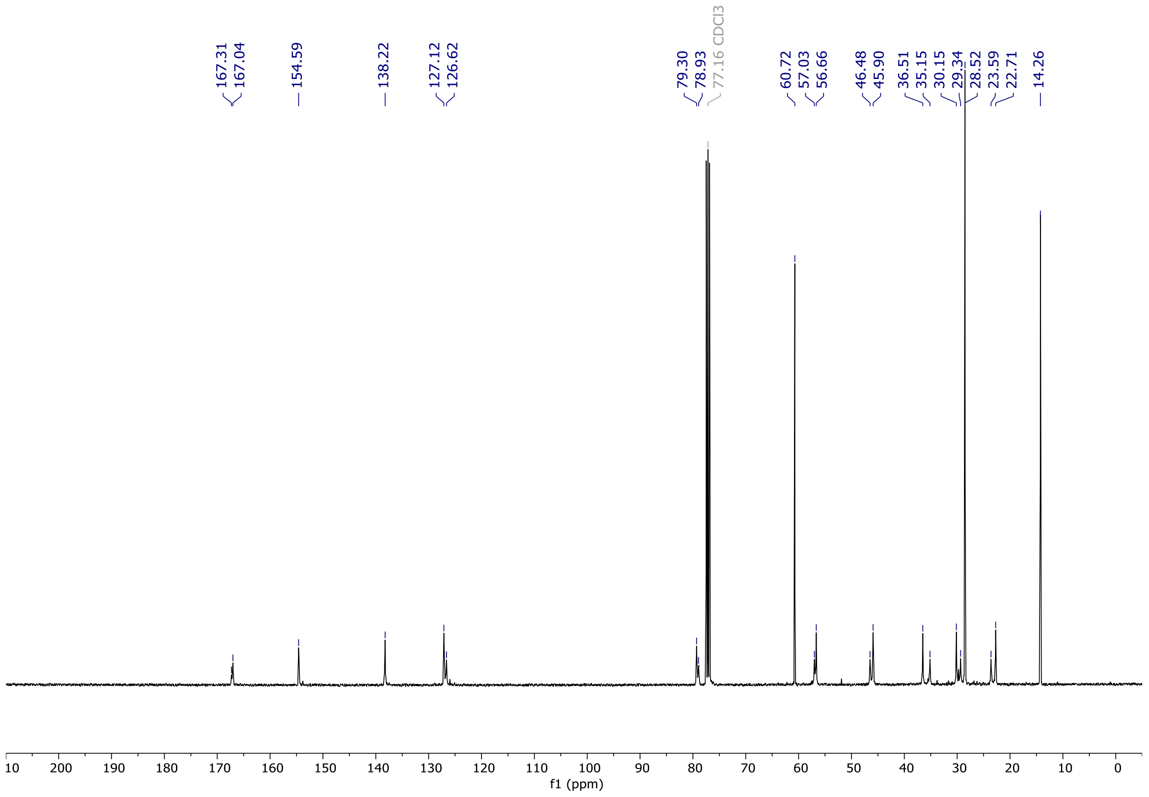

^1H NMR (400 MHz, $CDCl_3$) of compound **12**

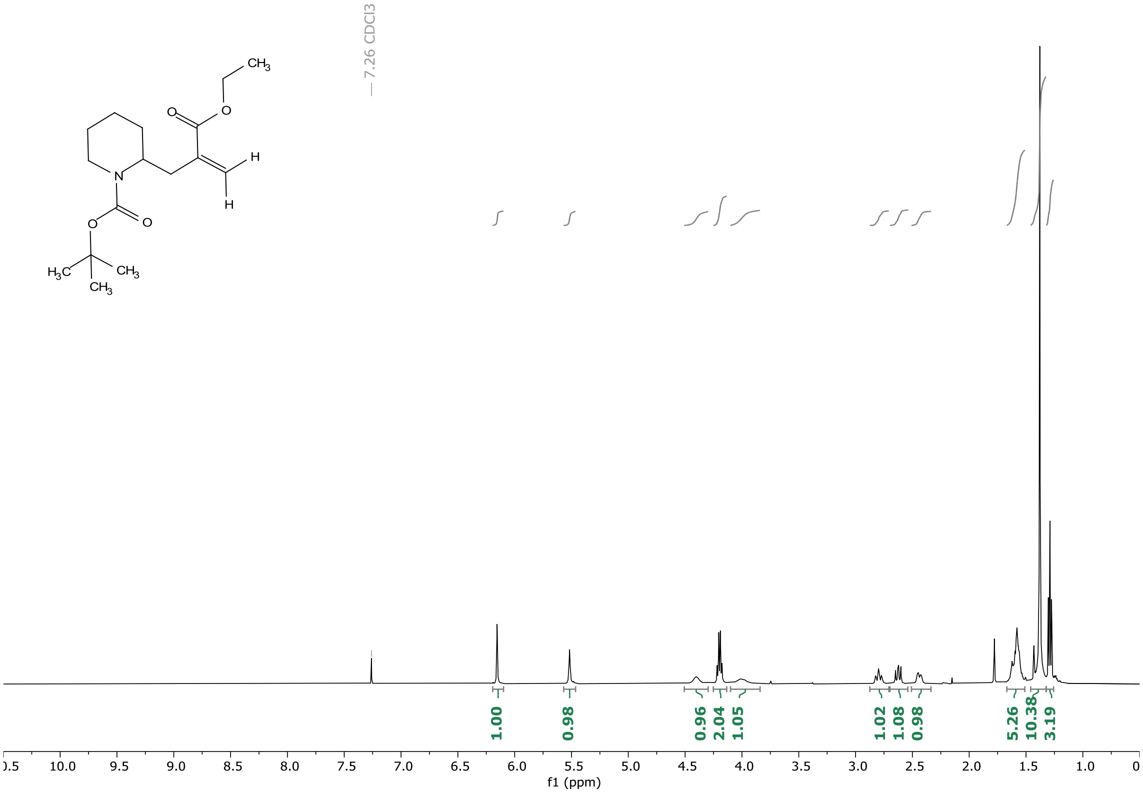

^{13}C NMR (101 MHz, $CDCl_3$) of compound **12**

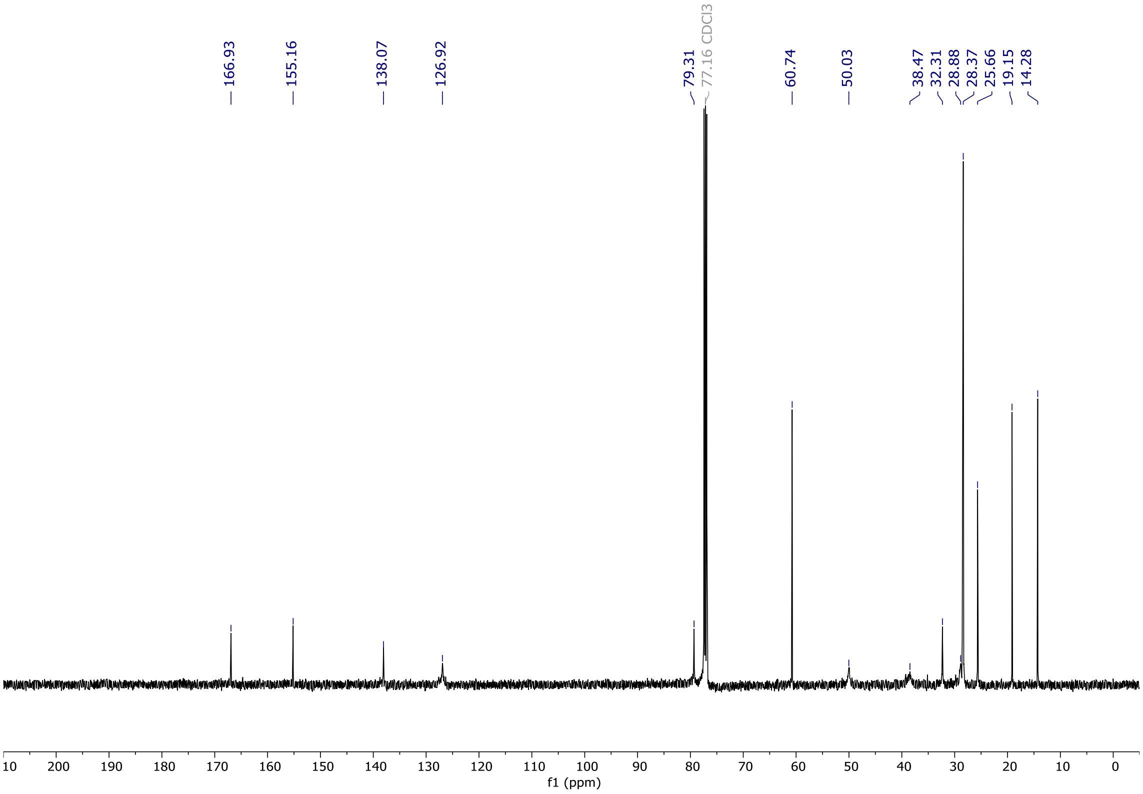

^1H NMR (400 MHz, $CDCl_3$) of compound **13**

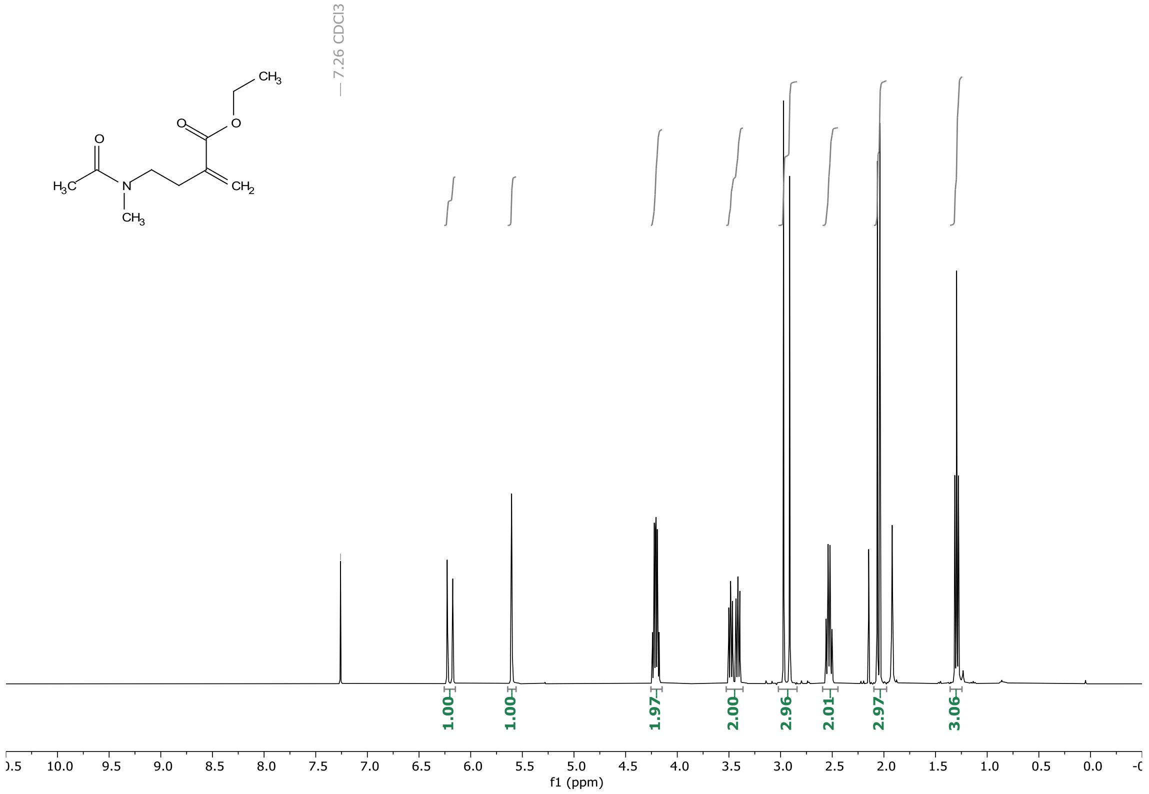

^{13}C NMR (101 MHz, $CDCl_3$) of compound **13**

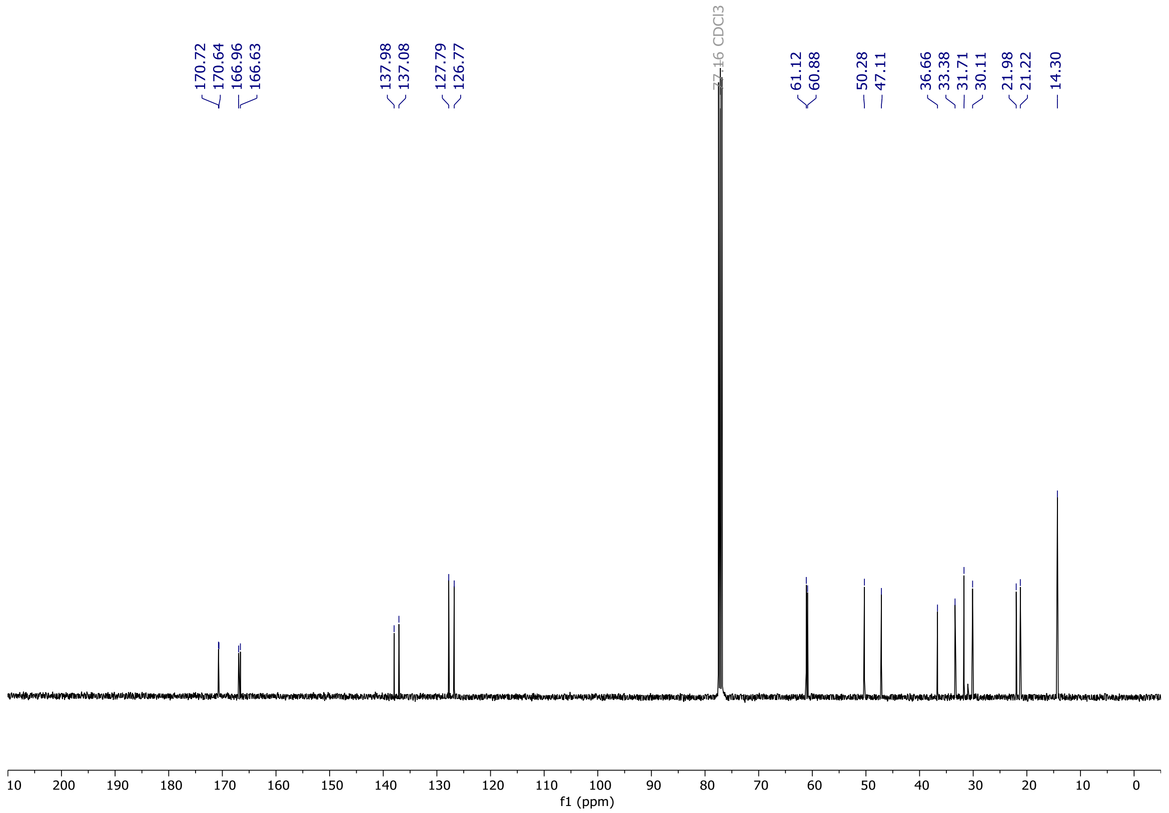

^{1}H NMR (400 MHz, $CDCl_3$) of compound **14**

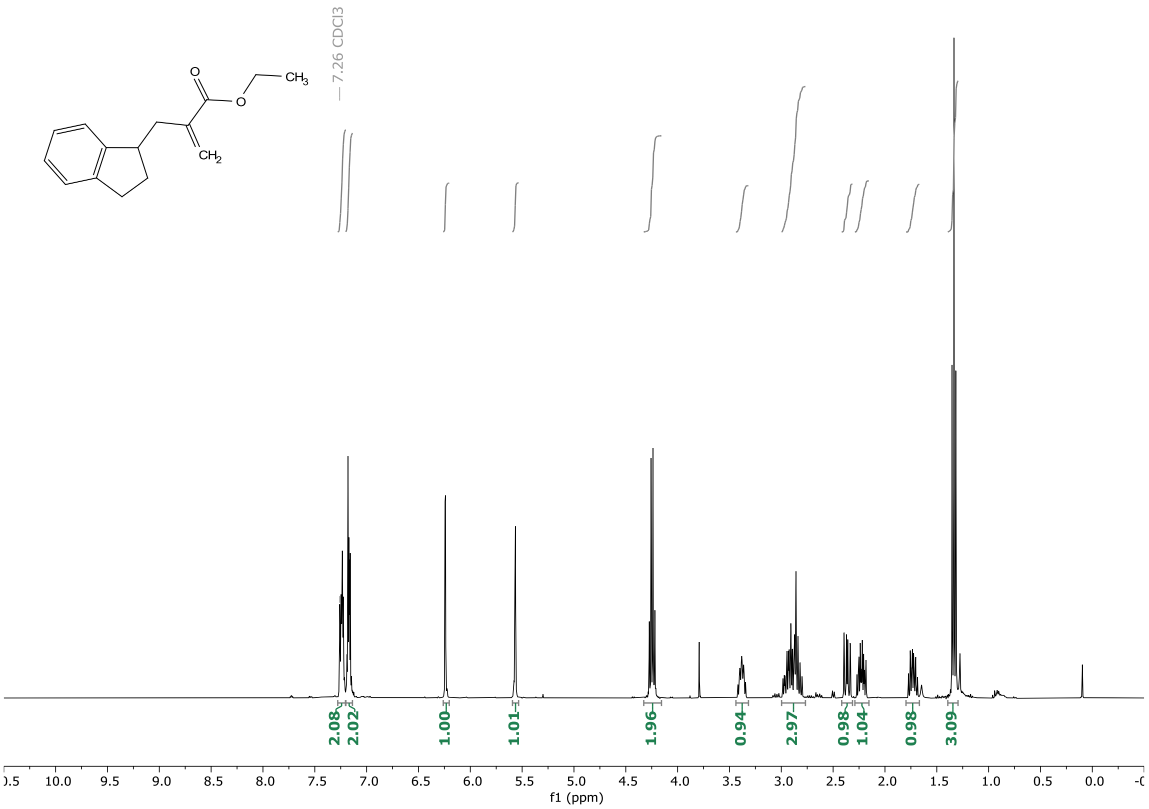

^{13}C NMR (100 MHz, $CDCl_3$) of compound **14**

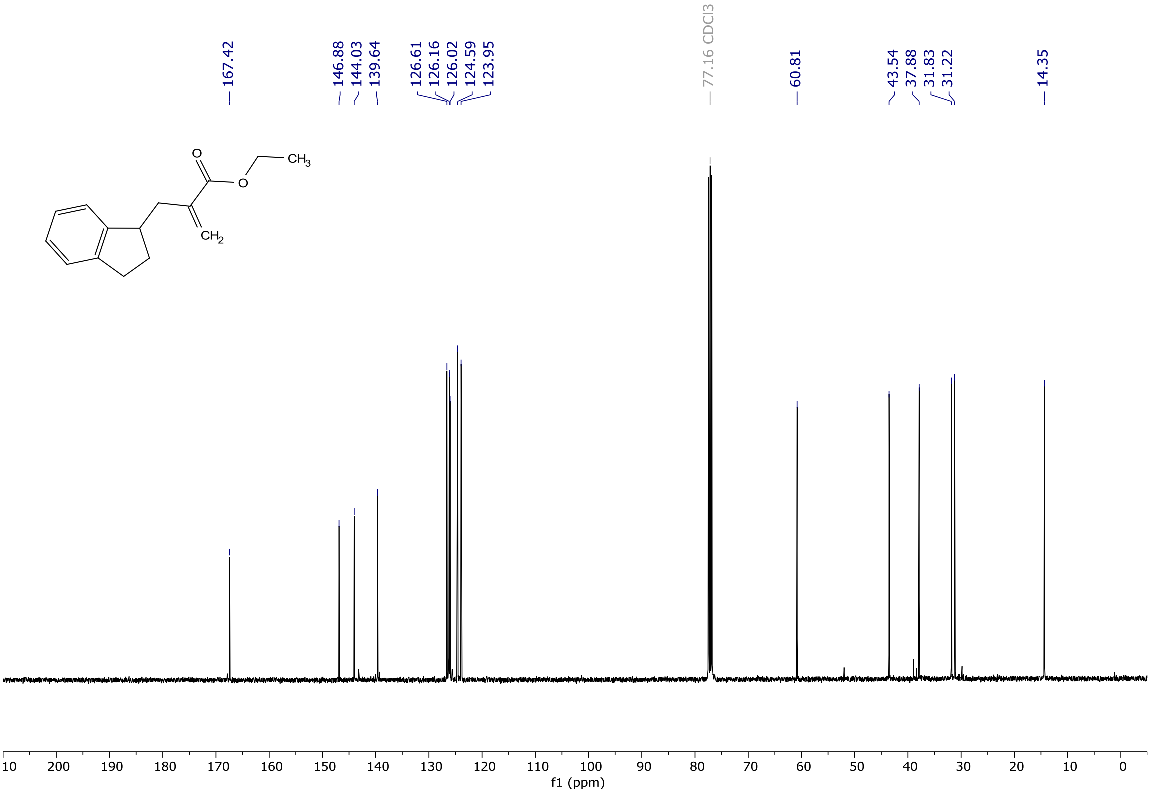

^1H NMR (400 MHz, $CDCl_3$) of compound **15**

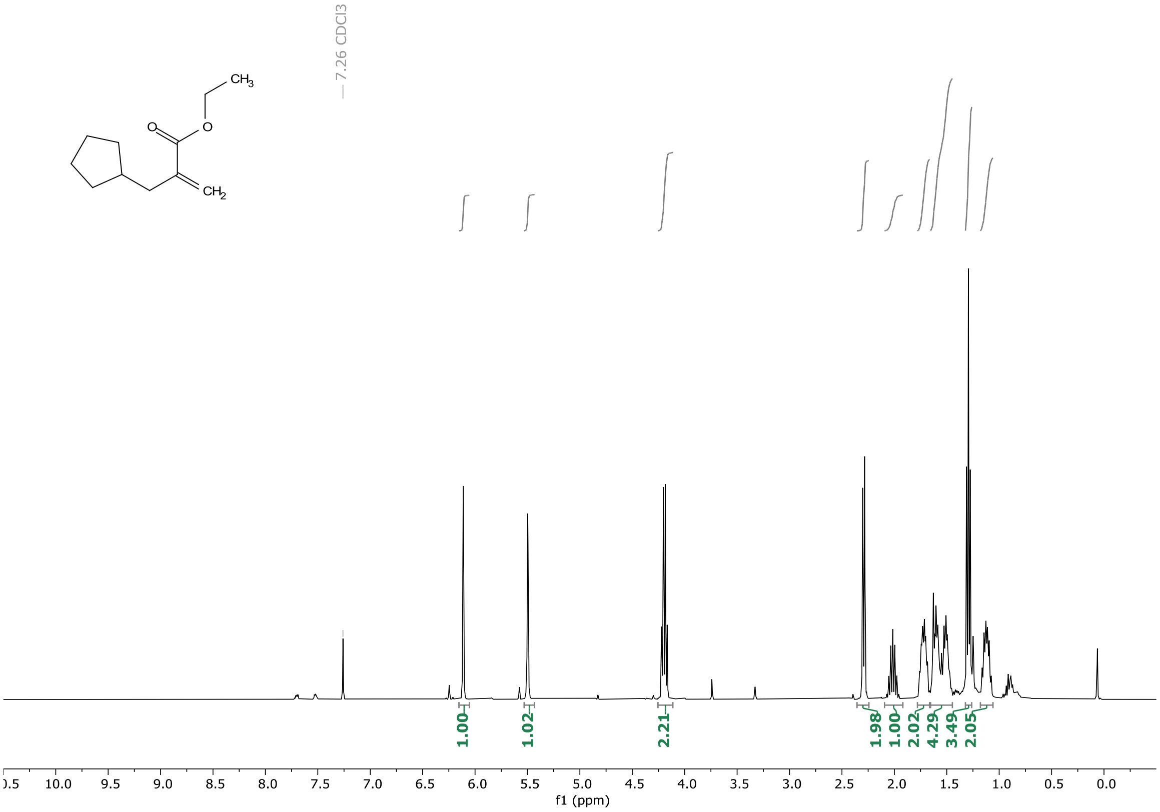

^{13}C NMR (101 MHz, $CDCl_3$) of compound **15**

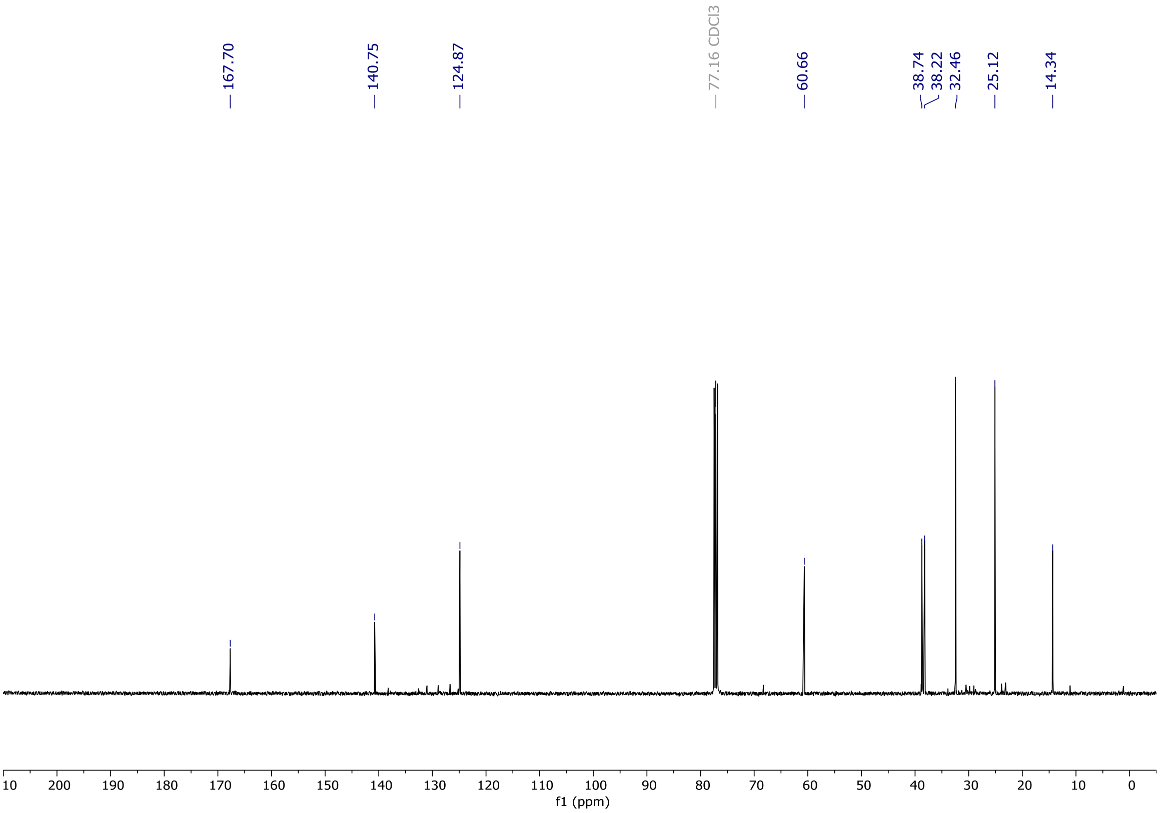

^{1}H NMR (400 MHz, $CDCl_3$) of compound **16**

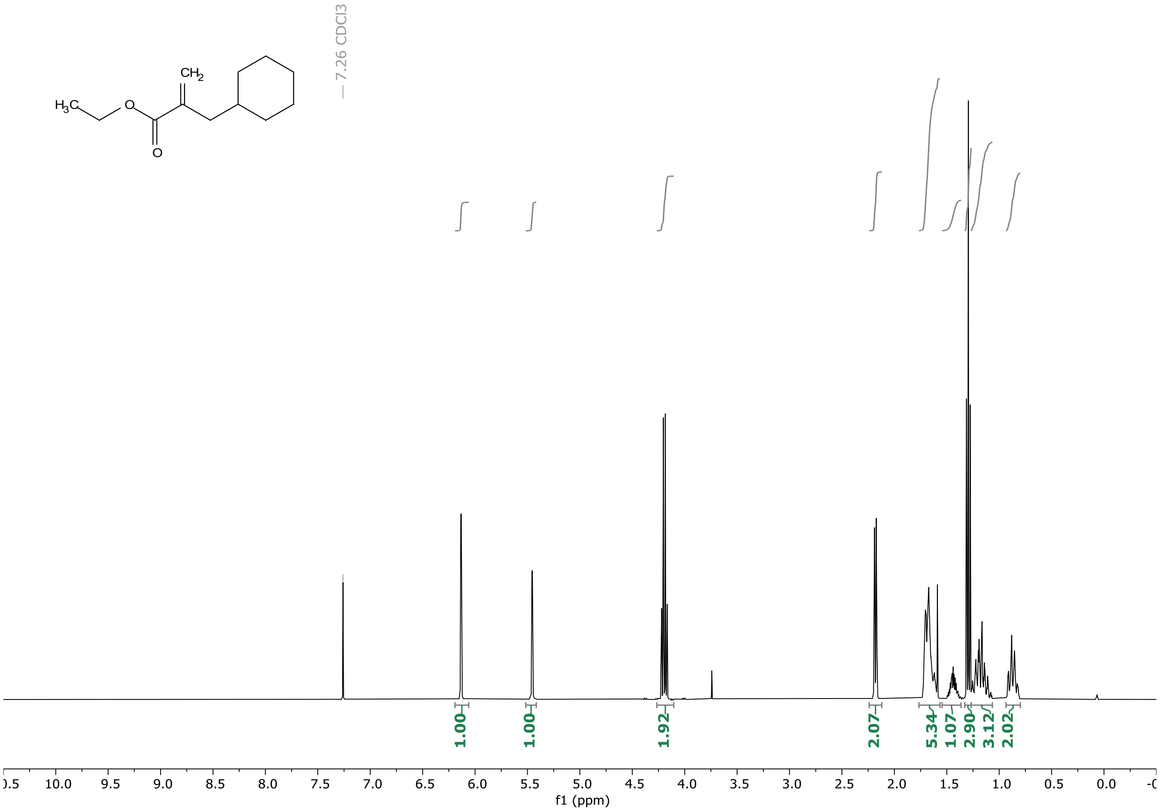

^{13}C NMR (101 MHz, $CDCl_3$) of compound **16**

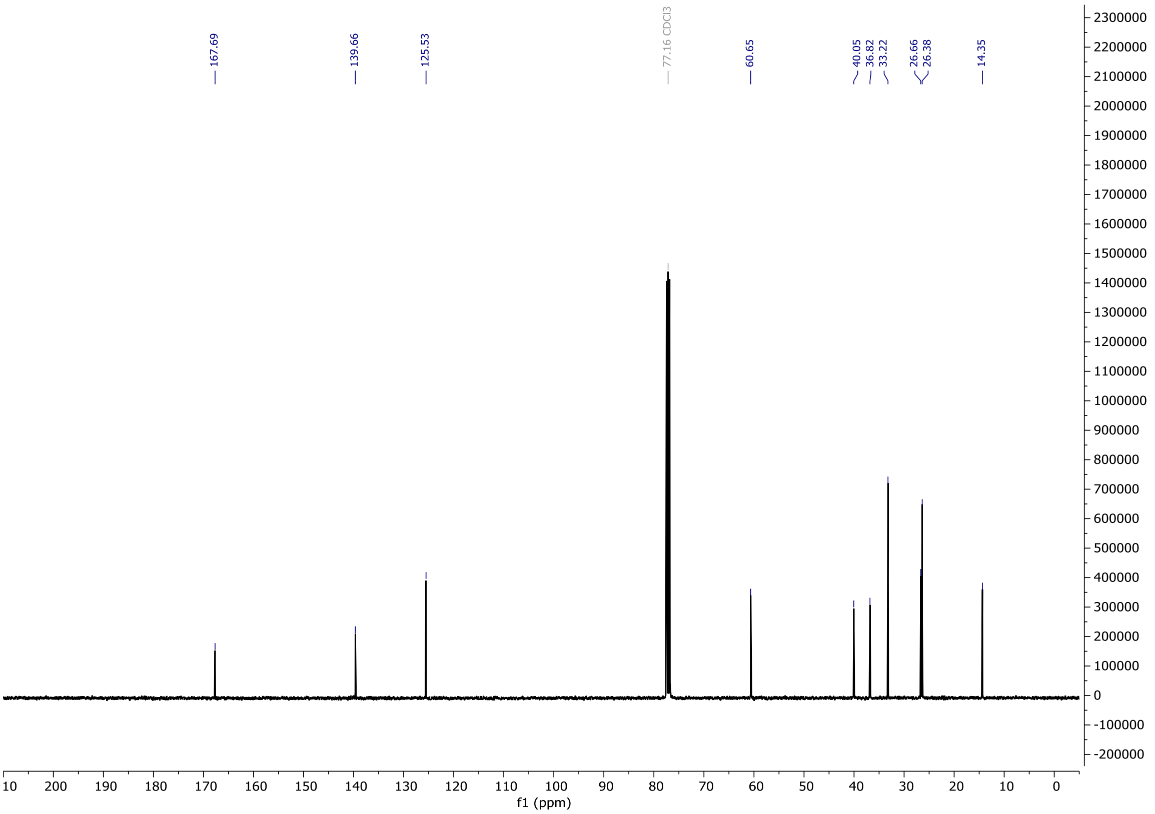

^{1}H NMR (400 MHz, $CDCl_3$) of compound **17**

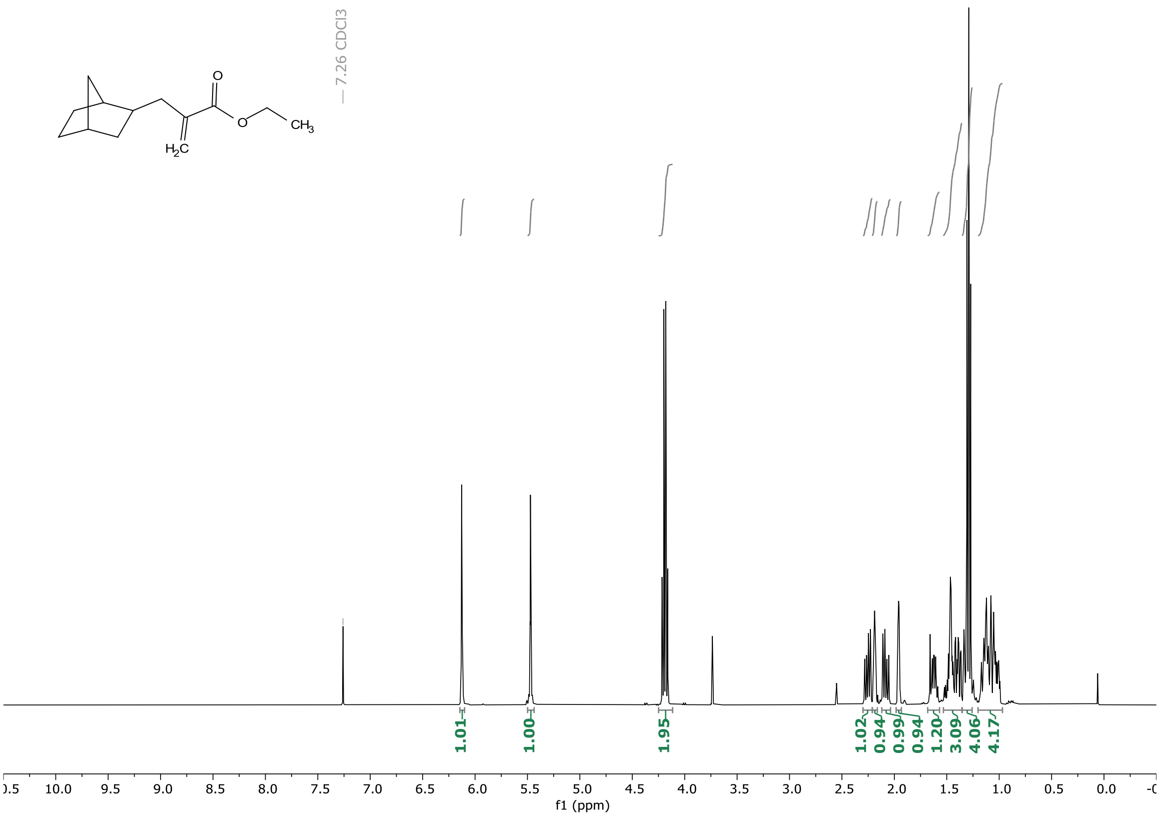

^{13}C NMR (101 MHz, $CDCl_3$) of compound **17**

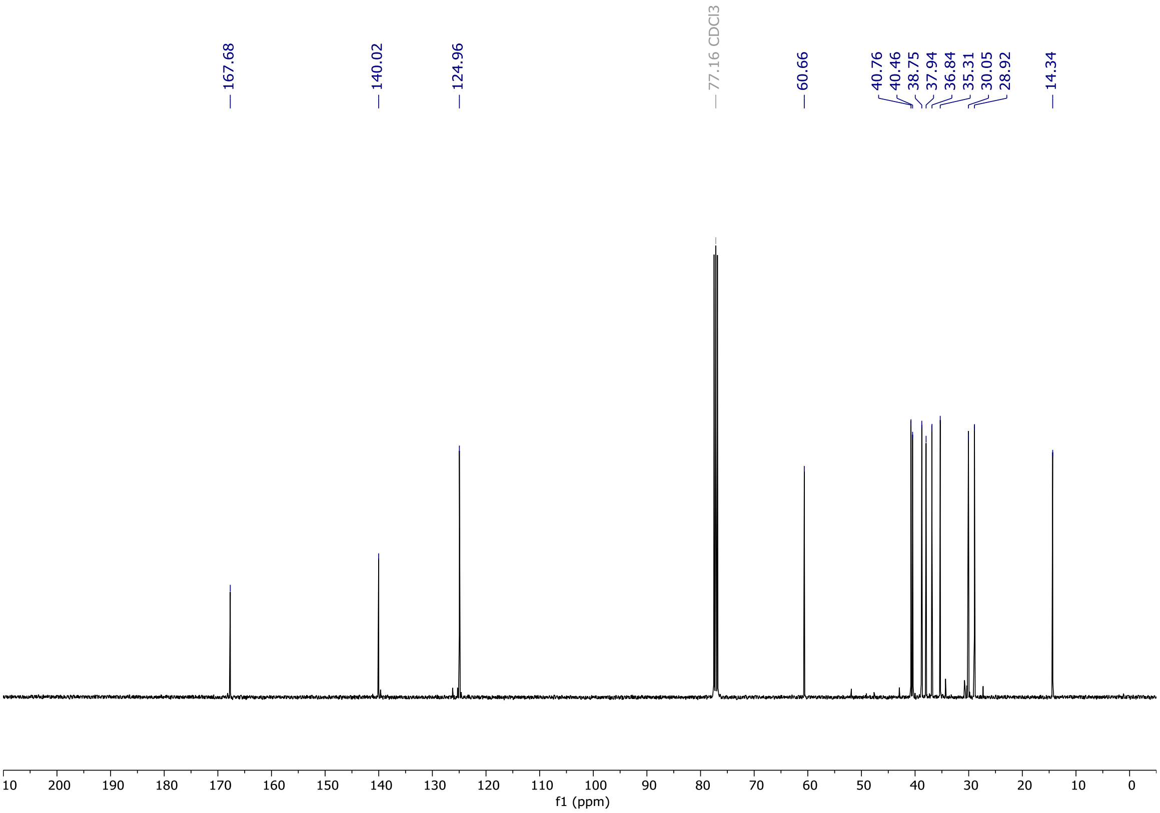

^{1}H NMR (400 MHz, $CDCl_3$) of compound **18**

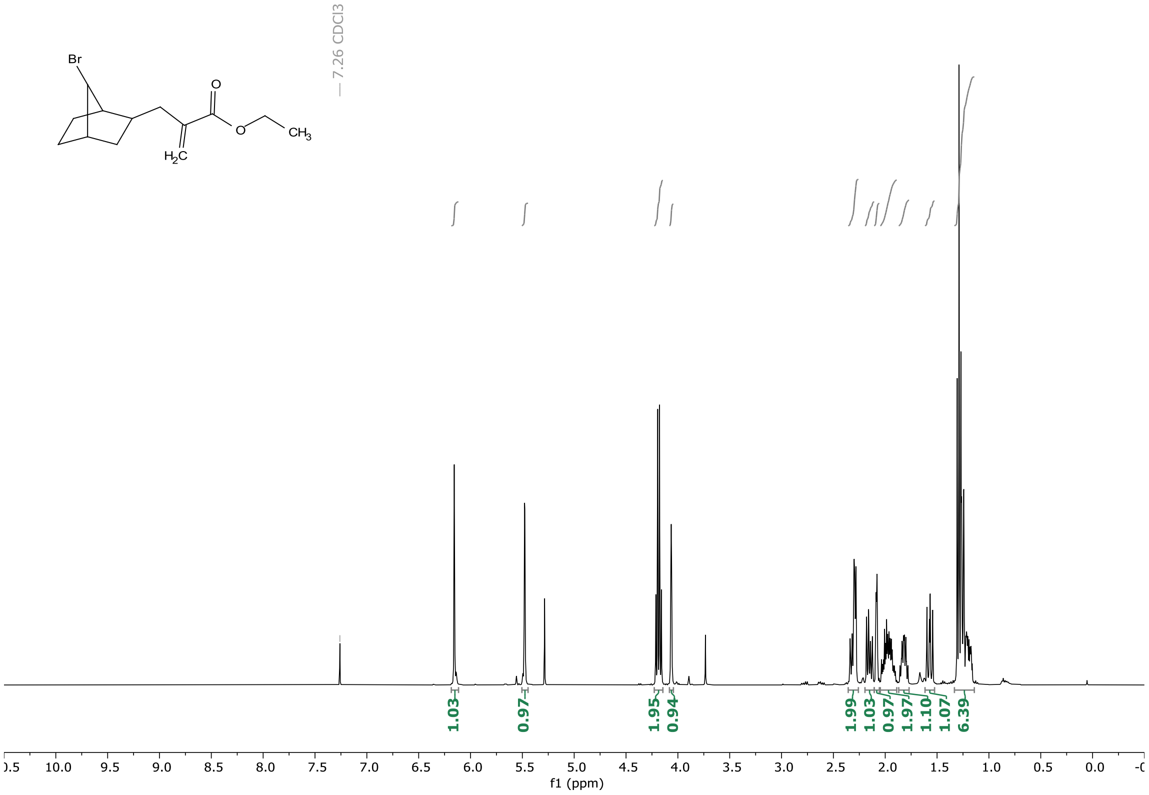

^{13}C NMR (101 MHz, $CDCl_3$) of compound **18**

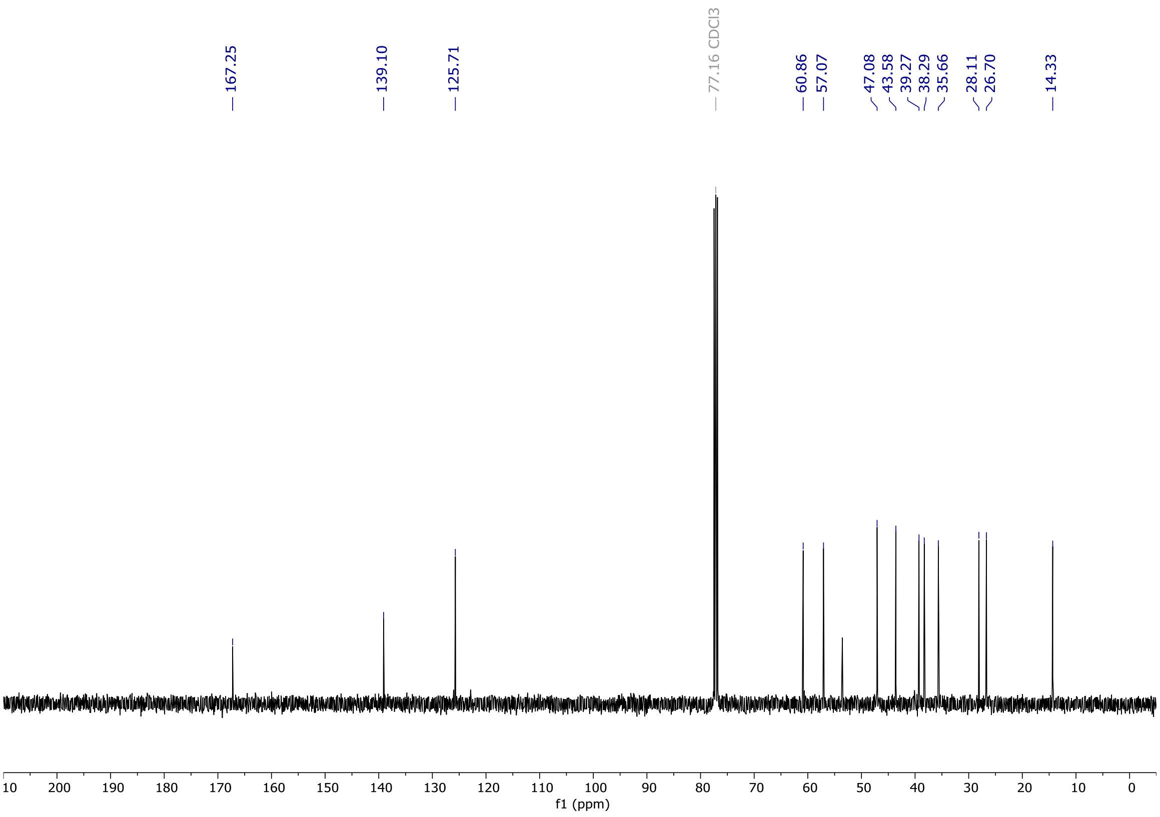

^{1}H–^{1}H NOESY (400 MHz, $CDCl_3$) of compound **18**

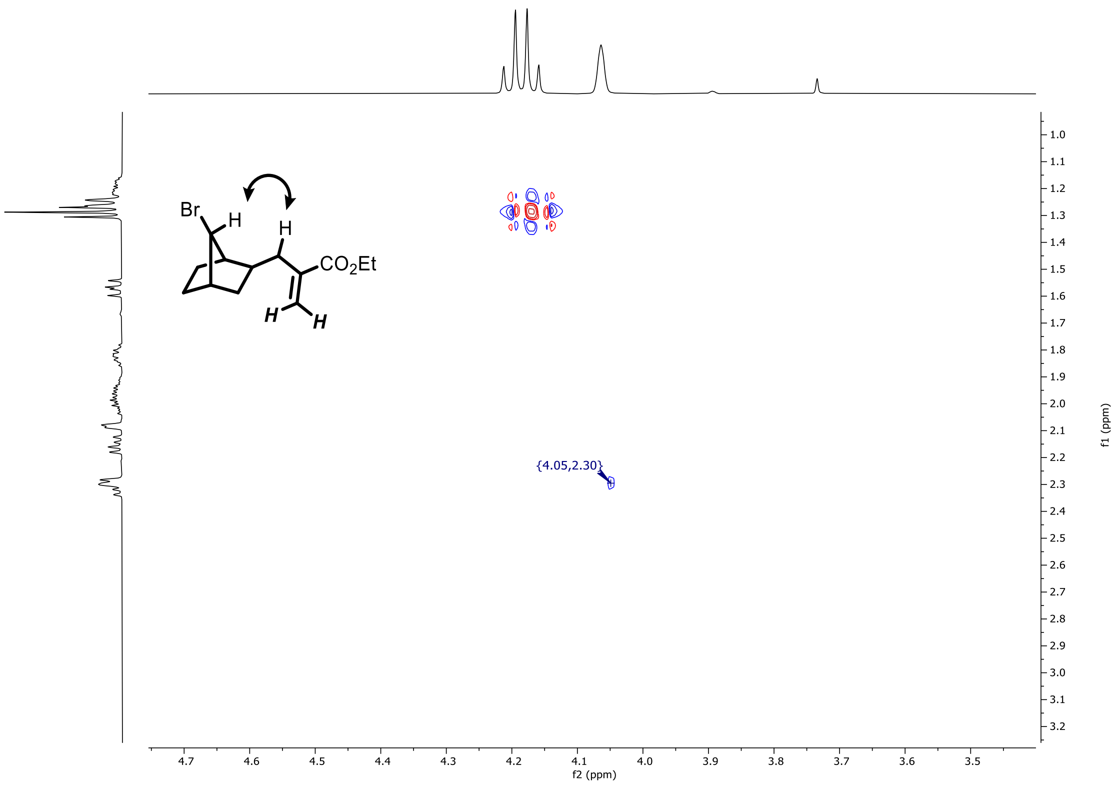

Br
H
H
CO2Et
H
H
{4.05,2.30}
f1 (ppm)
f2 (ppm)

^{1}H NMR (400 MHz, $CDCl_3$) of compound **19**

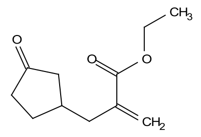

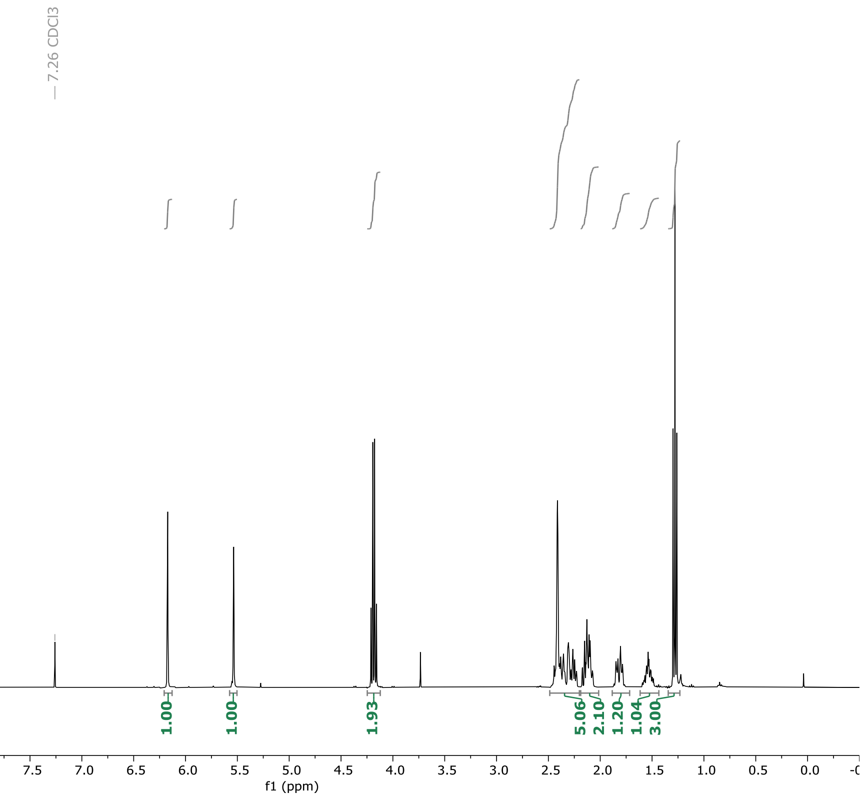

^{13}C NMR (101 MHz, $CDCl_3$) of compound **19**

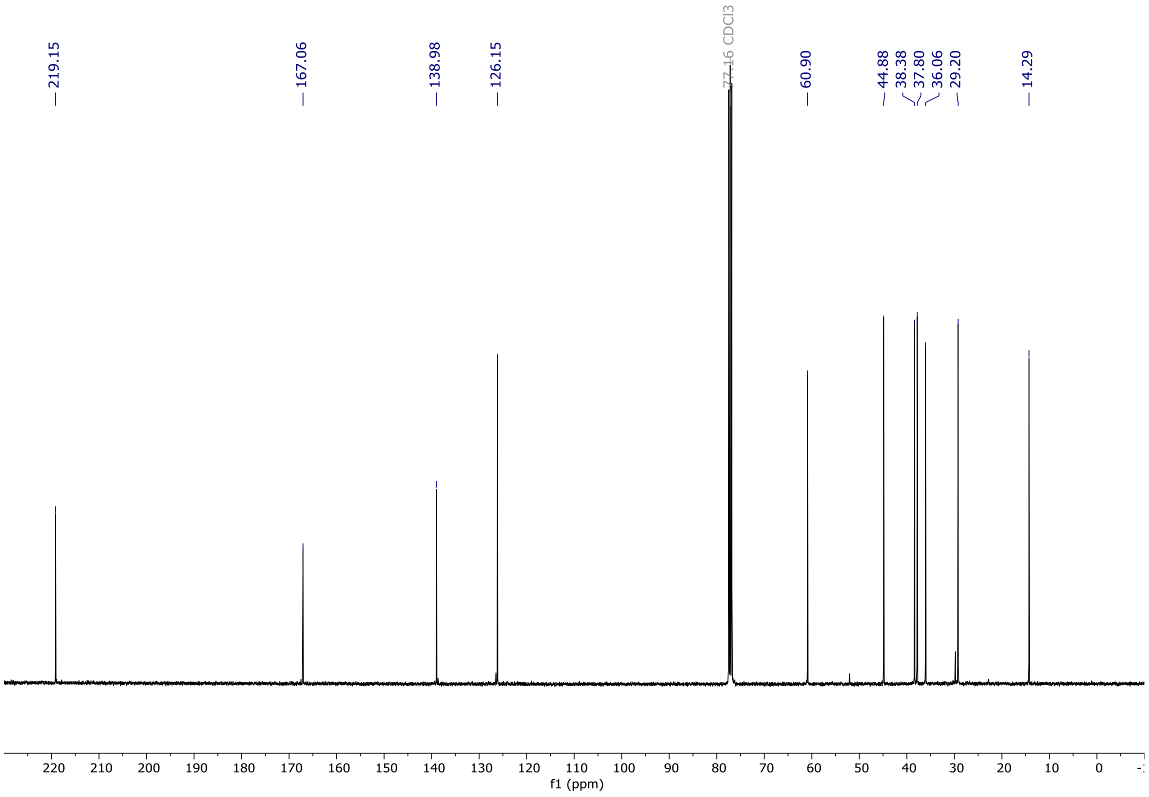

^{1}H NMR (400 MHz, $CDCl_3$) of compound **4-d₂**

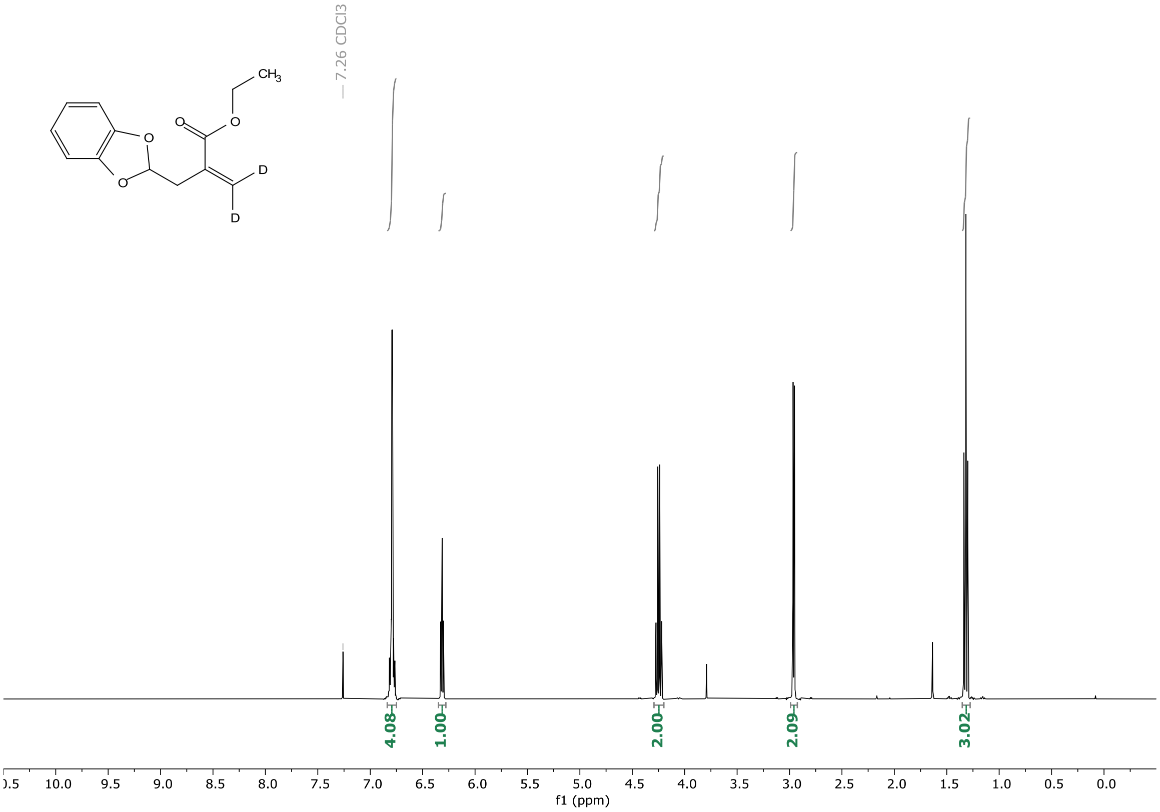

^{13}C NMR (101 MHz, $CDCl_3$) of compound **4-d₂**

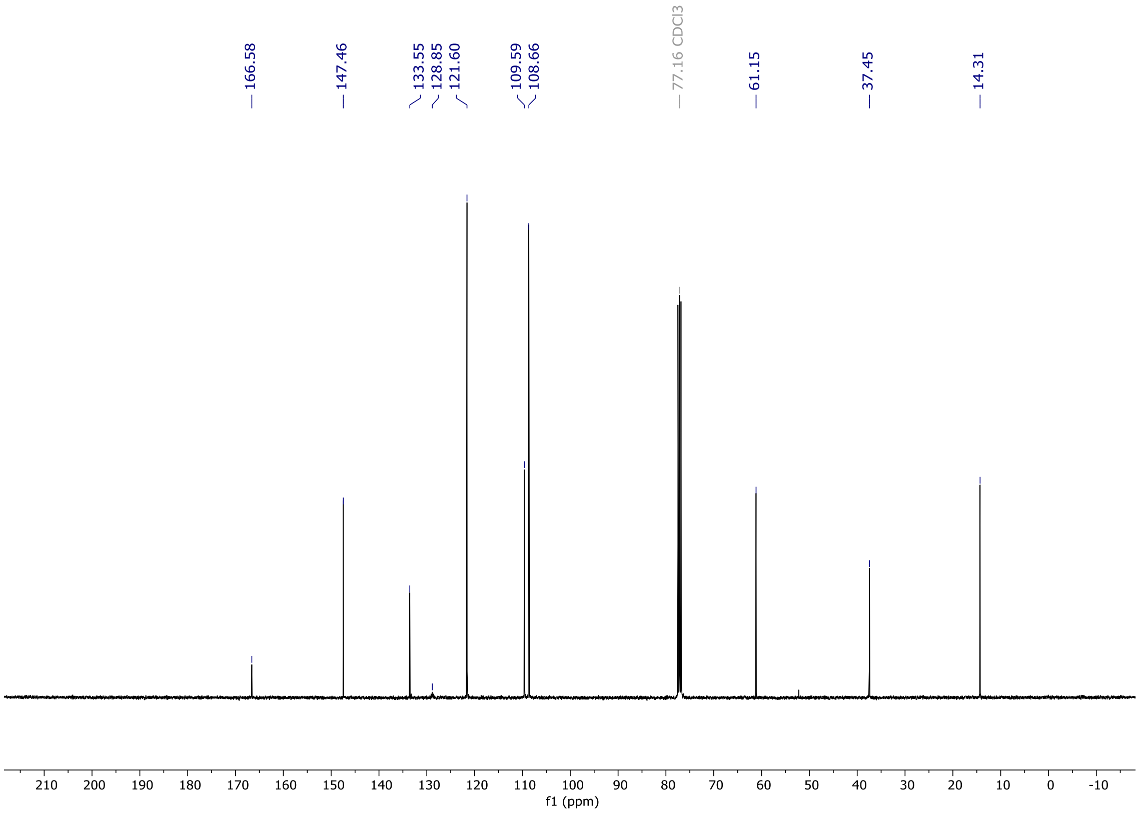

^{1}H NMR (400 MHz, $CDCl_3$) of compounds **20**

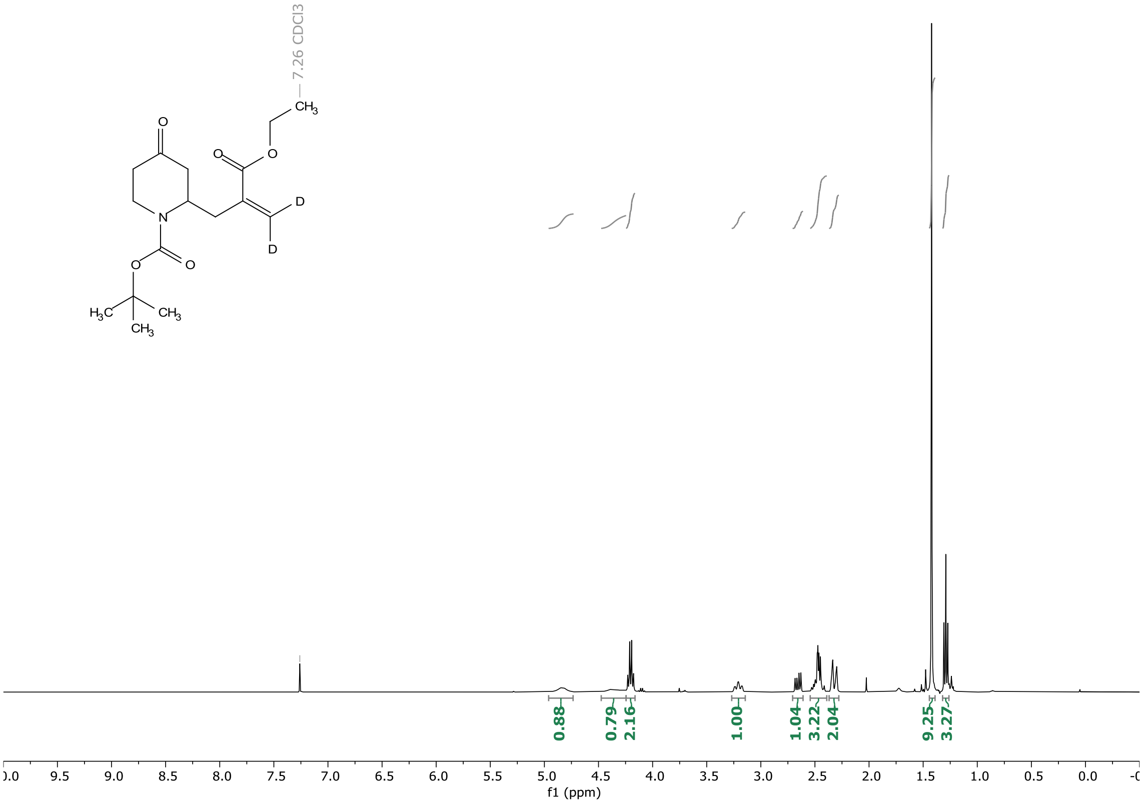

^{13}C NMR (101 MHz, $CDCl_3$) of compounds **20**

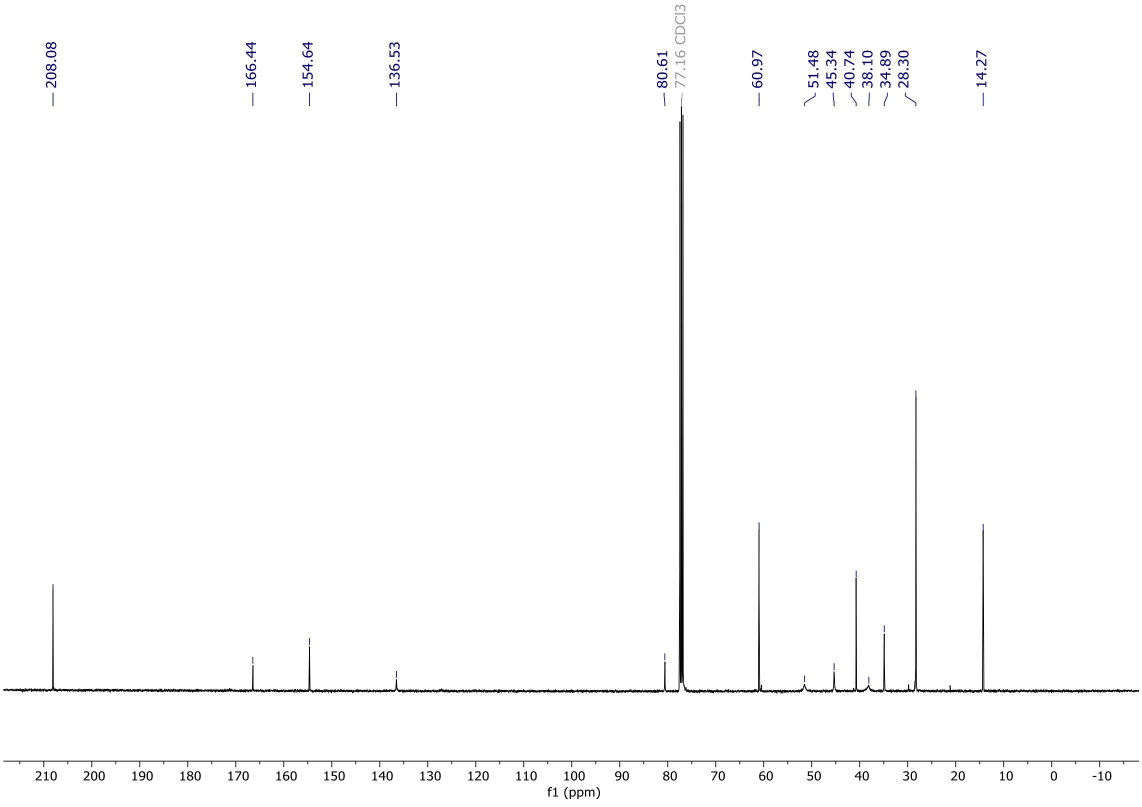

^{1}H NMR (400 MHz, $CDCl_3$) of compound **21**

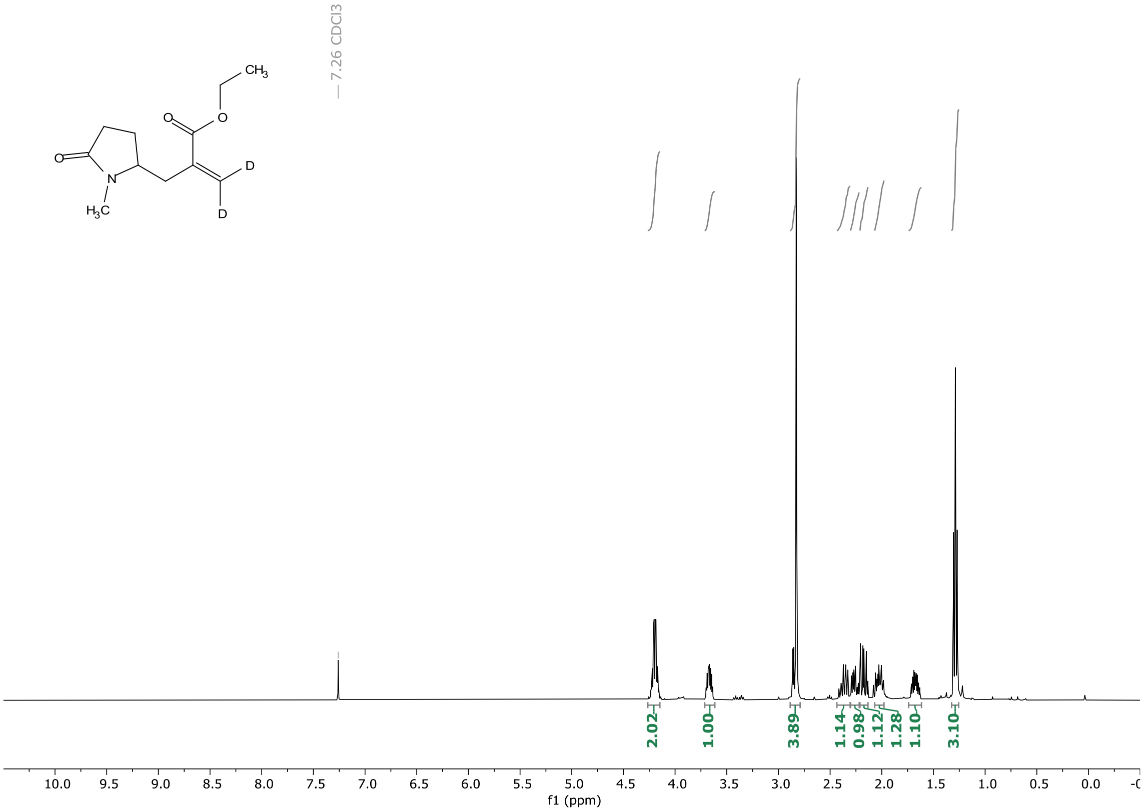

^{13}C NMR (101 MHz, $CDCl_3$) of compound **21**

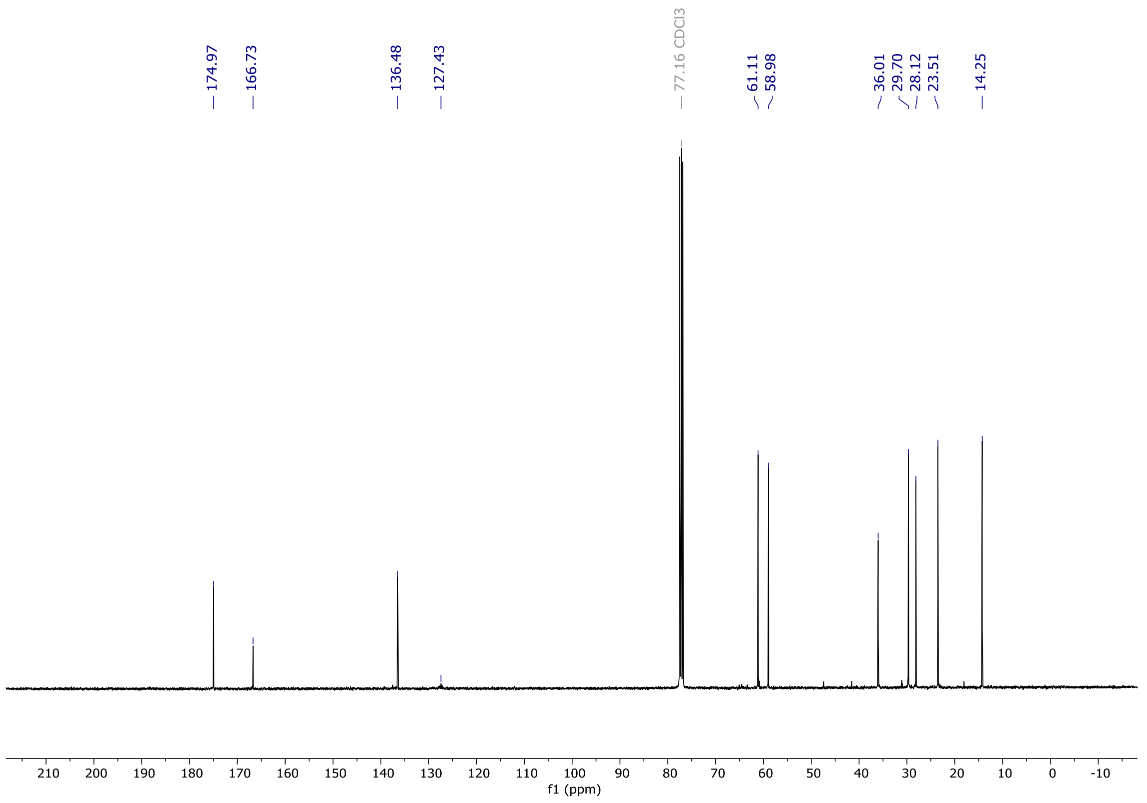

^{1}H NMR (400 MHz, $CDCl_3$) of compound **22**

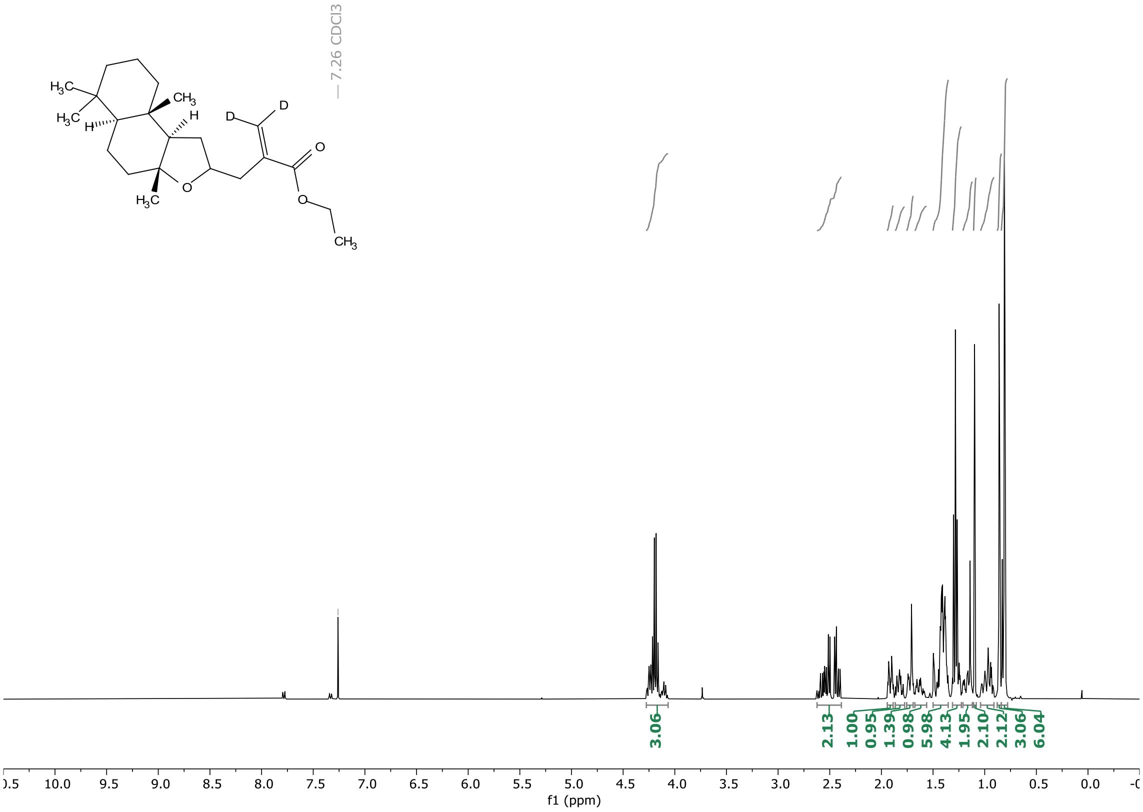

^{13}C NMR (101 MHz, $CDCl_3$) of compound **22**

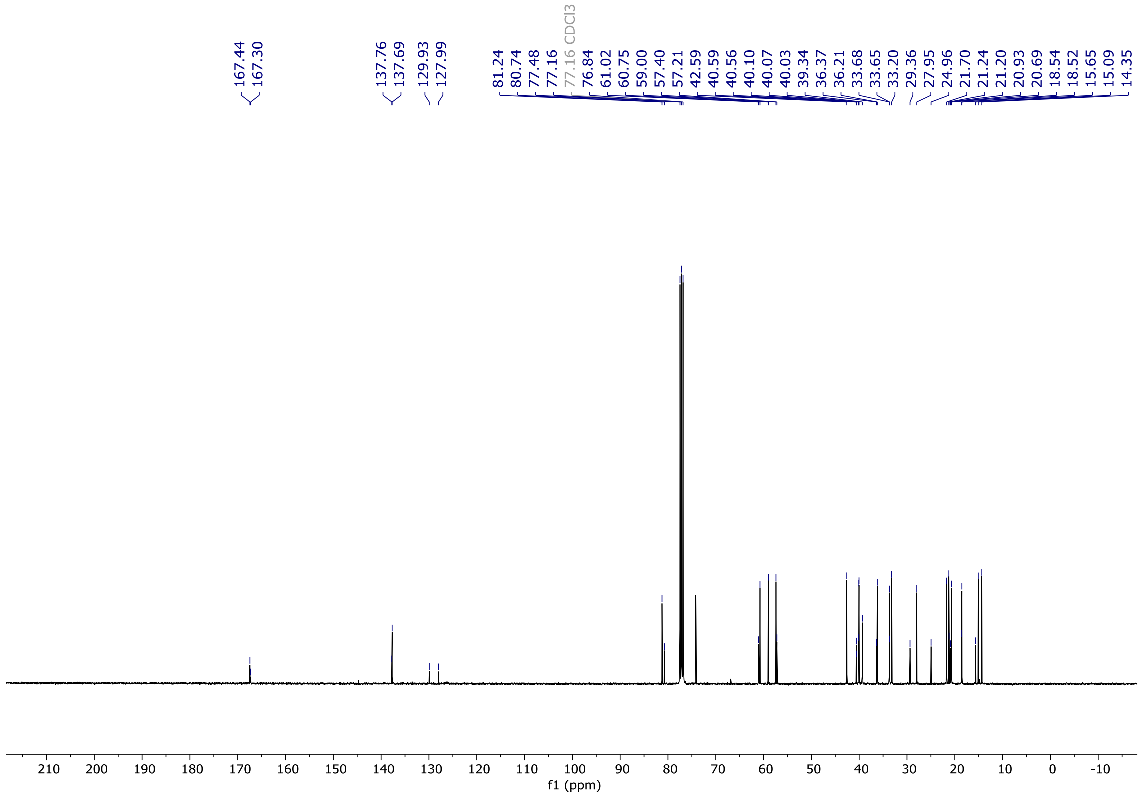

^{1}H NMR (400 MHz, $CDCl_3$) of compound **23**

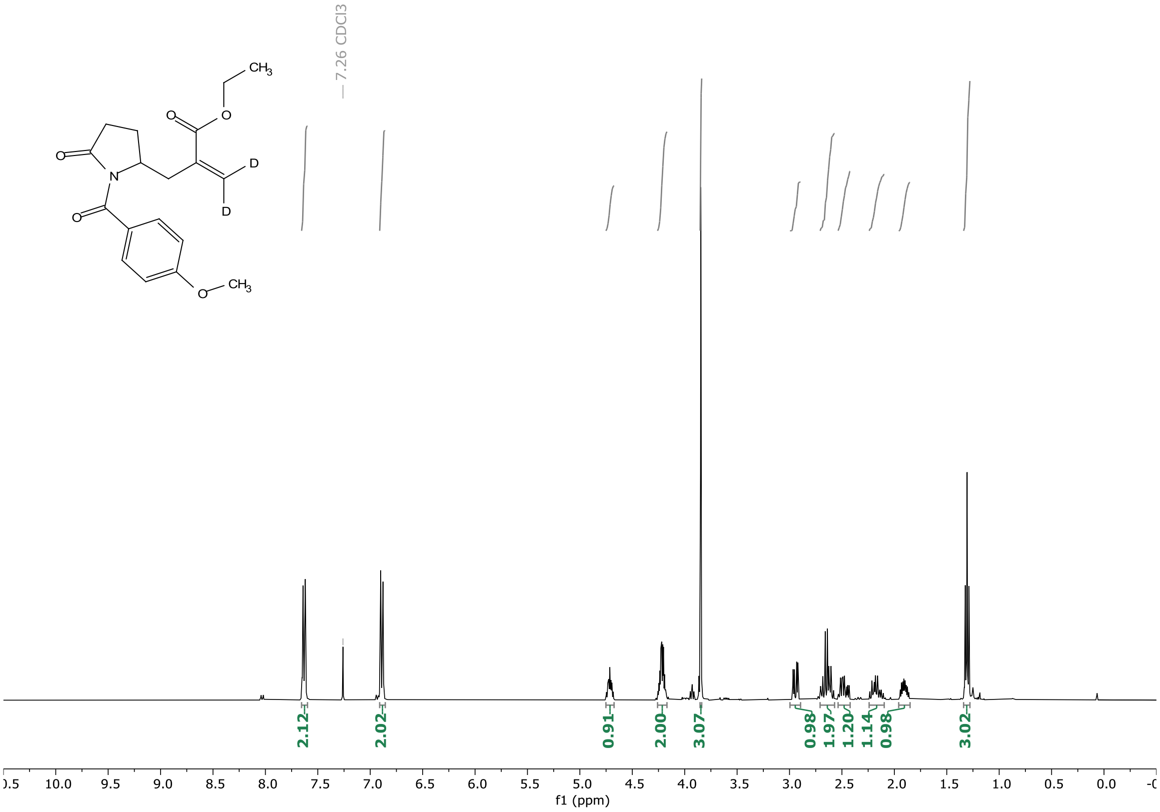

^{13}C NMR (101 MHz, $CDCl_3$) of compound **23**

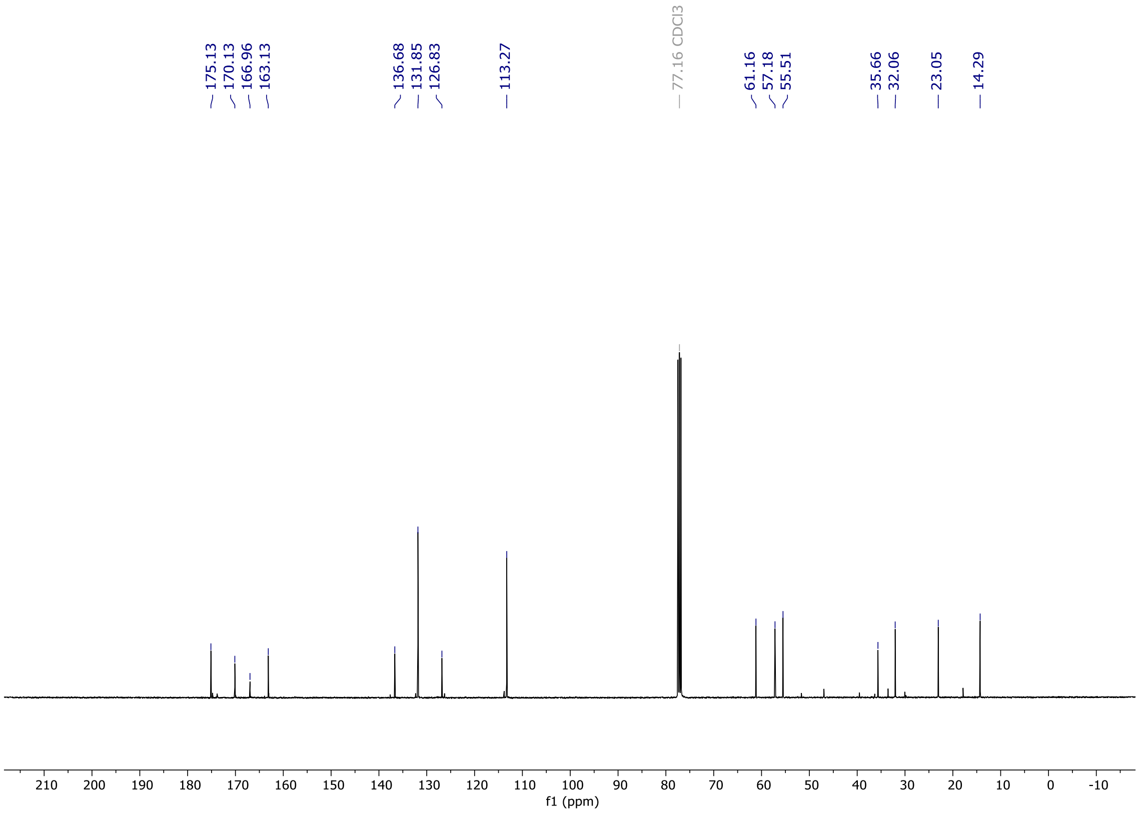

15. NMR Spectra of compounds 24-55

^{1}H NMR (400 MHz, $CDCl_3$) of compound **24** (major, E)

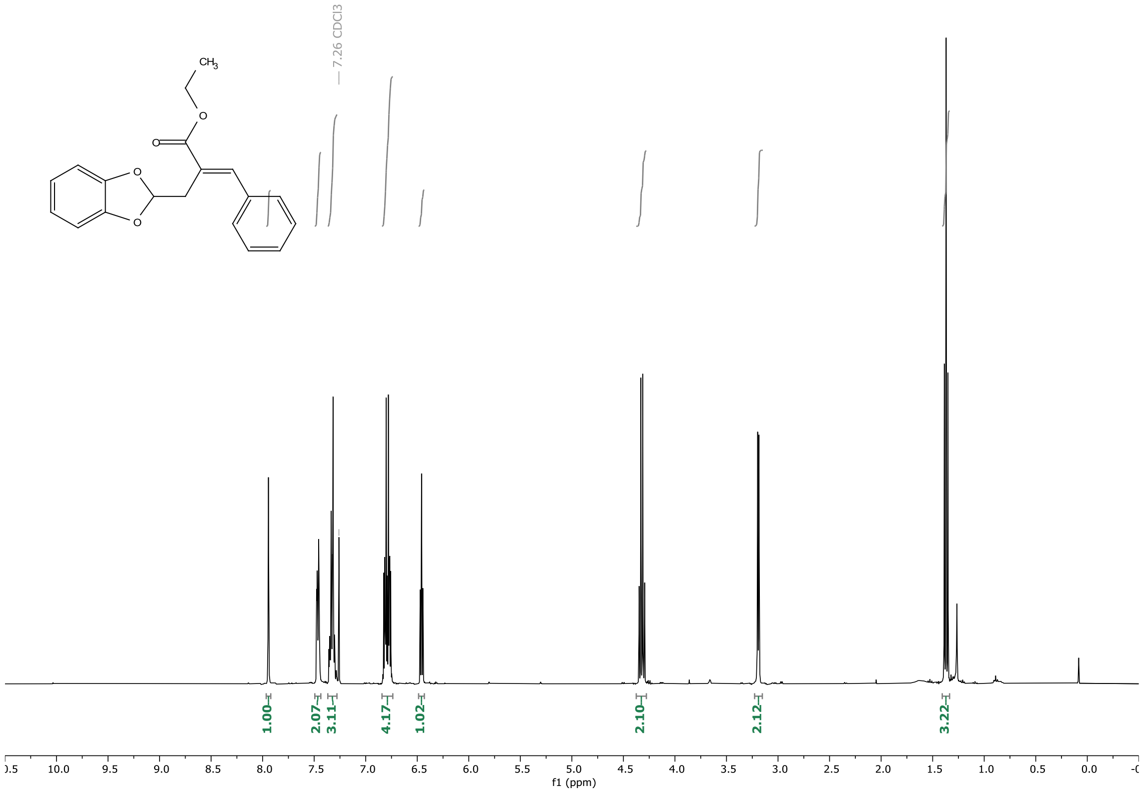

^{13}C NMR (101 MHz, $CDCl_3$) of compound **24** (major, E)

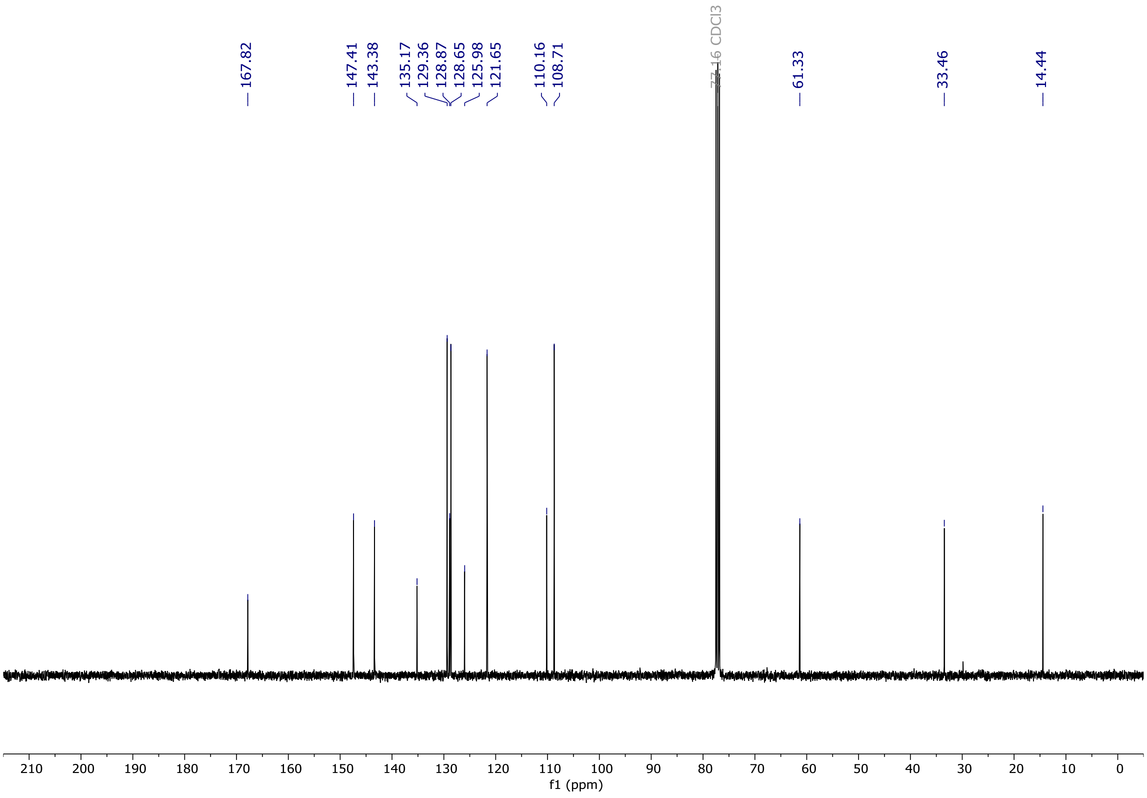

^{1}H NMR (400 MHz, $CDCl_3$) of compound **24** (minor, Z)

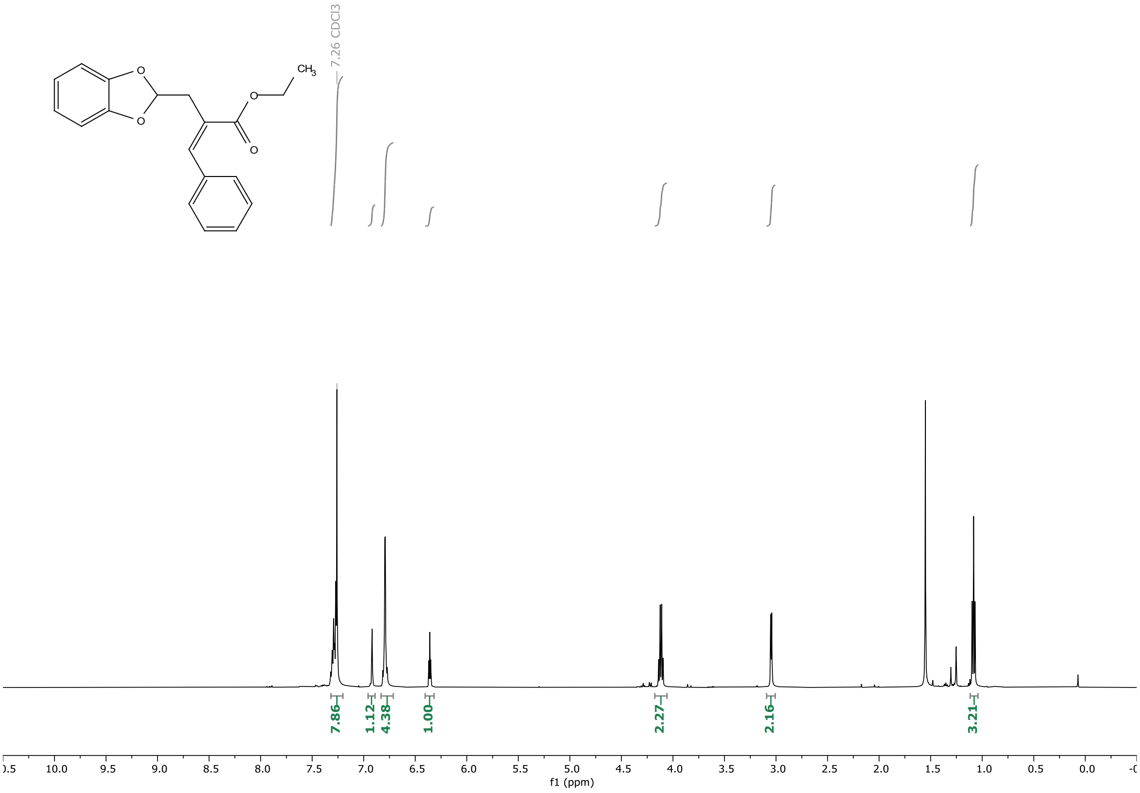

^{13}C NMR (101 MHz, $CDCl_3$) of compound **24** (minor, Z)

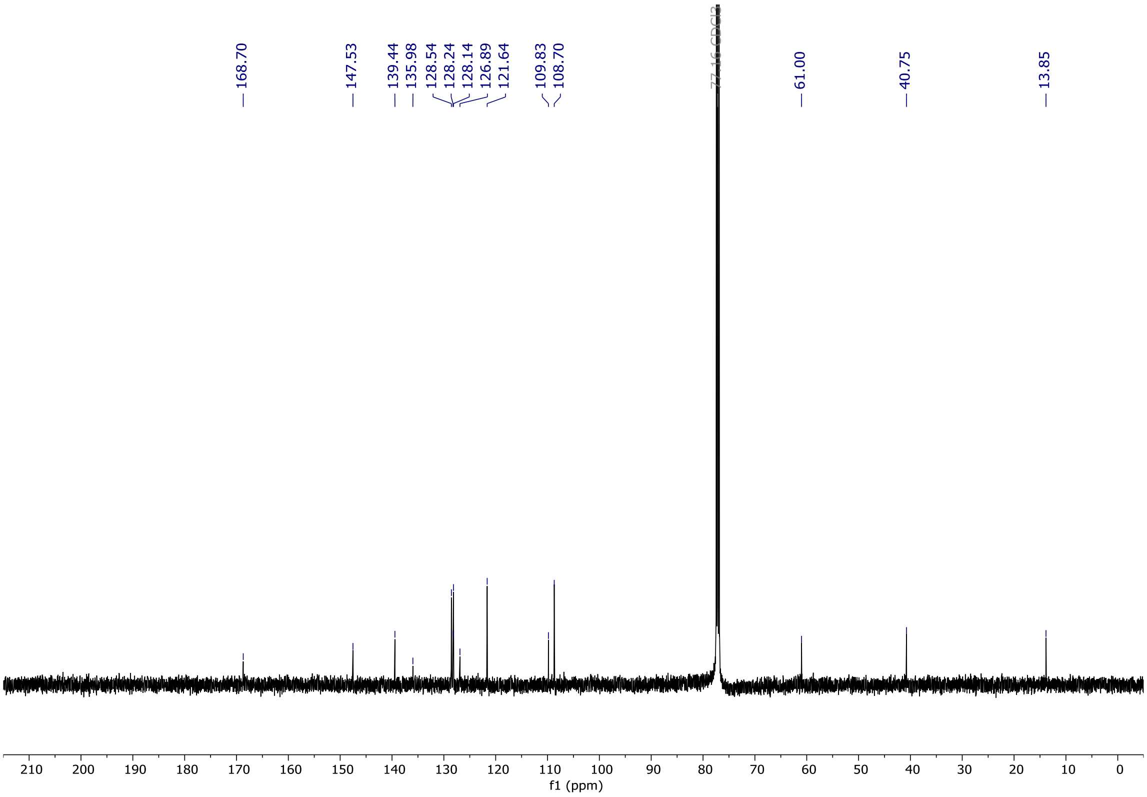

^{1}H NMR (400 MHz, $CDCl_3$) of compound **25** (major, E)

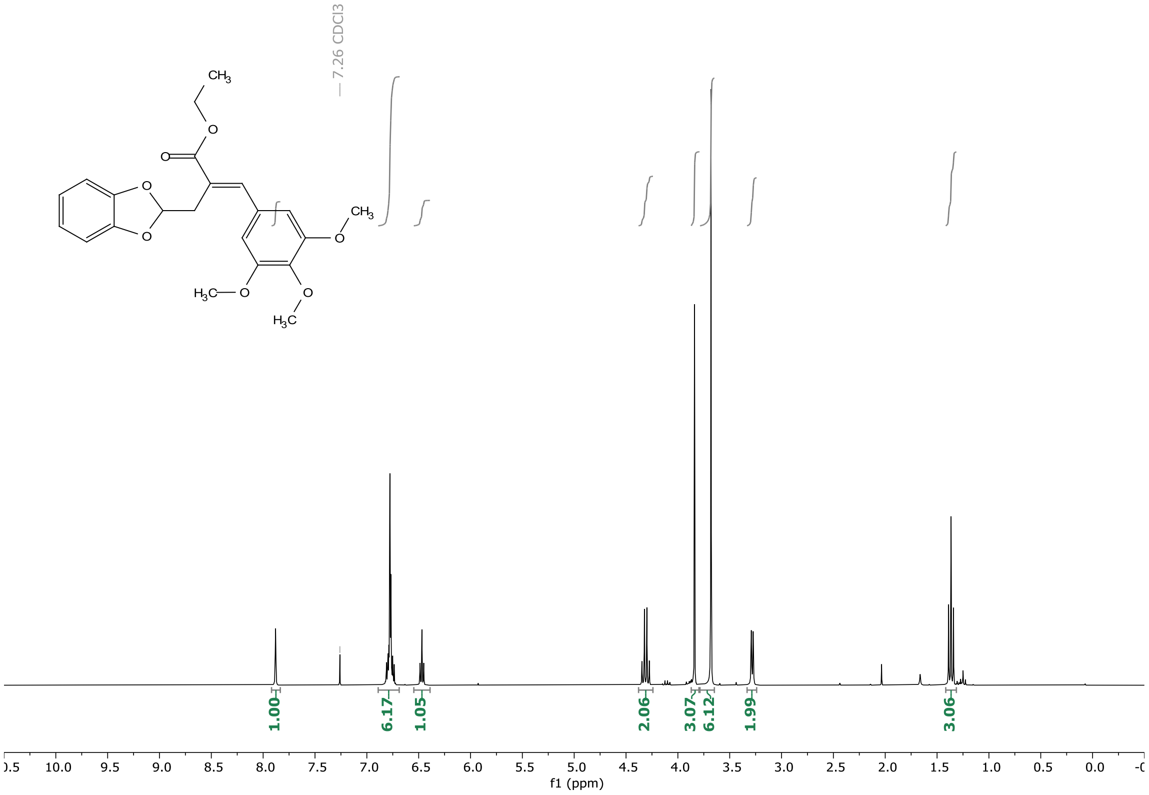

^{13}C NMR (101 MHz, $CDCl_3$) of compound **25** (major, E)

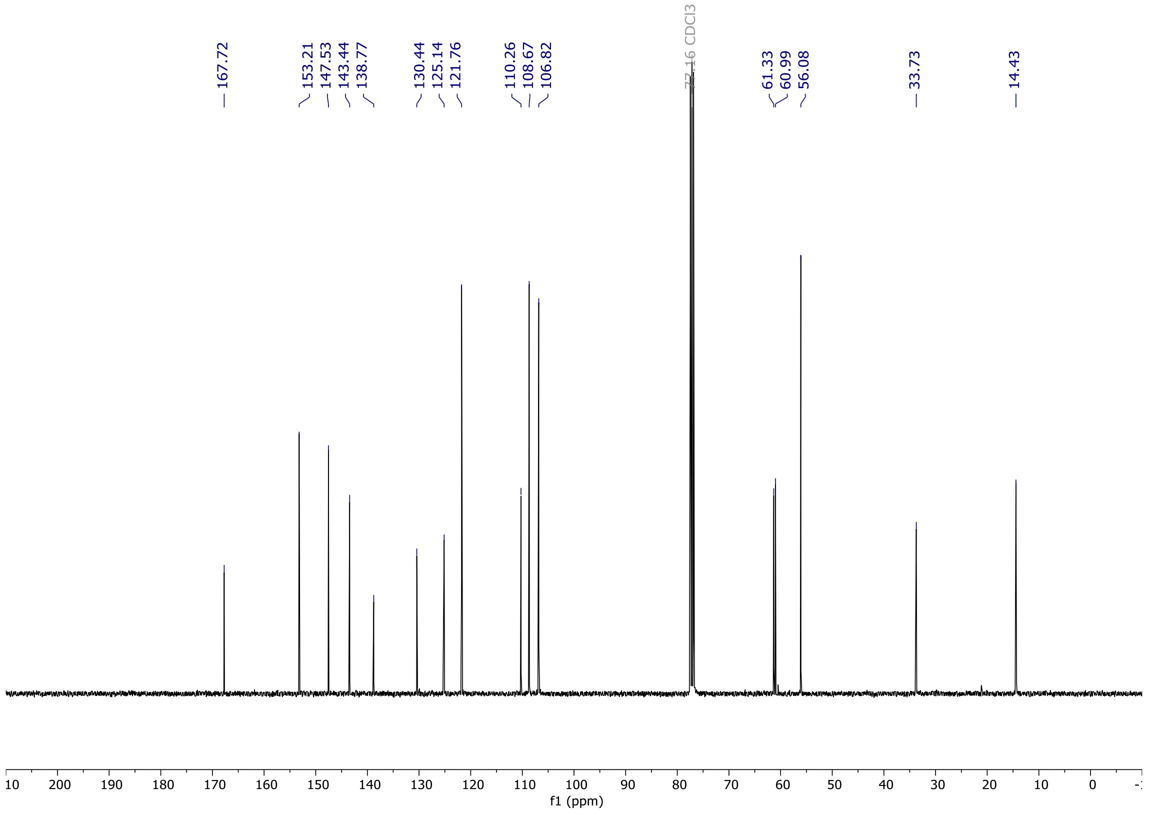

^{1}H–^{1}H NOESY (400 MHz, $CDCl_3$) of compound **25** (major, E)

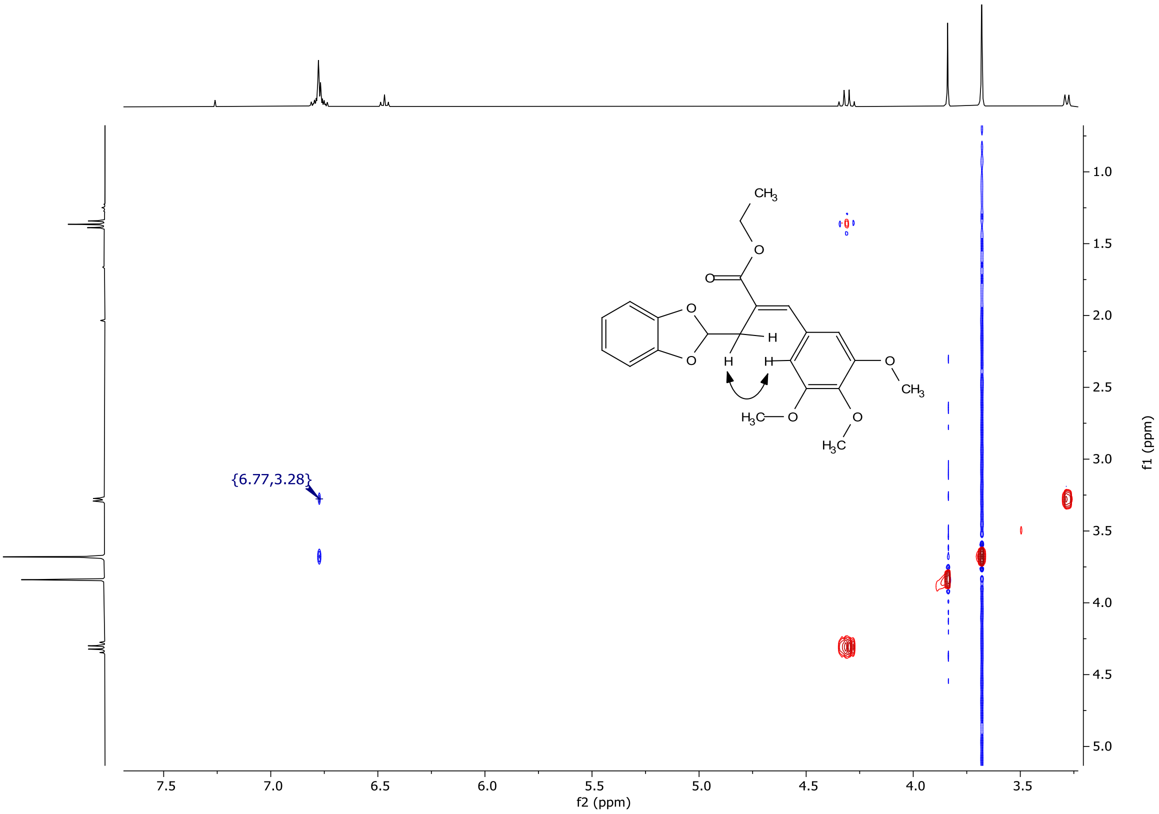

^{1}H NMR (400 MHz, $CDCl_3$) of compound **25** (minor, Z)

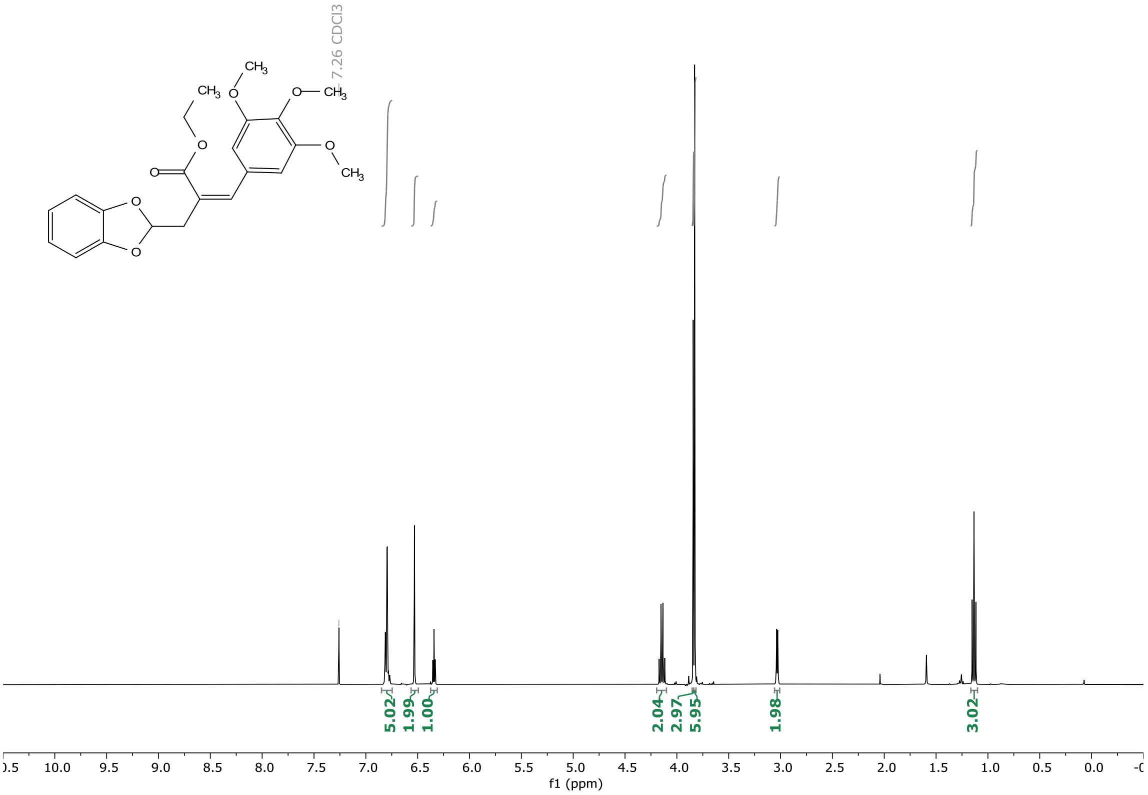

^{13}C NMR (101 MHz, $CDCl_3$) of compound **25** (minor, Z)

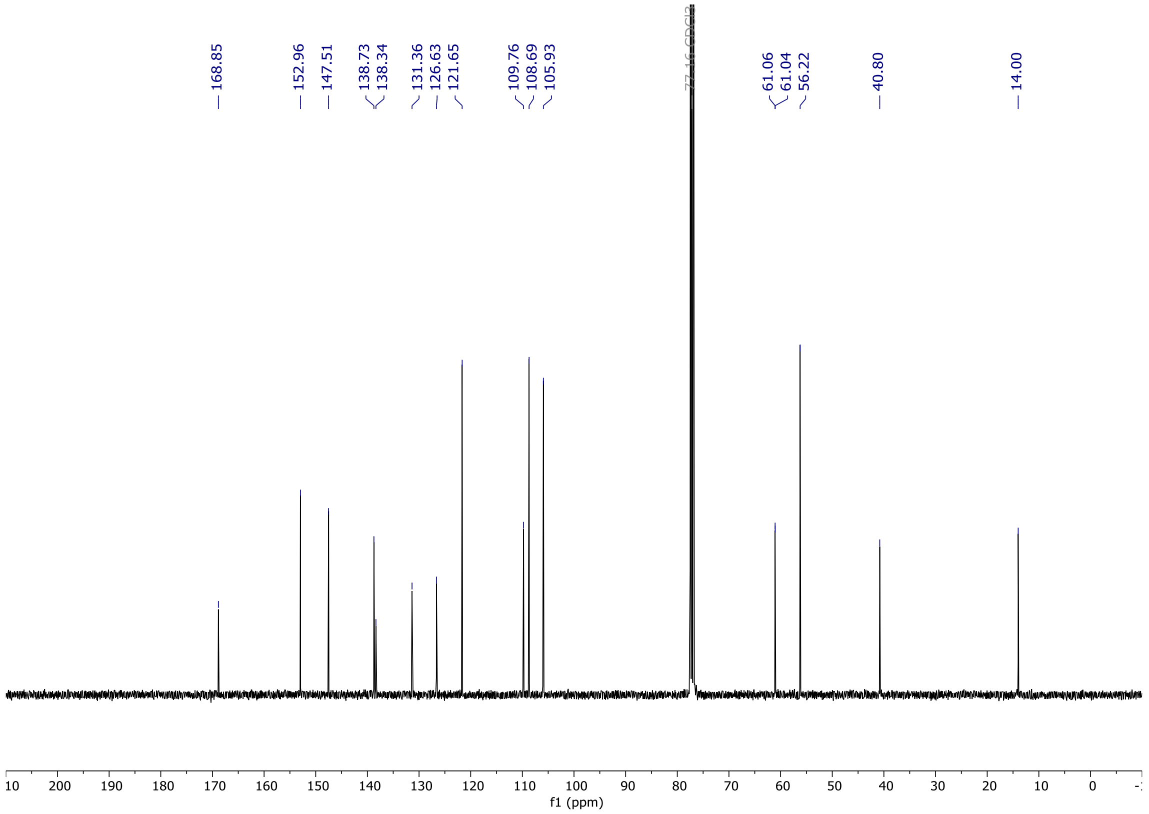

^{1}H–^{1}H NOESY (400 MHz, $CDCl_3$) of compound **25** (minor, Z)

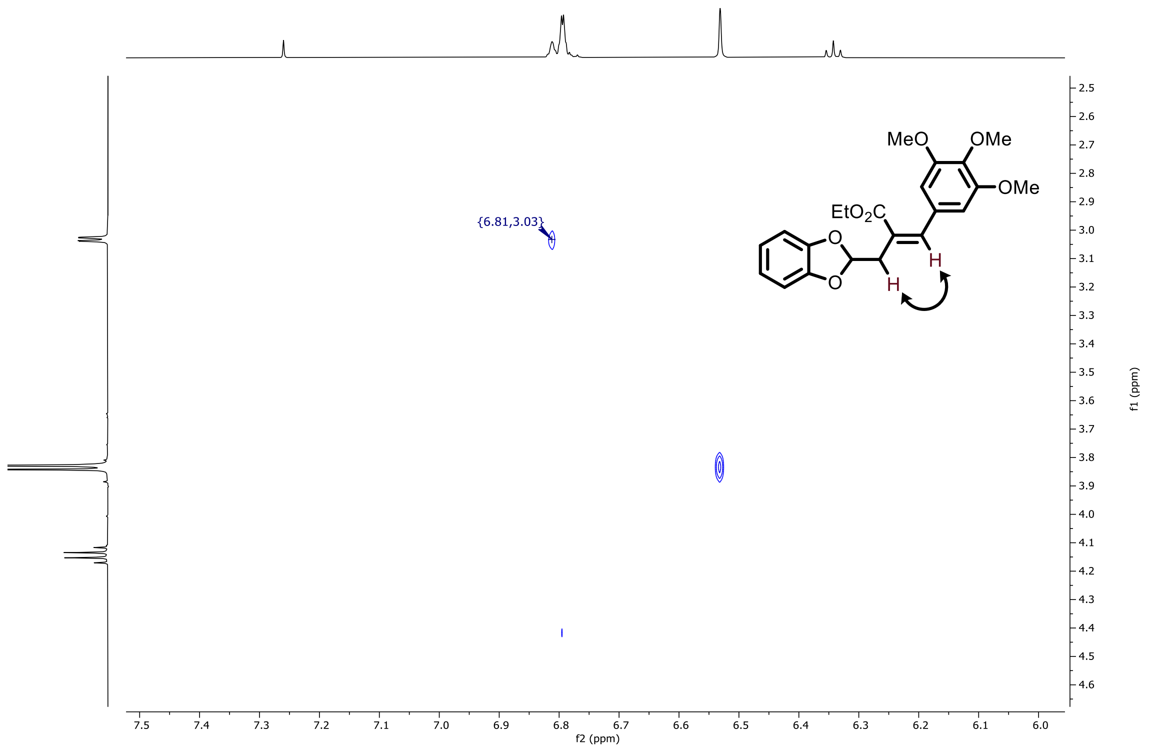

^{1}H NMR (400 MHz, $CDCl_3$) of compound **26** (isomer 1, E)

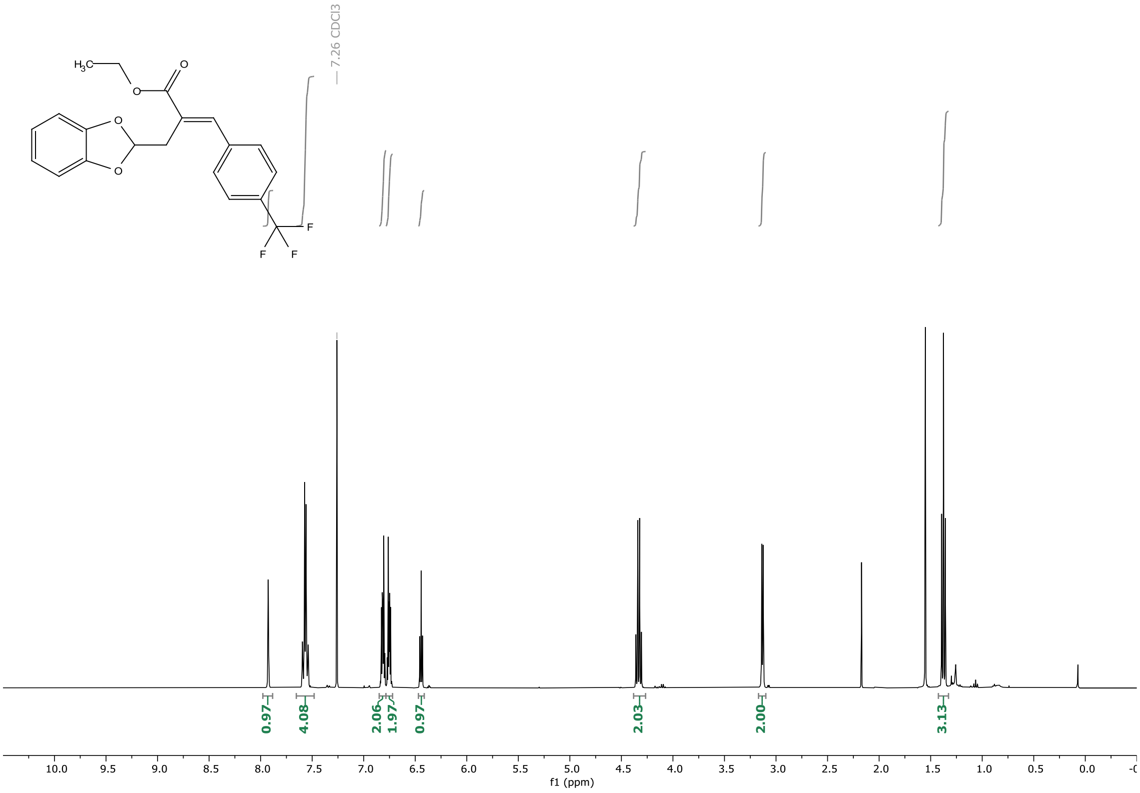

^{13}C NMR (101 MHz, $CDCl_3$) of compound **26** (isomer 1, E)

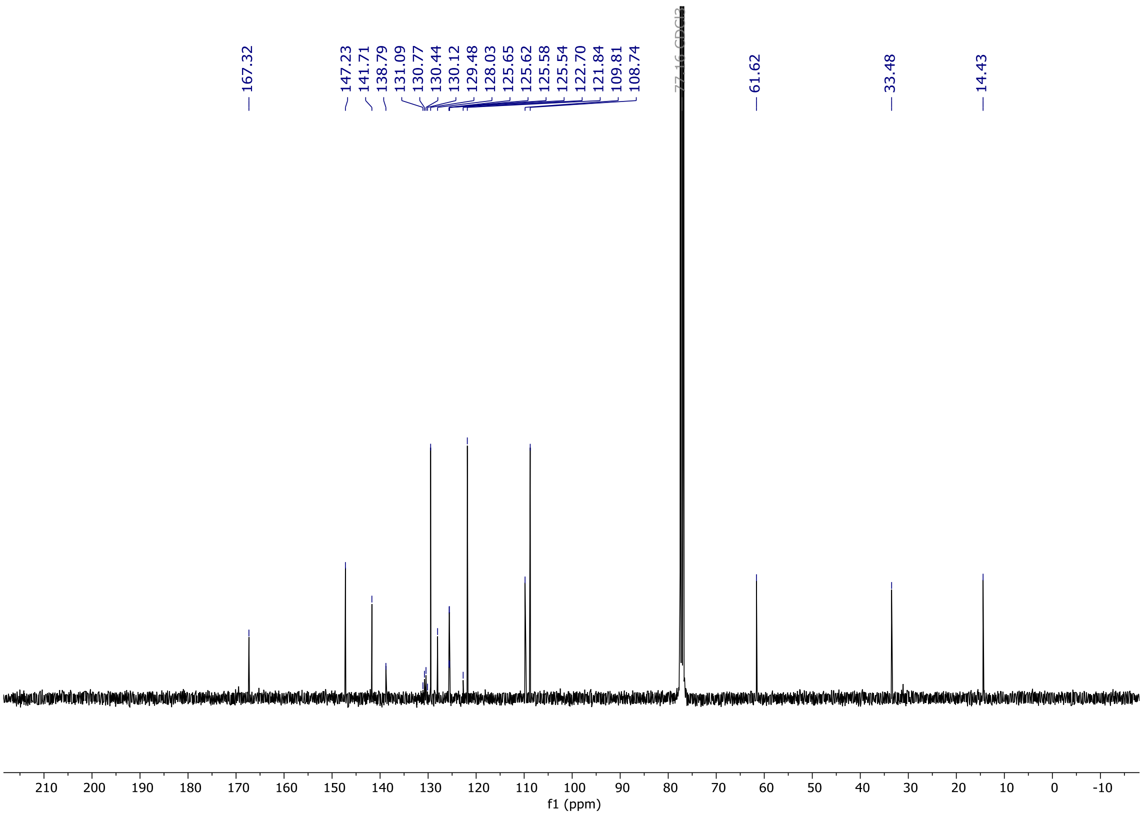

^{19}F NMR (376 MHz, $CDCl_3$) of compound **26** (isomer 1, E)

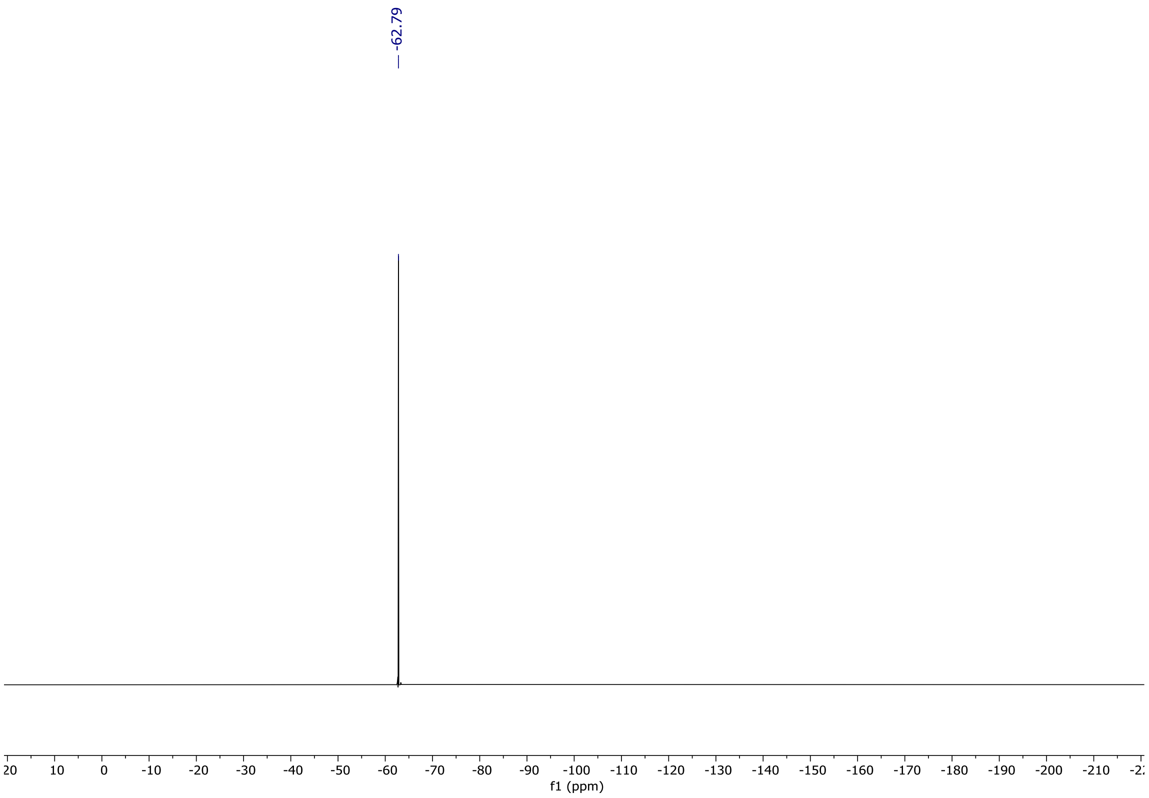

^{1}H NMR (400 MHz, $CDCl_3$) of compound **26** (isomer 2, Z)

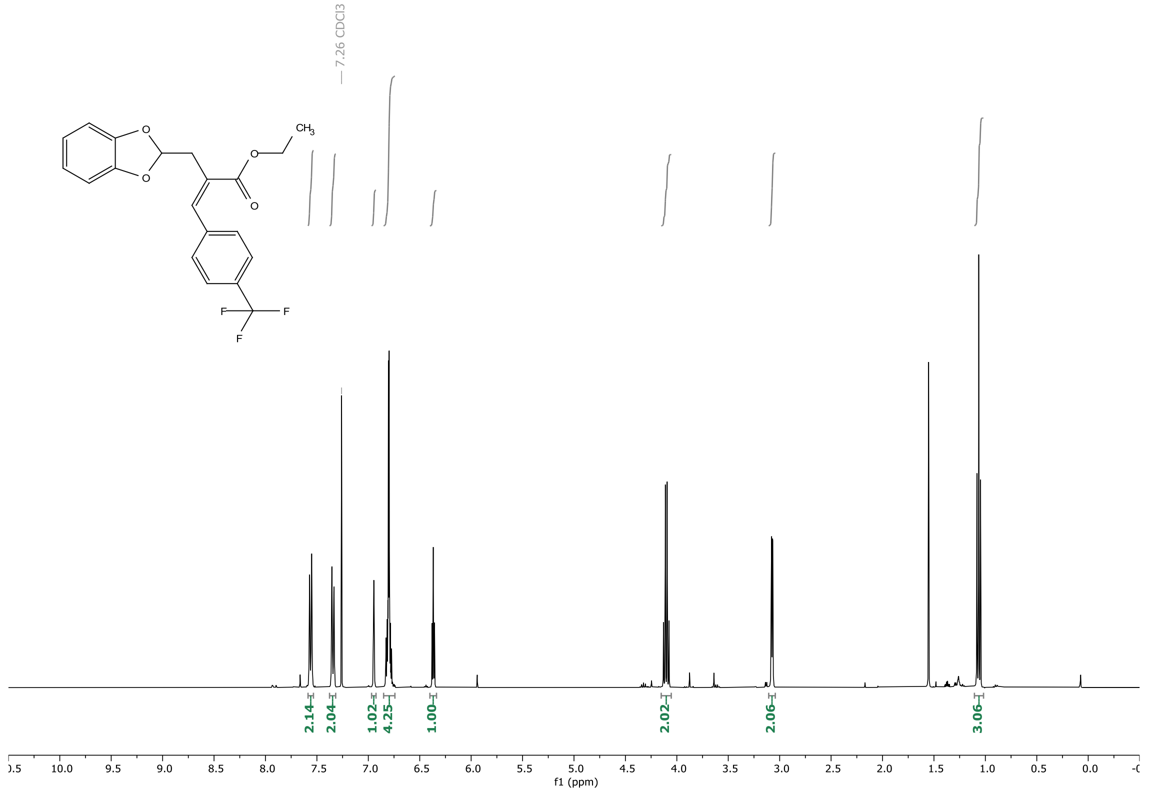

^{13}C NMR (101 MHz, $CDCl_3$) of compound **26** (isomer 2, Z)

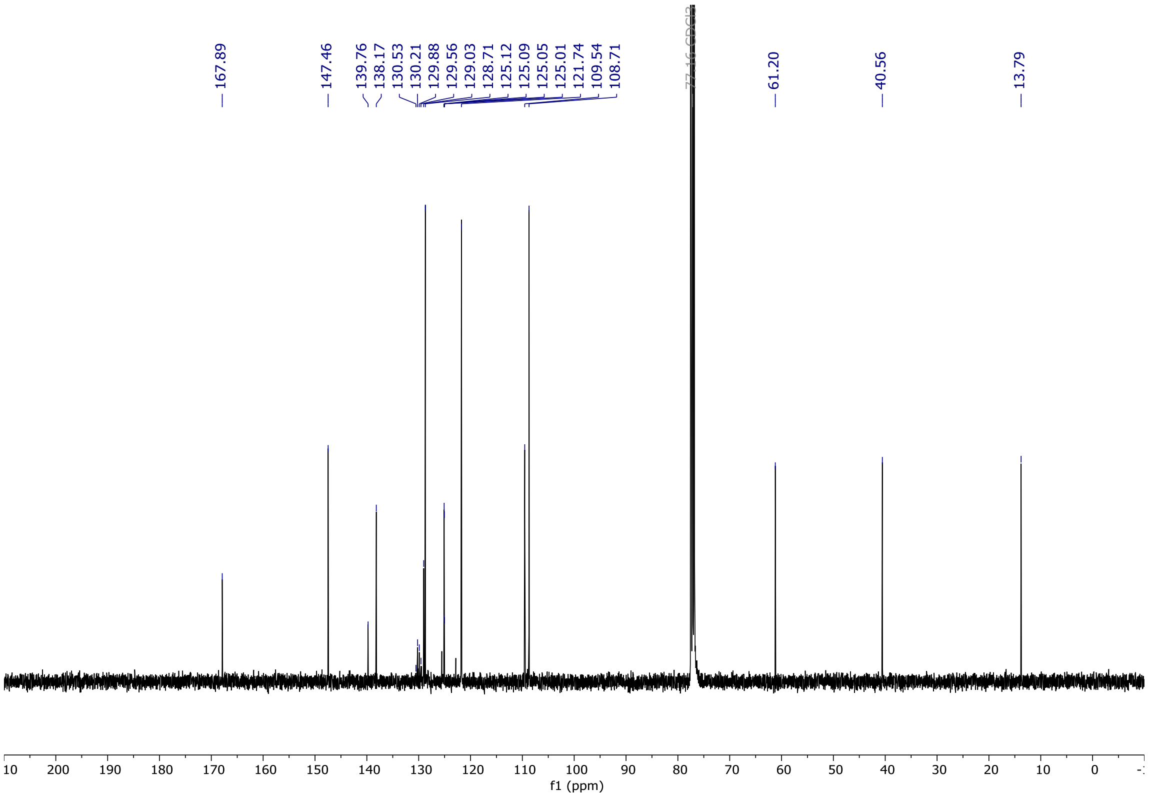

^{19}F NMR (376 MHz, $CDCl_3$) of compound **26** (isomer 2, Z)

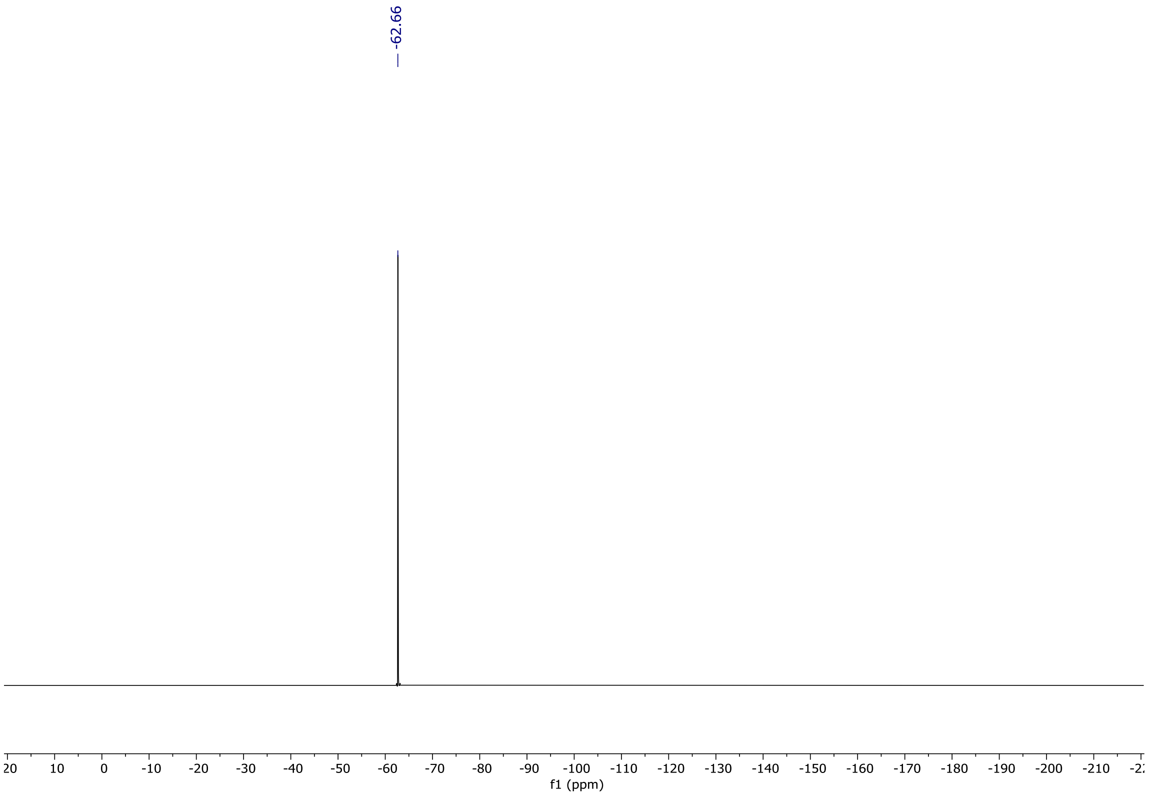

^{1}H NMR (400 MHz, $CDCl_3$) of compound **27** (isomer 1, E)

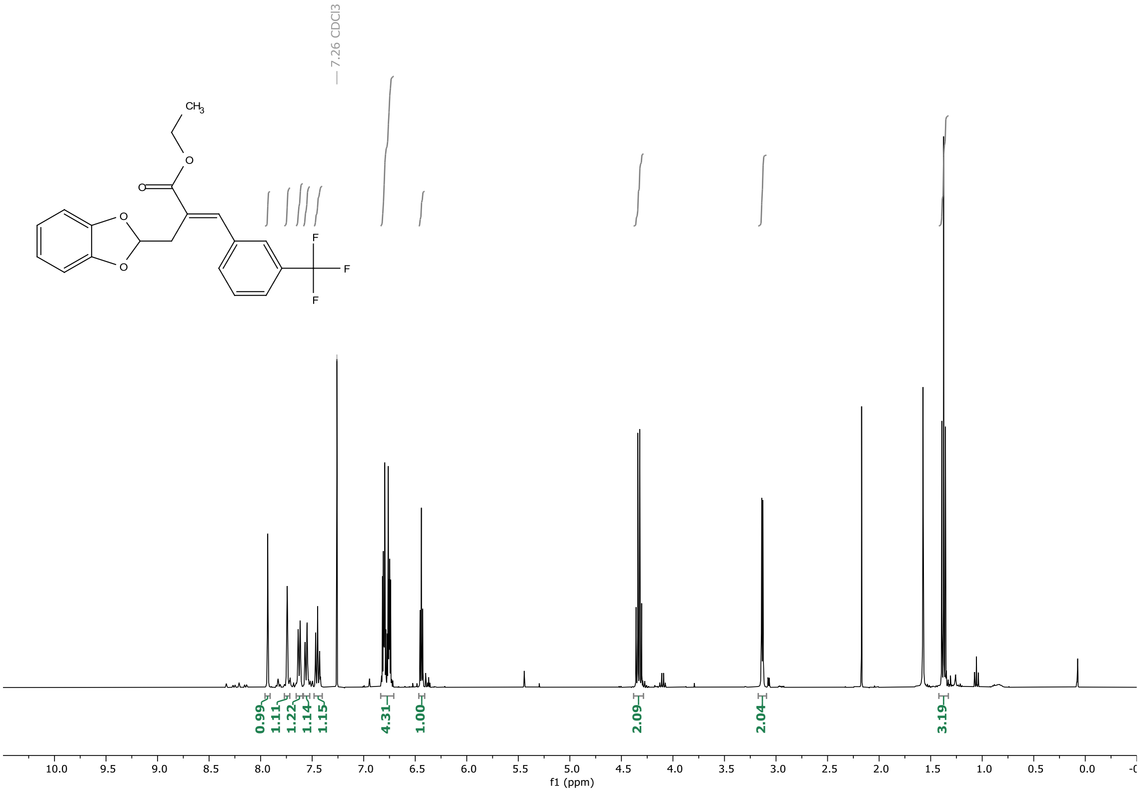

^{13}C NMR (101 MHz, $CDCl_3$) of compound **27** (isomer 1, E)

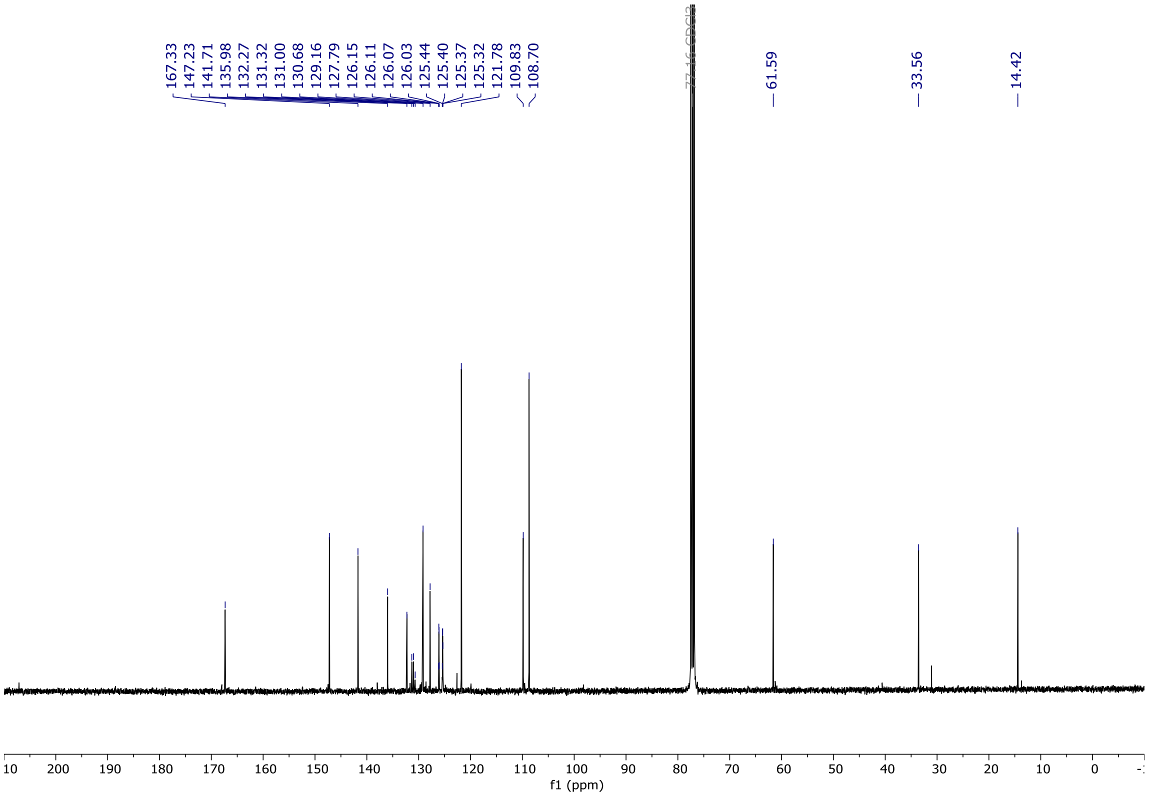

^{19}F NMR (376 MHz, $CDCl_3$) of compound **27** (isomer 1, E)

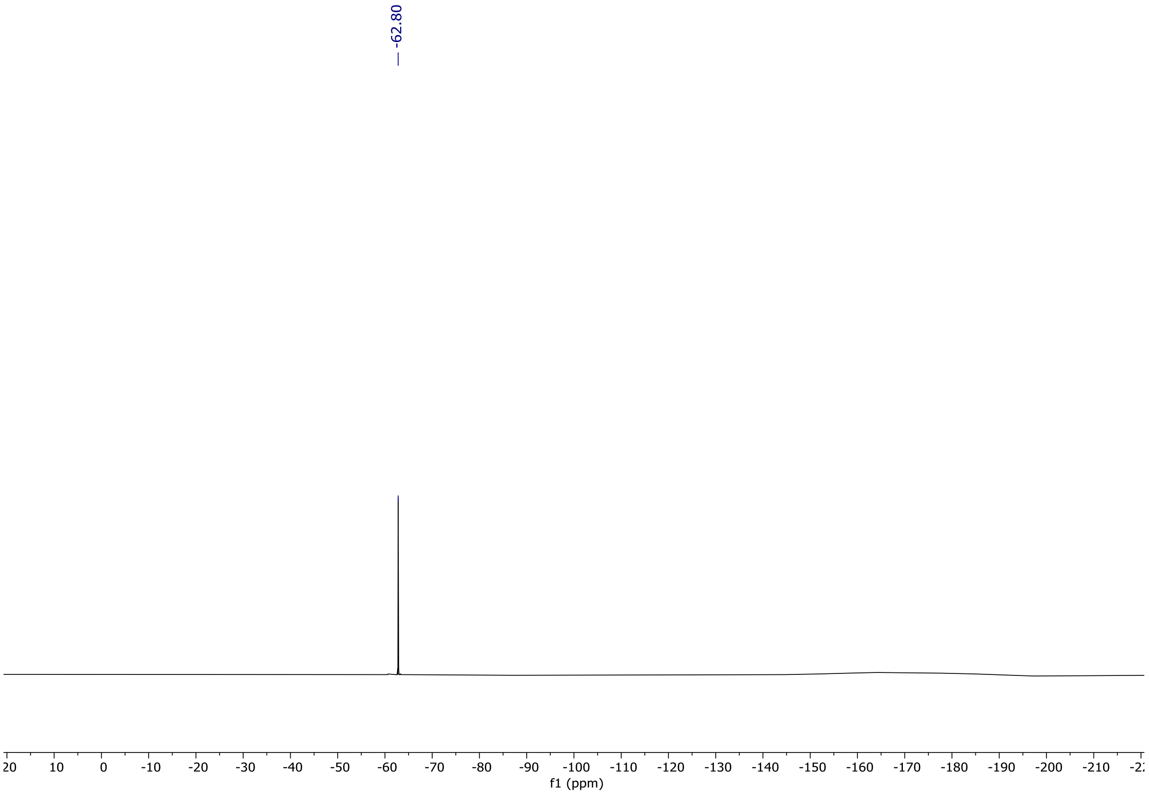

^{1}H NMR (400 MHz, $CDCl_3$) of compound **27** (isomer 2, Z)

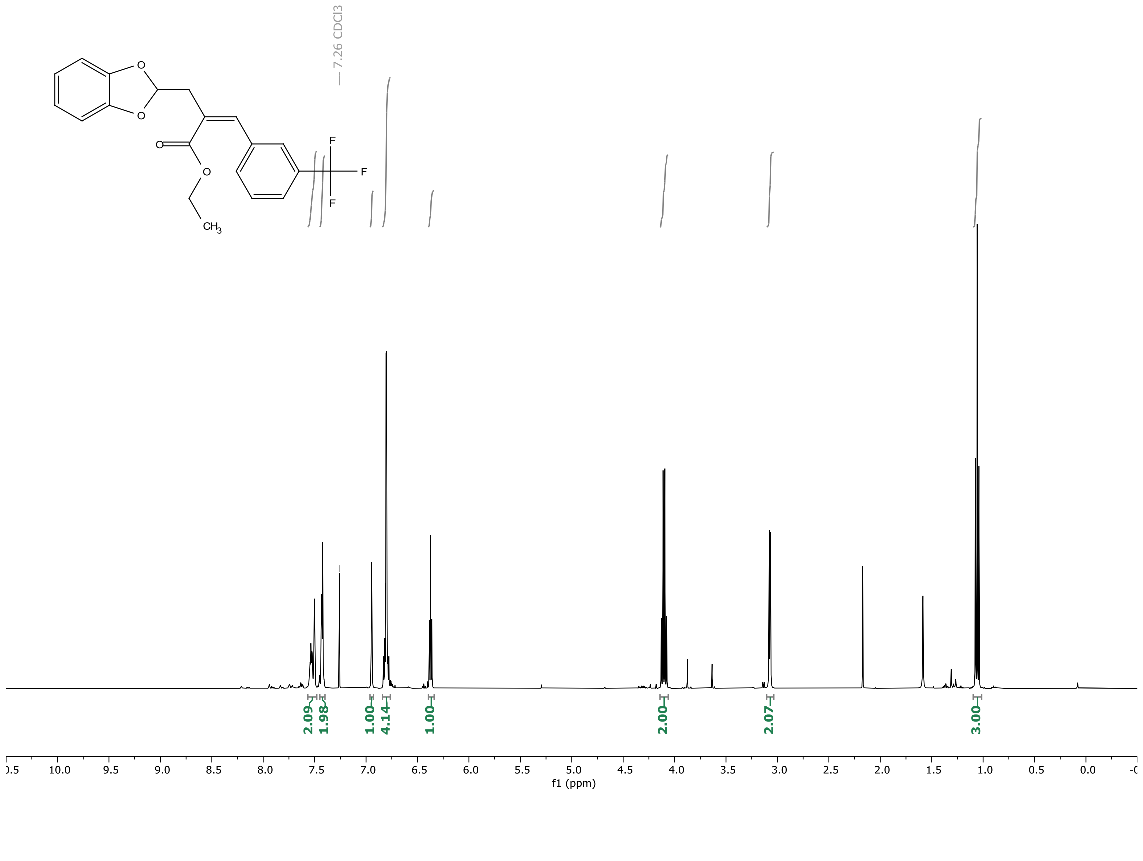

^{13}C NMR (100 MHz, $CDCl_3$) of compound **27** (isomer 2, Z)

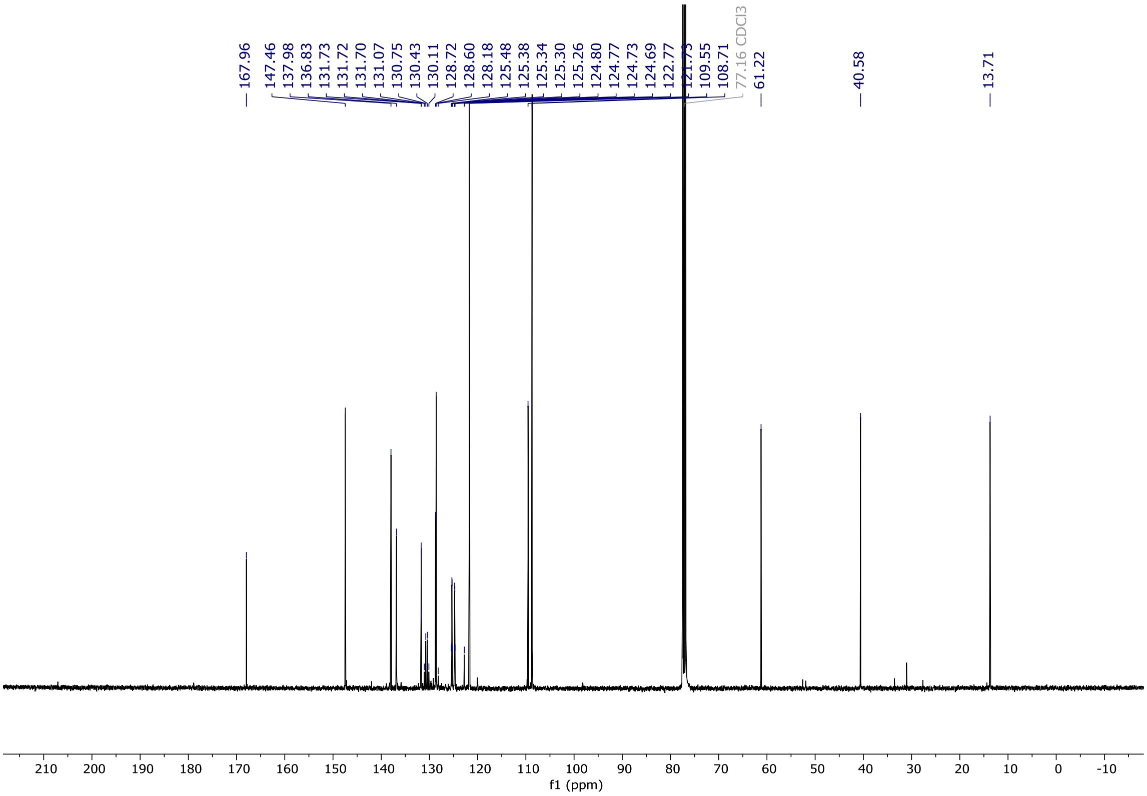

^{19}F NMR (376 MHz, $CDCl_3$) of compound **27** (isomer 2, Z)

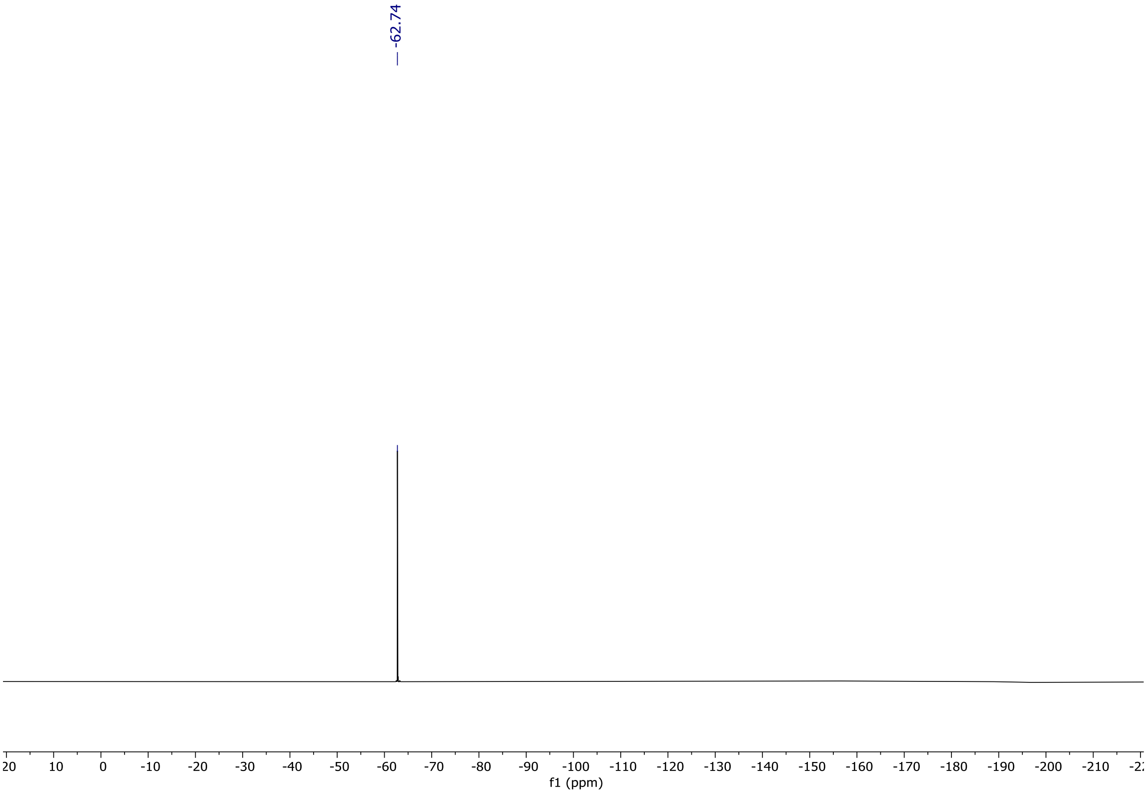

^{1}H NMR (400 MHz, $CDCl_3$) of compound **28** (major, E)

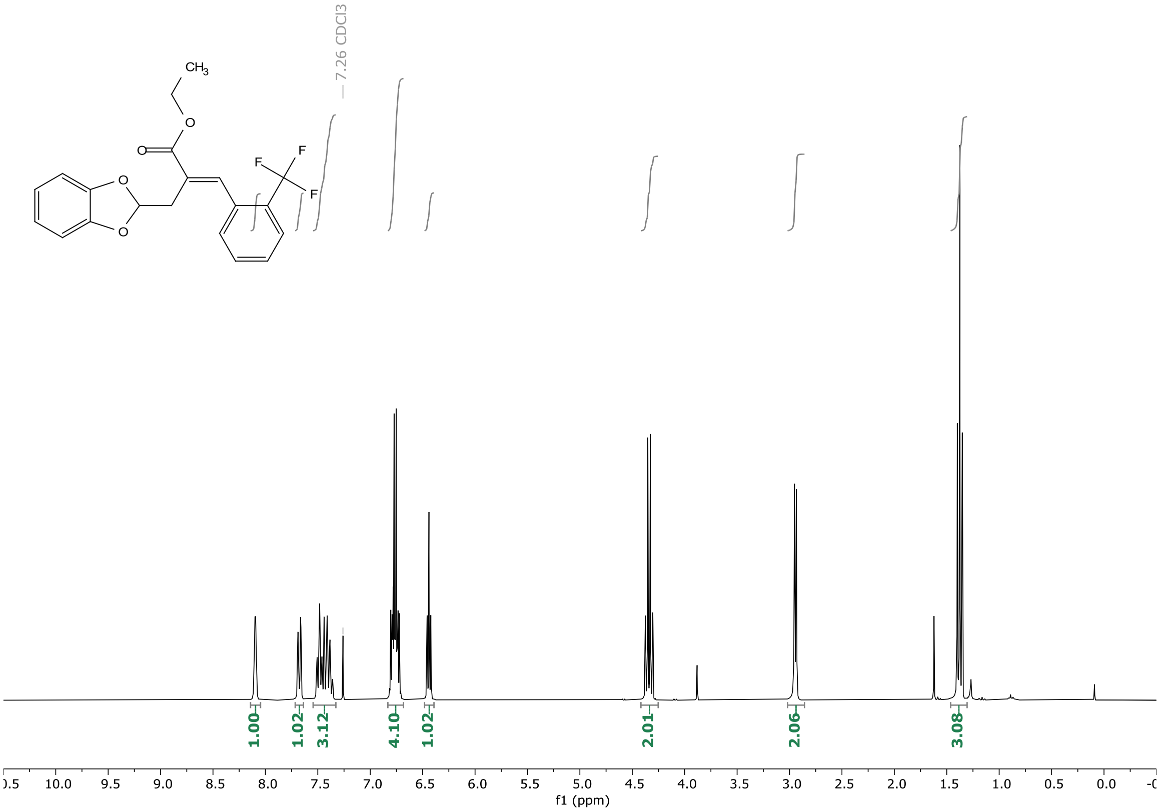

^{13}C NMR (101 MHz, $CDCl_3$) of compound **28** (major, E)

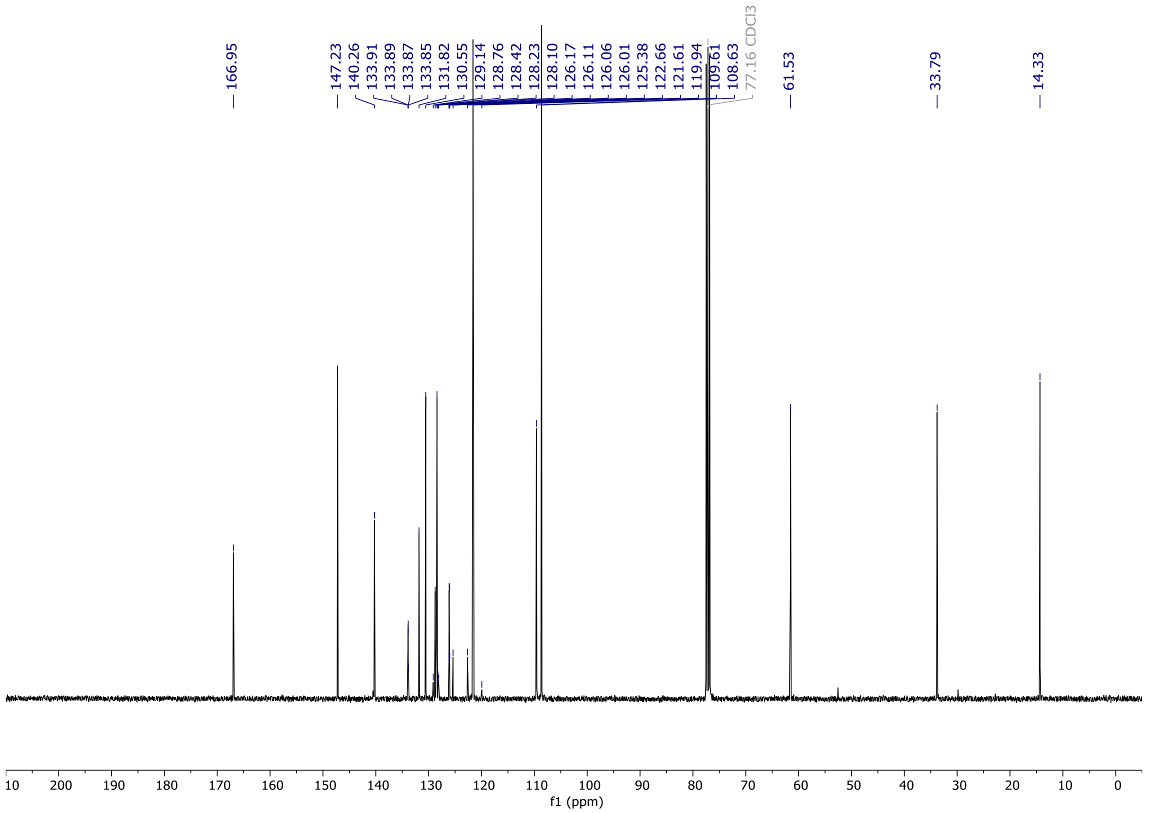

^{19}F NMR (376 MHz, $CDCl_3$) of compound **28** (major, E)

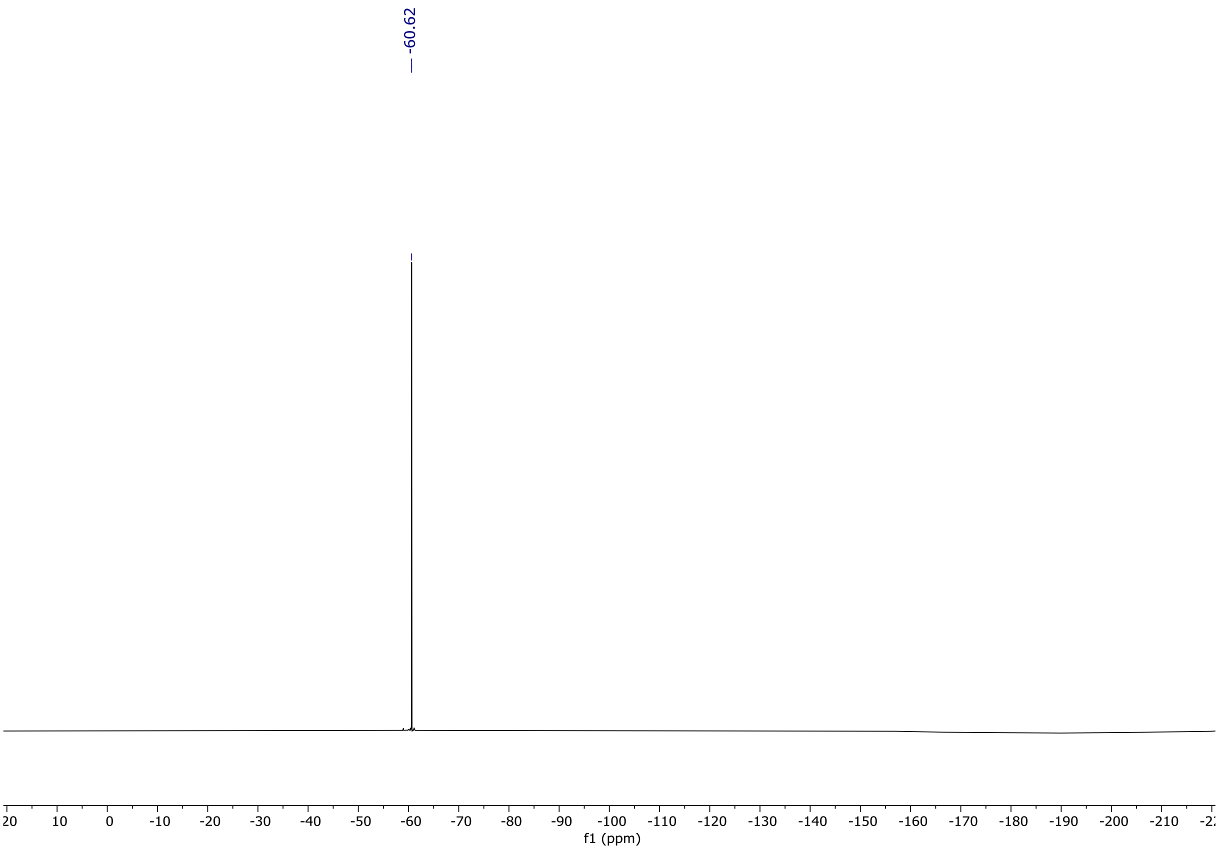

^1H–^1H NOESY (400 MHz, $CDCl_3$) of compound **28** (major, E)

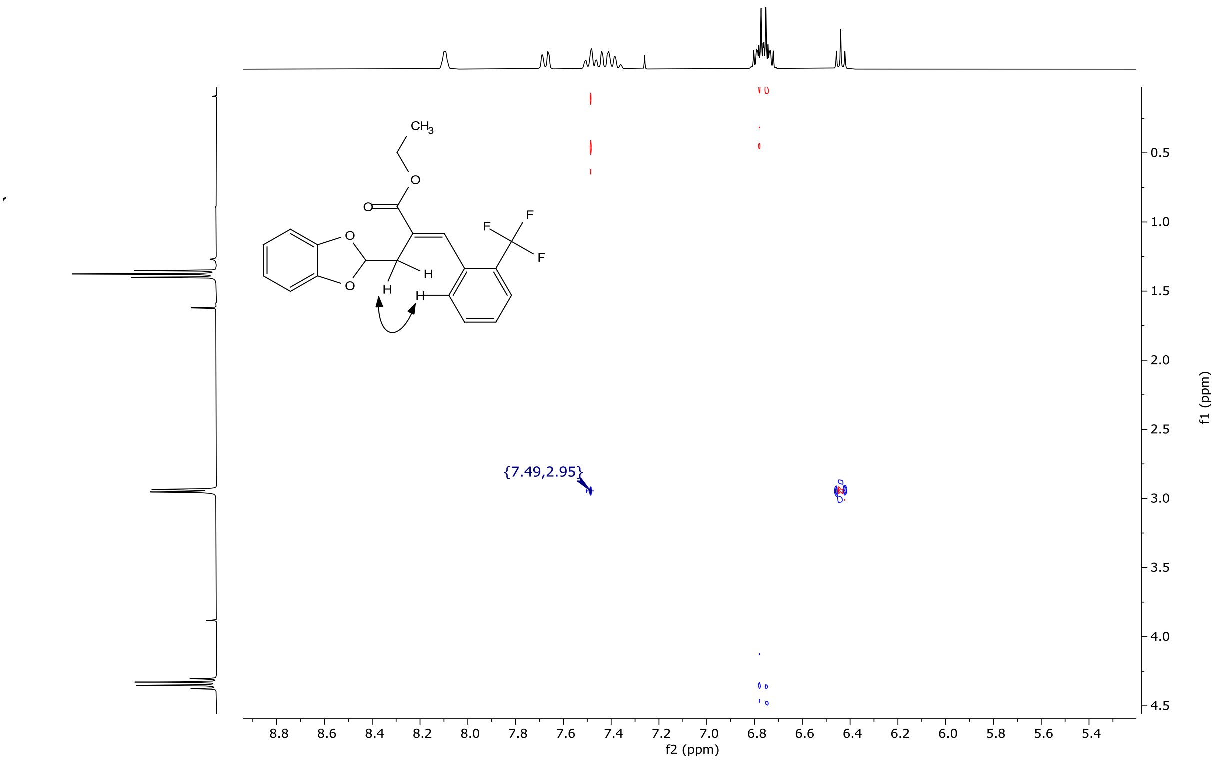

^{1}H NMR (400 MHz, $CDCl_3$) of compound **29** (major, E)

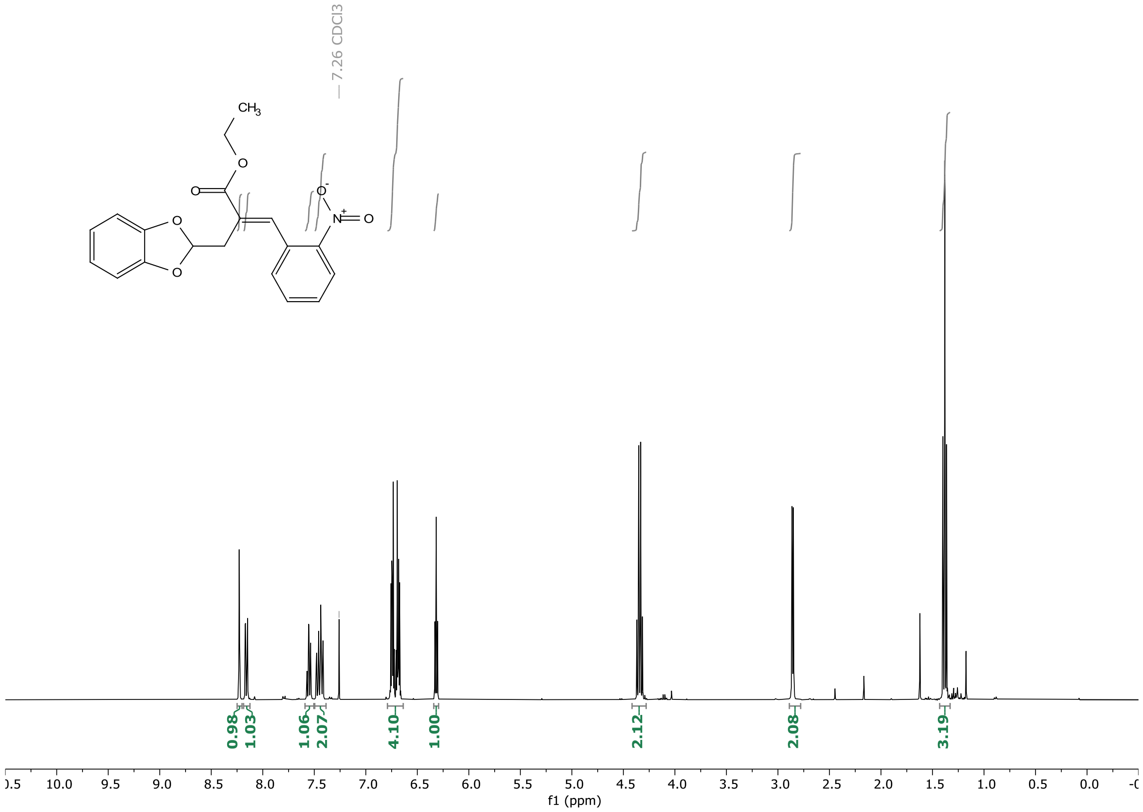

^{13}C NMR (101 MHz, $CDCl_3$) of compound **29** (major, E)

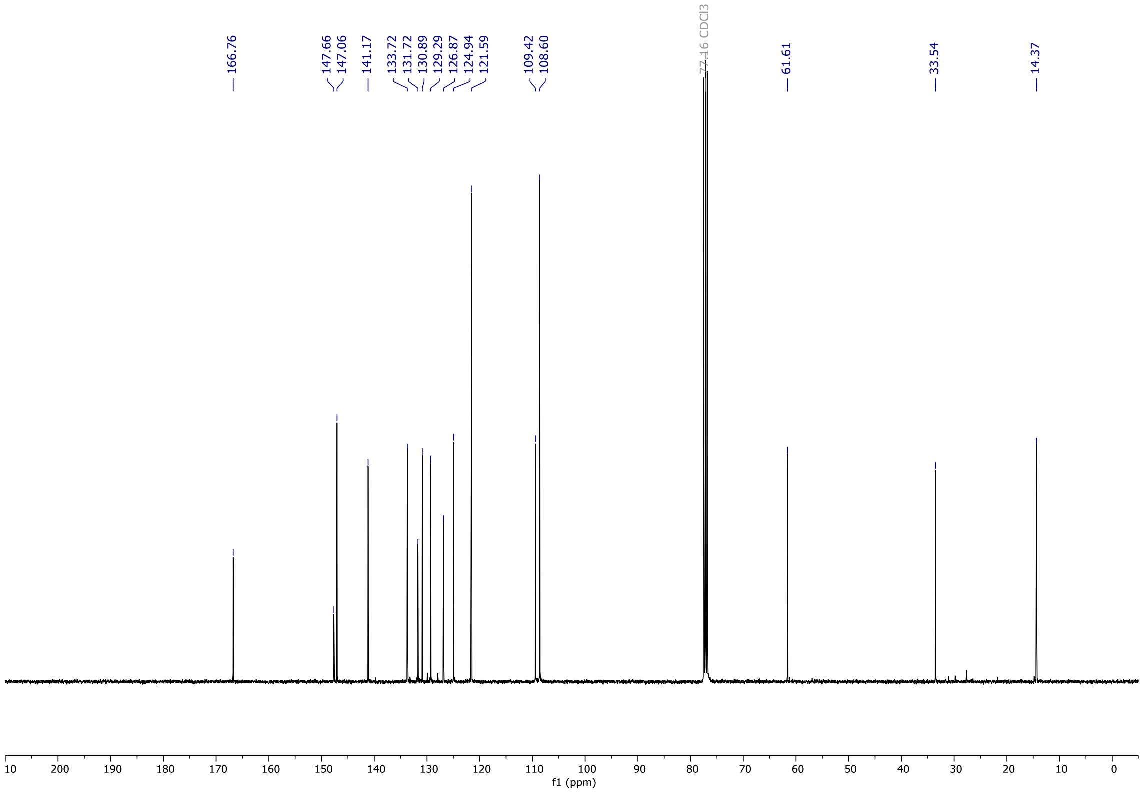

^{1}H–^{1}H NOESY (400 MHz, $CDCl_3$) of compound **29** (E isomer)

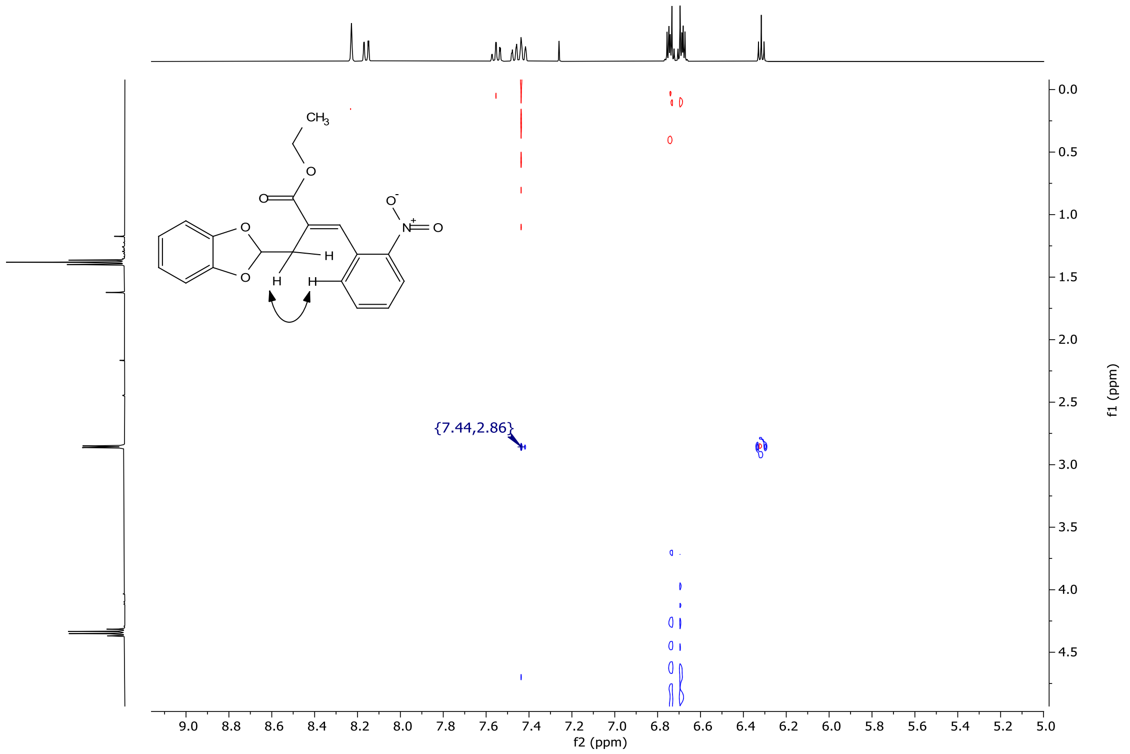

^{1}H (400 MHz, $CDCl_3$) of compound **30** (major, E)

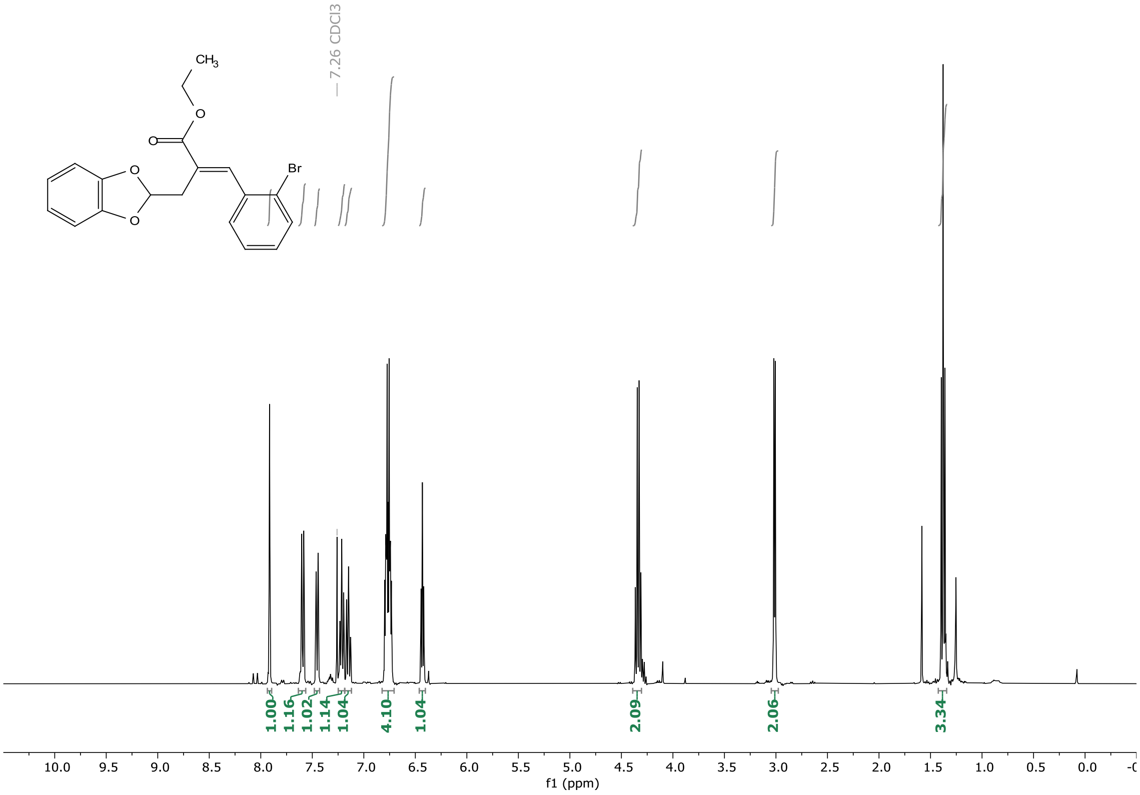

^{13}C NMR (101 MHz, $CDCl_3$) of compound **30** (major, E)

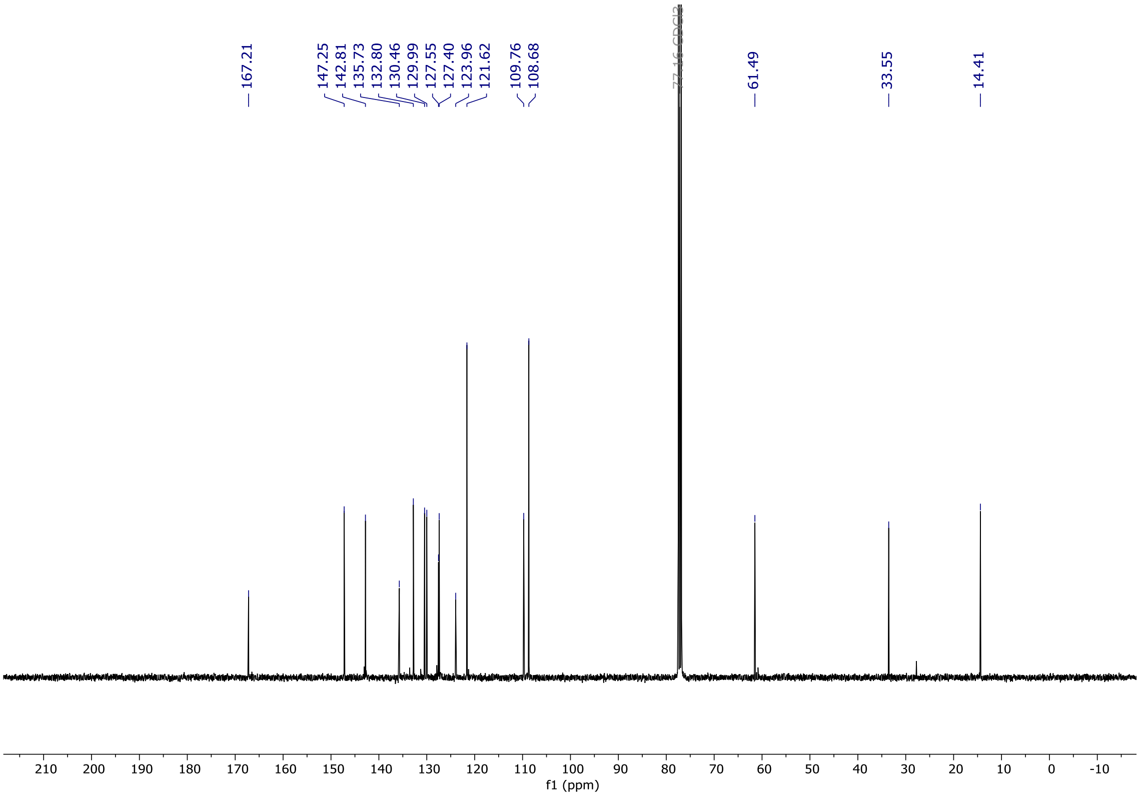

^{1}H–^{1}H NOESY (400 MHz, CDCl3) of compound **30** (major, E)

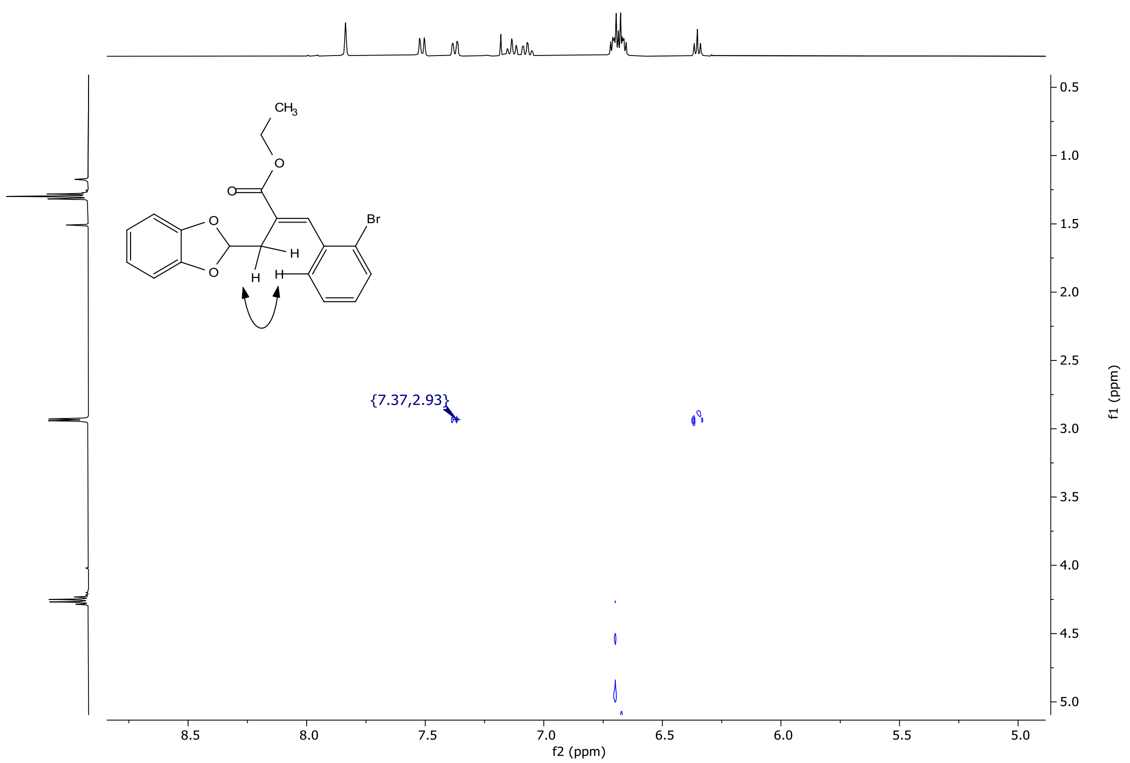

^{1}H (400 MHz, CDCl3) of compound **30** (minor, Z)

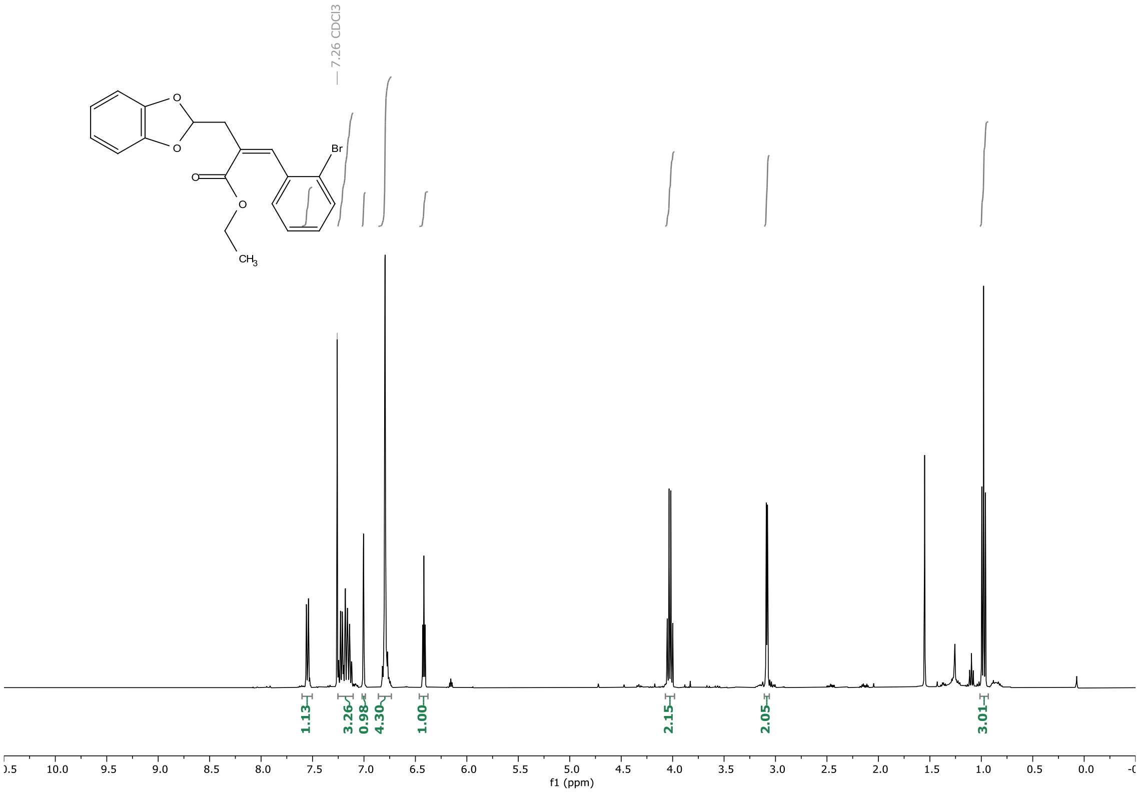

^{13}C NMR (101 MHz, $CDCl_3$) of compound **30** (minor, Z)

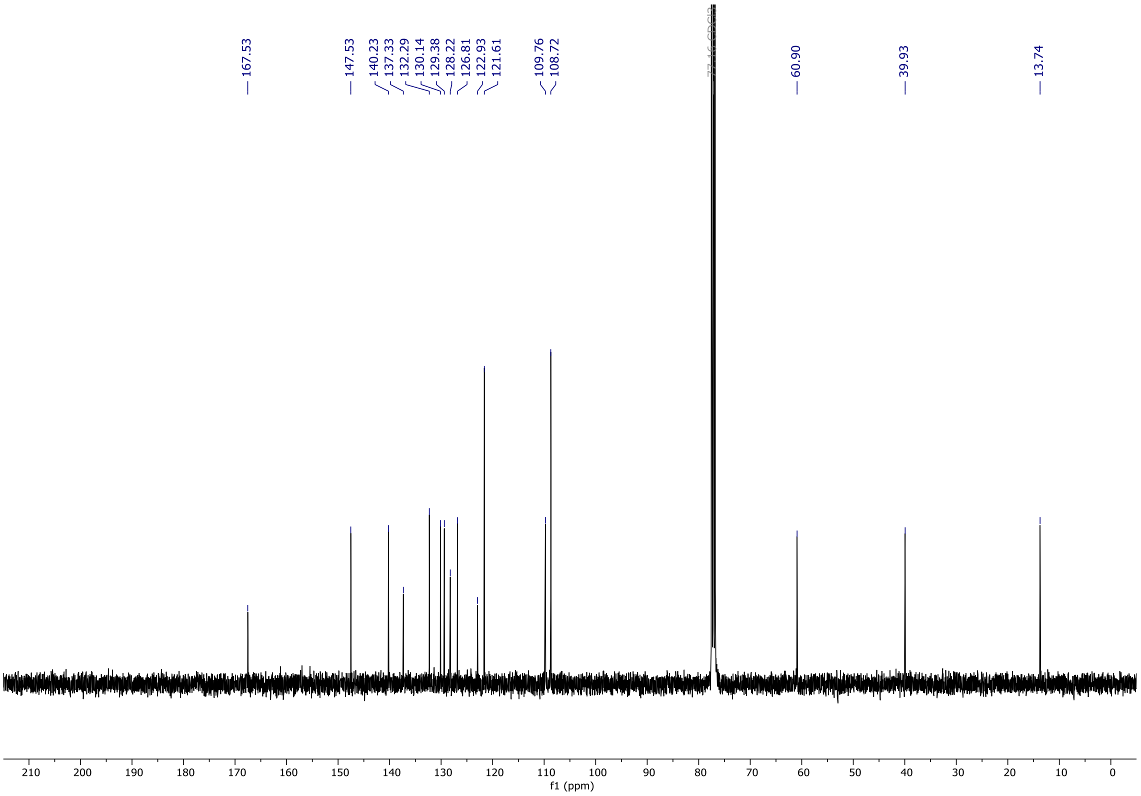

^1H–^1H NOESY (400 MHz, $CDCl_3$) of compound **30** (minor, Z)

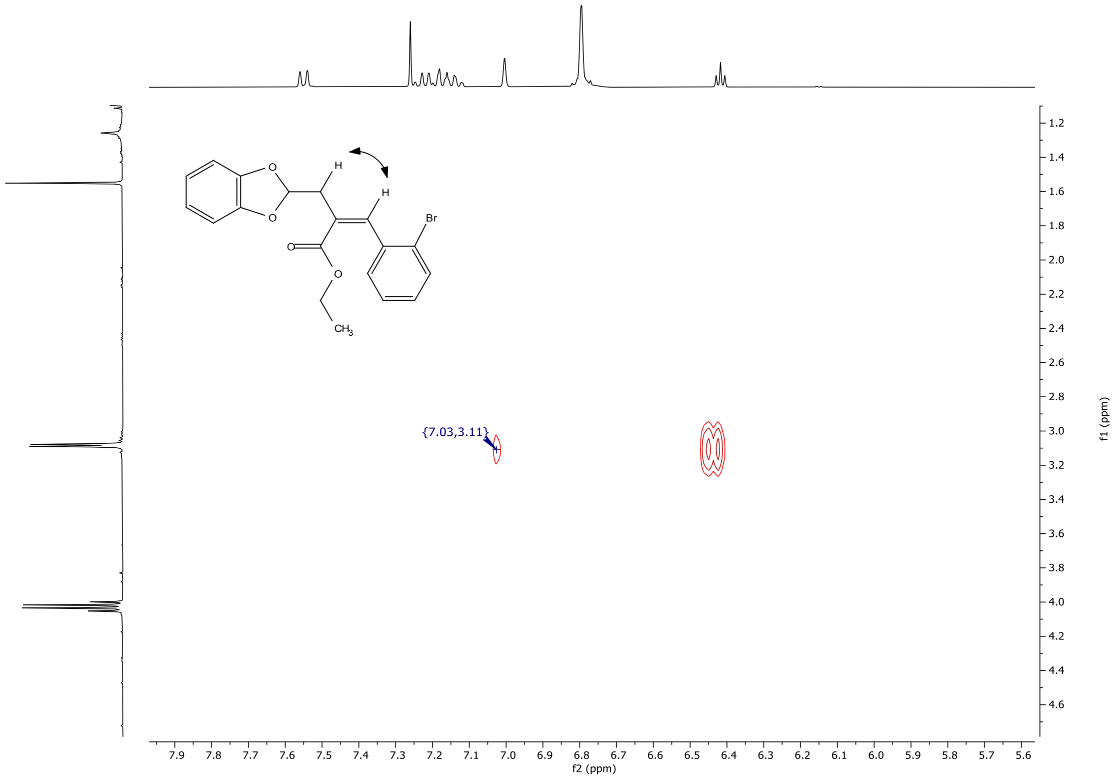

^{1}H NMR (400 MHz, $CDCl_3$) of compound **31** (major, E)

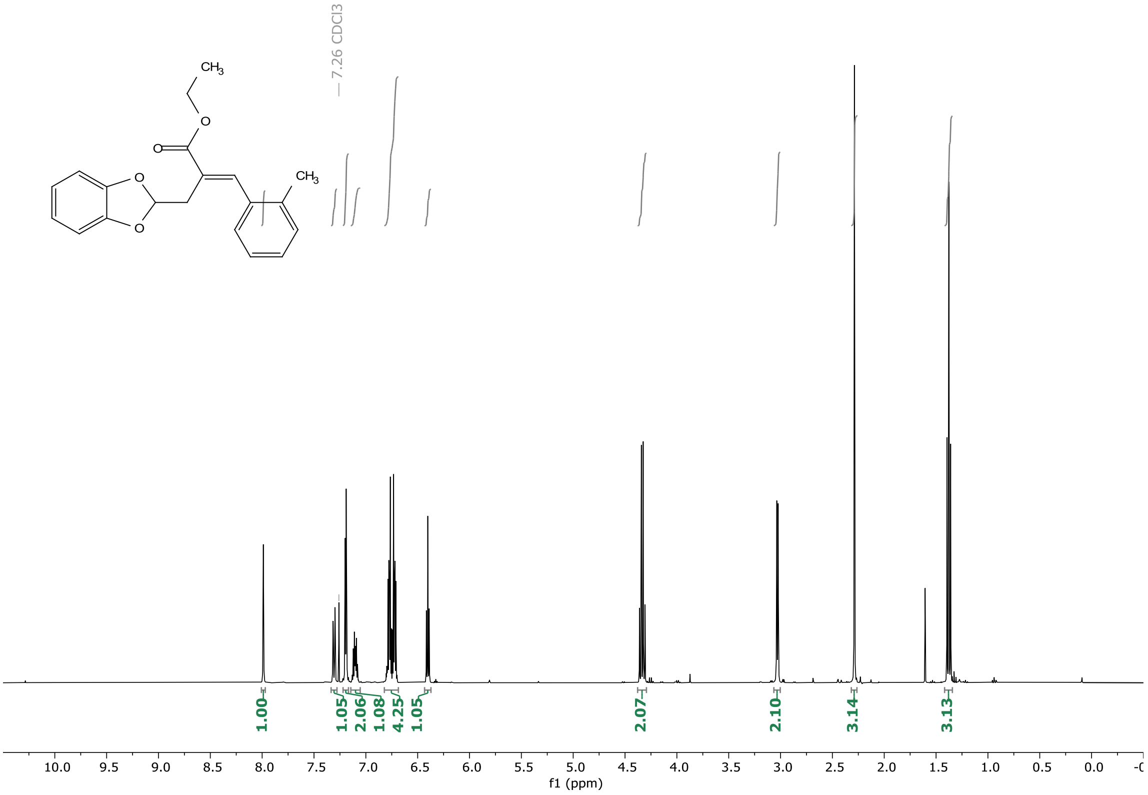

^{13}C NMR (101 MHz, $CDCl_3$) of compound **31** (major, E)

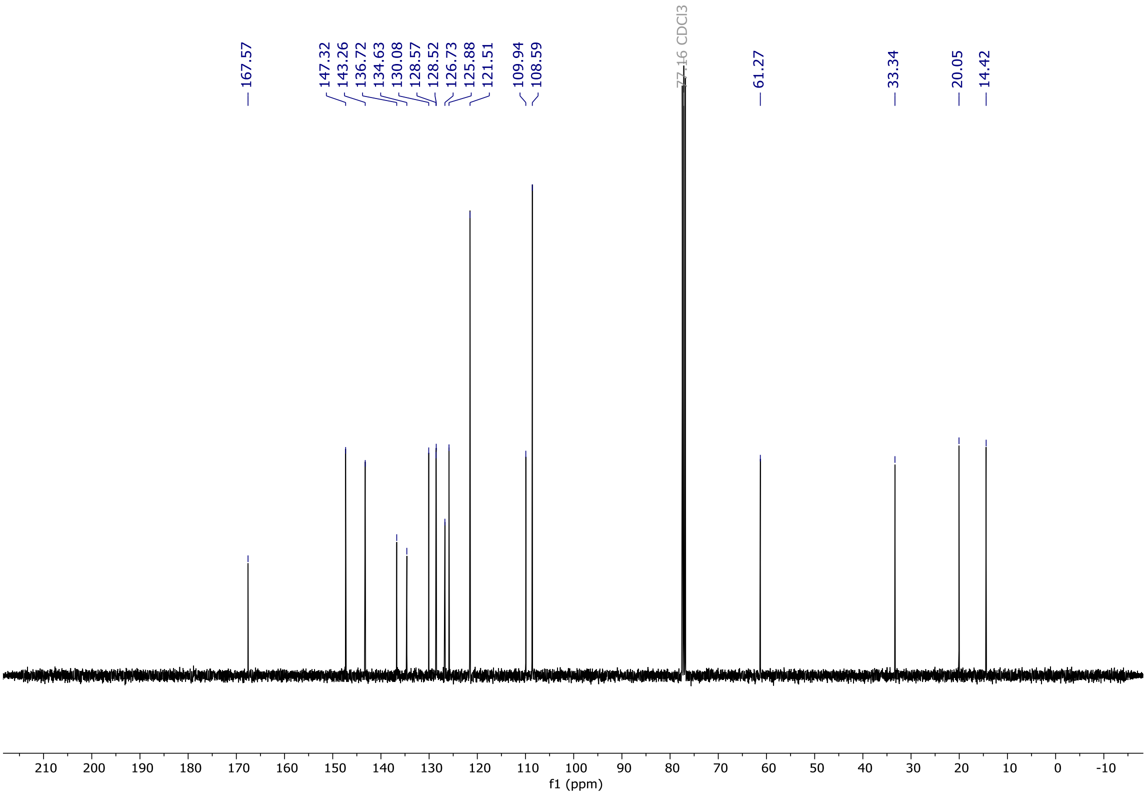

^{1}H–^{1}H NOESY (400 MHz, CDCl$_3$) of compound **31** (major, E)

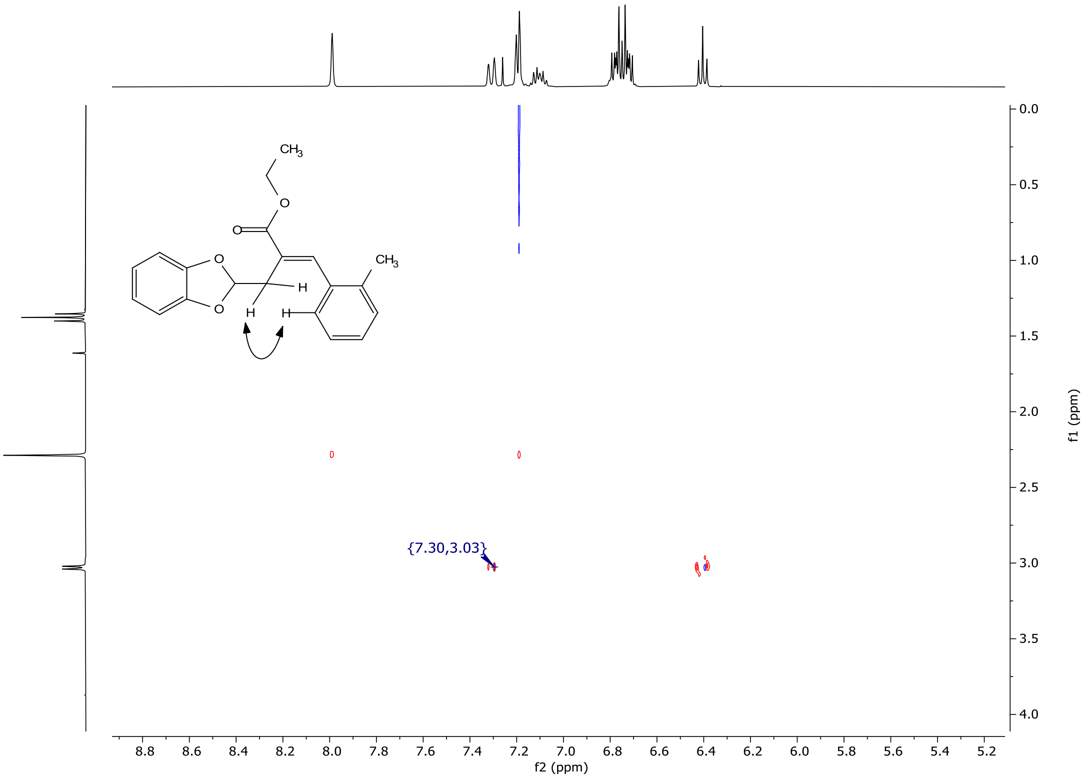

^{1}H NMR (400 MHz, $CDCl_3$) of compound **32** (major, E)

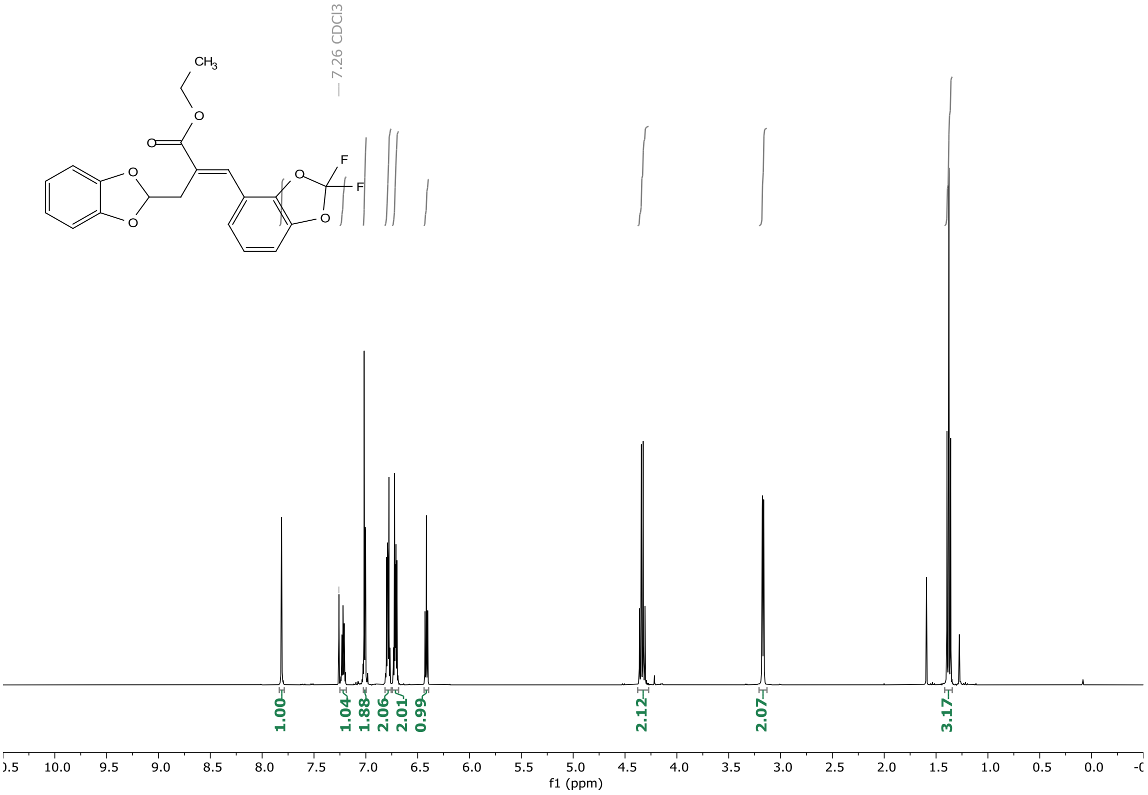

^{13}C NMR (101 MHz, $CDCl_3$) of compound **32** (major, E)

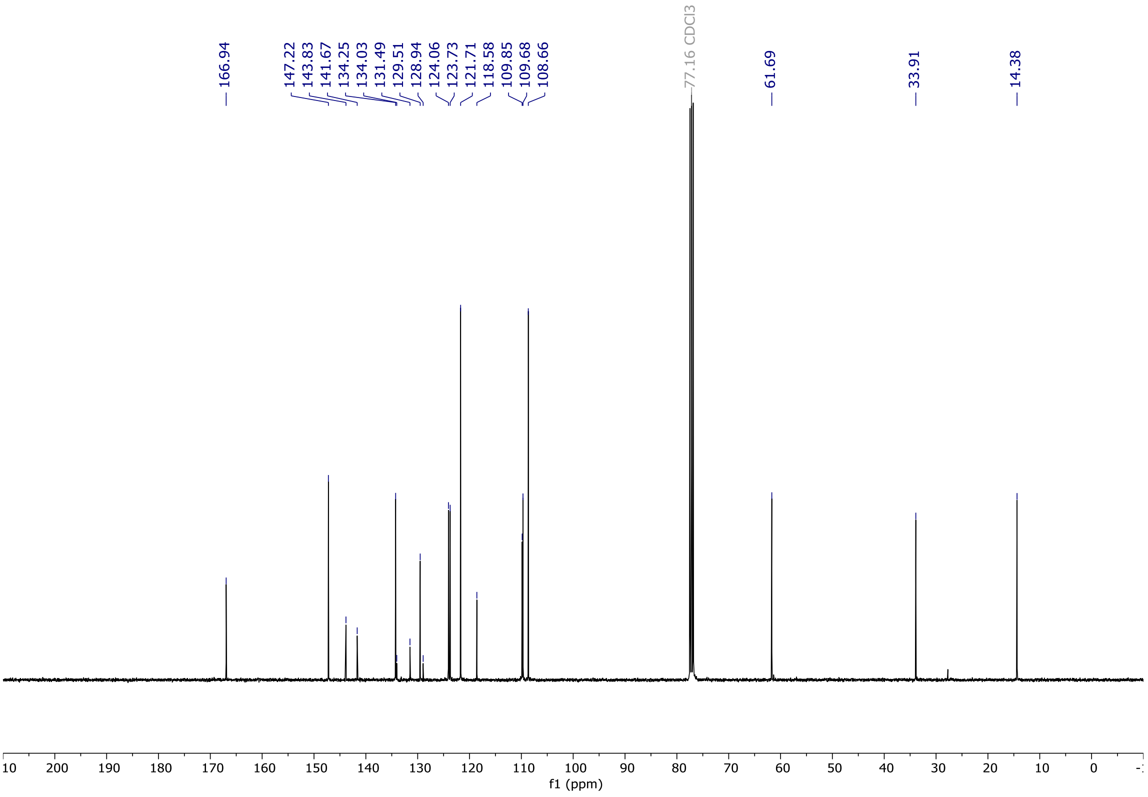

^{19}F NMR (376 MHz, $CDCl_3$) of compound **32** (major, E)

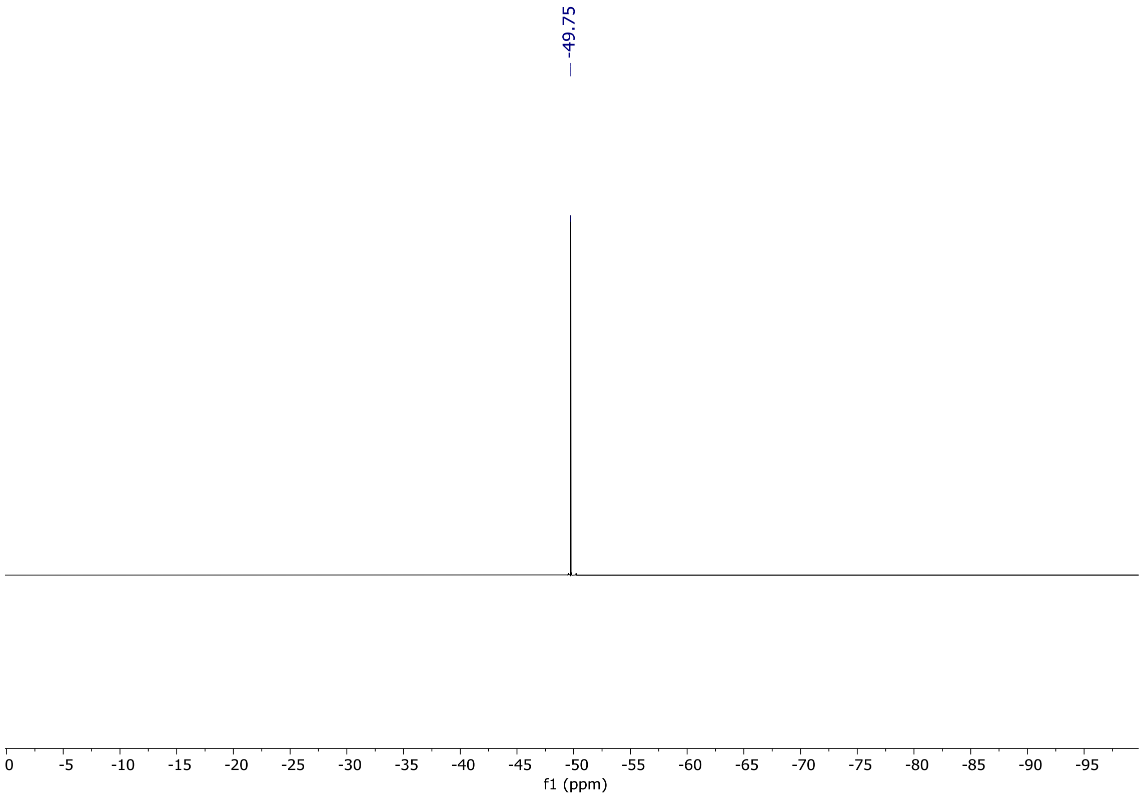

$^{1}H–^{1}H$ NOESY (400 MHz, $CDCl_3$) of compound **32** (major, E)

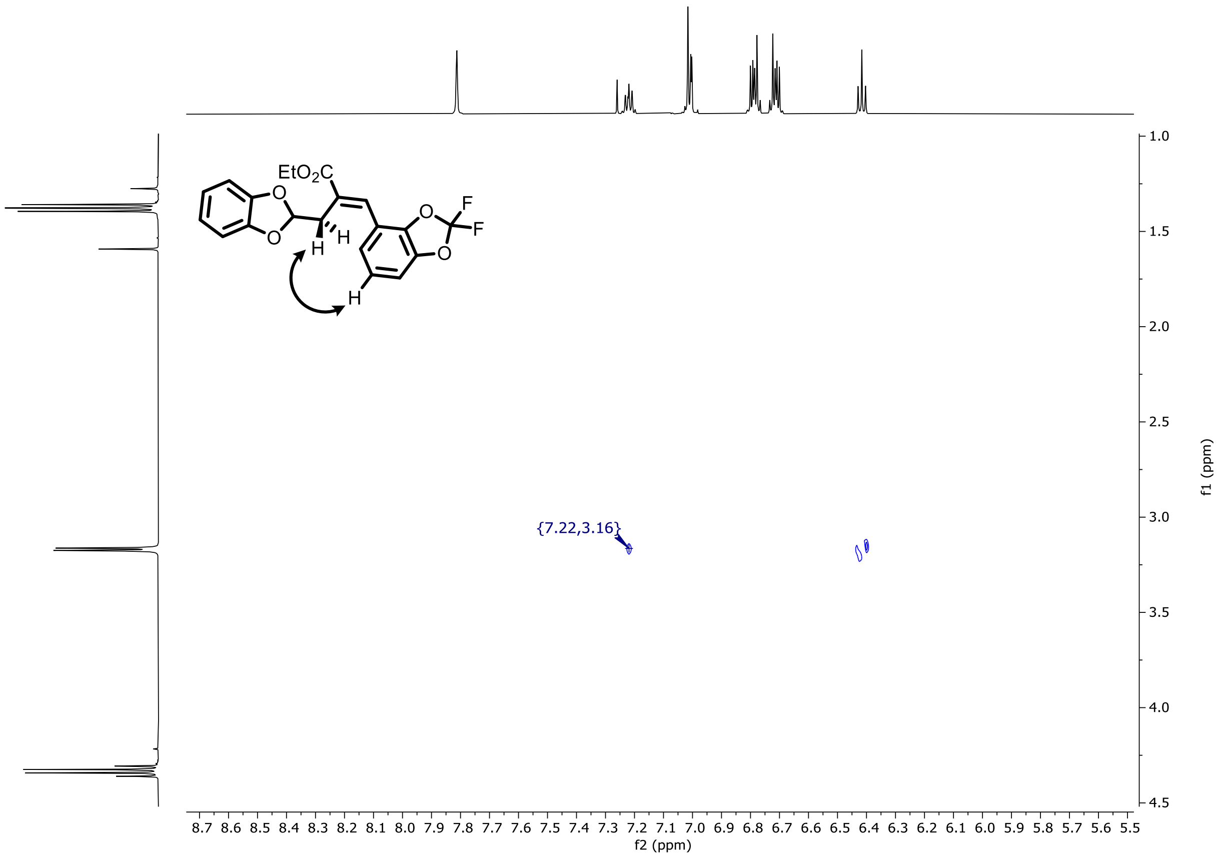

^{1}H NMR (400 MHz, $CDCl_3$) of compound **32** (minor, Z)

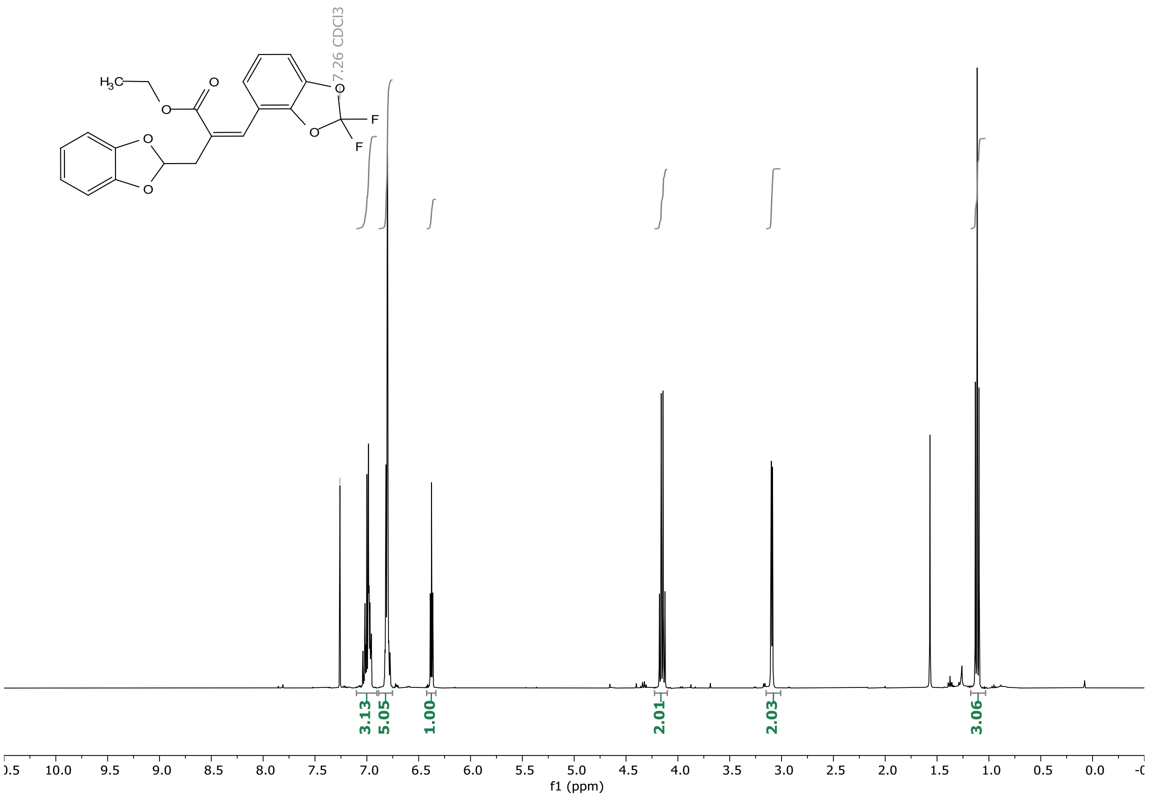

^{13}C NMR (101 MHz, $CDCl_3$) of compound **32** (minor, Z)

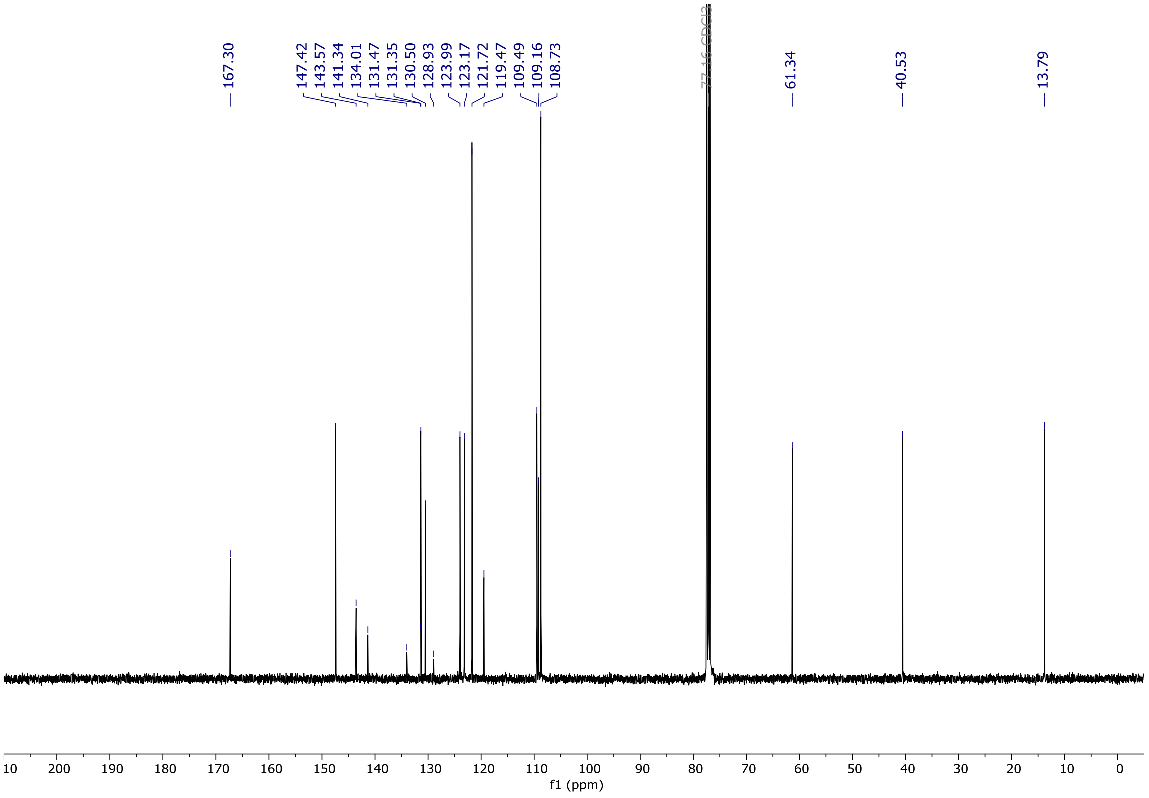

^{19}F NMR (376 MHz, $CDCl_3$) of compound **32** (minor, Z)

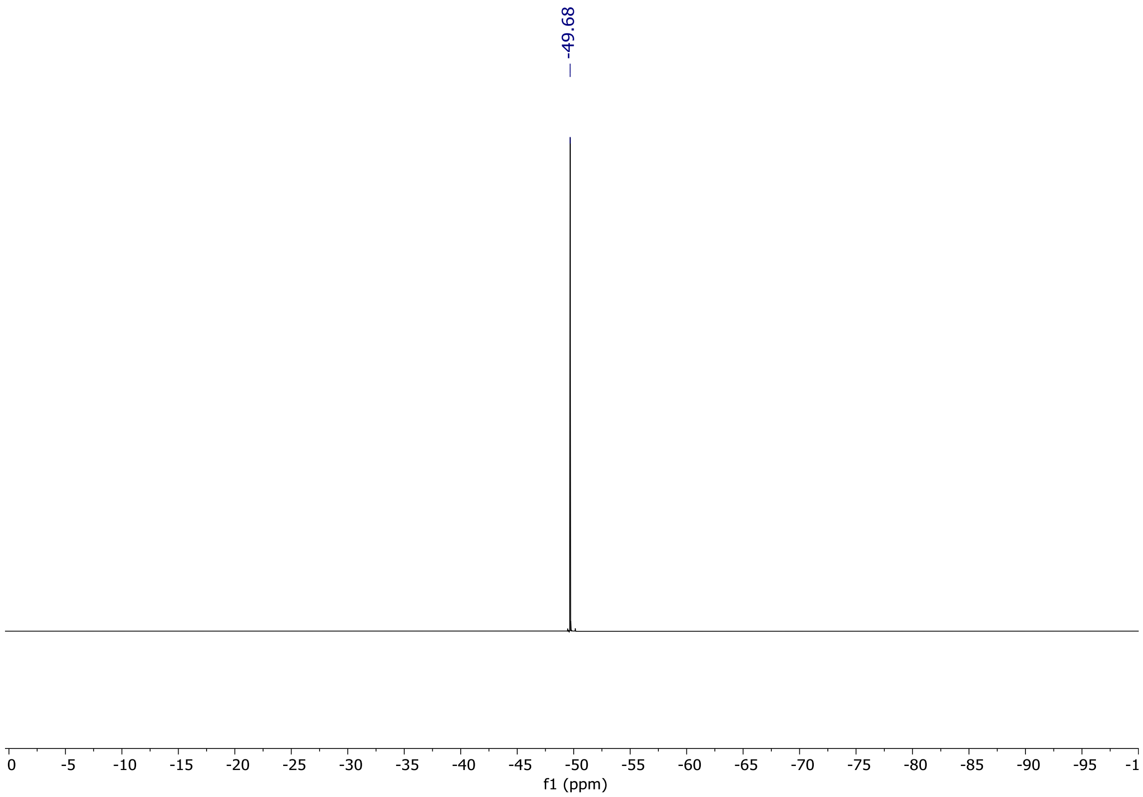

Key HSQC correlations of compound **32** (minor, Z)

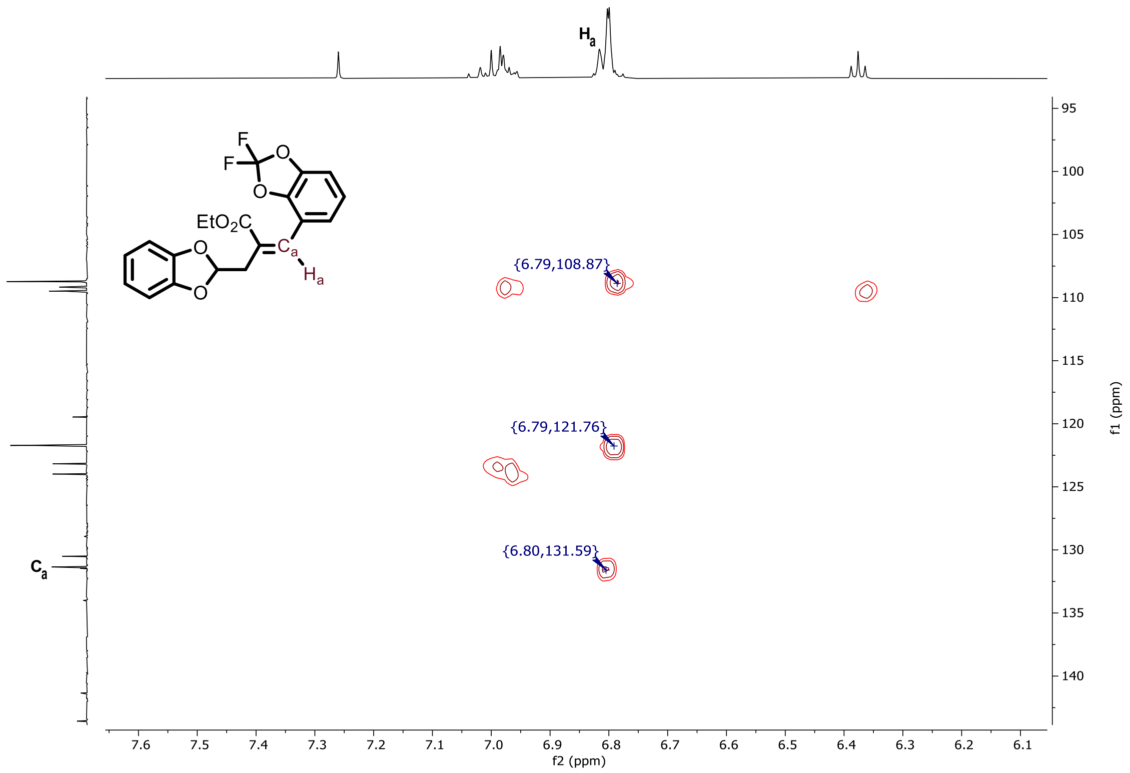

^{1}H–^{1}H NOESY (400 MHz, $CDCl_3$) of compound **32** (minor, Z)

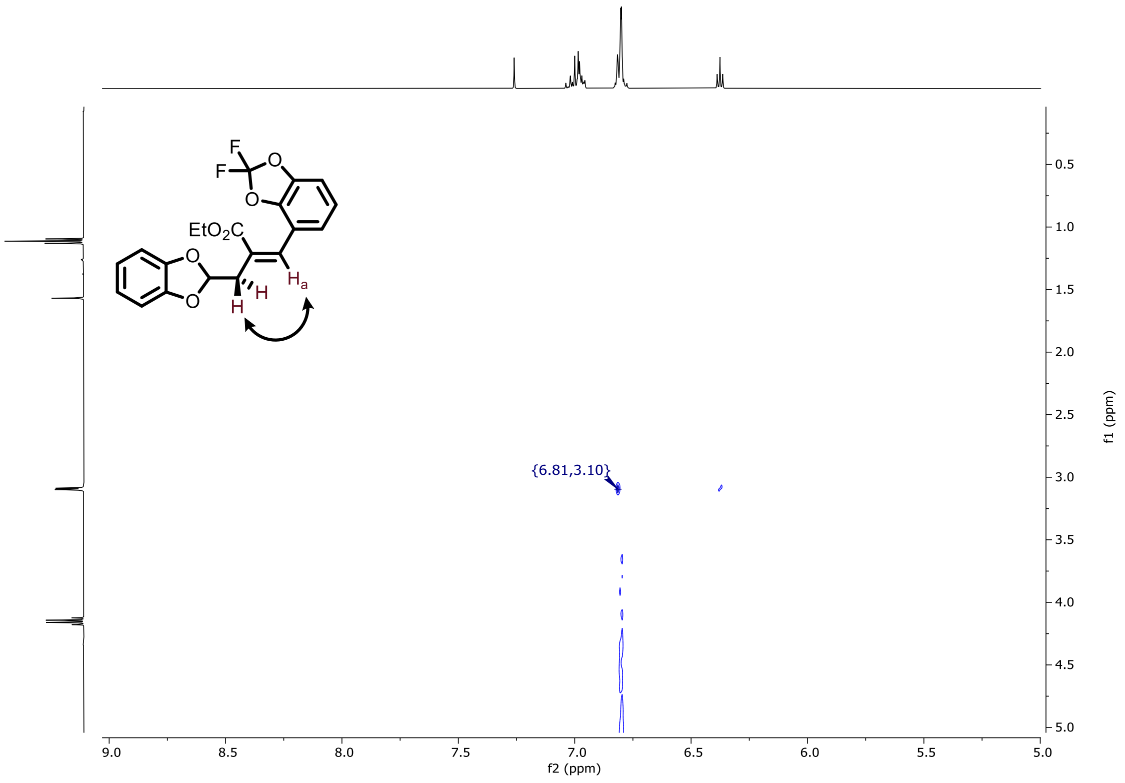

^1H NMR (400 MHz, $CDCl_3$) of compound **33** (major, E)

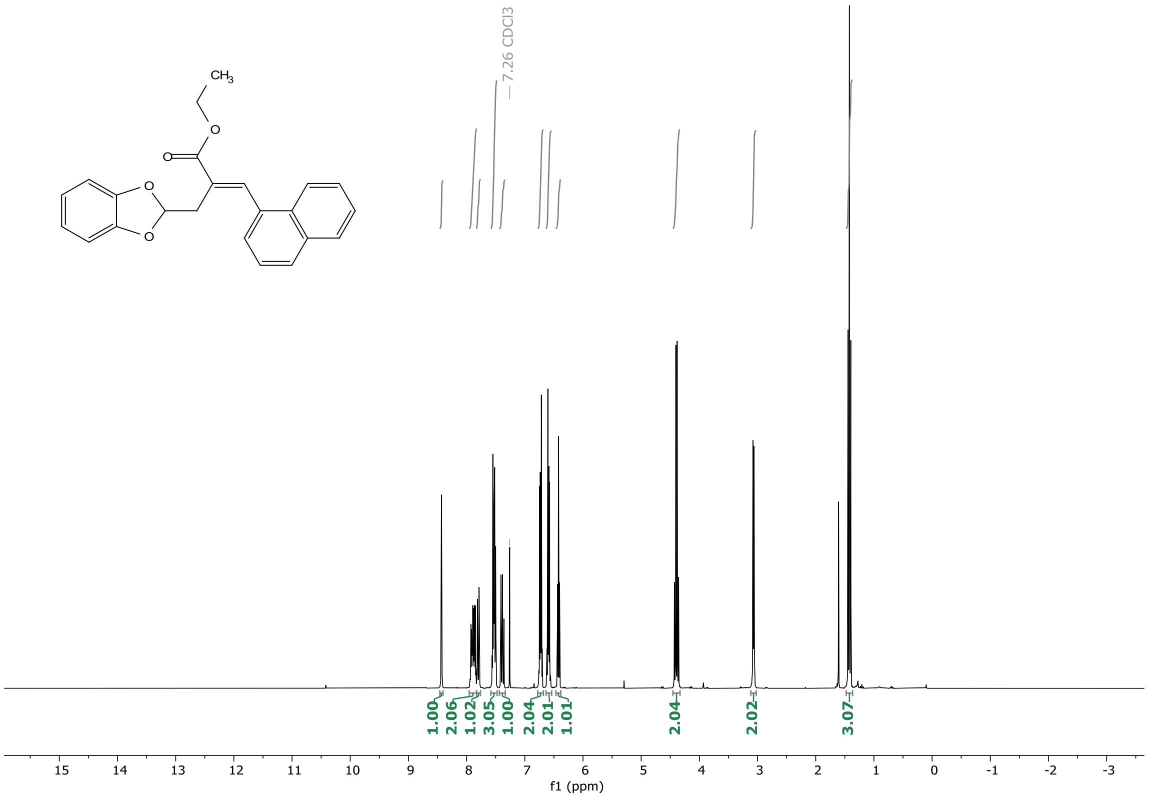

^{13}C NMR (101 MHz, $CDCl_3$) of compound **33** (major, E)

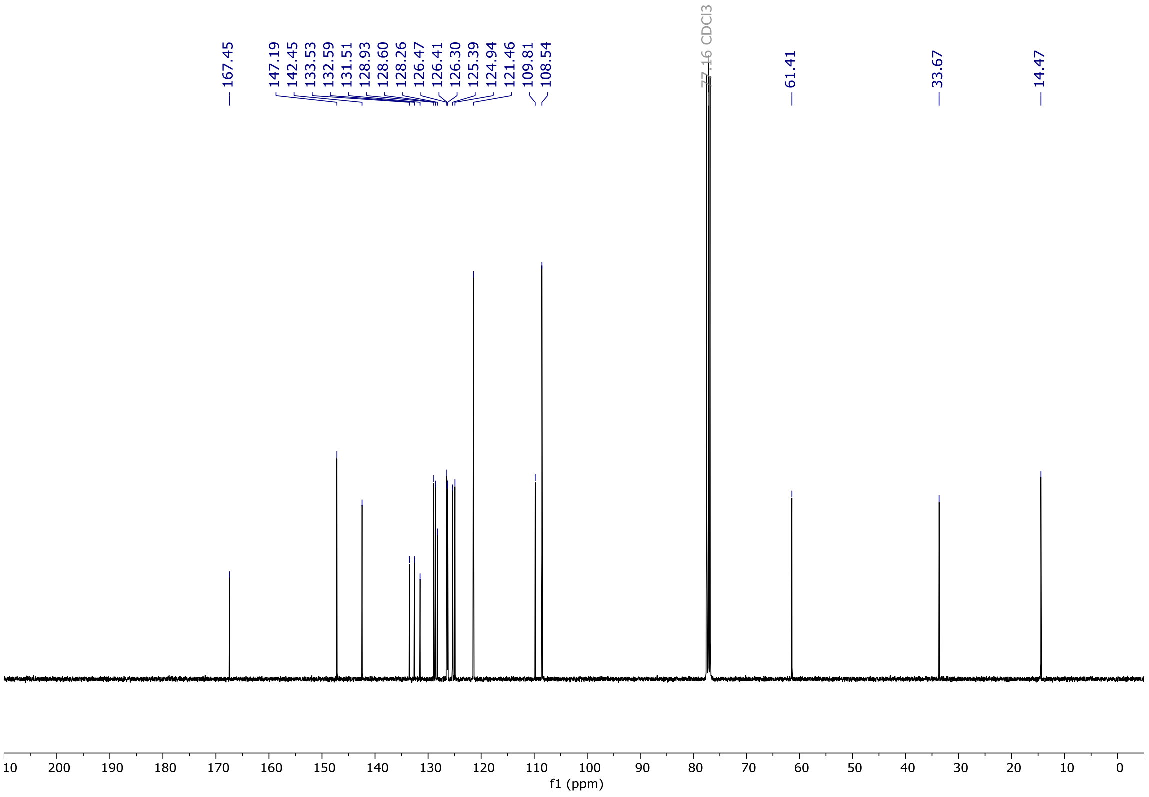

^{1}H–^{1}H NOESY (400 MHz, $CDCl_3$) of compound **33** (E isomer)

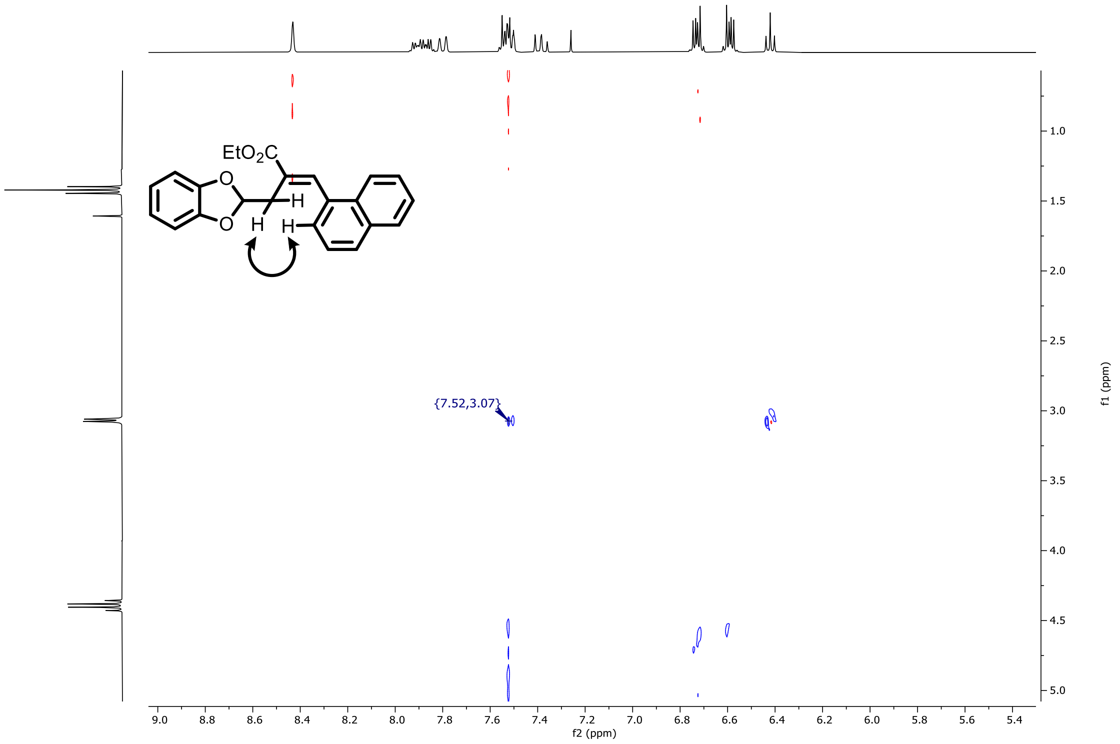

^{1}H NMR (400 MHz, $CDCl_3$) of compound **34** (major, E)

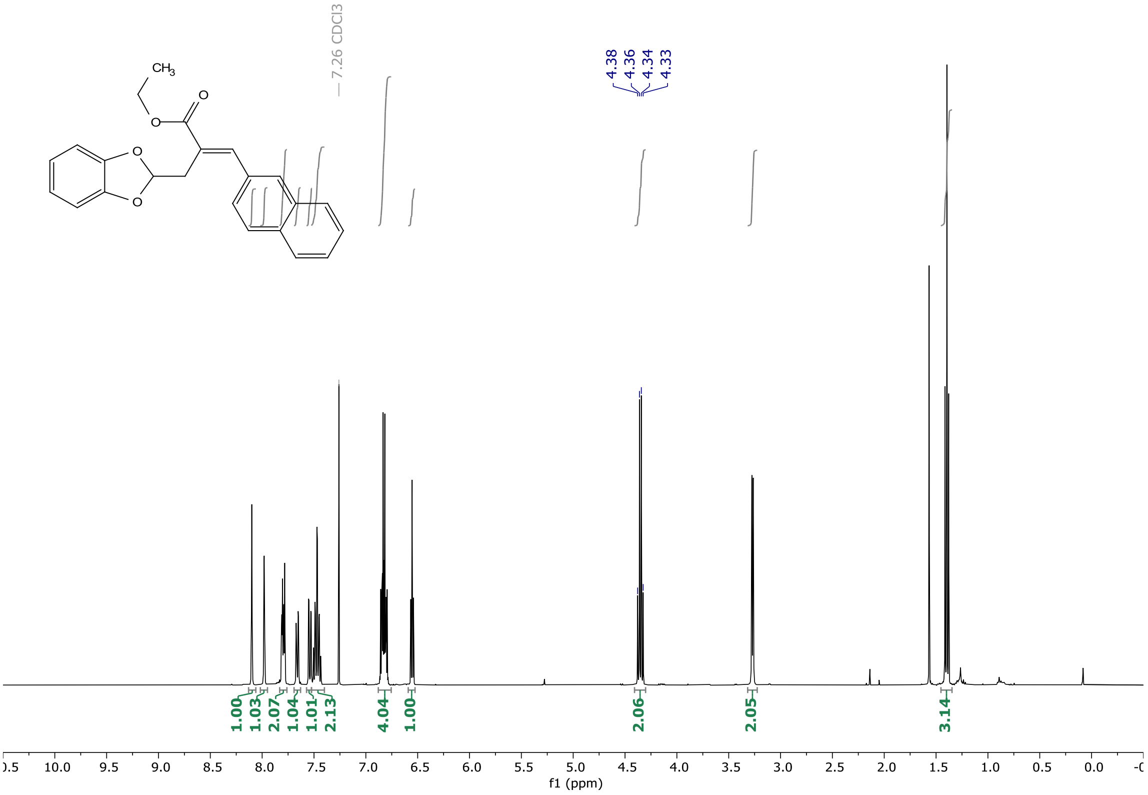

^{13}C NMR (101 MHz, $CDCl_3$) of compound **34** (major, E)

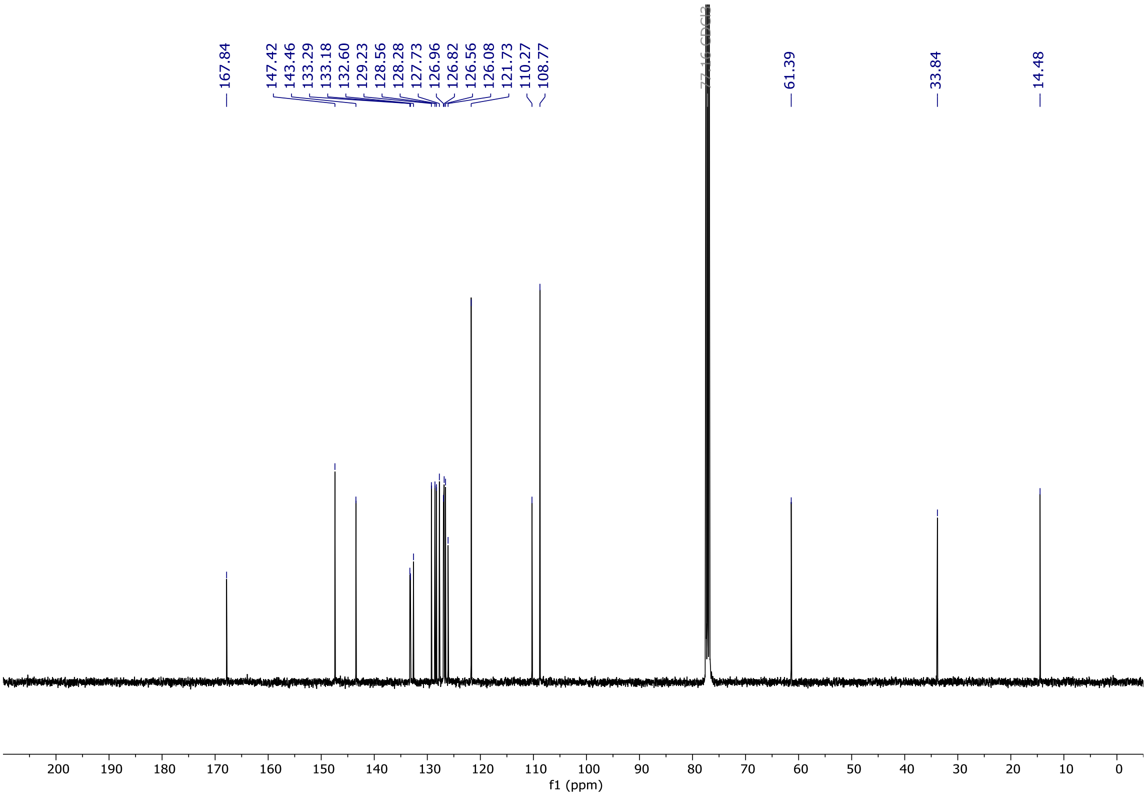

Key COSY (400 MHz, $CDCl_3$) correlations of compound **34** (major, E)

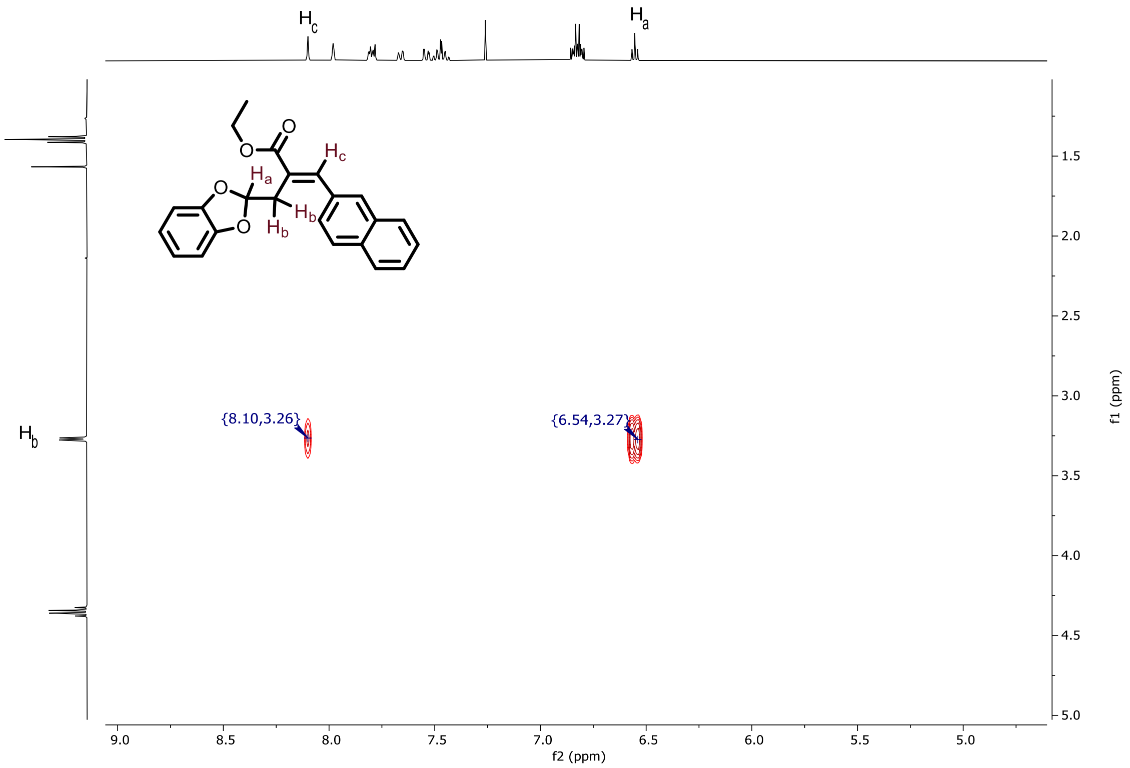

^1H–^1H NOESY (400 MHz, $CDCl_3$) of compound **34** (major, E)

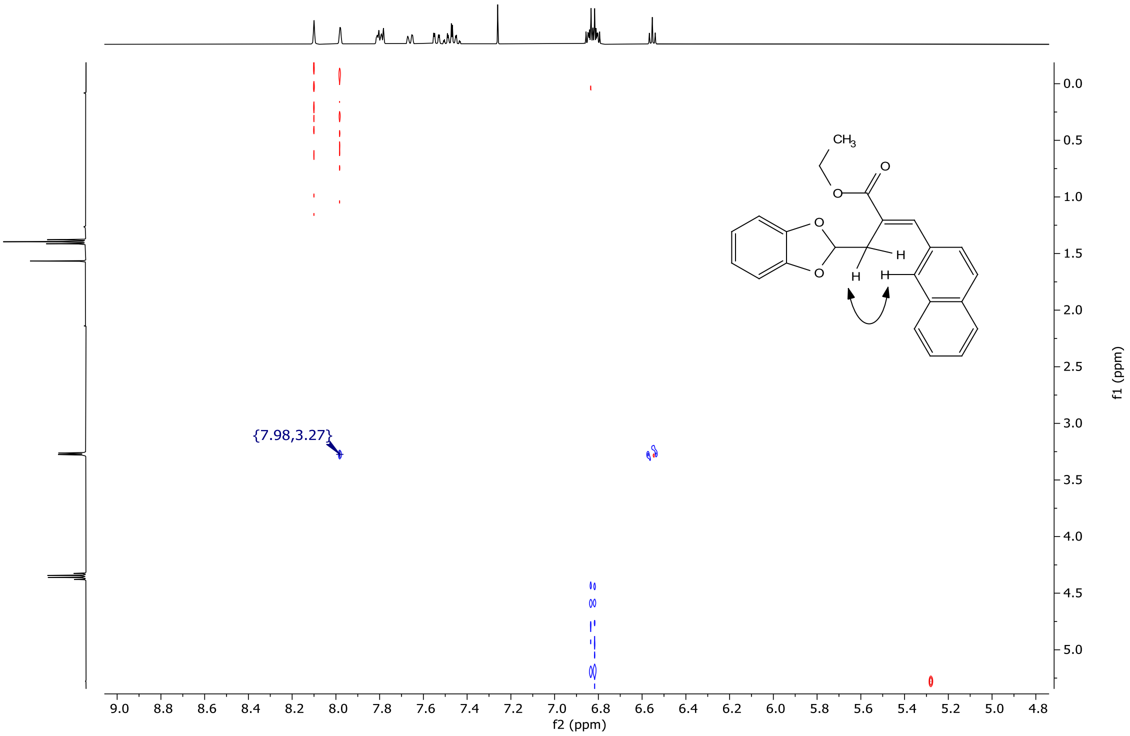

^{1}H NMR (400 MHz, $CDCl_3$) of compound **34** (minor, Z)

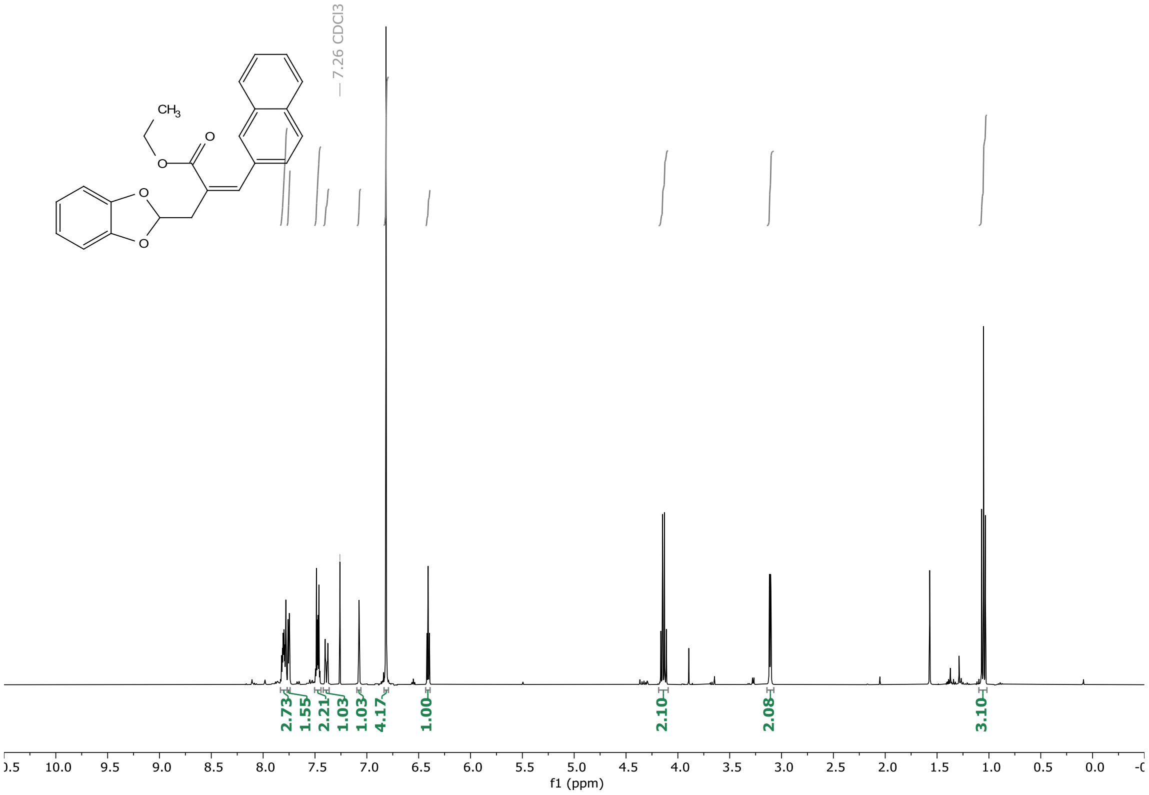

^{13}C NMR (101 MHz, $CDCl_3$) of compound **34** (minor, Z)

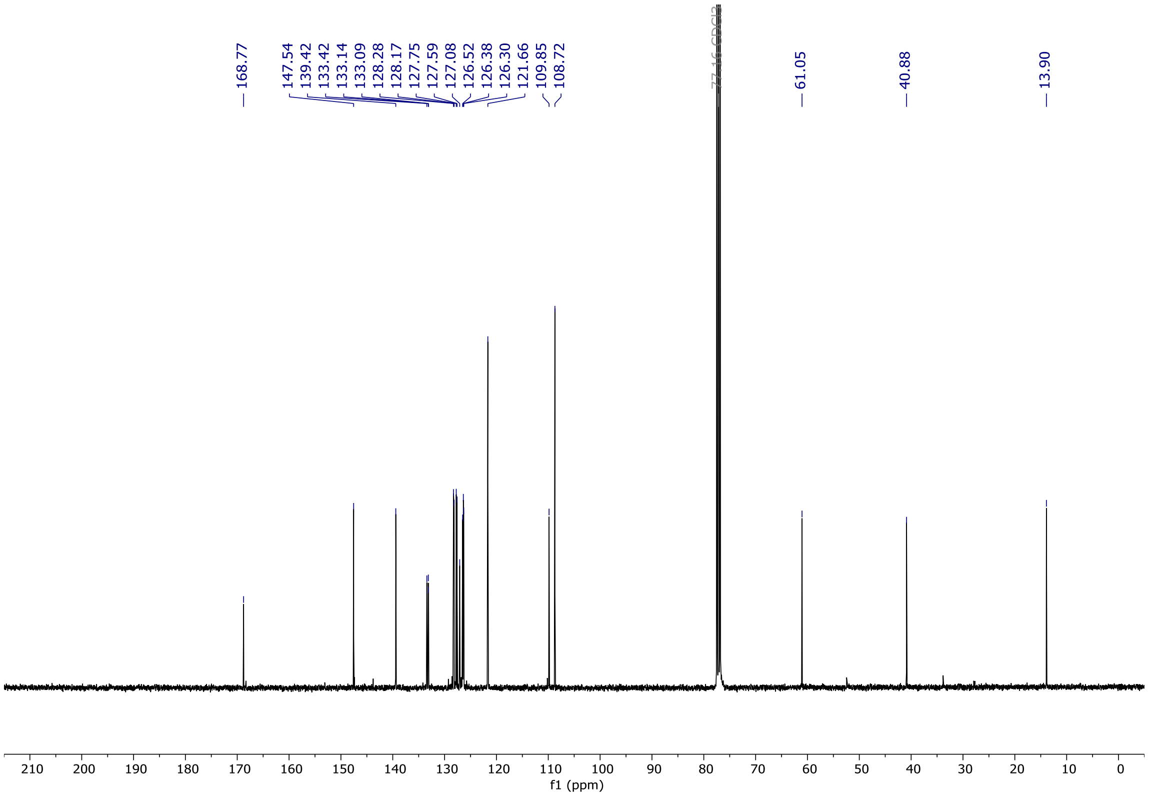

Key HSQC correlations of compound **34** (minor, Z)

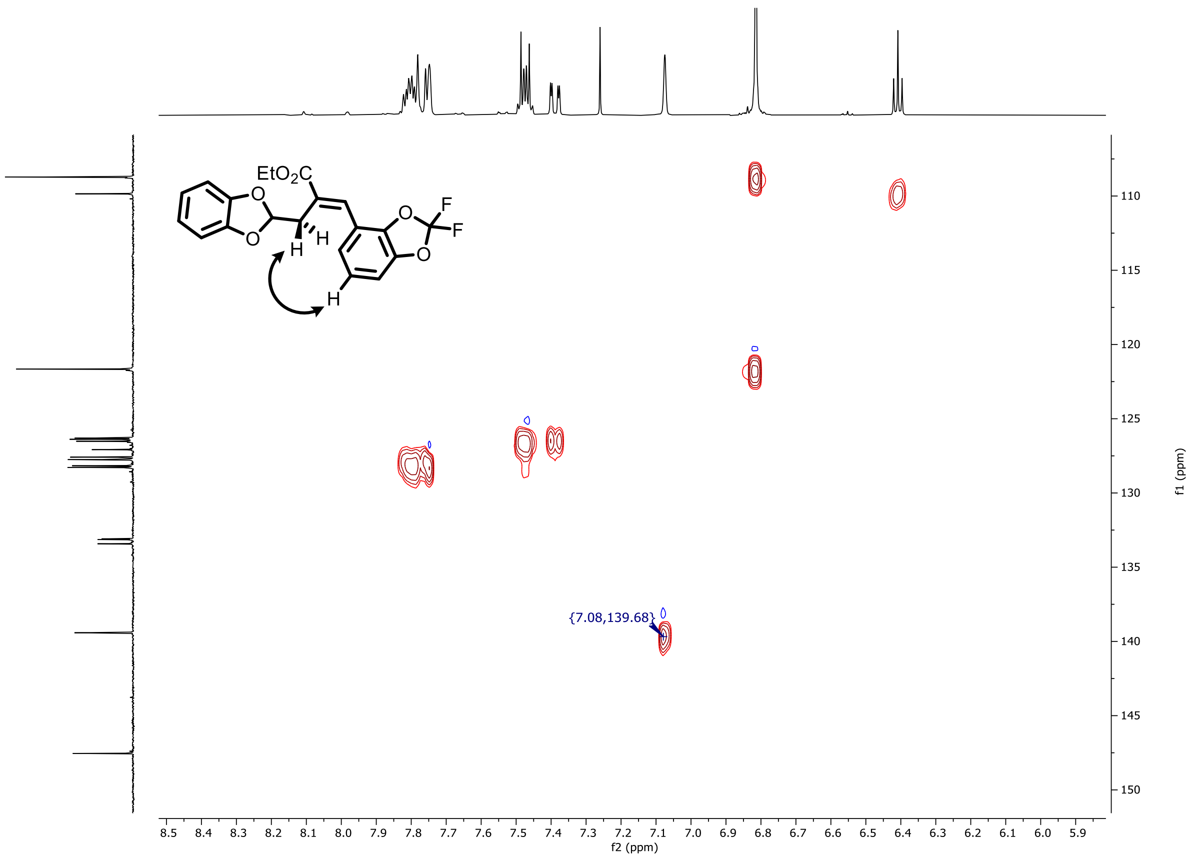

^{1}H–^{1}H NOESY (400 MHz, $CDCl_3$) of compound **34** (minor, Z)

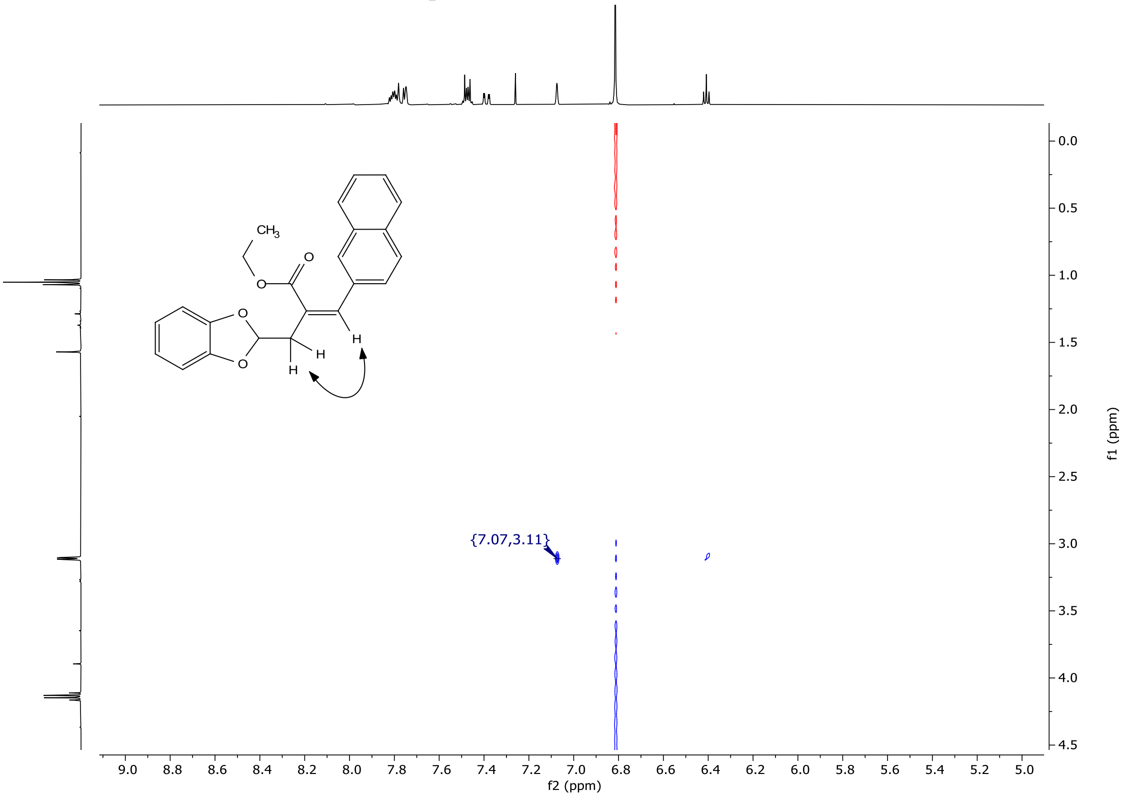

^{1}H NMR (400 MHz, $CDCl_3$) of compound **35** (major, E)

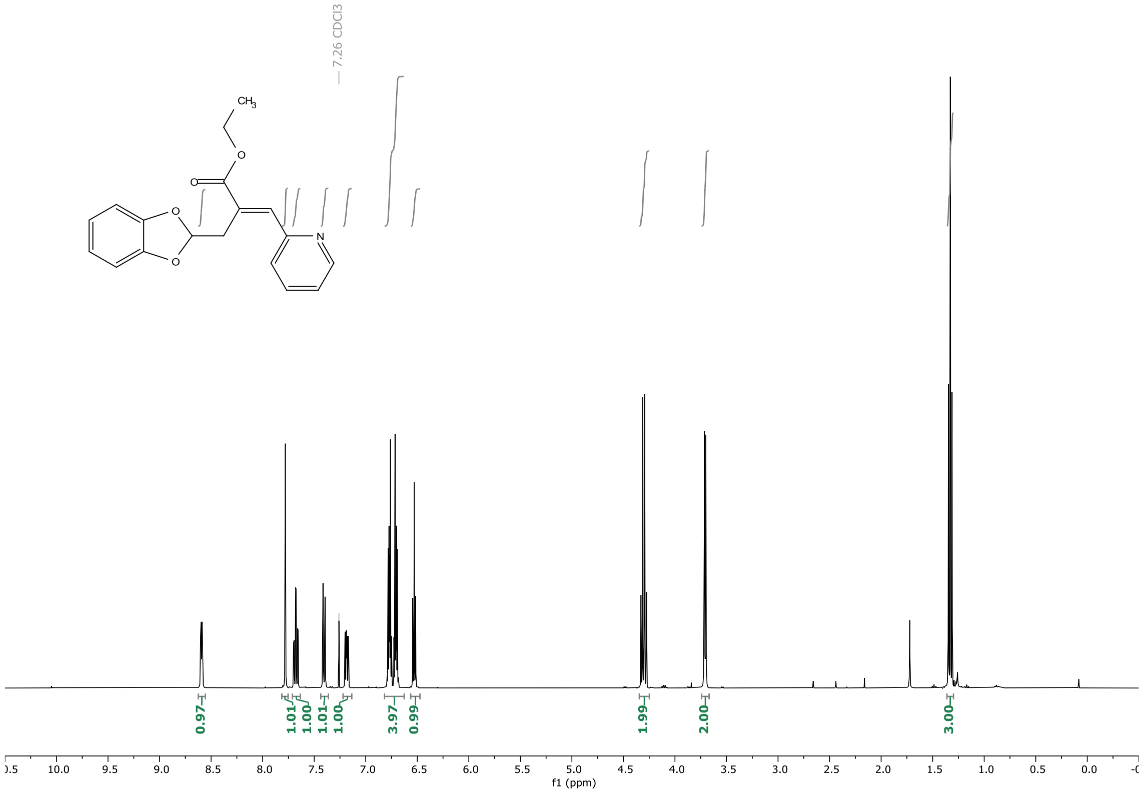

^{13}C NMR (101 MHz, $CDCl_3$) of compound **35** (major, E)

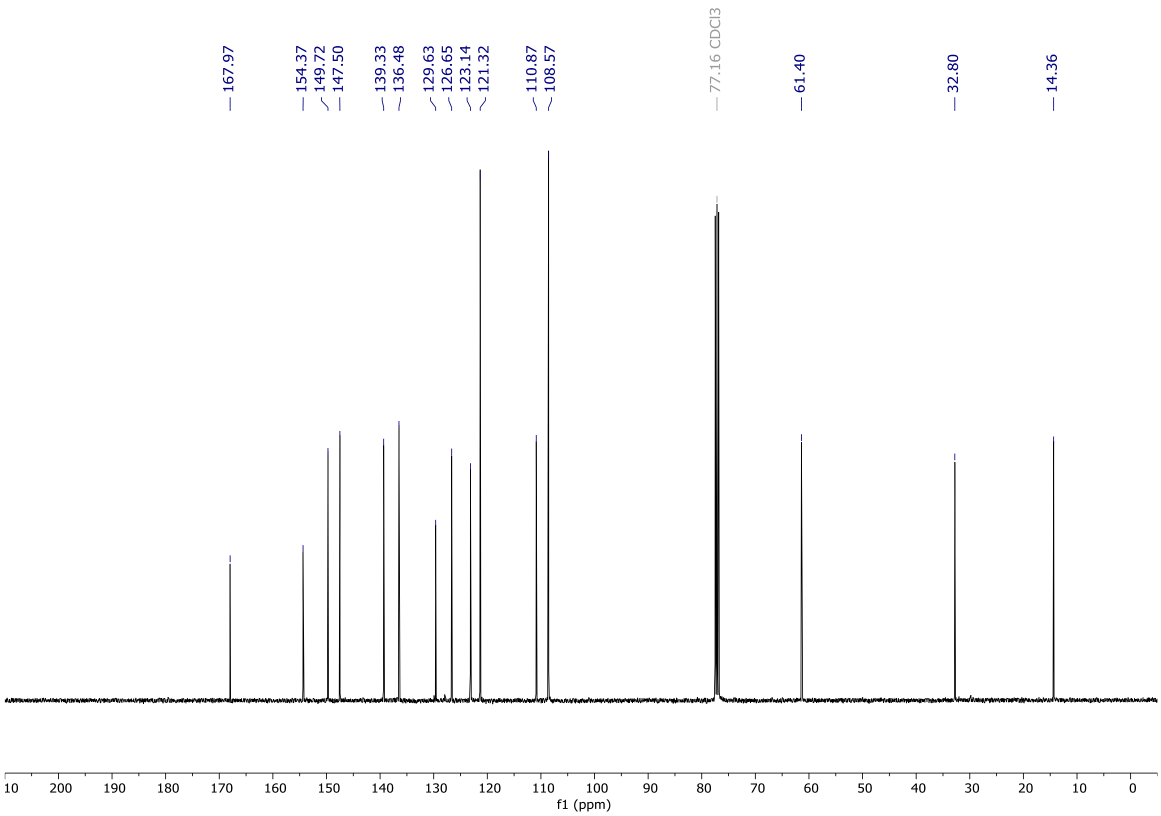

^{1}H NMR (400 MHz, $CDCl_3$) of compound **35** (minor, Z)

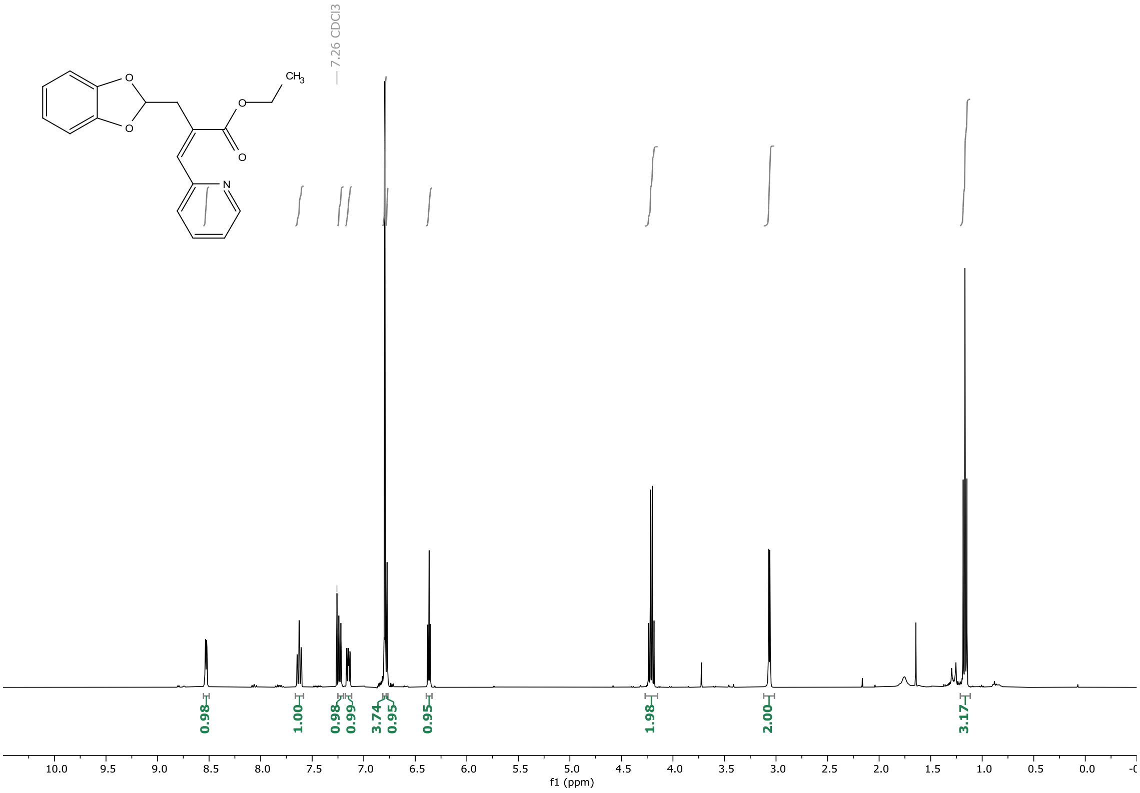

^{13}C NMR (101 MHz, $CDCl_3$) of compound **35** (minor, Z)

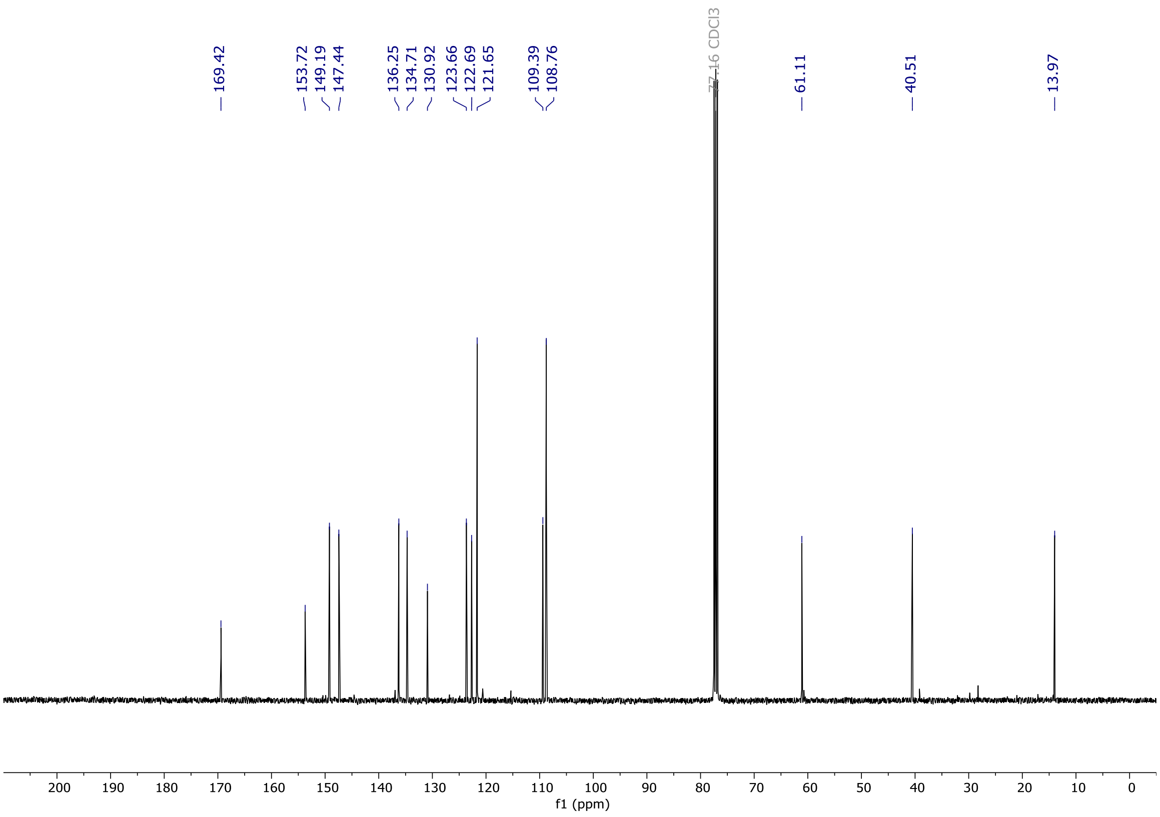

^{1}H NMR (400 MHz, $CDCl_3$) of compound **36** (isomer 1, E)

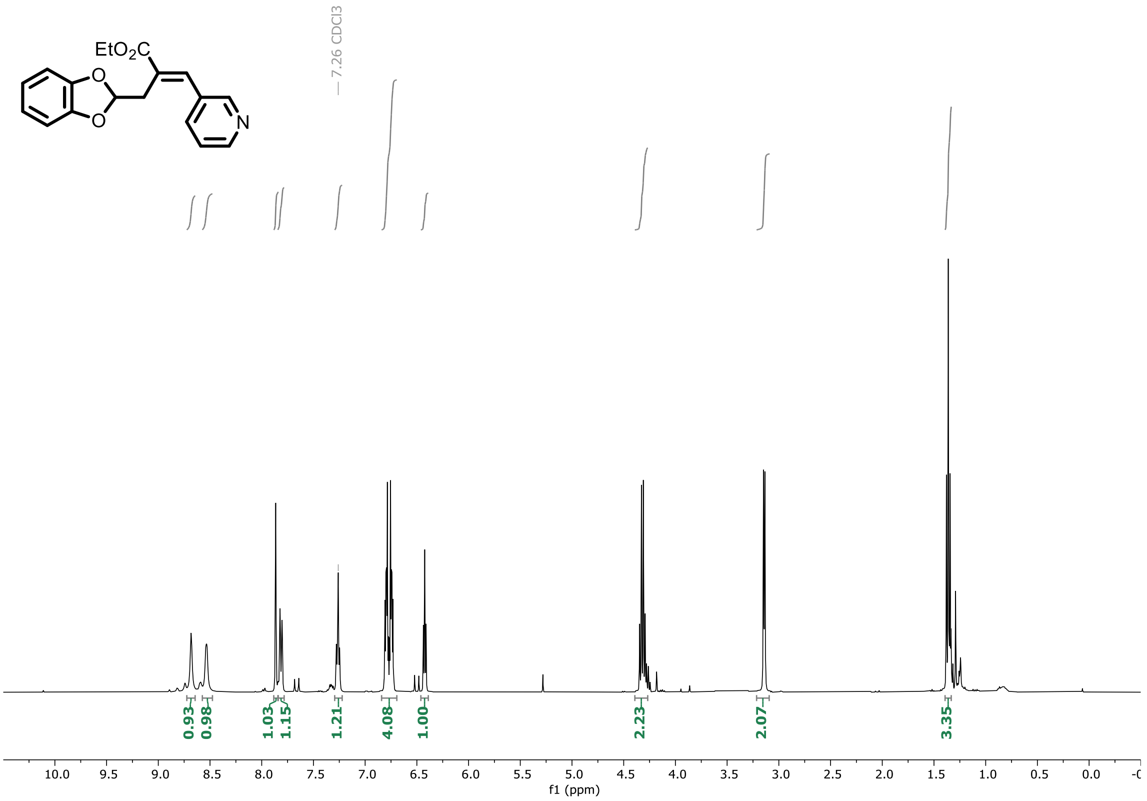

^{13}C NMR (101 MHz, $CDCl_3$) of compound **36** (isomer 1, E)

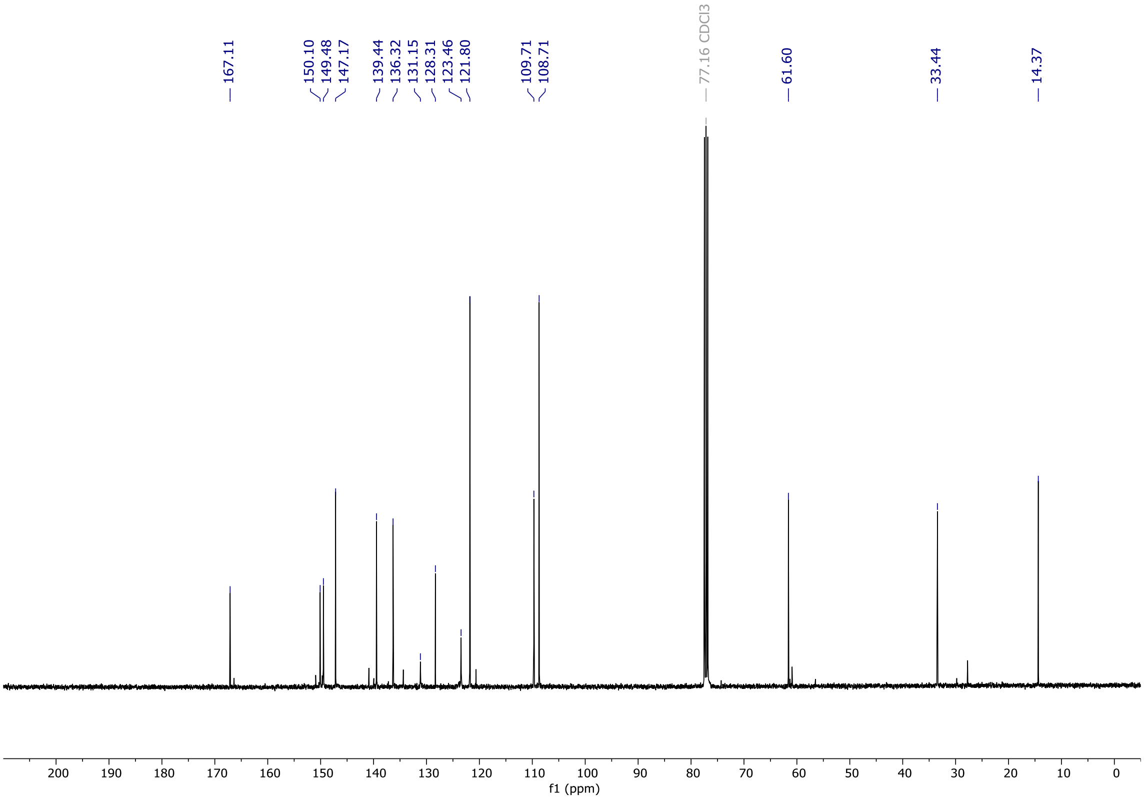

^{1}H–^{1}H NOESY (400 MHz, $CDCl_3$) of compound **36** (isomer 1, E)

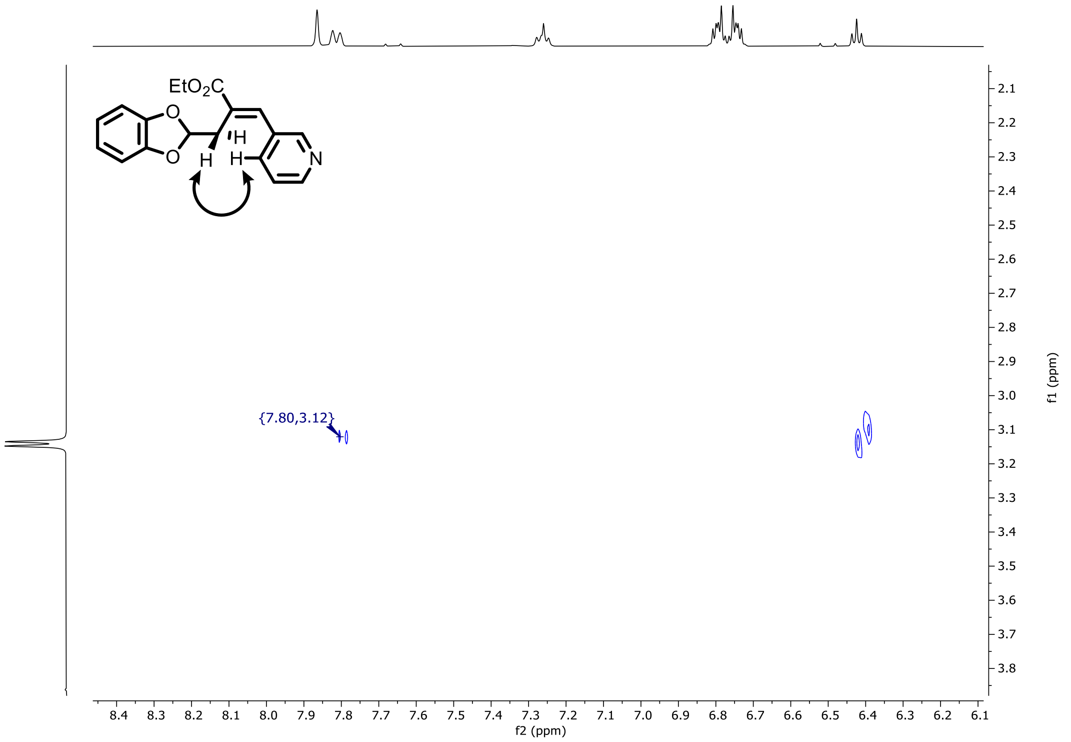

^{1}H NMR (400 MHz, $CDCl_3$) of compound **36** (isomer 2, Z)

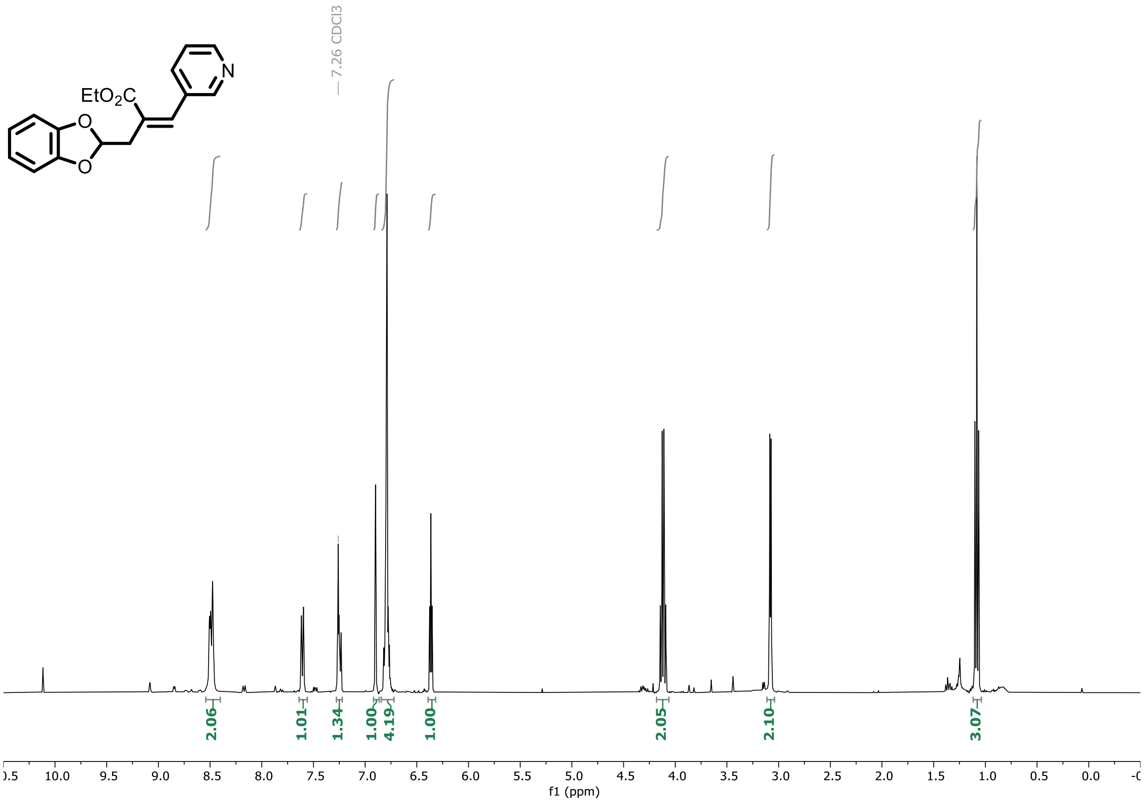

^{13}C NMR (101 MHz, $CDCl_3$) of compound **36** (isomer 2, Z)

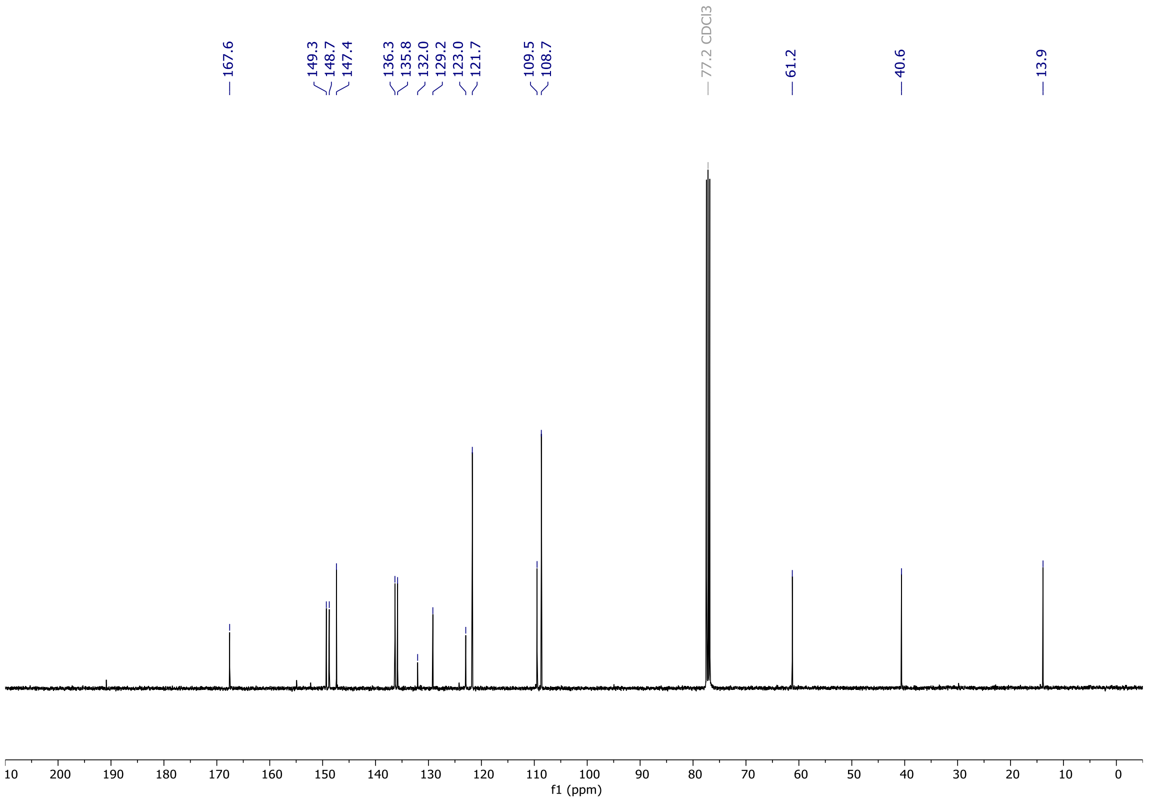

Key COSY (400 MHz, $CDCl_3$) correlations of compound **36** (isomer 2, Z)

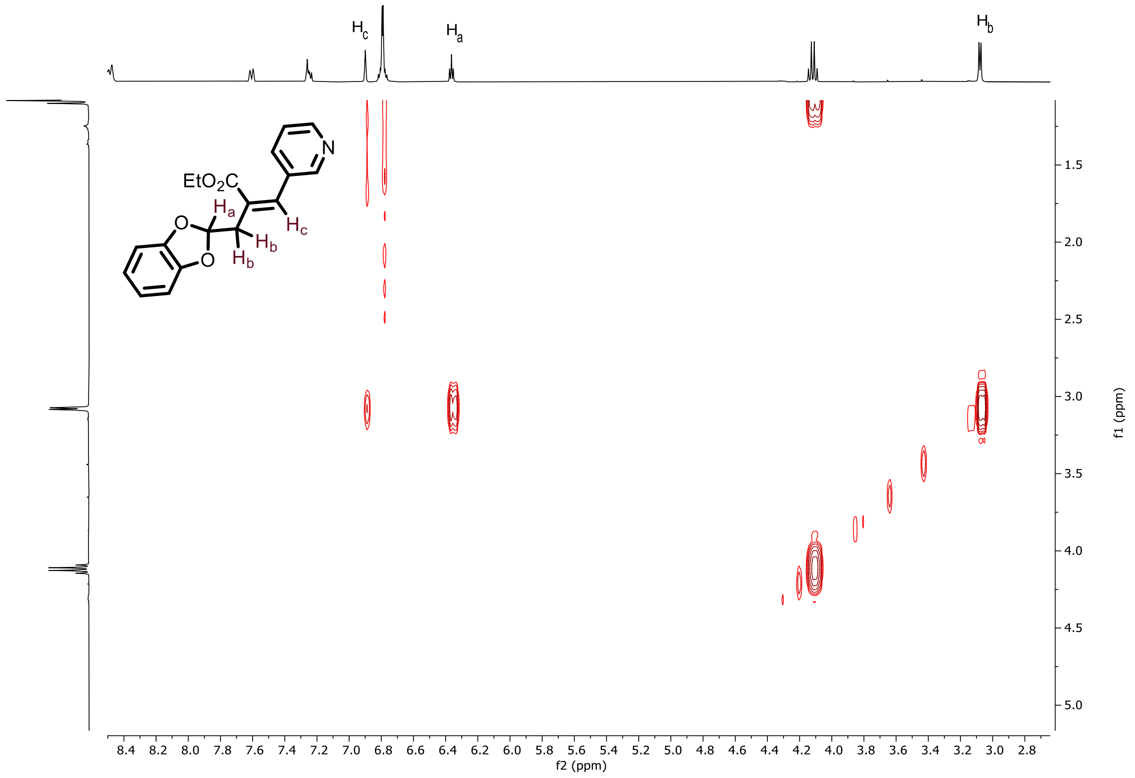

^{1}H–^{1}H NOESY (400 MHz, $CDCl_3$) of compound **36** (isomer 2, Z)

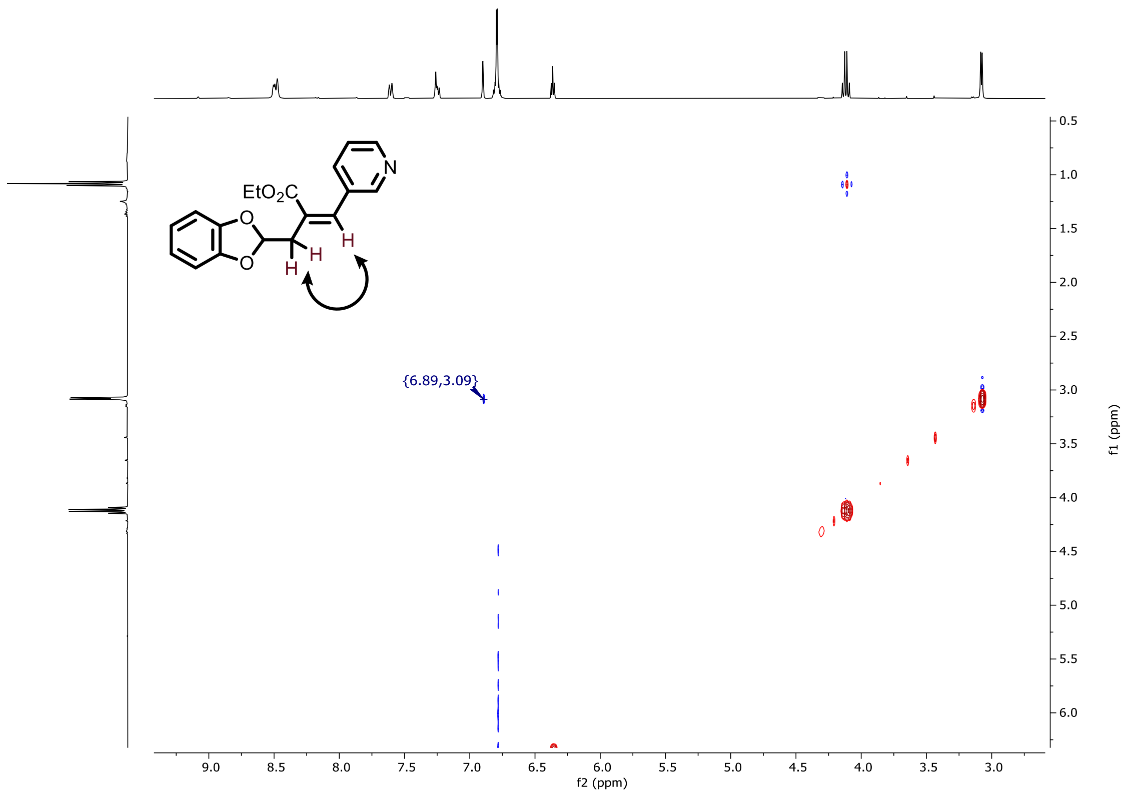

^{1}H NMR (400 MHz, $CDCl_3$) of compound **37** (major, E)

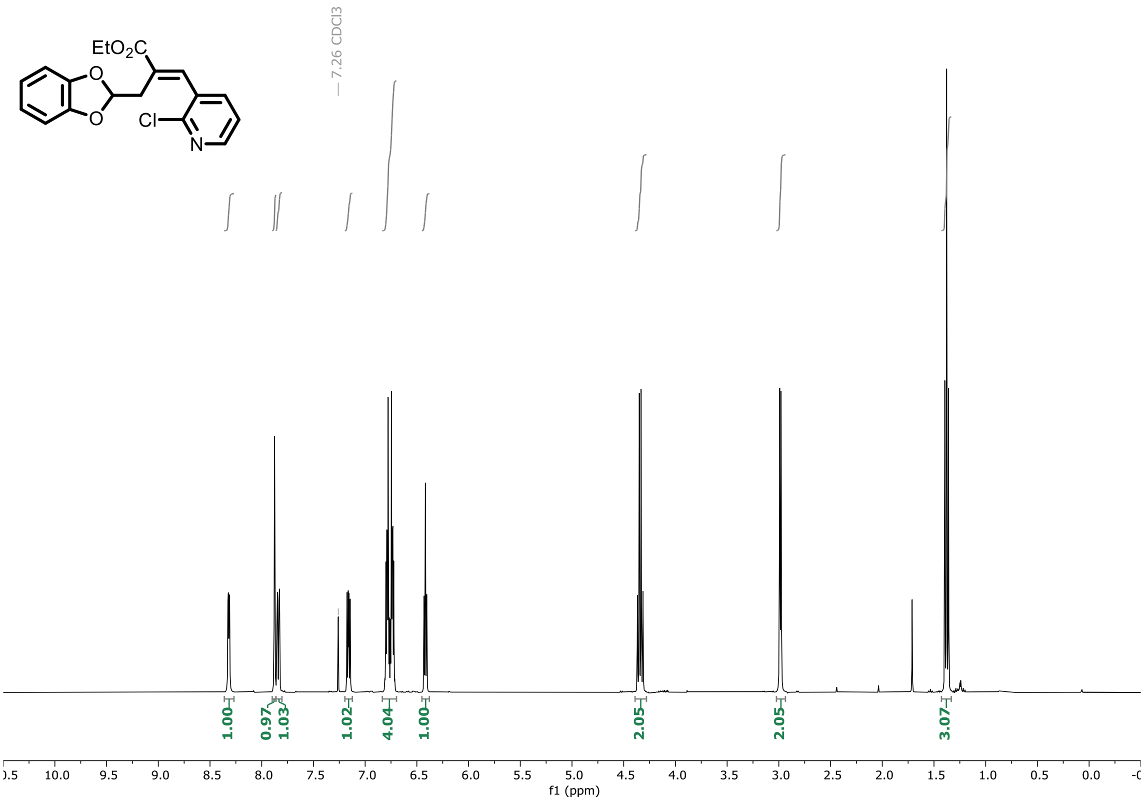

^{13}C NMR (101 MHz, $CDCl_3$) of compound **37** (major, E)

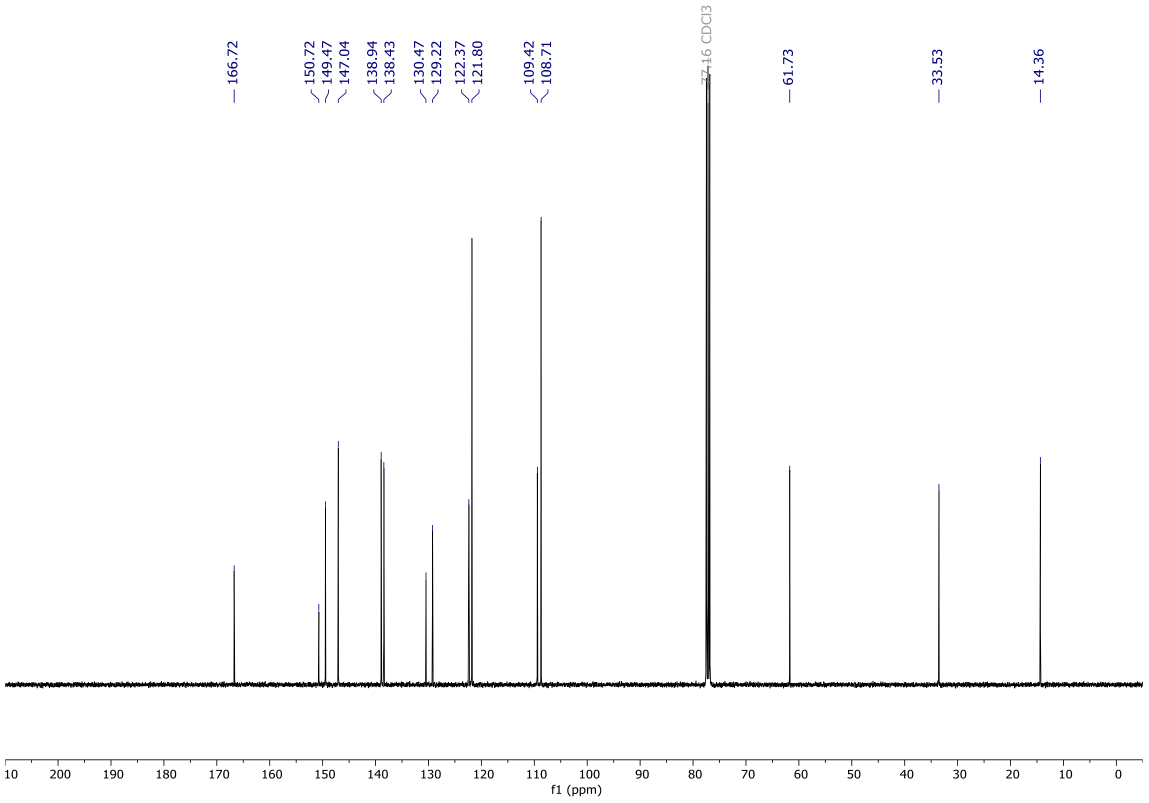

^{1}H–^{1}H NOESY (400 MHz, $CDCl_3$) of compound **37** (major, E)

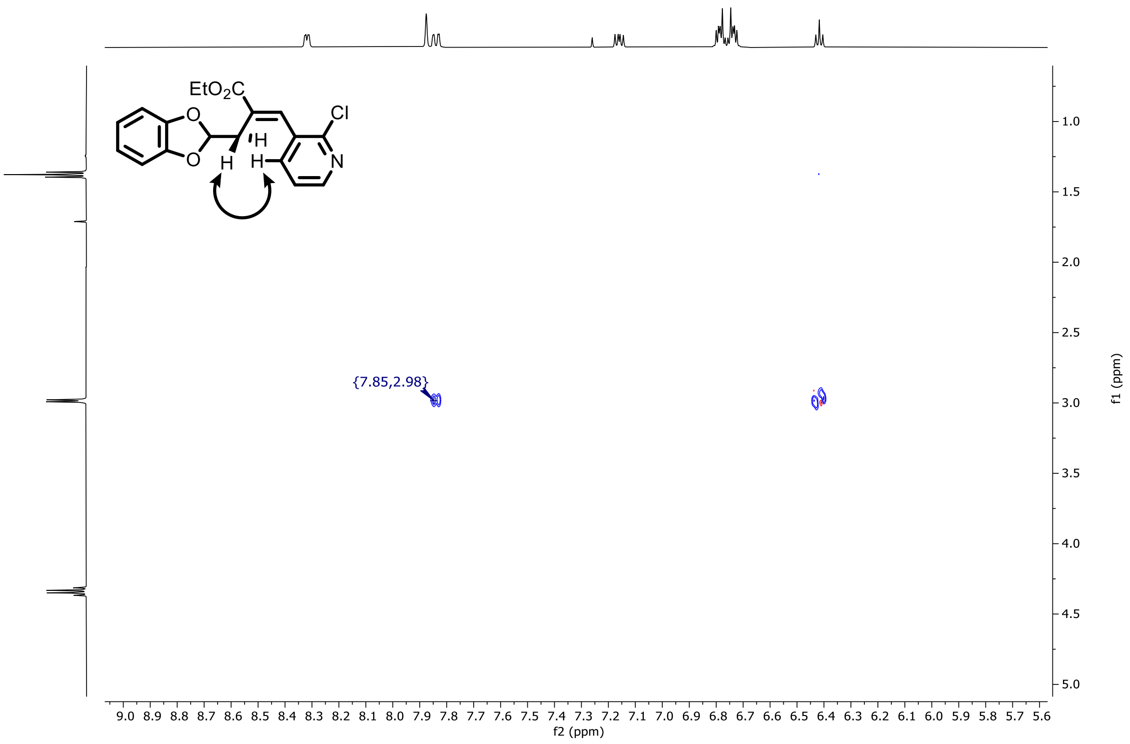

^1H NMR (400 MHz, $CDCl_3$) of compound **37** (minor, Z)

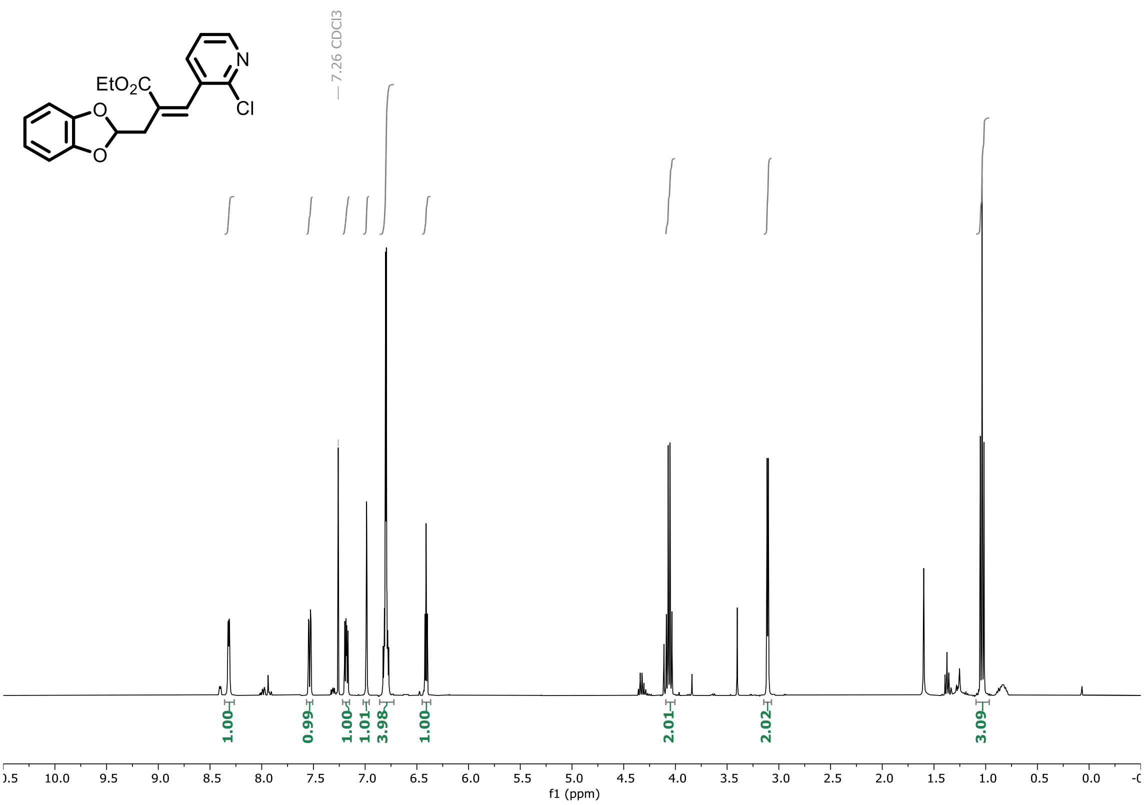

^{13}C NMR (101 MHz, $CDCl_3$) of compound **37** (minor, Z)

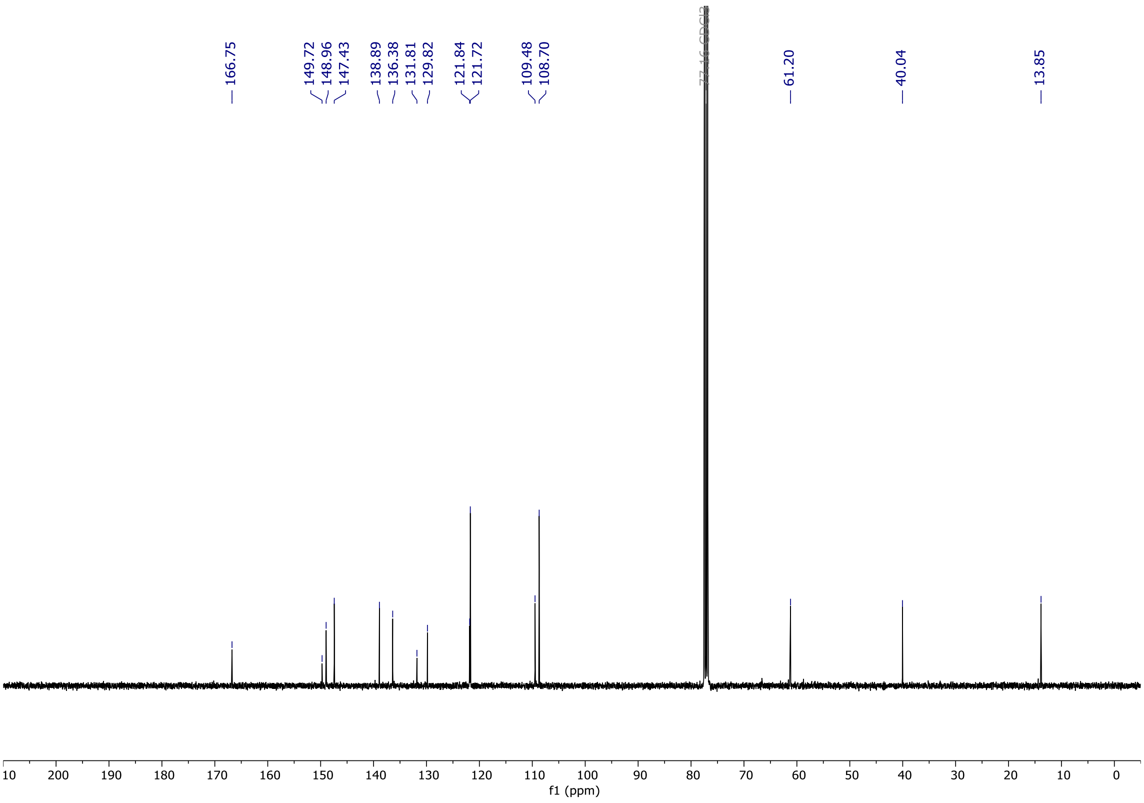

Key COSY (400 MHz, $CDCl_3$) correlations of compound **37** (minor, Z)

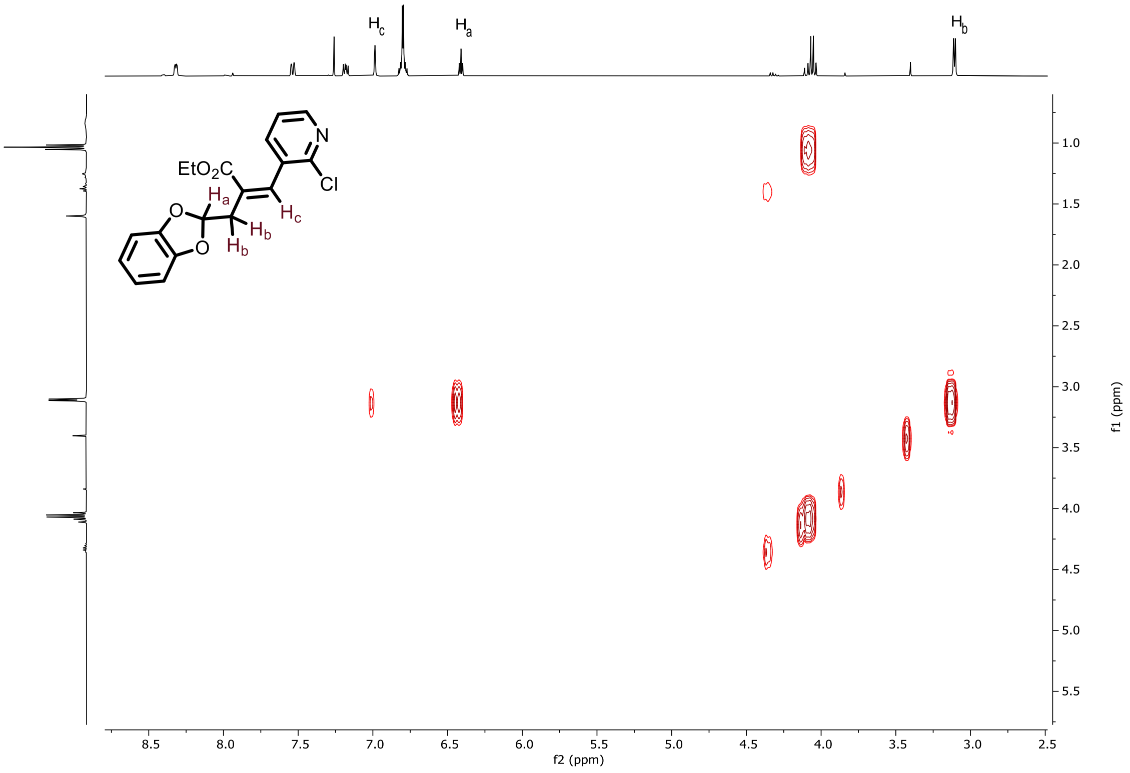

^{1}H–^{1}H NOESY (400 MHz, $CDCl_3$) of compound **37** (minor, Z)

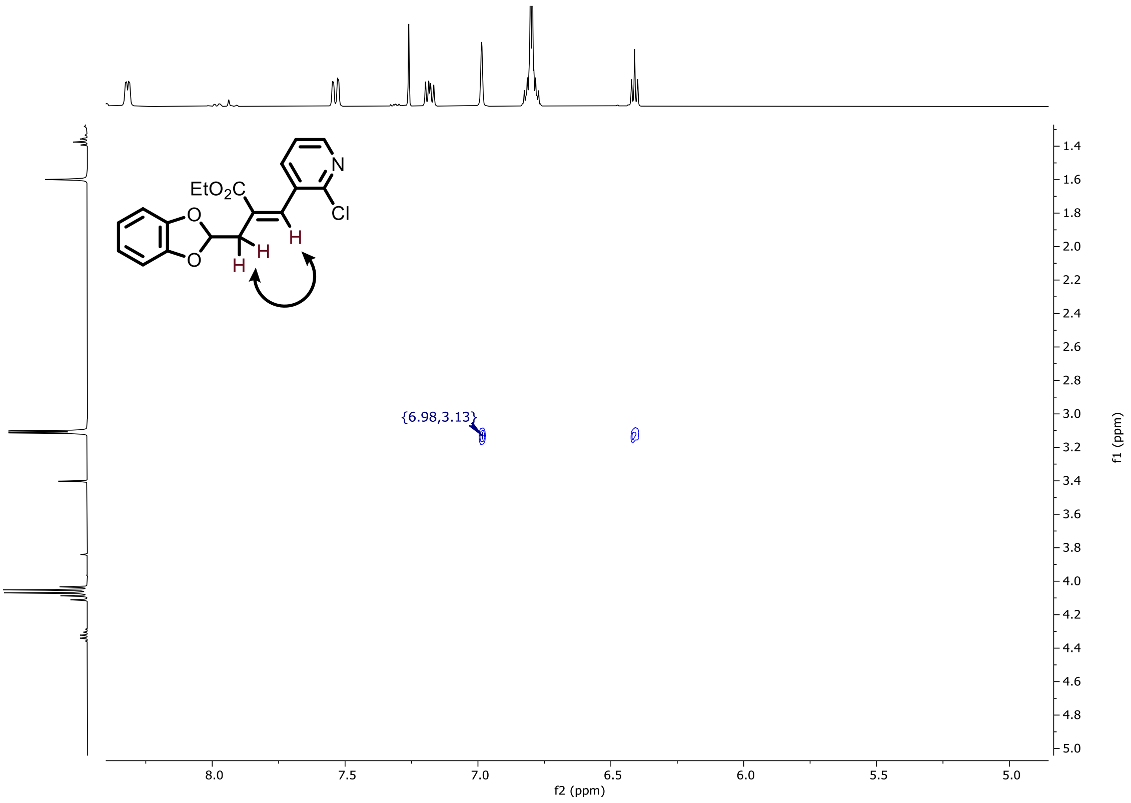

^{1}H NMR (400 MHz, $CDCl_3$) of compound **38** (isomer 1, E)

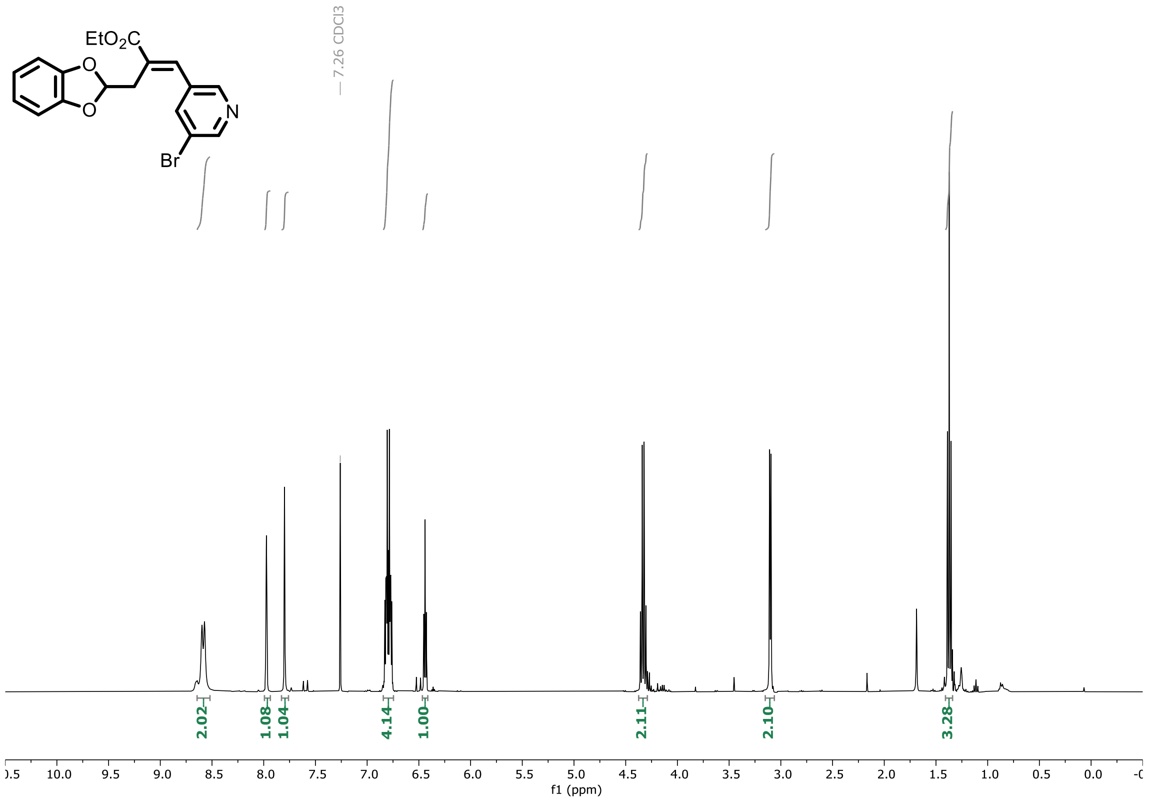

^{13}C NMR (101 MHz, $CDCl_3$) of compound **38** (isomer 1, E)

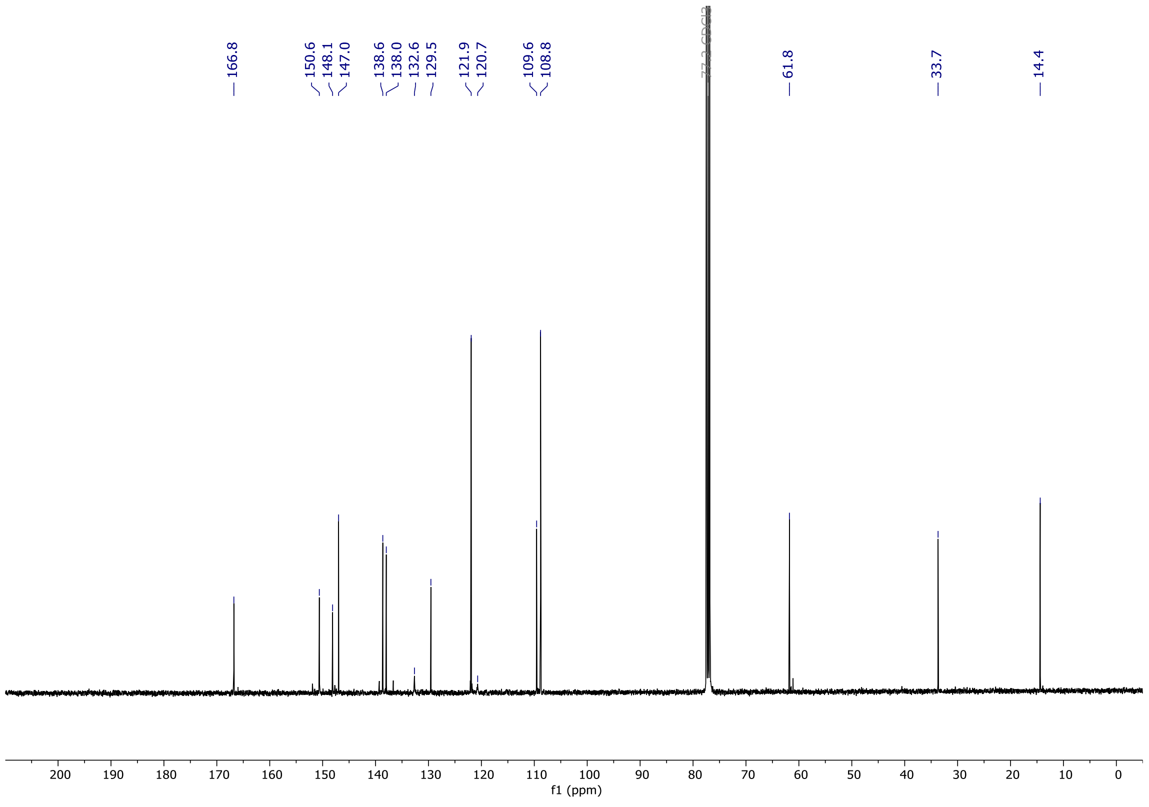

Key COSY (400 MHz, $CDCl_3$) correlations of compound **38** (isomer 1, E)

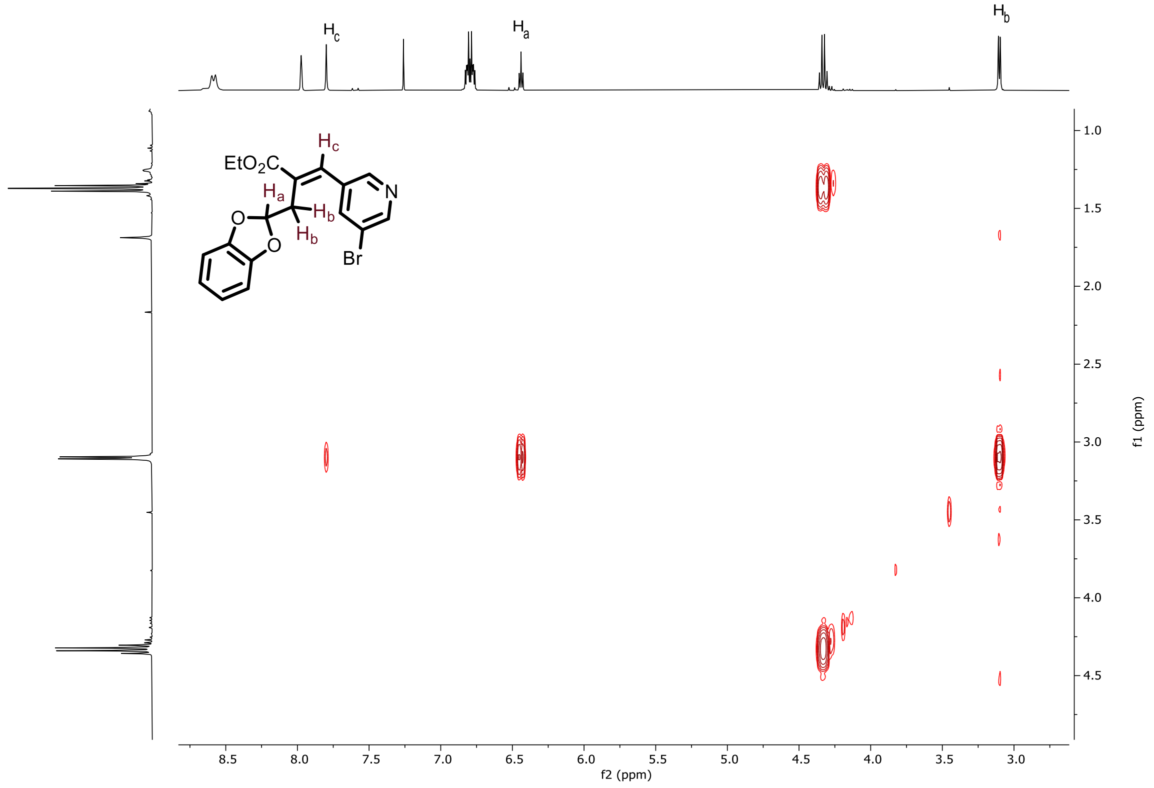

^1H–^1H NOESY (400 MHz, $CDCl_3$) of compound **38** (isomer 1, E)

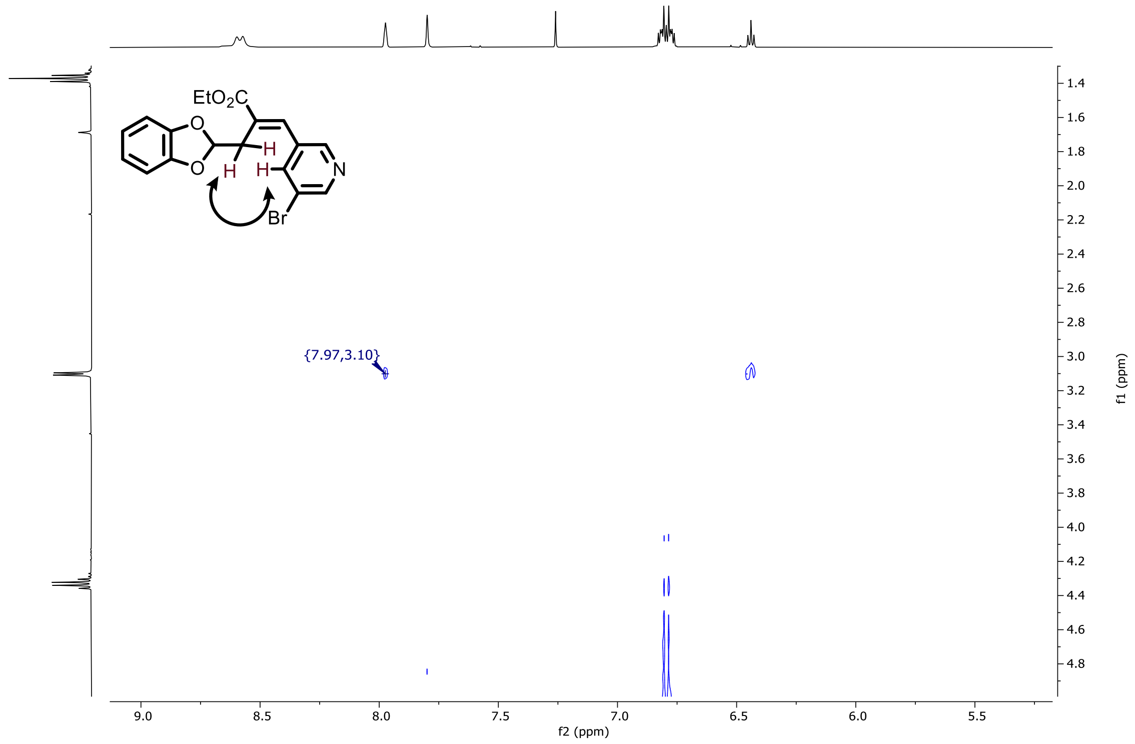

^{1}H NMR (400 MHz, $CDCl_3$) of compound **38** (isomer 2, Z)

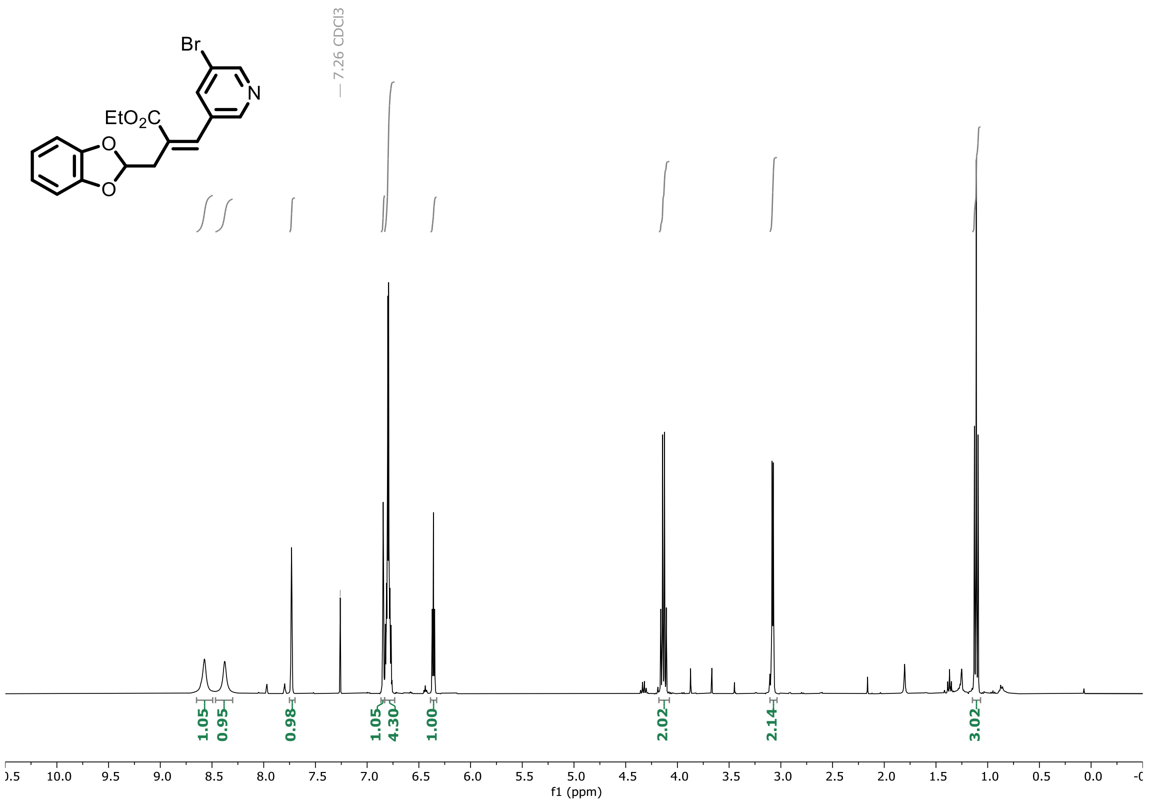

^{13}C NMR (101 MHz, $CDCl_3$) of compound **38** (isomer 2, Z)

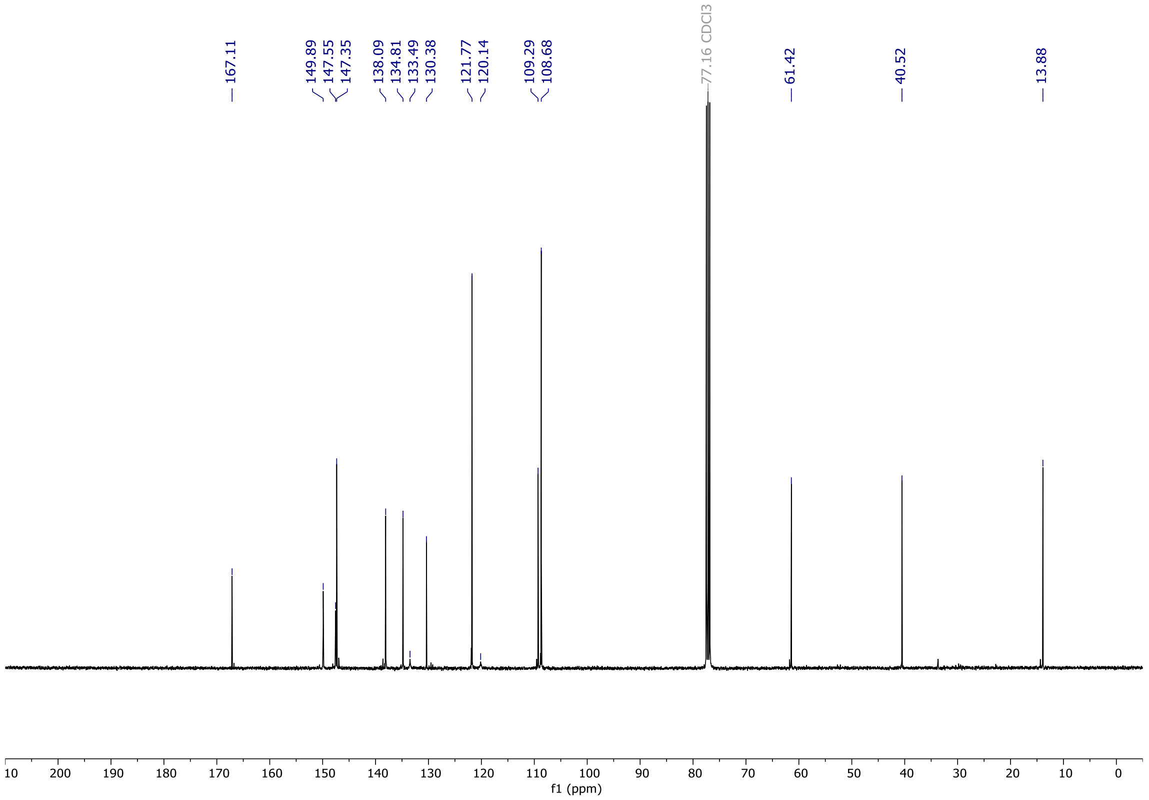

Key COSY (400 MHz, $CDCl_3$) correlations of compound **38** (isomer 2, Z)

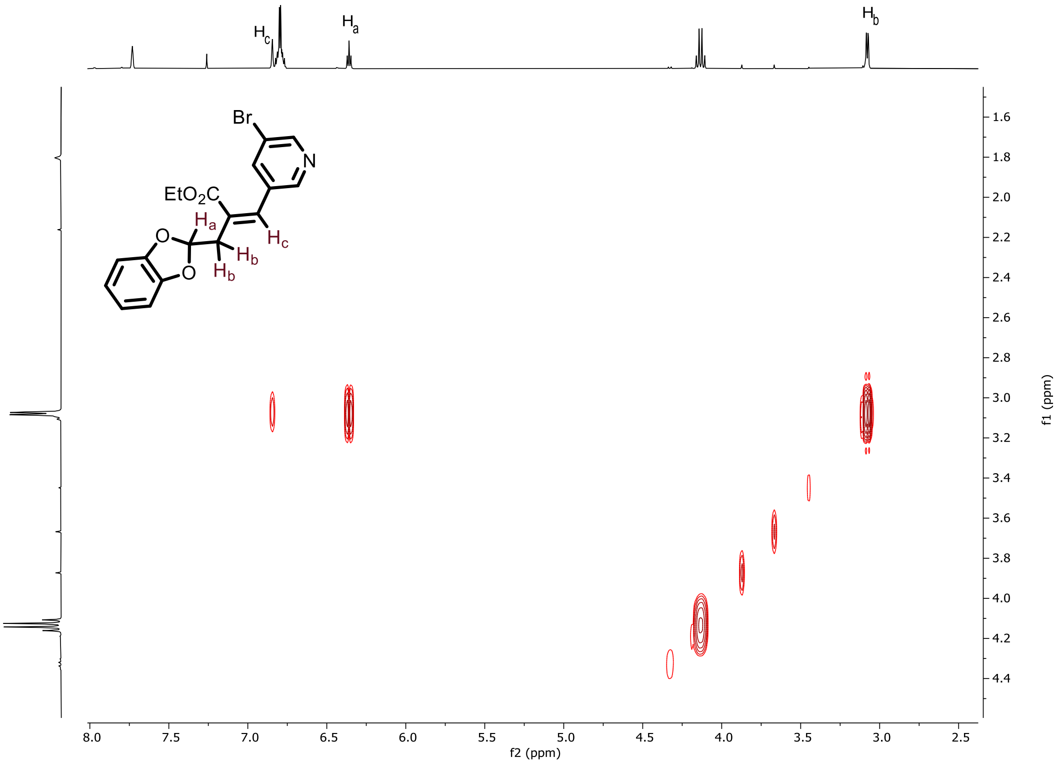

^1H–^1H NOESY (400 MHz, $CDCl_3$) of compound **38** (isomer 2, Z)

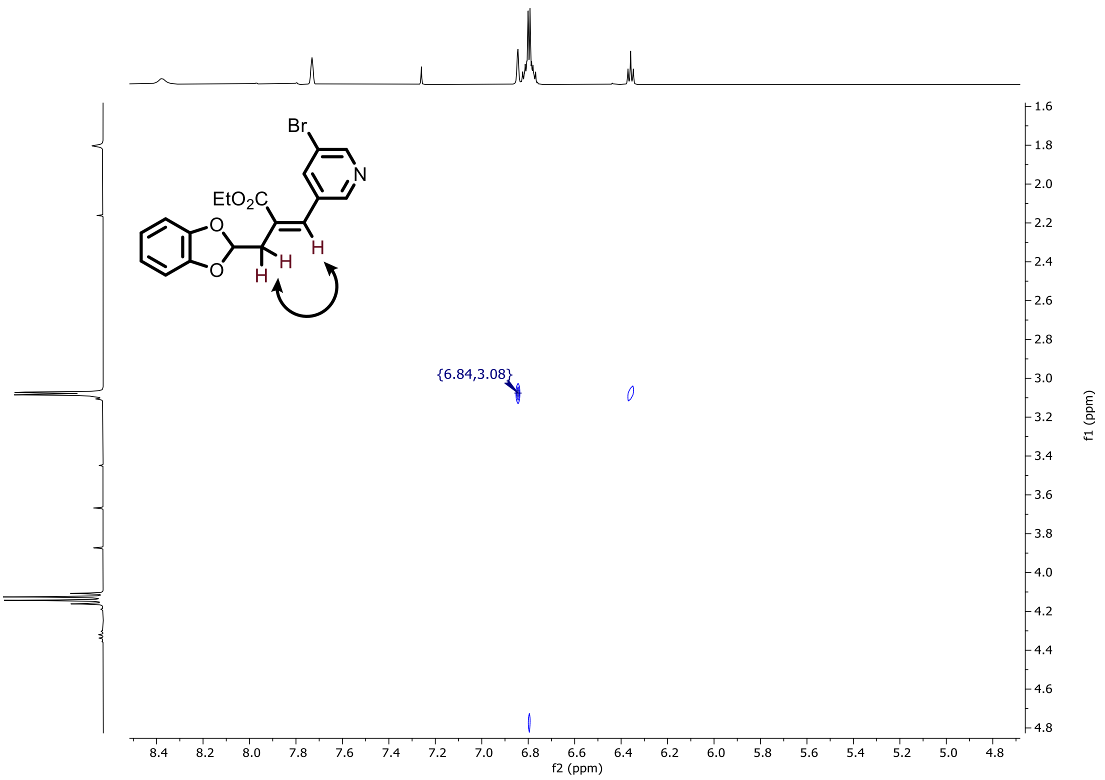

^{1}H NMR (400 MHz, $CDCl_3$) of compound **39** (major, E)

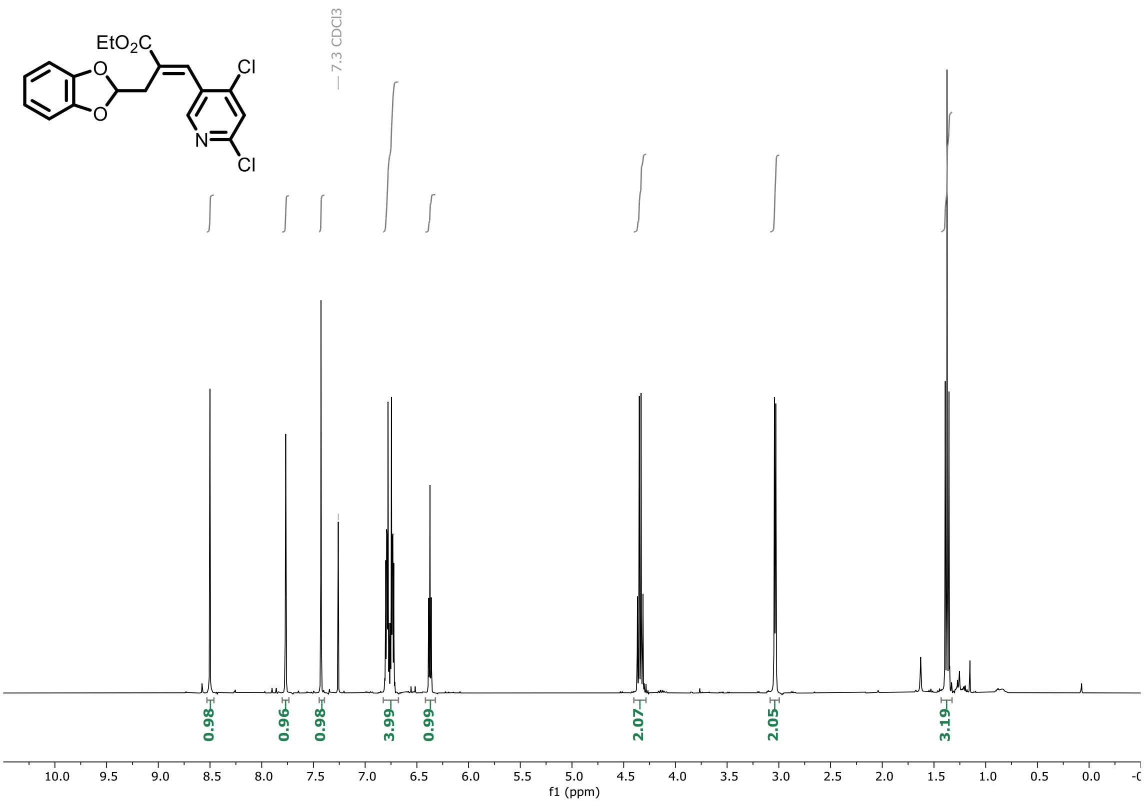

^{13}C NMR (101 MHz, $CDCl_3$) of compound **39** (major, E)

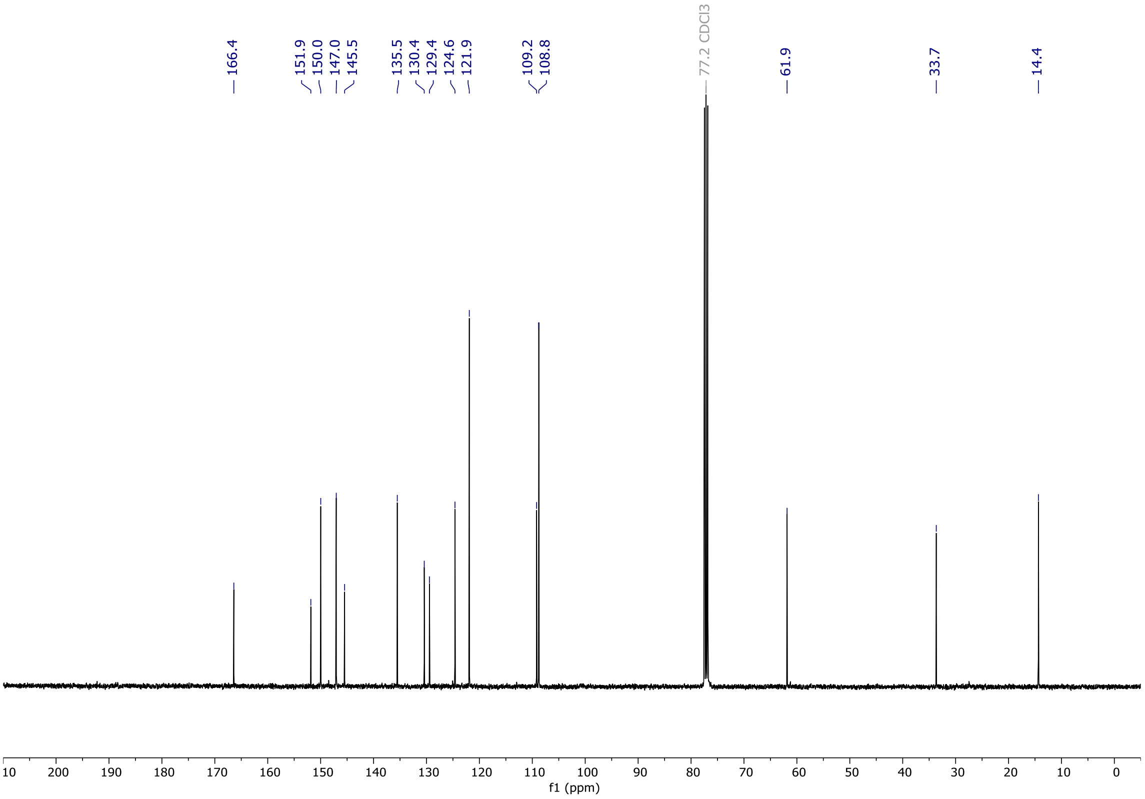

Key COSY (400 MHz, $CDCl_3$) correlations of compound **39** (major, E)

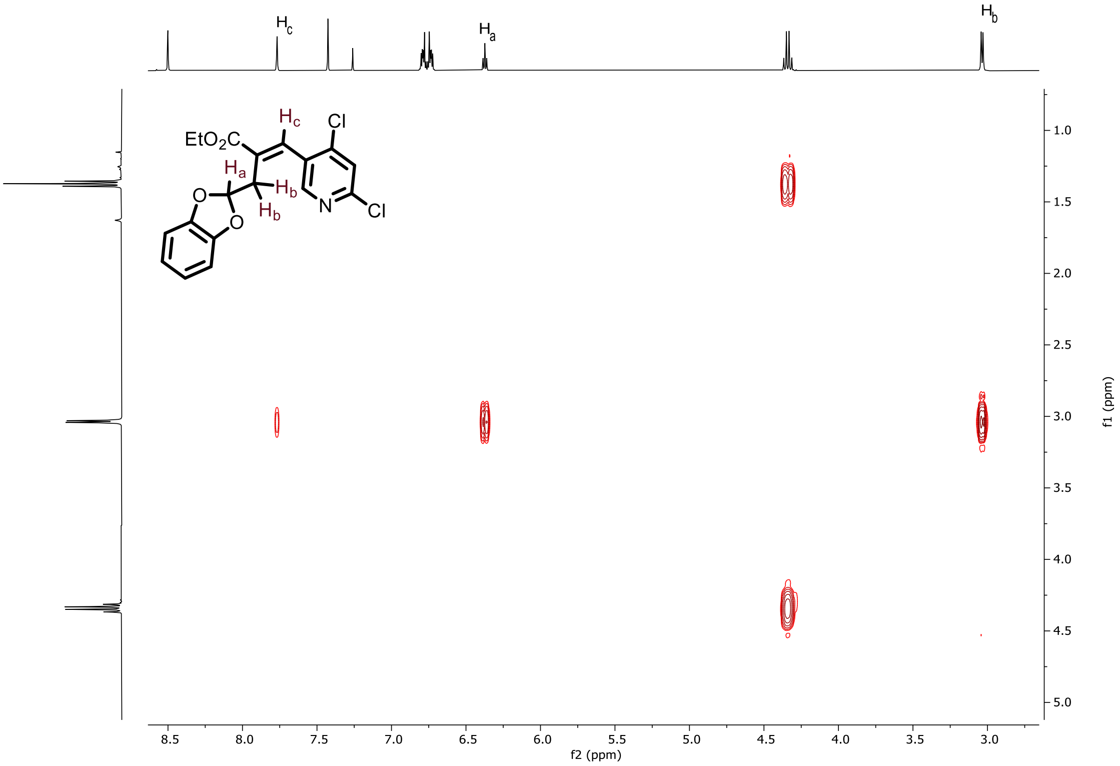

^{1}H–^{1}H NOESY (400 MHz, $CDCl_3$) of compound **39** (major, E)

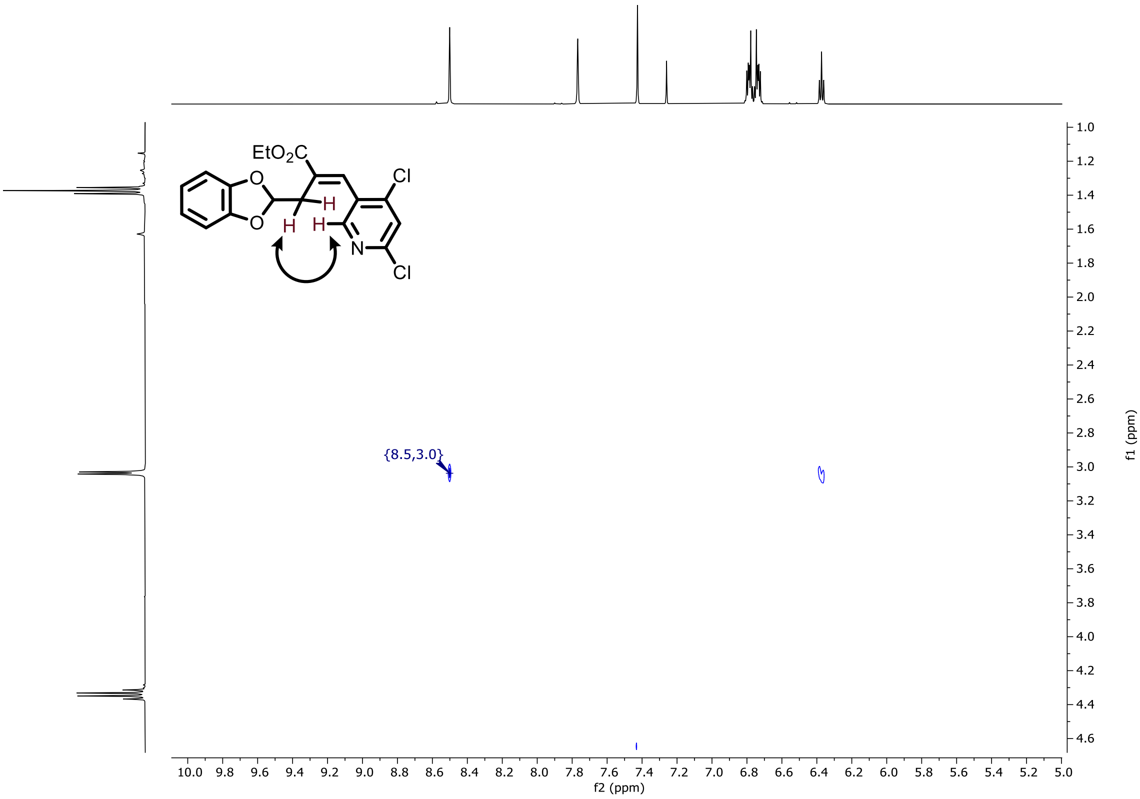

^{1}H NMR (400 MHz, $CDCl_3$) of compound **40** (major, E)

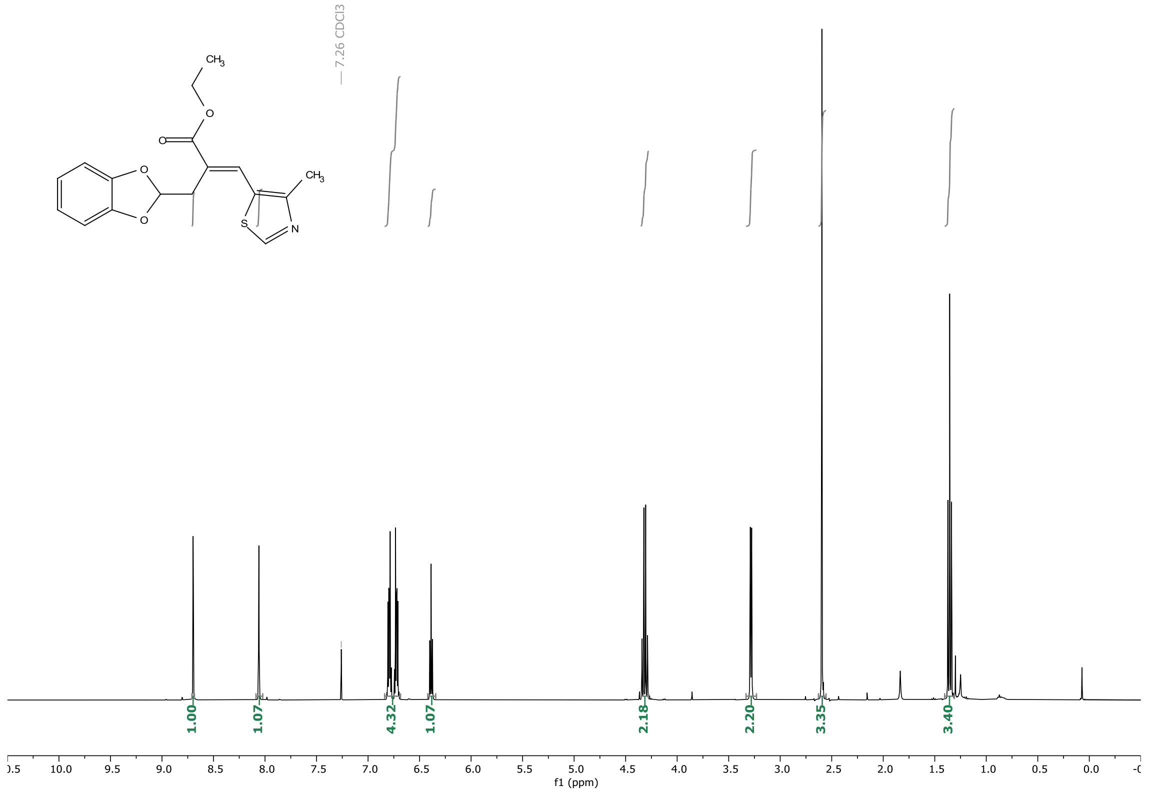

^{13}C NMR (101 MHz, $CDCl_3$) of compound **40** (major, E)

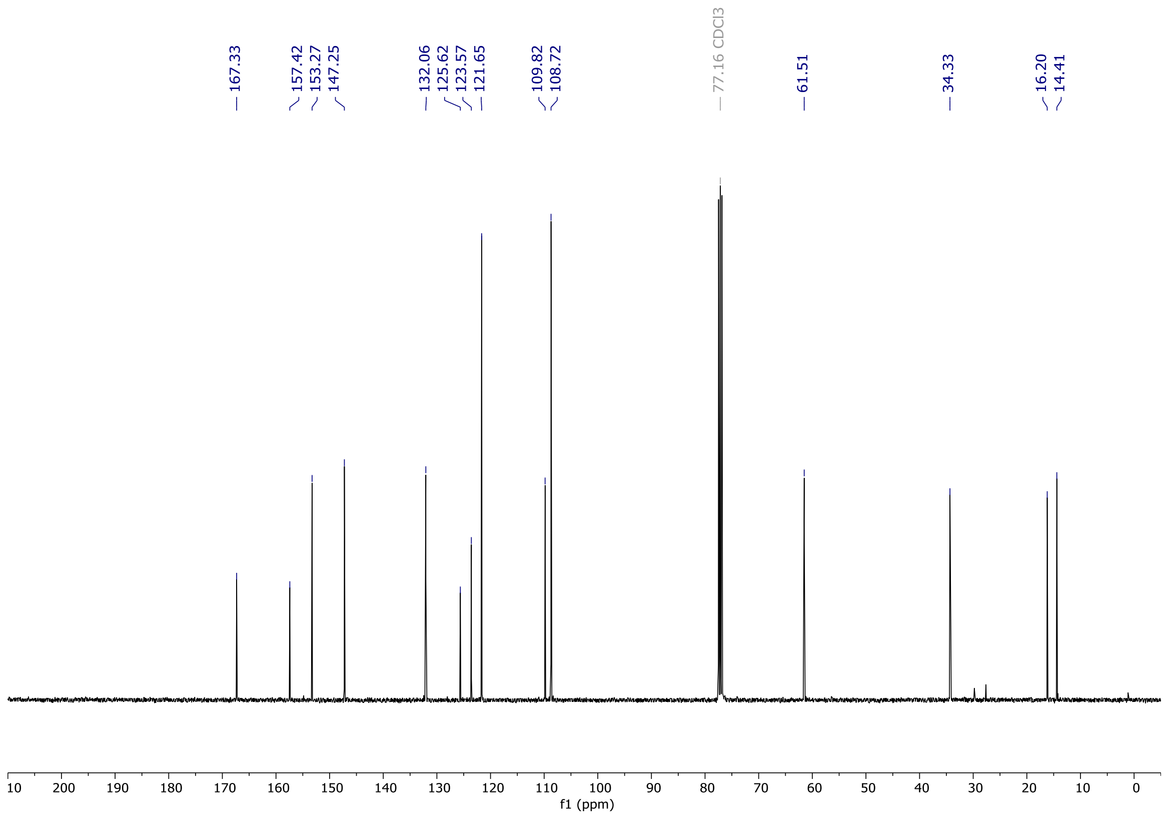

^{1}H NMR (400 MHz, $CDCl_3$) of compound **41**

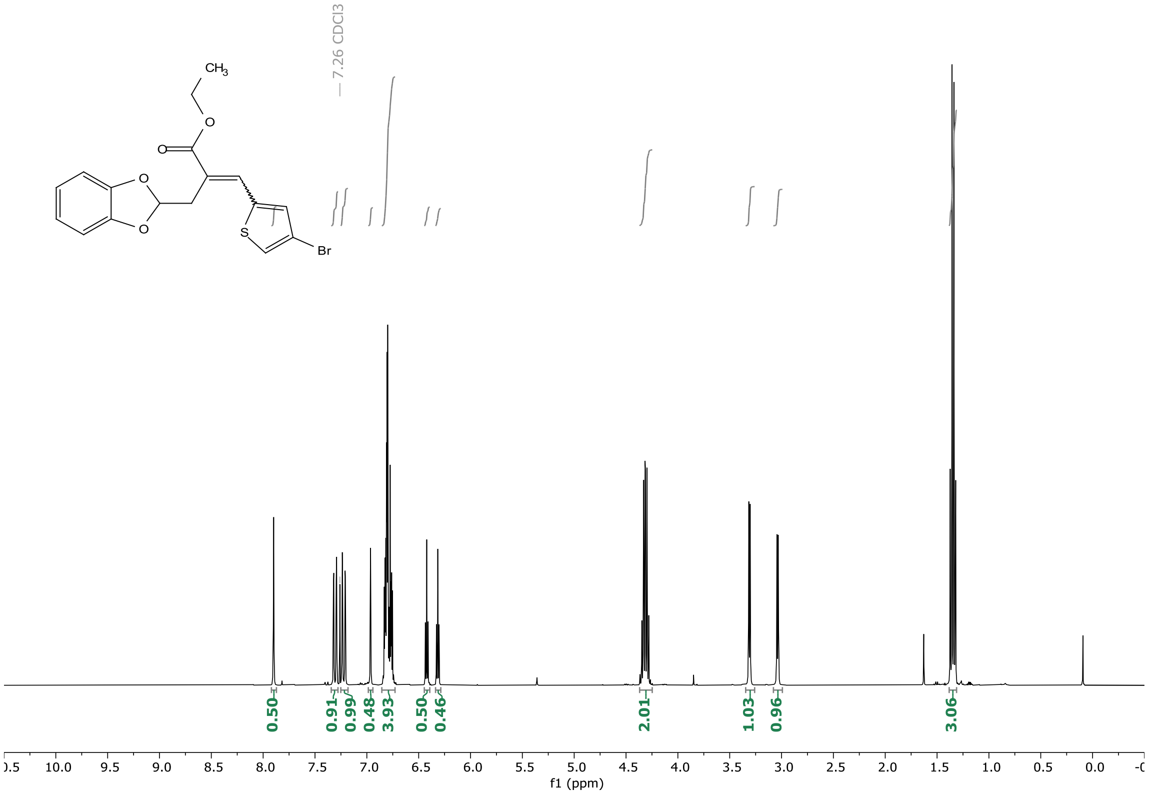

^{13}C NMR (101 MHz, $CDCl_3$) of compound **41**

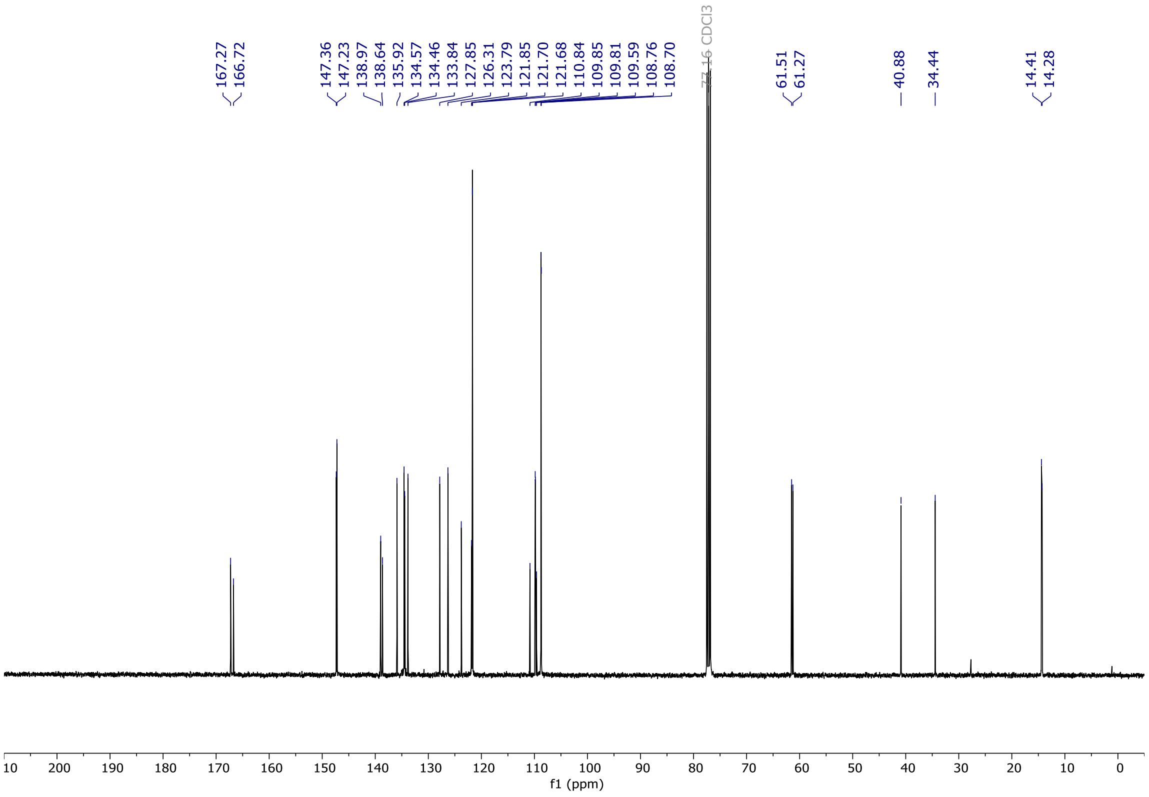

^1H NMR (400 MHz, $CDCl_3$) of compound **42** (major, E)

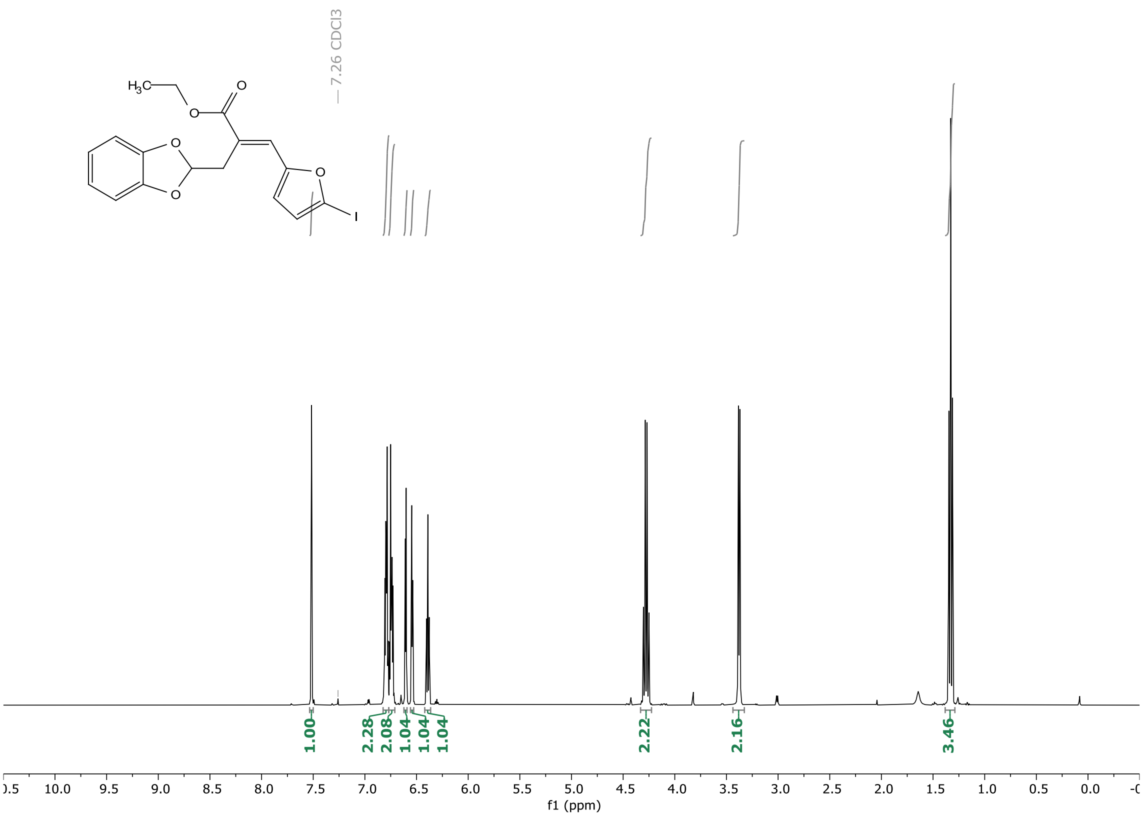

^{13}C NMR (101 MHz, $CDCl_3$) of compound **42** (major, E)

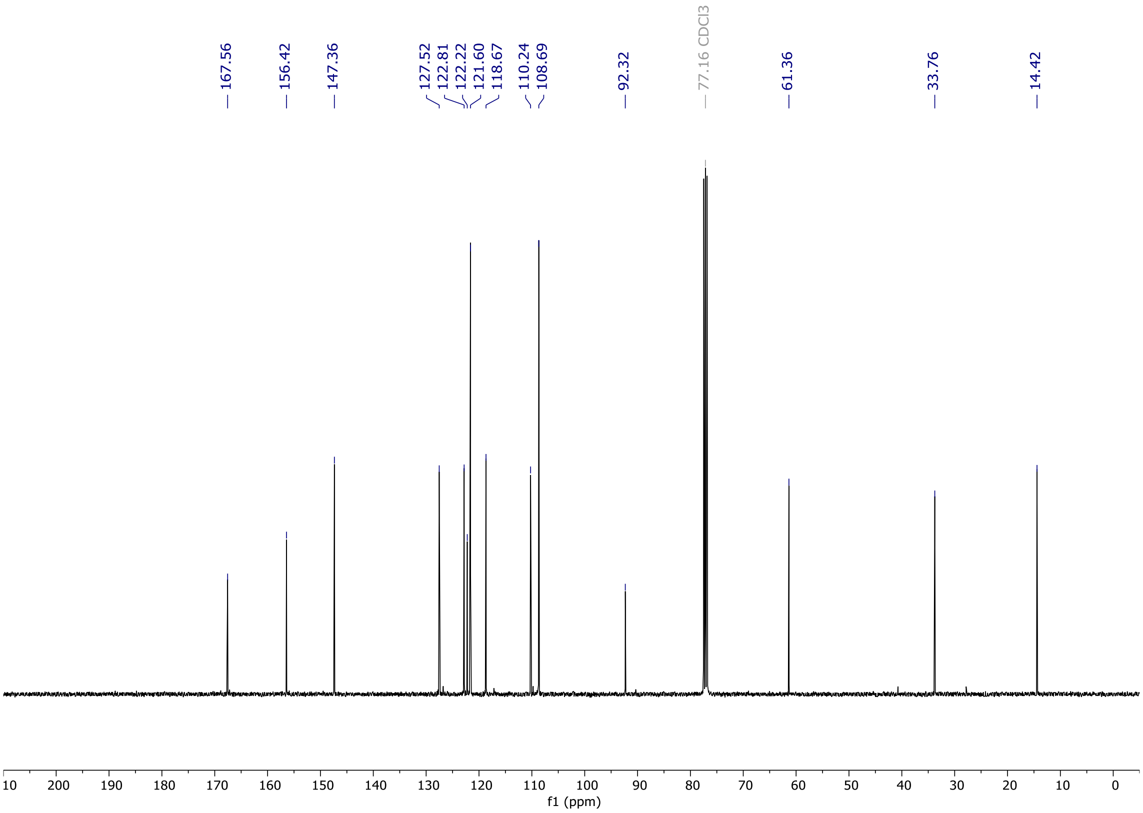

^{1}H–^{1}H NOESY (400 MHz, $CDCl_3$) of compound **42** (major, E)

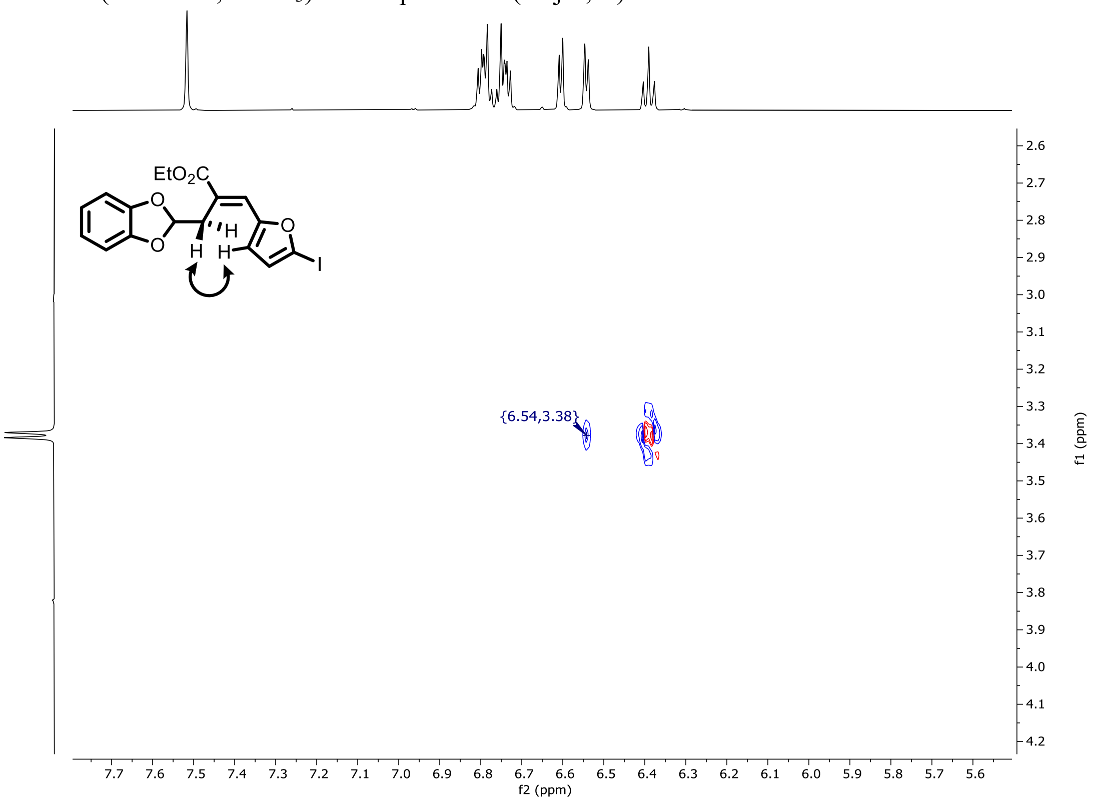

^{1}H NMR (400 MHz, $CDCl_3$) of compound **42** (minor, Z)

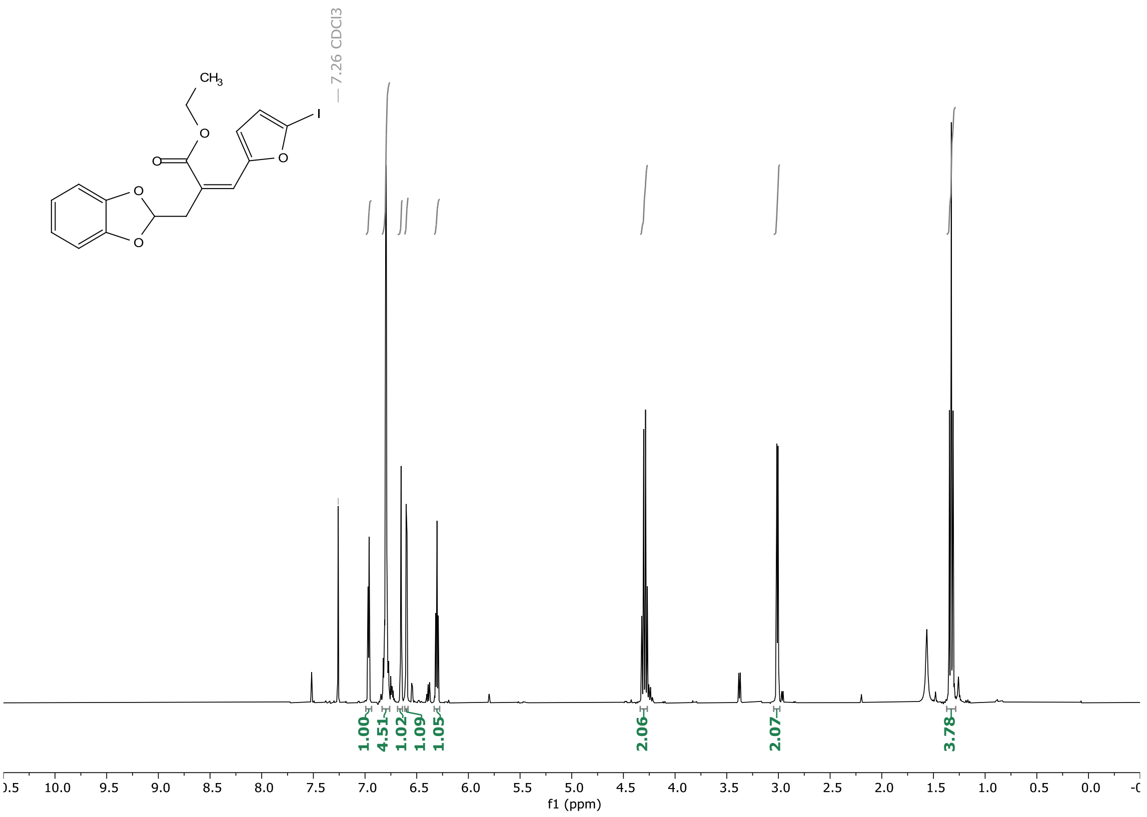

^{13}C NMR (101 MHz, $CDCl_3$) of compound **42** (minor, Z)

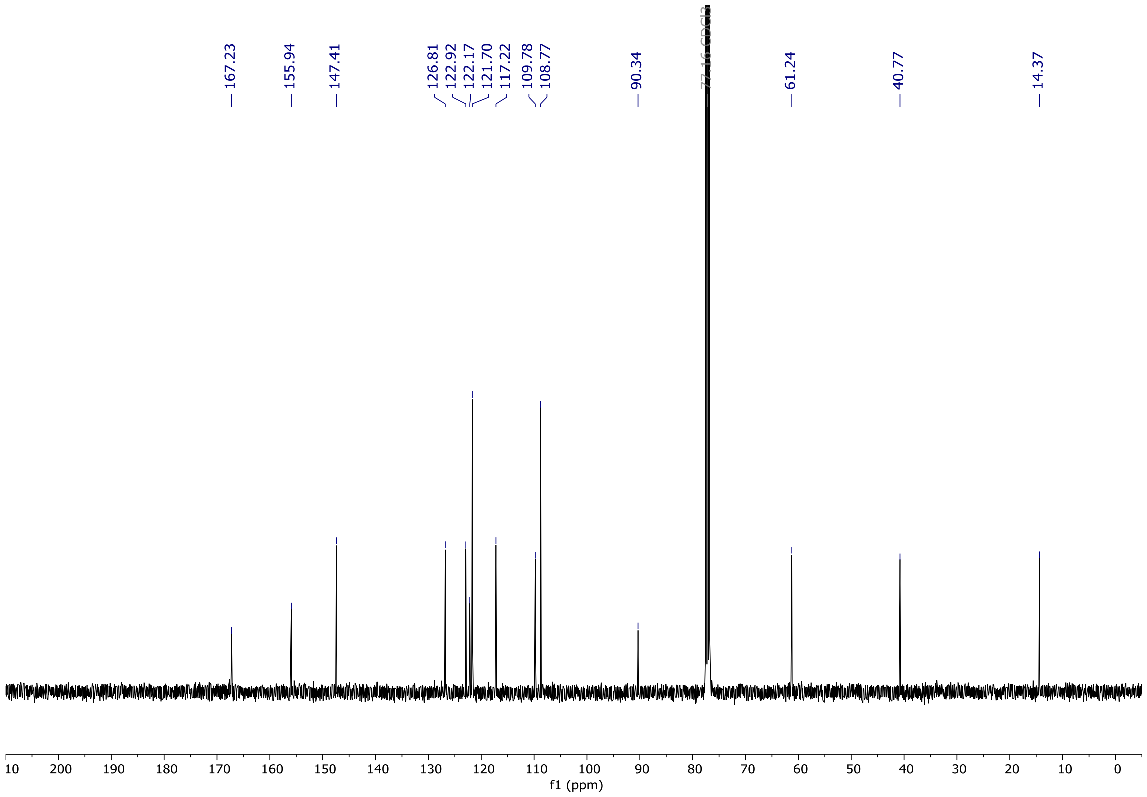

^{1}H–^{1}H NOESY (400 MHz, $CDCl_3$) of compound **42** (minor, Z)

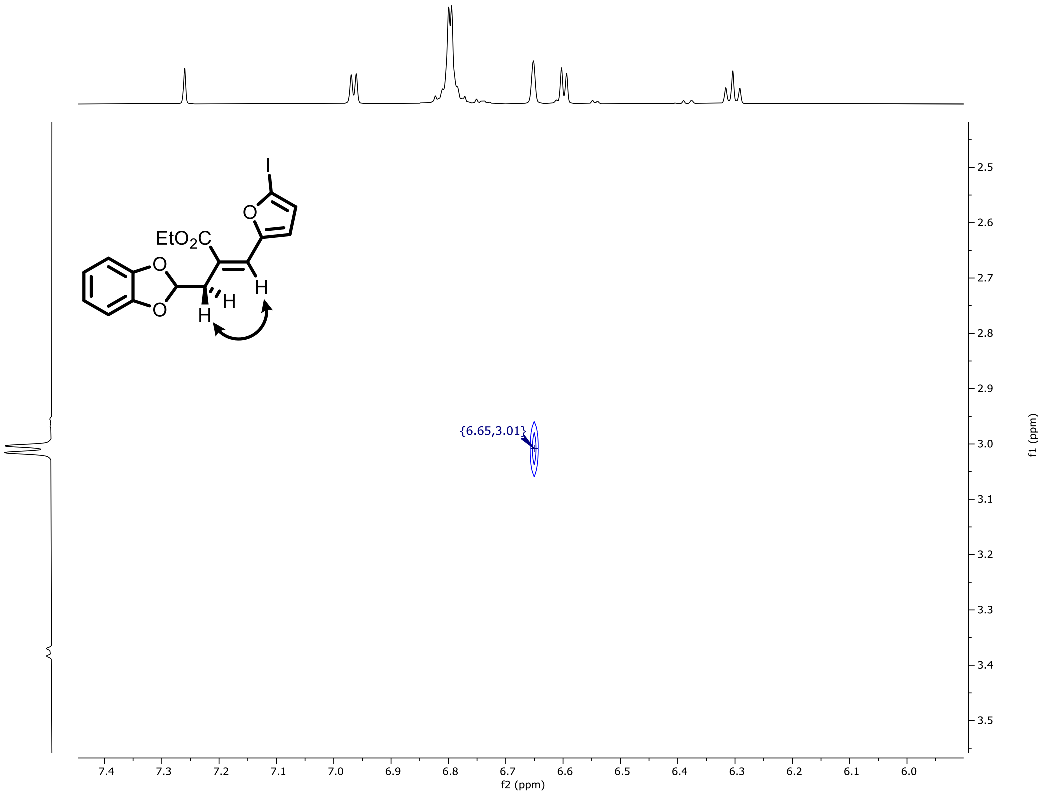

^{1}H NMR (400 MHz, $CDCl_3$) of compound **43** (E isomer)

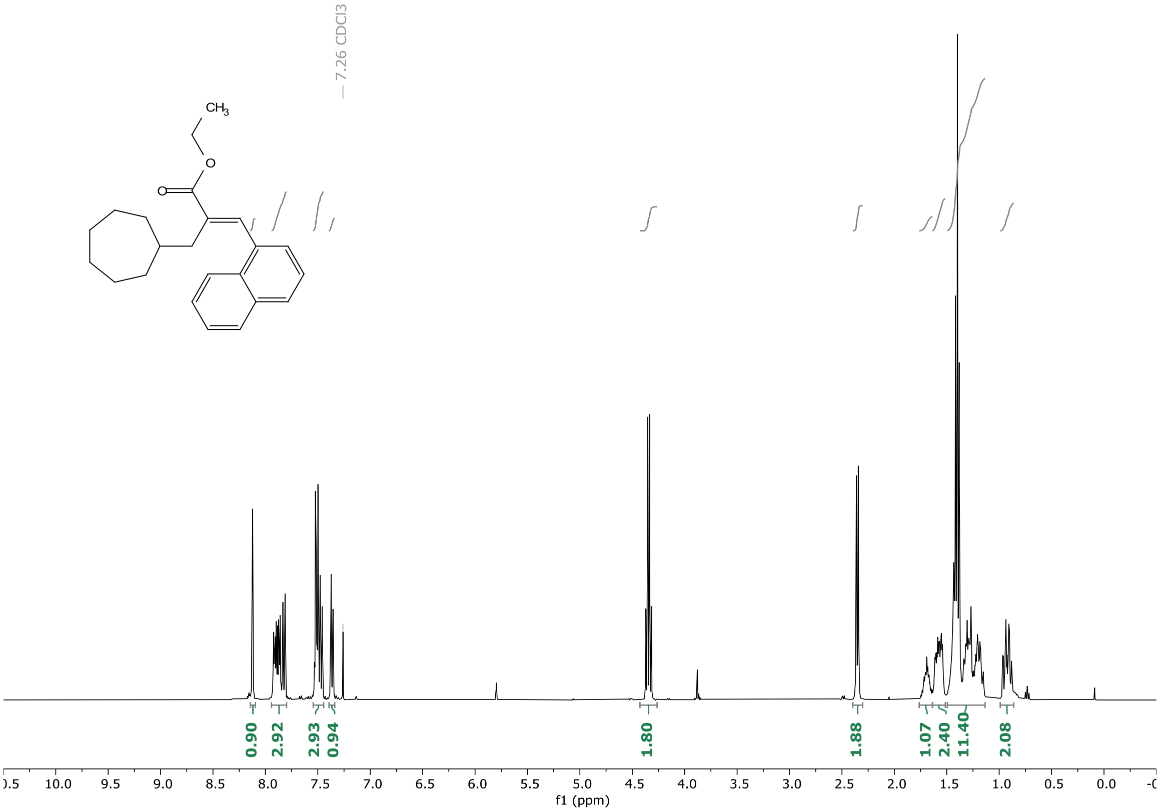

^{13}C NMR (101 MHz, $CDCl_3$) of compound **43** (E isomer)

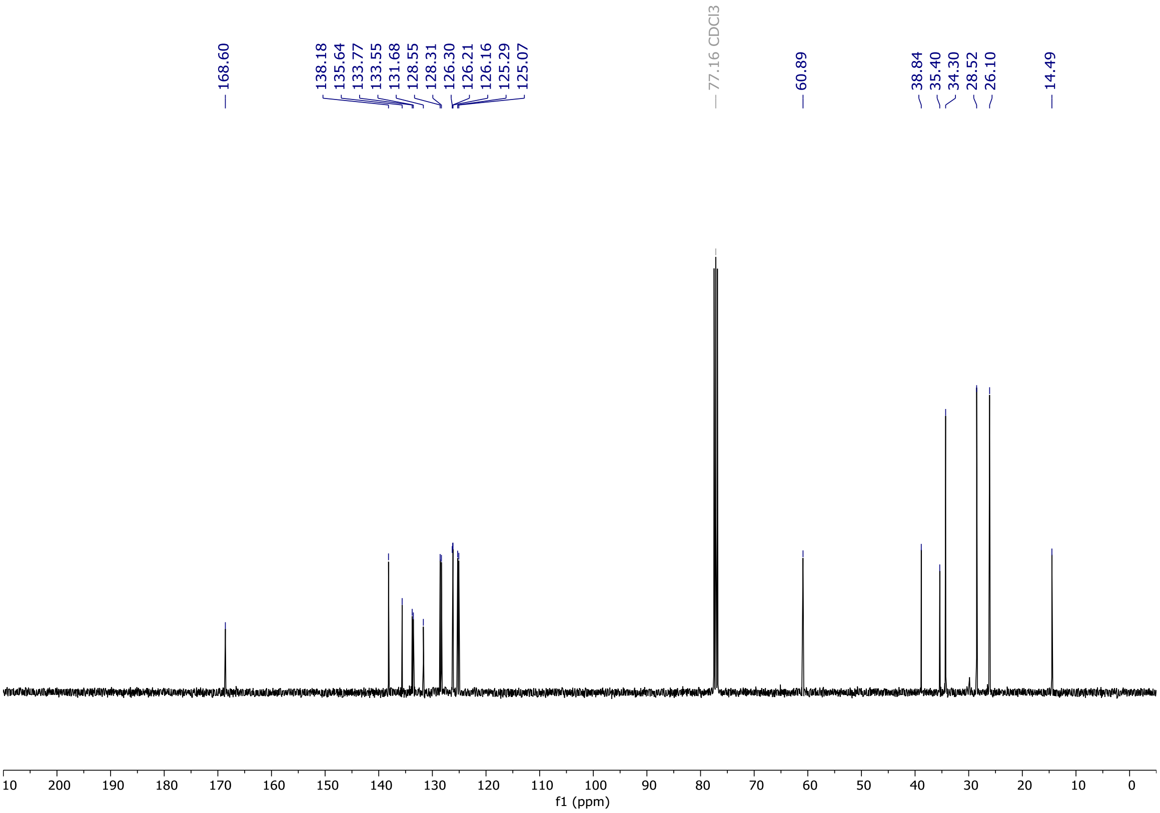

Key COSY (400 MHz, $CDCl_3$) correlations of compound **43** (E isomer)

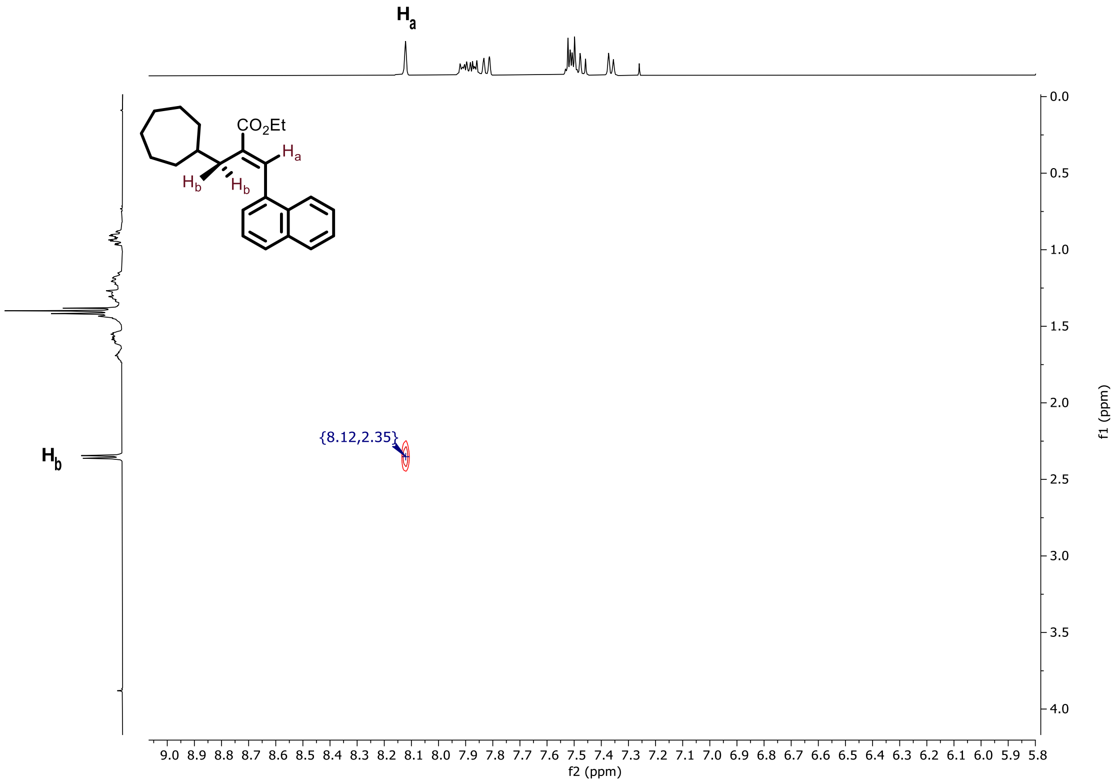

Key HSQC correlations of compound **43** (E isomer)

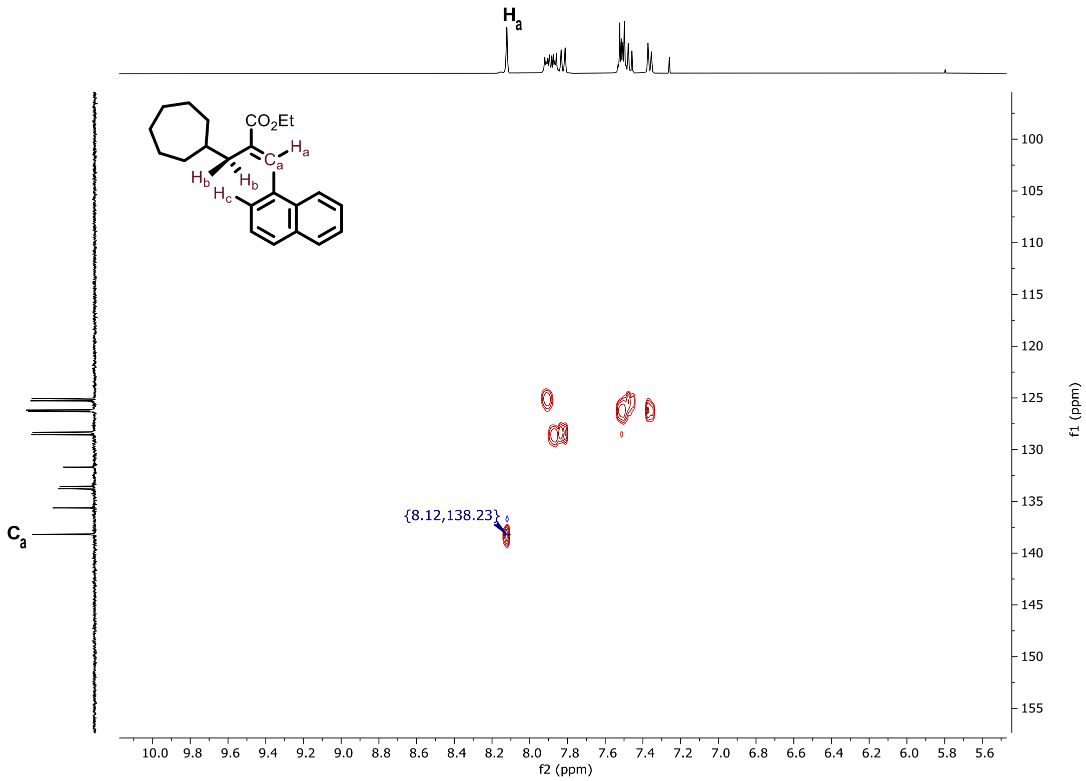

Key HMBC correlations of compound **43** (E isomer)

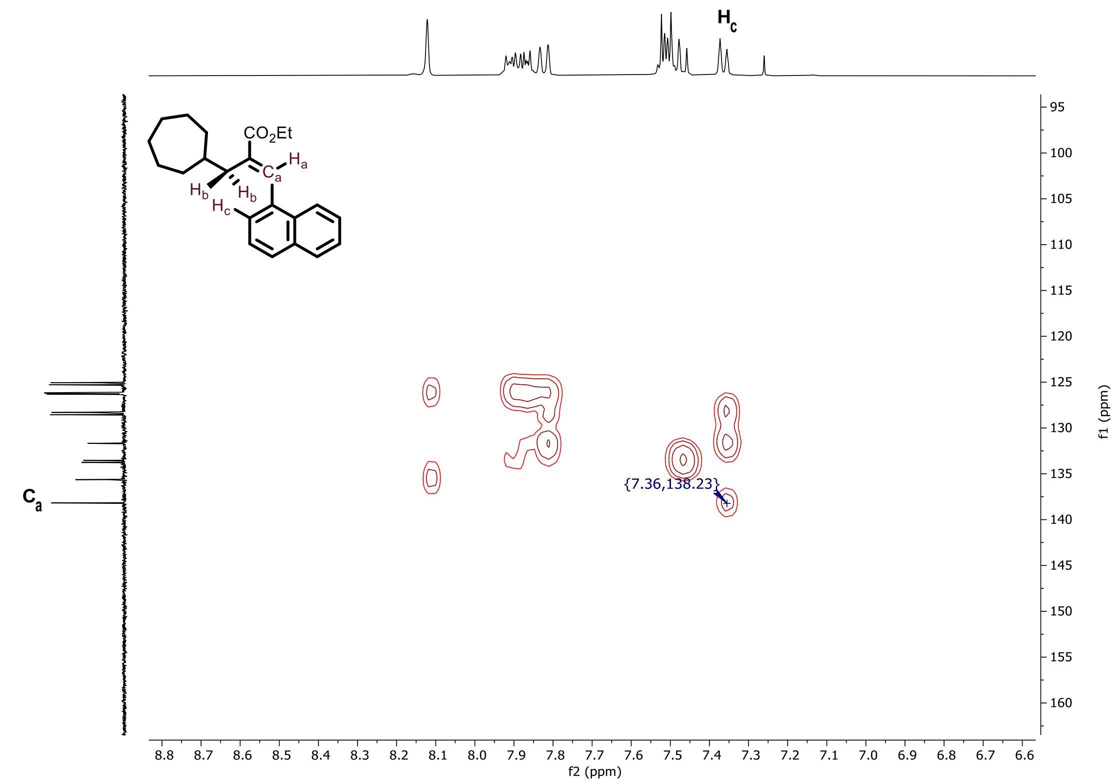

^{1}H–^{1}H NOESY (400 MHz, $CDCl_3$) of compound **43** (E isomer)

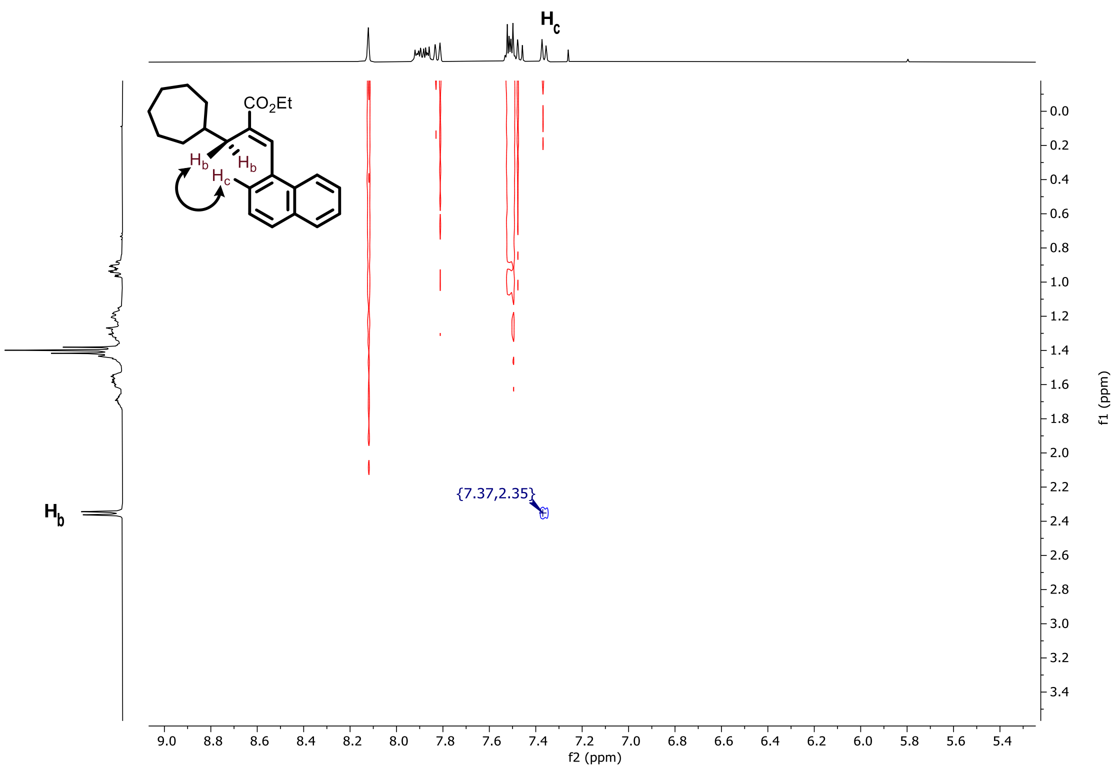

^{1}H NMR (400 MHz, $CDCl_3$) of compound **44** (E isomer)

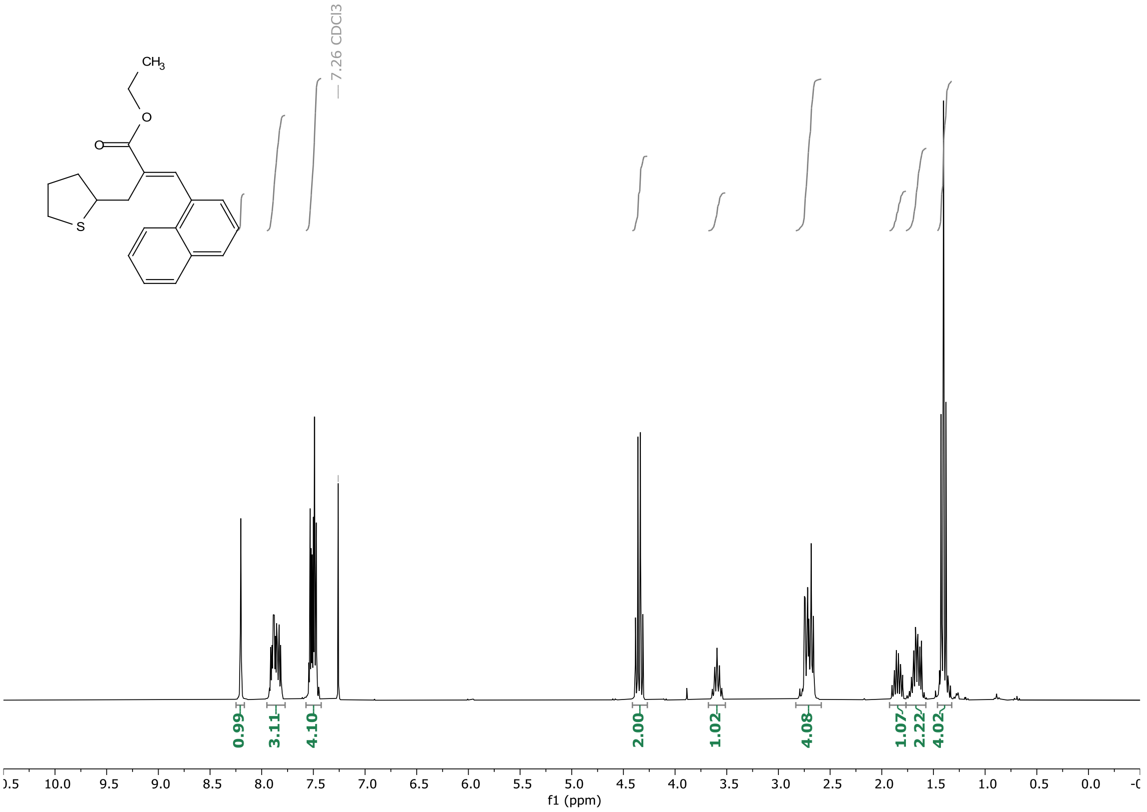

^{13}C NMR (101 MHz, $CDCl_3$) of compound **44** (E isomer)

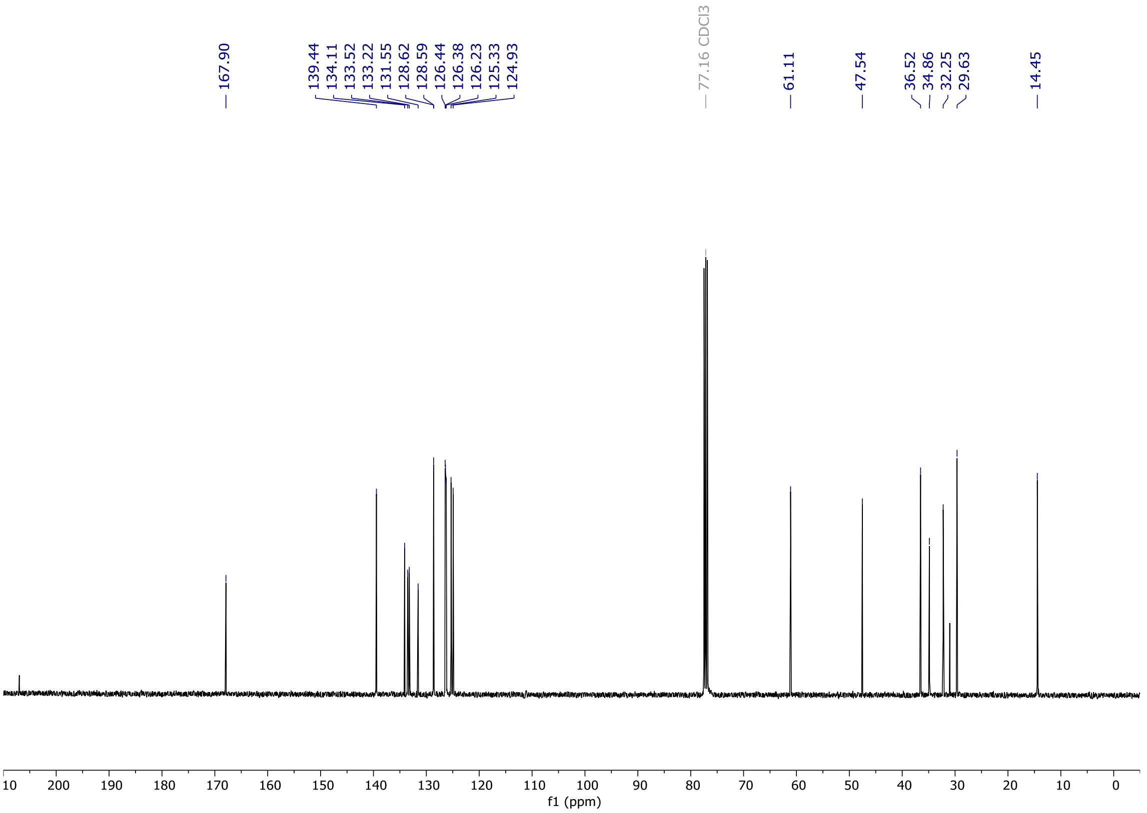

^{1}H–^{1}H NOESY (400 MHz, $CDCl_3$) of compound **44** (E isomer)

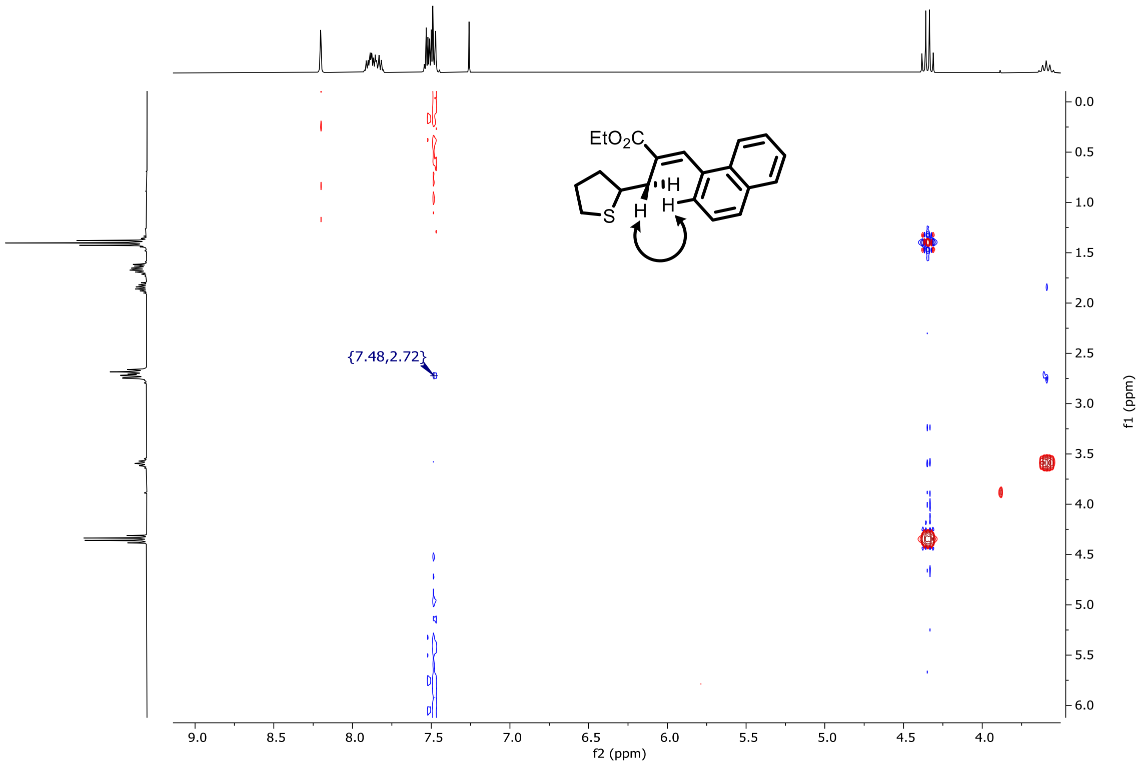

^{1}H NMR (400 MHz, $CDCl_3$) of compound **45** (E isomer)

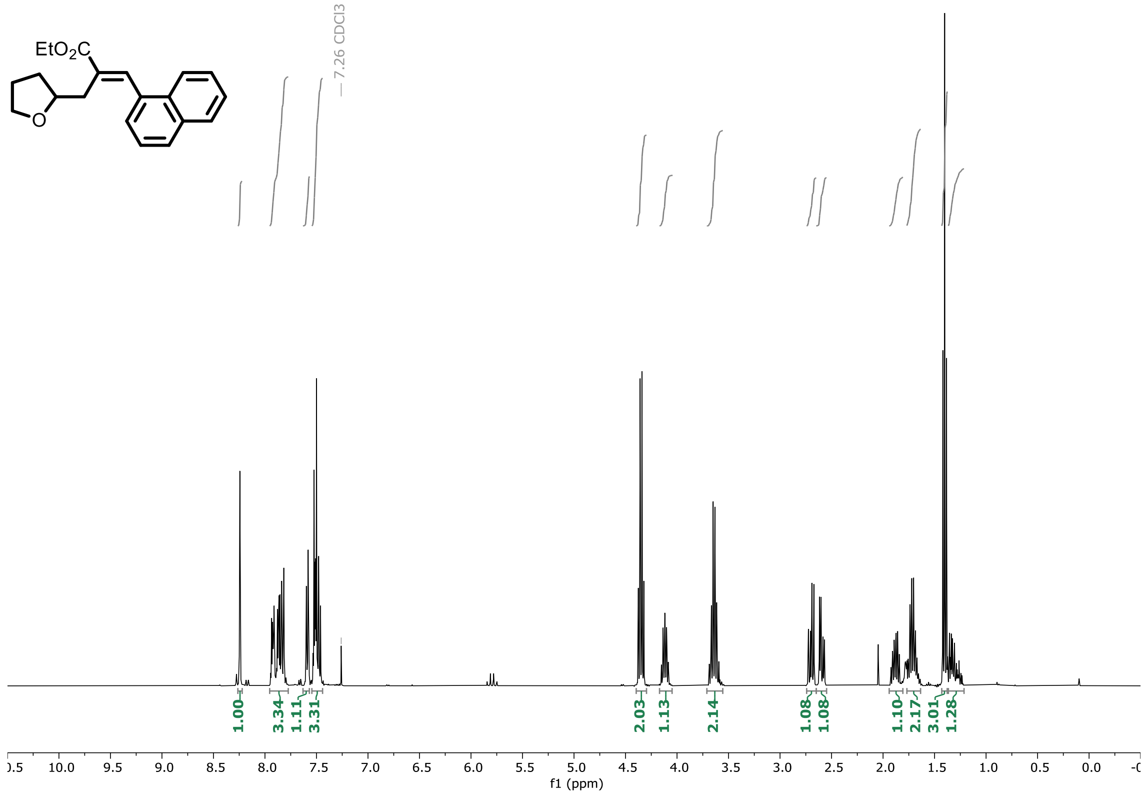

^{13}C NMR (101 MHz, $CDCl_3$) of compound **45** (E isomer)

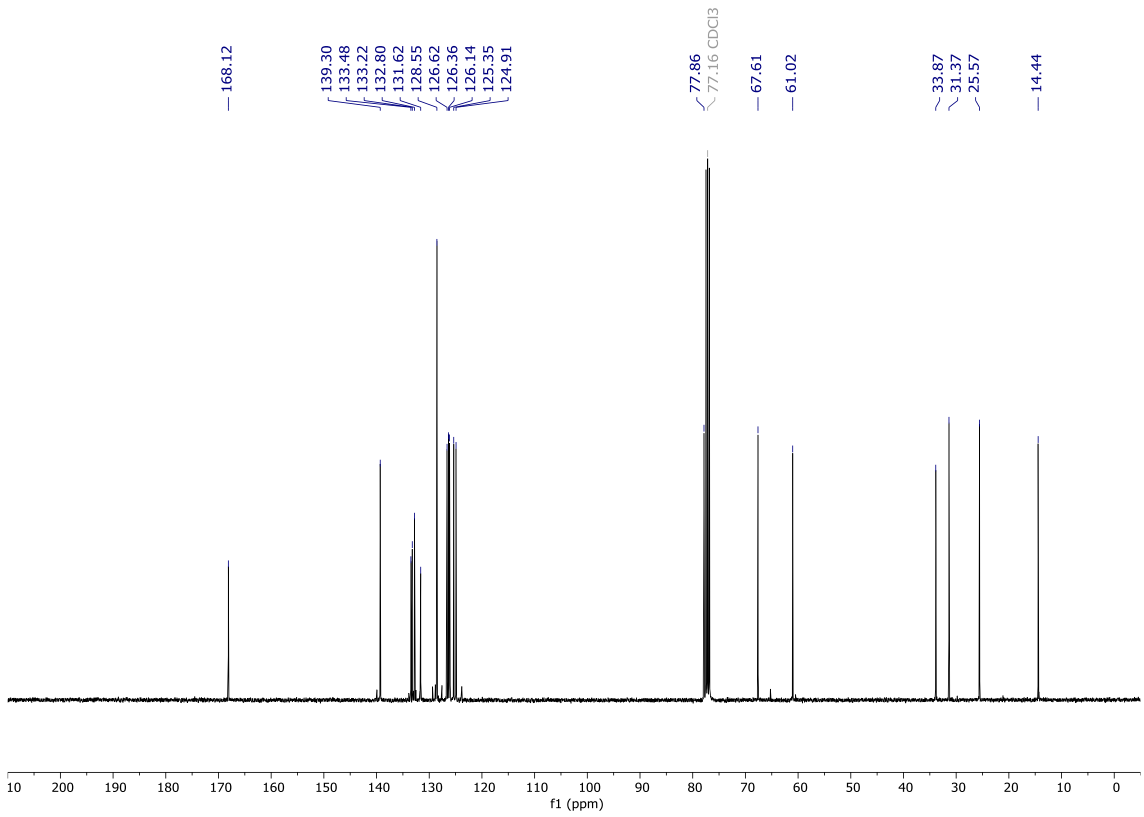

Key COSY (400 MHz, $CDCl_3$) correlations of compound **45** (E isomer)

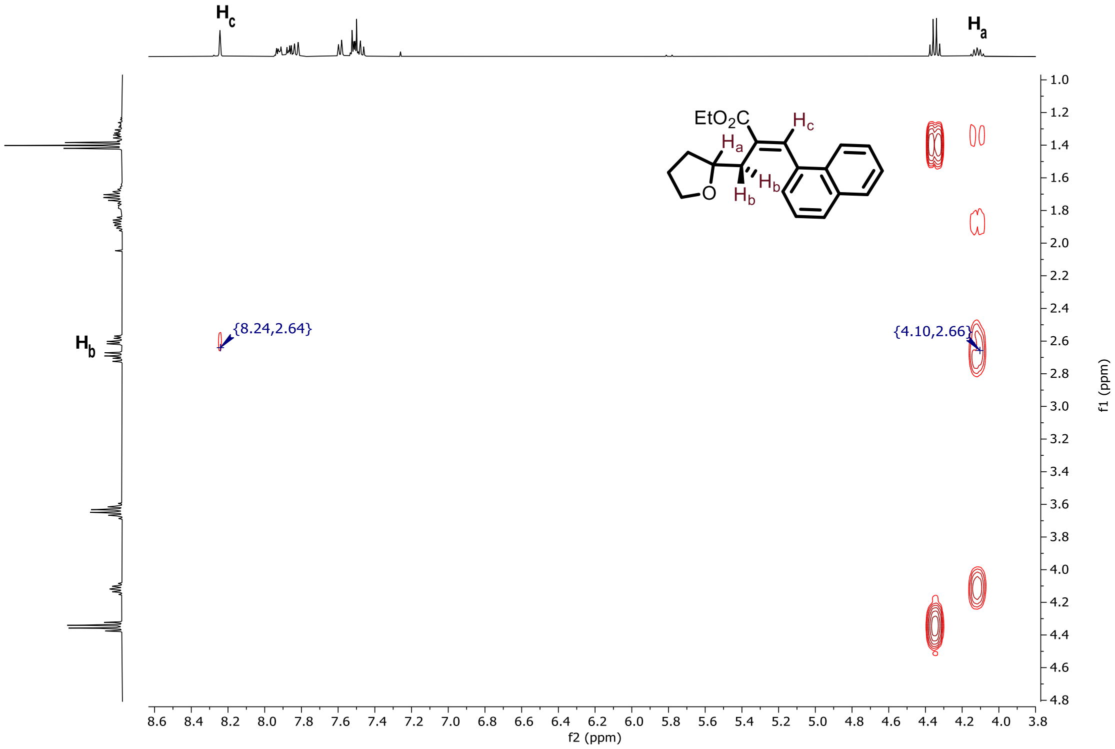

^1H–^1H NOESY (400 MHz, $CDCl_3$) of compound **45** (E isomer)

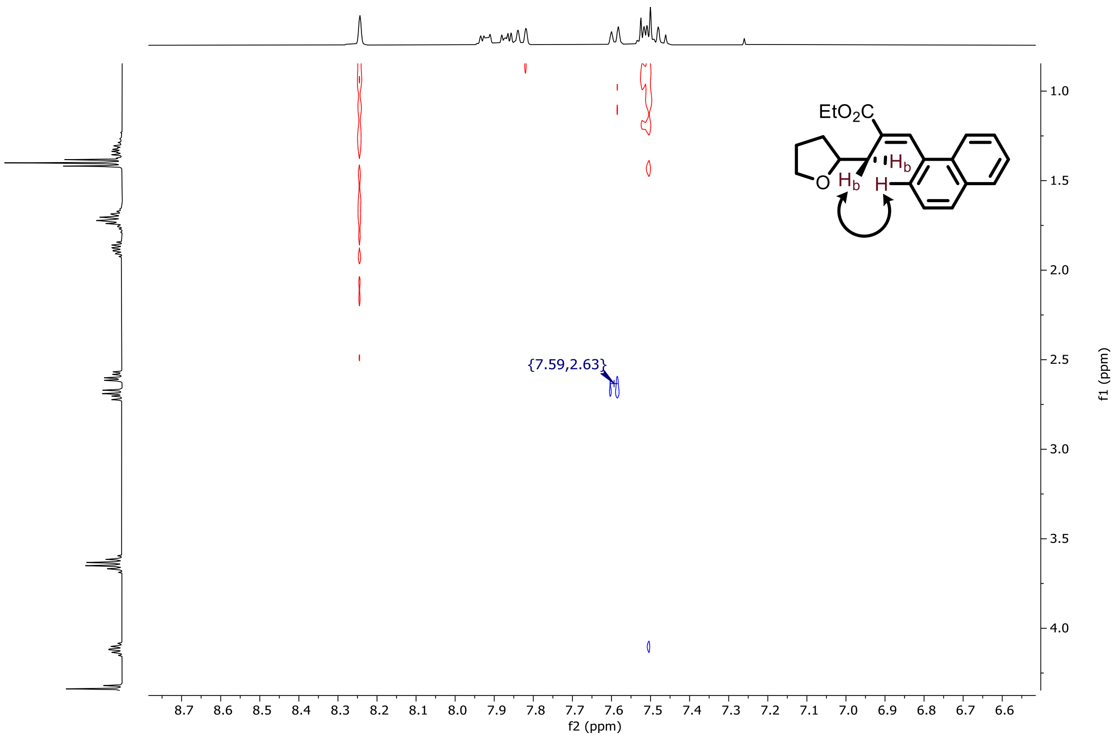

^{1}H NMR (400 MHz, $CDCl_3$) of compound **46** (E isomer)

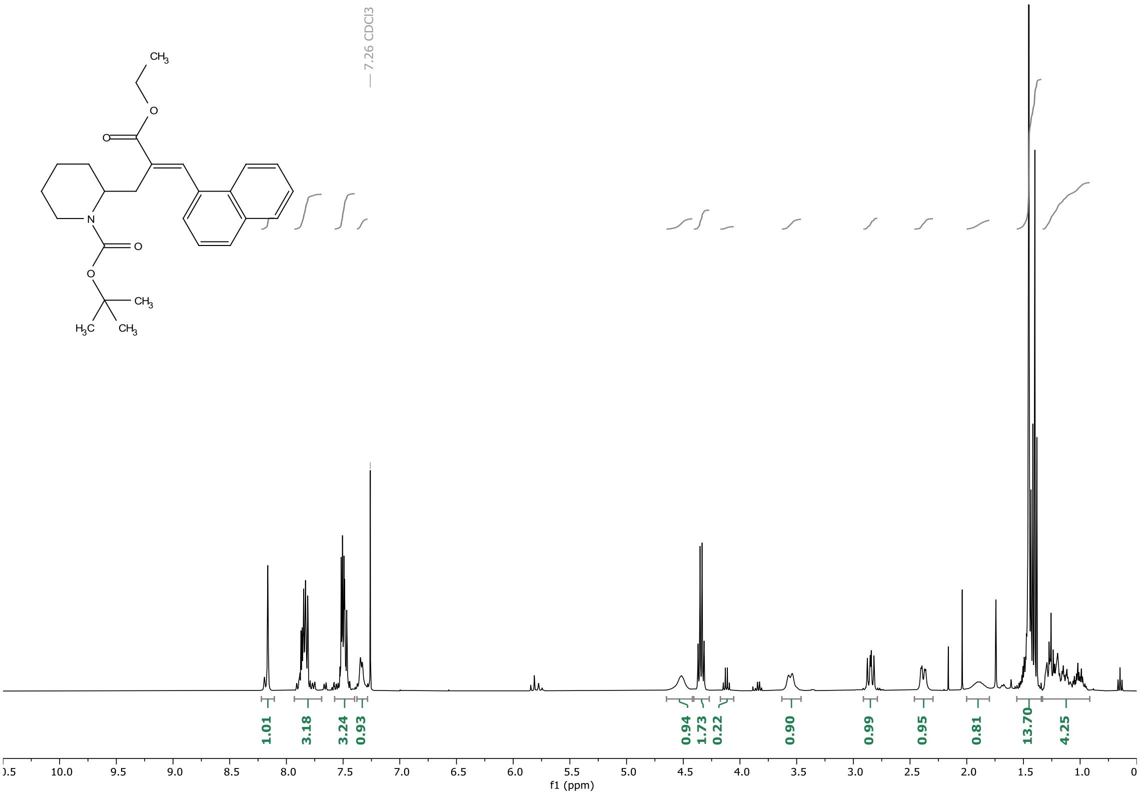

^{13}C NMR (101 MHz, $CDCl_3$) of compound **46** (E isomer)

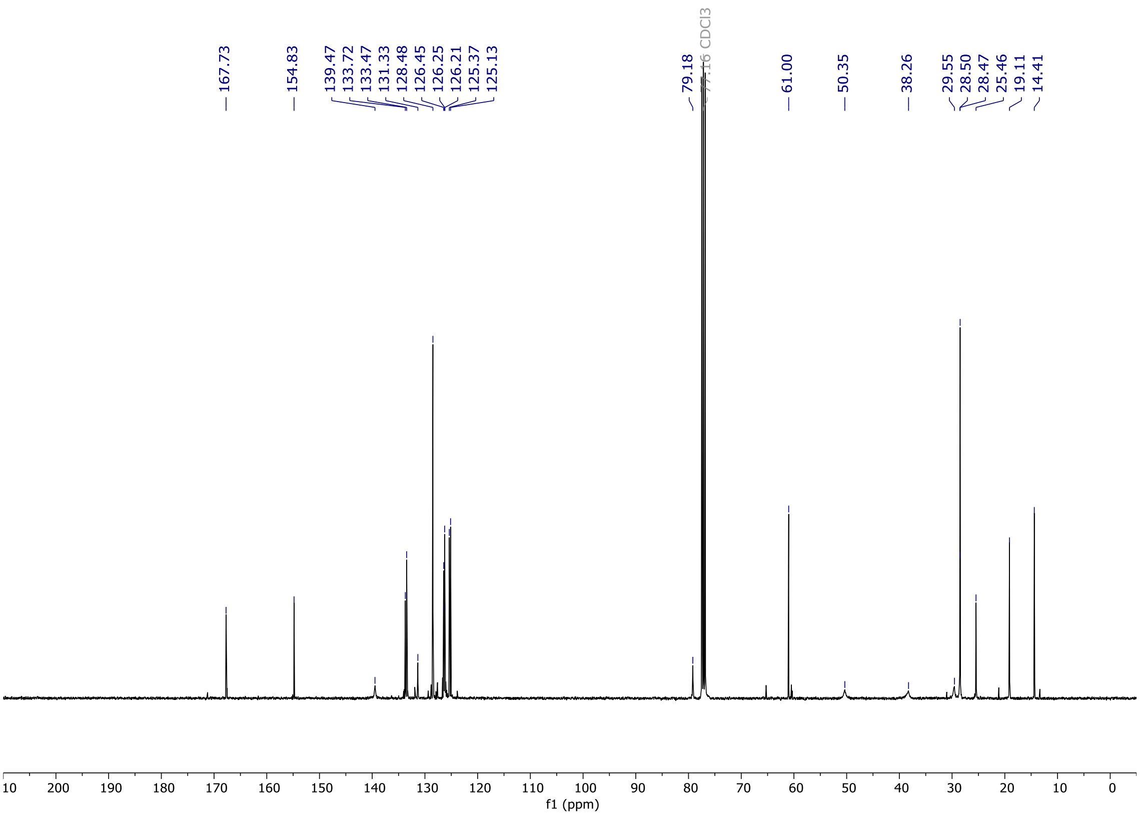

Key HSQC of compound **46** (E isomer)

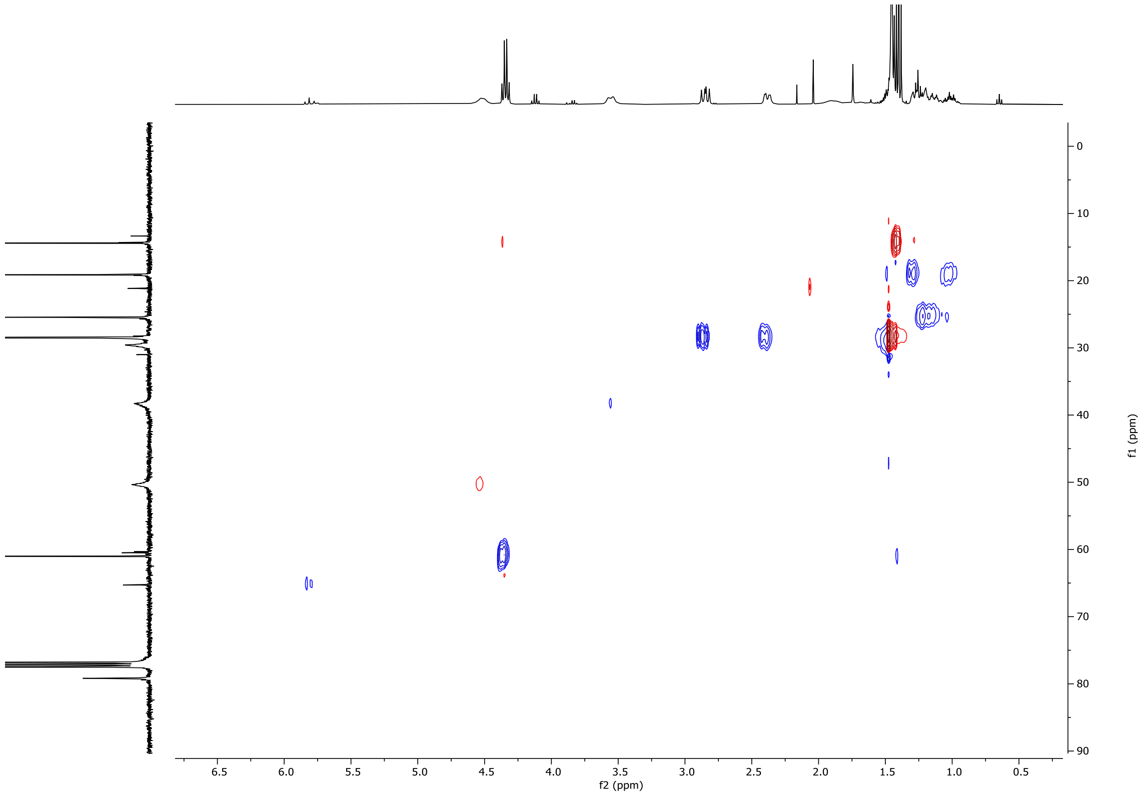

^{1}H NMR (400 MHz, $CDCl_3$) of compound **47** (E isomer)

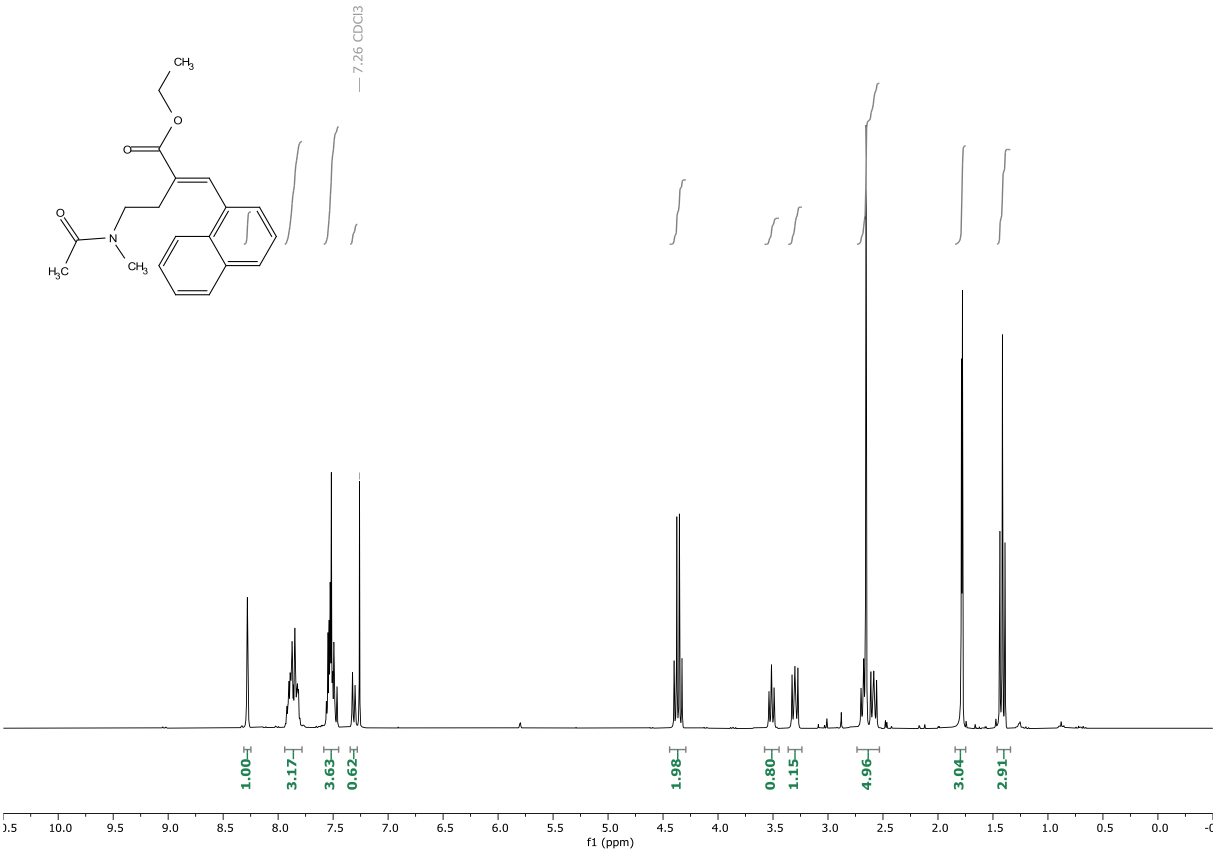

^{13}C NMR (101 MHz, $CDCl_3$) of compound **47** (E isomer)

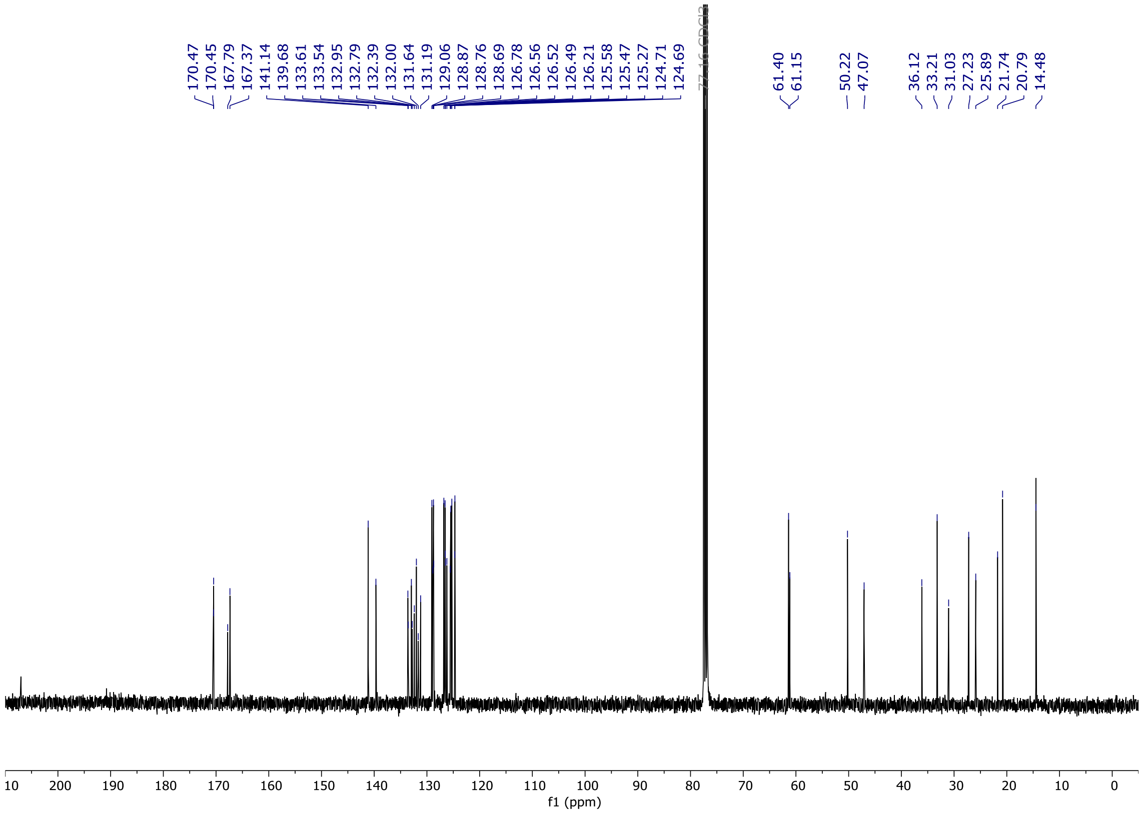

^{1}H NMR (400 MHz, $CDCl_3$) of compound **48**

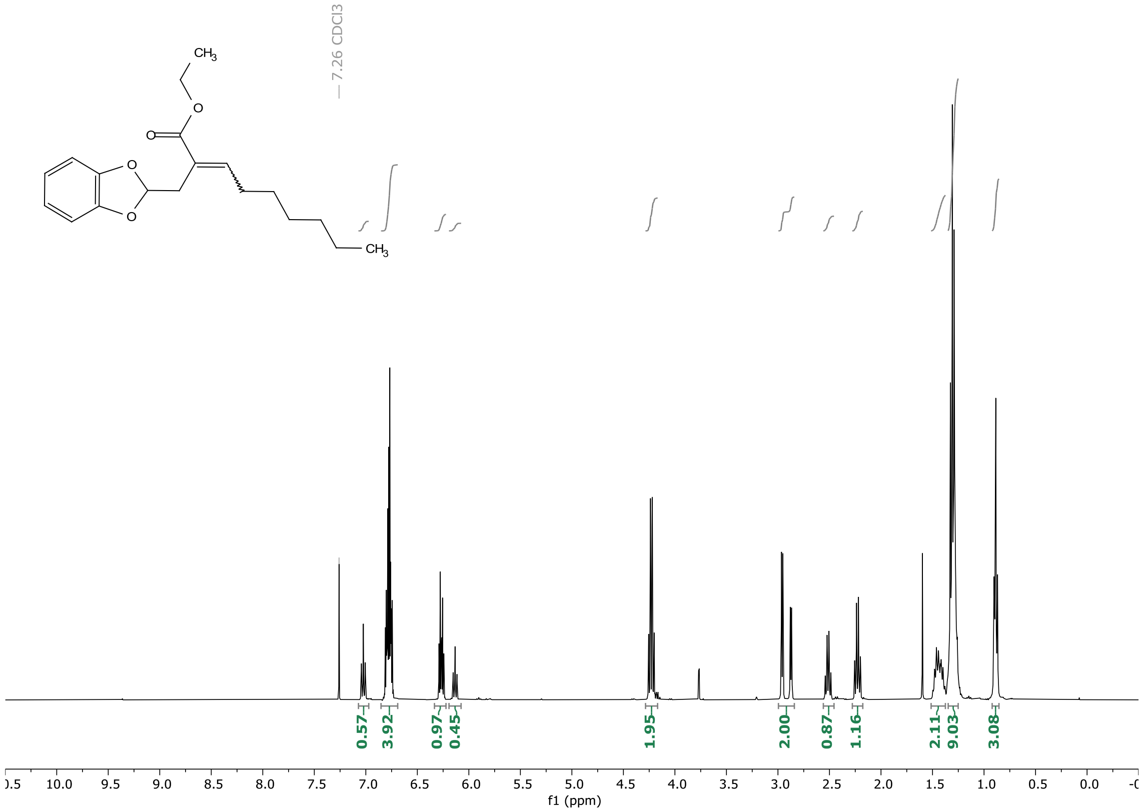

^{13}C NMR (101 MHz, $CDCl_3$) of compound **48**

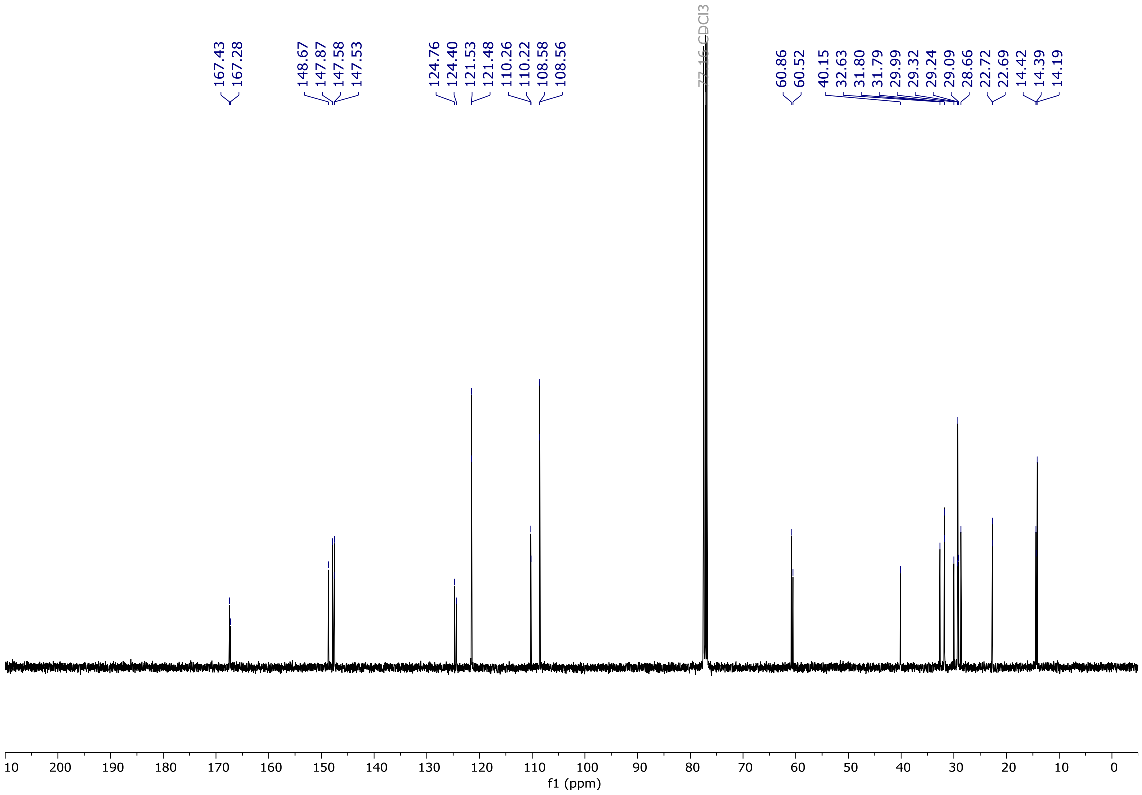

^{1}H NMR (400 MHz, $CDCl_3$) of compound **49**

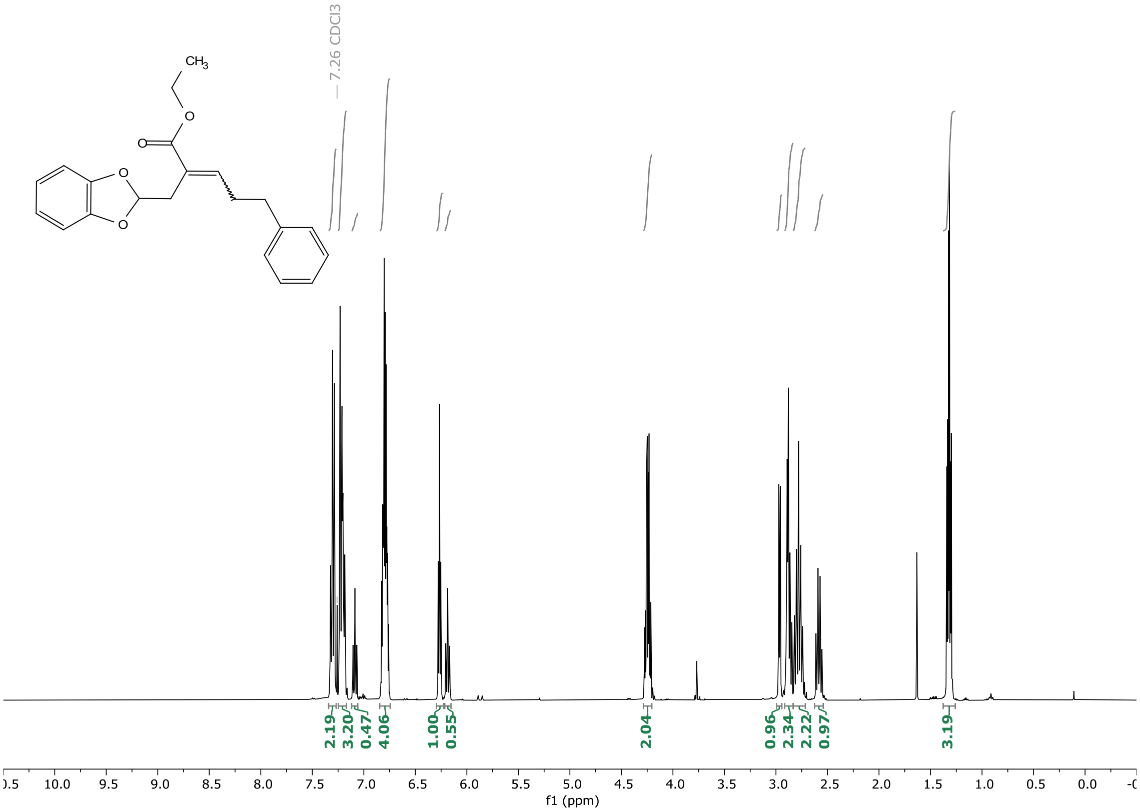

^{13}C NMR (101 MHz, $CDCl_3$) of compound **49**

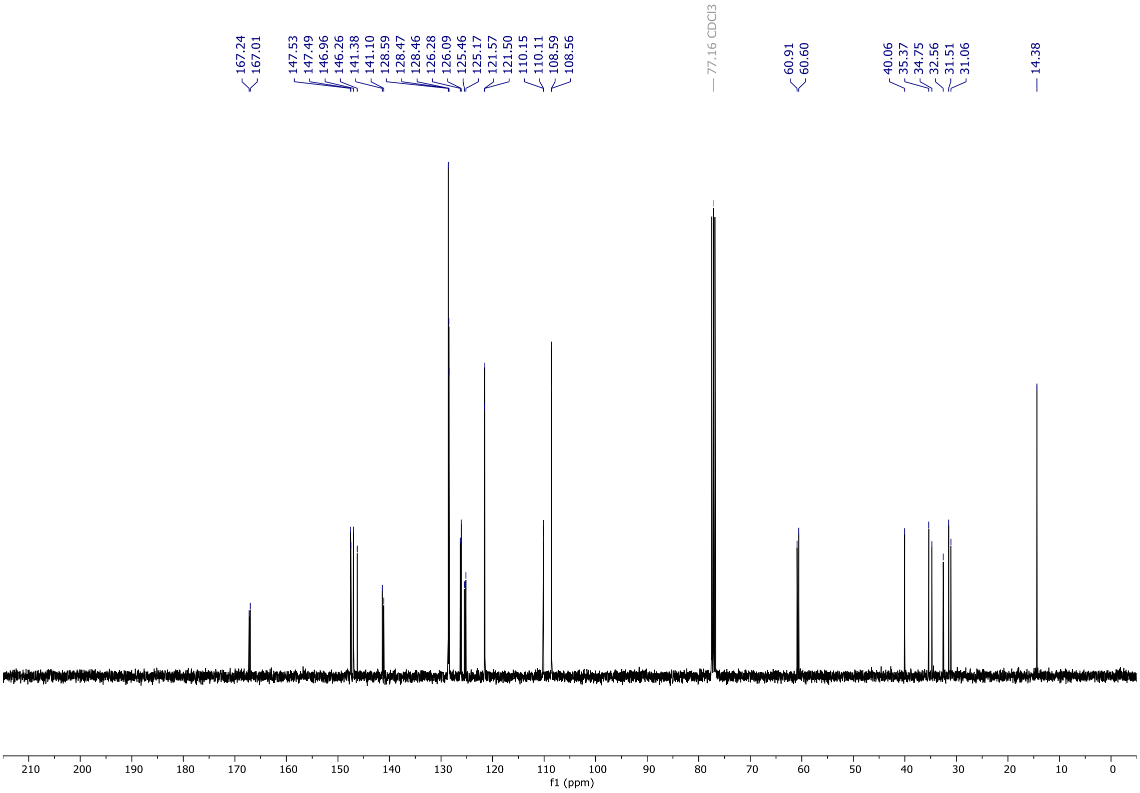

^{1}H NMR (400 MHz, $CDCl_3$) of compound **50**

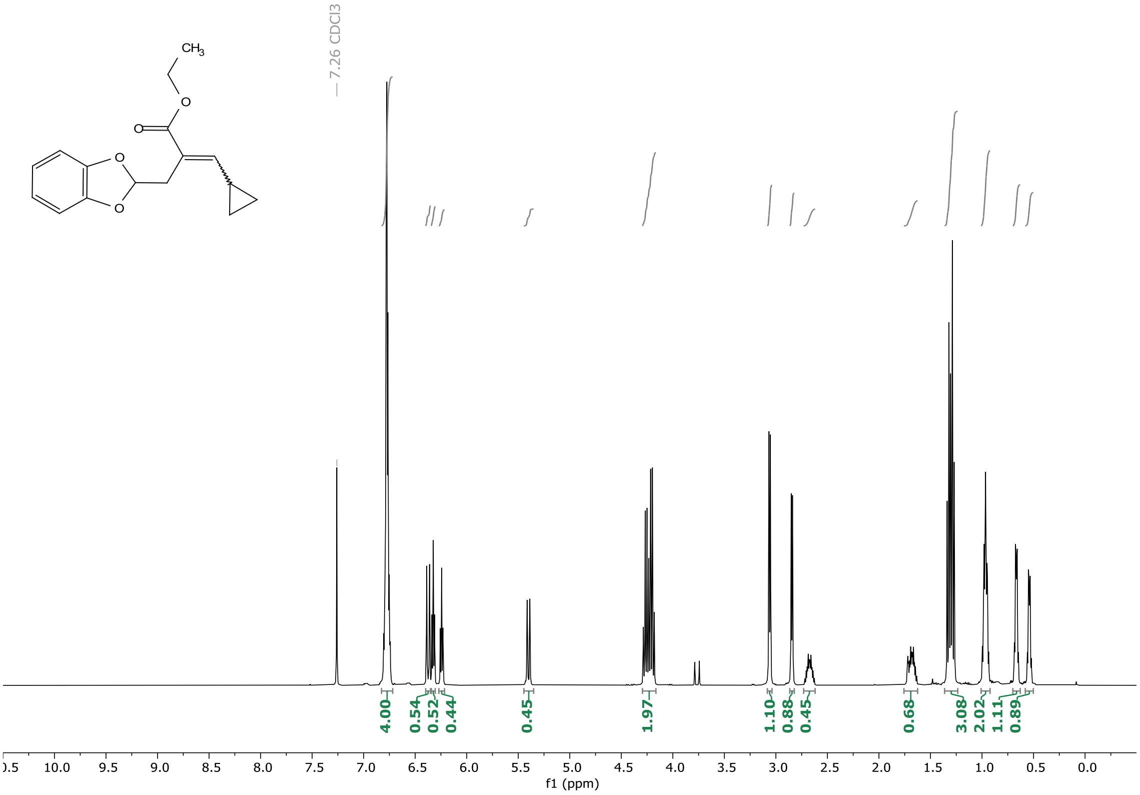

^{13}C NMR (101 MHz, $CDCl_3$) of compound **50**

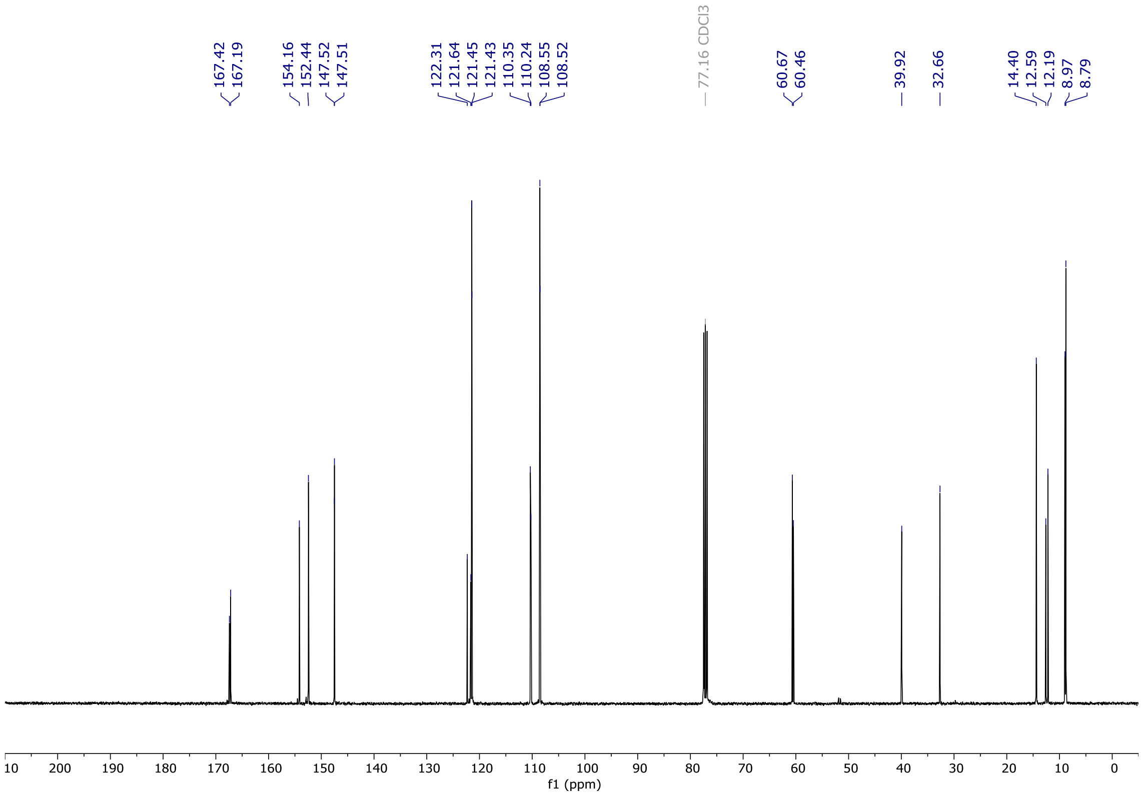

^{1}H NMR (400 MHz, $CDCl_3$) of compound **51**

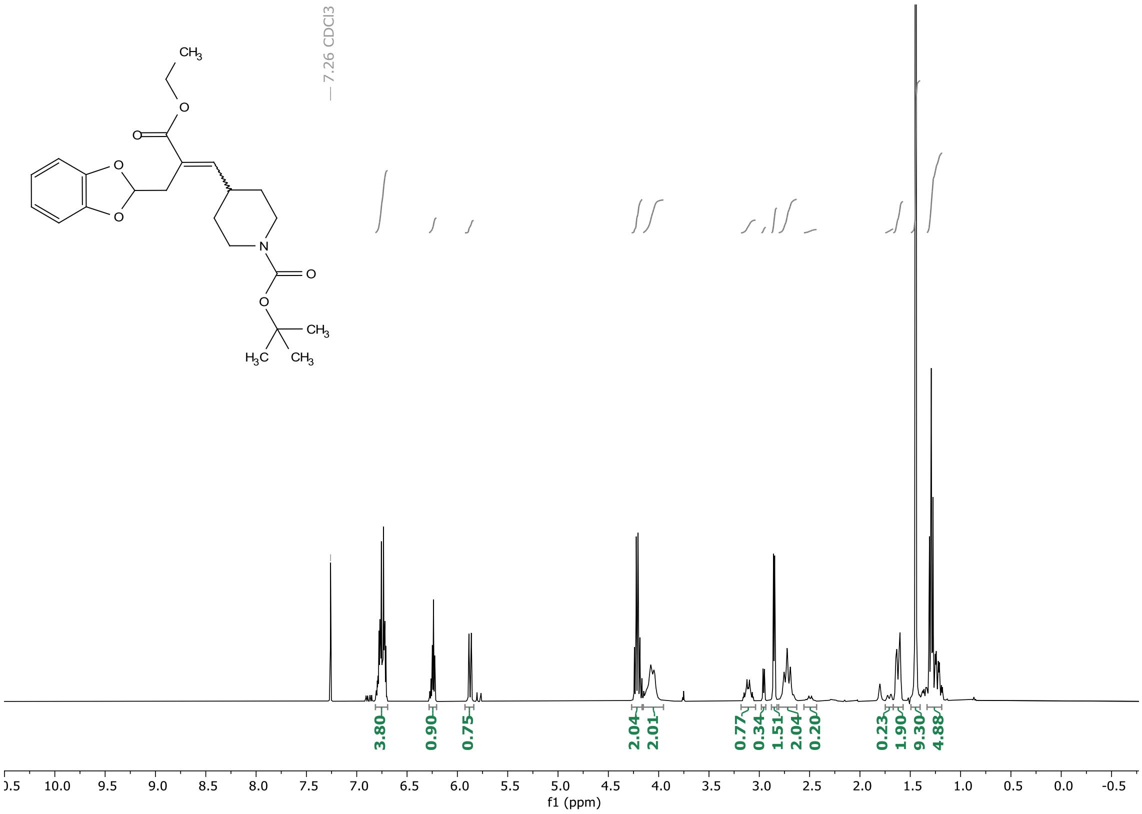

^{13}C NMR (101 MHz, $CDCl_3$) of compound **51**

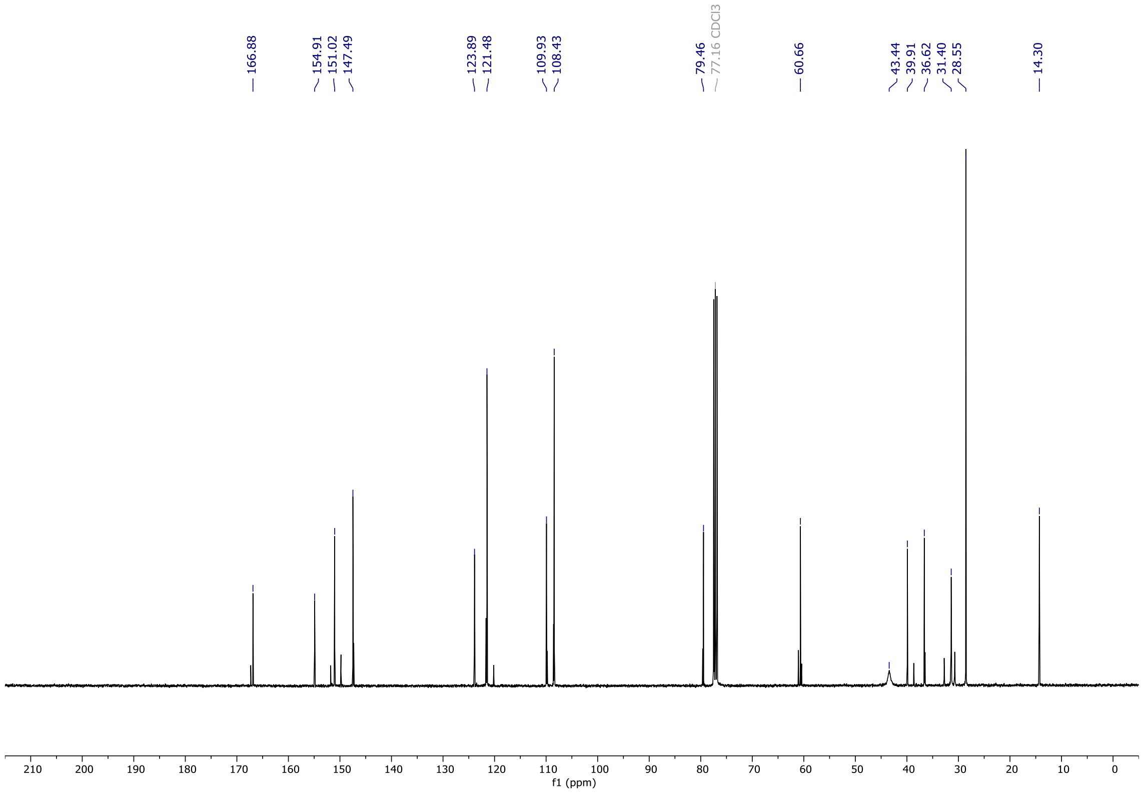

Key HSQC correlations of compound **51**

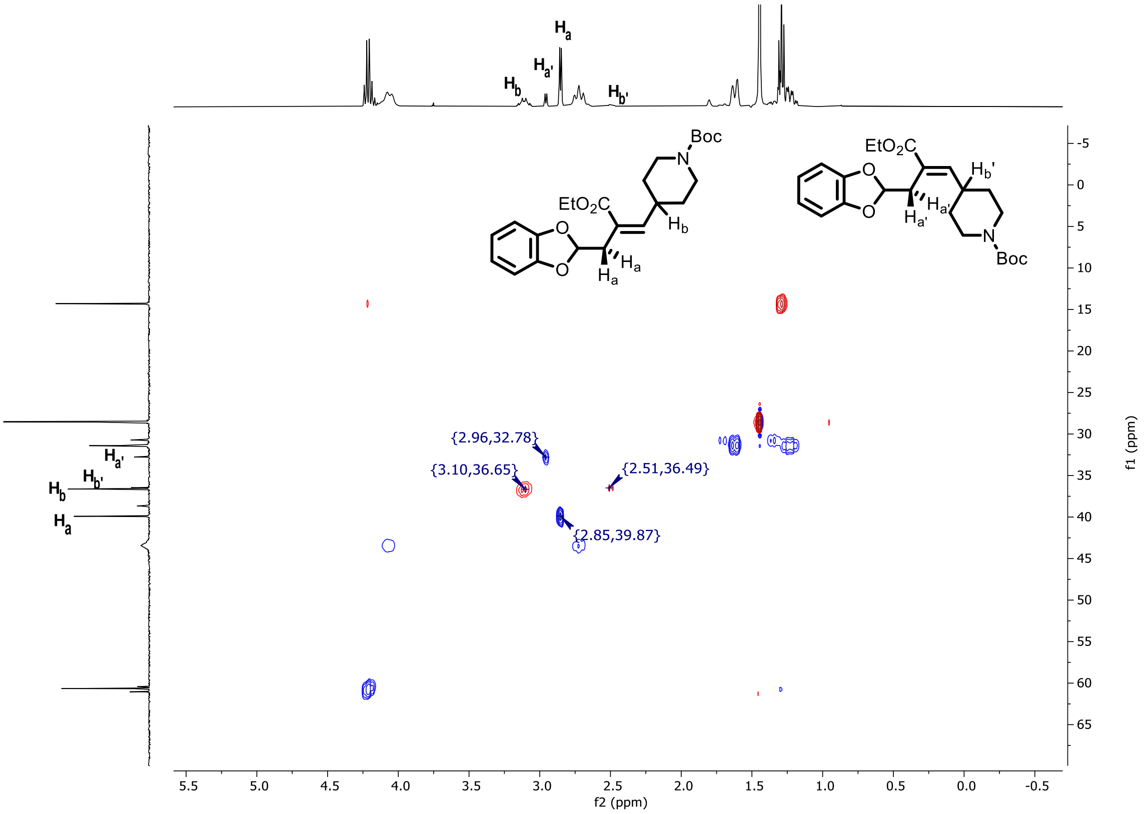

^{1}H–^{1}H NOESY (400 MHz, $CDCl_3$) of compound **51**

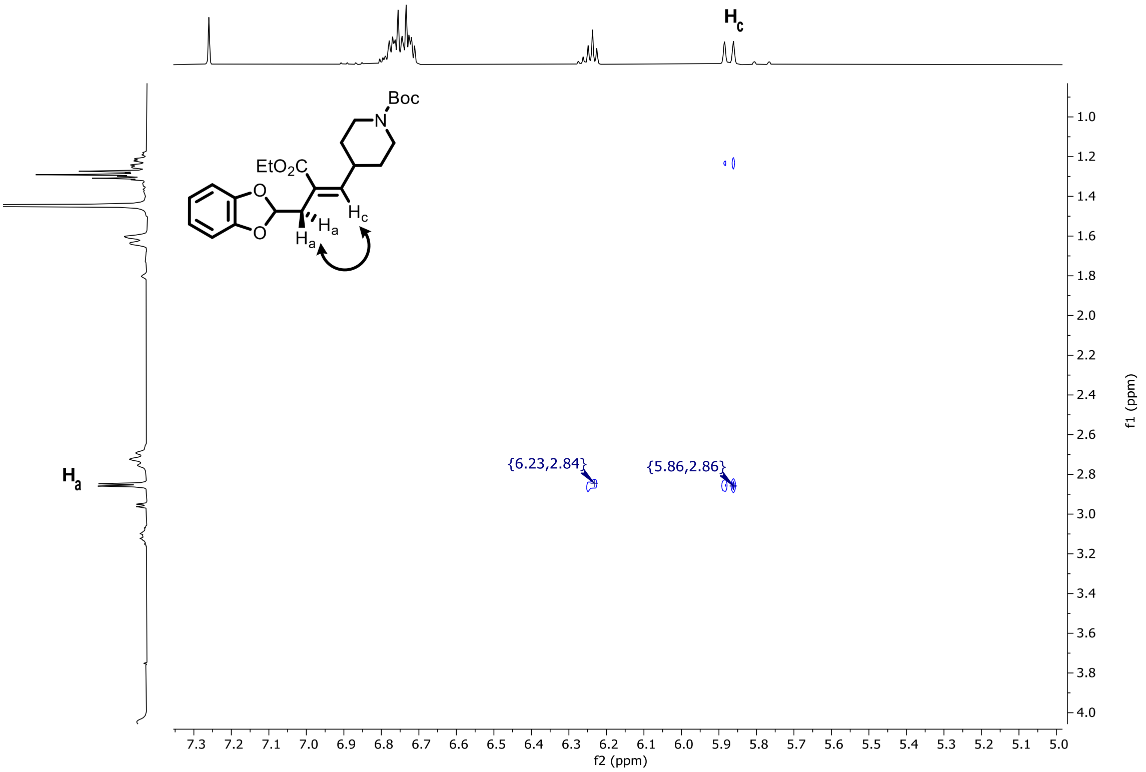

^{1}H NMR (400 MHz, $CDCl_3$) of compound **52**

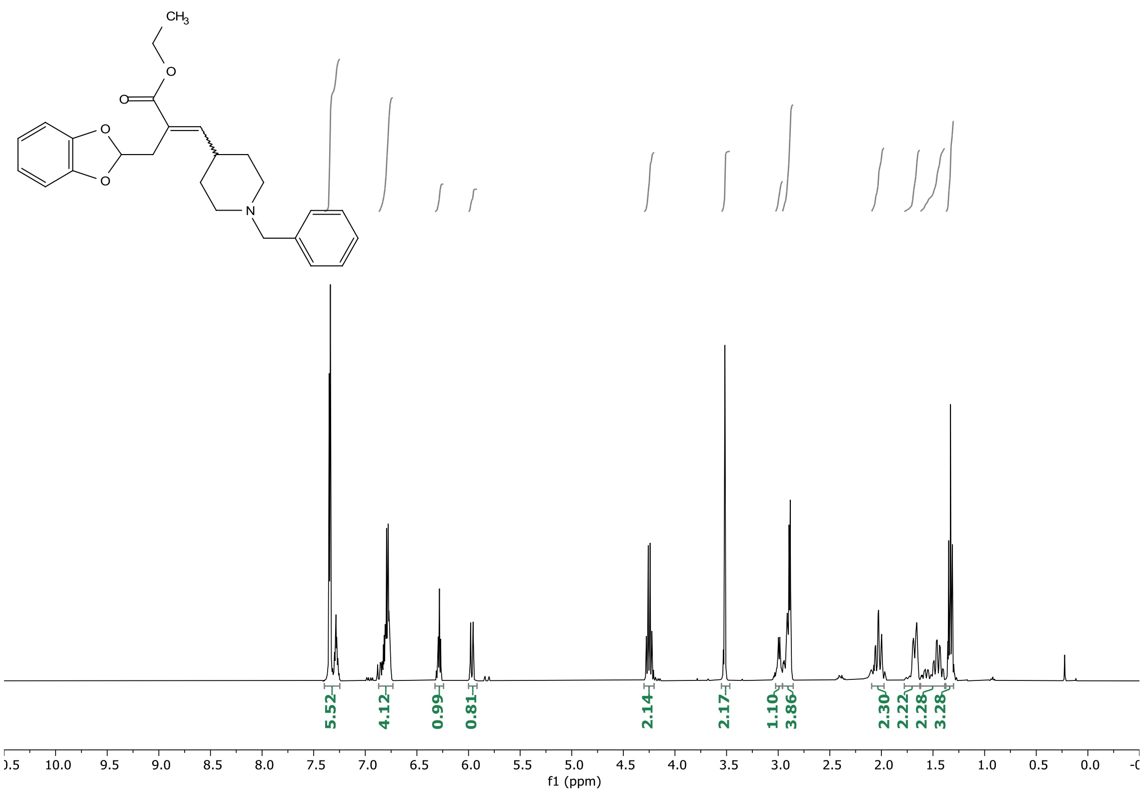

^{13}C NMR (101 MHz, $CDCl_3$) of compound **52**

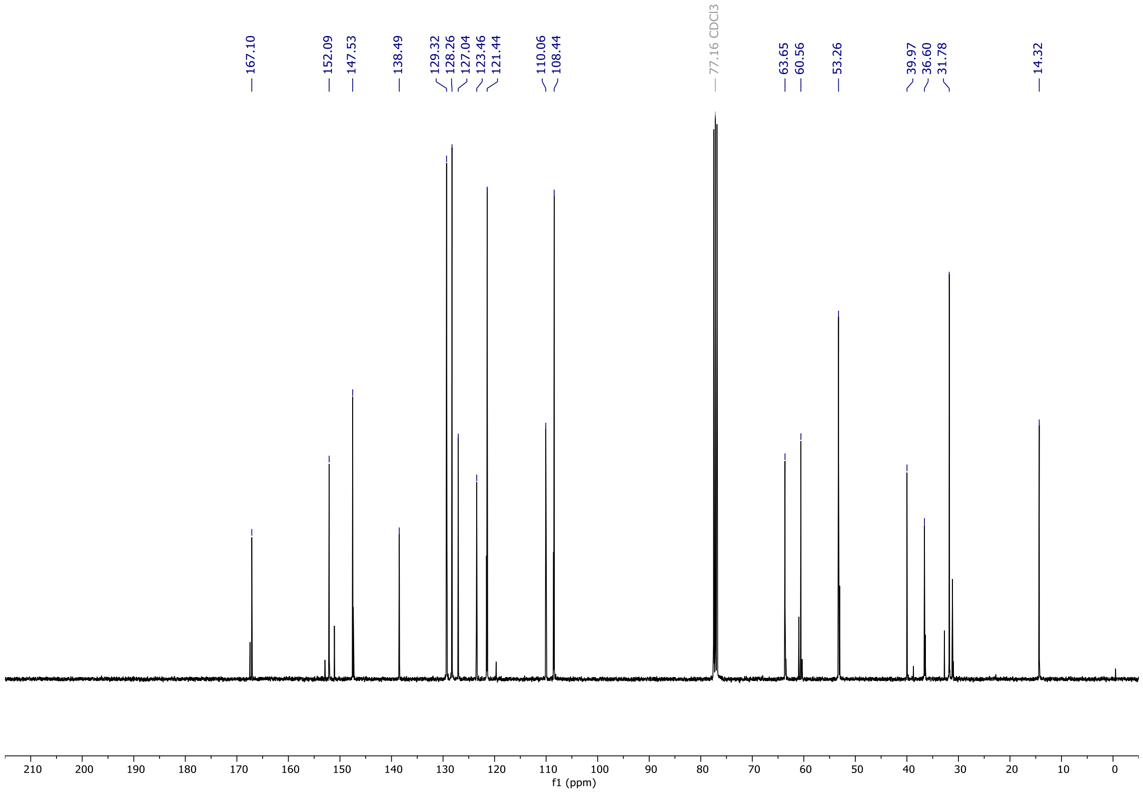

^{1}H–^{1}H NOESY (400 MHz, $CDCl_3$) of compound **52**

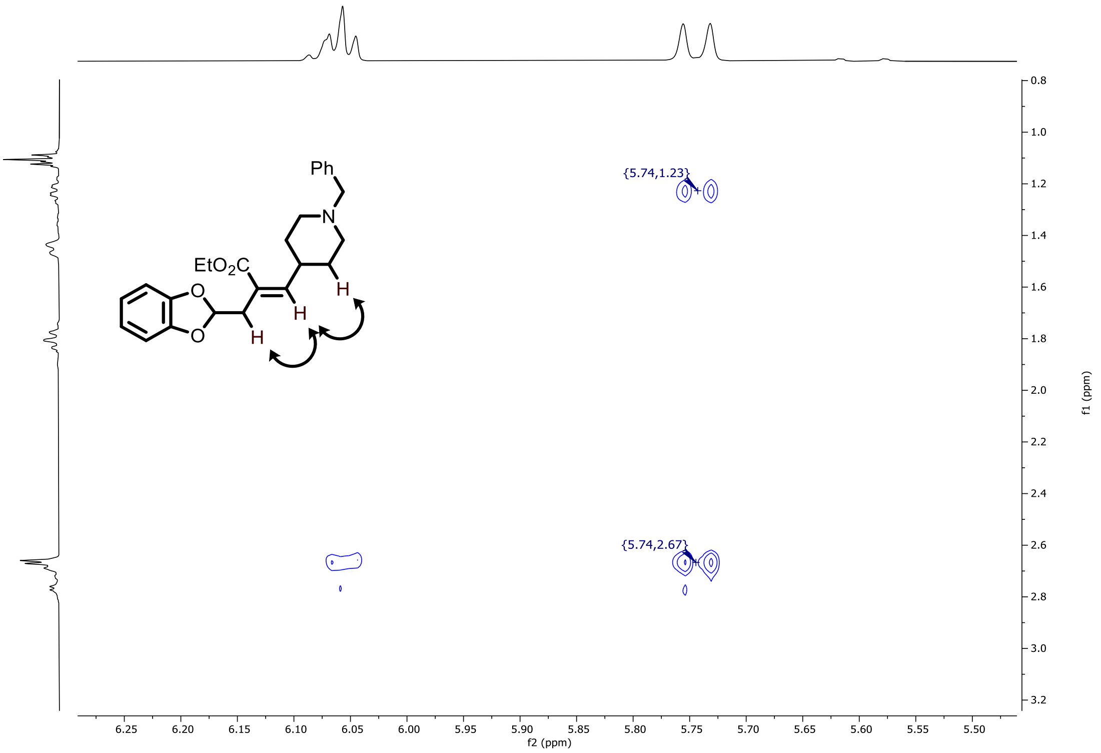

Ph
N
EtO_2C
O
O
H
H
H
{5.74,1.23}
{5.74,2.67}
f1 (ppm)
f2 (ppm)

^{1}H NMR (400 MHz, $CDCl_3$) of compound **53**

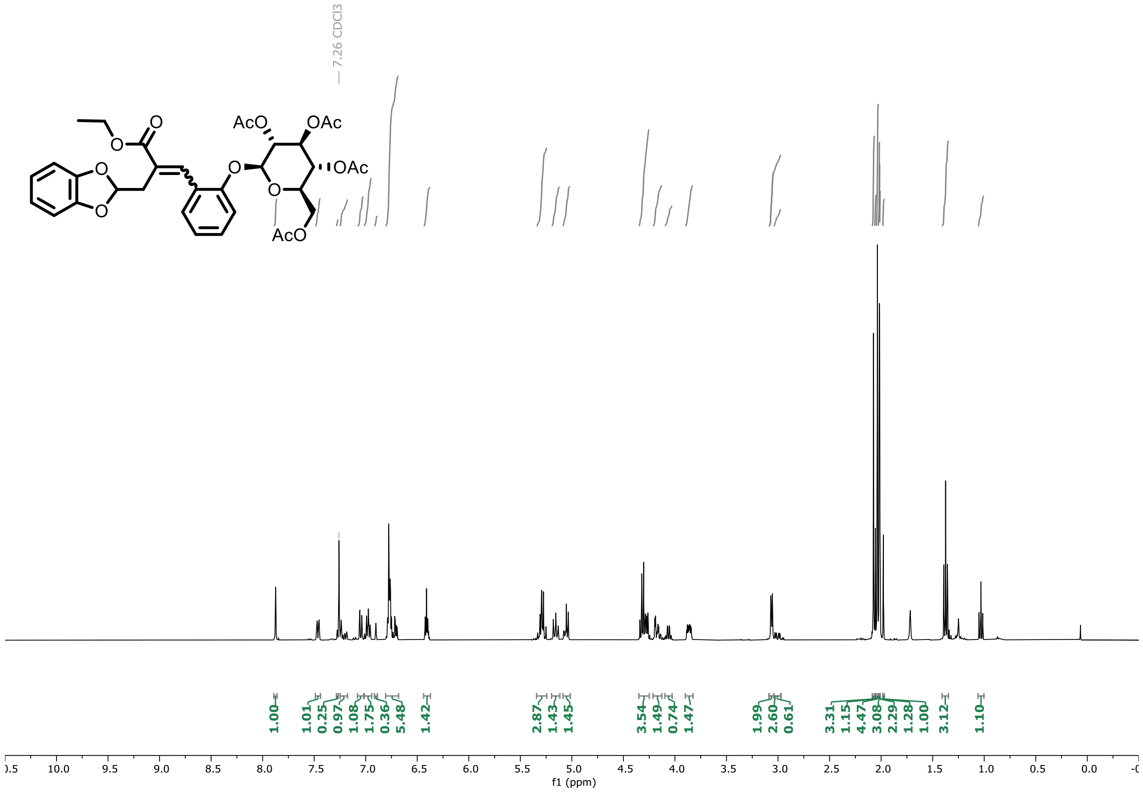

^{13}C NMR (101 MHz, $CDCl_3$) of compound **53**

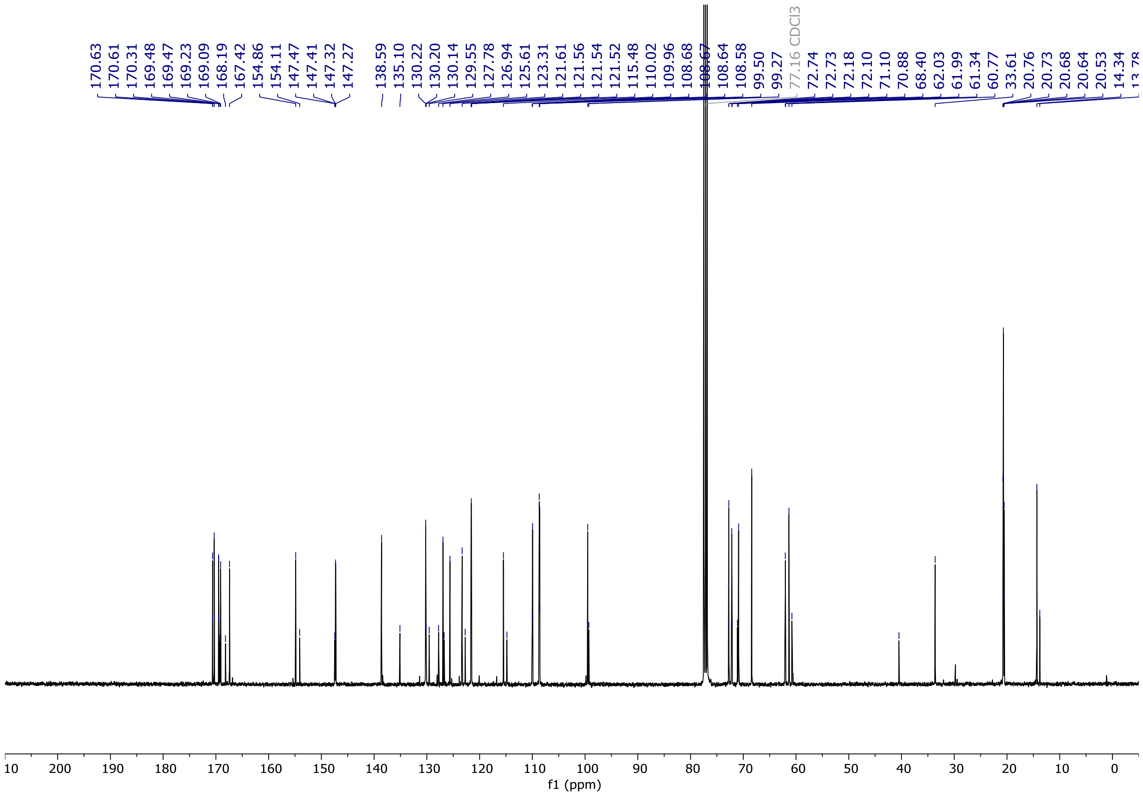

Key COSY (400 MHz, $CDCl_3$) correlations of compound **53**

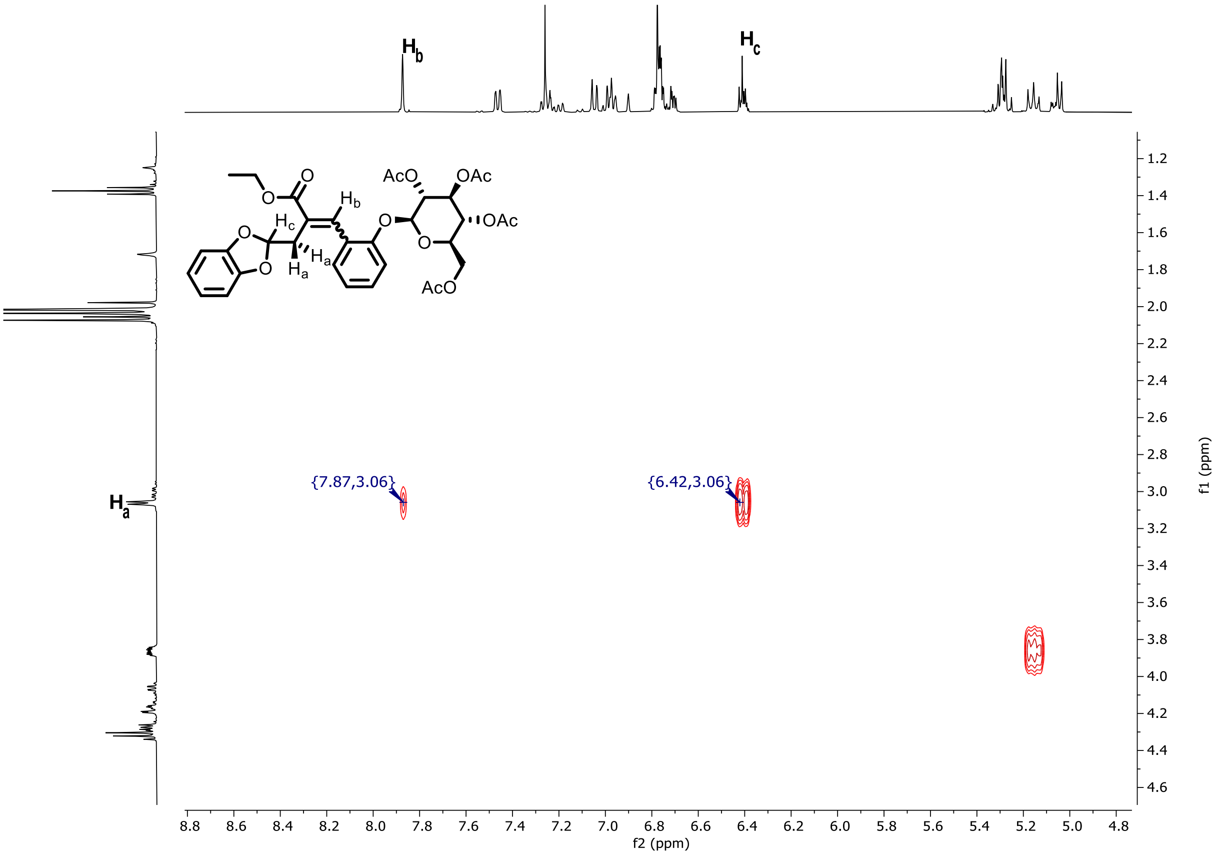

HSQC of compound **53**

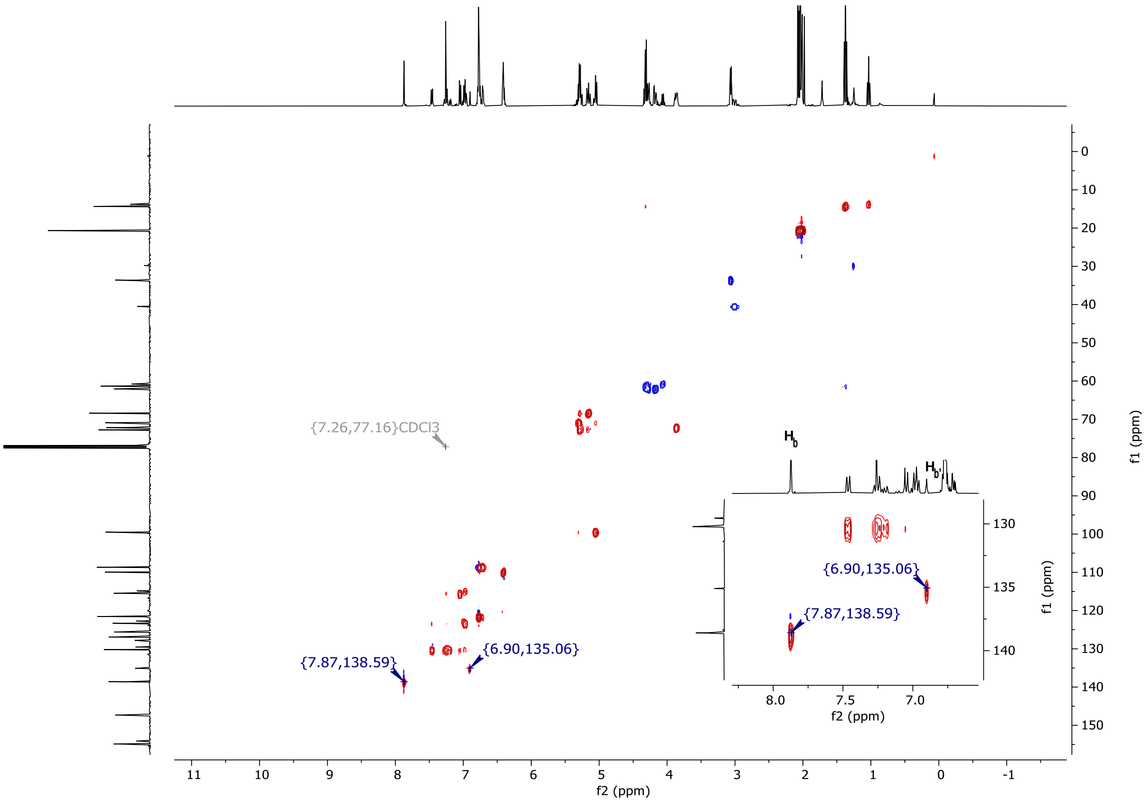

^{1}H–^{1}H NOESY (400 MHz, CDCl$_3$) of compound **53**

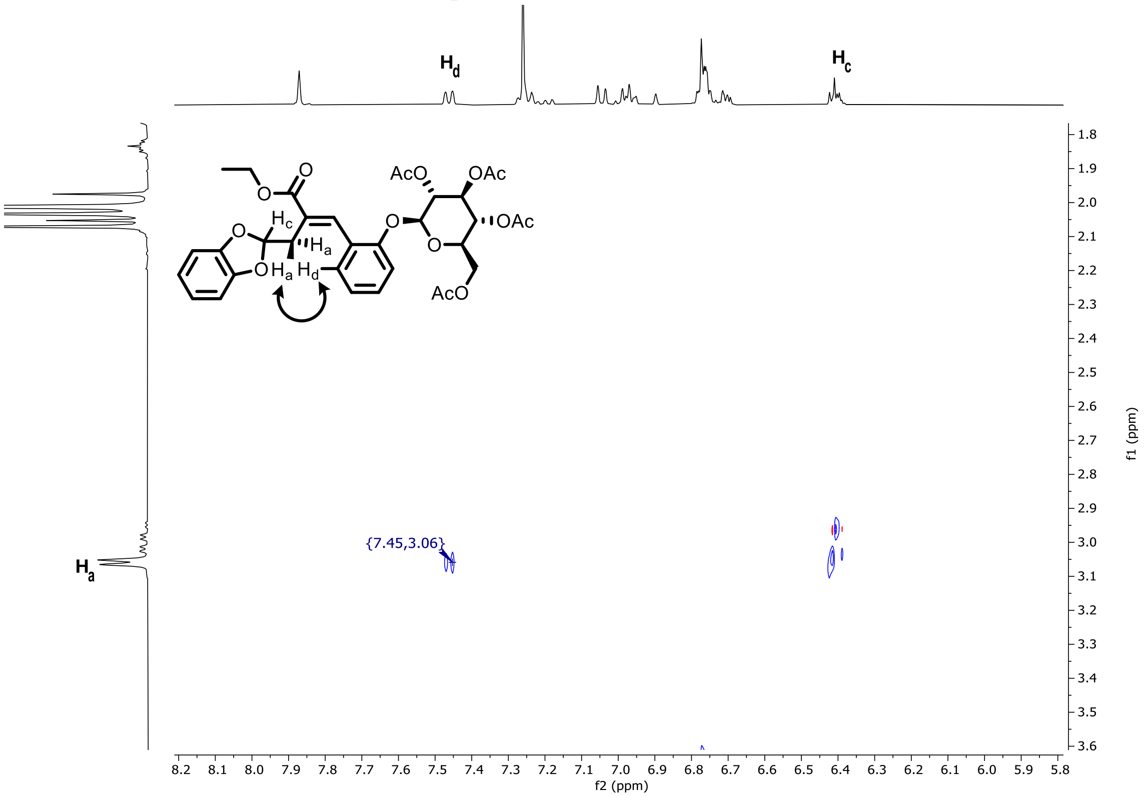

^{1}H NMR (400 MHz, $CDCl_3$) of compound **54**

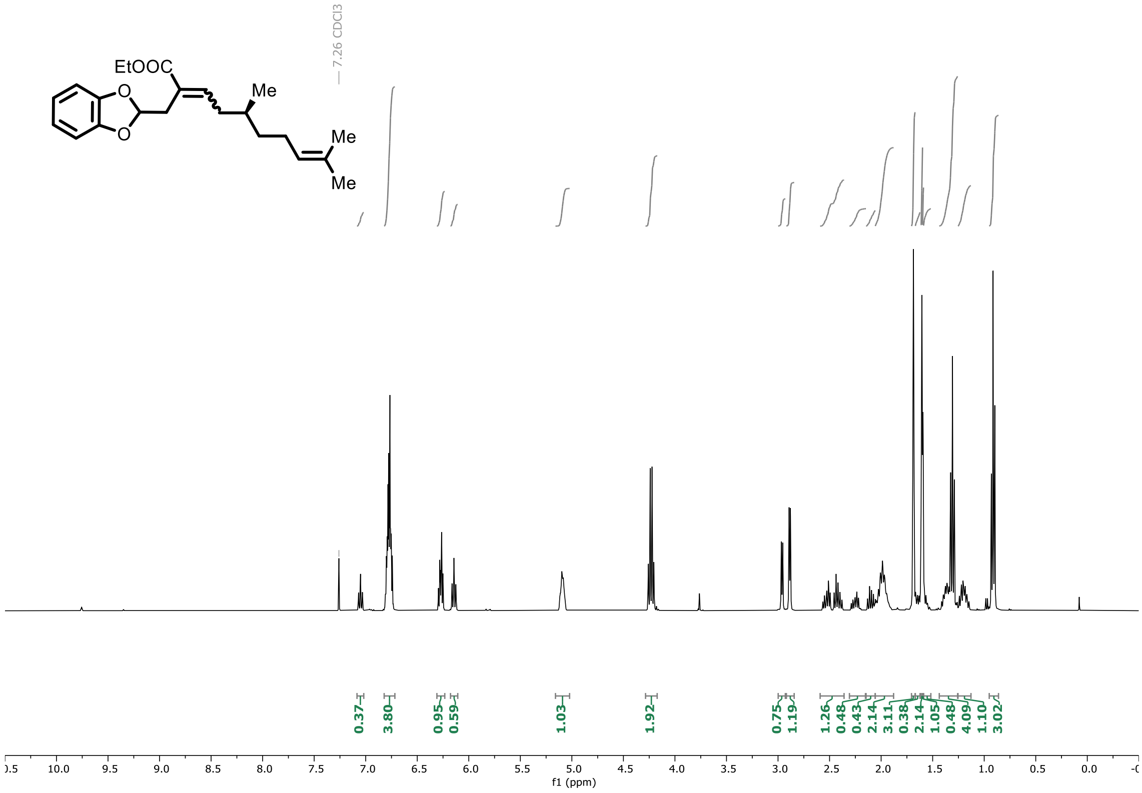

^{13}C NMR (101 MHz, $CDCl_3$) of compound **54**

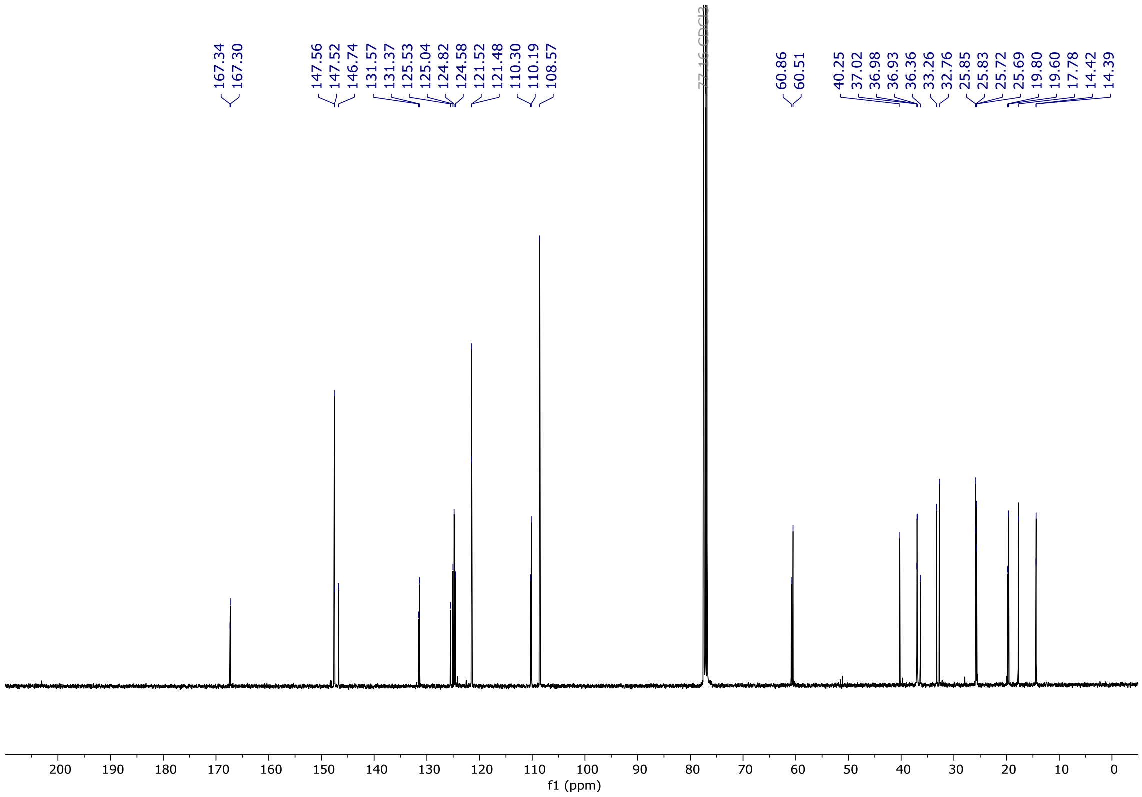

Key COSY (400 MHz, $CDCl_3$) correlations of compound **54**

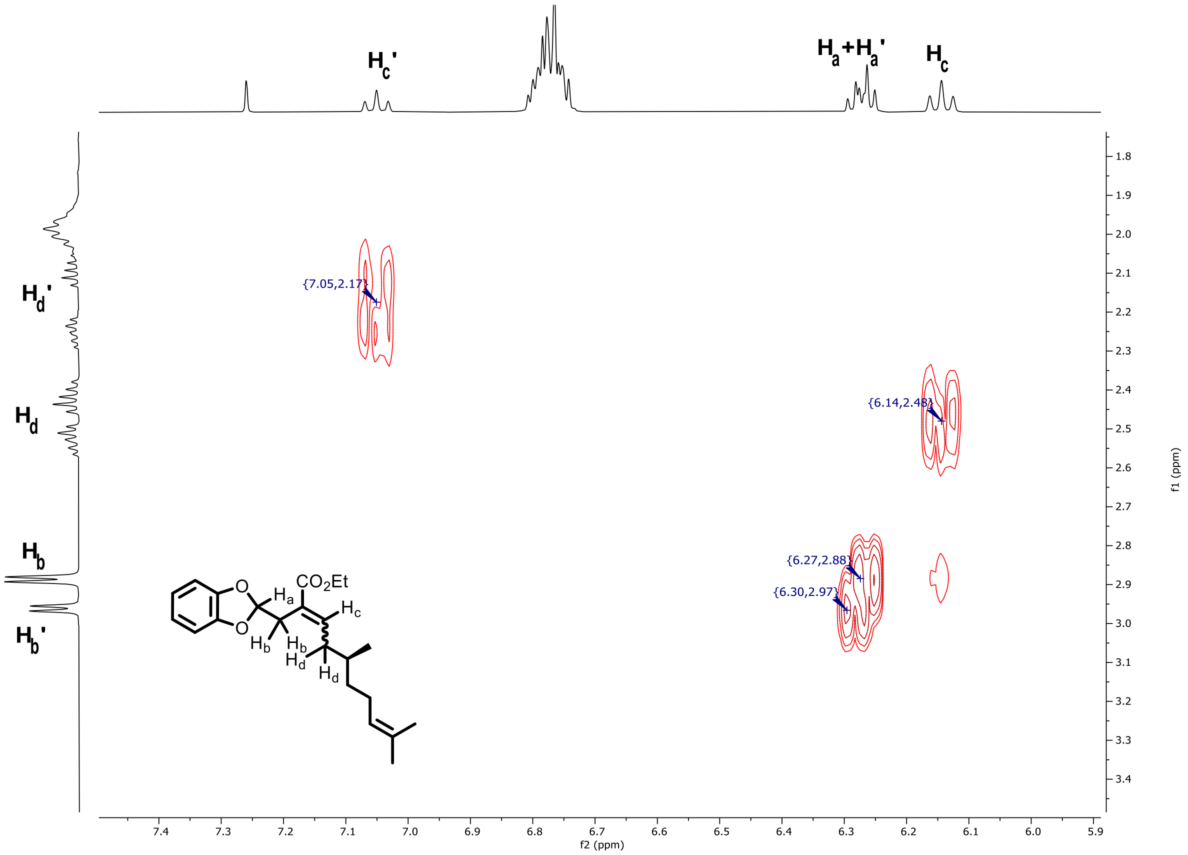

HSQC of compound **54**

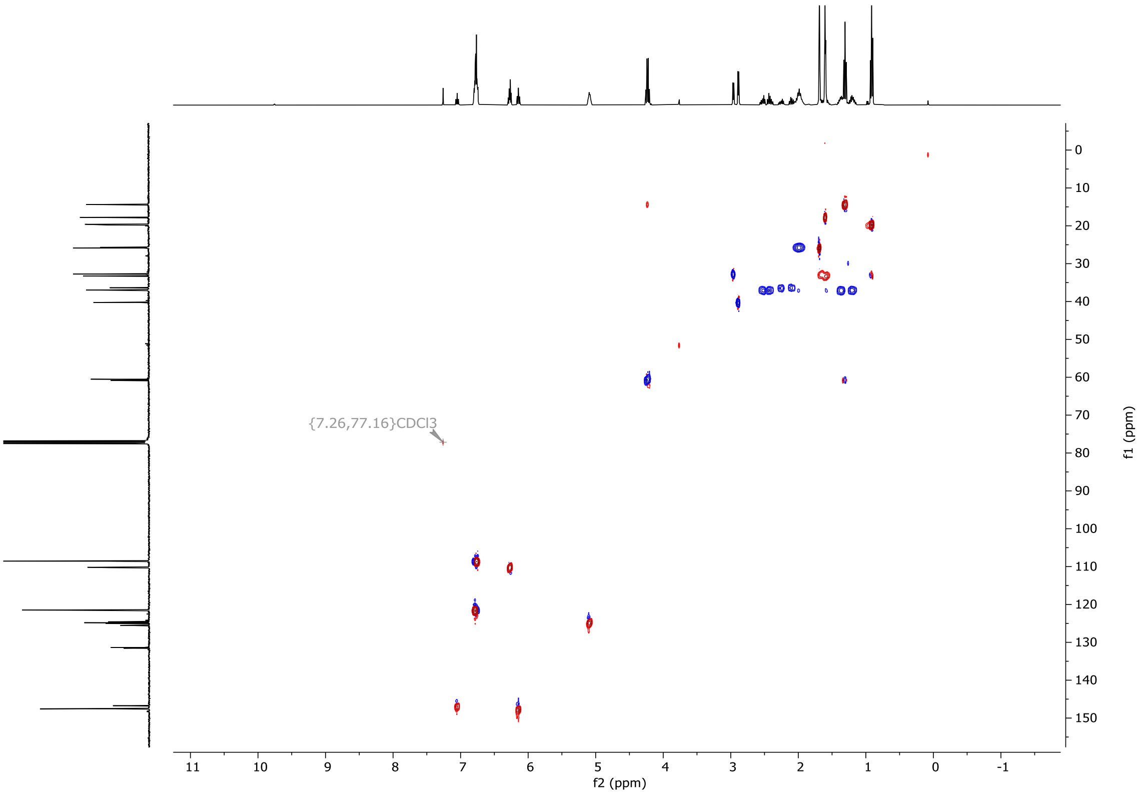

^{1}H NMR (400 MHz, $CDCl_3$) of compound **55**

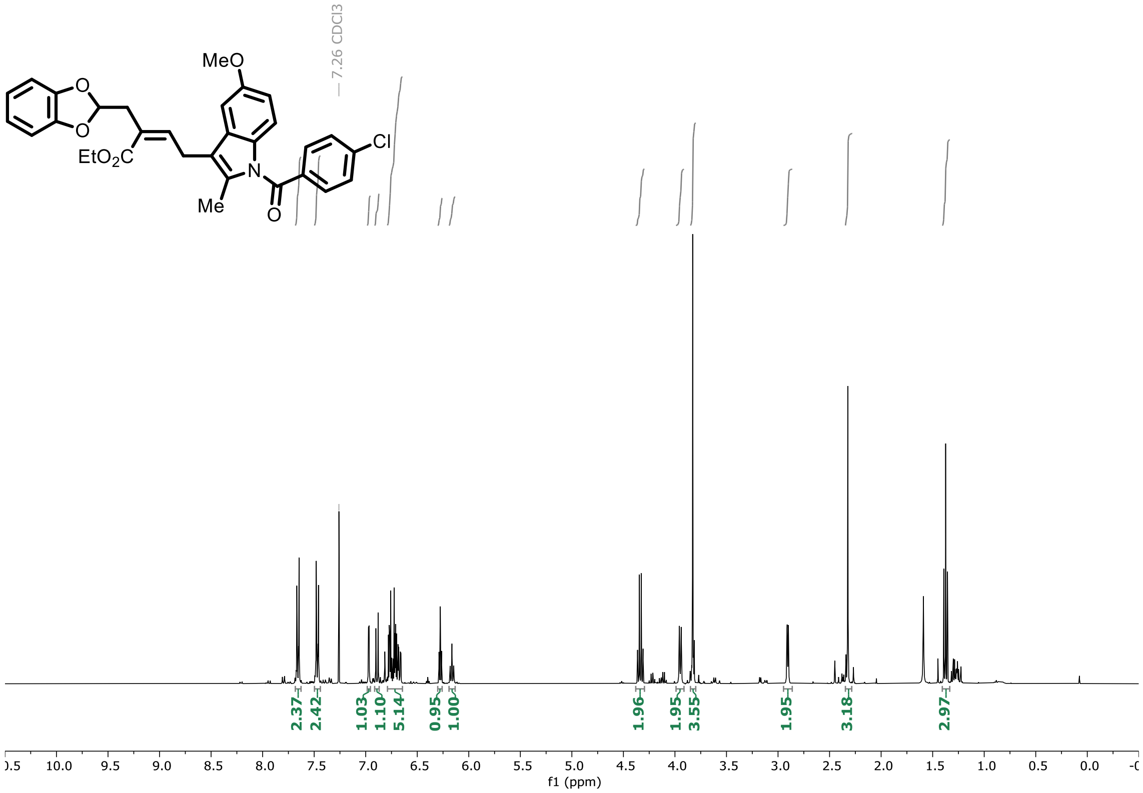

^{13}C NMR (101 MHz, $CDCl_3$) of compound **55**

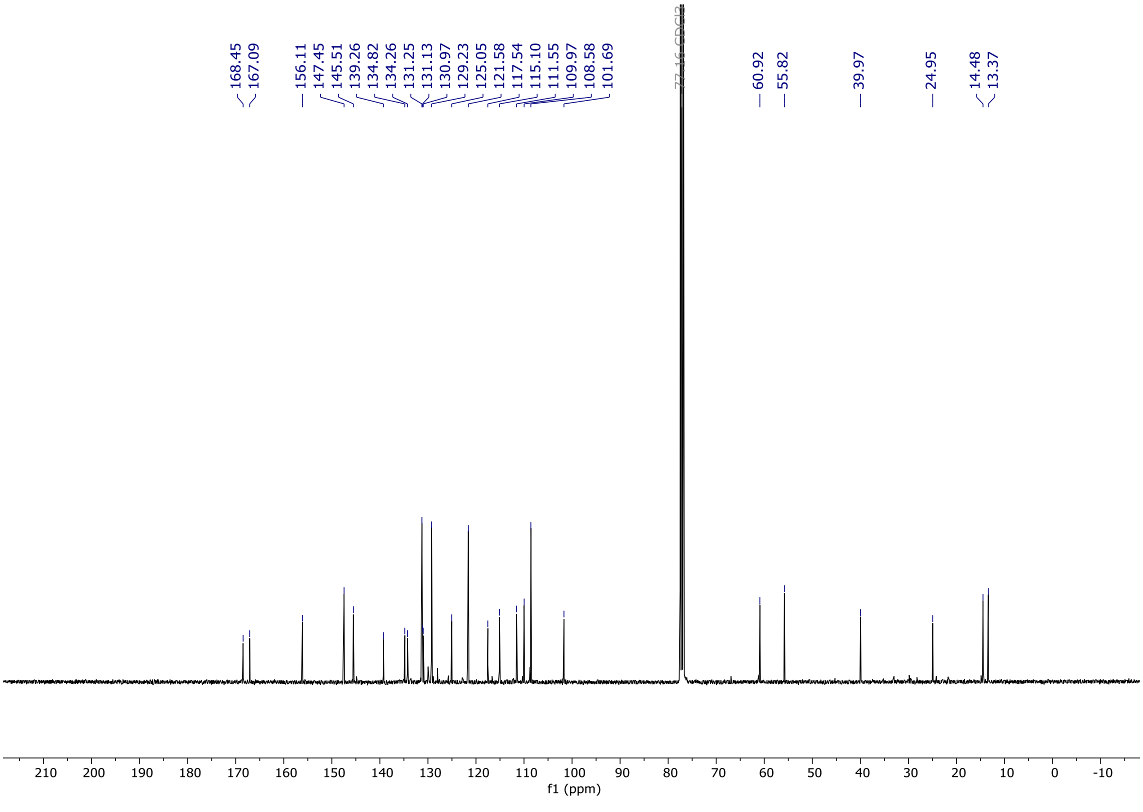

COSY (400 MHz, $CDCl_3$) of compound **55**

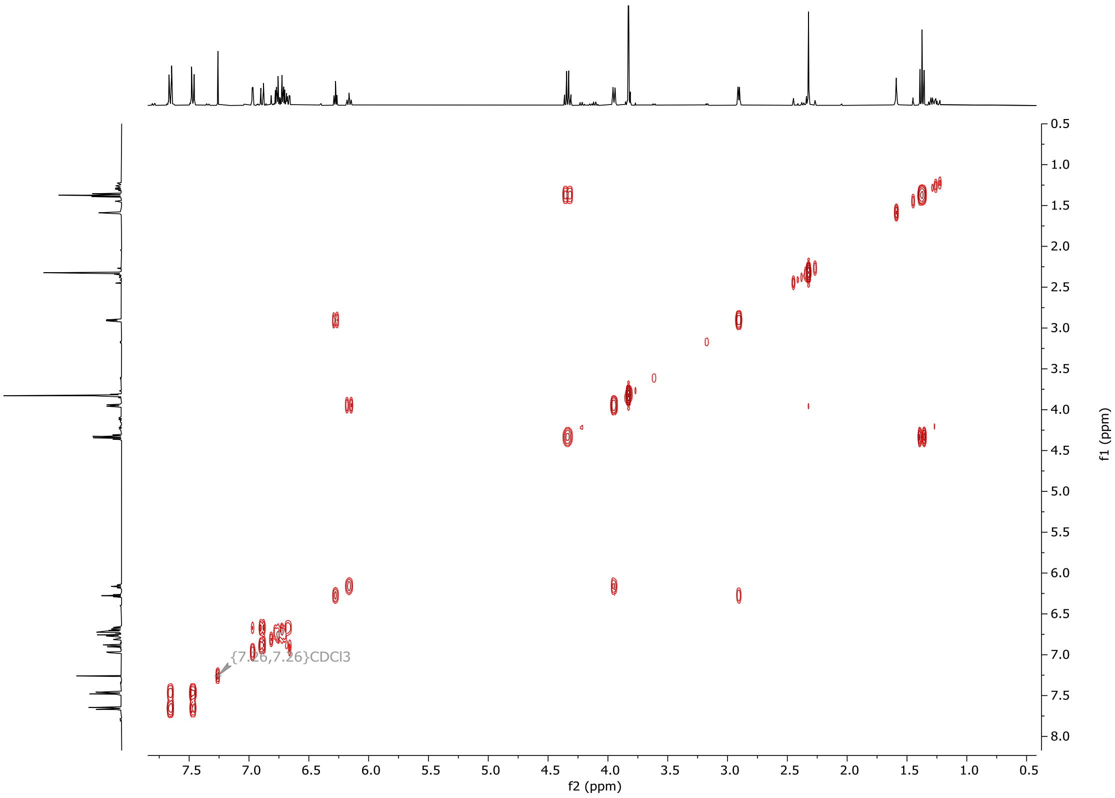

HSQC of compound **55**

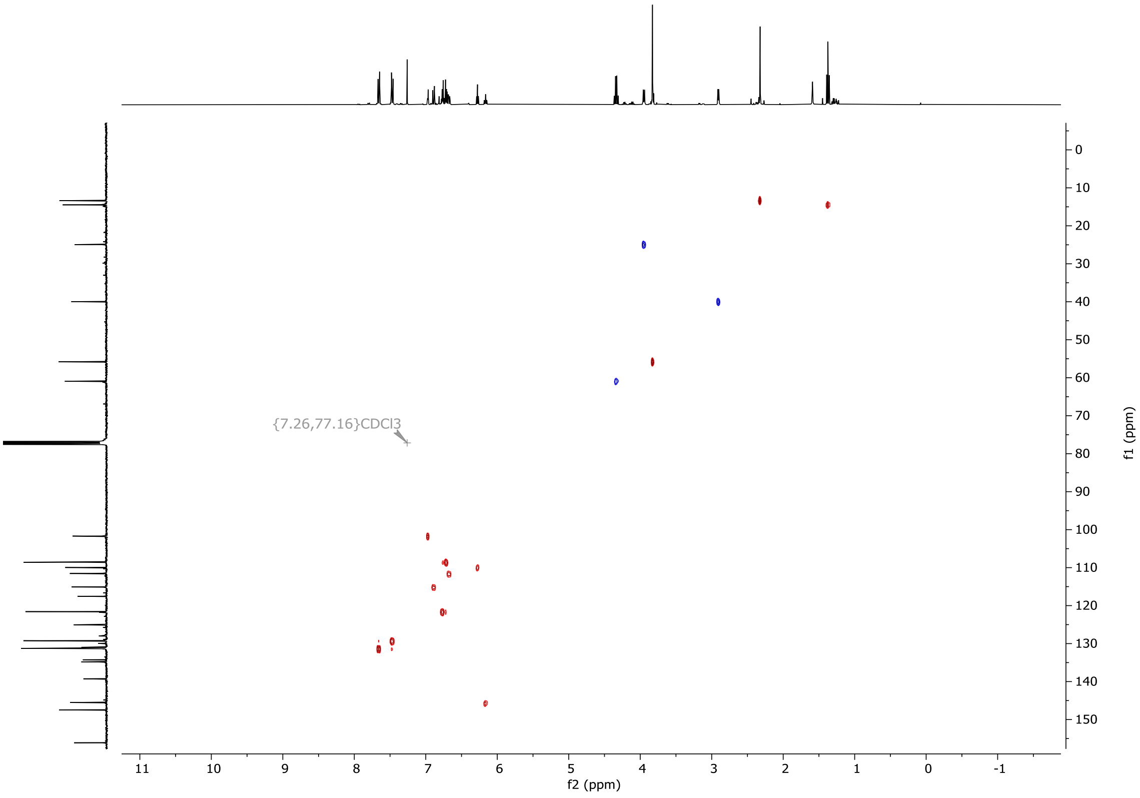

^{1}H–^{1}H NOESY (300 MHz, $CDCl_3$) of compound **55**

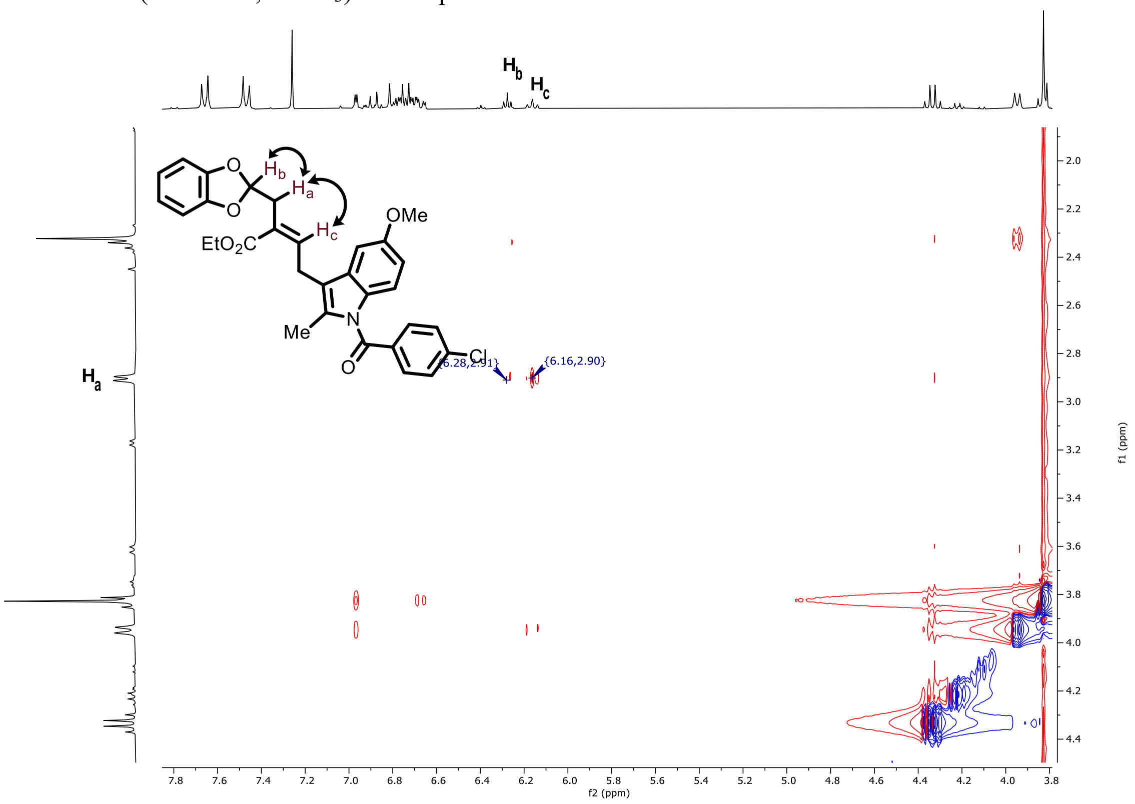

16. NMR Spectra of compounds 56, 59-65

^{1}H NMR (400 MHz, $CDCl_3$) of compound **56**

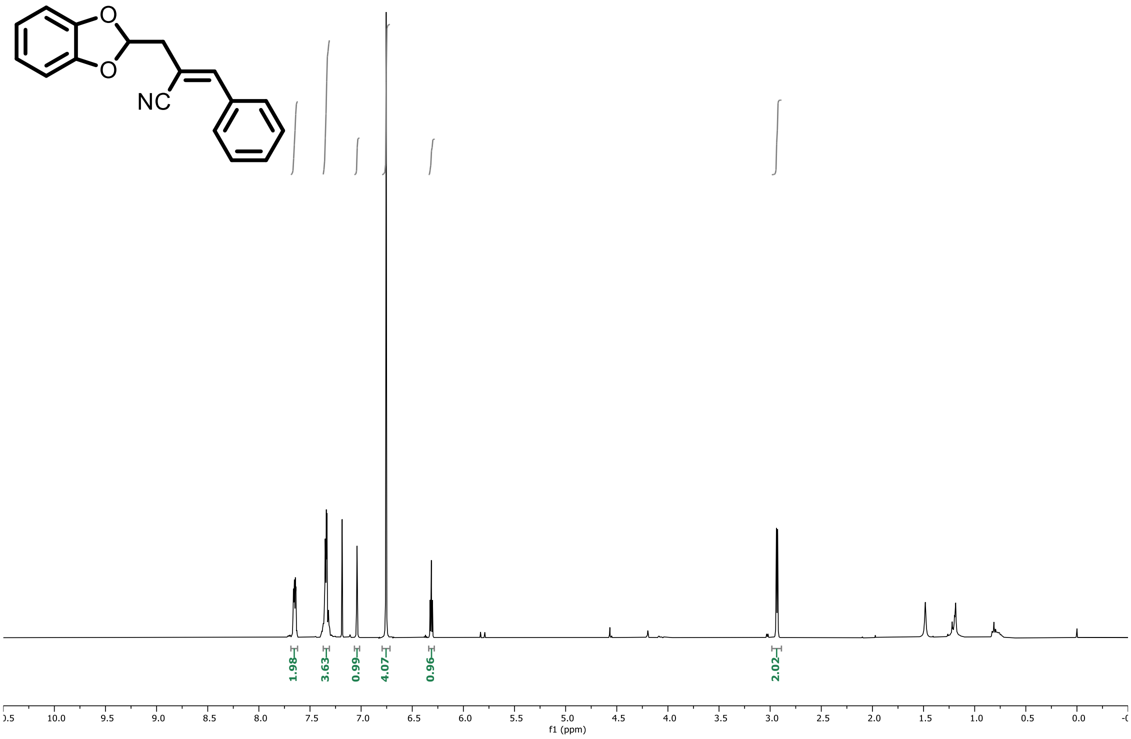

^{13}C NMR (101 MHz, $CDCl_3$) of compound **56**

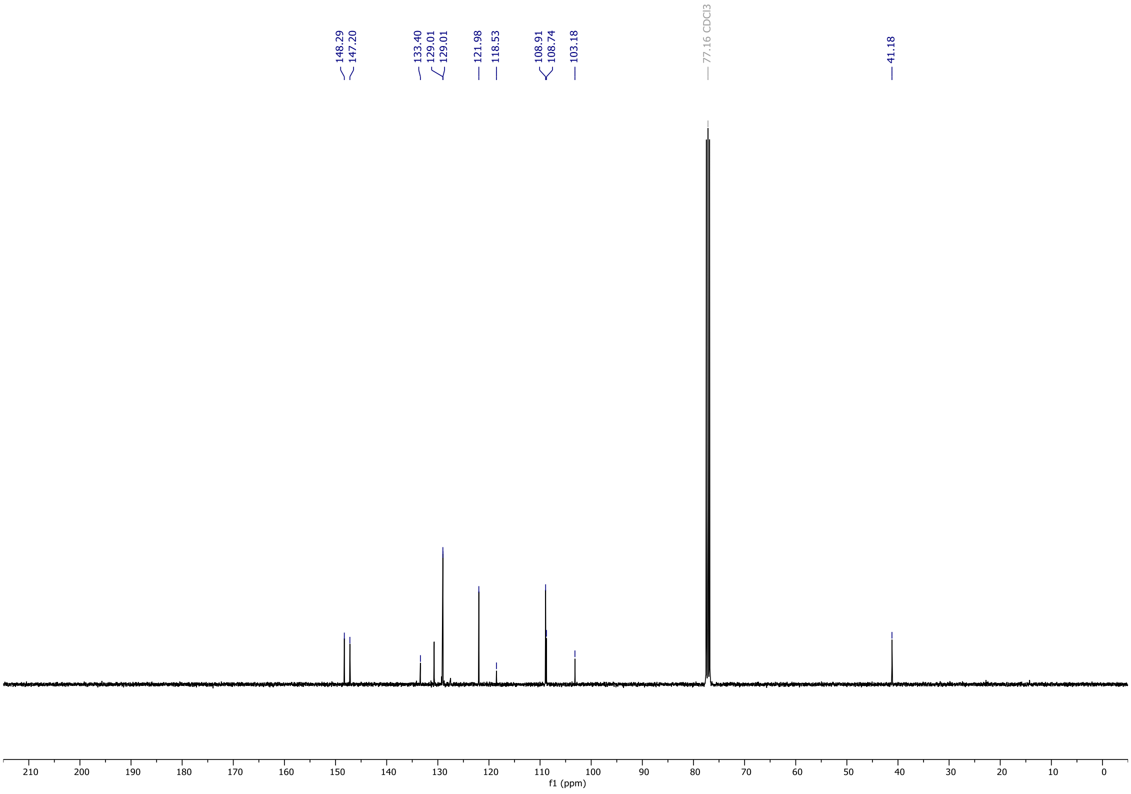

COSY (400 MHz, $CDCl_3$) of compound **56**

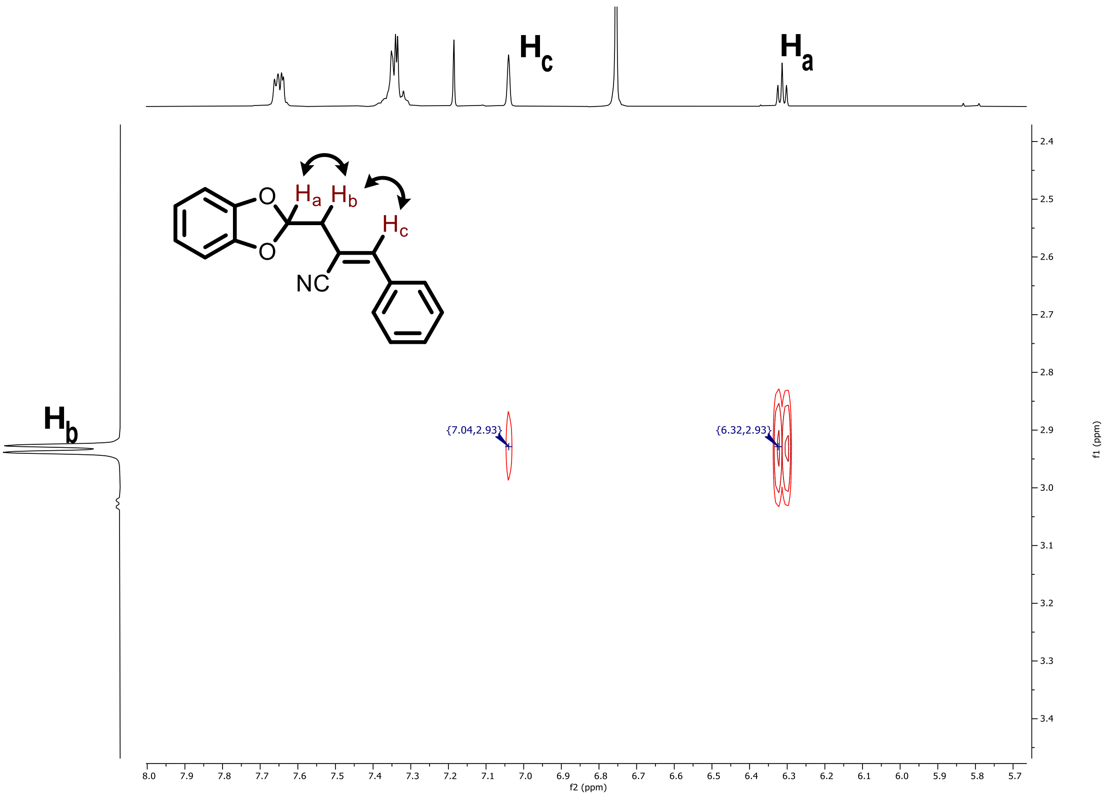

^{1}H–^{1}H NOESY (400 MHz, $CDCl_3$) of compound **56**

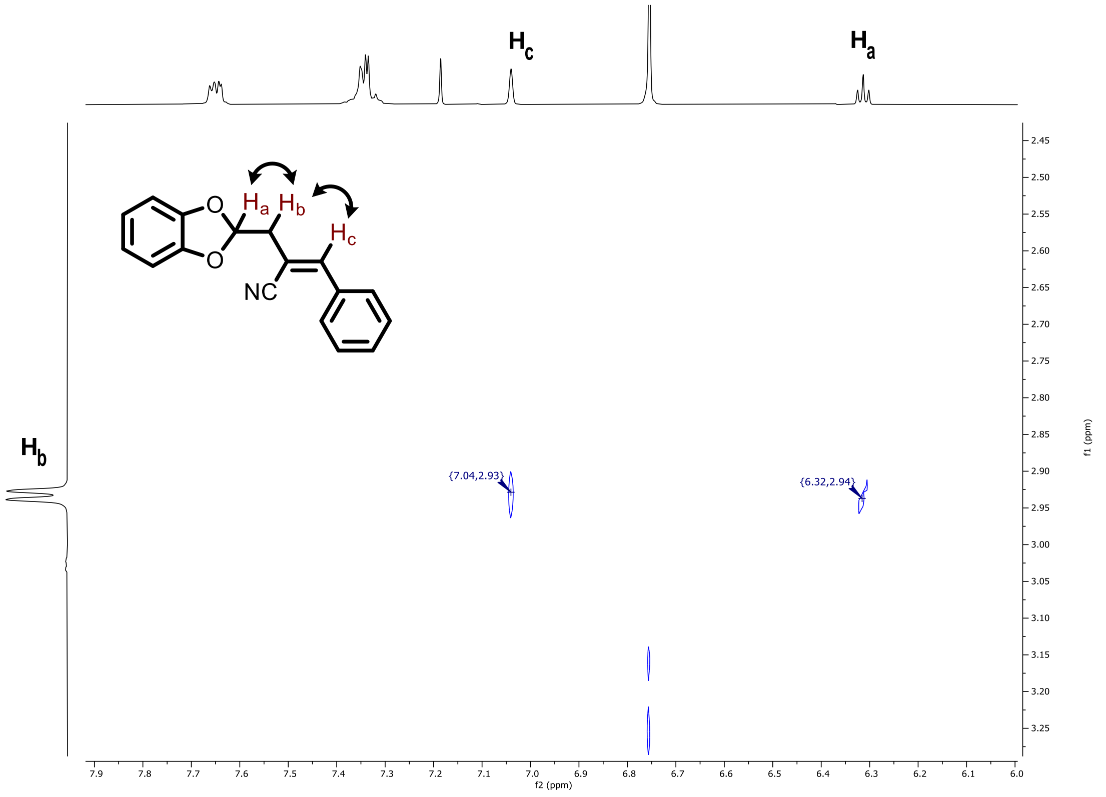

^{1}H NMR (400 MHz, $CDCl_3$) of compound **59**

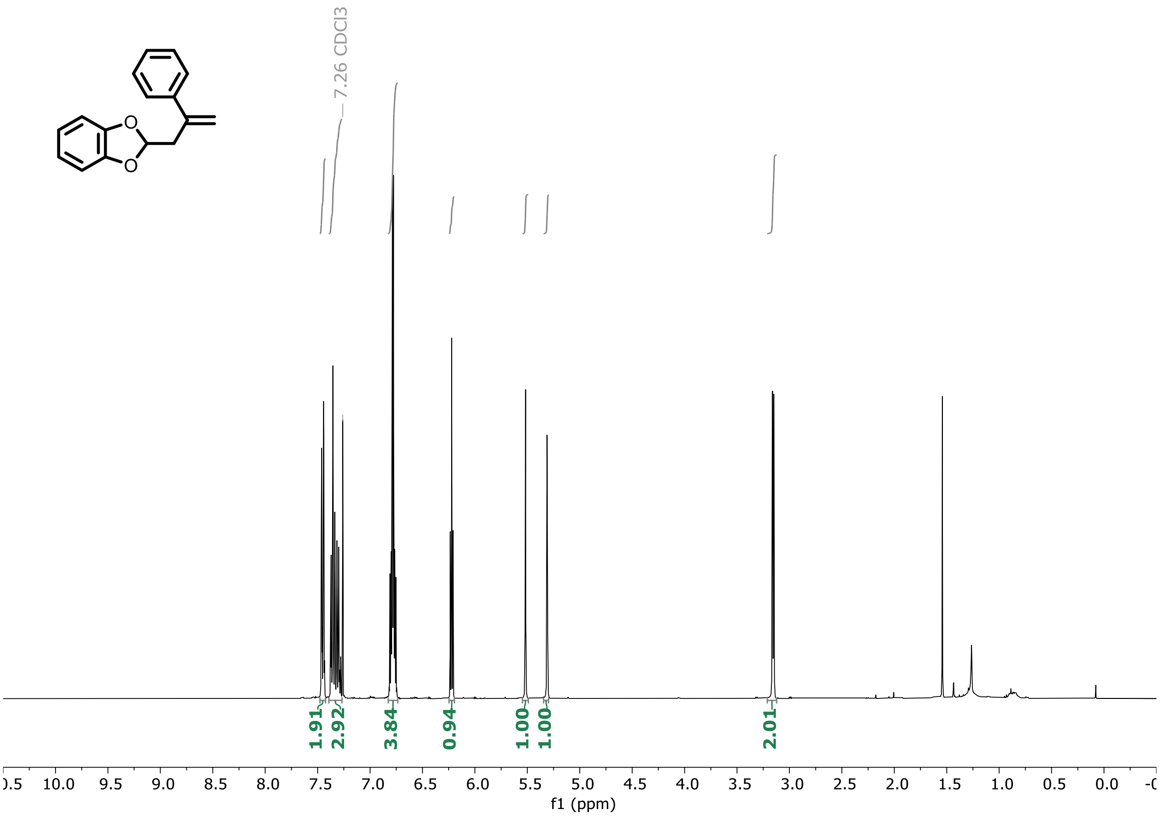

^{13}C NMR (101 MHz, $CDCl_3$) of compound **59**

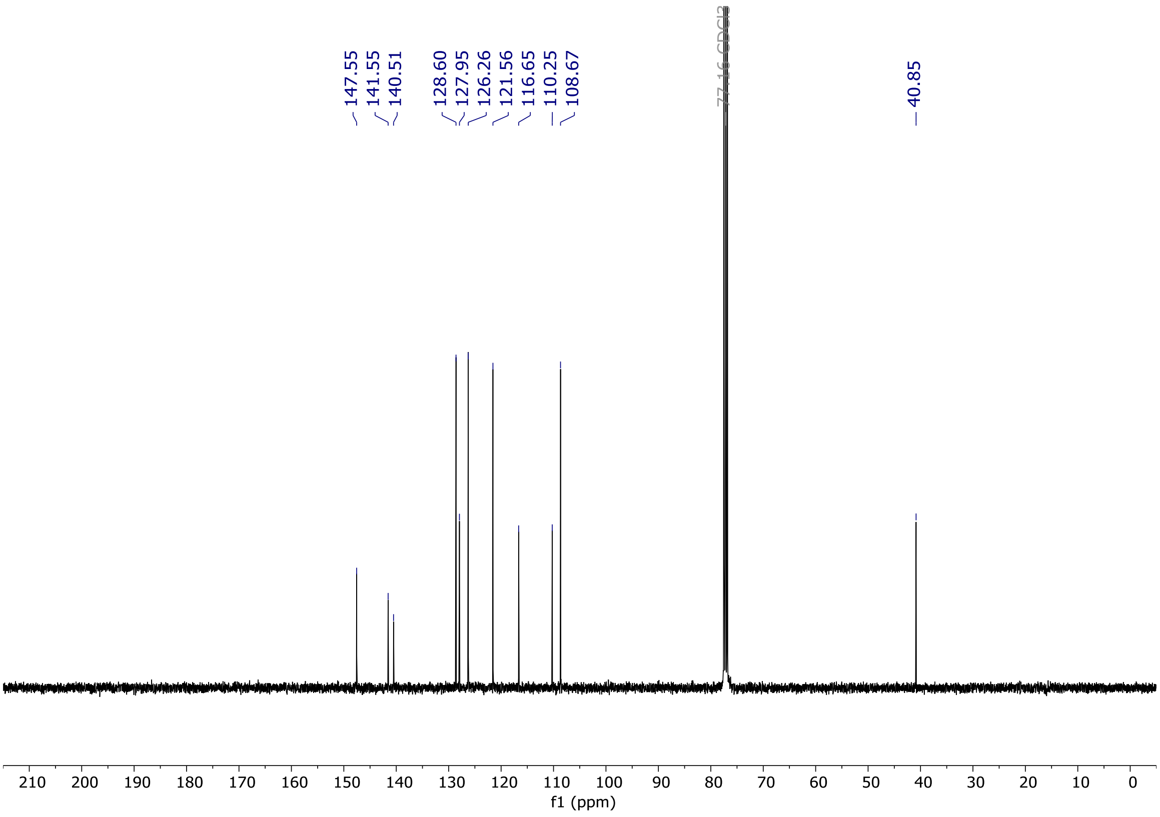

^{1}H NMR (400 MHz, $CDCl_3$) of compound **60**

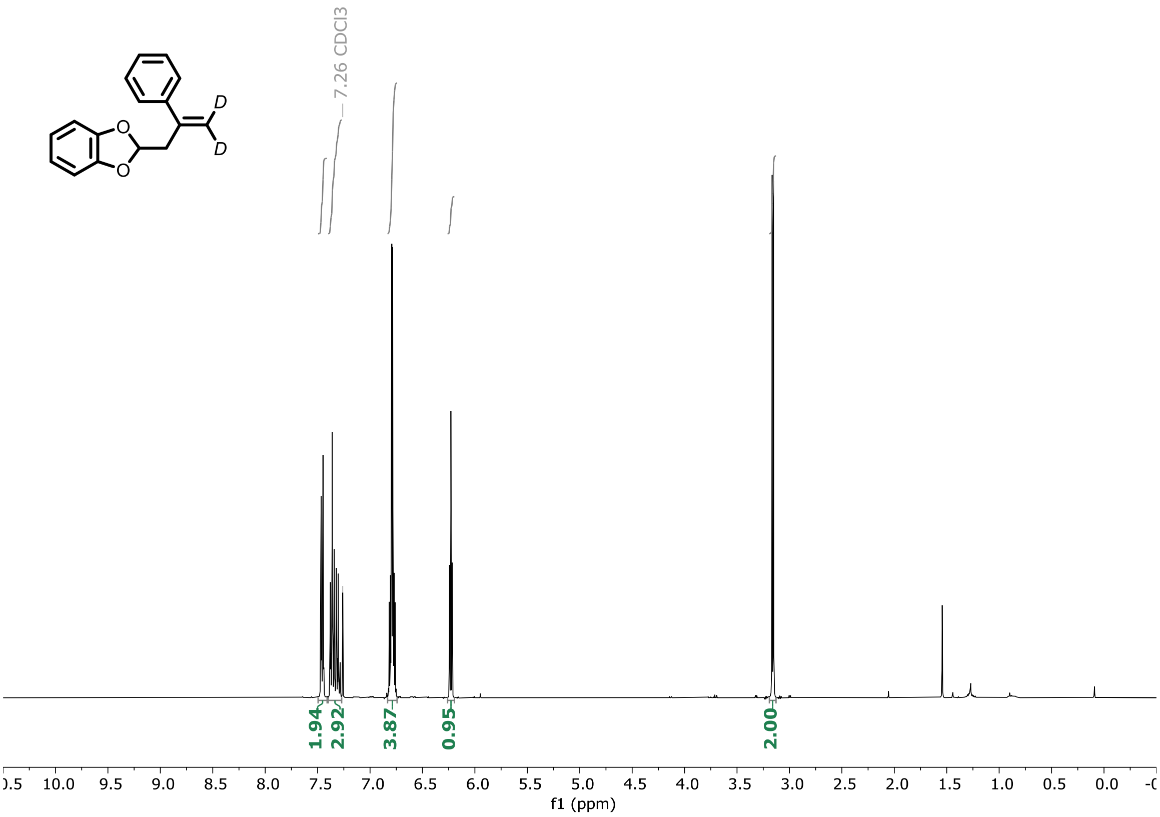

^{13}C NMR (101 MHz, $CDCl_3$) of compound **60**

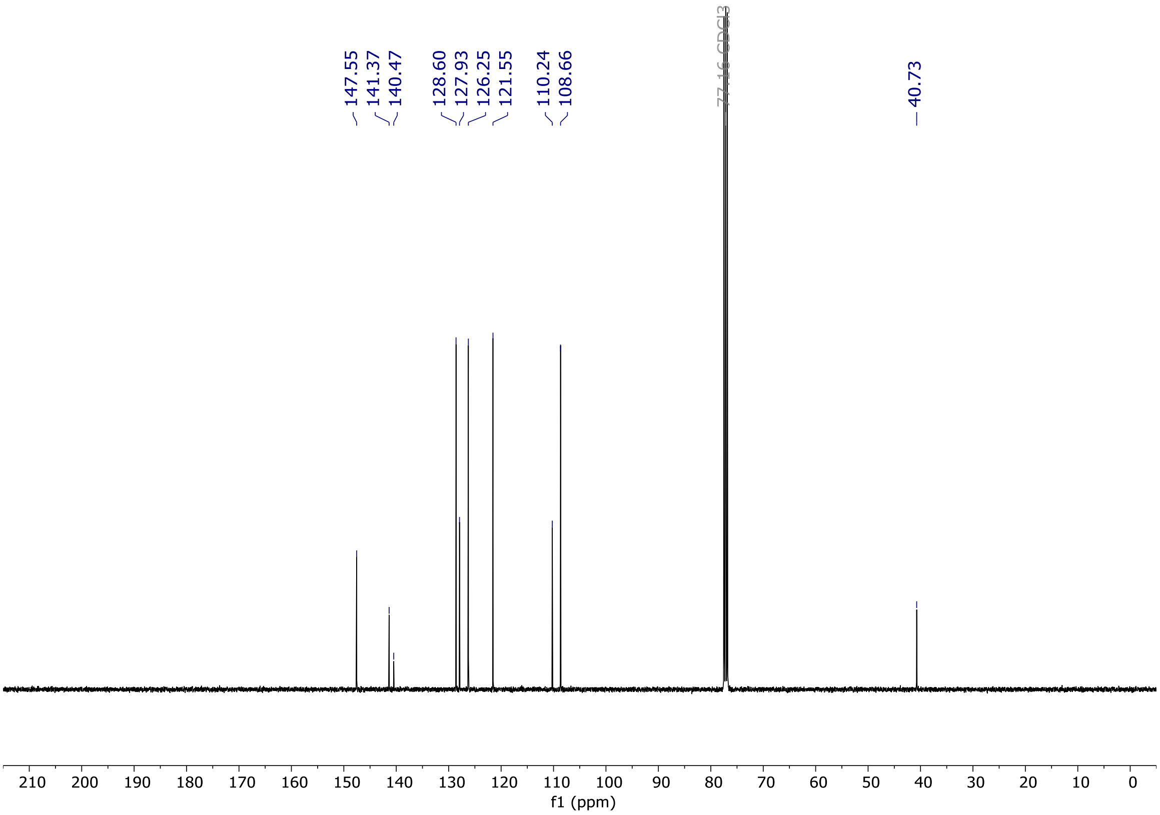

^{1}H NMR (300 MHz, $CDCl_3$) of compound **61**

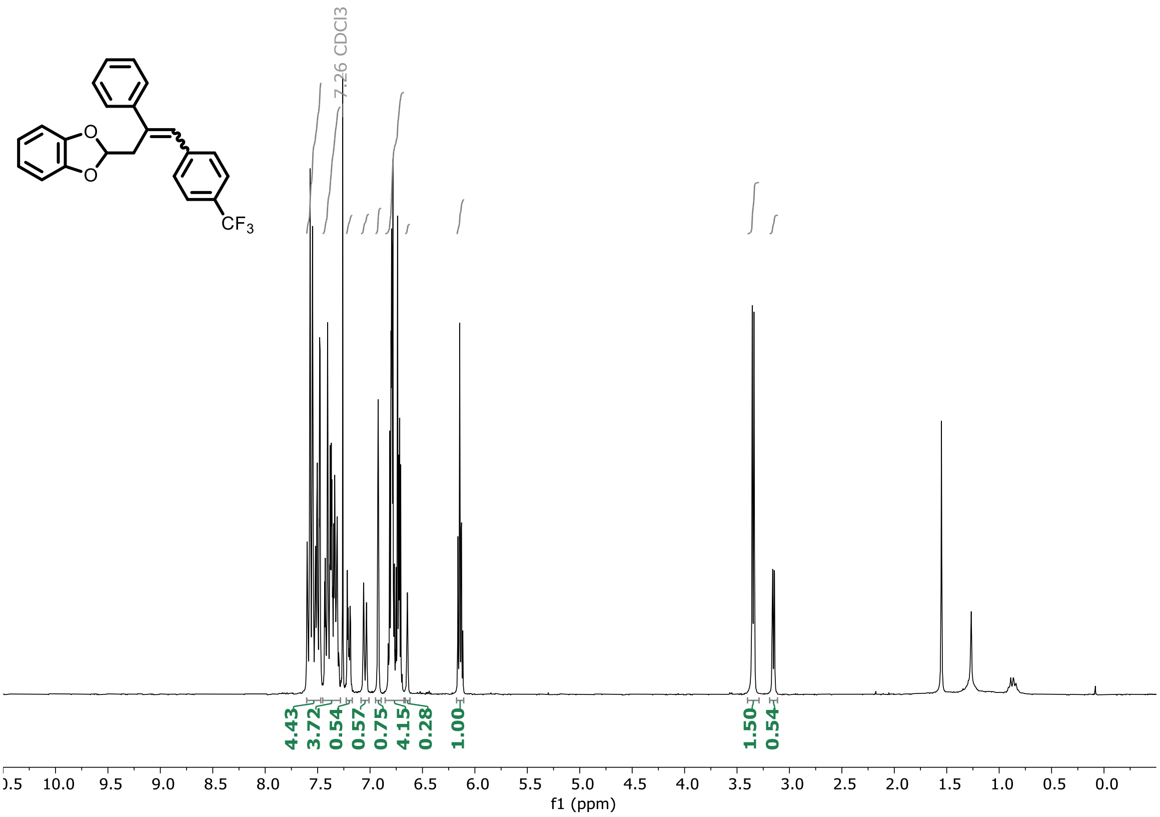

^{13}C NMR (76 MHz, $CDCl_3$) of compound **61**

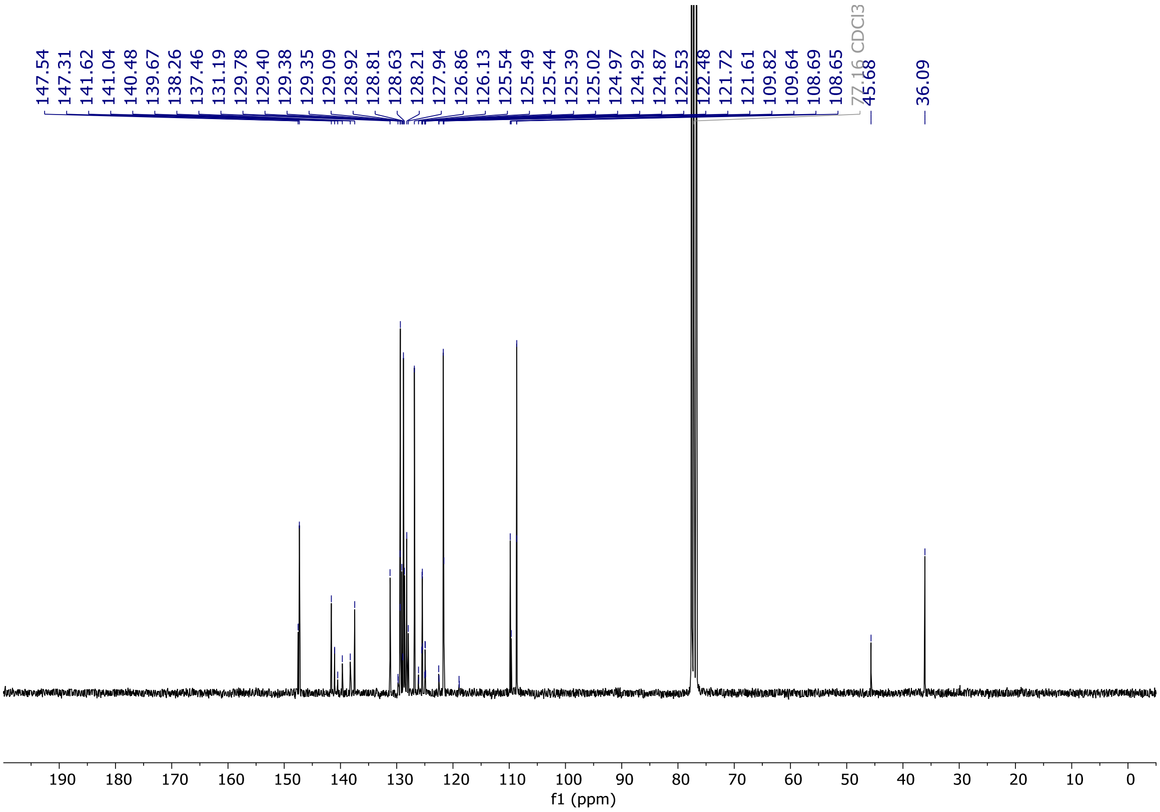

^{19}F NMR (282 MHz, $CDCl_3$) of compound **61**

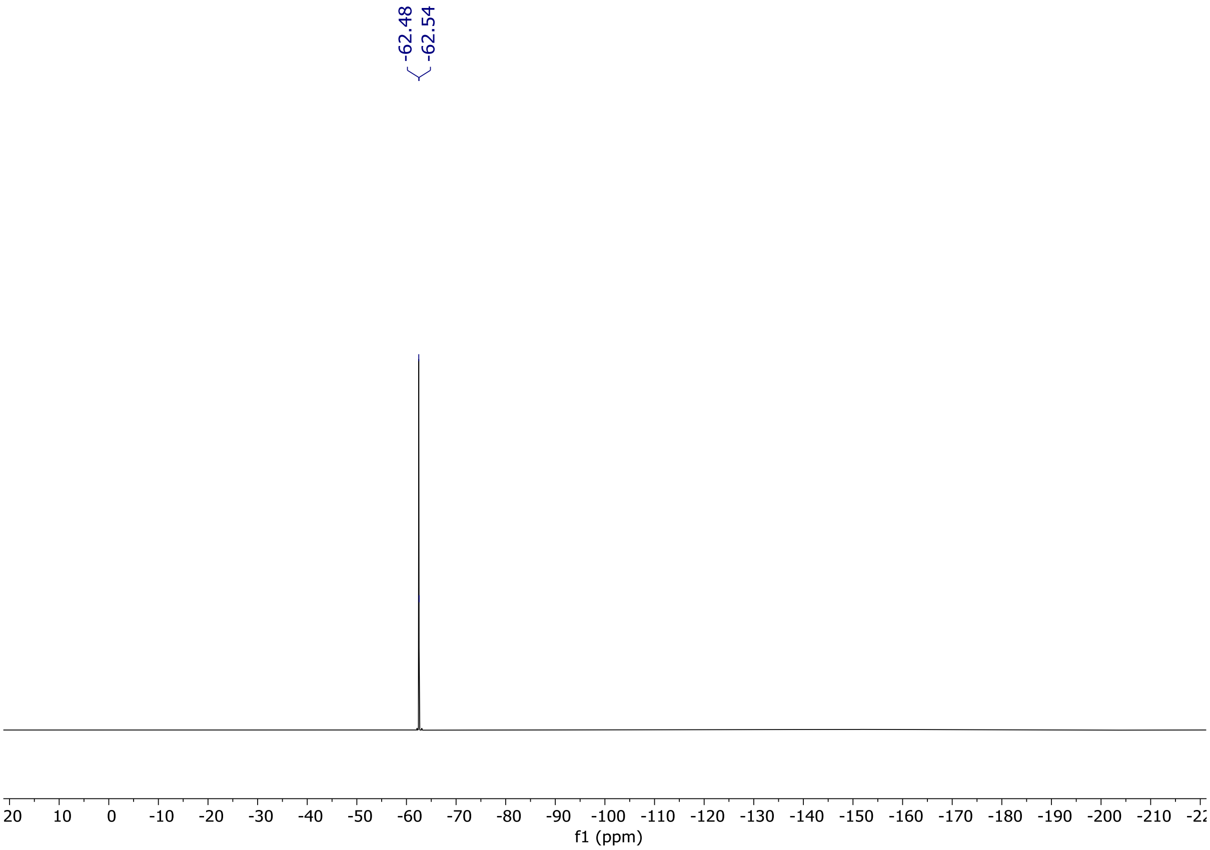

^{1}H–^{1}H NOESY (300 MHz, $CDCl_3$) of compound **61**

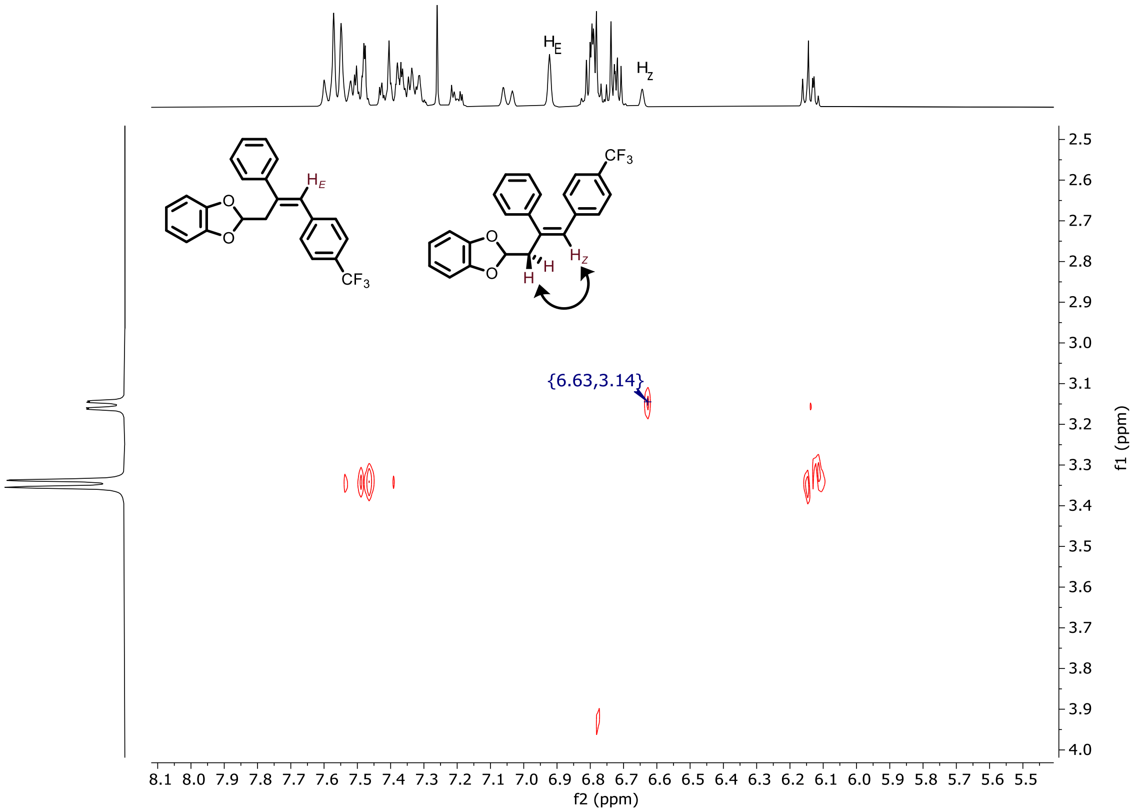

^{1}H NMR (400 MHz, $CDCl_3$) of compound **62**

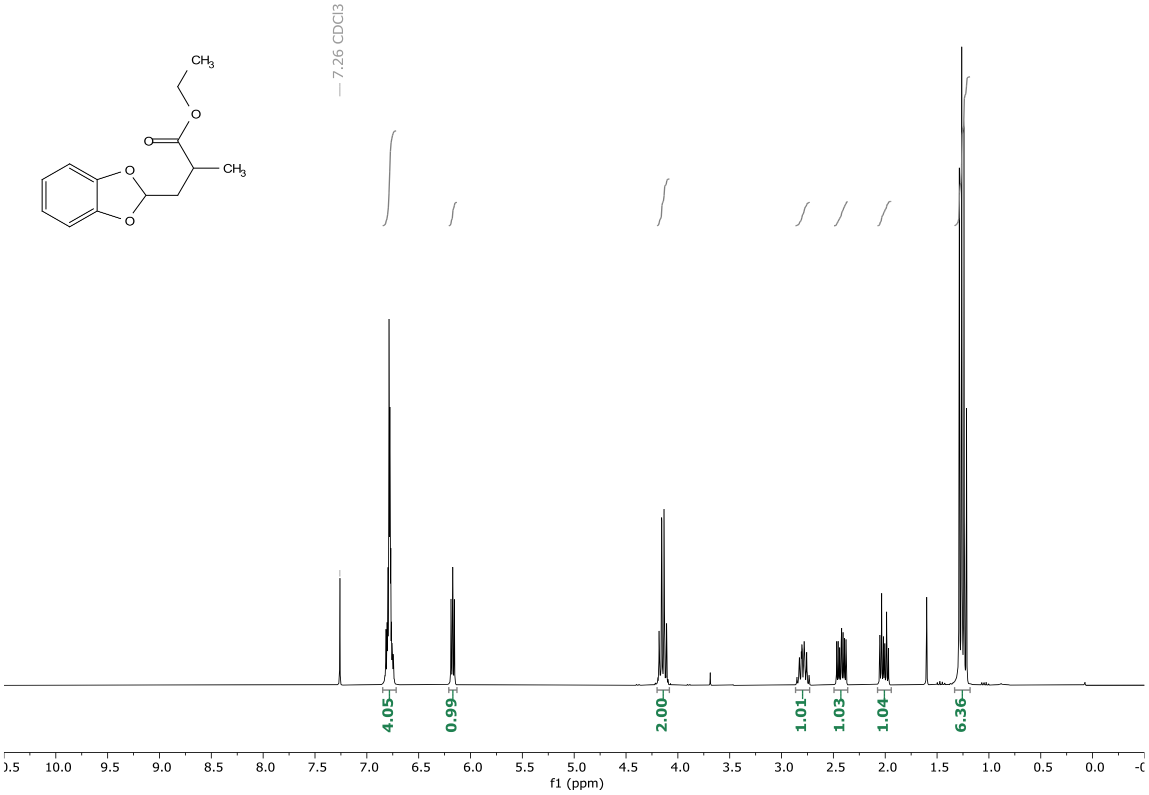

^{13}C NMR (101 MHz, $CDCl_3$) of compound **62**

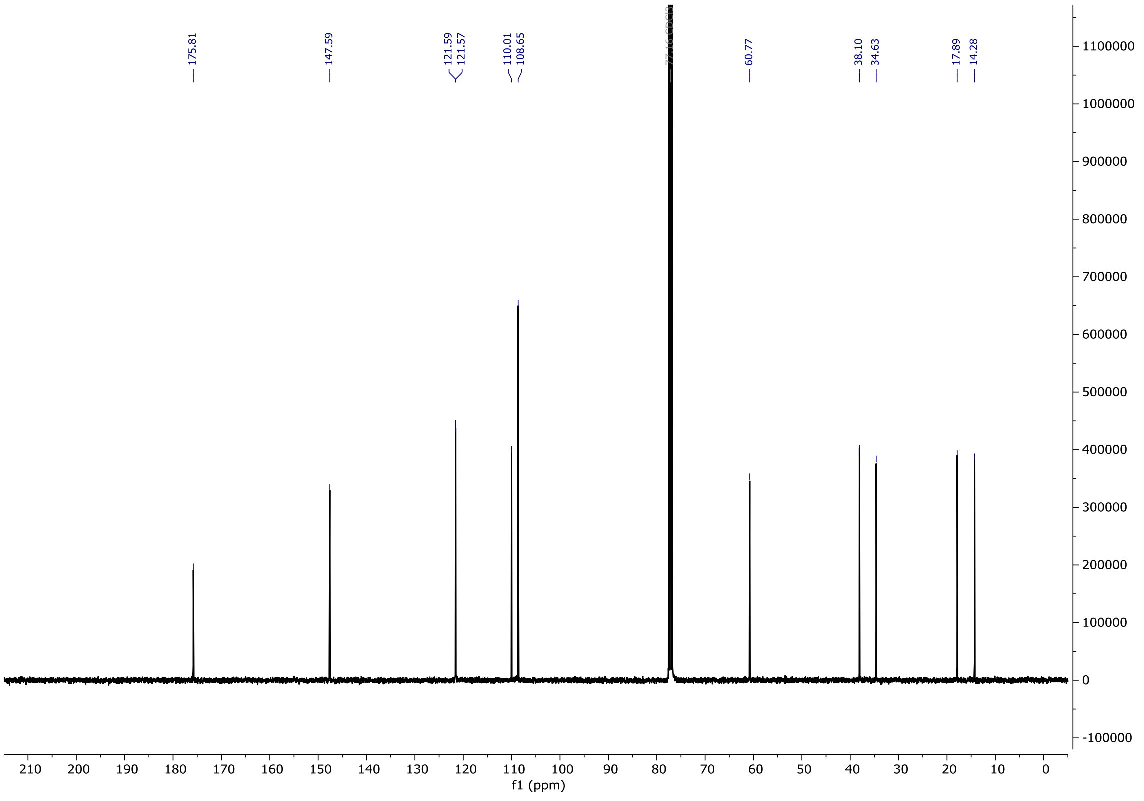

^1H NMR (400 MHz, $CDCl_3$) of compound **63**

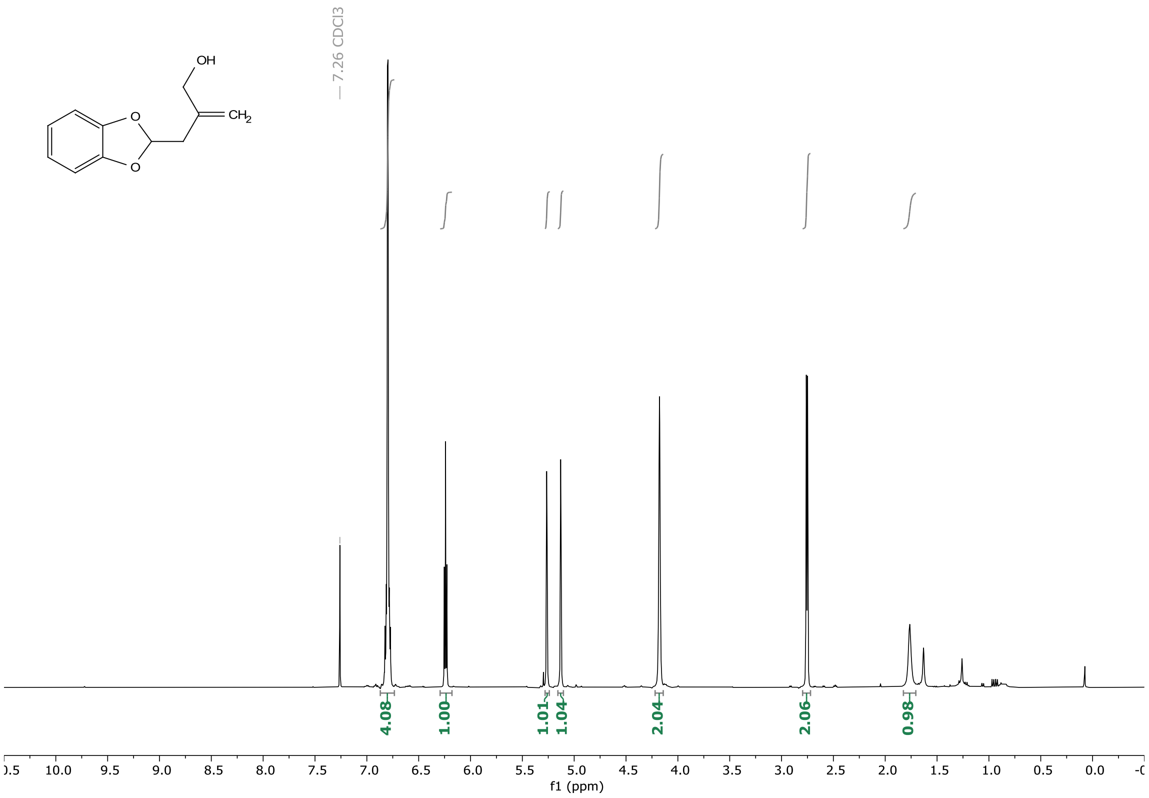

^{13}C NMR (100 MHz, $CDCl_3$) of compound **63**

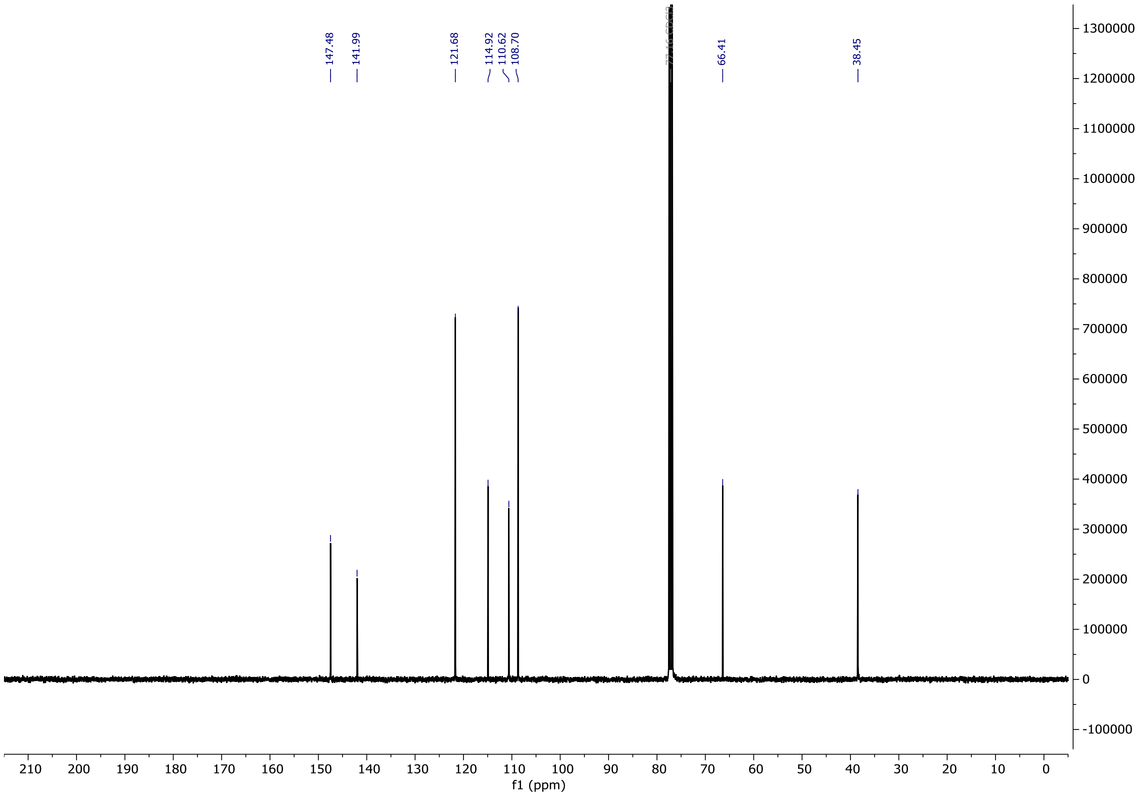

^{1}H NMR (400 MHz, $CDCl_3$) of compound **64**

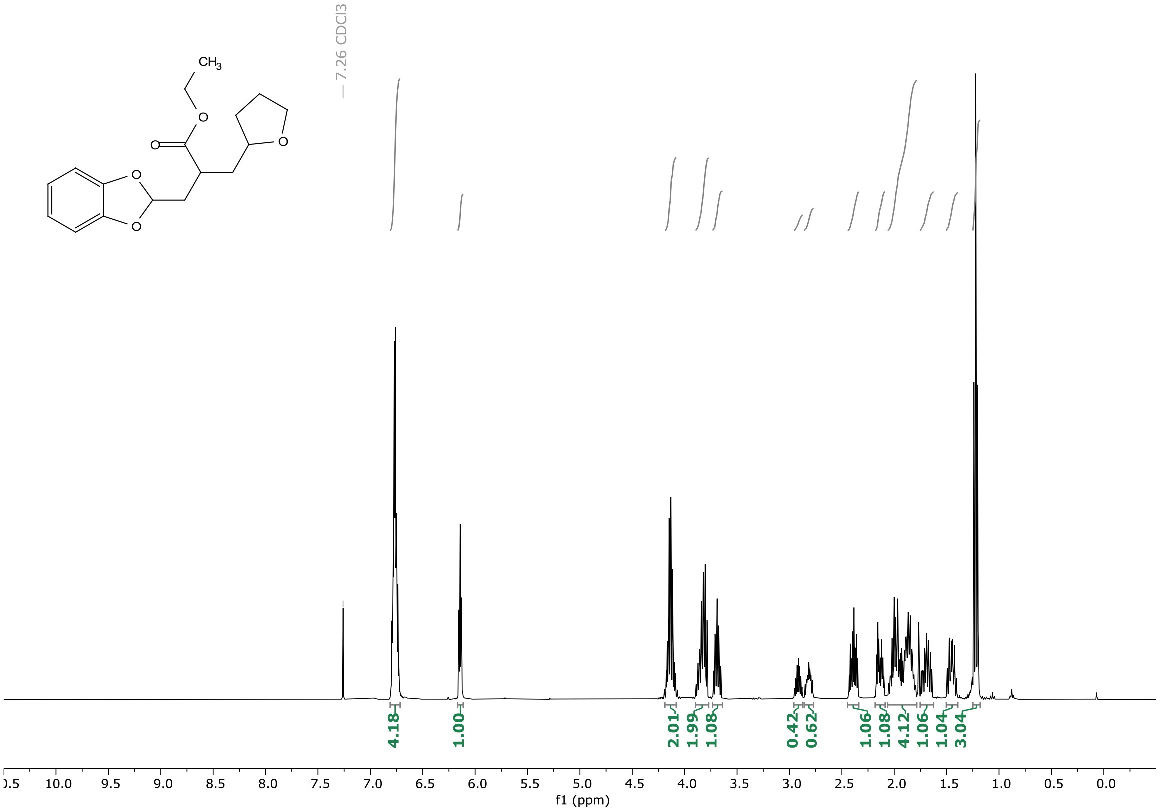

^{13}C NMR (101 MHz, $CDCl_3$) of compound **64**

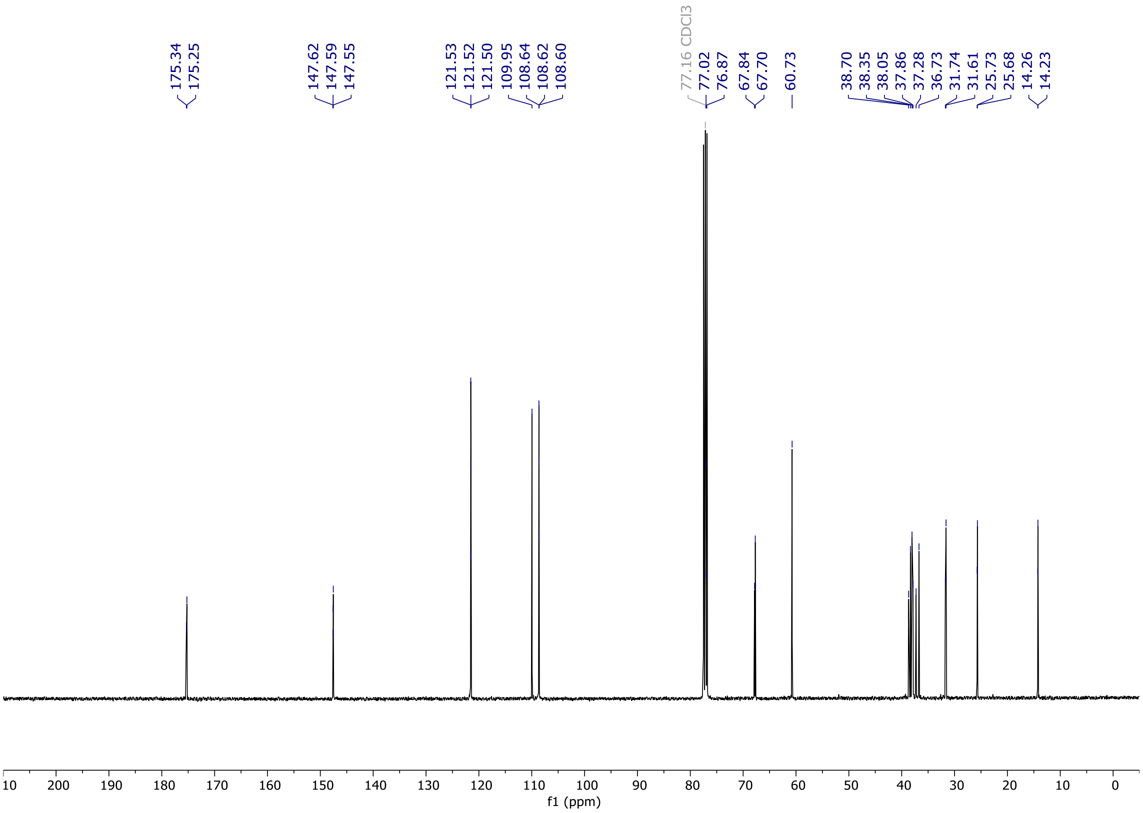

HSQC of compound **64**

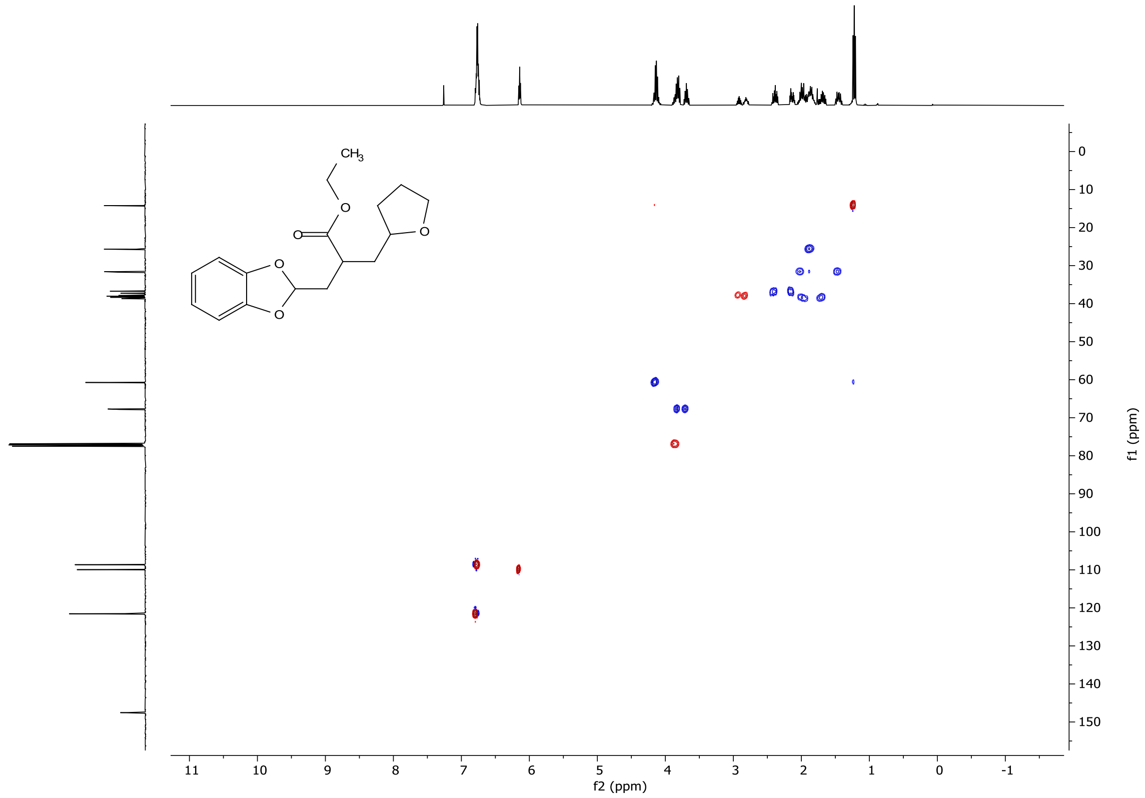

DEPT (101 MHz, $CDCl_3$) of compound **64**

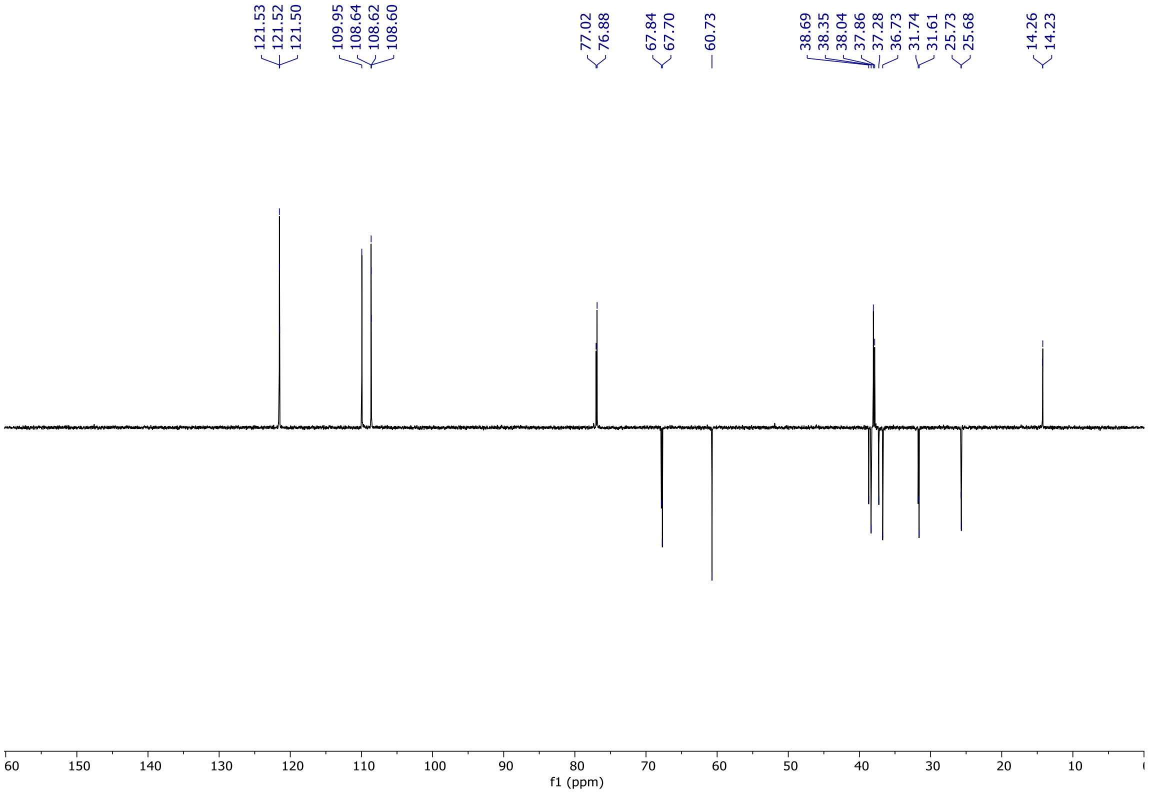

^{1}H NMR (400 MHz, $CDCl_3$) of compound **65** (major, E)

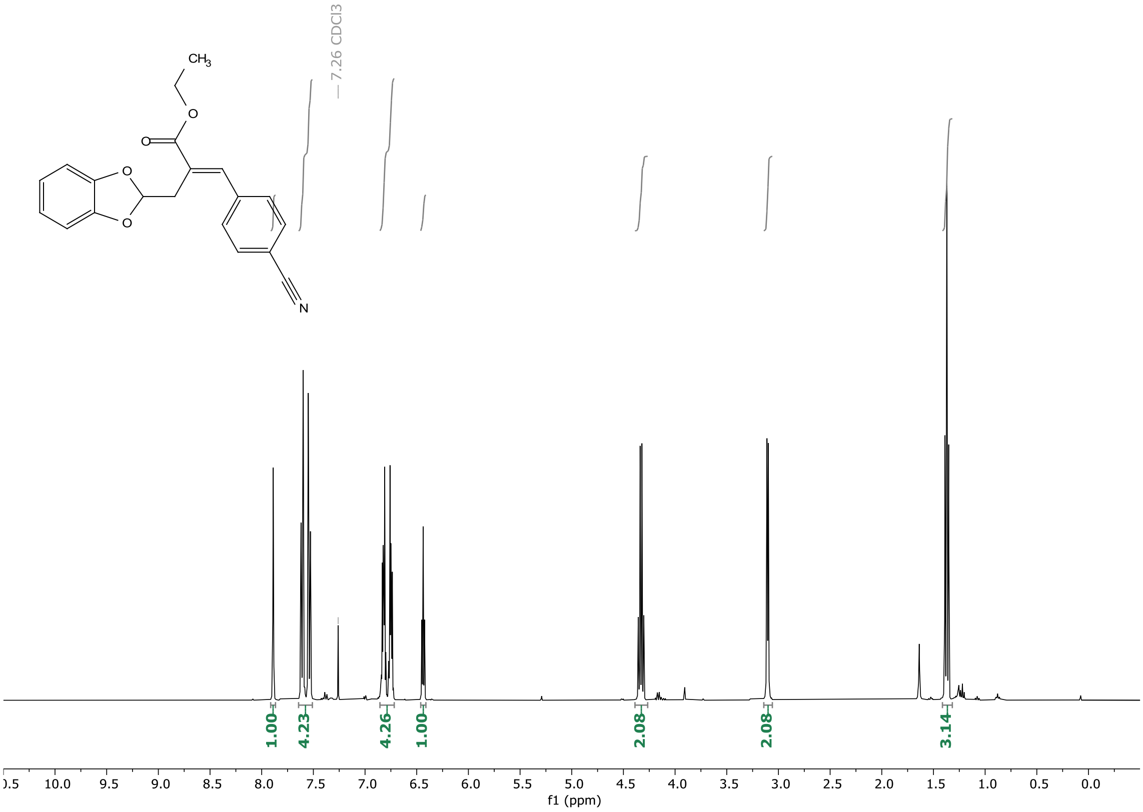

^{13}C NMR (101 MHz, $CDCl_3$) of compound **65** (major, E)

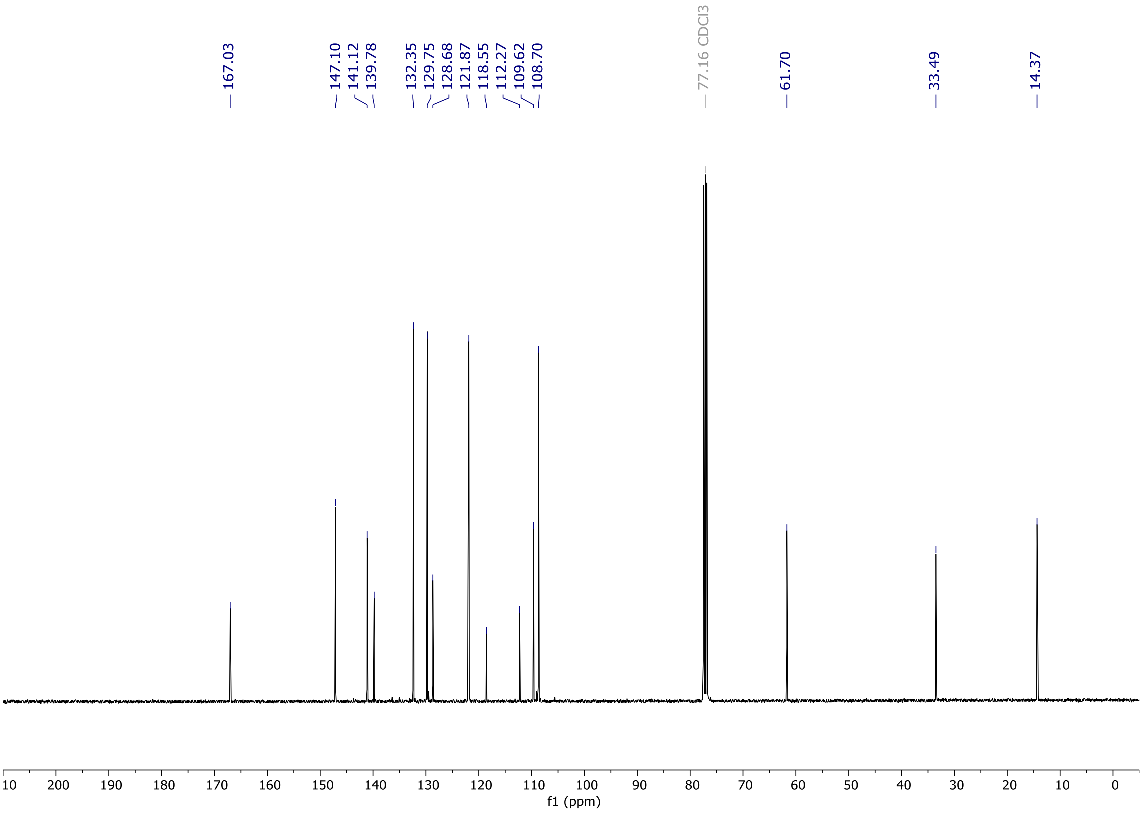

^{1}H–^{1}H NOESY (400 MHz, $CDCl_3$) of compound **65**

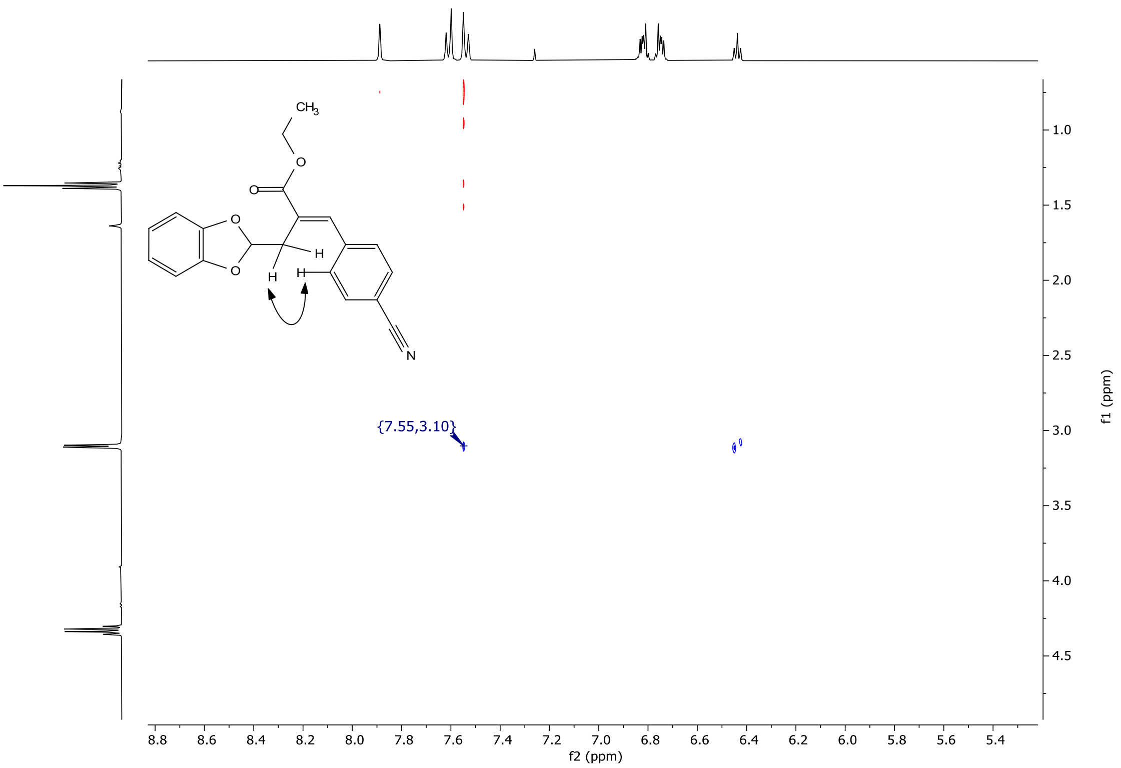

Supplement: SC-013-D2SC01581A-s001 [file SC-013-D2SC01581A-s001.pdf]
